# (Q)SAR Models of HIV-1 Protein Inhibition by Drug-Like Compounds

**DOI:** 10.3390/molecules25010087

**Published:** 2019-12-25

**Authors:** Leonid A. Stolbov, Dmitry S. Druzhilovskiy, Dmitry A. Filimonov, Marc C. Nicklaus, Vladimir V. Poroikov

**Affiliations:** 1 Laboratory of Structure-Function Based Drug Design, Institute of Biomedical Chemistry, 10 bldg. 8, Pogodinskaya str., 119121 Moscow, Russia; stolbovla@yandex.ru (L.A.S.); dmitry.druzhilovsky@ibmc.msk.ru (D.S.D.); dmitry.filimonov@ibmc.msk.ru (D.A.F.); 2 Computer-Aided Drug Design Group, Chemical Biology Laboratory, Center for Cancer Research, National Cancer Institute, Frederick, MD 21702, USA; mn1ahelix@gmail.com

**Keywords:** HIV-1, protease, reverse transcriptase, integrase, inhibitors, (Q)SAR models, GUSAR, PASS, SAVI library, virtual screening

## Abstract

Despite the achievements of antiretroviral therapy, discovery of new anti-HIV medicines remains an essential task because the existing drugs do not provide a complete cure for the infected patients, exhibit severe adverse effects, and lead to the appearance of resistant strains. To predict the interaction of drug-like compounds with multiple targets for HIV treatment, ligand-based drug design approach is widely applied. In this study, we evaluated the possibilities and limitations of (Q)SAR analysis aimed at the discovery of novel antiretroviral agents inhibiting the vital HIV enzymes. Local (Q)SAR models are based on the analysis of structure–activity relationships for molecules from the same chemical class, which significantly restrict their applicability domain. In contrast, global (Q)SAR models exploit data from heterogeneous sets of drug-like compounds, which allows their application to databases containing diverse structures. We compared the information for HIV-1 integrase, protease and reverse transcriptase inhibitors available in the EBI ChEMBL, NIAID HIV/OI/TB Therapeutics, and Clarivate Analytics Integrity databases as the sources for (Q)SAR training sets. Using the PASS and GUSAR software, we developed and validated a variety of (Q)SAR models, which can be further used for virtual screening of new antiretrovirals in the SAVI library. The developed models are implemented in the freely available web resource AntiHIV-Pred.

## 1. Introduction

Discovery of novel pharmaceutical agents is always based on existing knowledge about the mechanisms of the disease—molecular targets that should be affected to normalize the pathological process and ligands that may interact with the targets of interest [1]. Currently, no investigational drug is studied without some contribution of in silico methods [2]. When information about the three-dimensional structure of the target is known, Structure-Based Drug Design (SBDD) methods are applied for virtual screening in databases of available samples or de novo design of potential ligands with the subsequent synthesis and testing of activity in vitro. Despite the recognized limitations of SBDD methods [3], dozens of newly launched drugs have been developed using this approach [4], which is also utilized to overcome a target’s drug resistance [5]. Historically, the first computational methodology applied to research and development of new drugs was Ligand-Based Drug Design (LBDD) [6,7]. Starting from the analysis of Quantitative Structure–Activity Relationships (QSAR) within the same chemical series [8], modern LBDD methods analyze heterogeneous training sets allowing prediction of many biological activities using the same set of descriptors and the same mathematical method [9].

Current antiretroviral therapy does not provide the complete cure of the infected patients, exhibits severe adverse effects, and leads to the appearance of resistant strains [10]. Thus, the discovery of new anti-HIV medicines remains an essential task. Ligand-based drug design methods are widely used in finding [11] and optimization [12] of new anti-HIV agents. Many (Q)SAR studies have been dedicated to the analysis of structure–activity relationships for particular classes of compounds, such as tetrahydroimidazobenzodiazepines [13] and hydroxyethoxymethylphenylthiothymines [14]. The applicability domain of the local (Q)SAR models developed with training sets including molecules from a single chemical class is typically limited to this particular chemical class. Recent generation of very large virtual compound libraries containing hundreds of millions of molecules with presumably many new chemotypes [15,16] requires the development of global (Q)SAR models, which are characterized by a wide applicability domain. Such models can be built using publicly and commercially available large-scale sources of information about antiretroviral agents including the NIAID HIV/OI/TB Therapeutics [17], EBI ChEMBL [18], and Clarivate Analytics Integrity [19] databases. It is well-known that in any particular drug discovery project, careful curation of the information from the databases is needed [20,21,22]. Thus, our study consisted of the preparation of cleaned versions of the training sets on HIV-1 integrase (IN), protease (PR), and reverse transcriptase (RT) inhibitors; creation and validation of (Q)SAR models using the PASS [23] and GUSAR [24] software; investigation of the possibilities and limitations of practical application of the developed models; and creation of a freely available web service for prediction of anti-HIV activity using the integrated training set. 

## 2. Results

### 2.1. Training Sets

**Figure 1:**
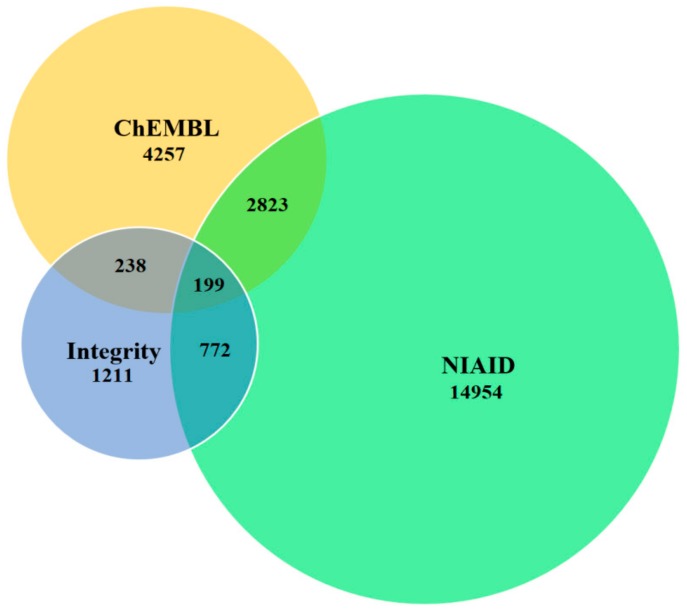
Total number of molecules exported from three databases and intersection between different datasets after the cleaning procedure.

We exported the data for HIV-1 IN, PR, and RT inhibitors from the NIAID, ChEMBL, and Integrity databases. During the cleaning procedure (see the Materials and Methods section) about 40% of initial data entries were excluded. The numbers of inhibitors in each dataset before and after the cleaning procedure are given in Table 1. Comparison of the total contents of the cleaned datasets shows that 2823, 772, and 238 molecules are in the overlap sets of NIAID with ChEMBL, NIAID with Integrity, and ChEMBL with Integrity, respectively (Figure 1). Only 199 molecules were found to be in all three datasets. Thus, for creating (Q)SAR models one may use as training sets the combined sets from three pairs of databases and then validate these models on the basis of independent test sets representing the unique part of the third database. For each of IN, PR, and RT inhibitors we developed two models using as the training set: NIAID and ChEMBL and NIAID and Integrity. The combined training sets were obtained by the further addition of the non-overlapping part of the second dataset to the core part represented by the NIAID dataset. The ChEMBL and Integrity combination was not used for the training procedure because in this case the chemical space of the training set would have been much smaller than that of the test set. An overall training set including data from all three databases was used for development of the (Q)SAR models with the broadest coverage of chemical space. These (Q)SAR models were used for creating the web application that predicts anti-HIV activity. 

### 2.2. Classification Models

Classification models were developed using the PASS software (see the Materials and Methods section for details). To develop the SAR models, compounds from the three databases were categorized as “active” if IC_50_ < 1000 nM and as “inactive” otherwise. Characteristics of the training set and Invariant Accuracy of Prediction (IAP) obtained by leave-one-out cross-validation are given in Table 2.

Table 2 shows that IAP values are comparable for all three training sets. The best results were achieved for HIV-1 PR inhibitors (IAP is about 0.94); for HIV-1 IN inhibitors IAP is about 0.92; for HIV-1 RT inhibitors IAP is about 0.88.

### 2.3. Quantitative Structure–Activity Relationships (QSAR) Models

**Figure 2:**
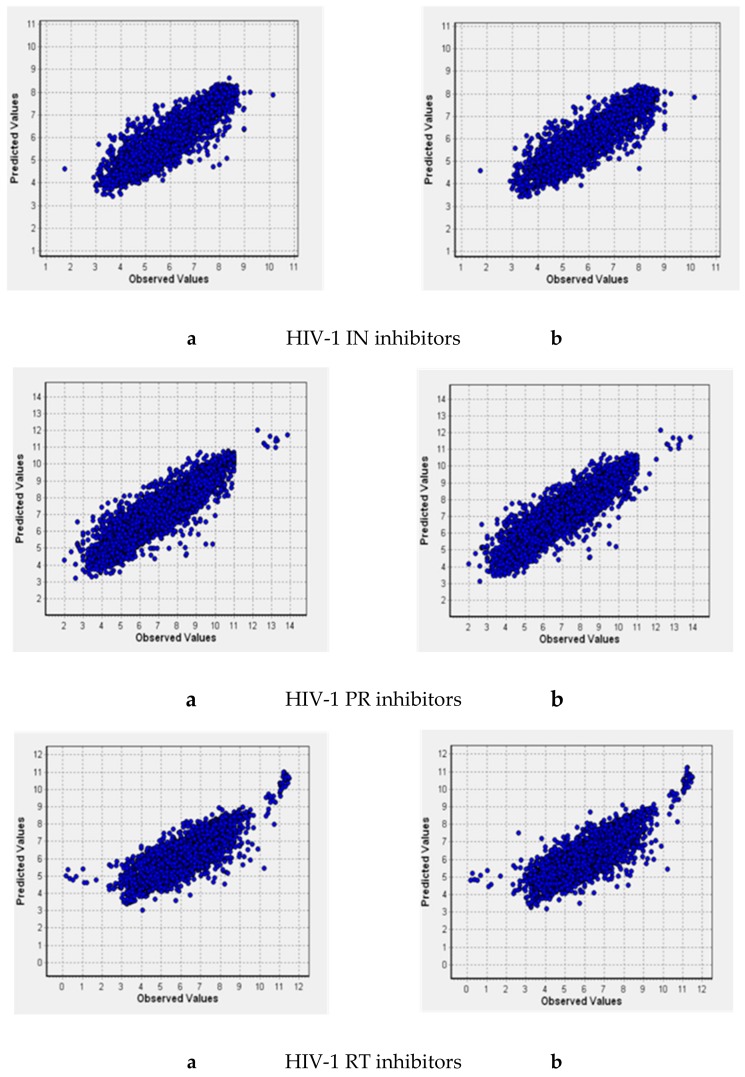
Predicted pIC50 vs. observed values for QSAR models based on NIAID and ChEMBL (**a**) and NIAID and Integrity (**b**) datasets.

Combination of the predictions obtained with the classification models with those obtained by regression QSAR models is needed to restrict the studied chemical space and identify the most potent inhibitors that act at low concentrations. Thus, in addition to the PASS models for six training sets, we built QSAR models using the GUSAR software (see the Materials and Methods section for details). Their characteristics are given in Table 3 and Figure 2. Here, N is the number of compounds in the training set; R^2^ is the coefficient of determination; Q^2^ is the cross-validated R^2^; RMSD is the root-mean-square deviation; V is the number of degrees of freedom.

The developed QSAR models are based on the large training sets with a broad chemical diversity. Comparison of characteristics of the models obtained before and after the cleaning procedure led to the conclusion that, due to the appropriate data selection, the reasonable accuracy and predictivity is achieved (see the Supplementary Materials). To check the models’ performance, we carried out the validation procedures.

### 2.4. Model Validation Using the External Test Sets

**Figure 3:**
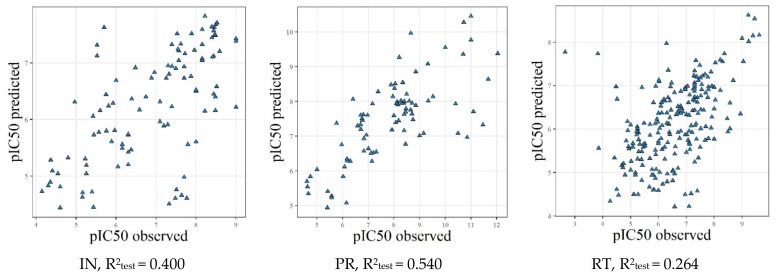
Results of prediction for the Integrity test sets with the GUSAR regression models.

**Figure 4:**
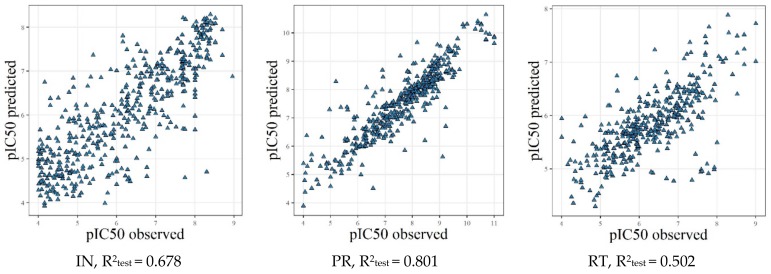
Results of prediction for the ChEMBL test sets with the GUSAR regression models.

To evaluate the predictivity of the built QSAR models, we prepared independent test sets. Estimation of the quality of the combined ChEMBL and NIAID models was performed using the Integrity test sets using those structures that did not overlap with ChEMBL or NIAID datasets. Estimation of the quality of the combined Integrity and NIAID models was performed using ChEMBL test sets with the structures that did not overlap with Integrity or NIAID datasets. Results of prediction for these test sets are summarized in Table 4 and Figure 3 and Figure 4.

As can be seen from the obtained results, the validation outcomes depend on the particular HIV-1 target, the model’s characteristics, and the test set.

For the classification models, the sensitivity parameter varies from 0.596 (RT, NIAID and Integrity training set vs. ChEMBL test set) to 0.826 (PR, NIAID and Integrity training set vs. ChEMBL test set). Specificity parameter varies from 0.620 (RT, NIAID and ChEMBL training set vs. Integrity test set) to 0.867 (RT, NIAID and Integrity training set vs. ChEMBL test set). The balanced accuracy varies from 0.615 (RT, NIAID and Integrity training set vs. ChEMBL test set) to 0.817 (IN, NIAID and Integrity training set vs. ChEMBL test set). The worst values for all three parameters were obtained for RT (NIAID and ChEMBL training set vs. Integrity test set). For all three enzymes, the balanced accuracy is relatively low for the Integrity test set, which could be explained by the peculiarities of chemical space in this database. Since the commercially available database Integrity is updated permanently, test set may contain some compounds that are not included in the other two databases yet. In general, the results of validation versus independent test sets are satisfactory; thus, the classification models could be applied for virtual screening. 

For the regression models obtained using NIAID and Integrity data as the training set and ChEMBL data as the test set, the R^2^_test_ values were 0.502 (RT), 0.678 (IN), and 0.801 (PR). When we used NIAID and ChEMBL data as the training set and Integrity data as the test set, the R^2^_test_ values were 0.264 (RT), 0.400 (IN) and 0.540 (PR). As was mentioned above, such poor performance can be explained by the peculiarities of chemical space in the Integrity compared to the NIAID and ChEMBL databases. Relatively low performance obtained for RT inhibitors indicates the probable existence of more complex relationships (differentiation on NNRTI and NRTI inhibitors and different binding sites), which are not reflected by the current QSAR models.

### 2.5. (Q)SAR Models Based on the Complete Dataset

**Figure 5:**
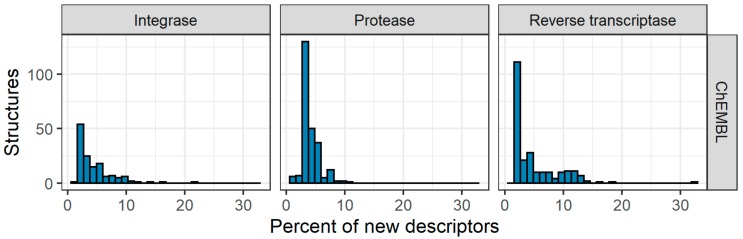
Distributions of new MNA descriptors for the ChEMBL test sets.

Since the datasets used for the training always cover only the part of the overall chemical space from large databases, we decided to estimate the fraction of novel MNA descriptors when ChEMBL data were used as the test set, and NIAID and Integrity data were used as the training set. The results are given in Figure 5.

As one can see from Figure 5, for all three targets there are some novel MNA descriptors that are not present in the training set. For some IN and RT inhibitors, their fraction exceeds 15%, which is considered as a cutoff value for Applicability Domain in classification models obtained with PASS [25]. However, even 10% of novel MNA descriptors in the analyzed molecule reduce the reliability of prediction. For PR inhibitors, the total number of novel MNA descriptors was 94, and two compounds contained more than 10% such descriptors. This lines up with the best performance of the (Q)SAR models for PR inhibitors. For IN inhibitors, the total number of novel MNA descriptors was 220. Seven compounds contained more than 10% such descriptors, and in two cases, this number exceeded 15%. For RT inhibitors, the total number of novel MNA descriptors was 119. A total of 38 compounds contained more than 10% such descriptors, and in three cases this number exceeded 15%. This lines up with the worst performance of the (Q)SAR models for RT inhibitors (Table 2). 

For regression models obtained by GUSAR, whether the analyzed compounds are in the applicability domain is estimated during the prediction. When NIAID and Integrity data were used as the training set and ChEMBL data as the test set, 14, 17, and 25 compounds fell out of the applicability domain for IN, PR, and RT, respectively. This again agrees with the worst performance of the (Q)SAR models for RT inhibitors (Figure 4).

To widen coverage of chemical space, it stands to reason that the overall training set should include molecules from all three databases. Characteristics of the classification models obtained with compounds from all three databases are given in Table 5. These data show that the IAP values obtained in leave-one-out cross-validation and in 20-fold cross-validation are very close. Thus, the models had good accuracy and predictivity.

**Figure 6:**
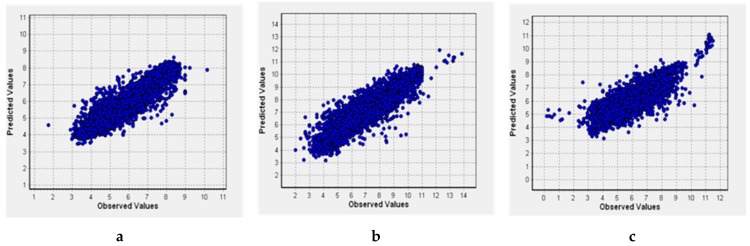
IN (**a**), PR (**b**), and RT (**c**) pIC50 observed vs. predicted values for the (Q)SAR models based on the complete dataset.

In addition to the classification models, using the GUSAR software, we built the QSAR models with NIAID and ChEMBL and Integrity as a training set. Characteristics of the regression models obtained with compounds from all three databases are given in Table 6. This shows that R^2^, Q^2^, and RMSD values are quite reasonable; thus, the models had good accuracy and predictivity. Distributions of the observed vs. predicted values are shown in Figure 6.

### 2.6. Implementation of (Q)SAR Models in Web-Service

**Figure 7:**
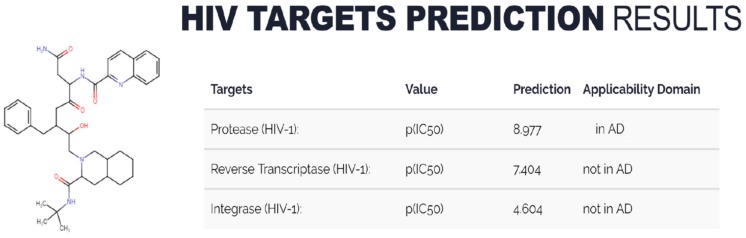
Example of prediction for an FDA-approved inhibitor by the AntiHIV-Pred web-service.

(Q)SAR models developed on the basis of the complete training sets (see Section 2.5) were implemented in the AntiHIV-Pred web resource ([26], http://www.way2drug.com/hiv/). This service, which is freely available to the scientific community, can be used as a tool for in silico selection of potential agents for treatment of HIV-infection and its comorbidities (Figure 7).

The user draws a chemical structure and chooses the particular HIV target of interest. Prediction of relevant biological activities is based on the structural formulae of drug-like molecules and can be obtained for single-component, uncharged structures with a molecular weight of less than 1250 Da and including at least 3 carbon atoms. Description of the AntiHIV-Pred functionality has been presented in a recent application note [27]. (Q)SAR models developed in the framework of the current study have been added to the initial version of web resource, which was based on the ChEMBL dataset models only. The user may select one of five HIV protein targets in AntiHIV-Pred and get prediction of the inhibiting activity for the analyzed molecule. For HIV IN, PR, and RT the user may choose both preferable quantitative (GUSAR) or classification (PASS) models and desired training set covering the particular part of chemical space (see above) to get predictions. Models based on distinct database sets are also available. For HIV REV an HIV TAT only ChEMBL based models are available, though the expansion of full AntiHIV-Pred functionality for these two targets is planned.

## 3. Discussion

Using data on HIV-1 IN, PR, and RT inhibitors from NIAID DB, ChEMBL, and Integrity we have developed classification and regression models with PASS and GUSAR, respectively. Validation of the built (Q)SAR models demonstrated reasonable accuracy and predictivity. It is necessary to stress that applying (Q)SAR models to large databases with highly diverse structures (e.g., SAVI [15]) requires (Q)SAR models based on the maximum diversity of available information. Validation of predictivity for such models could be performed using 20-fold cross-validation because all available information was included in the training set. Reasonable performance of the obtained models was shown; thus, they may be used for virtual screening.

**Figure 8:**
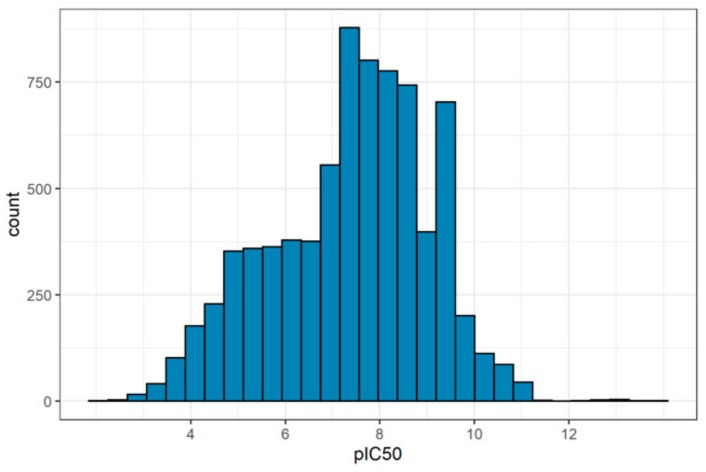
Activity distribution in the NIAID PR dataset.

In spite of the developed models having reasonable characteristics of performance, the datasets used as the training set have a problem. The distributions of activity data in all training sets are biased toward highly active compounds. Figure 8 shows the distribution for NIAID PR dataset as an example. 

A similar situation was observed for the other training sets. Internal or external validation of the models does not reveal any problem because the structures of the test sets are rather similar to those of the training sets. However, quantitative predictions obtained using our web-service for large and diverse databases may be incorrectly biased towards high activity because the training sets with quantitative data are biased towards the high active substances. This problem is solved by classification models that could be recommended as the first choice for prediction [28]. 

## 4. Materials and Methods 

### 4.1. Sources of Information for Preparation of the Training Sets

#### 4.1.1. NIAID HIV/OI/TB DB Dataset

NIAID ChemDB HIV, Opportunistic Infection and Tuberculosis Therapeutics Database (NIAID DB) is the publicly available database containing data extracted from scientific literature on the structure and activity of compounds tested against HIV, HIV enzymes, or opportunistic pathogens. It is created and supported by the National Institute of Allergic and Infectious Diseases, NIH. To analyze the structure–activity relationships, we extracted from this database information about structure and antiretroviral activity of 10,377, 7604, and 8936 molecules tested as HIV-1 IN, PR and RT inhibitors, respectively.

#### 4.1.2. ChEMBL Datasets

ChEMBL is the publicly available database containing information about structure and biological activity of drug-like molecules created and supported by the European Bioinformatics Institute–European Molecular Biology Laboratory (EMBL-EBI) for the past ten years. In the current study, we used ChEMBL version 24, which includes data on about 20,000 compounds, interactions with seven HIV-1 molecular targets, and more than 50,000 records with information about the associated bioactivities. To analyze the structure–activity relationships, we extracted information about structure and antiretroviral activity of 2283, 2387, and 2149 molecules tested as HIV-1 IN, PR and RT inhibitors, respectively, from the database.

#### 4.1.3. Integrity Dataset

Integrity is a commercially available database provided by Clarivate Analytics. To analyze the structure–activity relationships, we extracted information about structure and antiretroviral activity of 563, 316, and 731 molecules tested as HIV-1 IN, PR and RT inhibitors, respectively, from the database.

### 4.2. Data Curation Pipeline

Data curation was performed with caution relying on the modern recommendations [29] and containing three main steps:

1. Deletion of incorrect entries and standardization:

Deletion of incomplete entries and entries that are flagged as unreliable (e.g. DATA_VALIDITY_COMMENT field in ChEMBL DB) and leaving only IC_50_ entries with conversion of values to molar concentrations.

2. Chemical data curation:

Deletion or repair of entries with errors in molecular structure (charged structures, incomplete fragments, etc.), entries with metalorganics, fullerenes, structures with high molecular mass, structures with rare atoms. Deletion or repair of entries with mixtures or salts.

3. Experimental data curation:

Deletion of entries with activity values that are derived from unknown or rarely used experimental assay. Sampling experimental values that have to be checked in original data sources. Duplicates and activity cliffs handling (via each assay)—similarity values were calculated for structures in each assay, and if there were activity cliffs, the values were checked in the original source.

### 4.3. Modeling Methods

The modelling was accomplished using two types of descriptors—Multilevel Neighborhoods of Atoms (MNA) [30] and Quantitative Neighborhoods of Atoms (QNA) [24]. These descriptors are based on a representation of the molecular structure that includes hydrogens in accordance with the valences and partial charges of atoms and does not specify the types of bonds. Each of the MNA descriptors is a linear notation of an atom-centred fragment of the structure of an organic molecule. QNA descriptors are calculated based on the connectivity matrix of a molecule and the standard values of ionization potential and electron affinity of atoms in a molecule. QNA describes each of the atoms in a molecule by two real *P* and *Q* values, with each of the *P* and *Q* values depending on the whole composition and structure of a molecule.

The MNA and QNA descriptors are generated only if the molecular structure corresponds to the following usual criteria:

- Each atom must be presented by an atom symbol from the periodic table;
- Each bond must be a covalent bond presented by single, double, or triple bond types only;
- The structure must include three or more carbon atoms;
- The structure must include only one component;
- The molecule must be uncharged;
- The absolute molecular weight of the substance must be less than 1250 Da.

Biological activities in PASS are described qualitatively (“active” or “inactive”). The algorithm of activity prediction is based on a modified naïve Bayesian classifier [23]. 

GUSAR uses a self-consistent regression models building. Classical multiple linear regression has a number of limitations. In particular, it is important to use only non-collinear variables, and the number of the training examples should significantly exceed the number of independent variables. To overcome these limitations, an approach based on the statistical regularization of incorrect tasks is used in the self-consistent regression, the regularized least squares method [24].

Additional information on the modeling methods is presented in Supplementary Materials.

Widely used validation methods were used. All models were developed using 5-fold cross-validation with leave 20% out and Y-randomization procedures. External validation with an independent test set was also implemented. Information about test sets is shown in Table 7.

## Figures and Tables

**Table 1:** Numbers of HIV-1 IN, PR, and RT inhibitors exported from three databases before/after the cleaning procedure.

	IN	PR	RT
**NIAID**	10,377/3459	7604/5972	8936/5675
**ChEMBL**	2283/1430	2387/1437	2149/1390
**Integrity**	563/328	316/268	731/615

**Table 2:** Characteristics of classification models (PASS) for datasets NIAID and ChEMBL/NIAID and Integrity.

Inhibitors of	Active	Inactive	IAP
HIV-1 IN	1813/1622	2108/1930	0.924/0.922
HIV-1 PR	4762/4504	1337/1298	0.938/0.937
HIV-1 RT	3142/3054	2854/2752	0.878/0.878

**Table 3:** Characteristics of the regression models (GUSAR) for datasets NIAID and ChEMBL/NIAID and Integrity.

Inhibitors of	N	R^2^	Q^2^	RMSD	V
IN	3987/3597	0.960/0.960	0.821/0.819	0.587/0.592	384/371
PR	6462/6068	0.956/0.957	0.829/0.827	0.696/0.710	494/485
RT	6093/5894	0.943/0.942	0.723/0.715	0.760/0.776	455/441

**Table 4:** Results of prediction for the test sets with classification models.

Model	Test Set	Sensitivity	Specificity	Balanced Accuracy
IN, NIAID and ChEMBL	IN, Integrity test set	0.753	0.677	0.715
IN, NIAID and Integrity	IN, ChEMBL test set	0.813	0.820	**0.817**
PR, NIAID and ChEMBL	PR, Integrity test set	0.697	0.857	0.777
PR, NIAID and Integrity	PR, ChEMBL test set	**0.826**	0.788	0.807
RT, NIAID and ChEMBL	RT, Integrity test set	0.611	**0.620**	**0.615**
RT, NIAID and Integrity	RT, ChEMBL test set	**0.596**	**0.867**	0.732

**Table 5:** Characteristics of classification models (PASS) based on the complete dataset.

Inhibitors of	Active	Inactive	IAP LOO CV	IAP 20-fold CV
**IN**	1884	2139	0.922	0.921
**PR**	4840	1351	0.937	0.936
**RT**	3286	2935	0.876	0.875

**Table 6:** Characteristics of the regression models (GUSAR) based on the complete dataset.

Inhibitors of	N	R^2^	Q^2^	RMSD	V
**IN**	4091	0.96	0.818	0.595	392
**PR**	6554	0.954	0.824	0.709	470
**RT**	6309	0.941	0.714	0.767	452

**Table 7:** Number of compounds in the test sets.

	IN	PR	RT
**ChEMBL and NIAID**	104	92	216
**Integrity and NIAID**	494	486	415

## Supplementary Materials

The following are available online at https://www.mdpi.com/1420-3049/25/1/87/s1, Training sets, data curation pipeline, modeling methods and each part of investigation detailed.

## References

[1] Yella J.K., Yaddanapudi S., Wang Y., Jegga A.G. (2018). Changing trends in computational drug repositioning. Pharmaceuticals (Basel).

[2] Jorgensen W.L. (2004). The many roles of computation in drug discovery. Science.

[3] Chen Y.C. (2015). Beware of docking!. Trends Pharmacol. Sci..

[4] Phillips M.A., Stewart M.A., Woodling D.L., Xie Z.-R. (2018). Has molecular docking ever brought us a medicine. Molecular Docking.

[5] Tarasova O., Poroikov V., Veselovsky A. (2018). Molecular docking studies of HIV-1 resistance to reverse transcriptase inhibitors: Mini-review. Molecules.

[6] Hancsh C., Fujita T. (1964). Ro-sigma–pi analysis. A method for the correlation of biological activity and chemical structure. J. Am. Chem. Soc..

[7] Free S.M., Wilson J.W. (1964). A mathematical contribution to structure-activity studies. J. Med. Chem..

[8] Franke R. (1984). Theoretical Drug Design Methods.

[9] Mayr A., Klambauer G., Unterthiner T., Steijaert M., Wegner J.K., Ceulemans H., Clevert D.A., Hochreiter S. (2018). Large-scale comparison of machine learning methods for drug target prediction on ChEMBL. Chem. Sci..

[10] Geronikaki A., Eleftheriou P., Poroikov V. (2016). Anti-HIV Agents: Current Status and Recent Trends. Top. Med. Chem..

[11] Guasch L., Zakharov A.V., Tarasova O.A., Poroikov V.V., Liao C., Nicklaus M.C. (2016). Novel HIV-1 integrase inhibitor development by virtual screening based on QSAR models. Cur. Top. Med. Chem..

[12] Halder A.K. (2018). Finding the structural requirements of diverse HIV-1 protease inhibitors using multiple QSAR modelling for lead identification. SAR QSAR Environ. Res..

[13] Hdoufane I., Bjij I., Soliman M., Tadjer A., Villemin D., Bogdanov J., Cherqaoui D. (2018). In silico SAR studies of HIV-1 inhibitors. Pharmaceuticals (Basel).

[14] Toropova A.P., Toropov A.A., Veselinović J.B., Miljković F.N., Veselinović A.M. (2014). QSAR models for HEPT derivates as NNRTI inhibitors based on Monte Carlo method. Eur. J. Med. Chem..

[15] Synthetically Accessible Virtual Inventory. https://cactus.nci.nih.gov/download/savi_download/.

[16] REAL Compounds Library. https://www.enaminestore.com/products/real-compounds.

[17] NIAID HIV/OI/TB Therapeutics Database. https://chemdb.niaid.nih.gov/.

[18] European Bioinformatics Institute ChEMBL Database. https://www.ebi.ac.uk/chembl/.

[19] Clarivate Analytics Integrity Database. https://integrity.clarivate.com/.

[20] Fourches D., Muratov E., Tropsha A. (2016). Trust, but verify II: A practical guide to chemogenomics data curation. J. Chem. Inf. Model..

[21] Tarasova O.A., Urusova A.F., Filimonov D.A., Nicklaus M.C., Zakharov A.V., Poroikov V.V. (2015). QSAR Modeling using large-scale databases: case study for HIV-1 reverse transcriptase inhibitors. J. Chem. Inf. Model..

[22] Nikitina A.A., Orlov A.A., Kozlovskaya L.I., Palyulin V.A., Osolodkin D.I. (2019). Enhanced taxonomy annotation of antiviral activity data from ChEMBL. Database (Oxford).

[23] Filimonov D.A., Lagunin A.A., Gloriozova T.A., Rudik A.V., Druzhilovskiy D.S., Pogodin P.V., Poroikov V.V. (2014). Prediction of the biological activity spectra of organic compounds using the PASS online web resource. Chem. Heterocycl. Comp..

[24] Filimonov D.A., Zakharov A.V., Lagunin A.A., Poroikov V.V. (2009). QNA based “Star Track” QSAR approach. SAR QSAR Environ. Res..

[25] Pogodin P.V., Lagunin A.A., Rudik A.V., Druzhilovskiy D.S., Filimonov D.A., Poroikov V.V. (2019). AntiBac-Pred: A web portal for predicting antibacterial activity of chemical compounds. J. Chem. Inform. Model..

[26] AntiHIV-Pred. http://www.way2drug.com/hiv/.

[27] Stolbov L., Druzhilovskiy D., Rudik A., Filimonov D., Poroikov V., Nicklaus M. (2019). AntiHIV-Pred: Web-resource for in silico prediction of anti-HIV/AIDS activity. Bioinformatics.

[28] Tarasova O.A., Biziukova N.Y., Filimonov D.A., Poroikov V.V., Nicklaus M.C. (2019). Data mining approach for extraction of useful information about biologically active compounds from publications. J. Chem. Inform. Model..

[29] Tropsha A. (2010). Best Practices for QSAR Model Development, Validation, and Exploitation. Mol. Inform..

[30] Filimonov D., Poroikov V., Borodina Y., Gloriozova T. (1999). Chemical Similarity Assessment through Multilevel Neighborhoods of Atoms: Definition and Comparison with the Other Descriptors. J. Chem. Inf. Comput. Sci..

