# Supplementary material for: (Q)SAR Models of HIV-1 Protein Inhibition by Drug-Like Compounds
Source: Molecules. 2019 Dec 25;25(1):87. doi: 10.3390/molecules25010087 (PMC6983201; doi:10.3390/molecules25010087)
Supplement: Supplementary file 1 [file molecules-25-00087-s001.pdf]

SUPPLEMENTARY MATERIAL:
(Q)SAR models of HIV-1 proteins inhibition by drug-like compounds

Leonid Stolbov [1], Dmitry Druzhilovskiy [1], Dmitry Filimonov [1], Marc Nicklaus [2] and Vladimir Poroikov [1,*]

[1] Laboratory of Structure-Function Based Drug Design, Institute of Biomedical Chemistry, 10 bldg. 8, Pogodinskaya str., Moscow, 119121, Russia; vladimir.poroikov@ibmc.msk.ru
[2] Computer-Aided Drug Design Group, Chemical Biology Laboratory, Center for Cancer Research, National Cancer Institute, Frederick, MD 21702, USA; mn1ahelix@gmail.com
* Correspondence: vladimir.poroikov@ibmc.msk.ru

1. **Data Selection and Curation** 2
2. **Selected Descriptors** 5
3. **QSAR Modeling Using GUSAR Program** 10
4. **PASS (Prediction of Activity Spectra of Substances) Method** 14
5. **Qualitative Characteristics of the (Q)SAR Models** 17
6. **Models based on combined data sets and their validation** 20
7. **Training Sets** 27

   8.1 Integrase training set 27

   8.2 Protease training set 175

   8.3 Reverse transcriptase training set 378

1. Data Selection and Curation

Experimental data for building models of quantitative structure-activity relationships were downloaded from three databases (DB) and then analyzed.

NIAID ChemDB HIV, Opportunistic Infection and Tuberculosis Therapeutics Database (NIAID DB) is the publicly available DB containing extracted from scientific literature on the structure and activity of compounds tested against HIV, HIV enzymes or opportunistic pathogens [https://www.niaid.nih.gov/]. It is created and supported by the National Institute of Allergic and Infectious Disease, NIH. To analyze the structure-activity relationships, we extracted information about structure and antiretroviral activity of 10377, 7604 and 8936 molecules tested as HIV-1 integrase, protease and reverse transcriptase inhibitors from the DB.

ChEMBL is the publicly available DB containing information about structure and biological activity of drug-like molecules [Davies M. et al., 2015, https://www.ebi.ac.uk/chembl/]. It is created and supported by the European Bioinformatics Institute, European Molecular Biology Laboratory (EMBL-EBI) for the past ten years. In the current study, we used ChEMBL version 24, which includes data on about 20,000 compounds, interacted with seven HIV-1 molecular targets, and more than 50,000 records with information about the associated bioactivities. To analyze the structure-activity relationships, we extracted information about structure and antiretroviral activity of 2283, 2387 and 2149 molecules tested as HIV-1 integrase, protease and reverse transcriptase inhibitors from the DB.

Integrity is commercially available DB provided by Clarivate Analytics [https://clarivate.com/products/integrity/]. To analyze the structure-activity relationships, we extracted information about structure and antiretroviral activity of 563, 316 and 731 molecules tested as HIV-1 integrase, protease and reverse transcriptase inhibitors from the DB.

Data derived from scientific literature turned out to be highly heterogeneous having different experimental conditions and various ligand efficiency indices were measured (Ki, IC50, percent of inhibition etc.). The curation process was realizied according to the pipeline (Figure 1). It corresponds to the “Best QSAR practice” (Tropsha,A., et al. Molecular Informatics, 2010, 29, 476; Fourches,D., et al. Journal of Chemical Information and Modeling, 2016, 56, 1243).

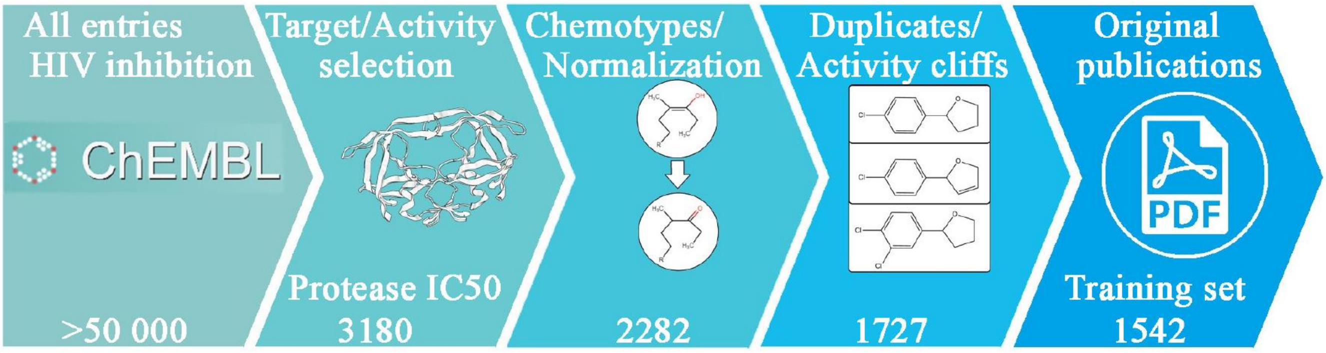

Figure 1. Data curation process pipeline.

The process could be subdivided to three parts – deletion of incorrect entries, chemical data curation and biological data curation.

Deletion of incorrect entries and standardization:

- Deletion of incomplete entries and entries that are flagged as unreliable (e.g. DATA_VALIDITY_COMMENT field in ChEMBL DB) and leaving only IC50 entries with conversion of values to molar concentrations.

Chemical data curation:

- Deletion of incomplete and insufficient entries;
- Deletion of inorganics, metallorganics, rare atom compounds;
- Mixture and salt compunds processing and standardization;
- Invalid structures recovery;
- Standardization of structures kekulization;
- Chemical groups normalization;
- Tautomeric data handling;
- Enantiomeric and stereomeric data inspection.

Biological data curation:

- Deletion of entries for mutants, drug-resistant stamps and invalid targets;
- Data split basing on experimental protocols;
- Potential duplicates were checked either for equal compunds with different activity values or for compounds having the same set of descriptors. Basing on each case the entries were deleted or the median value was calculated;
- External laboratory error estimation;
- Handling activity cliff data

In addition, the data validity was verified by analysis of the information presented in the original publications. Curated data were used to create training sets for HIV targets. Information about data sets before curation is aggregated in tables 1 and 2. Information about curated data sets is aggregated in tables 3, 4,5 and on figure 2.

Table 1. Number of molecules selected for the training sets before the cleaning procedure from three DBs.

MoA	NIAID HIV/OI/TB DB	ChEMBL DB	Integrity DB
IN inhibitors	10377	2283	563
PR inhibitors	7604	2387	316
RT inhibitors	8936	2149	731

Table 2. Intersections of data sets before the cleaning procedure from three DBs

MoA	DB pair	Intersection
	ChEMBL - Integrity	187
IN inhibitors	ChEMBL – NIAID DB	1603
	Integrity – NIAID DB	360
	ChEMBL - Integrity	57
PR inhibitors	ChEMBL – NIAID DB	1464
	Integrity – NIAID DB	232
	ChEMBL - Integrity	84
RT inhibitors	ChEMBL – NIAID DB	1380
	Integrity – NIAID DB	494

Table 3. Overlaps of DBs pairs after cleaning procedure – Unions in upper triangle, Intersections in lower triangle.

DB	MoA	NIAID HIV/OI/TB DB	ChEMBL DB	Integrity DB
NIAID HIV/OI/TB DB	IN inhibitors	3459	*3987*	*3597*
	PR inhibitors	5972	*6462*	*6068*
	RT inhibitors	5675	*6093*	*5894*
	Total	14954	*16388*	*15393*
ChEMBL DB	IN inhibitors	**902**	1430	*1628*
	PR inhibitors	**949**	1437	*1665*
	RT inhibitors	**972**	1390	*1940*
	Total	**2823**	4257	*5230*
Integrity DB	IN inhibitors	**190**	**130**	328
	PR inhibitors	**74**	**40**	268
	RT inhibitors	**396**	**65**	615
	Total	**772**	**238**	1211

Table 4. Overlaps of DBs pairs after cleaning procedure – Unions and Medians.

MoA	Union	Median
IN inhibitors	4091	1126
PR inhibitors	6554	1125
RT inhibitors	6309	1371
Total	16778	3634

Table 5. Data sets differences.

DB	MoA	Difference
NIAID HIV/OI/TB DB	IN inhibitors	2463
	PR inhibitors	4889
	RT inhibitors	4369
ChEMBL DB	IN inhibitors	494
	PR inhibitors	486
	RT inhibitors	415
Integrity DB	IN inhibitors	104
	PR inhibitors	92
	RT inhibitors	216

Figure 2. Total number of molecules extracted from three DBs and intersection between different DBs after the cleaning procedure.

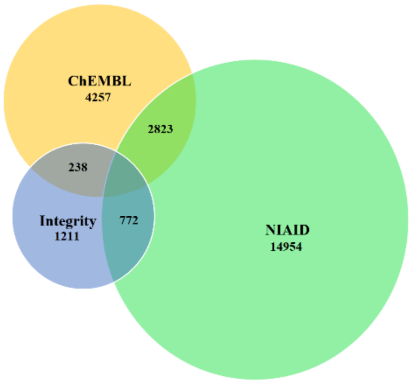

2. Selected Descriptors

The modelling was accomplished using two types of descriptors – Multilevel Neighborhoods Atoms (MNA) and Quantitative Neighborhoods of Atoms (QNA) descriptors.

MNA descriptors are based on the molecular structure representation, which includes the hydrogen atoms according to the valences and partial charges of other atoms and does not specify the types of bonds. MNA descriptors are generated as recursively defined sequence:

- zero-level MNA descriptor for each atom is the mark A of the atom itself;

any next-level MNA descriptor for the atom is the sub-structure notation $A(D_1, D_2, \ldots, D_i, \ldots)$, where D_i is the previous-level MNA descriptor for i–th immediate neighbor's of the atom A.

The mark of atom may include not only the atomic type but also any additional information about the atom. In particular, if the atom is not included into the ring, it is marked by «-». The neighbor descriptors $D_1, D_2, \ldots, D_i, \ldots$ are arranged in unique manner, e.g., in lexicographic order. Iterative process of MNA descriptors generation can be continued covering first, second, etc. neighborhoods of each atom.

Descriptors for each molecule are generated on the basis of connectivity matrix $\mathbf{C}$ and table of atoms types A. Connectivity matrix contains data on the covalent bonds in a molecule. Various bond types are not specified (topological approximation). All hydrogens based on valences and partial charges of atoms are taken into account. The types of atoms are specified according to the data presented in Table 6.

Table 6. Classification of different atom types used in calculation of descriptors

Class name	Elements
H	H
C	C
N	N
O	O
F	F
Si	Si
P	P
S	S
Cl	Cl
Ca	Ca
As	As
Se	Se

Br	Br
Li*	Li, Na
B*	B, Re
Mg*	Mg, Mn
Sn*	Sn, Pb
Te*	Te, Po
I*	I, At
Os*	Os, Ir
Sc*	Sc, Ti, Zr
Fe*	Fe, Hf, Ta
Co*	Co, Sb, W
Sr*	Sr, Ba, Ra
Pd*	Pd, Pt, Au
Be*	Be, Zn, Cd, Hg
K*	K, Rb, Cs, Fr
V*	V, Cr, Nb, Mo, Tc
Ni*	Ni, Cu, Ge, Ru, Rh, Ag, Bi
In*	In, La, Ce, Pr, Nd, Pm, Sm, Eu
Al*	Al, Ga, Y, Gd, Tb, Dy, Ho, Er, Tm, Yb, Lu, Tl
R*	R, He, Ne, Ar, Kr, Xe, Rn, Ac, Th, Pa, U, Np, Pu, Am, Cm, Bk, Cf, Es, Fm, Md, No, Lr, Db, Jl

Example of structure presentation by zero-, first- and second-levels MNA descriptors for the phenol's molecule is shown in Figure 3.

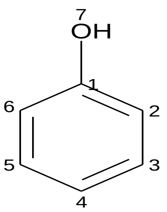

Atom	MNA/0	MNA/1	MNA/2
1	C	C(CC-O)	C(C(CC-H)C(CC-H)-O(C-H))
2	C	C(CC-H)	C(C(CC-H)C(CC-O)-H(C))
3	C	C(CC-H)	C(C(CC-H)C(CC-H)-H(C))
4	C	C(CC-H)	C(C(CC-H)C(CC-H)-H(C))
5	C	C(CC-H)	C(C(CC-H)C(CC-H)-H(C))
6	C	C(CC-H)	C(C(CC-H)C(CC-O)-H(C))
7	-O	-O(C-H)	-O(C(CC-O)-H(-O))
8	-H	-H(C)	-H(C(CC-H))
9	-H	-H(C)	-H(C(CC-H))
10	-H	-H(C)	-H(C(CC-H))
11	-H	-H(C)	-H(C(CC-H))
12	-H	-H(C)	-H(C(CC-H))
13	-H	-H(-O)	-H(-O(C-H))

MNA descriptors for phenol.
MNA/0, MNA/1,MNA/2 - zero, first and second levels of MNA descriptors.

Figure 3. MNA descriptors of 0, 1, 2 level calculated for an example structure.

QNA descriptors are calculated based on the connectivity matrix $\boldsymbol{C}$ and the standard values of ionization potential IP and electron affinity EA of atoms in a molecule. For any given atom $\boldsymbol{i}$ QNA descriptors are calculated as follow:

$$P_i = B_i \sum_k \left(\mathrm{Exp}\left(-\frac{1}{2}\boldsymbol{C}\right)\right)_{ik} B_k \qquad (\mathbf{1})$$

$$Q_i = B_i \sum_k \left(\mathrm{Exp}\left(-\frac{1}{2}\boldsymbol{C}\right)\right)_{ik} B_k A_k \qquad (\mathbf{2})$$

where

$$A_k = \frac{1}{2}(IP_k + EA_k) \qquad B_k = \frac{1}{2}(IP_k - EA_k)^{-\frac{1}{2}}$$

The values of EA and IP collected from many different sources and used in this work are represented in Table 7.

Table 7. Electron affinity (EA) and first ionization potential (IP), electron volts, and atomic radius (AR), angstroms.

Atom	EA	IP	AR	Atom	EA	IP	AR	Atom	EA	IP	AR
**H**	0.75	13.60	0.46	**Kr**	-0.42	14.00	1.98	**Lu**	0.20	6.15	1.75
**He**	0.08	24.59	1.22	**Rb**	0.49	4.18	2.48	**Hf**	0.33	7.50	1.59
**Li**	0.62	5.39	1.55	**Sr**	-0.15	5.69	2.15	**Ta**	0.40	7.89	1.46
**Be**	-0.20	9.32	1.13	**Y**	0.31	6.22	1.81	**W**	0.67	7.98	1.40

**B**	0.28	8.30	0.91	**Zr**	0.33	6.84	1.60	**Re**	0.23	7.88	1.37
**C**	1.26	11.26	0.77	**Nb**	0.51	6.88	1.45	**Os**	1.44	8.73	1.35
**N**	0.44	14.53	0.71	**Mo**	0.68	7.09	1.39	**Ir**	1.57	9.10	1.35
**O**	1.46	13.62	0.73	**Tc**	0.54	7.23	1.36	**Pt**	1.10	8.96	1.38
**F**	3.45	17.42	0.71	**Ru**	1.10	7.37	1.34	**Au**	1.25	9.23	1.44
**Ne**	0.00	21.57	1.60	**Rh**	1.14	7.46	1.34	**Hg**	-0.19	10.44	1.57
**Na**	0.55	5.14	1.87	**Pd**	1.11	8.34	1.37	**Tl**	0.31	6.11	1.71
**Mg**	-0.31	7.64	1.60	**Ag**	1.22	7.58	1.44	**Pb**	1.39	7.42	1.75
**Al**	0.30	5.99	1.43	**Cd**	-0.43	8.99	1.56	**Bi**	0.97	7.29	1.82
**Si**	1.39	8.15	1.34	**In**	0.31	5.79	1.66	**Po**	1.97	8.42	1.56
**P**	0.75	10.49	1.30	**Sn**	1.39	7.34	1.58	**At**	2.90	9.20	1.48
**S**	2.00	10.36	1.04	**Sb**	0.90	8.64	1.61	**Rn**	-0.15	10.75	2.27
**Cl**	3.61	12.97	0.99	**Te**	1.97	9.01	1.70	**Fr**	0.48	3.98	2.80
**Ar**	-0.37	15.76	1.92	**I**	3.23	10.45	1.53	**Ra**	-0.15	5.28	2.35
**K**	0.50	4.34	2.36	**Xe**	-0.25	12.13	2.18	**Ac**	0.80	5.20	2.03
**Ca**	-0.19	6.11	1.97	**Cs**	0.47	3.89	2.66	**Th**	0.80	6.10	1.80
**Sc**	0.19	6.56	1.64	**Ba**	-0.15	5.21	2.23	**Pa**	0.84	6.00	1.62
**Ti**	0.33	6.82	1.46	**La**	0.30	5.59	1.87	**U**	0.82	6.19	1.53
**V**	0.53	6.74	1.34	**Ce**	0.25	5.54	1.83	**Np**	0.82	6.20	1.50
**Cr**	0.67	6.77	1.27	**Pr**	0.20	5.47	1.83	**Pu**	0.84	6.06	1.62
**Mn**	-0.17	7.43	1.30	**Nd**	0.20	5.53	1.82	**Am**	0.85	6.00	1.70
**Fe**	0.50	7.90	1.26	**Pm**	0.20	5.58	1.81	**Cm**	0.85	6.09	1.55
**Co**	0.66	7.86	1.25	**Sm**	0.20	5.64	1.80	**Bk**	0.82	6.23	1.49
**Ni**	1.16	7.64	1.24	**Eu**	0.20	5.67	2.04	**Cf**	0.84	6.27	1.42
**Cu**	1.23	7.72	1.28	**Gd**	0.20	6.15	1.80	**Es**	0.86	6.47	1.43
**Zn**	-0.44	9.39	1.39	**Tb**	0.20	5.86	1.78	**Fm**	0.86	6.60	1.38
**Ga**	0.30	6.00	1.39	**Dy**	0.20	5.94	1.77	**Md**	0.83	6.68	1.38
**Ge**	1.39	7.90	1.39	**Ho**	0.20	6.02	1.78	**No**	0.79	6.58	1.47
**As**	0.80	9.79	1.48	**Er**	0.20	6.11	1.76	**Lr**	0.85	6.69	1.30
**Se**	2.02	9.75	1.17	**Tm**	0.20	6.18	1.75	**Db**	0.46	6.43	1.14
**Br**	3.45	11.81	1.14	**Yb**	0.20	6.25	1.94	**Jl**	0.50	6.78	1.01

Though the value $\mu(P-Q)$ can be considered by convention as the partial atomic charge, where μ is the chemical potential, in general, the P and Q values are not the estimate of partial atomic charges, hardness or so forth.

QNA describes each of the atoms in a molecule, and, at the same time, each of the P and Q values depends on the whole composition and structure of a molecule (Figure 4).

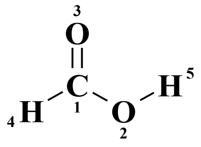

a)

$$\mathbf{C} = \begin{vmatrix} 0 & 1 & 1 & 1 & 0 \\ 1 & 0 & 0 & 0 & 1 \\ 1 & 0 & 0 & 0 & 0 \\ 1 & 0 & 0 & 0 & 0 \\ 0 & 1 & 0 & 0 & 0 \end{vmatrix}$$

b)

$$\mathrm{Exp}\left(-\tfrac{1}{2}\mathbf{C}\right) = \begin{vmatrix} 1.40 & -0.59 & -0.57 & -0.57 & 0.14 \\ -0.59 & 1.27 & 0.14 & 0.14 & -0.54 \\ -0.57 & 0.14 & 1.13 & 0.13 & -0.02 \\ -0.57 & 0.14 & 0.13 & 1.13 & -0.02 \\ 0.14 & -0.54 & -0.02 & -0.02 & 1.13 \end{vmatrix}$$

c)

	**EA**	**IP**	*A*	*B*	**P**	**Q**
**C**	1.263	11.26	6.262	0.316	-0.00218	-0.1820

**O**	1.461	13.62	7.541	0.287	0.02944	0.3019
**O**	1.461	13.62	7.541	0.287	0.06199	0.5297
**H**	0.754	13.60	7.177	0.279	0.05812	0.4706
**H**	0.754	13.60	7.177	0.279	0.05304	0.3533

d)

Figure 4. Example of QNA description of *formic acid*: (a) structure diagram; (b) connectivity matrix; (c) exponent of the connectivity matrix; (d) electron affinities, ionization potentials, variables of equations (1) and (2), P and Q values for each atom of *formic acid* molecule.

From Figure 3c it is clear that any atom influences the others, though the influence decreases with the increase of the distance between them; *e.g.* components of matrix $\mathrm{Exp}\left(-\frac{1}{2}\boldsymbol{C}\right)$ for atom 1 (C) are: 1.40 for atom 1 itself, -0.59 for its immediate neighbor atom 2 (O), -0.57 for atoms 3 (O) and 4 (H), and 0.14 for atom 5 (H).

The algorithm of QNA descriptor calculation is quite simple due to uselessness of the matrix $\mathrm{Exp}\left(-\frac{1}{2}\boldsymbol{C}\right)$ itself, only product of $\mathrm{Exp}\left(-\frac{1}{2}\boldsymbol{C}\right)$ by vector is necessary, and that matrix $\boldsymbol{C}$ consists of 0 and 1 only.

3. QSAR Modeling Using GUSAR Program

QNA describe each particular atom of a molecule, at the same time, each P or Q value depends on the total molecule composition and structure. To use this molecule structure representation we have proposed to calculate each $f_i(S)$ function of the structure of a molecule as the average value of $g_i(P,Q)$ function of P and Q variables for those m atoms in a molecule, which have two or more immediate neighbors:

$$f_i(S) = \frac{1}{m}\sum_k g_i(P,Q) \qquad (3)$$

$$y_{pred} = a_0 + \sum_i a_i \frac{1}{m}\sum_k g_i(P,Q) \quad (4)$$

According to the equation (4) the estimate y_{pred} for molecule can be interpreted as an average of values predicted for particular atoms in a molecule. Formally, QNA descriptors represent a molecule structure by two descriptors only (P and Q), in contrast to numerous traditional descriptors used in QSAR. Since P and Q values have different scales (standard deviations are 0.023 and 0.208, respectively), we made the normalization to optimize a family of functions $g_i(P,Q)$.

Normalization has been performed by calculation of the average values (E_P and E_Q), standard deviations (D_P and D_Q) and correlation between P and Q values (R_{PQ}):

$$P' = \frac{P - E_P}{D_P} \qquad Q' = \frac{Q - E_Q}{D_Q} \qquad (5)$$

$$U = \frac{P' + Q'}{\sqrt{2(1 + R_{PQ})}} \qquad V = \frac{P' - Q'}{\sqrt{2(1 - R_{PQ})}} \qquad (6)$$

The orthonormal U and V have zero mean, unit variance and they are uncorrelated.

Chebyshev polynomials were chosen as the family of functions$g_i(P,Q)$, and the orthonormal U and V values have been additionally transformed by using hyperbolic tangent, so, the "normalized QNA" vary from -1 to 1. After this, the functions $g_i(P,Q)$ in equation (4) are represented using Chebyshev polynomials as:

$$g_i(P,Q) = T_{uv}(P,Q) = \mathrm{Cos}\left(u * \mathrm{ArcCos}\big(\mathrm{TanH}(U)\big)\right) * \mathrm{Cos}\left(v * \mathrm{ArcCos}\big(\mathrm{TanH}(V)\big)\right) \quad (7)$$

where the integer numbers $u, v = 0,1,2$ ... define 2-dimesional Chebyshev polynomial degree. The final equation for estimate y_{pred} using QNA descriptors is:

$$y_{pred} = \frac{1}{m}\sum_k \left(a_0 + \sum_{uv} a_{uv} T_{uv}(P,Q)\right) \qquad (8)$$

QNA descriptors and their polynomial transformations (5)-(7) do not provide information on the shape and volume of a molecule although this information may be important for determination of the structure-activity relationships. Therefore, these parameters were added to QNA descriptors. Topological length of a molecule was calculated as the maximal distance between any two atoms and

the volume of a molecule – as the sum of each atom's volume, $\frac{4}{3}\pi R^3$, where R is the atomic radius (see Table 2 (AR)).

The Chebyshev polynomials are arranged in ascending order of their degrees $u + v$. For $u + v = 1$ they are $T_{1,0}$, $T_{0,1}$; for $u + v = 2$ they are $T_{2,0}$, $T_{1,1}$, $T_{0,2}$ etc. The first, second and third power of topological length and volume of a molecule were used. The number of initial variables equals to the number of Chebyshev polynomials plus the number of the first, second and third power of topological length and volume of a molecule.

The number of initial variables depends on the number of compounds in the training set. If the number of compounds in the training set less than 25, then the initial variables are 24. If the number of compounds in the training set varied from 25 to 100, then the initial variables depends on the following equation:

$$A = (Ln(B) \times 18.755) - 36.37,$$

where A – initial variables and B – the number of compounds in the training set. If the number of compounds in the training set varied from 100 to 2000, then the number of initial variables equal to one-half of the number of compounds in the training set. If the number of compounds in the training set exceeds 2000, then the initial variables are 1000.

GUSAR algorithm uses three procedures described below for generation of different QSAR models based on QNA descriptors.

1) U and V values from (7) are multiplied by value, which are randomly chosen from 0.98 to 1.09.

2) Coefficient before matrix **C** is randomly chosen from -0.1 to -0.9.

3) Calculation of QNA descriptors is randomly chosen from two cases. First case is when P and Q values are calculated for all atom types. Second case is when P and Q values are calculated for each atom, which has connection with more than one carbon.

All these procedures are used for creation of each QSAR model based on QNA descriptors. These procedures allow obtaining the different QNA models.

Self-Consistent Regression (SCR)

The classical MLR has a number of limitations. Particularly, the number of objects in the training set should significantly exceed the number of independent variables, and it is important to use the non-collinear variables only. To overcome these limitations, we have employed the approach based on statistical regularization of ill-posed problems. It has resulted in the regularized least-squares method:

$$\boldsymbol{a} = \text{ArgMin}\left[\sum_{i=1}^{n}\left(y_i - \sum_{k=0}^{m}(x_{ik}a_k)\right)^2 + \sum_{k=1}^{m}(v_k {a_k}^2)\right] \quad \textbf{(9)}$$

where $\boldsymbol{a}$ is the regression coefficient, n is the number of objects, y_i is the response value of ith object, m is, here and below, the number of the independent variables, x_{ik} is the value of kth independent

variable of ith object, a_k is the kth value of the regression coefficients, v_k is the kth value of the regularization parameters. Equation (9) has the following solution:

$$\boldsymbol{a} = \mathbf{T}\mathbf{X}^T\boldsymbol{y}, \quad \mathbf{T} = (\mathbf{X}^T\mathbf{X} + \mathbf{V})^{-1}$$

where $\mathbf{X}^T$ is the transposed regression matrix $\mathbf{X}$, $\mathbf{V}$ is a diagonal matrix of the regularization parameters. The best regularization $\mathbf{V}$ satisfies to the equations:

$$v_k({a_k}^2 + s^2 t_k) = s^2, \ \ k = 1, \dots, m$$

where s is the standard deviation of residuals, t_k is the kth diagonal element of matrix $\mathbf{T}$.

Use of Nearest Neighbours

Algorithm of GUSAR allows using nearest neighbours for improvement of prediction value obtained from regression model. The corrected value is estimated by taking an average of the three chemicals values in the training set that are the most similar to the chemical under study. Similarity of the any pair of chemical compounds is estimated as Pirson's coefficient calculated in the space of independent variables obtained after SCR. This function usually increases the prediction accuracy for the external test sets for QSAR models created on the basis of training sets with 500 and more structures. Sometimes the use of this function may lead to decrease of accuracy prediction, which depends on the quality of the training sets.

Consensus method

The predicted value is estimated by taking into account a weighted average of the predicted values from the obtained QSAR models (QSAR models provide the predictions that are within the respective applicability domains). Prediction obtained from each developed model is weighted on its applicability domain values (AD). GUSAR algorithm calculates the similarity value; leverage value and estimation of three nearest neighbours' accuracy of prediction for the applicability domain assessment (see AD description, page 70). Weighting of the predicted value is performed using similarity and leverage values only.

If all predicted values from the obtained QSAR models exceed the similarity threshold and leverage threshold, then the consensus prediction value is calculated from similarity weighted predicted values. If all predicted values from the obtained QSAR models are less than the leverage and similarity thresholds, then the consensus prediction value is calculated from leverage weighted predicted values. If the predicted values from the obtained QSAR models exceed similarity and is less than leverage thresholds, then the consensus prediction value is calculated as average from leverage weighted predicted values and similarity weighted predicted values. If the predicted values from the obtained QSAR models are less than similarity and exceed the leverage thresholds, then the consensus prediction value is calculated as average from all weighted predicted values.

Similarity

Three nearest neighbours from the training set are calculated for each chemical compound under study using similarity estimation. Similarity of the pair of chemical compounds is estimated as Pirson's coefficient calculated in the space of independent variables obtained after SCR. The average similarity of three nearest neighbours is used for assessment of the applicability domain (AD) of the model. If the average similarity exceeds the threshold, then

the studied chemical compound falls in AD of the model and vice-versa. The higher value of the threshold was selected, the more similar compounds fell in AD of the model.

Leverage

Hat value of leverage was used for domain applicability assessment. Hat values from the leverage matrix representing the "distance" of the molecule to the model structural space were calculated as:

$$Leverage = x^T(\mathbf{X}^T\mathbf{X})^{-1}x$$

where x is a vector of descriptors of a query compound, and $\mathbf{X}$ is a matrix formed with rows corresponding to the descriptors of molecules from the training set. Hat value of leverage was calculated for each compound of the training set and then a distribution of the obtained values was estimated.

A warning level of hat value was considered as percentile. The end-user could select 99th, 95th and 90th percentile. Therefore, if a chemical compound from the external test set has hat value exceeded this warning level, then this compound is considered as being out of the applicability domain.

Accuracy of three nearest neighbours' predictions

For the assessment of this type of the applicability domain the following equation is used:

$$AD_{value} = \frac{RMSE_{3NN}}{RMSE_{train}}$$

where AD_{value} is the applicability domain value, $RMSE_{3NN}$ is root mean square error of prediction of three most similar compounds from the training set, $RMSE_{train}$ is root mean square error of predictions for the training set.

Three nearest neighbours from the training set are calculated for each studied chemical compound using similarity value. Similarity of the two chemical compounds is estimated as Pirson's coefficient calculated in the space of independent variables obtained by SCR.

If the AD_{value} is less than the threshold , then the studied chemical compound falls into AD of the model and vice-versa. Therefore, any chemical compound, whose three nearest neighbours are predicted worse than the prediction accuracy of the whole training set, is considered as being out of the applicability domain.

Leave-Many-Out procedure

This procedure is used for assessment of external prediction accuracy. The initial data is randomly divided onto the training and the external test sets. End- user could select the following proportions:

10% for the external test set and 90% for the training set;

20% for the external test set and 80% for the training set;

30% for the external test set and 70% for the training set;

50% for the external test set and 50% for the training set.

For obtaining the objective assessment of predictive accuracy and robustness of the developed models each splitting should be repeated many times. End-user could select the following number of repeats: 1, 5, 10 and 20.

Y-randomization procedure

This procedure allows to be ensuring that the developed models do not have the overfitting. In this procedure the dependent-variable vector, Y vector, is randomly shuffled and new QSAR model is developed using the original independent-variable matrix. It is expected that the resulting models should generally have low Q^2 values. This procedure could be repeated many times for each model, and then the average Q^2 value is calculated.

4. PASS (Prediction of Activity Spectra of Substances) Method

In PASS biological activities are described qualitatively (active or inactive). Any property of chemical compounds, which is determined by their structural peculiarities, can be used for prediction by PASS. It is clear, that the applicability of PASS is broader than the prediction of biological activity spectra.

The MNA descriptors (both for predicting the activity spectrum of the compound and for adding substances to SAR Base) are generated only if molecular structure corresponds to the following criteria:

- each of the atoms in a molecule must be presented by atom symbol from the periodic table. Symbols of unspecified atom A, Q, *, or R group labels are not allowed;
- each of the bonds in a molecule must be covalent bond presented by single, double or triple bond types only;
- molecular structure must include three or more carbon atoms;
- molecular structure must include only one component. Single atom parts like HCl, Cl-, OH-, Na+, etc., (hydrogen atoms do not take into account) are excluded from MNA descriptors generation;
- structure (main part, see the previous sentence) must be uncharged;
- the absolute molecular weight of a substance must be less than 1250 Da.

A relevant error will be generated whenever a molecular structure does not correspond to these criteria or input data contains some other errors.

The PASS training sets for HIV-associated comorbidites include about 240,000 thoroughly selected data records about structure and biological activity of organic compounds from the general PASS training set. The knowledgebase SAR Base is created in process of training on the basis of the PASS training set.

The PASS estimations of biological activity spectra of new compounds are based on the Structure-Activity Relationships knowledge-base (SAR Base), which accumulates the results of the training set analysis. SAR Base includes a biological activities dictionary and MNA descriptors dictionary, data and knowledge on the "structure – biological activity" relationships, the DB of structure of compounds from the training set with their biological activity spectra. The structure of the compound represented as a set of MNA descriptors. Unfortunately, it is currently impossible to make a large collection of

biologically active compounds using only publicly available sources, for which assay results for all types of biological activity were known.

Algorithm of activity spectrum prediction description may be done easy using well known Bayesian approach. For the chemical compound C, which molecular structure is represented by the set $\{D_1,\ldots,D_m\}$ of m MNA descriptors, estimate the probability $P(A|C)$ that the compound C has an activity A. According to the Bayes formula:

$$P(A|C) = \frac{P(A)P(C|A)}{P(C)} \quad (\mathbf{9})$$

where $P(A)$ is the activity A *prior* probability; $P(C|A)$ is the conditional probability of compound C providing that it has activity A; $P(C)$ is the compound C prior probability.

On the assumption that descriptors $D_1,\ldots,D_m$ are independent one can write the probability $P(C|A)$ as the product of conditional probabilities for particular descriptors:

$$P(C|A) \cong P(D_1,\ldots,D_m|A) = \prod_{i=1}^{m} P(D_i|A)$$

This expression is approximately true since the MNA descriptors are a fortiori dependent due to their generation method. However, we have not acceptable alternatives, and we cannot forget about the approximation of obtained formulas.

After simple transformations, we obtain the expression for the log-likelihood ratio of the conditional probabilities $P(A|C)$ for activity A and $P(B|C)$ for activity B as:

$$ln\left[\frac{P(A|C)}{P(B|C)}\right] \cong ln\left[\frac{P(A)}{P(B)}\right] + \sum_{i=1}^{m}\left\{ln\left[\frac{P(A|D_i)}{P(B|D_i)}\right] - ln\left[\frac{P(A)}{P(B)}\right]\right\}$$

In a particular case, where B is the lack of activity A, we obtain:

$$ln\left[\frac{P(A|C)}{1-P(A|C)}\right] \cong ln\left[\frac{P(A)}{1-P(A)}\right] + \sum_{i=1}^{m}\left\{ln\left[\frac{P(A|D_i)}{1-P(A|D_i)}\right] - ln\left[\frac{P(A)}{1-P(A)}\right]\right\}$$

The implication of the expression is quite clear: the logarithm of the posterior likelihood ratio is the sum of the logarithm of the a priori likelihood ratio and the sum of individual descriptors contributions. And, if the activity is not dependent on this descriptor, then $P(A|D_i) = P(A)$ and the such descriptor has no affect on the results and its contribution to the sum is zero. This is the classical result of the probabilistic approach. But, apart from the already marked proximity, this result has another significant, well-known disadvantage: the contribution of some descriptors is too large and suppresses all other terms of the sum for which the conditional probability of activity is too close to 0 or 1, when its present in the structure. The most pronounced effect could be in the situation, when for probabilities $P(A|D_i)$ used frequency estimates based on analysis of the training set and the values 0 and 1 are the rule rather than the exception.

To overcome this problem one can offer many different approaches and they were tested in the PASS development. The best result was obtained by using of so-called Fischer $ArcSin(2p-1)$ conversion instead of $ln[p/(1-p)]$: its shape coincides with the shape of $ln[p/(1-p)]$ for almost all values of p, but $ArcSin(2-1)$ values are bounded by the values ±π/2. The accuracy of prediction also improved after changing the sum of descriptor contributions by the their average value, that apparently

compensates for the assumption of descriptors independence. Logarithm of the a priori likelihood ratio has little information about a specific predicted organic compound and can be omitted.

Bayesian approach described above explains why PASS prediction algorithm based on the following specific statistics: on the basis of a molecular structure represented by the set $\{D_1,\ldots,D_m\}$ of m MNA descriptors, the B_k values are calculated for each activity A_k:

$$B_k = \frac{S_k - S_{0k}}{1 - S_k \cdot S_{0k}}$$

$$S_k = Sin\left[\sum_i ArcSin(2P(A_k|D_i) - 1)/m\right] \quad S_{0k} = 2P(A_k) - 1$$

For each kind of activity, if for all descriptors of molecule $P(A_k|D_i) = 1$, then $B_k = 1$; if for all descriptors of molecule $P(A_k|D_i) = 0$, then $B_k = -1$; if the relationship between descriptors of molecule and activity A_k does not exist and $P(A_k|D_i) \approx P(A_k)$, then $B_k \approx 0$.

The PASS prediction algorithm uses the following data on the "structure-activity" relationships:

N is the total number of compounds in the SAR Base;

N_i is the number of compounds contained descriptor D_i in the structure description;

N_k is the number of compounds contained the activity A_k in the activity spectrum;

N_{ik} is the number of compounds contained both the activity A_k and the descriptor A_k.

The simplest frequency estimations of probabilities $P(A_k)$ и $P(A_k|D_i)$ are given by:

$$P(A_k) = \frac{N_k}{N}, \quad P(A_k|D_i) = \frac{N_{ik}}{N_i}$$

Estimation of PASS prediction accuracy and dependency required for calculation probabilities Pa and Pi on the basis of B statistics, are the end result of the training procedure, which consists in the following. According to the SAR Base, formed on the basis of the training set, for each kind of activity A_k, for each N_k active, and for each $N - N_k$ inactive compound, B statistics values are calculated. Calculations are carried out in the Leave-One-Out Cross-Validation (LOO CV), i.e., after the "exclusion" of the compound from SAR Base, for what is enough to not include it in sum. Smooth estimations of the distribution functions $Pa(B)$ and $Pi(B)$ are based on the obtained sets of B statistics.

The probabilities Pa and Pi are both the measures of belonging to subsets of "active" and "inactive" compounds, and the probabilities of the 1st and 2nd kinds of prediction error, respectively. These two interpretations of the probabilities Pa and Pi are equivalent and can be used for understanding the results of prediction. It allows constructing different critera for analyzing the results of prediction corresponding to the solution of specific practical problems.

An important feature of the PASS prediction algorithm is its robustness to the imperfection of information on the structure and biological activity spectrum of organic compounds in the training set. In a special study was showed that halving the actual well-known information about the structure or activity of organic compounds in the training set only slightly reduces the accuracy of prediction in cross-validation. There's demonstrated that the accuracy base on a LOO CV is even more rigid than for cross-validation.

5. Qualitative Characterstics of the (Q)SAR Models

Here we summarize the information on developed quantitative models for HIV-1 protease, reverse transcriptase, integrase.

Determination coefficient (R^2), Cross-validated determination coefficient (Q^2), Fisher coefficient (F) and root-mean-square-errors (RMSE) for quantitative models based on training sets before cleaning procedure are shown in Tables 8, 9, 10. Observed versus predicted plots for these models on figures 5, 6, 7.

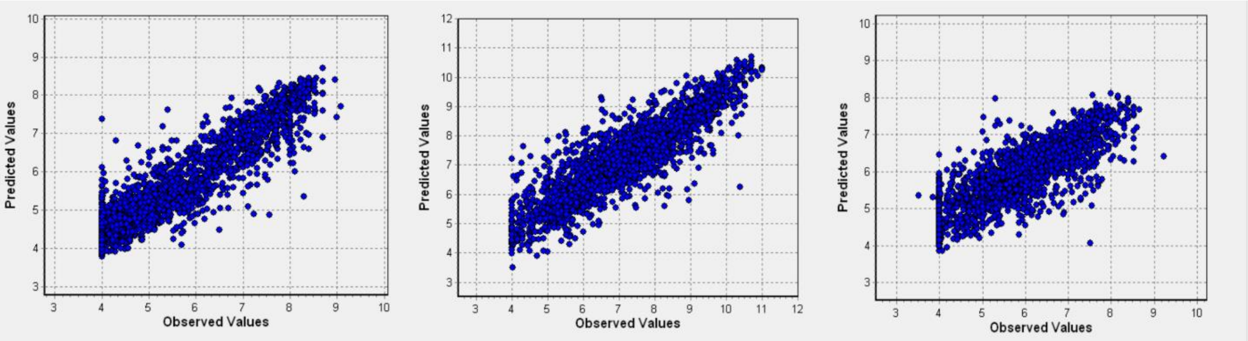

Figure 5. Observed versus predicted plots for ChEMBL training set based QSAR models.

Table 8. ChEMBL training set based QSAR models.

	HIV-IN pIC50	HIV-PR pIC50	HIV-RT pIC50
R^2	0.970	0.933	0.920
Q^2	0.856	0.800	0.655
F	25.368	22.483	10.045
RMSE	0.535	0.724	0.650
V	375	325	288
N	2283	2387	2149

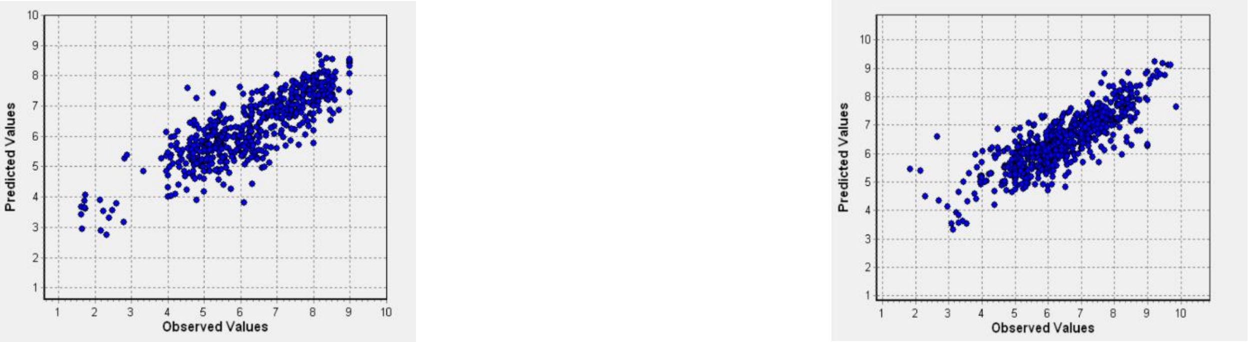

Figure 6. Observed versus predicted plots for Integrity training set based QSAR models.

Table 9. Integrity training set based QSAR models.

	HIV-IN pIC50	HIV-PR pIC50	HIV-RT pIC50
R^2	0.772		0.951
Q^2	0.694		0.694
F	7.204	Models were not created	7.761
RMSE	0.849		0.740
V	104		126
N	563		731

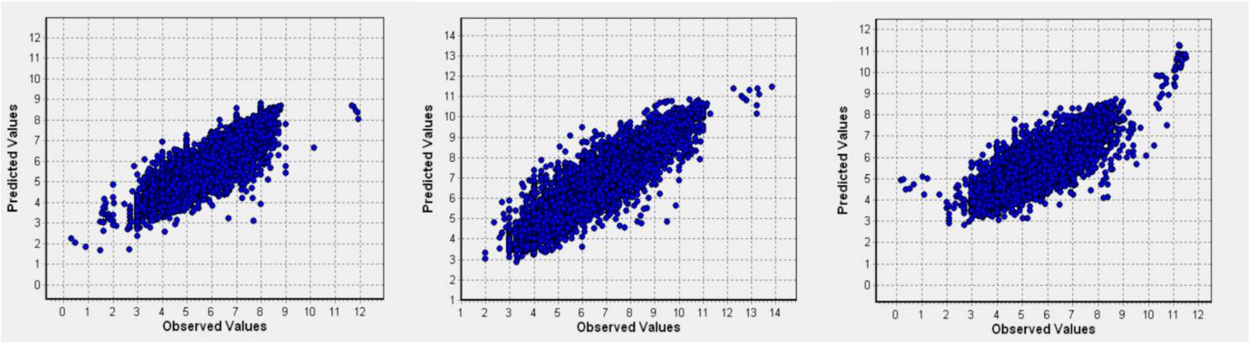

Figure 7. Observed versus predicted plots for NIAID training set based QSAR models.

Table 10. NIAID training set based QSAR models.

	HIV-IN pIC50	HIV-PR pIC50	HIV-RT pIC50
R^2	0.887	0.875	0.810
Q^2	0.803	0.789	0.658
F	82.762	62.263	38.127
RMSE	0.725	0.870	0.833
V	548	543	543
N	10377	7604	8936

Determination coefficient (R^2), Cross-validated determination coefficient (Q^2), Fisher coefficient (F) and root-mean-square-errors (RMSE) for quantitative models based on training sets after cleaning procedure are shown in Tables 11, 12, 13. Observed versus predicted plots for these models on figures 8, 9, 10.

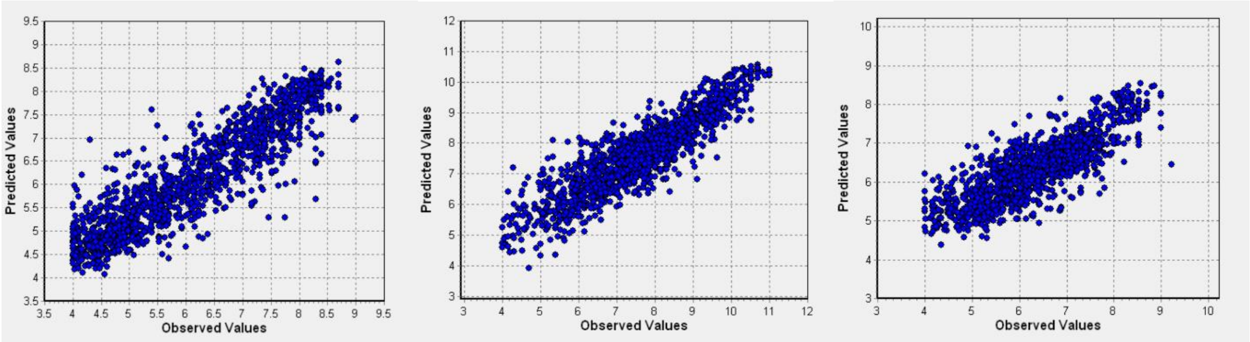

Figure 8. Observed versus predicted plots for ChEMBL training set based QSAR models after curation.

Table 11. ChEMBL training set based QSAR models after curation.

	HIV-IN pIC50	HIV-PR pIC50	HIV-RT pIC50
R^2	0.968	0.966	0.944
Q^2	0.829	0.809	0.675
F	19.769	17.814	10.890
RMSE	0.564	0.642	0.601
V	246	239	188
N	1430	1437	1390

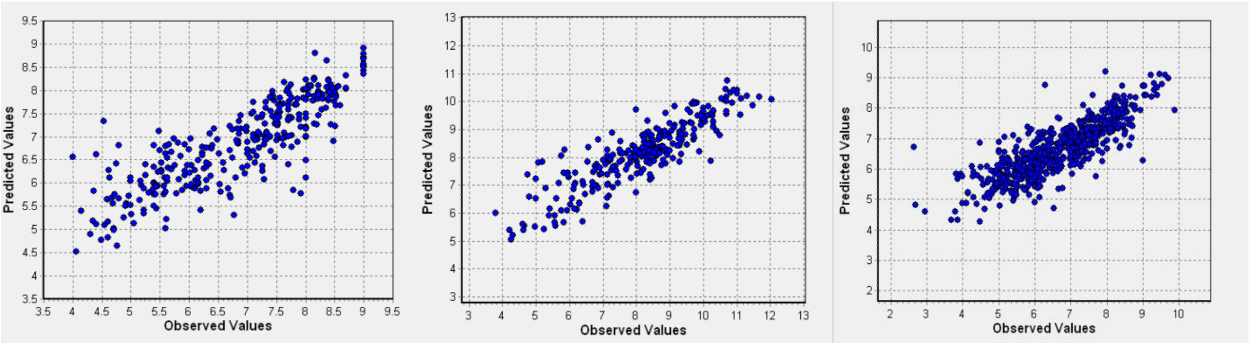

Figure 9. Observed versus predicted plots for Integrity training set based QSAR models after curation.

Table 12. Integrity training set based QSAR models after curation.

	HIV-IN pIC50	HIV-PR pIC50	HIV-RT pIC50
R^2	0.958	0.969	0.951
Q^2	0.723	0.768	0.688
F	8.546	7.68	7.887
RMSE	0.678	0.823	0.703
V	58	55	107
N	328	268	615

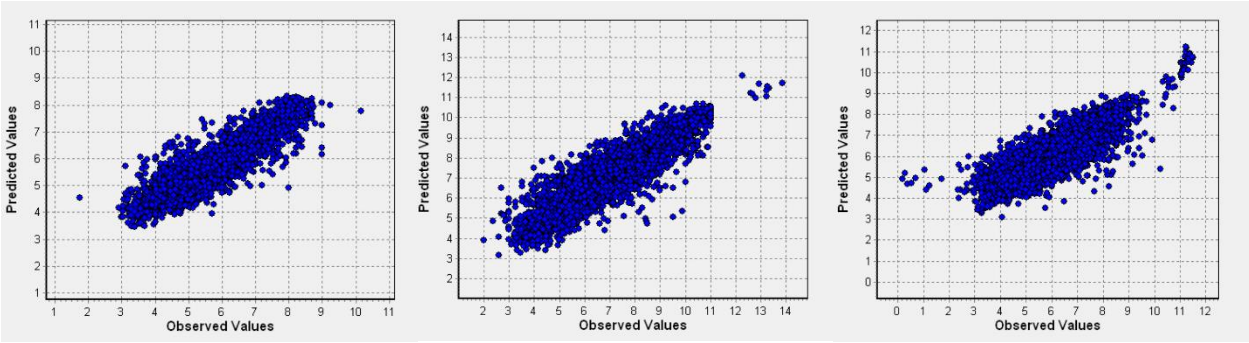

Figure 10. Observed versus predicted plots for NIAID training set based QSAR models after curation.

Table 13. NIAID training set based QSAR models after curation.

	HIV-IN pIC50	HIV-PR pIC50	HIV-RT pIC50
R^2	0.963	0.956	0.944
Q^2	0.829	0.826	0.724
F	34.181	46.746	23.534
RMSE	0.572	0.713	0.768
V	374	465	447
N	3459	5972	5675

6. Models based on combined data sets and their validation

Taking into account that NIAID set was the biggest one, for each of IN, PR and RT inhibitors we developed three models using as the training set: I – NIAID + ChEMBL; II – NIAID + Integrity; III - NIAID + ChEMBL + Integrity.

Determination coefficient (R2), Cross-validated determination coefficient (Q2), Fisher coefficient (F) and root-mean-square-errors (RMSE) for quantitative models based on integrase combined training sets are shown in Table 14. Observed versus predicted plots for these models on figure 11. IAP for classification models based on integrase combined training sets are shown in Table 15.

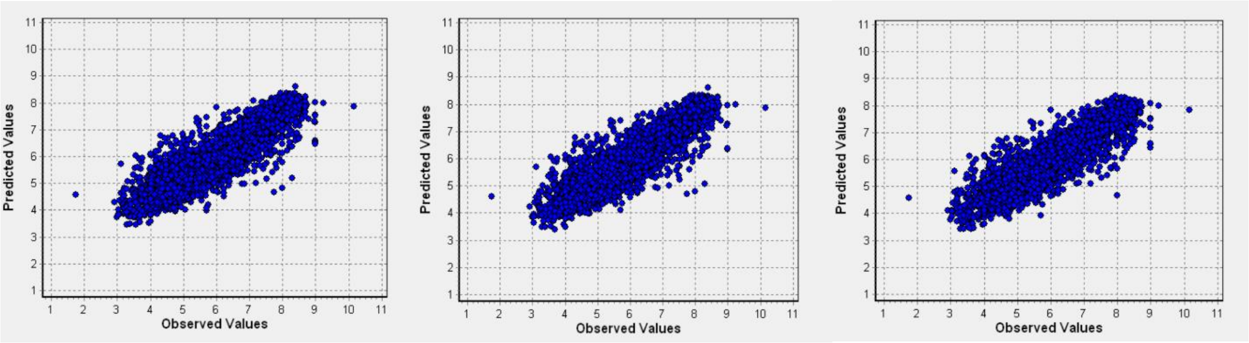

Figure 11. Observed versus predicted plots for Combined training sets based QSAR models for integrase.

Table 14. Combined training sets based QSAR models for integrase.

	ChEMBL+Integrity+NIAID	ChEMBL+NIAID	Integrity+NIAID
R^2	0.960	0.960	0.960
Q^2	0.818	0.821	0.819
F	36.457	36.998	33.695
RMSE	0.595	0.587	0.592
V	392	384	371
N	4091	3987	3597

Table 15. Combined training sets based classification models for integrase.

	ChEMBL+Integrity+NIAID	ChEMBL+NIAID	Integrity+NIAID
IAP	0.9215	0.9241	0.9220
Active	1884	1813	1622
Inactive	2139	2108	1930

Model ChEMBL+NIAID for integrase was validated on external test set obtained by excluding ChEMBL and NIAID structures from Integrity data set. Prediction results for continuous data model and classification model are presented on figure 12 and table 16.

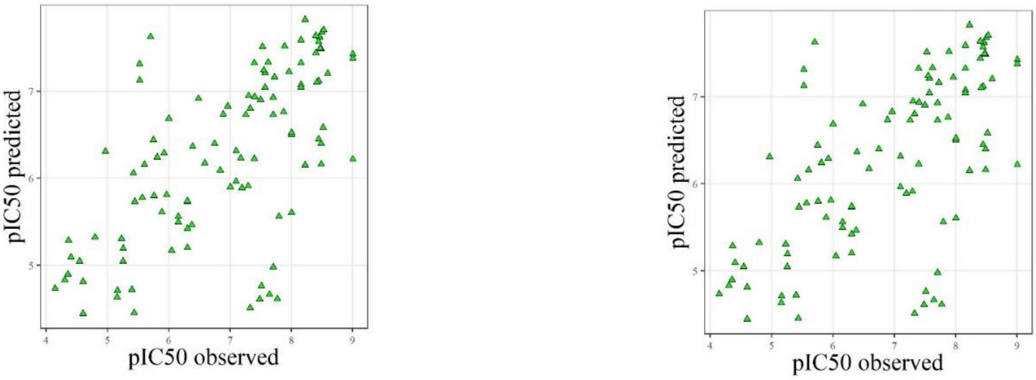

Figure 12. Observed versus predicted plots for predicting all structures ($R^2 = 0.399$) and structers in AD ($R^2 = 0.400$) for ChEMBL+NIAID integrase model.

Table 16. Prediction results for ChEMBL+NIAID integrase classification model.

Sensivity	Specifity	Balanced accuracy
0.753	0.677	0.715

Model Integrity+NIAID for integrase was validated on external test set obtained by excluding Integrity and NIAID structures from ChEMBL data set. Prediction results for continuous data model and classification model are presented on figure 13 and table 17.

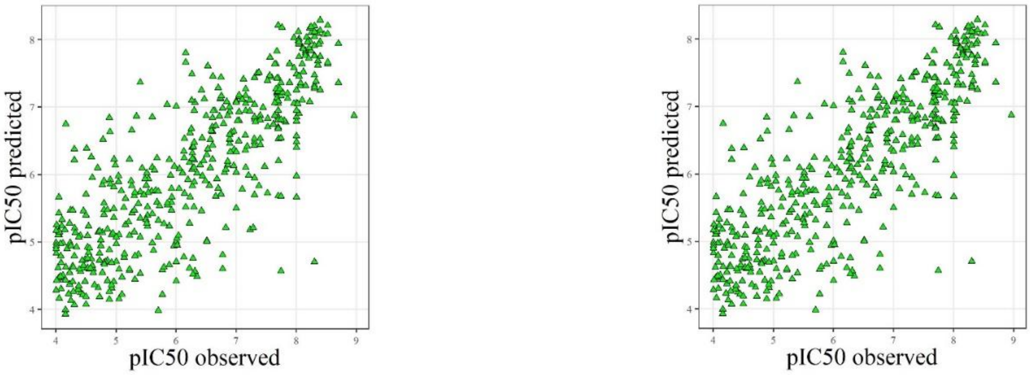

All structures prediction (R^2 = 0.667)   Structures in AD prediction (R^2 = 0.678)

Figure 13. Observed versus predicted plots for predicting all structures (R^2 = 0.667) and structers in AD (R^2 = 0.668) for Integrity+NIAID integrase model.

Table 17. Prediction results for Integrity+NIAID integrase classification model.

Sensivity	Specifity	Balanced accuracy
0.813	0.820	0.817

Determination coefficient (R2), Cross-validated determination coefficient (Q2), Fisher coefficient (F) and root-mean-square-errors (RMSE) for quantitative models based on protease combined training sets are shown in Table 18. Observed versus predicted plots for these models on figure 14. IAP for classification models based on integrase combined training sets are shown in Table 19.

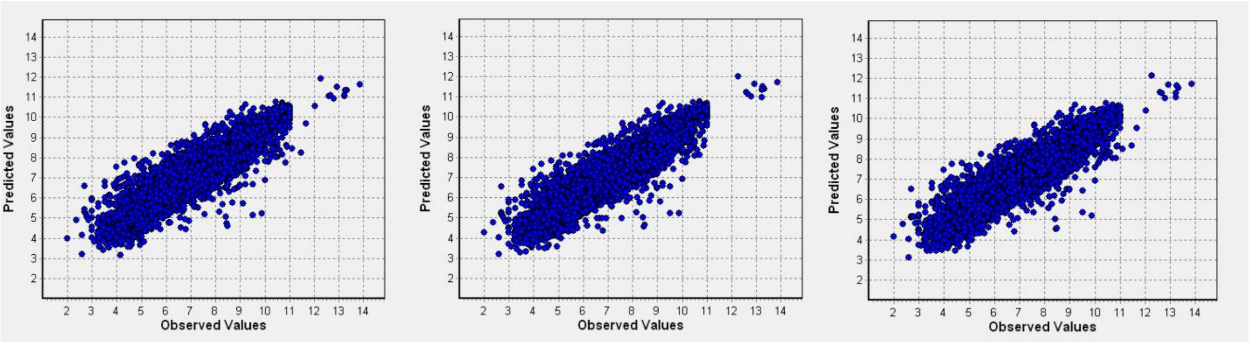

Figure 14. Observed versus predicted plots for Combined training sets based QSAR models for protease.

Table 18. Combined training sets based QSAR models for protease.

	ChEMBL+Integrity+NIAID	ChEMBL+NIAID	Integrity+NIAID
R^2	0.954	0.956	0.957
Q^2	0.824	0.829	0.827
F	50.024	49.217	46.100
RMSE	0.709	0.696	0.710
V	470	494	485
N	6554	6462	6068

Table 19. Combined training sets based classification models for protease.

	ChEMBL+Integrity+NIAID	ChEMBL+NIAID	Integrity+NIAID
IAP	0.9366	0.9376	0.9365
Active	4840	4762	4504
Inactive	1351	1337	1298

Model ChEMBL+NIAID for protease was validated on external test set obtained by excluding ChEMBL and NIAID structures from Integrity data set. Prediction results for continuous data model and classification model are presented on figure 15 and table 20.

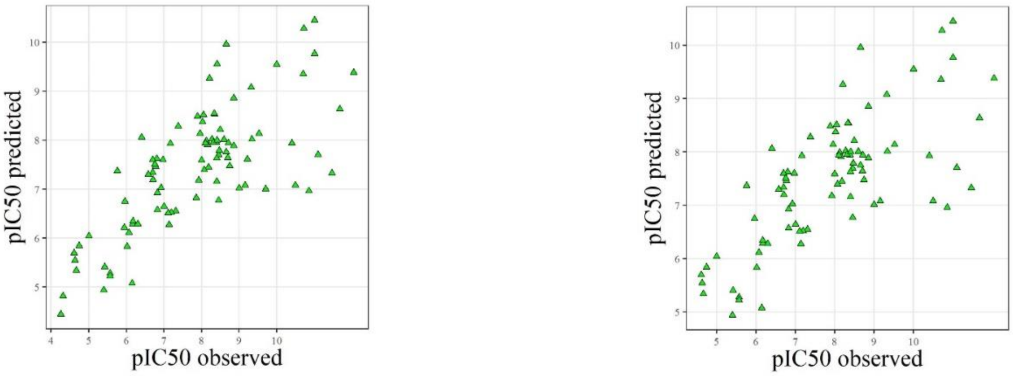

Figure 15. Observed versus predicted plots for predicting all structures ($R^2 = 0.556$) and structers in AD ($R^2 = 0.540$) for ChEMBL+NIAID protease model.

Table 20. Prediction results for ChEMBL+NIAID protease classification model.

Sensivity	Specifity	Balanced accuracy
0.697	0.857	0.777

Model Integrity+NIAID for protease was validated on external test set obtained by excluding Integrity and NIAID structures from ChEMBL data set. Prediction results for continuous data model and classification model are presented on figure 16 and table 21.

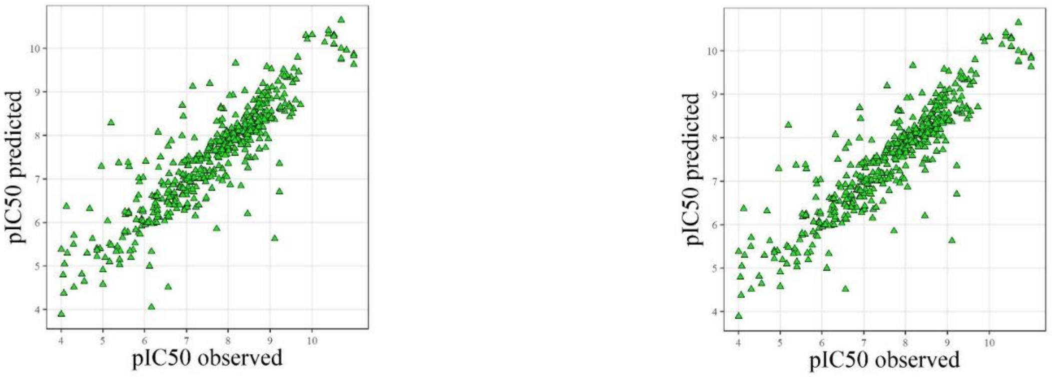

Figure 16. Observed versus predicted plots for predicting all structures ($R^2 = 0.791$) and structers in AD ($R^2 = 0.801$) for Integrity+NIAID protease model.

Table 21. Prediction results for Integrity+NIAID protease classification model.

Sensivity	Specifity	Balanced accuracy
0.826	0.788	0.807

Determination coefficient (R2), Cross-validated determination coefficient (Q2), Fisher coefficient (F) and root-mean-square-errors (RMSE) for quantitative models based on reverse transcriptase combined training sets are shown in Table 22. Observed versus predicted plots for these models on figure 17. IAP for classification models based on integrase combined training sets are shown in Table 23.

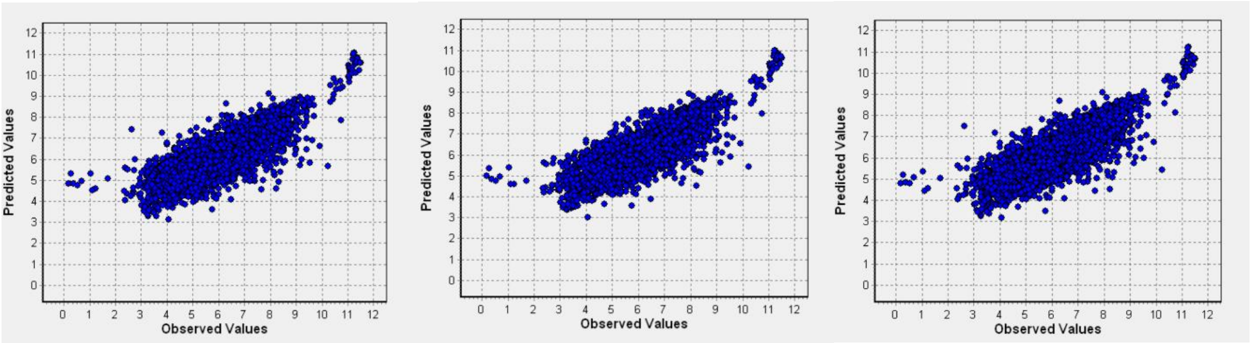

Figure 17. Observed versus predicted plots for Combined training sets based QSAR models for reverse transcriptase.

Table 22. Combined training sets based QSAR models for reverse transcriptase.

	ChEMBL+Integrity+NIAID	ChEMBL+NIAID	Integrity+NIAID
R^2	0.941	0.943	0.942
Q^2	0.714	0.723	0.715
F	24.306	23.993	23.749
RMSE	0.767	0.760	0.776
V	452	455	441
N	6309	6093	5894

Table 23. Combined training sets based classification models for reverse transcriptase.

	ChEMBL+Integrity+NIAID	ChEMBL+NIAID	Integrity+NIAID
IAP	0ı08760	0.8784	0.8780
Active	3286	3142	3054
Inactive	2935	2854	2752

Model ChEMBL+NIAID for reverse transcriptase was validated on external test set obtained by excluding ChEMBL and NIAID structures from Integrity data set. Prediction results for continuous data model and classification model are presented on figure 18 and table 24.

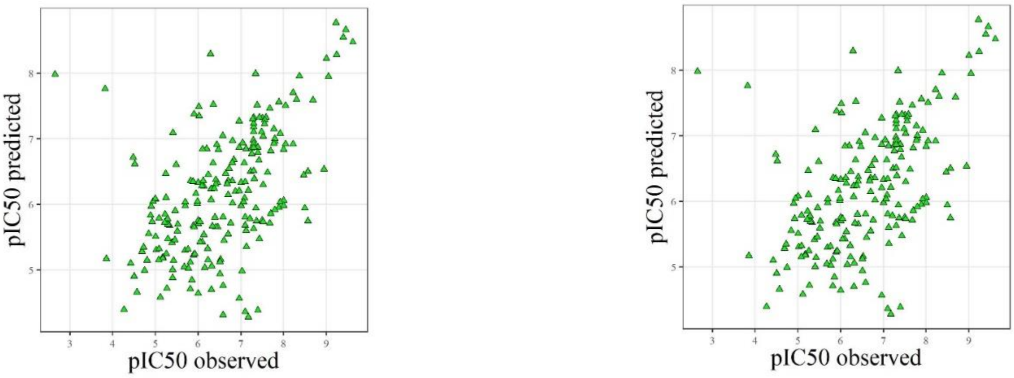

Figure 18. Observed versus predicted plots for predicting all structures ($R^2 = 0.261$) and structers in AD ($R^2 = 0.264$) for ChEMBL+NIAID reverse transcriptase model.

Table 24. Prediction results for ChEMBL+NIAID protease classification model.

Sensivity	Specifity	Balanced accuracy
0.611	0.620	0.615

Model Integrity+NIAID for reverse transcriptase was validated on external test set obtained by excluding Integrity and NIAID structures from ChEMBL data set. Prediction results for continuous data model and classification model are presented on figure 19 and table 25.

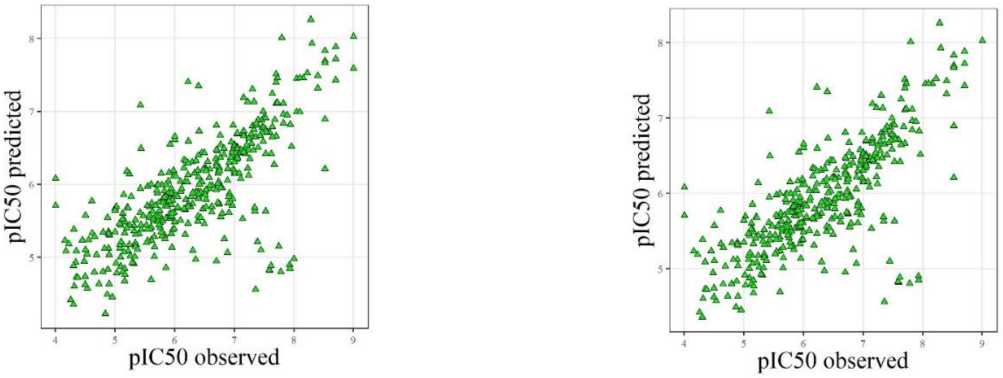

Figure 19. Observed versus predicted plots for predicting all structures ($R^2 = 0.500$) and structers in AD ($R^2 = 0.502$) for Integrity+NIAID reverse transcriptase model.

Table 25. Prediction results for Integrity+NIAID protease classification model.

Sensivity	Specifity	Balanced accuracy
0.596	0.867	0.732

Training Sets

7.1 Integrase set

Source	ID	pIC50
ChEMBL MOLREGNO	24222	5.785
ChEMBL MOLREGNO	37812	4.620
ChEMBL MOLREGNO	43417	5.523
ChEMBL MOLREGNO	47694	7.398
ChEMBL MOLREGNO	47726	7.301
ChEMBL MOLREGNO	48056	8.000
ChEMBL MOLREGNO	48302	5.745
ChEMBL MOLREGNO	49337	4.872
ChEMBL MOLREGNO	55288	6.155
ChEMBL MOLREGNO	72939	6.074
ChEMBL MOLREGNO	72977	4.377
ChEMBL MOLREGNO	72978	4.009
ChEMBL MOLREGNO	73160	4.824
ChEMBL MOLREGNO	73170	5.387
ChEMBL MOLREGNO	73697	6.784
ChEMBL MOLREGNO	73733	5.921
ChEMBL MOLREGNO	73771	7.585
ChEMBL MOLREGNO	73772	5.387
ChEMBL MOLREGNO	213630	4.400
ChEMBL MOLREGNO	213902	5.371
ChEMBL MOLREGNO	214079	5.130
ChEMBL MOLREGNO	249740	4.328

ChEMBL MOLREGNO	289182	5.337
ChEMBL MOLREGNO	306732	4.409
ChEMBL MOLREGNO	329475	7.562
ChEMBL MOLREGNO	330312	7.701
ChEMBL MOLREGNO	332034	4.796
ChEMBL MOLREGNO	340684	6.532
ChEMBL MOLREGNO	340743	6.586
ChEMBL MOLREGNO	353828	6.481
ChEMBL MOLREGNO	353829	6.721
ChEMBL MOLREGNO	353837	7.398
ChEMBL MOLREGNO	353896	7.301
ChEMBL MOLREGNO	353903	7.523
ChEMBL MOLREGNO	353912	7.155
ChEMBL MOLREGNO	353913	7.097
ChEMBL MOLREGNO	353915	6.602
ChEMBL MOLREGNO	353921	5.620
ChEMBL MOLREGNO	353922	7.046
ChEMBL MOLREGNO	353928	6.119
ChEMBL MOLREGNO	353929	5.921
ChEMBL MOLREGNO	353936	5.678
ChEMBL MOLREGNO	353937	6.456
ChEMBL MOLREGNO	361176	7.301
ChEMBL MOLREGNO	361188	6.658
ChEMBL MOLREGNO	361199	7.523
ChEMBL	361209	6.357

MOLREGNO		
ChEMBL MOLREGNO	361222	7.460
ChEMBL MOLREGNO	361273	7.070
ChEMBL MOLREGNO	361287	8.000
ChEMBL MOLREGNO	361305	5.097
ChEMBL MOLREGNO	377352	5.699
ChEMBL MOLREGNO	377570	7.038
ChEMBL MOLREGNO	377590	6.000
ChEMBL MOLREGNO	377592	6.151
ChEMBL MOLREGNO	377602	7.700
ChEMBL MOLREGNO	377699	6.886
ChEMBL MOLREGNO	378767	4.108
ChEMBL MOLREGNO	378768	4.000
ChEMBL MOLREGNO	378781	4.187
ChEMBL MOLREGNO	378784	4.569
ChEMBL MOLREGNO	378785	5.155
ChEMBL MOLREGNO	378786	4.638
ChEMBL MOLREGNO	378787	5.155
ChEMBL MOLREGNO	378788	5.523
ChEMBL MOLREGNO	378789	5.444
ChEMBL MOLREGNO	378791	6.420
ChEMBL MOLREGNO	378792	4.886
ChEMBL MOLREGNO	378793	5.770
ChEMBL MOLREGNO	378794	6.276
ChEMBL MOLREGNO	378795	4.456

ChEMBL MOLREGNO	378796	4.796
ChEMBL MOLREGNO	390400	7.104
ChEMBL MOLREGNO	390420	6.149
ChEMBL MOLREGNO	390425	7.361
ChEMBL MOLREGNO	390429	6.161
ChEMBL MOLREGNO	390433	7.000
ChEMBL MOLREGNO	390435	6.745
ChEMBL MOLREGNO	390436	6.699
ChEMBL MOLREGNO	390438	6.886
ChEMBL MOLREGNO	390440	7.523
ChEMBL MOLREGNO	390450	7.215
ChEMBL MOLREGNO	390451	7.699
ChEMBL MOLREGNO	390455	7.523
ChEMBL MOLREGNO	390458	7.678
ChEMBL MOLREGNO	390463	7.208
ChEMBL MOLREGNO	390469	7.155
ChEMBL MOLREGNO	390473	7.432
ChEMBL MOLREGNO	390476	6.854
ChEMBL MOLREGNO	390480	7.000
ChEMBL MOLREGNO	390482	7.301
ChEMBL MOLREGNO	390490	6.699
ChEMBL MOLREGNO	390491	6.602
ChEMBL MOLREGNO	390493	6.886
ChEMBL MOLREGNO	390494	7.387
ChEMBL	390497	7.858

MOLREGNO		
ChEMBL MOLREGNO	390498	5.398
ChEMBL MOLREGNO	390502	7.921
ChEMBL MOLREGNO	390505	8.398
ChEMBL MOLREGNO	390507	7.284
ChEMBL MOLREGNO	390508	7.741
ChEMBL MOLREGNO	390509	8.155
ChEMBL MOLREGNO	390510	7.898
ChEMBL MOLREGNO	390511	7.301
ChEMBL MOLREGNO	390512	7.481
ChEMBL MOLREGNO	390514	7.824
ChEMBL MOLREGNO	390516	7.699
ChEMBL MOLREGNO	390517	7.180
ChEMBL MOLREGNO	390518	7.149
ChEMBL MOLREGNO	390524	6.757
ChEMBL MOLREGNO	390526	7.208
ChEMBL MOLREGNO	390528	7.208
ChEMBL MOLREGNO	390529	7.721
ChEMBL MOLREGNO	390530	6.854
ChEMBL MOLREGNO	390532	7.678
ChEMBL MOLREGNO	390533	7.066
ChEMBL MOLREGNO	390535	7.553
ChEMBL MOLREGNO	390537	7.357
ChEMBL MOLREGNO	395120	7.096
ChEMBL MOLREGNO	395121	6.805

ChEMBL MOLREGNO	395123	6.678
ChEMBL MOLREGNO	395124	6.955
ChEMBL MOLREGNO	395126	4.780
ChEMBL MOLREGNO	395127	7.000
ChEMBL MOLREGNO	395128	6.699
ChEMBL MOLREGNO	395129	5.602
ChEMBL MOLREGNO	395130	8.000
ChEMBL MOLREGNO	395131	7.301
ChEMBL MOLREGNO	395132	7.700
ChEMBL MOLREGNO	395133	7.040
ChEMBL MOLREGNO	395134	7.300
ChEMBL MOLREGNO	395135	6.260
ChEMBL MOLREGNO	395136	6.960
ChEMBL MOLREGNO	395137	7.400
ChEMBL MOLREGNO	395138	7.150
ChEMBL MOLREGNO	395139	6.890
ChEMBL MOLREGNO	395141	7.300
ChEMBL MOLREGNO	395142	4.300
ChEMBL MOLREGNO	395143	7.700
ChEMBL MOLREGNO	395145	6.790
ChEMBL MOLREGNO	395146	7.150
ChEMBL MOLREGNO	395148	8.000
ChEMBL MOLREGNO	399116	4.174
ChEMBL MOLREGNO	399137	4.161
ChEMBL	399138	4.699

MOLREGNO		
ChEMBL MOLREGNO	399139	4.377
ChEMBL MOLREGNO	399140	4.585
ChEMBL MOLREGNO	399141	4.523
ChEMBL MOLREGNO	399142	4.367
ChEMBL MOLREGNO	399143	4.367
ChEMBL MOLREGNO	399145	4.409
ChEMBL MOLREGNO	399146	4.108
ChEMBL MOLREGNO	399147	4.046
ChEMBL MOLREGNO	399148	4.678
ChEMBL MOLREGNO	399149	4.481
ChEMBL MOLREGNO	399150	5.156
ChEMBL MOLREGNO	399151	4.678
ChEMBL MOLREGNO	399152	4.046
ChEMBL MOLREGNO	399153	4.081
ChEMBL MOLREGNO	400056	5.983
ChEMBL MOLREGNO	400058	7.000
ChEMBL MOLREGNO	400060	7.398
ChEMBL MOLREGNO	400061	6.638
ChEMBL MOLREGNO	400062	6.432
ChEMBL MOLREGNO	400064	7.398
ChEMBL MOLREGNO	400065	6.420
ChEMBL MOLREGNO	400067	7.398
ChEMBL MOLREGNO	400068	7.699
ChEMBL MOLREGNO	400070	7.523

ChEMBL MOLREGNO	400071	7.699
ChEMBL MOLREGNO	401455	7.398
ChEMBL MOLREGNO	401456	6.658
ChEMBL MOLREGNO	401457	7.398
ChEMBL MOLREGNO	401460	6.959
ChEMBL MOLREGNO	401462	7.523
ChEMBL MOLREGNO	401465	7.699
ChEMBL MOLREGNO	401466	8.000
ChEMBL MOLREGNO	401469	7.097
ChEMBL MOLREGNO	401470	8.000
ChEMBL MOLREGNO	401473	7.097
ChEMBL MOLREGNO	401474	7.222
ChEMBL MOLREGNO	401475	7.398
ChEMBL MOLREGNO	406995	4.051
ChEMBL MOLREGNO	406996	4.036
ChEMBL MOLREGNO	407004	4.180
ChEMBL MOLREGNO	407006	5.208
ChEMBL MOLREGNO	407009	4.310
ChEMBL MOLREGNO	407011	4.046
ChEMBL MOLREGNO	407012	4.523
ChEMBL MOLREGNO	407014	4.143
ChEMBL MOLREGNO	407016	4.745
ChEMBL MOLREGNO	407017	4.921
ChEMBL MOLREGNO	407018	4.699
ChEMBL	407020	4.770

MOLREGNO		
ChEMBL MOLREGNO	407021	4.222
ChEMBL MOLREGNO	407022	4.337
ChEMBL MOLREGNO	407023	6.000
ChEMBL MOLREGNO	407024	4.553
ChEMBL MOLREGNO	407025	4.523
ChEMBL MOLREGNO	407026	4.745
ChEMBL MOLREGNO	407027	4.481
ChEMBL MOLREGNO	407028	4.319
ChEMBL MOLREGNO	407029	4.155
ChEMBL MOLREGNO	407030	4.092
ChEMBL MOLREGNO	407031	4.585
ChEMBL MOLREGNO	407033	4.602
ChEMBL MOLREGNO	407034	4.051
ChEMBL MOLREGNO	407035	4.114
ChEMBL MOLREGNO	407037	4.081
ChEMBL MOLREGNO	407038	4.244
ChEMBL MOLREGNO	407039	4.444
ChEMBL MOLREGNO	407040	4.237
ChEMBL MOLREGNO	407042	4.796
ChEMBL MOLREGNO	407043	4.071
ChEMBL MOLREGNO	407044	4.699
ChEMBL MOLREGNO	407045	4.000
ChEMBL MOLREGNO	407049	4.959
ChEMBL MOLREGNO	407051	4.000

ChEMBL MOLREGNO	407053	4.921
ChEMBL MOLREGNO	407054	4.097
ChEMBL MOLREGNO	407055	4.000
ChEMBL MOLREGNO	407056	4.000
ChEMBL MOLREGNO	407058	4.276
ChEMBL MOLREGNO	407059	5.398
ChEMBL MOLREGNO	407062	4.495
ChEMBL MOLREGNO	407066	4.538
ChEMBL MOLREGNO	407068	6.222
ChEMBL MOLREGNO	407072	4.155
ChEMBL MOLREGNO	407073	4.886
ChEMBL MOLREGNO	407074	4.367
ChEMBL MOLREGNO	407078	4.076
ChEMBL MOLREGNO	407080	4.432
ChEMBL MOLREGNO	407100	4.222
ChEMBL MOLREGNO	409915	4.886
ChEMBL MOLREGNO	409916	4.357
ChEMBL MOLREGNO	409917	4.921
ChEMBL MOLREGNO	409920	4.921
ChEMBL MOLREGNO	409934	4.770
ChEMBL MOLREGNO	409936	4.870
ChEMBL MOLREGNO	409938	4.745
ChEMBL MOLREGNO	410312	5.018
ChEMBL MOLREGNO	410313	4.863
ChEMBL	410314	4.355

MOLREGNO		
ChEMBL MOLREGNO	410315	4.447
ChEMBL MOLREGNO	410316	4.039
ChEMBL MOLREGNO	410317	4.440
ChEMBL MOLREGNO	410319	4.011
ChEMBL MOLREGNO	410699	8.000
ChEMBL MOLREGNO	410701	7.854
ChEMBL MOLREGNO	410704	8.155
ChEMBL MOLREGNO	410709	7.523
ChEMBL MOLREGNO	410714	7.347
ChEMBL MOLREGNO	410719	8.000
ChEMBL MOLREGNO	410724	6.959
ChEMBL MOLREGNO	410728	8.000
ChEMBL MOLREGNO	410863	7.292
ChEMBL MOLREGNO	417542	5.523
ChEMBL MOLREGNO	417544	5.222
ChEMBL MOLREGNO	417546	5.046
ChEMBL MOLREGNO	417547	5.046
ChEMBL MOLREGNO	417549	5.097
ChEMBL MOLREGNO	417550	4.770
ChEMBL MOLREGNO	417552	4.770
ChEMBL MOLREGNO	417553	5.301
ChEMBL MOLREGNO	417554	4.678
ChEMBL MOLREGNO	417556	4.921
ChEMBL MOLREGNO	417557	4.509

ChEMBL MOLREGNO	417558	4.237
ChEMBL MOLREGNO	417559	4.569
ChEMBL MOLREGNO	417563	4.215
ChEMBL MOLREGNO	419258	6.301
ChEMBL MOLREGNO	419259	5.548
ChEMBL MOLREGNO	419260	4.701
ChEMBL MOLREGNO	419261	4.645
ChEMBL MOLREGNO	419263	4.301
ChEMBL MOLREGNO	419264	4.509
ChEMBL MOLREGNO	419265	4.456
ChEMBL MOLREGNO	419267	5.077
ChEMBL MOLREGNO	419268	4.456
ChEMBL MOLREGNO	419269	4.658
ChEMBL MOLREGNO	419270	5.097
ChEMBL MOLREGNO	419282	5.222
ChEMBL MOLREGNO	419284	4.678
ChEMBL MOLREGNO	419285	4.481
ChEMBL MOLREGNO	419286	6.678
ChEMBL MOLREGNO	419291	4.387
ChEMBL MOLREGNO	419294	5.921
ChEMBL MOLREGNO	419298	4.959
ChEMBL MOLREGNO	419299	6.222
ChEMBL MOLREGNO	419300	6.092
ChEMBL MOLREGNO	419302	4.276
ChEMBL	419303	4.268

MOLREGNO		
ChEMBL MOLREGNO	419312	4.910
ChEMBL MOLREGNO	419313	4.553
ChEMBL MOLREGNO	419314	6.131
ChEMBL MOLREGNO	419315	5.000
ChEMBL MOLREGNO	419316	6.796
ChEMBL MOLREGNO	419317	4.886
ChEMBL MOLREGNO	421608	5.000
ChEMBL MOLREGNO	421611	5.745
ChEMBL MOLREGNO	421612	5.585
ChEMBL MOLREGNO	421613	5.469
ChEMBL MOLREGNO	421614	5.678
ChEMBL MOLREGNO	422253	7.113
ChEMBL MOLREGNO	427303	6.310
ChEMBL MOLREGNO	427394	6.638
ChEMBL MOLREGNO	427440	6.745
ChEMBL MOLREGNO	427489	7.046
ChEMBL MOLREGNO	427491	7.155
ChEMBL MOLREGNO	427492	7.046
ChEMBL MOLREGNO	427494	7.155
ChEMBL MOLREGNO	427551	7.699
ChEMBL MOLREGNO	427552	7.699
ChEMBL MOLREGNO	427607	7.699
ChEMBL MOLREGNO	427608	7.699
ChEMBL MOLREGNO	427610	7.699

ChEMBL MOLREGNO	427659	7.699
ChEMBL MOLREGNO	427661	8.000
ChEMBL MOLREGNO	427663	7.222
ChEMBL MOLREGNO	427706	7.699
ChEMBL MOLREGNO	427708	7.699
ChEMBL MOLREGNO	427761	8.000
ChEMBL MOLREGNO	427762	8.000
ChEMBL MOLREGNO	427763	8.000
ChEMBL MOLREGNO	428152	7.523
ChEMBL MOLREGNO	428155	6.721
ChEMBL MOLREGNO	428191	6.658
ChEMBL MOLREGNO	428194	6.569
ChEMBL MOLREGNO	428196	5.633
ChEMBL MOLREGNO	428235	5.578
ChEMBL MOLREGNO	428236	6.180
ChEMBL MOLREGNO	428239	5.122
ChEMBL MOLREGNO	428240	5.631
ChEMBL MOLREGNO	428280	4.035
ChEMBL MOLREGNO	429799	7.155
ChEMBL MOLREGNO	429840	5.432
ChEMBL MOLREGNO	441227	8.398
ChEMBL MOLREGNO	441230	8.301
ChEMBL MOLREGNO	441231	7.367
ChEMBL MOLREGNO	441233	7.770
ChEMBL	441284	7.180

MOLREGNO		
ChEMBL MOLREGNO	441285	7.469
ChEMBL MOLREGNO	441286	7.638
ChEMBL MOLREGNO	441333	7.745
ChEMBL MOLREGNO	441380	8.301
ChEMBL MOLREGNO	441381	7.284
ChEMBL MOLREGNO	441382	7.337
ChEMBL MOLREGNO	441384	8.301
ChEMBL MOLREGNO	441459	7.362
ChEMBL MOLREGNO	441849	7.222
ChEMBL MOLREGNO	441894	7.187
ChEMBL MOLREGNO	441896	7.886
ChEMBL MOLREGNO	441939	7.229
ChEMBL MOLREGNO	441941	8.398
ChEMBL MOLREGNO	444673	4.387
ChEMBL MOLREGNO	446593	5.398
ChEMBL MOLREGNO	446594	5.301
ChEMBL MOLREGNO	446595	4.903
ChEMBL MOLREGNO	446596	5.097
ChEMBL MOLREGNO	446597	5.222
ChEMBL MOLREGNO	446598	4.347
ChEMBL MOLREGNO	446599	4.066
ChEMBL MOLREGNO	446601	4.903
ChEMBL MOLREGNO	451464	4.367
ChEMBL MOLREGNO	451465	4.276

ChEMBL MOLREGNO	451466	4.456
ChEMBL MOLREGNO	451467	4.092
ChEMBL MOLREGNO	451468	4.409
ChEMBL MOLREGNO	451520	4.377
ChEMBL MOLREGNO	451521	4.509
ChEMBL MOLREGNO	451522	4.538
ChEMBL MOLREGNO	451523	4.276
ChEMBL MOLREGNO	451583	5.398
ChEMBL MOLREGNO	451584	5.222
ChEMBL MOLREGNO	451585	5.398
ChEMBL MOLREGNO	451586	5.222
ChEMBL MOLREGNO	451646	4.081
ChEMBL MOLREGNO	451647	4.481
ChEMBL MOLREGNO	451649	4.444
ChEMBL MOLREGNO	451713	4.114
ChEMBL MOLREGNO	451714	4.032
ChEMBL MOLREGNO	451715	4.284
ChEMBL MOLREGNO	451716	4.108
ChEMBL MOLREGNO	451717	4.155
ChEMBL MOLREGNO	451787	4.046
ChEMBL MOLREGNO	451788	5.310
ChEMBL MOLREGNO	457208	6.022
ChEMBL MOLREGNO	457209	6.886
ChEMBL MOLREGNO	457210	6.444
ChEMBL	457211	7.959

MOLREGNO		
ChEMBL MOLREGNO	457212	8.699
ChEMBL MOLREGNO	457905	6.886
ChEMBL MOLREGNO	457906	6.194
ChEMBL MOLREGNO	457907	5.987
ChEMBL MOLREGNO	457908	5.553
ChEMBL MOLREGNO	457909	5.201
ChEMBL MOLREGNO	457971	5.620
ChEMBL MOLREGNO	457972	5.337
ChEMBL MOLREGNO	457973	5.456
ChEMBL MOLREGNO	457974	6.886
ChEMBL MOLREGNO	457975	7.377
ChEMBL MOLREGNO	457976	5.102
ChEMBL MOLREGNO	458259	5.301
ChEMBL MOLREGNO	458315	5.046
ChEMBL MOLREGNO	458317	5.222
ChEMBL MOLREGNO	458318	6.046
ChEMBL MOLREGNO	458319	4.854
ChEMBL MOLREGNO	458320	7.244
ChEMBL MOLREGNO	461356	8.398
ChEMBL MOLREGNO	461357	8.398
ChEMBL MOLREGNO	461358	8.301
ChEMBL MOLREGNO	461413	8.398
ChEMBL MOLREGNO	461486	7.347
ChEMBL MOLREGNO	461487	8.222

ChEMBL MOLREGNO	463788	4.699
ChEMBL MOLREGNO	463789	4.699
ChEMBL MOLREGNO	464398	5.046
ChEMBL MOLREGNO	464400	4.824
ChEMBL MOLREGNO	464401	4.854
ChEMBL MOLREGNO	464402	5.252
ChEMBL MOLREGNO	464403	5.319
ChEMBL MOLREGNO	464404	4.071
ChEMBL MOLREGNO	464430	4.398
ChEMBL MOLREGNO	464441	4.921
ChEMBL MOLREGNO	464442	4.921
ChEMBL MOLREGNO	464450	4.939
ChEMBL MOLREGNO	464549	4.658
ChEMBL MOLREGNO	467004	7.000
ChEMBL MOLREGNO	467005	8.222
ChEMBL MOLREGNO	467006	8.398
ChEMBL MOLREGNO	467070	8.301
ChEMBL MOLREGNO	467071	7.027
ChEMBL MOLREGNO	467139	7.745
ChEMBL MOLREGNO	470381	6.638
ChEMBL MOLREGNO	470382	8.523
ChEMBL MOLREGNO	470383	8.699
ChEMBL MOLREGNO	470384	8.155
ChEMBL MOLREGNO	470385	8.097
ChEMBL	470443	7.745

MOLREGNO

ChEMBL MOLREGNO	470444	7.921
ChEMBL MOLREGNO	470445	8.000
ChEMBL MOLREGNO	470446	7.824
ChEMBL MOLREGNO	470447	8.398
ChEMBL MOLREGNO	470515	7.824
ChEMBL MOLREGNO	470516	7.699
ChEMBL MOLREGNO	470517	7.585
ChEMBL MOLREGNO	470582	7.678
ChEMBL MOLREGNO	470583	8.398
ChEMBL MOLREGNO	470584	8.046
ChEMBL MOLREGNO	470585	7.699
ChEMBL MOLREGNO	470647	7.824
ChEMBL MOLREGNO	470648	8.155
ChEMBL MOLREGNO	470649	8.155
ChEMBL MOLREGNO	470650	8.097
ChEMBL MOLREGNO	470720	8.222
ChEMBL MOLREGNO	470721	8.398
ChEMBL MOLREGNO	476350	4.092
ChEMBL MOLREGNO	476427	4.585
ChEMBL MOLREGNO	476428	4.658
ChEMBL MOLREGNO	476429	4.745
ChEMBL MOLREGNO	476430	4.523
ChEMBL MOLREGNO	476431	4.377
ChEMBL MOLREGNO	476498	4.252

ChEMBL MOLREGNO	476500	4.387
ChEMBL MOLREGNO	476501	5.097
ChEMBL MOLREGNO	476568	4.319
ChEMBL MOLREGNO	476569	4.137
ChEMBL MOLREGNO	476570	4.161
ChEMBL MOLREGNO	476571	4.022
ChEMBL MOLREGNO	476572	4.036
ChEMBL MOLREGNO	476654	4.244
ChEMBL MOLREGNO	477588	7.444
ChEMBL MOLREGNO	477589	7.155
ChEMBL MOLREGNO	477590	7.745
ChEMBL MOLREGNO	477591	8.046
ChEMBL MOLREGNO	477593	7.638
ChEMBL MOLREGNO	477594	7.301
ChEMBL MOLREGNO	477595	7.678
ChEMBL MOLREGNO	477597	7.444
ChEMBL MOLREGNO	477598	7.745
ChEMBL MOLREGNO	477599	7.678
ChEMBL MOLREGNO	477663	7.658
ChEMBL MOLREGNO	477664	6.886
ChEMBL MOLREGNO	477665	6.770
ChEMBL MOLREGNO	477666	6.733
ChEMBL MOLREGNO	477667	7.620
ChEMBL MOLREGNO	477668	6.733
ChEMBL	477669	7.301

MOLREGNO		
ChEMBL MOLREGNO	477670	7.678
ChEMBL MOLREGNO	477671	7.745
ChEMBL MOLREGNO	477672	7.585
ChEMBL MOLREGNO	477673	7.721
ChEMBL MOLREGNO	477675	7.678
ChEMBL MOLREGNO	477676	7.678
ChEMBL MOLREGNO	478458	6.456
ChEMBL MOLREGNO	490767	4.959
ChEMBL MOLREGNO	490769	4.638
ChEMBL MOLREGNO	490875	7.699
ChEMBL MOLREGNO	490876	7.854
ChEMBL MOLREGNO	490877	7.398
ChEMBL MOLREGNO	490878	7.620
ChEMBL MOLREGNO	490955	7.481
ChEMBL MOLREGNO	490956	6.910
ChEMBL MOLREGNO	490957	7.585
ChEMBL MOLREGNO	491790	4.409
ChEMBL MOLREGNO	491864	4.000
ChEMBL MOLREGNO	494499	6.143
ChEMBL MOLREGNO	494500	5.646
ChEMBL MOLREGNO	494574	7.921
ChEMBL MOLREGNO	494575	6.174
ChEMBL MOLREGNO	494576	6.398
ChEMBL MOLREGNO	494577	7.155

ChEMBL MOLREGNO	494666	5.807
ChEMBL MOLREGNO	494667	5.562
ChEMBL MOLREGNO	494668	5.939
ChEMBL MOLREGNO	494669	6.086
ChEMBL MOLREGNO	494753	5.851
ChEMBL MOLREGNO	494754	7.398
ChEMBL MOLREGNO	494755	7.523
ChEMBL MOLREGNO	494756	6.921
ChEMBL MOLREGNO	494757	7.699
ChEMBL MOLREGNO	494828	7.699
ChEMBL MOLREGNO	494829	7.097
ChEMBL MOLREGNO	494830	6.222
ChEMBL MOLREGNO	494831	6.620
ChEMBL MOLREGNO	494934	6.553
ChEMBL MOLREGNO	494935	6.796
ChEMBL MOLREGNO	494936	7.000
ChEMBL MOLREGNO	495264	5.000
ChEMBL MOLREGNO	495265	4.523
ChEMBL MOLREGNO	495266	4.854
ChEMBL MOLREGNO	495267	4.854
ChEMBL MOLREGNO	495268	4.757
ChEMBL MOLREGNO	495269	5.000
ChEMBL MOLREGNO	495343	4.952
ChEMBL MOLREGNO	495473	5.038
ChEMBL	495474	4.824

MOLREGNO		
ChEMBL MOLREGNO	496083	4.921
ChEMBL MOLREGNO	496155	4.701
ChEMBL MOLREGNO	496156	4.523
ChEMBL MOLREGNO	496157	4.187
ChEMBL MOLREGNO	496158	4.301
ChEMBL MOLREGNO	496159	5.155
ChEMBL MOLREGNO	496160	5.523
ChEMBL MOLREGNO	496223	4.602
ChEMBL MOLREGNO	496224	4.602
ChEMBL MOLREGNO	496225	5.155
ChEMBL MOLREGNO	496226	4.046
ChEMBL MOLREGNO	496228	5.097
ChEMBL MOLREGNO	496298	4.935
ChEMBL MOLREGNO	496299	4.770
ChEMBL MOLREGNO	496300	5.523
ChEMBL MOLREGNO	496301	4.898
ChEMBL MOLREGNO	496302	4.553
ChEMBL MOLREGNO	501832	4.160
ChEMBL MOLREGNO	501980	4.010
ChEMBL MOLREGNO	506530	5.137
ChEMBL MOLREGNO	510971	5.963
ChEMBL MOLREGNO	510972	6.137
ChEMBL MOLREGNO	510973	6.523
ChEMBL MOLREGNO	511158	7.500

ChEMBL MOLREGNO	511159	7.270
ChEMBL MOLREGNO	511160	6.975
ChEMBL MOLREGNO	511161	7.340
ChEMBL MOLREGNO	511162	6.465
ChEMBL MOLREGNO	511212	6.222
ChEMBL MOLREGNO	511213	4.618
ChEMBL MOLREGNO	511214	4.783
ChEMBL MOLREGNO	511232	7.303
ChEMBL MOLREGNO	511440	7.149
ChEMBL MOLREGNO	511441	5.580
ChEMBL MOLREGNO	511443	4.252
ChEMBL MOLREGNO	511444	4.601
ChEMBL MOLREGNO	511519	7.845
ChEMBL MOLREGNO	511564	5.678
ChEMBL MOLREGNO	511565	5.796
ChEMBL MOLREGNO	511566	4.231
ChEMBL MOLREGNO	512025	8.000
ChEMBL MOLREGNO	512101	4.167
ChEMBL MOLREGNO	512102	4.013
ChEMBL MOLREGNO	512103	4.569
ChEMBL MOLREGNO	512157	8.000
ChEMBL MOLREGNO	512167	4.092
ChEMBL MOLREGNO	512168	4.000
ChEMBL MOLREGNO	512276	6.265
ChEMBL	512277	6.437

MOLREGNO		
ChEMBL MOLREGNO	512278	5.903
ChEMBL MOLREGNO	512350	4.935
ChEMBL MOLREGNO	512351	5.100
ChEMBL MOLREGNO	512352	5.149
ChEMBL MOLREGNO	512353	4.889
ChEMBL MOLREGNO	512406	4.733
ChEMBL MOLREGNO	512407	6.699
ChEMBL MOLREGNO	512408	5.886
ChEMBL MOLREGNO	512427	5.577
ChEMBL MOLREGNO	512428	5.603
ChEMBL MOLREGNO	512429	7.310
ChEMBL MOLREGNO	512430	6.081
ChEMBL MOLREGNO	512575	7.000
ChEMBL MOLREGNO	512576	7.699
ChEMBL MOLREGNO	513550	6.046
ChEMBL MOLREGNO	518343	7.000
ChEMBL MOLREGNO	518344	7.854
ChEMBL MOLREGNO	518345	6.222
ChEMBL MOLREGNO	518346	7.097
ChEMBL MOLREGNO	518347	6.577
ChEMBL MOLREGNO	519357	7.000
ChEMBL MOLREGNO	519403	5.678
ChEMBL MOLREGNO	521906	7.553
ChEMBL MOLREGNO	554482	5.222

ChEMBL MOLREGNO	554484	5.174
ChEMBL MOLREGNO	554681	5.523
ChEMBL MOLREGNO	554683	5.462
ChEMBL MOLREGNO	554697	5.602
ChEMBL MOLREGNO	554705	4.745
ChEMBL MOLREGNO	554706	5.398
ChEMBL MOLREGNO	554707	4.602
ChEMBL MOLREGNO	554720	5.097
ChEMBL MOLREGNO	554721	6.523
ChEMBL MOLREGNO	554745	6.699
ChEMBL MOLREGNO	554756	5.886
ChEMBL MOLREGNO	554769	6.398
ChEMBL MOLREGNO	554933	8.222
ChEMBL MOLREGNO	555265	7.495
ChEMBL MOLREGNO	555281	7.208
ChEMBL MOLREGNO	555303	8.301
ChEMBL MOLREGNO	555312	8.222
ChEMBL MOLREGNO	555507	5.699
ChEMBL MOLREGNO	555522	5.699
ChEMBL MOLREGNO	555785	6.260
ChEMBL MOLREGNO	555786	7.000
ChEMBL MOLREGNO	555787	7.398
ChEMBL MOLREGNO	555788	7.301
ChEMBL MOLREGNO	555789	7.222
ChEMBL	556428	5.398

MOLREGNO		
ChEMBL MOLREGNO	556440	4.836
ChEMBL MOLREGNO	556441	4.398
ChEMBL MOLREGNO	556460	5.699
ChEMBL MOLREGNO	556472	6.638
ChEMBL MOLREGNO	556485	5.456
ChEMBL MOLREGNO	556880	8.222
ChEMBL MOLREGNO	556886	8.046
ChEMBL MOLREGNO	557002	8.155
ChEMBL MOLREGNO	557016	8.097
ChEMBL MOLREGNO	557027	8.301
ChEMBL MOLREGNO	557274	5.770
ChEMBL MOLREGNO	557510	6.921
ChEMBL MOLREGNO	557511	7.523
ChEMBL MOLREGNO	557562	6.921
ChEMBL MOLREGNO	558125	4.678
ChEMBL MOLREGNO	558126	6.046
ChEMBL MOLREGNO	558134	4.569
ChEMBL MOLREGNO	558135	4.903
ChEMBL MOLREGNO	558138	6.046
ChEMBL MOLREGNO	558139	4.018
ChEMBL MOLREGNO	558154	5.357
ChEMBL MOLREGNO	558166	6.927
ChEMBL MOLREGNO	558180	6.523
ChEMBL MOLREGNO	558216	5.886

ChEMBL MOLREGNO	558370	8.097
ChEMBL MOLREGNO	558631	8.523
ChEMBL MOLREGNO	558703	8.155
ChEMBL MOLREGNO	558737	7.678
ChEMBL MOLREGNO	558747	8.301
ChEMBL MOLREGNO	558943	5.699
ChEMBL MOLREGNO	558960	5.824
ChEMBL MOLREGNO	558969	4.301
ChEMBL MOLREGNO	558970	4.921
ChEMBL MOLREGNO	558986	5.796
ChEMBL MOLREGNO	559701	4.623
ChEMBL MOLREGNO	559704	5.086
ChEMBL MOLREGNO	559705	5.252
ChEMBL MOLREGNO	559909	6.387
ChEMBL MOLREGNO	559910	5.573
ChEMBL MOLREGNO	559920	5.520
ChEMBL MOLREGNO	559921	5.335
ChEMBL MOLREGNO	559927	6.301
ChEMBL MOLREGNO	559928	4.699
ChEMBL MOLREGNO	559929	5.658
ChEMBL MOLREGNO	559964	6.398
ChEMBL MOLREGNO	559988	6.097
ChEMBL MOLREGNO	559989	6.921
ChEMBL MOLREGNO	559998	6.222
ChEMBL	559999	5.398

MOLREGNO		
ChEMBL MOLREGNO	560175	8.222
ChEMBL MOLREGNO	560180	8.301
ChEMBL MOLREGNO	560445	7.602
ChEMBL MOLREGNO	560514	7.523
ChEMBL MOLREGNO	560526	7.796
ChEMBL MOLREGNO	560527	8.222
ChEMBL MOLREGNO	560538	8.097
ChEMBL MOLREGNO	560551	7.119
ChEMBL MOLREGNO	560552	7.678
ChEMBL MOLREGNO	560561	8.097
ChEMBL MOLREGNO	560571	8.046
ChEMBL MOLREGNO	560751	5.523
ChEMBL MOLREGNO	560775	5.796
ChEMBL MOLREGNO	561041	8.398
ChEMBL MOLREGNO	561046	7.097
ChEMBL MOLREGNO	561047	7.046
ChEMBL MOLREGNO	561056	7.699
ChEMBL MOLREGNO	561057	7.699
ChEMBL MOLREGNO	561102	7.523
ChEMBL MOLREGNO	561532	5.046
ChEMBL MOLREGNO	561541	5.237
ChEMBL MOLREGNO	561741	5.398
ChEMBL MOLREGNO	561752	5.222
ChEMBL MOLREGNO	561753	5.921

ChEMBL MOLREGNO	561755	4.432
ChEMBL MOLREGNO	561756	5.114
ChEMBL MOLREGNO	561771	5.051
ChEMBL MOLREGNO	561772	5.288
ChEMBL MOLREGNO	561784	6.000
ChEMBL MOLREGNO	561806	6.046
ChEMBL MOLREGNO	561824	5.745
ChEMBL MOLREGNO	562006	8.699
ChEMBL MOLREGNO	562011	8.222
ChEMBL MOLREGNO	562248	8.046
ChEMBL MOLREGNO	562326	8.301
ChEMBL MOLREGNO	562357	8.523
ChEMBL MOLREGNO	562562	5.347
ChEMBL MOLREGNO	562582	5.699
ChEMBL MOLREGNO	562829	7.699
ChEMBL MOLREGNO	563237	5.237
ChEMBL MOLREGNO	563411	5.391
ChEMBL MOLREGNO	563418	6.155
ChEMBL MOLREGNO	563420	5.502
ChEMBL MOLREGNO	563427	4.879
ChEMBL MOLREGNO	563428	4.252
ChEMBL MOLREGNO	563439	5.699
ChEMBL MOLREGNO	563440	5.569
ChEMBL MOLREGNO	563441	5.432
ChEMBL	563450	6.403

MOLREGNO		
ChEMBL MOLREGNO	563483	6.301
ChEMBL MOLREGNO	563766	8.301
ChEMBL MOLREGNO	563935	7.678
ChEMBL MOLREGNO	564036	8.000
ChEMBL MOLREGNO	564037	7.824
ChEMBL MOLREGNO	564070	7.921
ChEMBL MOLREGNO	564261	5.097
ChEMBL MOLREGNO	564278	6.155
ChEMBL MOLREGNO	564279	6.155
ChEMBL MOLREGNO	564280	6.046
ChEMBL MOLREGNO	564290	5.824
ChEMBL MOLREGNO	564535	7.000
ChEMBL MOLREGNO	564536	7.699
ChEMBL MOLREGNO	564550	6.602
ChEMBL MOLREGNO	565194	5.202
ChEMBL MOLREGNO	565206	4.658
ChEMBL MOLREGNO	565236	6.398
ChEMBL MOLREGNO	565237	6.398
ChEMBL MOLREGNO	565250	5.699
ChEMBL MOLREGNO	565262	6.509
ChEMBL MOLREGNO	565475	7.208
ChEMBL MOLREGNO	565476	7.886
ChEMBL MOLREGNO	565739	8.155
ChEMBL MOLREGNO	565748	8.398

ChEMBL MOLREGNO	565825	7.699
ChEMBL MOLREGNO	565826	8.301
ChEMBL MOLREGNO	565842	8.301
ChEMBL MOLREGNO	566061	5.602
ChEMBL MOLREGNO	566068	5.886
ChEMBL MOLREGNO	566094	5.699
ChEMBL MOLREGNO	566328	7.699
ChEMBL MOLREGNO	566369	7.770
ChEMBL MOLREGNO	566838	5.009
ChEMBL MOLREGNO	567012	4.602
ChEMBL MOLREGNO	567016	6.523
ChEMBL MOLREGNO	567017	5.198
ChEMBL MOLREGNO	567018	5.215
ChEMBL MOLREGNO	567030	6.000
ChEMBL MOLREGNO	567031	7.000
ChEMBL MOLREGNO	567032	4.699
ChEMBL MOLREGNO	567076	6.770
ChEMBL MOLREGNO	567077	6.046
ChEMBL MOLREGNO	567090	5.734
ChEMBL MOLREGNO	567107	6.097
ChEMBL MOLREGNO	567289	8.699
ChEMBL MOLREGNO	567300	7.161
ChEMBL MOLREGNO	567620	8.222
ChEMBL MOLREGNO	567654	8.155
ChEMBL	567681	8.155

MOLREGNO		
ChEMBL MOLREGNO	567914	5.602
ChEMBL MOLREGNO	567929	5.523
ChEMBL MOLREGNO	568061	5.721
ChEMBL MOLREGNO	568177	7.761
ChEMBL MOLREGNO	568190	7.854
ChEMBL MOLREGNO	568488	6.301
ChEMBL MOLREGNO	568489	5.125
ChEMBL MOLREGNO	568491	6.337
ChEMBL MOLREGNO	568493	5.620
ChEMBL MOLREGNO	568494	5.602
ChEMBL MOLREGNO	568495	5.246
ChEMBL MOLREGNO	568496	6.745
ChEMBL MOLREGNO	568497	6.125
ChEMBL MOLREGNO	568498	6.602
ChEMBL MOLREGNO	568501	6.854
ChEMBL MOLREGNO	568502	4.943
ChEMBL MOLREGNO	568503	6.658
ChEMBL MOLREGNO	568504	6.260
ChEMBL MOLREGNO	568505	6.481
ChEMBL MOLREGNO	568506	6.886
ChEMBL MOLREGNO	568507	6.301
ChEMBL MOLREGNO	568510	6.824
ChEMBL MOLREGNO	568854	7.398
ChEMBL MOLREGNO	568855	7.699

ChEMBL MOLREGNO	568858	7.523
ChEMBL MOLREGNO	568859	7.699
ChEMBL MOLREGNO	568868	7.398
ChEMBL MOLREGNO	573755	4.076
ChEMBL MOLREGNO	573788	6.602
ChEMBL MOLREGNO	573849	5.456
ChEMBL MOLREGNO	573850	5.134
ChEMBL MOLREGNO	573861	5.433
ChEMBL MOLREGNO	573862	6.022
ChEMBL MOLREGNO	573877	5.924
ChEMBL MOLREGNO	573878	5.266
ChEMBL MOLREGNO	573913	5.495
ChEMBL MOLREGNO	573916	6.602
ChEMBL MOLREGNO	575625	6.143
ChEMBL MOLREGNO	575626	4.613
ChEMBL MOLREGNO	575666	6.018
ChEMBL MOLREGNO	575667	5.167
ChEMBL MOLREGNO	575668	5.398
ChEMBL MOLREGNO	575678	4.155
ChEMBL MOLREGNO	575679	4.765
ChEMBL MOLREGNO	575696	4.857
ChEMBL MOLREGNO	575712	4.979
ChEMBL MOLREGNO	575736	5.046
ChEMBL MOLREGNO	575776	6.097
ChEMBL	575777	4.638

MOLREGNO		
ChEMBL MOLREGNO	575828	5.333
ChEMBL MOLREGNO	575842	4.546
ChEMBL MOLREGNO	615071	4.300
ChEMBL MOLREGNO	615072	4.770
ChEMBL MOLREGNO	615093	7.000
ChEMBL MOLREGNO	615094	6.700
ChEMBL MOLREGNO	615095	5.600
ChEMBL MOLREGNO	615096	8.000
ChEMBL MOLREGNO	615097	7.300
ChEMBL MOLREGNO	615379	7.520
ChEMBL MOLREGNO	630555	4.167
ChEMBL MOLREGNO	685569	7.523
ChEMBL MOLREGNO	685570	6.229
ChEMBL MOLREGNO	685571	4.895
ChEMBL MOLREGNO	685572	7.222
ChEMBL MOLREGNO	685573	6.569
ChEMBL MOLREGNO	685634	6.886
ChEMBL MOLREGNO	685635	7.523
ChEMBL MOLREGNO	685636	6.921
ChEMBL MOLREGNO	685637	6.602
ChEMBL MOLREGNO	685638	7.046
ChEMBL MOLREGNO	685696	5.258
ChEMBL MOLREGNO	685697	5.339
ChEMBL MOLREGNO	686270	6.137

ChEMBL MOLREGNO	686332	5.539
ChEMBL MOLREGNO	686333	5.083
ChEMBL MOLREGNO	686334	5.036
ChEMBL MOLREGNO	686335	5.592
ChEMBL MOLREGNO	686387	5.532
ChEMBL MOLREGNO	686388	5.921
ChEMBL MOLREGNO	686389	6.131
ChEMBL MOLREGNO	686390	7.155
ChEMBL MOLREGNO	686438	5.499
ChEMBL MOLREGNO	686439	6.051
ChEMBL MOLREGNO	686440	5.479
ChEMBL MOLREGNO	686441	4.691
ChEMBL MOLREGNO	686442	6.284
ChEMBL MOLREGNO	689439	7.444
ChEMBL MOLREGNO	689440	7.699
ChEMBL MOLREGNO	689441	7.377
ChEMBL MOLREGNO	690426	5.699
ChEMBL MOLREGNO	690427	5.602
ChEMBL MOLREGNO	690428	5.301
ChEMBL MOLREGNO	690430	5.523
ChEMBL MOLREGNO	690431	5.046
ChEMBL MOLREGNO	690432	5.222
ChEMBL MOLREGNO	690433	5.398
ChEMBL MOLREGNO	690434	4.886
ChEMBL	690435	5.000

MOLREGNO		
ChEMBL MOLREGNO	690436	4.921
ChEMBL MOLREGNO	690504	4.892
ChEMBL MOLREGNO	690505	4.758
ChEMBL MOLREGNO	690506	4.603
ChEMBL MOLREGNO	690536	4.886
ChEMBL MOLREGNO	690537	5.000
ChEMBL MOLREGNO	690575	4.715
ChEMBL MOLREGNO	690576	4.504
ChEMBL MOLREGNO	690577	4.812
ChEMBL MOLREGNO	691562	8.569
ChEMBL MOLREGNO	699397	4.469
ChEMBL MOLREGNO	705972	8.307
ChEMBL MOLREGNO	706581	6.111
ChEMBL MOLREGNO	706702	6.509
ChEMBL MOLREGNO	706703	6.347
ChEMBL MOLREGNO	706827	6.648
ChEMBL MOLREGNO	707055	7.229
ChEMBL MOLREGNO	707056	7.347
ChEMBL MOLREGNO	707174	6.770
ChEMBL MOLREGNO	707515	7.022
ChEMBL MOLREGNO	707629	7.155
ChEMBL MOLREGNO	707739	7.456
ChEMBL MOLREGNO	707740	7.328
ChEMBL MOLREGNO	708711	4.824

ChEMBL MOLREGNO	709129	4.056
ChEMBL MOLREGNO	709130	4.721
ChEMBL MOLREGNO	709237	4.959
ChEMBL MOLREGNO	709238	4.854
ChEMBL MOLREGNO	709345	4.469
ChEMBL MOLREGNO	709347	5.261
ChEMBL MOLREGNO	709453	4.824
ChEMBL MOLREGNO	709454	4.523
ChEMBL MOLREGNO	709548	4.398
ChEMBL MOLREGNO	709549	4.658
ChEMBL MOLREGNO	709558	5.620
ChEMBL MOLREGNO	709648	4.959
ChEMBL MOLREGNO	709658	4.745
ChEMBL MOLREGNO	709659	5.041
ChEMBL MOLREGNO	709744	4.620
ChEMBL MOLREGNO	709753	5.108
ChEMBL MOLREGNO	709754	5.092
ChEMBL MOLREGNO	709846	4.796
ChEMBL MOLREGNO	709855	4.620
ChEMBL MOLREGNO	709945	4.721
ChEMBL MOLREGNO	709946	4.222
ChEMBL MOLREGNO	710051	4.523
ChEMBL MOLREGNO	710052	4.456
ChEMBL MOLREGNO	710155	4.921
ChEMBL	710156	4.602

MOLREGNO		
ChEMBL MOLREGNO	710255	4.161
ChEMBL MOLREGNO	710256	4.387
ChEMBL MOLREGNO	710465	4.569
ChEMBL MOLREGNO	710677	4.000
ChEMBL MOLREGNO	710678	4.244
ChEMBL MOLREGNO	715482	5.847
ChEMBL MOLREGNO	715603	6.272
ChEMBL MOLREGNO	715604	6.411
ChEMBL MOLREGNO	715717	6.292
ChEMBL MOLREGNO	715836	6.839
ChEMBL MOLREGNO	715837	6.418
ChEMBL MOLREGNO	715949	6.504
ChEMBL MOLREGNO	715950	6.616
ChEMBL MOLREGNO	716059	6.511
ChEMBL MOLREGNO	716060	6.298
ChEMBL MOLREGNO	716165	6.496
ChEMBL MOLREGNO	716166	6.600
ChEMBL MOLREGNO	716273	6.253
ChEMBL MOLREGNO	716274	6.469
ChEMBL MOLREGNO	716392	6.258
ChEMBL MOLREGNO	880841	4.301
ChEMBL MOLREGNO	1070648	5.633
ChEMBL MOLREGNO	1072032	4.036
ChEMBL MOLREGNO	1072050	4.260

ChEMBL MOLREGNO	1138754	4.432
ChEMBL MOLREGNO	1138755	4.602
ChEMBL MOLREGNO	1138756	4.678
ChEMBL MOLREGNO	1138757	4.387
ChEMBL MOLREGNO	1138758	5.387
ChEMBL MOLREGNO	1138759	5.155
ChEMBL MOLREGNO	1142729	7.301
ChEMBL MOLREGNO	1142731	7.523
ChEMBL MOLREGNO	1142732	7.523
ChEMBL MOLREGNO	1144348	8.538
ChEMBL MOLREGNO	1144349	7.495
ChEMBL MOLREGNO	1144350	7.745
ChEMBL MOLREGNO	1144351	7.301
ChEMBL MOLREGNO	1144352	7.770
ChEMBL MOLREGNO	1144353	7.092
ChEMBL MOLREGNO	1144354	7.824
ChEMBL MOLREGNO	1144355	7.824
ChEMBL MOLREGNO	1144356	7.585
ChEMBL MOLREGNO	1144357	7.456
ChEMBL MOLREGNO	1144358	7.959
ChEMBL MOLREGNO	1144359	7.921
ChEMBL MOLREGNO	1144360	7.959
ChEMBL MOLREGNO	1144438	7.854
ChEMBL MOLREGNO	1144439	7.770
ChEMBL	1144440	7.854

MOLREGNO		
ChEMBL MOLREGNO	1144441	7.796
ChEMBL MOLREGNO	1144442	7.886
ChEMBL MOLREGNO	1144443	7.886
ChEMBL MOLREGNO	1144444	7.854
ChEMBL MOLREGNO	1144445	8.056
ChEMBL MOLREGNO	1144446	7.854
ChEMBL MOLREGNO	1144447	7.886
ChEMBL MOLREGNO	1144448	7.367
ChEMBL MOLREGNO	1144449	7.180
ChEMBL MOLREGNO	1144450	7.886
ChEMBL MOLREGNO	1144451	7.658
ChEMBL MOLREGNO	1144452	7.444
ChEMBL MOLREGNO	1145087	4.963
ChEMBL MOLREGNO	1145088	5.481
ChEMBL MOLREGNO	1145091	4.983
ChEMBL MOLREGNO	1145092	5.221
ChEMBL MOLREGNO	1145093	5.036
ChEMBL MOLREGNO	1145095	4.043
ChEMBL MOLREGNO	1168156	5.699
ChEMBL MOLREGNO	1168157	4.377
ChEMBL MOLREGNO	1168414	4.000
ChEMBL MOLREGNO	1168415	4.042
ChEMBL MOLREGNO	1168418	4.585
ChEMBL MOLREGNO	1168419	4.638

ChEMBL MOLREGNO	1168527	4.620
ChEMBL MOLREGNO	1168528	4.000
ChEMBL MOLREGNO	1168529	4.237
ChEMBL MOLREGNO	1168530	4.921
ChEMBL MOLREGNO	1168531	4.824
ChEMBL MOLREGNO	1168532	4.678
ChEMBL MOLREGNO	1168535	4.745
ChEMBL MOLREGNO	1168536	4.252
ChEMBL MOLREGNO	1168537	4.000
ChEMBL MOLREGNO	1168538	5.301
ChEMBL MOLREGNO	1168539	4.357
ChEMBL MOLREGNO	1168540	5.097
ChEMBL MOLREGNO	1168541	4.886
ChEMBL MOLREGNO	1168542	4.959
ChEMBL MOLREGNO	1168543	4.721
ChEMBL MOLREGNO	1168544	4.357
ChEMBL MOLREGNO	1168545	4.469
ChEMBL MOLREGNO	1168546	4.854
ChEMBL MOLREGNO	1168547	4.658
ChEMBL MOLREGNO	1168548	4.699
ChEMBL MOLREGNO	1168549	4.409
ChEMBL MOLREGNO	1249566	4.000
ChEMBL MOLREGNO	1249568	4.398
ChEMBL MOLREGNO	1249569	4.347
ChEMBL	1249570	4.357

MOLREGNO		
ChEMBL MOLREGNO	1261987	7.495
ChEMBL MOLREGNO	1261988	8.155
ChEMBL MOLREGNO	1261989	8.000
ChEMBL MOLREGNO	1261990	8.222
ChEMBL MOLREGNO	1261991	8.097
ChEMBL MOLREGNO	1261992	7.699
ChEMBL MOLREGNO	1261993	7.770
ChEMBL MOLREGNO	1261994	7.745
ChEMBL MOLREGNO	1261995	7.469
ChEMBL MOLREGNO	1261996	7.553
ChEMBL MOLREGNO	1261997	7.553
ChEMBL MOLREGNO	1261998	7.495
ChEMBL MOLREGNO	1261999	7.854
ChEMBL MOLREGNO	1262000	8.398
ChEMBL MOLREGNO	1262001	7.721
ChEMBL MOLREGNO	1262002	8.097
ChEMBL MOLREGNO	1265343	7.886
ChEMBL MOLREGNO	1265344	9.000
ChEMBL MOLREGNO	1265345	8.222
ChEMBL MOLREGNO	1265346	7.886
ChEMBL MOLREGNO	1265347	8.222
ChEMBL MOLREGNO	1265348	7.456
ChEMBL MOLREGNO	1269130	4.398
ChEMBL MOLREGNO	1269131	5.585

ChEMBL MOLREGNO	1269133	4.495
ChEMBL MOLREGNO	1269134	6.108
ChEMBL MOLREGNO	1299633	4.745
ChEMBL MOLREGNO	1348813	7.155
ChEMBL MOLREGNO	1348980	8.260
ChEMBL MOLREGNO	1349582	8.222
ChEMBL MOLREGNO	1368967	4.509
ChEMBL MOLREGNO	1431161	6.886
ChEMBL MOLREGNO	1431162	6.523
ChEMBL MOLREGNO	1431163	6.420
ChEMBL MOLREGNO	1431164	5.886
ChEMBL MOLREGNO	1431165	6.268
ChEMBL MOLREGNO	1431166	7.745
ChEMBL MOLREGNO	1431167	8.000
ChEMBL MOLREGNO	1441402	6.155
ChEMBL MOLREGNO	1441403	6.155
ChEMBL MOLREGNO	1441404	6.699
ChEMBL MOLREGNO	1441405	6.000
ChEMBL MOLREGNO	1441406	6.523
ChEMBL MOLREGNO	1441407	6.611
ChEMBL MOLREGNO	1441410	4.161
ChEMBL MOLREGNO	1441416	6.301
ChEMBL MOLREGNO	1441417	4.886
ChEMBL MOLREGNO	1441418	4.509
ChEMBL	1445859	6.585

MOLREGNO		
ChEMBL MOLREGNO	1445860	6.602
ChEMBL MOLREGNO	1445861	4.939
ChEMBL MOLREGNO	1445862	5.167
ChEMBL MOLREGNO	1445863	5.268
ChEMBL MOLREGNO	1445864	6.060
ChEMBL MOLREGNO	1445865	5.824
ChEMBL MOLREGNO	1446396	6.398
ChEMBL MOLREGNO	1446397	6.921
ChEMBL MOLREGNO	1446398	7.268
ChEMBL MOLREGNO	1446399	6.959
ChEMBL MOLREGNO	1446400	6.620
ChEMBL MOLREGNO	1446401	6.495
ChEMBL MOLREGNO	1446402	6.509
ChEMBL MOLREGNO	1446403	6.432
ChEMBL MOLREGNO	1446404	6.398
ChEMBL MOLREGNO	1446405	6.721
ChEMBL MOLREGNO	1446406	7.328
ChEMBL MOLREGNO	1446407	7.097
ChEMBL MOLREGNO	1446408	7.237
ChEMBL MOLREGNO	1446409	5.553
ChEMBL MOLREGNO	1446410	5.638
ChEMBL MOLREGNO	1446411	6.721
ChEMBL MOLREGNO	1446412	6.387
ChEMBL MOLREGNO	1448156	5.523

ChEMBL MOLREGNO	1472109	7.420
ChEMBL MOLREGNO	1472110	5.854
ChEMBL MOLREGNO	1472119	5.310
ChEMBL MOLREGNO	1472120	7.824
ChEMBL MOLREGNO	1472121	6.602
ChEMBL MOLREGNO	1472122	7.721
ChEMBL MOLREGNO	1472123	4.796
ChEMBL MOLREGNO	1472124	6.854
ChEMBL MOLREGNO	1500729	5.000
ChEMBL MOLREGNO	1500730	6.046
ChEMBL MOLREGNO	1500732	4.377
ChEMBL MOLREGNO	1500733	6.854
ChEMBL MOLREGNO	1500734	4.850
ChEMBL MOLREGNO	1500735	5.060
ChEMBL MOLREGNO	1510908	4.886
ChEMBL MOLREGNO	1510914	4.824
ChEMBL MOLREGNO	1510919	4.000
ChEMBL MOLREGNO	1510922	4.125
ChEMBL MOLREGNO	1510923	4.097
ChEMBL MOLREGNO	1510928	4.284
ChEMBL MOLREGNO	1510943	4.000
ChEMBL MOLREGNO	1514909	7.796
ChEMBL MOLREGNO	1514910	7.886
ChEMBL MOLREGNO	1514911	8.301
ChEMBL	1514912	8.301

MOLREGNO		
ChEMBL MOLREGNO	1514913	8.301
ChEMBL MOLREGNO	1515194	8.398
ChEMBL MOLREGNO	1515195	8.046
ChEMBL MOLREGNO	1515196	8.301
ChEMBL MOLREGNO	1515197	8.349
ChEMBL MOLREGNO	1515199	8.523
ChEMBL MOLREGNO	1515200	8.372
ChEMBL MOLREGNO	1515202	8.398
ChEMBL MOLREGNO	1515203	8.301
ChEMBL MOLREGNO	1515204	8.301
ChEMBL MOLREGNO	1515205	8.247
ChEMBL MOLREGNO	1515207	8.222
ChEMBL MOLREGNO	1515208	8.155
ChEMBL MOLREGNO	1515209	8.699
ChEMBL MOLREGNO	1515210	8.460
ChEMBL MOLREGNO	1515212	8.222
ChEMBL MOLREGNO	1515213	8.301
ChEMBL MOLREGNO	1515214	8.046
ChEMBL MOLREGNO	1515215	8.046
ChEMBL MOLREGNO	1515216	8.301
ChEMBL MOLREGNO	1515217	7.959
ChEMBL MOLREGNO	1515218	7.854
ChEMBL MOLREGNO	1515219	7.638
ChEMBL MOLREGNO	1515220	8.398

ChEMBL MOLREGNO	1515221	8.155
ChEMBL MOLREGNO	1515222	8.097
ChEMBL MOLREGNO	1515223	7.959
ChEMBL MOLREGNO	1569497	7.319
ChEMBL MOLREGNO	1570714	8.699
ChEMBL MOLREGNO	1570716	8.301
ChEMBL MOLREGNO	1570837	8.523
ChEMBL MOLREGNO	1571673	6.699
ChEMBL MOLREGNO	1571674	6.900
ChEMBL MOLREGNO	1571675	6.529
ChEMBL MOLREGNO	1571676	5.252
ChEMBL MOLREGNO	1571677	6.137
ChEMBL MOLREGNO	1571678	6.558
ChEMBL MOLREGNO	1571679	6.959
ChEMBL MOLREGNO	1571680	5.951
ChEMBL MOLREGNO	1571681	7.523
ChEMBL MOLREGNO	1571682	7.076
ChEMBL MOLREGNO	1571683	5.987
ChEMBL MOLREGNO	1571684	6.595
ChEMBL MOLREGNO	1571685	6.243
ChEMBL MOLREGNO	1571686	6.513
ChEMBL MOLREGNO	1571687	6.588
ChEMBL MOLREGNO	1571688	7.004
ChEMBL MOLREGNO	1571690	4.102
ChEMBL	1604805	5.921

MOLREGNO		
ChEMBL MOLREGNO	1604806	5.310
ChEMBL MOLREGNO	1604807	6.770
ChEMBL MOLREGNO	1604808	6.013
ChEMBL MOLREGNO	1604809	6.509
ChEMBL MOLREGNO	1604810	5.222
ChEMBL MOLREGNO	1604811	6.125
ChEMBL MOLREGNO	1604812	5.770
ChEMBL MOLREGNO	1604813	6.276
ChEMBL MOLREGNO	1604814	6.509
ChEMBL MOLREGNO	1604815	5.796
ChEMBL MOLREGNO	1605095	7.229
ChEMBL MOLREGNO	1605096	7.377
ChEMBL MOLREGNO	1605097	7.284
ChEMBL MOLREGNO	1605098	6.824
ChEMBL MOLREGNO	1605099	5.796
ChEMBL MOLREGNO	1605100	6.000
ChEMBL MOLREGNO	1605101	6.347
ChEMBL MOLREGNO	1605102	5.398
ChEMBL MOLREGNO	1605103	6.222
ChEMBL MOLREGNO	1605104	6.310
ChEMBL MOLREGNO	1605106	5.770
ChEMBL MOLREGNO	1605107	5.097
ChEMBL MOLREGNO	1605108	6.770
ChEMBL MOLREGNO	1605109	5.886

ChEMBL MOLREGNO	1605110	6.009
ChEMBL MOLREGNO	1605111	6.036
ChEMBL MOLREGNO	1605112	6.000
ChEMBL MOLREGNO	1605113	4.959
ChEMBL MOLREGNO	1605114	5.222
ChEMBL MOLREGNO	1605115	5.046
ChEMBL MOLREGNO	1605266	4.886
ChEMBL MOLREGNO	1605268	4.638
ChEMBL MOLREGNO	1605269	4.921
ChEMBL MOLREGNO	1605270	4.721
ChEMBL MOLREGNO	1605271	6.276
ChEMBL MOLREGNO	1605272	4.495
ChEMBL MOLREGNO	1605273	5.097
ChEMBL MOLREGNO	1619292	4.799
ChEMBL MOLREGNO	1619293	5.611
ChEMBL MOLREGNO	1619294	4.912
ChEMBL MOLREGNO	1619295	5.446
ChEMBL MOLREGNO	1622578	4.706
ChEMBL MOLREGNO	1622580	7.569
ChEMBL MOLREGNO	1622581	7.398
ChEMBL MOLREGNO	1622582	7.260
ChEMBL MOLREGNO	1622583	5.108
ChEMBL MOLREGNO	1622584	5.092
ChEMBL MOLREGNO	1622585	5.149
ChEMBL	1622586	6.276

MOLREGNO		
ChEMBL MOLREGNO	1622587	6.108
ChEMBL MOLREGNO	1622588	4.745
ChEMBL MOLREGNO	1622589	7.854
ChEMBL MOLREGNO	1622590	7.770
ChEMBL MOLREGNO	1622591	7.387
ChEMBL MOLREGNO	1736672	5.620
ChEMBL MOLREGNO	1736680	7.097
ChEMBL MOLREGNO	1778997	8.119
ChEMBL MOLREGNO	1778998	8.032
ChEMBL MOLREGNO	1778999	8.032
ChEMBL MOLREGNO	1779000	8.086
ChEMBL MOLREGNO	1779001	8.357
ChEMBL MOLREGNO	1779002	7.886
ChEMBL MOLREGNO	1779172	8.036
ChEMBL MOLREGNO	1779173	8.108
ChEMBL MOLREGNO	1779174	8.357
ChEMBL MOLREGNO	1779175	8.161
ChEMBL MOLREGNO	1779176	8.097
ChEMBL MOLREGNO	1779177	8.149
ChEMBL MOLREGNO	1779178	8.167
ChEMBL MOLREGNO	1779179	7.658
ChEMBL MOLREGNO	1779180	8.244
ChEMBL MOLREGNO	1779181	8.523
ChEMBL MOLREGNO	1779182	8.244

ChEMBL MOLREGNO	1779183	8.398
ChEMBL MOLREGNO	1779184	8.107
ChEMBL MOLREGNO	1779186	8.187
ChEMBL MOLREGNO	1779187	7.638
ChEMBL MOLREGNO	1779188	8.212
ChEMBL MOLREGNO	1779190	8.066
ChEMBL MOLREGNO	1779191	8.114
ChEMBL MOLREGNO	1779192	8.046
ChEMBL MOLREGNO	1779193	8.137
ChEMBL MOLREGNO	1779194	8.071
ChEMBL MOLREGNO	1779509	8.208
ChEMBL MOLREGNO	1779510	7.959
ChEMBL MOLREGNO	1779511	7.347
ChEMBL MOLREGNO	1779512	7.377
ChEMBL MOLREGNO	1779513	8.328
ChEMBL MOLREGNO	1779514	8.119
ChEMBL MOLREGNO	1779515	7.553
ChEMBL MOLREGNO	1790759	6.222
ChEMBL MOLREGNO	1790760	5.898
ChEMBL MOLREGNO	1790761	6.151
ChEMBL MOLREGNO	1790762	6.000
ChEMBL MOLREGNO	1790763	6.301
ChEMBL MOLREGNO	1790764	5.699
ChEMBL MOLREGNO	1790765	5.398
ChEMBL	1790766	5.854

MOLREGNO		
ChEMBL MOLREGNO	1790767	5.222
ChEMBL MOLREGNO	1790768	5.523
ChEMBL MOLREGNO	1790769	5.155
ChEMBL MOLREGNO	1790770	5.854
ChEMBL MOLREGNO	1790771	5.398
ChEMBL MOLREGNO	1790772	5.097
ChEMBL MOLREGNO	1790773	6.000
ChEMBL MOLREGNO	1790774	5.000
ChEMBL MOLREGNO	1790776	5.699
ChEMBL MOLREGNO	1790778	6.000
ChEMBL MOLREGNO	1824284	7.678
ChEMBL MOLREGNO	1824285	7.721
ChEMBL MOLREGNO	1824286	8.398
ChEMBL MOLREGNO	1824287	8.222
ChEMBL MOLREGNO	1824288	8.523
ChEMBL MOLREGNO	1824289	8.301
ChEMBL MOLREGNO	1824290	8.301
ChEMBL MOLREGNO	1824291	8.301
ChEMBL MOLREGNO	1824292	8.000
ChEMBL MOLREGNO	1824293	8.046
ChEMBL MOLREGNO	1824294	8.000
ChEMBL MOLREGNO	1824295	8.046
ChEMBL MOLREGNO	1824296	8.398
ChEMBL MOLREGNO	1824297	7.921

ChEMBL MOLREGNO	1824298	8.000
ChEMBL MOLREGNO	1824299	8.222
ChEMBL MOLREGNO	1824300	7.745
ChEMBL MOLREGNO	1824301	7.796
ChEMBL MOLREGNO	1824302	8.000
ChEMBL MOLREGNO	1824303	8.222
ChEMBL MOLREGNO	1824304	8.523
ChEMBL MOLREGNO	1824305	8.097
ChEMBL MOLREGNO	1824306	7.921
ChEMBL MOLREGNO	1824307	7.959
ChEMBL MOLREGNO	1824308	8.222
ChEMBL MOLREGNO	1824309	8.097
ChEMBL MOLREGNO	1824310	7.886
ChEMBL MOLREGNO	1824311	8.046
ChEMBL MOLREGNO	1824312	7.770
ChEMBL MOLREGNO	1824313	8.222
ChEMBL MOLREGNO	1829568	6.046
ChEMBL MOLREGNO	1829569	5.620
ChEMBL MOLREGNO	1829570	4.724
ChEMBL MOLREGNO	1829571	5.456
ChEMBL MOLREGNO	1829572	4.650
ChEMBL MOLREGNO	1829573	4.730
ChEMBL MOLREGNO	1829574	5.602
ChEMBL MOLREGNO	1829575	6.155
ChEMBL	1839026	6.377

MOLREGNO		
ChEMBL MOLREGNO	1839027	5.796
ChEMBL MOLREGNO	1839029	5.367
ChEMBL MOLREGNO	1839030	5.921
ChEMBL MOLREGNO	1839032	5.602
ChEMBL MOLREGNO	1839033	4.046
ChEMBL MOLREGNO	1839035	6.292
ChEMBL MOLREGNO	1839036	6.102
ChEMBL MOLREGNO	1839037	7.658
ChEMBL MOLREGNO	1839039	7.620
ChEMBL MOLREGNO	1839040	7.367
ChEMBL MOLREGNO	1839041	7.201
ChEMBL MOLREGNO	1839042	6.229
ChEMBL MOLREGNO	1839044	7.180
ChEMBL MOLREGNO	1839045	4.585
ChEMBL MOLREGNO	1839046	6.137
ChEMBL MOLREGNO	1839047	7.721
ChEMBL MOLREGNO	1839048	6.959
ChEMBL MOLREGNO	1839049	4.009
ChEMBL MOLREGNO	1839050	6.222
ChEMBL MOLREGNO	1948101	6.155
ChEMBL MOLREGNO	1948102	6.553
ChEMBL MOLREGNO	1948104	5.854
ChEMBL MOLREGNO	1948107	5.658
ChEMBL MOLREGNO	1948108	5.432

ChEMBL MOLREGNO	1948109	5.260
ChEMBL MOLREGNO	1948110	4.959
ChEMBL MOLREGNO	1948112	4.495
ChEMBL MOLREGNO	1948114	5.886
ChEMBL MOLREGNO	1948115	5.967
ChEMBL MOLREGNO	1948116	5.509
ChEMBL MOLREGNO	1948117	4.601
ChEMBL MOLREGNO	1948119	7.469
ChEMBL MOLREGNO	1948120	7.796
ChEMBL MOLREGNO	1948121	6.268
ChEMBL MOLREGNO	1948122	6.347
ChEMBL MOLREGNO	1948123	6.292
ChEMBL MOLREGNO	1948124	6.194
ChEMBL MOLREGNO	1948125	8.000
ChEMBL MOLREGNO	1948126	5.495
ChEMBL MOLREGNO	1948127	6.959
ChEMBL MOLREGNO	1948128	6.658
ChEMBL MOLREGNO	1948129	5.638
ChEMBL MOLREGNO	1948130	6.569
ChEMBL MOLREGNO	1948131	5.155
ChEMBL MOLREGNO	1948132	7.301
ChEMBL MOLREGNO	1948133	7.745
ChEMBL MOLREGNO	1948134	6.721
ChEMBL MOLREGNO	1948135	7.018
ChEMBL	1948136	6.886

MOLREGNO		
ChEMBL MOLREGNO	1948139	4.495
ChEMBL MOLREGNO	1948140	4.854
ChEMBL MOLREGNO	1948141	5.456
ChEMBL MOLREGNO	1948144	4.796
ChEMBL MOLREGNO	1948145	6.398
ChEMBL MOLREGNO	2038879	4.208
ChEMBL MOLREGNO	2051744	5.620
ChEMBL MOLREGNO	2051745	5.886
ChEMBL MOLREGNO	2051746	6.721
ChEMBL MOLREGNO	2051747	6.194
ChEMBL MOLREGNO	2051748	5.959
ChEMBL MOLREGNO	2051749	5.252
ChEMBL MOLREGNO	2051750	5.638
ChEMBL MOLREGNO	2051751	5.444
ChEMBL MOLREGNO	2051752	5.481
ChEMBL MOLREGNO	2051753	5.699
ChEMBL MOLREGNO	2051754	5.620
ChEMBL MOLREGNO	2051755	5.721
ChEMBL MOLREGNO	2051756	5.886
ChEMBL MOLREGNO	2051757	6.071
ChEMBL MOLREGNO	2051758	5.652
ChEMBL MOLREGNO	2051759	6.036
ChEMBL MOLREGNO	2051760	5.644
ChEMBL MOLREGNO	2051761	5.215

ChEMBL MOLREGNO	2051762	5.553
ChEMBL MOLREGNO	2051763	5.921
ChEMBL MOLREGNO	2053275	4.921
ChEMBL MOLREGNO	2053279	6.337
ChEMBL MOLREGNO	2053280	6.222
ChEMBL MOLREGNO	2053281	5.538
ChEMBL MOLREGNO	2053282	5.180
ChEMBL MOLREGNO	2053283	4.821
ChEMBL MOLREGNO	2053284	4.747
ChEMBL MOLREGNO	2053285	5.119
ChEMBL MOLREGNO	2053286	4.656
ChEMBL MOLREGNO	2053287	6.086
ChEMBL MOLREGNO	2053288	4.975
ChEMBL MOLREGNO	2053289	4.928
ChEMBL MOLREGNO	2053290	5.102
ChEMBL MOLREGNO	2053291	4.654
ChEMBL MOLREGNO	2053292	4.599
ChEMBL MOLREGNO	2053293	5.284
ChEMBL MOLREGNO	2053296	6.155
ChEMBL MOLREGNO	2053297	5.509
ChEMBL MOLREGNO	2085861	7.331
ChEMBL MOLREGNO	2085862	7.520
ChEMBL MOLREGNO	2085863	6.749
ChEMBL MOLREGNO	2085865	6.858
ChEMBL	2085867	6.890

MOLREGNO		
ChEMBL MOLREGNO	2085869	8.023
ChEMBL MOLREGNO	2085871	8.960
ChEMBL MOLREGNO	2085887	5.701
ChEMBL MOLREGNO	2085888	5.297
ChEMBL MOLREGNO	2085889	6.319
ChEMBL MOLREGNO	2085947	5.060
ChEMBL MOLREGNO	2085949	5.351
ChEMBL MOLREGNO	2085950	5.875
ChEMBL MOLREGNO	2085955	5.398
ChEMBL MOLREGNO	2085956	5.457
ChEMBL MOLREGNO	2085958	5.041
Integrity Entry Number	1000628	4.921
Integrity Entry Number	1015129	5.928
Integrity Entry Number	1034472	7.328
Integrity Entry Number	1034473	7.770
Integrity Entry Number	1034475	7.481
Integrity Entry Number	1034477	7.638
Integrity Entry Number	1034480	7.509
Integrity Entry Number	214729	4.790
Integrity Entry Number	285754	4.602
Integrity Entry Number	289258	4.594
Integrity Entry Number	294773	5.222
Integrity Entry Number	294774	4.940
Integrity Entry Number	294775	5.002

Integrity Entry Number	322551	6.092
Integrity Entry Number	325704	8.000
Integrity Entry Number	327893	5.626
Integrity Entry Number	327895	6.704
Integrity Entry Number	327896	6.192
Integrity Entry Number	327897	5.182
Integrity Entry Number	327898	4.620
Integrity Entry Number	327899	5.784
Integrity Entry Number	339599	7.699
Integrity Entry Number	339604	8.000
Integrity Entry Number	339907	8.000
Integrity Entry Number	339909	7.301
Integrity Entry Number	339911	6.699
Integrity Entry Number	361036	8.000
Integrity Entry Number	361040	8.000
Integrity Entry Number	370085	7.046
Integrity Entry Number	371224	7.076
Integrity Entry Number	383019	7.000
Integrity Entry Number	383089	6.000
Integrity Entry Number	394308	7.456
Integrity Entry Number	395361	5.921
Integrity Entry Number	395365	5.000
Integrity Entry Number	411791	7.959
Integrity Entry Number	411792	8.155
Integrity Entry	411793	7.699

Number		
Integrity Entry Number	411795	7.398
Integrity Entry Number	411798	7.870
Integrity Entry Number	411803	8.516
Integrity Entry Number	411804	8.352
Integrity Entry Number	411806	8.284
Integrity Entry Number	411807	8.131
Integrity Entry Number	414242	8.495
Integrity Entry Number	414244	8.456
Integrity Entry Number	414246	8.444
Integrity Entry Number	414248	8.444
Integrity Entry Number	414249	8.481
Integrity Entry Number	414251	8.444
Integrity Entry Number	425528	8.000
Integrity Entry Number	425909	5.569
Integrity Entry Number	431305	8.155
Integrity Entry Number	433924	7.169
Integrity Entry Number	437359	7.523
Integrity Entry Number	449510	5.569
Integrity Entry Number	450173	7.252
Integrity Entry Number	450174	6.886
Integrity Entry Number	450176	7.393
Integrity Entry Number	450718	4.695
Integrity Entry Number	454762	4.745
Integrity Entry Number	458892	8.155

Integrity Entry Number	466889	7.523
Integrity Entry Number	466890	7.432
Integrity Entry Number	467907	8.000
Integrity Entry Number	469685	4.910
Integrity Entry Number	469686	5.000
Integrity Entry Number	472351	6.222
Integrity Entry Number	476526	7.770
Integrity Entry Number	476527	8.188
Integrity Entry Number	478208	5.398
Integrity Entry Number	478209	5.398
Integrity Entry Number	478234	7.155
Integrity Entry Number	632866	7.699
Integrity Entry Number	632869	7.699
Integrity Entry Number	633911	7.372
Integrity Entry Number	637126	7.149
Integrity Entry Number	641330	6.699
Integrity Entry Number	641332	6.222
Integrity Entry Number	646822	6.796
Integrity Entry Number	646823	5.427
Integrity Entry Number	646991	6.523
Integrity Entry Number	648213	7.699
Integrity Entry Number	654677	6.432
Integrity Entry Number	655066	7.097
Integrity Entry Number	655067	8.222
Integrity Entry	655784	7.444

Number		
Integrity Entry Number	655785	7.721
Integrity Entry Number	659422	5.523
Integrity Entry Number	663044	7.745
Integrity Entry Number	663045	8.222
Integrity Entry Number	663046	8.523
Integrity Entry Number	663109	7.921
Integrity Entry Number	663113	7.959
Integrity Entry Number	663114	8.699
Integrity Entry Number	665459	5.699
Integrity Entry Number	665656	6.046
Integrity Entry Number	666736	8.398
Integrity Entry Number	668607	4.872
Integrity Entry Number	670027	5.155
Integrity Entry Number	670453	5.602
Integrity Entry Number	670454	6.155
Integrity Entry Number	670455	5.824
Integrity Entry Number	670456	6.155
Integrity Entry Number	670620	7.523
Integrity Entry Number	670892	8.301
Integrity Entry Number	670893	8.699
Integrity Entry Number	670895	8.222
Integrity Entry Number	680574	6.883
Integrity Entry Number	680575	6.991
Integrity Entry Number	680579	6.863

Integrity Entry Number	682554	6.484
Integrity Entry Number	684678	8.222
Integrity Entry Number	684685	7.523
Integrity Entry Number	685458	9.000
Integrity Entry Number	685459	9.000
Integrity Entry Number	685460	9.000
Integrity Entry Number	685461	9.000
Integrity Entry Number	685546	9.000
Integrity Entry Number	685547	9.000
Integrity Entry Number	685548	8.164
Integrity Entry Number	685549	9.000
Integrity Entry Number	685550	9.000
Integrity Entry Number	685551	9.000
Integrity Entry Number	685553	8.361
Integrity Entry Number	688869	7.222
Integrity Entry Number	688870	7.523
Integrity Entry Number	688871	7.523
Integrity Entry Number	688872	6.991
Integrity Entry Number	692977	7.426
Integrity Entry Number	692994	7.262
Integrity Entry Number	703447	6.770
Integrity Entry Number	703823	7.204
Integrity Entry Number	703824	6.745
Integrity Entry Number	703826	6.827
Integrity Entry	703828	7.159

Number		
Integrity Entry Number	705233	7.886
Integrity Entry Number	708266	5.222
Integrity Entry Number	708324	9.000
Integrity Entry Number	708328	8.361
Integrity Entry Number	708407	7.620
Integrity Entry Number	708411	7.569
Integrity Entry Number	708415	7.495
Integrity Entry Number	708418	7.699
Integrity Entry Number	708419	7.721
Integrity Entry Number	710926	8.222
Integrity Entry Number	710928	8.398
Integrity Entry Number	710931	8.523
Integrity Entry Number	714959	8.301
Integrity Entry Number	714960	8.460
Integrity Entry Number	714961	8.398
Integrity Entry Number	715288	8.046
Integrity Entry Number	723963	7.000
Integrity Entry Number	723964	6.921
Integrity Entry Number	723965	7.000
Integrity Entry Number	723967	5.495
Integrity Entry Number	729433	8.469
Integrity Entry Number	729434	8.155
Integrity Entry Number	729436	8.155
Integrity Entry Number	729441	8.398

Integrity Entry Number	730493	5.398
Integrity Entry Number	733999	4.406
Integrity Entry Number	734000	4.545
Integrity Entry Number	734001	4.599
Integrity Entry Number	734003	4.299
Integrity Entry Number	734004	4.351
Integrity Entry Number	735694	5.316
Integrity Entry Number	735971	5.456
Integrity Entry Number	735972	4.678
Integrity Entry Number	735973	5.638
Integrity Entry Number	738505	5.432
Integrity Entry Number	738946	5.387
Integrity Entry Number	739933	7.301
Integrity Entry Number	742253	5.481
Integrity Entry Number	742634	7.824
Integrity Entry Number	751185	7.527
Integrity Entry Number	751189	7.547
Integrity Entry Number	751195	7.569
Integrity Entry Number	755496	4.398
Integrity Entry Number	757703	6.842
Integrity Entry Number	761682	8.097
Integrity Entry Number	761684	7.854
Integrity Entry Number	761691	7.770
Integrity Entry Number	761693	7.886
Integrity Entry	764606	6.260

Number		
Integrity Entry Number	764608	6.018
Integrity Entry Number	766302	5.585
Integrity Entry Number	766304	6.108
Integrity Entry Number	769926	8.222
Integrity Entry Number	773580	7.328
Integrity Entry Number	773581	7.268
Integrity Entry Number	781187	6.710
Integrity Entry Number	781188	7.128
Integrity Entry Number	781189	6.849
Integrity Entry Number	788096	7.174
Integrity Entry Number	788098	7.260
Integrity Entry Number	792003	6.155
Integrity Entry Number	792006	6.155
Integrity Entry Number	798912	6.398
Integrity Entry Number	806122	6.155
Integrity Entry Number	806125	6.046
Integrity Entry Number	806127	5.620
Integrity Entry Number	806128	5.456
Integrity Entry Number	806131	5.602
Integrity Entry Number	811125	8.409
Integrity Entry Number	813280	6.194
Integrity Entry Number	813282	4.719
Integrity Entry Number	824473	7.479
Integrity Entry Number	824475	6.024

Integrity Entry Number	830412	5.523
Integrity Entry Number	830415	5.699
Integrity Entry Number	830416	5.523
Integrity Entry Number	830493	5.886
Integrity Entry Number	834896	7.190
Integrity Entry Number	843771	5.611
Integrity Entry Number	843971	7.323
Integrity Entry Number	843972	7.323
Integrity Entry Number	849232	6.585
Integrity Entry Number	849234	5.810
Integrity Entry Number	849235	5.600
Integrity Entry Number	849237	5.963
Integrity Entry Number	849239	6.377
Integrity Entry Number	850066	5.620
Integrity Entry Number	850070	7.097
Integrity Entry Number	850073	7.097
Integrity Entry Number	850075	7.301
Integrity Entry Number	851565	7.699
Integrity Entry Number	852017	5.398
Integrity Entry Number	854632	8.000
Integrity Entry Number	855756	8.032
Integrity Entry Number	855757	8.398
Integrity Entry Number	855758	8.046
Integrity Entry Number	856257	7.569
Integrity Entry	856258	7.678

Number		
Integrity Entry Number	856260	7.721
Integrity Entry Number	862156	6.387
Integrity Entry Number	862157	6.745
Integrity Entry Number	865651	6.222
Integrity Entry Number	865652	6.301
Integrity Entry Number	865653	5.854
Integrity Entry Number	865655	5.398
Integrity Entry Number	874207	8.046
Integrity Entry Number	874518	7.630
Integrity Entry Number	877840	5.420
Integrity Entry Number	877842	5.444
Integrity Entry Number	881660	5.229
Integrity Entry Number	881669	6.339
Integrity Entry Number	881675	5.921
Integrity Entry Number	881676	7.658
Integrity Entry Number	881677	7.620
Integrity Entry Number	881678	7.721
Integrity Entry Number	894771	5.658
Integrity Entry Number	894773	6.602
Integrity Entry Number	894774	8.000
Integrity Entry Number	913756	7.602
Integrity Entry Number	913757	7.387
Integrity Entry Number	913758	7.097
Integrity Entry Number	913759	4.538

Integrity Entry Number	936048	8.569
Integrity Entry Number	937680	4.638
Integrity Entry Number	940955	4.793
Integrity Entry Number	944324	5.000
Integrity Entry Number	944472	5.398
Integrity Entry Number	952575	6.300
Integrity Entry Number	952577	6.300
Integrity Entry Number	952578	6.300
Integrity Entry Number	977257	8.000
Integrity Entry Number	977260	8.699
Integrity Entry Number	977264	8.509
Integrity Entry Number	989760	4.361
Integrity Entry Number	739731	4.963
Integrity Entry Number	401420	4.144
Integrity Entry Number	206579	4.699
Integrity Entry Number	343244	7.292
Integrity Entry Number	415111	8.523
Integrity Entry Number	676160	8.000
Integrity Entry Number	854294	8.125
Integrity Entry Number	688868	6.854
Integrity Entry Number	791548	6.836
Integrity Entry Number	437811	8.523
Integrity Entry Number	271175	4.602
Integrity Entry Number	338086	4.699
Integrity Entry	414415	8.222

Number		
Integrity Entry Number	855063	6.046
Integrity Entry Number	855057	5.252
Integrity Entry Number	439511	7.553
Integrity Entry Number	439749	7.409
Integrity Entry Number	424303	8.097
Integrity Entry Number	790829	6.669
Integrity Entry Number	442607	6.301
Integrity Entry Number	471611	7.398
Integrity Entry Number	155796	5.046
Integrity Entry Number	321046	4.495
Integrity Entry Number	321047	4.770
Integrity Entry Number	321048	4.056
Integrity Entry Number	320725	5.460
Integrity Entry Number	300893	5.959
Integrity Entry Number	300892	5.602
Integrity Entry Number	284453	6.548
Integrity Entry Number	283999	6.812
Integrity Entry Number	320387	8.004
Integrity Entry Number	349976	8.000
Integrity Entry Number	270281	7.284
Integrity Entry Number	738509	5.161
Integrity Entry Number	781186	7.128
Integrity Entry Number	447021	8.589
Integrity Entry Number	855065	5.256

Integrity Entry Number	1026656	7.000
Integrity Entry Number	1023319	7.097
Integrity Entry Number	684834	7.393
Integrity Entry Number	666183	6.149
Integrity Entry Number	409033	5.755
Integrity Entry Number	409035	7.328
Integrity Entry Number	409036	6.959
Integrity Entry Number	409037	7.858
Integrity Entry Number	667830	7.562
Integrity Entry Number	292402	6.509
Integrity Entry Number	646990	6.102
Integrity Entry Number	690056	4.000
Integrity Entry Number	690057	6.959
Integrity Entry Number	690055	7.921
Integrity Entry Number	688812	7.796
Integrity Entry Number	326765	7.770
Integrity Entry Number	326767	6.357
Integrity Entry Number	326768	7.328
Integrity Entry Number	773583	5.745
Integrity Entry Number	940027	8.481
NIAID AIDSno	2	5.174
NIAID AIDSno	122	5.620
NIAID AIDSno	297	3.854
NIAID AIDSno	487	5.009
NIAID AIDSno	731	5.866
NIAID AIDSno	733	4.611
NIAID AIDSno	1022	4.481
NIAID AIDSno	1300	3.726

NIAID AIDSno	1333	4.614
NIAID AIDSno	1406	5.051
NIAID AIDSno	1410	5.151
NIAID AIDSno	2319	6.222
NIAID AIDSno	2326	4.955
NIAID AIDSno	2756	7.523
NIAID AIDSno	4250	4.745
NIAID AIDSno	4336	6.538
NIAID AIDSno	5214	4.952
NIAID AIDSno	5499	4.602
NIAID AIDSno	7125	5.738
NIAID AIDSno	7818	4.959
NIAID AIDSno	9001	5.941
NIAID AIDSno	9605	4.706
NIAID AIDSno	10688	4.114
NIAID AIDSno	11885	5.054
NIAID AIDSno	11901	4.456
NIAID AIDSno	11902	5.301
NIAID AIDSno	11914	5.801
NIAID AIDSno	11944	5.022
NIAID AIDSno	11992	3.908
NIAID AIDSno	12116	5.108
NIAID AIDSno	12148	4.314
NIAID AIDSno	12149	4.551
NIAID AIDSno	12168	4.319
NIAID AIDSno	12208	5.569
NIAID AIDSno	12210	4.398
NIAID AIDSno	12211	4.284
NIAID AIDSno	12212	4.108
NIAID AIDSno	12217	6.523
NIAID AIDSno	12218	4.222
NIAID AIDSno	12230	4.699
NIAID AIDSno	12231	4.046
NIAID AIDSno	12232	4.143
NIAID AIDSno	12357	4.013
NIAID AIDSno	12551	4.367
NIAID AIDSno	12783	4.627
NIAID AIDSno	13326	6.000
NIAID AIDSno	13692	3.609
NIAID AIDSno	13883	8.000
NIAID AIDSno	13884	8.004
NIAID AIDSno	14132	4.788
NIAID AIDSno	14274	3.890
NIAID AIDSno	15135	4.187
NIAID AIDSno	15136	3.845

NIAID AIDSno	15137	3.750
NIAID AIDSno	15837	5.046
NIAID AIDSno	16075	4.921
NIAID AIDSno	16099	5.208
NIAID AIDSno	16150	6.247
NIAID AIDSno	16327	4.824
NIAID AIDSno	16872	4.215
NIAID AIDSno	22373	4.000
NIAID AIDSno	22528	5.046
NIAID AIDSno	22798	5.462
NIAID AIDSno	22799	5.198
NIAID AIDSno	22811	5.202
NIAID AIDSno	25075	6.387
NIAID AIDSno	25077	5.215
NIAID AIDSno	25083	4.903
NIAID AIDSno	25085	4.879
NIAID AIDSno	25087	4.658
NIAID AIDSno	25095	5.391
NIAID AIDSno	25109	4.836
NIAID AIDSno	25250	5.658
NIAID AIDSno	25251	5.658
NIAID AIDSno	25252	5.155
NIAID AIDSno	25253	5.398
NIAID AIDSno	26336	5.398
NIAID AIDSno	26339	4.620
NIAID AIDSno	28061	4.393
NIAID AIDSno	28062	6.201
NIAID AIDSno	28600	5.000
NIAID AIDSno	29129	4.721
NIAID AIDSno	29130	4.854
NIAID AIDSno	29331	3.827
NIAID AIDSno	29422	4.622
NIAID AIDSno	29423	4.851
NIAID AIDSno	29424	4.777
NIAID AIDSno	29425	5.004
NIAID AIDSno	29426	5.081
NIAID AIDSno	29434	5.076
NIAID AIDSno	29768	6.199
NIAID AIDSno	30183	2.913
NIAID AIDSno	30245	4.495
NIAID AIDSno	30400	4.634
NIAID AIDSno	30446	4.678
NIAID AIDSno	30447	4.616
NIAID AIDSno	30450	4.987
NIAID AIDSno	30451	4.883

NIAID AIDSno	30452	4.640
NIAID AIDSno	30455	3.837
NIAID AIDSno	30564	4.238
NIAID AIDSno	30652	6.046
NIAID AIDSno	31252	6.000
NIAID AIDSno	31255	4.588
NIAID AIDSno	31279	5.796
NIAID AIDSno	31423	4.000
NIAID AIDSno	31425	4.745
NIAID AIDSno	32343	4.314
NIAID AIDSno	32693	4.116
NIAID AIDSno	32697	4.046
NIAID AIDSno	32700	4.084
NIAID AIDSno	32701	4.081
NIAID AIDSno	32702	4.183
NIAID AIDSno	32705	4.046
NIAID AIDSno	32706	4.046
NIAID AIDSno	32707	4.046
NIAID AIDSno	32711	4.000
NIAID AIDSno	32713	5.602
NIAID AIDSno	32714	4.192
NIAID AIDSno	32717	5.215
NIAID AIDSno	32718	5.310
NIAID AIDSno	32721	4.333
NIAID AIDSno	32723	4.046
NIAID AIDSno	32726	4.660
NIAID AIDSno	32727	4.046
NIAID AIDSno	32729	4.672
NIAID AIDSno	32731	5.886
NIAID AIDSno	32735	4.730
NIAID AIDSno	32736	4.530
NIAID AIDSno	32758	3.751
NIAID AIDSno	32798	3.828
NIAID AIDSno	32800	3.623
NIAID AIDSno	32801	4.134
NIAID AIDSno	32957	6.432
NIAID AIDSno	35190	5.959
NIAID AIDSno	35995	6.460
NIAID AIDSno	38772	3.523
NIAID AIDSno	40146	4.126
NIAID AIDSno	40812	5.602
NIAID AIDSno	42494	5.301
NIAID AIDSno	42742	5.854
NIAID AIDSno	43940	5.301
NIAID AIDSno	44064	4.780

NIAID AIDSno	47344	5.046
NIAID AIDSno	47439	4.721
NIAID AIDSno	50663	3.764
NIAID AIDSno	50664	5.328
NIAID AIDSno	50665	4.205
NIAID AIDSno	50668	4.278
NIAID AIDSno	50675	6.092
NIAID AIDSno	50677	6.959
NIAID AIDSno	50680	5.284
NIAID AIDSno	50684	5.959
NIAID AIDSno	50688	5.237
NIAID AIDSno	50689	4.301
NIAID AIDSno	51077	5.553
NIAID AIDSno	51079	5.959
NIAID AIDSno	51085	4.468
NIAID AIDSno	51096	5.523
NIAID AIDSno	51103	6.048
NIAID AIDSno	51108	6.523
NIAID AIDSno	51109	5.444
NIAID AIDSno	51112	5.602
NIAID AIDSno	51114	6.000
NIAID AIDSno	51140	3.658
NIAID AIDSno	51142	3.845
NIAID AIDSno	51190	5.699
NIAID AIDSno	51198	5.105
NIAID AIDSno	51229	4.682
NIAID AIDSno	57469	4.097
NIAID AIDSno	59015	4.754
NIAID AIDSno	59018	4.975
NIAID AIDSno	59022	5.201
NIAID AIDSno	59027	4.606
NIAID AIDSno	59035	4.411
NIAID AIDSno	59095	4.444
NIAID AIDSno	60467	6.000
NIAID AIDSno	60844	4.699
NIAID AIDSno	62756	4.793
NIAID AIDSno	62978	4.018
NIAID AIDSno	65443	4.891
NIAID AIDSno	70961	7.398
NIAID AIDSno	71155	3.777
NIAID AIDSno	71156	3.945
NIAID AIDSno	71157	3.578
NIAID AIDSno	71158	4.027
NIAID AIDSno	71159	3.903
NIAID AIDSno	71160	3.545

NIAID AIDSno	71161	3.816
NIAID AIDSno	71162	3.582
NIAID AIDSno	71163	3.975
NIAID AIDSno	71165	4.133
NIAID AIDSno	71166	3.654
NIAID AIDSno	71167	5.553
NIAID AIDSno	71168	6.222
NIAID AIDSno	71169	6.222
NIAID AIDSno	71170	5.585
NIAID AIDSno	71171	6.046
NIAID AIDSno	71172	5.131
NIAID AIDSno	71173	6.222
NIAID AIDSno	71174	6.301
NIAID AIDSno	71175	5.699
NIAID AIDSno	71176	5.699
NIAID AIDSno	71478	6.319
NIAID AIDSno	72105	6.398
NIAID AIDSno	72107	5.125
NIAID AIDSno	72389	8.000
NIAID AIDSno	72583	6.000
NIAID AIDSno	72623	5.108
NIAID AIDSno	72624	5.119
NIAID AIDSno	72625	5.187
NIAID AIDSno	73314	4.509
NIAID AIDSno	77413	3.523
NIAID AIDSno	80746	4.845
NIAID AIDSno	80747	5.569
NIAID AIDSno	80762	5.056
NIAID AIDSno	80774	5.699
NIAID AIDSno	80775	5.301
NIAID AIDSno	80780	4.351
NIAID AIDSno	80781	4.863
NIAID AIDSno	80783	6.000
NIAID AIDSno	80784	5.301
NIAID AIDSno	80785	5.000
NIAID AIDSno	80786	6.097
NIAID AIDSno	80787	5.721
NIAID AIDSno	80788	6.796
NIAID AIDSno	80789	6.398
NIAID AIDSno	80790	5.301
NIAID AIDSno	80791	6.244
NIAID AIDSno	80792	5.301
NIAID AIDSno	80793	6.770
NIAID AIDSno	80794	5.959
NIAID AIDSno	80795	5.301

NIAID AIDSno	80796	5.301
NIAID AIDSno	80797	5.301
NIAID AIDSno	80798	5.301
NIAID AIDSno	80799	5.301
NIAID AIDSno	80800	5.301
NIAID AIDSno	80801	5.523
NIAID AIDSno	80802	6.071
NIAID AIDSno	80803	5.921
NIAID AIDSno	80804	5.301
NIAID AIDSno	80805	5.000
NIAID AIDSno	80806	6.301
NIAID AIDSno	80807	5.569
NIAID AIDSno	80808	5.000
NIAID AIDSno	80809	5.000
NIAID AIDSno	80810	5.000
NIAID AIDSno	80811	5.301
NIAID AIDSno	80812	5.000
NIAID AIDSno	80814	5.000
NIAID AIDSno	80815	5.301
NIAID AIDSno	81137	3.951
NIAID AIDSno	81408	5.272
NIAID AIDSno	81414	4.000
NIAID AIDSno	81415	4.996
NIAID AIDSno	81416	5.495
NIAID AIDSno	81418	5.553
NIAID AIDSno	81419	5.387
NIAID AIDSno	81420	5.770
NIAID AIDSno	81421	5.770
NIAID AIDSno	81423	5.000
NIAID AIDSno	81427	3.955
NIAID AIDSno	81432	7.301
NIAID AIDSno	82022	4.854
NIAID AIDSno	82024	3.532
NIAID AIDSno	82025	4.504
NIAID AIDSno	82027	3.699
NIAID AIDSno	82028	5.638
NIAID AIDSno	82032	3.854
NIAID AIDSno	82036	5.824
NIAID AIDSno	82037	5.387
NIAID AIDSno	85045	4.046
NIAID AIDSno	85046	4.319
NIAID AIDSno	85047	4.260
NIAID AIDSno	85048	3.644
NIAID AIDSno	85049	3.870
NIAID AIDSno	85050	4.131

NIAID AIDSno	85051	4.201
NIAID AIDSno	85052	4.000
NIAID AIDSno	85053	3.699
NIAID AIDSno	85054	3.480
NIAID AIDSno	85057	3.465
NIAID AIDSno	85058	3.229
NIAID AIDSno	85059	3.661
NIAID AIDSno	85060	3.620
NIAID AIDSno	85061	3.848
NIAID AIDSno	85062	4.328
NIAID AIDSno	85063	4.028
NIAID AIDSno	85066	3.486
NIAID AIDSno	85071	3.523
NIAID AIDSno	85075	4.347
NIAID AIDSno	85412	5.000
NIAID AIDSno	85413	5.072
NIAID AIDSno	85414	4.989
NIAID AIDSno	85924	4.989
NIAID AIDSno	86031	6.699
NIAID AIDSno	86032	6.854
NIAID AIDSno	86033	6.301
NIAID AIDSno	86034	5.125
NIAID AIDSno	86543	5.155
NIAID AIDSno	86545	5.770
NIAID AIDSno	86546	5.046
NIAID AIDSno	87314	5.678
NIAID AIDSno	88104	4.658
NIAID AIDSno	88105	4.602
NIAID AIDSno	88106	4.292
NIAID AIDSno	88107	4.620
NIAID AIDSno	88108	4.854
NIAID AIDSno	88109	4.721
NIAID AIDSno	88131	3.699
NIAID AIDSno	88133	4.602
NIAID AIDSno	88319	3.754
NIAID AIDSno	88397	5.523
NIAID AIDSno	89112	5.770
NIAID AIDSno	89546	5.810
NIAID AIDSno	89547	5.372
NIAID AIDSno	92374	5.187
NIAID AIDSno	92375	5.357
NIAID AIDSno	92376	5.337
NIAID AIDSno	92377	4.510
NIAID AIDSno	92378	4.340
NIAID AIDSno	92385	5.721

NIAID AIDSno	92386	5.276
NIAID AIDSno	92388	4.499
NIAID AIDSno	92389	5.215
NIAID AIDSno	92390	4.164
NIAID AIDSno	92391	4.504
NIAID AIDSno	92392	4.256
NIAID AIDSno	92393	6.222
NIAID AIDSno	92394	4.446
NIAID AIDSno	92395	4.777
NIAID AIDSno	92396	4.662
NIAID AIDSno	92397	4.824
NIAID AIDSno	92398	4.435
NIAID AIDSno	92400	4.413
NIAID AIDSno	92402	5.102
NIAID AIDSno	92406	4.565
NIAID AIDSno	92407	3.632
NIAID AIDSno	92409	4.261
NIAID AIDSno	92644	4.481
NIAID AIDSno	92648	4.161
NIAID AIDSno	92649	5.222
NIAID AIDSno	92650	3.636
NIAID AIDSno	92651	5.000
NIAID AIDSno	92652	5.506
NIAID AIDSno	92653	5.287
NIAID AIDSno	92654	4.138
NIAID AIDSno	92655	4.465
NIAID AIDSno	92657	3.456
NIAID AIDSno	92658	5.699
NIAID AIDSno	92659	4.594
NIAID AIDSno	92660	5.927
NIAID AIDSno	92973	4.167
NIAID AIDSno	94678	5.500
NIAID AIDSno	94679	6.301
NIAID AIDSno	94680	5.495
NIAID AIDSno	94681	6.155
NIAID AIDSno	94682	5.927
NIAID AIDSno	94683	6.481
NIAID AIDSno	94684	6.000
NIAID AIDSno	94685	6.261
NIAID AIDSno	94686	6.199
NIAID AIDSno	94687	6.188
NIAID AIDSno	94688	5.398
NIAID AIDSno	94689	5.886
NIAID AIDSno	94690	6.222
NIAID AIDSno	94691	6.349

NIAID AIDSno	94692	5.638
NIAID AIDSno	94693	6.000
NIAID AIDSno	94694	6.000
NIAID AIDSno	94695	5.456
NIAID AIDSno	94696	6.125
NIAID AIDSno	96006	6.432
NIAID AIDSno	96427	4.125
NIAID AIDSno	96885	6.301
NIAID AIDSno	96886	5.398
NIAID AIDSno	96887	4.264
NIAID AIDSno	97572	5.062
NIAID AIDSno	97573	5.222
NIAID AIDSno	97574	5.048
NIAID AIDSno	97575	4.000
NIAID AIDSno	97587	4.000
NIAID AIDSno	97589	5.046
NIAID AIDSno	97590	5.155
NIAID AIDSno	97591	4.886
NIAID AIDSno	97592	4.921
NIAID AIDSno	97593	4.907
NIAID AIDSno	97594	5.222
NIAID AIDSno	97595	4.000
NIAID AIDSno	97596	4.000
NIAID AIDSno	97597	4.939
NIAID AIDSno	97598	5.125
NIAID AIDSno	97599	4.745
NIAID AIDSno	97600	4.432
NIAID AIDSno	97601	4.509
NIAID AIDSno	97602	4.276
NIAID AIDSno	97603	4.276
NIAID AIDSno	97604	4.602
NIAID AIDSno	97605	5.222
NIAID AIDSno	97606	4.699
NIAID AIDSno	97607	4.569
NIAID AIDSno	97608	4.658
NIAID AIDSno	97609	4.824
NIAID AIDSno	97610	5.046
NIAID AIDSno	97613	4.886
NIAID AIDSno	104313	6.222
NIAID AIDSno	104359	6.000
NIAID AIDSno	104384	6.155
NIAID AIDSno	104386	5.699
NIAID AIDSno	104387	6.000
NIAID AIDSno	104390	5.523
NIAID AIDSno	104400	6.000

NIAID AIDSno	104401	5.699
NIAID AIDSno	104402	6.046
NIAID AIDSno	104403	6.301
NIAID AIDSno	104404	5.699
NIAID AIDSno	104408	6.046
NIAID AIDSno	104410	6.000
NIAID AIDSno	104411	5.699
NIAID AIDSno	104484	6.000
NIAID AIDSno	104486	6.301
NIAID AIDSno	104487	6.301
NIAID AIDSno	104489	6.046
NIAID AIDSno	104491	6.000
NIAID AIDSno	104492	6.301
NIAID AIDSno	104493	6.222
NIAID AIDSno	104494	5.824
NIAID AIDSno	104495	6.301
NIAID AIDSno	104496	6.398
NIAID AIDSno	104497	6.155
NIAID AIDSno	104501	6.523
NIAID AIDSno	104502	6.155
NIAID AIDSno	104503	5.699
NIAID AIDSno	104504	6.155
NIAID AIDSno	104506	6.398
NIAID AIDSno	104507	6.398
NIAID AIDSno	104508	6.301
NIAID AIDSno	104509	6.155
NIAID AIDSno	104510	6.398
NIAID AIDSno	104511	6.523
NIAID AIDSno	105218	5.201
NIAID AIDSno	105219	6.678
NIAID AIDSno	105220	6.745
NIAID AIDSno	105221	7.199
NIAID AIDSno	105222	7.000
NIAID AIDSno	105223	6.301
NIAID AIDSno	105224	6.261
NIAID AIDSno	105227	6.562
NIAID AIDSno	105229	6.713
NIAID AIDSno	105231	6.761
NIAID AIDSno	105232	6.854
NIAID AIDSno	105235	7.921
NIAID AIDSno	106467	6.398
NIAID AIDSno	106468	7.745
NIAID AIDSno	106479	7.432
NIAID AIDSno	107006	4.921
NIAID AIDSno	107007	5.023

NIAID AIDSno	107008	5.143
NIAID AIDSno	107991	4.502
NIAID AIDSno	107992	4.770
NIAID AIDSno	107993	4.056
NIAID AIDSno	108201	5.225
NIAID AIDSno	109546	4.921
NIAID AIDSno	109548	5.046
NIAID AIDSno	109549	5.398
NIAID AIDSno	110024	4.097
NIAID AIDSno	110025	3.921
NIAID AIDSno	110026	5.509
NIAID AIDSno	110027	5.046
NIAID AIDSno	110679	4.161
NIAID AIDSno	112641	4.921
NIAID AIDSno	112642	5.398
NIAID AIDSno	112699	3.410
NIAID AIDSno	113433	4.347
NIAID AIDSno	113434	4.066
NIAID AIDSno	113531	4.523
NIAID AIDSno	113534	3.721
NIAID AIDSno	113541	4.469
NIAID AIDSno	113936	4.252
NIAID AIDSno	122033	6.775
NIAID AIDSno	122034	4.319
NIAID AIDSno	122858	5.699
NIAID AIDSno	122859	6.886
NIAID AIDSno	122860	4.482
NIAID AIDSno	122861	5.108
NIAID AIDSno	122862	4.244
NIAID AIDSno	122864	5.893
NIAID AIDSno	122865	5.187
NIAID AIDSno	122866	5.161
NIAID AIDSno	122867	4.821
NIAID AIDSno	122872	4.469
NIAID AIDSno	122873	5.721
NIAID AIDSno	122875	5.745
NIAID AIDSno	122876	4.398
NIAID AIDSno	122881	3.107
NIAID AIDSno	122885	3.696
NIAID AIDSno	124992	3.679
NIAID AIDSno	125940	3.221
NIAID AIDSno	127545	3.393
NIAID AIDSno	134333	3.478
NIAID AIDSno	135976	4.025
NIAID AIDSno	153756	4.367

NIAID AIDSno	153890	6.048
NIAID AIDSno	153891	4.367
NIAID AIDSno	153892	4.203
NIAID AIDSno	154159	5.398
NIAID AIDSno	154160	5.215
NIAID AIDSno	160821	3.222
NIAID AIDSno	165866	6.018
NIAID AIDSno	165867	6.824
NIAID AIDSno	165868	6.495
NIAID AIDSno	165869	6.444
NIAID AIDSno	165870	6.525
NIAID AIDSno	165871	5.215
NIAID AIDSno	165872	5.071
NIAID AIDSno	165873	4.745
NIAID AIDSno	165874	4.638
NIAID AIDSno	165875	5.824
NIAID AIDSno	167875	4.469
NIAID AIDSno	167877	4.854
NIAID AIDSno	167879	6.292
NIAID AIDSno	167880	5.569
NIAID AIDSno	167881	5.301
NIAID AIDSno	167882	5.301
NIAID AIDSno	167884	5.301
NIAID AIDSno	167885	6.060
NIAID AIDSno	167886	6.326
NIAID AIDSno	167887	6.433
NIAID AIDSno	167888	6.998
NIAID AIDSno	167889	6.364
NIAID AIDSno	167890	6.023
NIAID AIDSno	167891	6.252
NIAID AIDSno	167892	5.301
NIAID AIDSno	167893	6.821
NIAID AIDSno	167895	6.208
NIAID AIDSno	167896	5.301
NIAID AIDSno	167897	6.000
NIAID AIDSno	167900	6.147
NIAID AIDSno	167902	5.553
NIAID AIDSno	167903	6.439
NIAID AIDSno	167904	6.592
NIAID AIDSno	167905	6.810
NIAID AIDSno	167906	5.745
NIAID AIDSno	167907	5.921
NIAID AIDSno	167908	6.240
NIAID AIDSno	167909	5.796
NIAID AIDSno	167910	5.854

NIAID AIDSno	167911	6.654
NIAID AIDSno	167912	6.078
NIAID AIDSno	167913	5.943
NIAID AIDSno	167914	6.355
NIAID AIDSno	167915	6.818
NIAID AIDSno	167917	6.351
NIAID AIDSno	167920	5.939
NIAID AIDSno	167921	5.887
NIAID AIDSno	167922	5.403
NIAID AIDSno	167924	5.481
NIAID AIDSno	167926	6.427
NIAID AIDSno	167928	5.276
NIAID AIDSno	167929	5.959
NIAID AIDSno	167930	5.921
NIAID AIDSno	167931	6.208
NIAID AIDSno	167932	5.745
NIAID AIDSno	167935	5.301
NIAID AIDSno	167936	5.149
NIAID AIDSno	167941	6.461
NIAID AIDSno	167942	6.395
NIAID AIDSno	167943	6.217
NIAID AIDSno	167945	5.658
NIAID AIDSno	167946	6.086
NIAID AIDSno	167947	5.921
NIAID AIDSno	167948	6.417
NIAID AIDSno	167949	5.328
NIAID AIDSno	167952	6.600
NIAID AIDSno	167954	5.593
NIAID AIDSno	167955	5.648
NIAID AIDSno	167956	5.387
NIAID AIDSno	167957	5.585
NIAID AIDSno	167958	5.699
NIAID AIDSno	167962	6.079
NIAID AIDSno	167966	5.034
NIAID AIDSno	167967	5.155
NIAID AIDSno	167969	5.538
NIAID AIDSno	167972	5.585
NIAID AIDSno	167981	5.276
NIAID AIDSno	167983	5.215
NIAID AIDSno	167984	5.699
NIAID AIDSno	167985	5.187
NIAID AIDSno	170300	6.959
NIAID AIDSno	170307	4.155
NIAID AIDSno	170369	5.228
NIAID AIDSno	170370	5.020

NIAID AIDSno	170371	5.620
NIAID AIDSno	170372	6.523
NIAID AIDSno	170373	6.699
NIAID AIDSno	170374	5.959
NIAID AIDSno	170375	5.377
NIAID AIDSno	170376	5.854
NIAID AIDSno	170377	5.046
NIAID AIDSno	170378	5.678
NIAID AIDSno	170379	6.222
NIAID AIDSno	170380	3.745
NIAID AIDSno	170381	5.523
NIAID AIDSno	170382	6.699
NIAID AIDSno	170383	5.125
NIAID AIDSno	170384	5.770
NIAID AIDSno	170385	4.620
NIAID AIDSno	171844	4.678
NIAID AIDSno	175846	4.824
NIAID AIDSno	184703	4.745
NIAID AIDSno	185448	6.770
NIAID AIDSno	186226	5.770
NIAID AIDSno	186229	3.585
NIAID AIDSno	188013	5.796
NIAID AIDSno	188014	6.301
NIAID AIDSno	188015	5.046
NIAID AIDSno	188016	5.959
NIAID AIDSno	188017	6.523
NIAID AIDSno	188020	5.155
NIAID AIDSno	188703	7.432
NIAID AIDSno	189854	4.161
NIAID AIDSno	189862	5.151
NIAID AIDSno	190946	5.046
NIAID AIDSno	190952	6.046
NIAID AIDSno	190955	5.959
NIAID AIDSno	190958	5.658
NIAID AIDSno	190961	6.301
NIAID AIDSno	190962	6.222
NIAID AIDSno	190963	6.398
NIAID AIDSno	190964	5.638
NIAID AIDSno	190965	6.046
NIAID AIDSno	190966	6.155
NIAID AIDSno	190967	6.301
NIAID AIDSno	190968	5.398
NIAID AIDSno	190969	6.398
NIAID AIDSno	190970	6.000
NIAID AIDSno	190971	6.523

NIAID AIDSno	190972	6.523
NIAID AIDSno	190973	5.367
NIAID AIDSno	190974	5.921
NIAID AIDSno	190975	5.721
NIAID AIDSno	190976	6.699
NIAID AIDSno	195737	5.155
NIAID AIDSno	195738	5.125
NIAID AIDSno	195739	4.187
NIAID AIDSno	195740	4.046
NIAID AIDSno	195741	4.143
NIAID AIDSno	195742	4.022
NIAID AIDSno	195743	4.174
NIAID AIDSno	195744	4.056
NIAID AIDSno	195745	4.387
NIAID AIDSno	195746	4.301
NIAID AIDSno	195747	4.036
NIAID AIDSno	195748	4.347
NIAID AIDSno	208905	4.167
NIAID AIDSno	208906	4.301
NIAID AIDSno	209006	3.000
NIAID AIDSno	209008	3.071
NIAID AIDSno	209009	3.046
NIAID AIDSno	209013	3.347
NIAID AIDSno	209014	3.187
NIAID AIDSno	209016	3.478
NIAID AIDSno	209017	3.222
NIAID AIDSno	209018	3.155
NIAID AIDSno	209019	3.222
NIAID AIDSno	209020	3.222
NIAID AIDSno	209022	3.222
NIAID AIDSno	209029	3.155
NIAID AIDSno	209880	6.699
NIAID AIDSno	210507	6.301
NIAID AIDSno	210508	5.741
NIAID AIDSno	210601	4.387
NIAID AIDSno	211927	4.921
NIAID AIDSno	211931	4.481
NIAID AIDSno	211932	4.523
NIAID AIDSno	211933	4.187
NIAID AIDSno	211934	4.377
NIAID AIDSno	211936	4.301
NIAID AIDSno	211937	5.155
NIAID AIDSno	211938	5.000
NIAID AIDSno	211939	5.523
NIAID AIDSno	211940	4.602

NIAID AIDSno	211941	4.602
NIAID AIDSno	211942	5.155
NIAID AIDSno	211943	4.046
NIAID AIDSno	211944	4.699
NIAID AIDSno	211945	4.721
NIAID AIDSno	211946	5.097
NIAID AIDSno	214682	3.770
NIAID AIDSno	214683	4.056
NIAID AIDSno	214684	4.032
NIAID AIDSno	214685	5.310
NIAID AIDSno	214686	5.745
NIAID AIDSno	214687	4.620
NIAID AIDSno	214688	5.036
NIAID AIDSno	214689	5.745
NIAID AIDSno	214690	5.585
NIAID AIDSno	214691	4.036
NIAID AIDSno	214692	3.738
NIAID AIDSno	214693	4.201
NIAID AIDSno	214694	4.268
NIAID AIDSno	214695	4.387
NIAID AIDSno	214696	4.678
NIAID AIDSno	214697	4.721
NIAID AIDSno	214698	5.469
NIAID AIDSno	214699	4.215
NIAID AIDSno	214700	4.553
NIAID AIDSno	214701	5.149
NIAID AIDSno	214702	5.620
NIAID AIDSno	214703	4.658
NIAID AIDSno	214704	3.523
NIAID AIDSno	214705	3.523
NIAID AIDSno	214706	3.523
NIAID AIDSno	214707	5.495
NIAID AIDSno	214708	5.131
NIAID AIDSno	214709	5.678
NIAID AIDSno	214710	5.495
NIAID AIDSno	214711	4.886
NIAID AIDSno	214712	4.347
NIAID AIDSno	214713	5.585
NIAID AIDSno	214714	4.807
NIAID AIDSno	214715	4.611
NIAID AIDSno	214779	4.686
NIAID AIDSno	214782	6.658
NIAID AIDSno	214783	7.097
NIAID AIDSno	214784	7.398
NIAID AIDSno	214786	7.097

NIAID AIDSno	214789	7.523
NIAID AIDSno	214790	8.000
NIAID AIDSno	214791	6.398
NIAID AIDSno	214792	6.959
NIAID AIDSno	214794	7.398
NIAID AIDSno	214795	7.699
NIAID AIDSno	214796	8.000
NIAID AIDSno	214797	7.222
NIAID AIDSno	214799	7.398
NIAID AIDSno	217988	4.824
NIAID AIDSno	217989	4.854
NIAID AIDSno	217990	5.252
NIAID AIDSno	217991	5.319
NIAID AIDSno	217992	4.071
NIAID AIDSno	217998	4.398
NIAID AIDSno	218009	4.921
NIAID AIDSno	218010	4.921
NIAID AIDSno	218018	4.939
NIAID AIDSno	218111	4.481
NIAID AIDSno	219897	5.222
NIAID AIDSno	219898	5.699
NIAID AIDSno	219899	5.796
NIAID AIDSno	219900	5.301
NIAID AIDSno	219901	6.000
NIAID AIDSno	219902	5.699
NIAID AIDSno	219903	5.699
NIAID AIDSno	219904	5.523
NIAID AIDSno	219905	5.523
NIAID AIDSno	219906	5.523
NIAID AIDSno	219907	6.761
NIAID AIDSno	219908	4.347
NIAID AIDSno	219909	4.301
NIAID AIDSno	219911	4.187
NIAID AIDSno	219912	4.854
NIAID AIDSno	219913	4.222
NIAID AIDSno	219914	4.585
NIAID AIDSno	219967	4.187
NIAID AIDSno	219968	4.387
NIAID AIDSno	219969	3.943
NIAID AIDSno	223305	4.921
NIAID AIDSno	223307	4.870
NIAID AIDSno	223308	4.770
NIAID AIDSno	223317	4.886
NIAID AIDSno	223320	4.921
NIAID AIDSno	223720	8.000

NIAID AIDSno	223721	6.959
NIAID AIDSno	223829	6.631
NIAID AIDSno	223830	6.306
NIAID AIDSno	223831	6.398
NIAID AIDSno	223832	6.551
NIAID AIDSno	223834	6.334
NIAID AIDSno	223835	5.857
NIAID AIDSno	223836	6.147
NIAID AIDSno	223837	6.156
NIAID AIDSno	223872	4.301
NIAID AIDSno	224586	5.824
NIAID AIDSno	225299	4.959
NIAID AIDSno	225300	4.770
NIAID AIDSno	225301	4.456
NIAID AIDSno	225302	4.959
NIAID AIDSno	225303	4.420
NIAID AIDSno	225304	4.638
NIAID AIDSno	225305	4.602
NIAID AIDSno	225306	4.638
NIAID AIDSno	225307	4.770
NIAID AIDSno	225311	4.071
NIAID AIDSno	225312	4.066
NIAID AIDSno	225318	4.000
NIAID AIDSno	225320	4.046
NIAID AIDSno	225322	4.097
NIAID AIDSno	225324	4.745
NIAID AIDSno	225325	4.770
NIAID AIDSno	225327	4.125
NIAID AIDSno	225328	4.161
NIAID AIDSno	225330	4.357
NIAID AIDSno	225332	4.000
NIAID AIDSno	225333	4.032
NIAID AIDSno	225334	4.076
NIAID AIDSno	225336	4.569
NIAID AIDSno	225338	4.420
NIAID AIDSno	225339	4.000
NIAID AIDSno	225341	4.000
NIAID AIDSno	225343	4.367
NIAID AIDSno	225344	4.495
NIAID AIDSno	225345	4.921
NIAID AIDSno	225346	4.357
NIAID AIDSno	225347	5.097
NIAID AIDSno	225348	4.137
NIAID AIDSno	225350	4.125
NIAID AIDSno	226954	3.824

NIAID AIDSno	226958	4.000
NIAID AIDSno	226959	5.097
NIAID AIDSno	226960	4.367
NIAID AIDSno	226961	4.398
NIAID AIDSno	226966	4.092
NIAID AIDSno	226967	4.013
NIAID AIDSno	226968	4.180
NIAID AIDSno	226969	4.420
NIAID AIDSno	226970	4.886
NIAID AIDSno	226972	4.950
NIAID AIDSno	226973	4.481
NIAID AIDSno	226975	4.155
NIAID AIDSno	227244	5.850
NIAID AIDSno	227459	6.420
NIAID AIDSno	227460	6.590
NIAID AIDSno	227461	5.440
NIAID AIDSno	227462	6.680
NIAID AIDSno	227463	4.310
NIAID AIDSno	227464	4.980
NIAID AIDSno	227466	5.620
NIAID AIDSno	227467	5.660
NIAID AIDSno	227468	5.835
NIAID AIDSno	227470	5.000
NIAID AIDSno	227471	6.250
NIAID AIDSno	227472	5.850
NIAID AIDSno	227473	6.090
NIAID AIDSno	227474	6.250
NIAID AIDSno	227475	5.700
NIAID AIDSno	227476	6.370
NIAID AIDSno	227477	6.370
NIAID AIDSno	227478	6.110
NIAID AIDSno	227479	5.800
NIAID AIDSno	227480	5.690
NIAID AIDSno	227481	6.370
NIAID AIDSno	227482	6.250
NIAID AIDSno	227483	6.380
NIAID AIDSno	227484	6.360
NIAID AIDSno	227485	6.450
NIAID AIDSno	227486	6.429
NIAID AIDSno	227487	6.750
NIAID AIDSno	227488	6.660
NIAID AIDSno	227489	6.380
NIAID AIDSno	227490	6.951
NIAID AIDSno	227491	6.370
NIAID AIDSno	227492	6.200

NIAID AIDSno	227493	5.170
NIAID AIDSno	227494	6.170
NIAID AIDSno	227495	6.300
NIAID AIDSno	227496	6.000
NIAID AIDSno	227497	5.840
NIAID AIDSno	227498	5.690
NIAID AIDSno	227875	4.301
NIAID AIDSno	228381	7.959
NIAID AIDSno	228382	7.903
NIAID AIDSno	228383	7.893
NIAID AIDSno	228384	7.947
NIAID AIDSno	228389	7.561
NIAID AIDSno	230859	6.699
NIAID AIDSno	231382	4.699
NIAID AIDSno	231383	4.699
NIAID AIDSno	241598	7.678
NIAID AIDSno	241599	7.469
NIAID AIDSno	241602	5.509
NIAID AIDSno	241604	7.456
NIAID AIDSno	241605	7.585
NIAID AIDSno	241607	7.420
NIAID AIDSno	241610	6.936
NIAID AIDSno	241622	7.114
NIAID AIDSno	241625	7.495
NIAID AIDSno	241633	7.081
NIAID AIDSno	241634	7.509
NIAID AIDSno	241635	7.260
NIAID AIDSno	241636	7.187
NIAID AIDSno	241643	6.668
NIAID AIDSno	241661	7.357
NIAID AIDSno	241681	8.041
NIAID AIDSno	241692	7.367
NIAID AIDSno	241693	6.171
NIAID AIDSno	241694	7.623
NIAID AIDSno	241698	7.301
NIAID AIDSno	241722	8.046
NIAID AIDSno	241746	7.602
NIAID AIDSno	241748	7.076
NIAID AIDSno	241750	7.387
NIAID AIDSno	241753	8.252
NIAID AIDSno	241767	7.842
NIAID AIDSno	241768	8.237
NIAID AIDSno	241773	8.237
NIAID AIDSno	241777	8.174
NIAID AIDSno	284313	8.222

NIAID AIDSno	284314	8.155
NIAID AIDSno	284378	7.585
NIAID AIDSno	284379	7.357
NIAID AIDSno	284380	6.469
NIAID AIDSno	298346	4.036
NIAID AIDSno	298970	4.620
NIAID AIDSno	299070	4.658
NIAID AIDSno	299483	4.602
NIAID AIDSno	299965	4.886
NIAID AIDSno	300942	4.770
NIAID AIDSno	301243	4.796
NIAID AIDSno	301431	4.268
NIAID AIDSno	303316	4.585
NIAID AIDSno	309600	5.022
NIAID AIDSno	313372	4.097
NIAID AIDSno	314961	4.161
NIAID AIDSno	325404	4.046
NIAID AIDSno	327231	3.403
NIAID AIDSno	329670	3.201
NIAID AIDSno	332207	4.102
NIAID AIDSno	335819	4.155
NIAID AIDSno	339006	5.402
NIAID AIDSno	339007	5.165
NIAID AIDSno	339008	5.615
NIAID AIDSno	339009	5.293
NIAID AIDSno	339010	5.411
NIAID AIDSno	339011	5.550
NIAID AIDSno	339012	5.488
NIAID AIDSno	339013	5.730
NIAID AIDSno	339014	4.655
NIAID AIDSno	339015	4.161
NIAID AIDSno	339016	4.762
NIAID AIDSno	342642	7.483
NIAID AIDSno	342660	7.481
NIAID AIDSno	343235	3.699
NIAID AIDSno	343665	8.000
NIAID AIDSno	343667	8.000
NIAID AIDSno	343671	8.000
NIAID AIDSno	345144	4.244
NIAID AIDSno	346037	5.268
NIAID AIDSno	346038	5.824
NIAID AIDSno	346039	7.523
NIAID AIDSno	346040	7.155
NIAID AIDSno	346041	6.167
NIAID AIDSno	346042	5.609

NIAID AIDSno	346043	7.824
NIAID AIDSno	346044	7.000
NIAID AIDSno	346045	8.000
NIAID AIDSno	346046	7.678
NIAID AIDSno	346047	8.398
NIAID AIDSno	346048	4.959
NIAID AIDSno	346049	5.585
NIAID AIDSno	346050	6.108
NIAID AIDSno	346051	3.921
NIAID AIDSno	346052	5.276
NIAID AIDSno	346053	5.921
NIAID AIDSno	346054	4.770
NIAID AIDSno	346055	5.149
NIAID AIDSno	346056	4.745
NIAID AIDSno	346057	5.509
NIAID AIDSno	346058	5.432
NIAID AIDSno	346059	5.420
NIAID AIDSno	346060	5.770
NIAID AIDSno	346061	5.959
NIAID AIDSno	346064	4.208
NIAID AIDSno	346068	5.222
NIAID AIDSno	346069	5.155
NIAID AIDSno	346070	4.201
NIAID AIDSno	346071	5.000
NIAID AIDSno	346072	5.000
NIAID AIDSno	346073	5.155
NIAID AIDSno	346074	4.678
NIAID AIDSno	346075	4.770
NIAID AIDSno	346076	4.252
NIAID AIDSno	346077	4.638
NIAID AIDSno	346078	4.420
NIAID AIDSno	346079	4.620
NIAID AIDSno	346080	4.553
NIAID AIDSno	346081	4.046
NIAID AIDSno	346082	4.553
NIAID AIDSno	346083	4.509
NIAID AIDSno	346084	4.420
NIAID AIDSno	346085	4.230
NIAID AIDSno	346086	4.229
NIAID AIDSno	346087	4.252
NIAID AIDSno	346088	4.215
NIAID AIDSno	346089	4.174
NIAID AIDSno	346090	4.167
NIAID AIDSno	346091	4.102
NIAID AIDSno	346092	4.041

NIAID AIDSno	346093	4.071
NIAID AIDSno	346094	4.553
NIAID AIDSno	346095	4.538
NIAID AIDSno	346097	4.187
NIAID AIDSno	346098	4.027
NIAID AIDSno	346099	4.000
NIAID AIDSno	346100	4.149
NIAID AIDSno	347705	6.301
NIAID AIDSno	347722	5.953
NIAID AIDSno	347724	5.155
NIAID AIDSno	347727	4.899
NIAID AIDSno	347728	5.432
NIAID AIDSno	347729	4.524
NIAID AIDSno	348157	4.301
NIAID AIDSno	348158	4.237
NIAID AIDSno	348182	3.824
NIAID AIDSno	348579	3.102
NIAID AIDSno	350646	5.358
NIAID AIDSno	350647	7.362
NIAID AIDSno	350648	7.616
NIAID AIDSno	350649	8.086
NIAID AIDSno	350846	7.076
NIAID AIDSno	350847	7.281
NIAID AIDSno	350848	6.710
NIAID AIDSno	350849	6.465
NIAID AIDSno	350850	6.815
NIAID AIDSno	350851	6.967
NIAID AIDSno	350852	6.854
NIAID AIDSno	350853	7.347
NIAID AIDSno	350854	7.699
NIAID AIDSno	350855	7.076
NIAID AIDSno	350856	7.432
NIAID AIDSno	350857	7.444
NIAID AIDSno	350858	6.158
NIAID AIDSno	350859	6.268
NIAID AIDSno	350860	7.387
NIAID AIDSno	350861	6.965
NIAID AIDSno	350862	6.524
NIAID AIDSno	350863	6.328
NIAID AIDSno	350864	6.662
NIAID AIDSno	350865	5.812
NIAID AIDSno	350866	6.287
NIAID AIDSno	350867	5.886
NIAID AIDSno	350868	5.886
NIAID AIDSno	350869	5.983

NIAID AIDSno	350870	5.764
NIAID AIDSno	350871	6.114
NIAID AIDSno	350872	5.097
NIAID AIDSno	350873	6.206
NIAID AIDSno	350874	5.770
NIAID AIDSno	350875	6.095
NIAID AIDSno	350876	6.730
NIAID AIDSno	350877	6.668
NIAID AIDSno	350878	6.334
NIAID AIDSno	350879	6.310
NIAID AIDSno	350880	6.351
NIAID AIDSno	350881	6.091
NIAID AIDSno	350882	6.277
NIAID AIDSno	350883	6.839
NIAID AIDSno	350885	7.569
NIAID AIDSno	350886	6.431
NIAID AIDSno	350887	5.648
NIAID AIDSno	350888	6.530
NIAID AIDSno	350889	6.040
NIAID AIDSno	350890	6.124
NIAID AIDSno	350891	6.107
NIAID AIDSno	350892	6.445
NIAID AIDSno	350893	6.127
NIAID AIDSno	350894	6.666
NIAID AIDSno	350895	6.556
NIAID AIDSno	350896	6.266
NIAID AIDSno	350897	6.233
NIAID AIDSno	350898	6.656
NIAID AIDSno	350899	6.283
NIAID AIDSno	350900	6.038
NIAID AIDSno	350901	6.373
NIAID AIDSno	350902	6.484
NIAID AIDSno	350903	6.204
NIAID AIDSno	350904	6.447
NIAID AIDSno	350905	6.548
NIAID AIDSno	350906	6.260
NIAID AIDSno	350907	6.122
NIAID AIDSno	350908	6.479
NIAID AIDSno	350909	6.388
NIAID AIDSno	350910	6.182
NIAID AIDSno	350911	6.182
NIAID AIDSno	350912	6.447
NIAID AIDSno	350913	6.418
NIAID AIDSno	350914	6.396
NIAID AIDSno	350915	6.316

NIAID AIDSno	350916	6.243
NIAID AIDSno	350917	6.614
NIAID AIDSno	350918	6.302
NIAID AIDSno	350919	6.198
NIAID AIDSno	350920	6.021
NIAID AIDSno	350921	6.500
NIAID AIDSno	350922	6.650
NIAID AIDSno	350923	6.503
NIAID AIDSno	350924	6.089
NIAID AIDSno	350925	6.623
NIAID AIDSno	350926	6.150
NIAID AIDSno	350927	6.674
NIAID AIDSno	350928	6.285
NIAID AIDSno	350929	6.372
NIAID AIDSno	350930	6.728
NIAID AIDSno	350931	6.541
NIAID AIDSno	350932	6.896
NIAID AIDSno	350933	6.292
NIAID AIDSno	350934	6.507
NIAID AIDSno	350935	6.592
NIAID AIDSno	350936	6.558
NIAID AIDSno	350937	6.569
NIAID AIDSno	350938	6.326
NIAID AIDSno	350939	6.092
NIAID AIDSno	350940	6.154
NIAID AIDSno	350941	6.740
NIAID AIDSno	350942	8.195
NIAID AIDSno	350944	7.674
NIAID AIDSno	350945	7.201
NIAID AIDSno	350946	7.854
NIAID AIDSno	350947	7.699
NIAID AIDSno	350948	7.303
NIAID AIDSno	350950	6.824
NIAID AIDSno	350953	6.699
NIAID AIDSno	350954	7.244
NIAID AIDSno	350955	7.409
NIAID AIDSno	350956	7.244
NIAID AIDSno	350959	7.292
NIAID AIDSno	350966	4.350
NIAID AIDSno	351979	4.114
NIAID AIDSno	351980	5.538
NIAID AIDSno	351981	6.538
NIAID AIDSno	351982	7.783
NIAID AIDSno	351983	5.187
NIAID AIDSno	352034	4.638

NIAID AIDSno	352035	4.398
NIAID AIDSno	352039	3.456
NIAID AIDSno	352042	5.000
NIAID AIDSno	352043	4.252
NIAID AIDSno	352876	7.602
NIAID AIDSno	352877	7.398
NIAID AIDSno	352878	7.523
NIAID AIDSno	352879	7.237
NIAID AIDSno	352880	7.602
NIAID AIDSno	352881	7.398
NIAID AIDSno	352882	7.824
NIAID AIDSno	352883	7.699
NIAID AIDSno	352884	8.155
NIAID AIDSno	352885	7.398
NIAID AIDSno	352886	8.000
NIAID AIDSno	352983	3.620
NIAID AIDSno	352986	3.523
NIAID AIDSno	352987	5.000
NIAID AIDSno	352988	4.097
NIAID AIDSno	352994	3.648
NIAID AIDSno	352995	3.180
NIAID AIDSno	354108	3.398
NIAID AIDSno	354109	3.564
NIAID AIDSno	354110	3.222
NIAID AIDSno	354111	3.796
NIAID AIDSno	354112	3.323
NIAID AIDSno	354113	3.303
NIAID AIDSno	354114	3.699
NIAID AIDSno	354115	3.323
NIAID AIDSno	356387	5.222
NIAID AIDSno	356388	4.538
NIAID AIDSno	356389	5.585
NIAID AIDSno	356390	5.523
NIAID AIDSno	356391	4.921
NIAID AIDSno	356392	5.310
NIAID AIDSno	356393	5.518
NIAID AIDSno	356394	4.745
NIAID AIDSno	356395	5.877
NIAID AIDSno	356396	5.585
NIAID AIDSno	362561	7.301
NIAID AIDSno	362562	6.678
NIAID AIDSno	362563	4.620
NIAID AIDSno	362564	7.481
NIAID AIDSno	362565	6.593
NIAID AIDSno	362566	7.032

NIAID AIDSno	362567	8.143
NIAID AIDSno	362568	5.269
NIAID AIDSno	362569	7.155
NIAID AIDSno	362570	7.097
NIAID AIDSno	362571	5.620
NIAID AIDSno	362572	6.119
NIAID AIDSno	362573	5.921
NIAID AIDSno	362574	5.678
NIAID AIDSno	362575	6.456
NIAID AIDSno	362576	6.475
NIAID AIDSno	362577	6.721
NIAID AIDSno	362578	7.444
NIAID AIDSno	362579	7.056
NIAID AIDSno	362580	6.824
NIAID AIDSno	362581	8.155
NIAID AIDSno	362582	6.678
NIAID AIDSno	362583	7.444
NIAID AIDSno	362584	7.194
NIAID AIDSno	362585	6.509
NIAID AIDSno	362586	6.530
NIAID AIDSno	362587	7.229
NIAID AIDSno	362588	6.089
NIAID AIDSno	362589	6.377
NIAID AIDSno	362590	6.447
NIAID AIDSno	367057	4.420
NIAID AIDSno	367847	4.400
NIAID AIDSno	370481	4.367
NIAID AIDSno	375768	4.244
NIAID AIDSno	386153	7.523
NIAID AIDSno	386154	7.699
NIAID AIDSno	386158	5.796
NIAID AIDSno	386159	6.398
NIAID AIDSno	386160	6.824
NIAID AIDSno	386161	5.523
NIAID AIDSno	411070	4.886
NIAID AIDSno	411071	4.336
NIAID AIDSno	411072	4.311
NIAID AIDSno	411073	4.378
NIAID AIDSno	411074	4.442
NIAID AIDSno	411075	4.659
NIAID AIDSno	411076	4.409
NIAID AIDSno	411156	4.469
NIAID AIDSno	411168	4.796
NIAID AIDSno	411171	3.301
NIAID AIDSno	411172	4.699

NIAID AIDSno	411174	4.222
NIAID AIDSno	411706	5.377
NIAID AIDSno	411709	5.910
NIAID AIDSno	411710	4.959
NIAID AIDSno	411716	4.456
NIAID AIDSno	411717	3.550
NIAID AIDSno	411718	5.398
NIAID AIDSno	411719	5.824
NIAID AIDSno	411720	6.155
NIAID AIDSno	411721	7.523
NIAID AIDSno	411722	6.721
NIAID AIDSno	411723	5.678
NIAID AIDSno	411724	6.398
NIAID AIDSno	411725	6.000
NIAID AIDSno	411726	4.762
NIAID AIDSno	411727	5.155
NIAID AIDSno	411728	5.155
NIAID AIDSno	411729	6.032
NIAID AIDSno	411730	5.699
NIAID AIDSno	411952	4.854
NIAID AIDSno	411953	4.472
NIAID AIDSno	411954	4.390
NIAID AIDSno	411955	4.907
NIAID AIDSno	413222	3.479
NIAID AIDSno	413223	3.717
NIAID AIDSno	413224	7.229
NIAID AIDSno	413226	4.174
NIAID AIDSno	413227	6.750
NIAID AIDSno	413228	6.438
NIAID AIDSno	413229	6.432
NIAID AIDSno	413230	6.010
NIAID AIDSno	413231	5.347
NIAID AIDSno	413232	6.487
NIAID AIDSno	413233	7.469
NIAID AIDSno	413234	7.443
NIAID AIDSno	413235	4.770
NIAID AIDSno	413236	6.569
NIAID AIDSno	413237	6.260
NIAID AIDSno	413239	6.678
NIAID AIDSno	413240	5.638
NIAID AIDSno	413241	5.638
NIAID AIDSno	413242	6.444
NIAID AIDSno	413243	6.000
NIAID AIDSno	413246	3.558
NIAID AIDSno	413247	4.661

NIAID AIDSno	413248	4.684
NIAID AIDSno	413249	3.604
NIAID AIDSno	413630	7.699
NIAID AIDSno	413631	7.699
NIAID AIDSno	413632	7.699
NIAID AIDSno	413633	7.699
NIAID AIDSno	413634	7.699
NIAID AIDSno	413635	7.699
NIAID AIDSno	413636	7.699
NIAID AIDSno	413637	7.699
NIAID AIDSno	413638	7.699
NIAID AIDSno	413639	7.699
NIAID AIDSno	413640	7.699
NIAID AIDSno	413641	7.699
NIAID AIDSno	413642	7.699
NIAID AIDSno	413643	7.699
NIAID AIDSno	413644	7.699
NIAID AIDSno	413645	7.699
NIAID AIDSno	413646	7.699
NIAID AIDSno	413647	7.699
NIAID AIDSno	413648	8.111
NIAID AIDSno	413649	8.111
NIAID AIDSno	413650	7.699
NIAID AIDSno	413651	7.000
NIAID AIDSno	413652	8.000
NIAID AIDSno	413653	7.000
NIAID AIDSno	413654	7.000
NIAID AIDSno	413655	7.699
NIAID AIDSno	413656	7.872
NIAID AIDSno	413657	7.699
NIAID AIDSno	413658	7.699
NIAID AIDSno	413659	7.699
NIAID AIDSno	413660	7.699
NIAID AIDSno	413661	8.111
NIAID AIDSno	413662	7.699
NIAID AIDSno	413663	7.699
NIAID AIDSno	413664	7.699
NIAID AIDSno	413665	7.849
NIAID AIDSno	413666	7.699
NIAID AIDSno	413667	7.699
NIAID AIDSno	413668	8.199
NIAID AIDSno	413669	7.699
NIAID AIDSno	413670	7.699
NIAID AIDSno	413671	7.699
NIAID AIDSno	413672	7.699

NIAID AIDSno	413673	7.699
NIAID AIDSno	413674	7.699
NIAID AIDSno	413675	7.699
NIAID AIDSno	413676	7.699
NIAID AIDSno	413677	7.699
NIAID AIDSno	413678	7.699
NIAID AIDSno	413679	7.699
NIAID AIDSno	413680	7.699
NIAID AIDSno	413681	7.699
NIAID AIDSno	413682	7.699
NIAID AIDSno	413683	7.699
NIAID AIDSno	413684	7.699
NIAID AIDSno	413685	7.699
NIAID AIDSno	413686	7.699
NIAID AIDSno	413687	7.699
NIAID AIDSno	413688	7.699
NIAID AIDSno	413689	7.699
NIAID AIDSno	413690	7.699
NIAID AIDSno	413691	7.699
NIAID AIDSno	413692	7.699
NIAID AIDSno	413693	7.699
NIAID AIDSno	413694	7.699
NIAID AIDSno	413695	7.699
NIAID AIDSno	413696	7.699
NIAID AIDSno	413697	7.699
NIAID AIDSno	413698	7.699
NIAID AIDSno	413699	7.699
NIAID AIDSno	413700	7.699
NIAID AIDSno	413701	7.699
NIAID AIDSno	413702	7.699
NIAID AIDSno	413703	7.699
NIAID AIDSno	413704	7.699
NIAID AIDSno	413705	7.699
NIAID AIDSno	413706	7.699
NIAID AIDSno	413707	7.699
NIAID AIDSno	413708	7.699
NIAID AIDSno	413709	7.699
NIAID AIDSno	413710	7.699
NIAID AIDSno	413711	7.699
NIAID AIDSno	413713	7.699
NIAID AIDSno	413714	7.699
NIAID AIDSno	413715	7.699
NIAID AIDSno	413716	7.699
NIAID AIDSno	413717	7.699
NIAID AIDSno	413718	7.699

NIAID AIDSno	413719	7.699
NIAID AIDSno	413720	7.699
NIAID AIDSno	413721	7.699
NIAID AIDSno	413722	7.699
NIAID AIDSno	413723	7.699
NIAID AIDSno	413724	7.699
NIAID AIDSno	413725	7.699
NIAID AIDSno	413726	7.699
NIAID AIDSno	413727	7.699
NIAID AIDSno	413728	7.699
NIAID AIDSno	413729	7.699
NIAID AIDSno	413730	7.960
NIAID AIDSno	413731	7.699
NIAID AIDSno	413732	7.699
NIAID AIDSno	413733	7.000
NIAID AIDSno	413734	7.699
NIAID AIDSno	413735	7.699
NIAID AIDSno	413736	7.699
NIAID AIDSno	413737	7.699
NIAID AIDSno	413738	7.699
NIAID AIDSno	413739	7.699
NIAID AIDSno	413740	7.699
NIAID AIDSno	413741	7.699
NIAID AIDSno	413742	7.699
NIAID AIDSno	413743	7.699
NIAID AIDSno	413744	7.699
NIAID AIDSno	413745	7.699
NIAID AIDSno	413746	7.699
NIAID AIDSno	413747	7.699
NIAID AIDSno	413748	7.699
NIAID AIDSno	413749	7.699
NIAID AIDSno	413750	7.699
NIAID AIDSno	413751	7.699
NIAID AIDSno	413752	7.699
NIAID AIDSno	413753	7.699
NIAID AIDSno	413754	7.699
NIAID AIDSno	413755	7.699
NIAID AIDSno	413756	7.699
NIAID AIDSno	413757	7.699
NIAID AIDSno	413758	7.699
NIAID AIDSno	413760	7.000
NIAID AIDSno	413761	7.699
NIAID AIDSno	413762	7.000
NIAID AIDSno	413763	7.699
NIAID AIDSno	413764	7.699

NIAID AIDSno	413765	7.699
NIAID AIDSno	413766	7.699
NIAID AIDSno	413767	7.699
NIAID AIDSno	413768	7.699
NIAID AIDSno	413769	7.699
NIAID AIDSno	413770	7.699
NIAID AIDSno	413771	7.699
NIAID AIDSno	413772	7.699
NIAID AIDSno	413773	7.000
NIAID AIDSno	413775	7.699
NIAID AIDSno	413776	7.699
NIAID AIDSno	413777	7.000
NIAID AIDSno	413778	7.699
NIAID AIDSno	413779	7.699
NIAID AIDSno	413780	7.699
NIAID AIDSno	413781	7.699
NIAID AIDSno	413782	7.699
NIAID AIDSno	413783	7.699
NIAID AIDSno	413784	7.699
NIAID AIDSno	413785	7.699
NIAID AIDSno	413786	7.699
NIAID AIDSno	413787	7.699
NIAID AIDSno	413788	7.000
NIAID AIDSno	413789	7.699
NIAID AIDSno	413790	7.699
NIAID AIDSno	413791	7.699
NIAID AIDSno	413792	7.699
NIAID AIDSno	413793	7.699
NIAID AIDSno	413794	7.699
NIAID AIDSno	413795	7.000
NIAID AIDSno	413796	7.699
NIAID AIDSno	413797	7.699
NIAID AIDSno	413798	7.699
NIAID AIDSno	413799	7.699
NIAID AIDSno	413800	7.699
NIAID AIDSno	413801	7.699
NIAID AIDSno	413802	7.699
NIAID AIDSno	413803	7.699
NIAID AIDSno	413804	7.699
NIAID AIDSno	413805	7.699
NIAID AIDSno	413806	7.699
NIAID AIDSno	413807	7.699
NIAID AIDSno	413808	7.699
NIAID AIDSno	413809	7.699
NIAID AIDSno	413810	7.699

NIAID AIDSno	413811	7.699
NIAID AIDSno	413812	7.699
NIAID AIDSno	413813	7.699
NIAID AIDSno	413814	7.699
NIAID AIDSno	413815	7.699
NIAID AIDSno	413816	7.699
NIAID AIDSno	413817	7.699
NIAID AIDSno	413818	7.699
NIAID AIDSno	413819	7.699
NIAID AIDSno	413820	7.699
NIAID AIDSno	413821	7.699
NIAID AIDSno	413822	7.699
NIAID AIDSno	413823	7.699
NIAID AIDSno	413824	7.699
NIAID AIDSno	413825	7.699
NIAID AIDSno	413826	7.699
NIAID AIDSno	413827	7.699
NIAID AIDSno	413828	7.699
NIAID AIDSno	413829	7.699
NIAID AIDSno	413830	7.699
NIAID AIDSno	413831	7.699
NIAID AIDSno	413832	7.699
NIAID AIDSno	413833	7.699
NIAID AIDSno	413834	7.699
NIAID AIDSno	413835	7.699
NIAID AIDSno	413836	7.699
NIAID AIDSno	413837	7.699
NIAID AIDSno	413839	7.699
NIAID AIDSno	413840	7.699
NIAID AIDSno	413841	7.699
NIAID AIDSno	413842	7.699
NIAID AIDSno	413843	7.699
NIAID AIDSno	413844	7.699
NIAID AIDSno	413845	7.699
NIAID AIDSno	413846	7.699
NIAID AIDSno	413847	7.699
NIAID AIDSno	413848	7.699
NIAID AIDSno	413849	7.699
NIAID AIDSno	413850	7.699
NIAID AIDSno	413851	7.699
NIAID AIDSno	413852	7.699
NIAID AIDSno	413853	7.699
NIAID AIDSno	413854	7.699
NIAID AIDSno	413855	7.699
NIAID AIDSno	413856	7.699

NIAID AIDSno	413857	7.699
NIAID AIDSno	413858	7.699
NIAID AIDSno	413859	7.699
NIAID AIDSno	413860	7.699
NIAID AIDSno	413861	7.699
NIAID AIDSno	413862	7.699
NIAID AIDSno	413863	7.699
NIAID AIDSno	413864	7.699
NIAID AIDSno	413865	7.699
NIAID AIDSno	413866	7.699
NIAID AIDSno	413867	7.699
NIAID AIDSno	413868	7.699
NIAID AIDSno	413869	7.699
NIAID AIDSno	413870	7.699
NIAID AIDSno	413871	7.699
NIAID AIDSno	413872	7.699
NIAID AIDSno	413873	7.699
NIAID AIDSno	413874	7.699
NIAID AIDSno	413875	7.699
NIAID AIDSno	413876	7.000
NIAID AIDSno	413878	7.699
NIAID AIDSno	413879	7.699
NIAID AIDSno	413880	7.000
NIAID AIDSno	413881	7.699
NIAID AIDSno	413882	7.699
NIAID AIDSno	413883	7.699
NIAID AIDSno	413884	7.699
NIAID AIDSno	413885	7.699
NIAID AIDSno	413886	7.699
NIAID AIDSno	413887	7.699
NIAID AIDSno	413888	7.699
NIAID AIDSno	413889	7.699
NIAID AIDSno	413890	7.699
NIAID AIDSno	413891	7.699
NIAID AIDSno	413892	7.699
NIAID AIDSno	413893	7.699
NIAID AIDSno	413894	7.699
NIAID AIDSno	413895	7.699
NIAID AIDSno	413896	7.699
NIAID AIDSno	413897	7.699
NIAID AIDSno	413898	7.699
NIAID AIDSno	413899	7.699
NIAID AIDSno	413900	7.699
NIAID AIDSno	413901	7.699
NIAID AIDSno	413902	7.699

NIAID AIDSno	413903	7.699
NIAID AIDSno	413904	7.699
NIAID AIDSno	413905	7.699
NIAID AIDSno	413906	7.699
NIAID AIDSno	415737	4.481
NIAID AIDSno	415738	3.301
NIAID AIDSno	415739	4.770
NIAID AIDSno	415740	3.301
NIAID AIDSno	415741	3.046
NIAID AIDSno	415743	4.022
NIAID AIDSno	415745	3.478
NIAID AIDSno	415754	4.409
NIAID AIDSno	415756	4.347
NIAID AIDSno	415759	4.495
NIAID AIDSno	415764	4.444
NIAID AIDSno	417214	7.084
NIAID AIDSno	417215	8.000
NIAID AIDSno	417216	7.222
NIAID AIDSno	417217	7.222
NIAID AIDSno	417218	7.155
NIAID AIDSno	417219	7.301
NIAID AIDSno	417220	6.699
NIAID AIDSno	417221	7.097
NIAID AIDSno	417222	6.699
NIAID AIDSno	417223	7.301
NIAID AIDSno	417224	6.699
NIAID AIDSno	417225	7.761
NIAID AIDSno	417226	6.517
NIAID AIDSno	417227	7.602
NIAID AIDSno	424263	7.523
NIAID AIDSno	424265	7.495
NIAID AIDSno	424299	7.796
NIAID AIDSno	424300	8.222
NIAID AIDSno	424301	8.222
NIAID AIDSno	424302	8.301
NIAID AIDSno	424303	8.000
NIAID AIDSno	424308	8.301
NIAID AIDSno	424309	8.222
NIAID AIDSno	424310	8.523
NIAID AIDSno	424311	8.398
NIAID AIDSno	424313	8.222
NIAID AIDSno	424314	7.824
NIAID AIDSno	424315	8.097
NIAID AIDSno	424316	8.046
NIAID AIDSno	424317	7.921

NIAID AIDSno	424318	8.155
NIAID AIDSno	424319	8.046
NIAID AIDSno	424320	8.155
NIAID AIDSno	424321	8.046
NIAID AIDSno	424322	8.097
NIAID AIDSno	424327	7.602
NIAID AIDSno	424329	7.208
NIAID AIDSno	424330	7.886
NIAID AIDSno	424331	8.301
NIAID AIDSno	424332	8.699
NIAID AIDSno	424333	8.699
NIAID AIDSno	424334	8.222
NIAID AIDSno	424337	7.678
NIAID AIDSno	424338	8.097
NIAID AIDSno	424340	7.678
NIAID AIDSno	424343	8.301
NIAID AIDSno	424344	8.301
NIAID AIDSno	424345	8.222
NIAID AIDSno	424346	8.301
NIAID AIDSno	424347	8.222
NIAID AIDSno	424348	8.301
NIAID AIDSno	424349	8.097
NIAID AIDSno	424363	8.155
NIAID AIDSno	424433	7.699
NIAID AIDSno	439641	4.695
NIAID AIDSno	446109	3.478
NIAID AIDSno	446113	3.301
NIAID AIDSno	446114	3.963
NIAID AIDSno	446117	3.046
NIAID AIDSno	446118	4.108
NIAID AIDSno	446119	4.000
NIAID AIDSno	446120	3.301
NIAID AIDSno	446121	3.398
NIAID AIDSno	446122	3.693
NIAID AIDSno	446123	3.573
NIAID AIDSno	446125	3.301
NIAID AIDSno	446127	3.382
NIAID AIDSno	446131	4.208
NIAID AIDSno	446132	4.187
NIAID AIDSno	446135	4.569
NIAID AIDSno	446136	5.155
NIAID AIDSno	446137	4.638
NIAID AIDSno	446138	5.523
NIAID AIDSno	446139	5.569
NIAID AIDSno	446140	5.319

NIAID AIDSno	446141	6.420
NIAID AIDSno	446142	4.886
NIAID AIDSno	446143	5.770
NIAID AIDSno	446144	6.276
NIAID AIDSno	446145	4.456
NIAID AIDSno	446146	4.796
NIAID AIDSno	446177	8.000
NIAID AIDSno	446178	7.523
NIAID AIDSno	446179	7.347
NIAID AIDSno	446180	8.000
NIAID AIDSno	446181	7.292
NIAID AIDSno	446182	7.854
NIAID AIDSno	446183	8.155
NIAID AIDSno	446190	5.018
NIAID AIDSno	446191	4.754
NIAID AIDSno	446192	4.355
NIAID AIDSno	446193	4.359
NIAID AIDSno	446194	4.039
NIAID AIDSno	446195	4.440
NIAID AIDSno	446196	3.880
NIAID AIDSno	446197	4.011
NIAID AIDSno	446198	3.551
NIAID AIDSno	471309	7.411
NIAID AIDSno	472640	4.357
NIAID AIDSno	472642	4.745
NIAID AIDSno	472755	4.979
NIAID AIDSno	472756	3.892
NIAID AIDSno	472757	3.765
NIAID AIDSno	472758	3.829
NIAID AIDSno	472759	3.887
NIAID AIDSno	472760	4.022
NIAID AIDSno	472761	3.889
NIAID AIDSno	472762	4.921
NIAID AIDSno	472763	6.000
NIAID AIDSno	472764	4.301
NIAID AIDSno	472765	4.301
NIAID AIDSno	472766	5.801
NIAID AIDSno	472768	7.699
NIAID AIDSno	472769	7.000
NIAID AIDSno	472770	7.699
NIAID AIDSno	472771	7.699
NIAID AIDSno	472772	7.301
NIAID AIDSno	472773	7.155
NIAID AIDSno	472774	6.824
NIAID AIDSno	472775	6.824

NIAID AIDSno	472776	6.824
NIAID AIDSno	472777	7.301
NIAID AIDSno	472778	7.000
NIAID AIDSno	472779	6.943
NIAID AIDSno	472780	6.097
NIAID AIDSno	472781	5.910
NIAID AIDSno	472782	7.000
NIAID AIDSno	472783	6.699
NIAID AIDSno	472784	6.638
NIAID AIDSno	472785	6.921
NIAID AIDSno	472786	8.000
NIAID AIDSno	472787	7.301
NIAID AIDSno	472788	5.699
NIAID AIDSno	472789	7.222
NIAID AIDSno	472790	7.398
NIAID AIDSno	472791	8.097
NIAID AIDSno	472792	8.000
NIAID AIDSno	472793	7.284
NIAID AIDSno	472794	7.056
NIAID AIDSno	472795	7.097
NIAID AIDSno	472796	7.585
NIAID AIDSno	473857	4.886
NIAID AIDSno	476009	5.987
NIAID AIDSno	476010	6.420
NIAID AIDSno	476013	3.558
NIAID AIDSno	476014	4.312
NIAID AIDSno	476015	4.042
NIAID AIDSno	476016	4.330
NIAID AIDSno	476017	5.398
NIAID AIDSno	476018	4.096
NIAID AIDSno	476020	4.161
NIAID AIDSno	476022	3.896
NIAID AIDSno	476023	7.000
NIAID AIDSno	476024	7.000
NIAID AIDSno	476025	5.389
NIAID AIDSno	476026	5.523
NIAID AIDSno	476055	4.721
NIAID AIDSno	476056	5.102
NIAID AIDSno	476057	5.000
NIAID AIDSno	476093	6.155
NIAID AIDSno	476094	6.523
NIAID AIDSno	476095	5.745
NIAID AIDSno	476096	5.921
NIAID AIDSno	476097	5.770
NIAID AIDSno	476099	6.155

NIAID AIDSno	476100	6.523
NIAID AIDSno	476101	6.699
NIAID AIDSno	478200	4.638
NIAID AIDSno	478201	4.620
NIAID AIDSno	478202	5.357
NIAID AIDSno	478204	4.602
NIAID AIDSno	478205	4.321
NIAID AIDSno	478206	4.917
NIAID AIDSno	478207	4.602
NIAID AIDSno	478209	5.959
NIAID AIDSno	478215	3.903
NIAID AIDSno	478218	4.602
NIAID AIDSno	478219	4.602
NIAID AIDSno	479612	7.699
NIAID AIDSno	479613	7.854
NIAID AIDSno	479614	7.398
NIAID AIDSno	479615	7.620
NIAID AIDSno	479616	7.481
NIAID AIDSno	479617	6.910
NIAID AIDSno	479618	7.585
NIAID AIDSno	479644	7.553
NIAID AIDSno	479878	4.569
NIAID AIDSno	479881	4.398
NIAID AIDSno	479887	4.292
NIAID AIDSno	479888	4.602
NIAID AIDSno	479889	4.602
NIAID AIDSno	479890	4.553
NIAID AIDSno	479963	6.143
NIAID AIDSno	479964	5.646
NIAID AIDSno	479965	7.921
NIAID AIDSno	479966	6.174
NIAID AIDSno	479967	6.398
NIAID AIDSno	479968	7.155
NIAID AIDSno	479969	5.807
NIAID AIDSno	479970	5.562
NIAID AIDSno	479971	5.939
NIAID AIDSno	479972	6.086
NIAID AIDSno	479973	5.851
NIAID AIDSno	479974	7.398
NIAID AIDSno	479975	7.523
NIAID AIDSno	479976	6.921
NIAID AIDSno	479977	7.699
NIAID AIDSno	479978	7.699
NIAID AIDSno	479979	7.097
NIAID AIDSno	479980	6.222

NIAID AIDSno	479981	6.620
NIAID AIDSno	479982	6.553
NIAID AIDSno	479983	6.796
NIAID AIDSno	479984	7.000
NIAID AIDSno	479985	6.854
NIAID AIDSno	480452	4.886
NIAID AIDSno	480453	5.155
NIAID AIDSno	480454	5.155
NIAID AIDSno	480455	5.398
NIAID AIDSno	480456	4.959
NIAID AIDSno	480457	4.959
NIAID AIDSno	480458	5.155
NIAID AIDSno	480459	4.481
NIAID AIDSno	480460	4.959
NIAID AIDSno	480461	5.699
NIAID AIDSno	480462	5.000
NIAID AIDSno	480463	4.886
NIAID AIDSno	480464	4.921
NIAID AIDSno	480465	4.721
NIAID AIDSno	480468	4.824
NIAID AIDSno	480609	6.022
NIAID AIDSno	480613	6.886
NIAID AIDSno	480614	6.444
NIAID AIDSno	480617	6.886
NIAID AIDSno	480621	6.194
NIAID AIDSno	480625	5.987
NIAID AIDSno	480631	5.553
NIAID AIDSno	480635	5.201
NIAID AIDSno	480642	5.620
NIAID AIDSno	480647	5.337
NIAID AIDSno	480653	5.456
NIAID AIDSno	480660	6.886
NIAID AIDSno	480661	7.377
NIAID AIDSno	480663	5.102
NIAID AIDSno	480733	8.699
NIAID AIDSno	480974	3.495
NIAID AIDSno	480977	5.222
NIAID AIDSno	480979	6.046
NIAID AIDSno	480981	4.854
NIAID AIDSno	480982	5.301
NIAID AIDSno	480984	3.959
NIAID AIDSno	480986	3.658
NIAID AIDSno	481376	8.097
NIAID AIDSno	481378	8.523
NIAID AIDSno	481379	7.886

NIAID AIDSno	481381	7.658
NIAID AIDSno	481383	7.824
NIAID AIDSno	481386	8.222
NIAID AIDSno	481388	7.347
NIAID AIDSno	481390	8.222
NIAID AIDSno	481391	8.000
NIAID AIDSno	481395	8.222
NIAID AIDSno	481396	8.387
NIAID AIDSno	481399	8.398
NIAID AIDSno	481403	8.301
NIAID AIDSno	481406	8.155
NIAID AIDSno	481407	8.398
NIAID AIDSno	481408	6.370
NIAID AIDSno	481525	4.035
NIAID AIDSno	481526	4.139
NIAID AIDSno	481733	6.921
NIAID AIDSno	481734	7.284
NIAID AIDSno	481735	7.215
NIAID AIDSno	481736	7.409
NIAID AIDSno	481737	6.870
NIAID AIDSno	481738	7.131
NIAID AIDSno	481740	8.301
NIAID AIDSno	482051	7.886
NIAID AIDSno	482116	8.155
NIAID AIDSno	482165	8.155
NIAID AIDSno	482166	8.155
NIAID AIDSno	482167	7.161
NIAID AIDSno	482169	6.260
NIAID AIDSno	482170	7.201
NIAID AIDSno	482171	8.398
NIAID AIDSno	482172	8.301
NIAID AIDSno	482173	7.208
NIAID AIDSno	482174	8.398
NIAID AIDSno	482175	7.208
NIAID AIDSno	482176	8.097
NIAID AIDSno	482177	8.301
NIAID AIDSno	482178	7.721
NIAID AIDSno	482311	7.959
NIAID AIDSno	482312	8.046
NIAID AIDSno	482313	8.222
NIAID AIDSno	482314	8.097
NIAID AIDSno	482316	7.745
NIAID AIDSno	482319	8.523
NIAID AIDSno	482331	8.108
NIAID AIDSno	482335	8.000

NIAID AIDSno	482337	8.222
NIAID AIDSno	482338	7.770
NIAID AIDSno	482339	8.155
NIAID AIDSno	482340	7.921
NIAID AIDSno	482342	7.796
NIAID AIDSno	482343	8.155
NIAID AIDSno	482344	8.155
NIAID AIDSno	482345	8.301
NIAID AIDSno	482346	8.000
NIAID AIDSno	482347	7.854
NIAID AIDSno	482348	8.222
NIAID AIDSno	482443	7.000
NIAID AIDSno	482444	7.854
NIAID AIDSno	482446	6.222
NIAID AIDSno	482448	7.097
NIAID AIDSno	482449	6.577
NIAID AIDSno	482656	8.176
NIAID AIDSno	482859	7.000
NIAID AIDSno	482886	7.149
NIAID AIDSno	482888	7.208
NIAID AIDSno	482891	6.328
NIAID AIDSno	482892	6.577
NIAID AIDSno	482895	7.886
NIAID AIDSno	482896	7.886
NIAID AIDSno	482899	7.398
NIAID AIDSno	482900	6.959
NIAID AIDSno	482913	7.699
NIAID AIDSno	482915	7.398
NIAID AIDSno	482916	6.886
NIAID AIDSno	482929	7.169
NIAID AIDSno	483245	5.573
NIAID AIDSno	483247	5.520
NIAID AIDSno	483254	5.335
NIAID AIDSno	483259	3.975
NIAID AIDSno	483260	3.699
NIAID AIDSno	483265	6.620
NIAID AIDSno	483282	6.602
NIAID AIDSno	483284	7.638
NIAID AIDSno	483294	6.260
NIAID AIDSno	483297	7.161
NIAID AIDSno	483300	7.222
NIAID AIDSno	483301	7.658
NIAID AIDSno	483302	6.585
NIAID AIDSno	483311	6.745
NIAID AIDSno	483313	4.301

NIAID AIDSno	483317	7.699
NIAID AIDSno	483324	6.208
NIAID AIDSno	483327	5.658
NIAID AIDSno	483330	5.502
NIAID AIDSno	483854	3.914
NIAID AIDSno	483858	4.432
NIAID AIDSno	483860	4.398
NIAID AIDSno	483861	6.658
NIAID AIDSno	483863	5.114
NIAID AIDSno	483866	4.699
NIAID AIDSno	483867	4.252
NIAID AIDSno	483868	6.252
NIAID AIDSno	483869	4.018
NIAID AIDSno	483872	3.967
NIAID AIDSno	483879	6.658
NIAID AIDSno	483882	7.569
NIAID AIDSno	483889	6.155
NIAID AIDSno	483891	5.770
NIAID AIDSno	483894	6.638
NIAID AIDSno	483901	5.770
NIAID AIDSno	483908	5.187
NIAID AIDSno	483926	6.658
NIAID AIDSno	483936	5.745
NIAID AIDSno	483943	5.721
NIAID AIDSno	483945	6.658
NIAID AIDSno	483954	6.222
NIAID AIDSno	483957	5.620
NIAID AIDSno	483959	6.180
NIAID AIDSno	483978	6.444
NIAID AIDSno	483981	6.721
NIAID AIDSno	484016	5.041
NIAID AIDSno	484063	8.155
NIAID AIDSno	484065	8.046
NIAID AIDSno	484066	7.959
NIAID AIDSno	484068	7.699
NIAID AIDSno	484070	7.921
NIAID AIDSno	484072	5.638
NIAID AIDSno	484075	5.959
NIAID AIDSno	484077	5.092
NIAID AIDSno	484098	4.620
NIAID AIDSno	484099	7.301
NIAID AIDSno	484112	5.208
NIAID AIDSno	484114	5.131
NIAID AIDSno	484142	5.745
NIAID AIDSno	484144	4.337

NIAID AIDSno	484321	6.301
NIAID AIDSno	484588	5.548
NIAID AIDSno	484590	4.701
NIAID AIDSno	484593	4.645
NIAID AIDSno	484598	3.951
NIAID AIDSno	484602	4.301
NIAID AIDSno	484603	4.509
NIAID AIDSno	484606	4.405
NIAID AIDSno	484610	3.955
NIAID AIDSno	484611	5.077
NIAID AIDSno	484615	4.456
NIAID AIDSno	484618	4.284
NIAID AIDSno	484621	5.097
NIAID AIDSno	484640	3.889
NIAID AIDSno	484644	3.533
NIAID AIDSno	484647	5.222
NIAID AIDSno	484650	3.770
NIAID AIDSno	484652	4.678
NIAID AIDSno	484654	4.481
NIAID AIDSno	484655	6.678
NIAID AIDSno	484657	4.387
NIAID AIDSno	484660	5.921
NIAID AIDSno	484671	4.959
NIAID AIDSno	484673	6.222
NIAID AIDSno	484675	6.092
NIAID AIDSno	484678	4.276
NIAID AIDSno	484680	4.268
NIAID AIDSno	484686	4.910
NIAID AIDSno	484687	4.553
NIAID AIDSno	484689	6.131
NIAID AIDSno	484690	5.000
NIAID AIDSno	484691	6.796
NIAID AIDSno	484692	4.886
NIAID AIDSno	484695	3.764
NIAID AIDSno	484879	4.261
NIAID AIDSno	485275	5.432
NIAID AIDSno	485277	7.155
NIAID AIDSno	485320	5.631
NIAID AIDSno	485325	5.578
NIAID AIDSno	485332	4.799
NIAID AIDSno	485336	6.180
NIAID AIDSno	485361	4.447
NIAID AIDSno	485376	4.910
NIAID AIDSno	485413	6.658
NIAID AIDSno	485415	6.721

NIAID AIDSno	485416	7.523
NIAID AIDSno	485417	5.260
NIAID AIDSno	485418	7.824
NIAID AIDSno	485419	7.854
NIAID AIDSno	485422	7.933
NIAID AIDSno	485458	4.638
NIAID AIDSno	485676	5.562
NIAID AIDSno	485678	4.276
NIAID AIDSno	485679	4.327
NIAID AIDSno	485681	4.452
NIAID AIDSno	485686	5.237
NIAID AIDSno	485688	4.595
NIAID AIDSno	485692	4.389
NIAID AIDSno	485694	4.145
NIAID AIDSno	485697	3.903
NIAID AIDSno	485711	4.652
NIAID AIDSno	485714	3.871
NIAID AIDSno	485718	4.131
NIAID AIDSno	485921	7.000
NIAID AIDSno	485922	8.222
NIAID AIDSno	485923	8.398
NIAID AIDSno	485924	8.301
NIAID AIDSno	485925	7.027
NIAID AIDSno	485932	7.745
NIAID AIDSno	485952	5.284
NIAID AIDSno	485953	4.237
NIAID AIDSno	485954	5.523
NIAID AIDSno	485956	5.444
NIAID AIDSno	485958	5.284
NIAID AIDSno	485960	5.745
NIAID AIDSno	485962	5.603
NIAID AIDSno	485965	6.854
NIAID AIDSno	485968	7.301
NIAID AIDSno	485969	4.000
NIAID AIDSno	485971	6.796
NIAID AIDSno	485973	6.398
NIAID AIDSno	485974	6.959
NIAID AIDSno	485976	6.292
NIAID AIDSno	485978	7.125
NIAID AIDSno	485979	6.222
NIAID AIDSno	485980	7.149
NIAID AIDSno	485982	5.328
NIAID AIDSno	485983	5.678
NIAID AIDSno	485985	3.650
NIAID AIDSno	485986	4.481

NIAID AIDSno	485987	4.252
NIAID AIDSno	485989	4.770
NIAID AIDSno	485990	4.347
NIAID AIDSno	485996	5.155
NIAID AIDSno	486000	5.745
NIAID AIDSno	486002	5.523
NIAID AIDSno	486003	4.260
NIAID AIDSno	486004	4.450
NIAID AIDSno	486005	4.523
NIAID AIDSno	486006	4.387
NIAID AIDSno	486010	4.585
NIAID AIDSno	486011	4.056
NIAID AIDSno	486012	4.509
NIAID AIDSno	486014	4.276
NIAID AIDSno	486018	4.538
NIAID AIDSno	486023	4.602
NIAID AIDSno	486026	4.585
NIAID AIDSno	486356	5.523
NIAID AIDSno	486360	4.174
NIAID AIDSno	486362	5.222
NIAID AIDSno	486364	4.398
NIAID AIDSno	486365	5.046
NIAID AIDSno	486366	4.377
NIAID AIDSno	486368	5.046
NIAID AIDSno	486369	4.585
NIAID AIDSno	486370	4.523
NIAID AIDSno	486371	5.097
NIAID AIDSno	486374	4.770
NIAID AIDSno	486376	4.367
NIAID AIDSno	486383	4.770
NIAID AIDSno	486385	4.409
NIAID AIDSno	486387	4.108
NIAID AIDSno	486389	4.678
NIAID AIDSno	486390	4.481
NIAID AIDSno	486392	5.097
NIAID AIDSno	486394	5.301
NIAID AIDSno	486395	4.678
NIAID AIDSno	486396	4.046
NIAID AIDSno	486397	4.678
NIAID AIDSno	486398	4.081
NIAID AIDSno	486399	4.921
NIAID AIDSno	486400	4.509
NIAID AIDSno	486597	4.167
NIAID AIDSno	486598	4.013
NIAID AIDSno	486599	4.569

NIAID AIDSno	486602	4.092
NIAID AIDSno	486603	4.000
NIAID AIDSno	486625	4.733
NIAID AIDSno	486626	6.699
NIAID AIDSno	486628	5.886
NIAID AIDSno	486629	6.222
NIAID AIDSno	486630	4.618
NIAID AIDSno	486633	4.783
NIAID AIDSno	486653	5.678
NIAID AIDSno	486654	5.796
NIAID AIDSno	487161	4.237
NIAID AIDSno	487171	4.569
NIAID AIDSno	487540	4.959
NIAID AIDSno	487546	4.638
NIAID AIDSno	487560	4.409
NIAID AIDSno	487571	4.000
NIAID AIDSno	487618	4.276
NIAID AIDSno	487619	4.456
NIAID AIDSno	487620	4.092
NIAID AIDSno	487621	4.409
NIAID AIDSno	487624	4.377
NIAID AIDSno	487625	4.509
NIAID AIDSno	487626	4.538
NIAID AIDSno	487627	4.276
NIAID AIDSno	487628	5.398
NIAID AIDSno	487803	5.222
NIAID AIDSno	487805	5.398
NIAID AIDSno	487814	5.222
NIAID AIDSno	487815	4.081
NIAID AIDSno	487816	4.481
NIAID AIDSno	487818	4.444
NIAID AIDSno	487819	4.114
NIAID AIDSno	487820	4.032
NIAID AIDSno	487821	4.284
NIAID AIDSno	487822	4.108
NIAID AIDSno	487823	4.155
NIAID AIDSno	487824	4.046
NIAID AIDSno	488007	6.100
NIAID AIDSno	488012	4.646
NIAID AIDSno	488015	6.409
NIAID AIDSno	488020	5.301
NIAID AIDSno	488026	6.143
NIAID AIDSno	488032	6.018
NIAID AIDSno	488035	5.161
NIAID AIDSno	488036	5.398

NIAID AIDSno	488037	4.004
NIAID AIDSno	488038	4.481
NIAID AIDSno	488039	4.155
NIAID AIDSno	488040	4.765
NIAID AIDSno	488041	4.857
NIAID AIDSno	488042	4.979
NIAID AIDSno	488059	4.928
NIAID AIDSno	488062	4.093
NIAID AIDSno	488994	4.215
NIAID AIDSno	489342	4.678
NIAID AIDSno	489343	4.641
NIAID AIDSno	489344	5.441
NIAID AIDSno	489345	5.502
NIAID AIDSno	489346	5.387
NIAID AIDSno	489347	6.043
NIAID AIDSno	489348	6.502
NIAID AIDSno	489349	4.572
NIAID AIDSno	489350	6.155
NIAID AIDSno	489351	6.301
NIAID AIDSno	489352	4.704
NIAID AIDSno	489353	6.000
NIAID AIDSno	489354	6.046
NIAID AIDSno	489355	5.222
NIAID AIDSno	489356	7.000
NIAID AIDSno	489357	5.602
NIAID AIDSno	489358	5.921
NIAID AIDSno	489359	5.734
NIAID AIDSno	489360	4.745
NIAID AIDSno	489361	5.398
NIAID AIDSno	489362	5.569
NIAID AIDSno	489363	5.432
NIAID AIDSno	489364	4.602
NIAID AIDSno	489365	5.051
NIAID AIDSno	489366	5.357
NIAID AIDSno	489367	5.097
NIAID AIDSno	489368	6.523
NIAID AIDSno	489369	5.699
NIAID AIDSno	489370	6.409
NIAID AIDSno	489371	6.377
NIAID AIDSno	489372	5.979
NIAID AIDSno	489373	6.813
NIAID AIDSno	489375	6.638
NIAID AIDSno	489376	6.699
NIAID AIDSno	489377	6.523
NIAID AIDSno	489379	5.688

NIAID AIDSno	489380	6.770
NIAID AIDSno	489381	6.046
NIAID AIDSno	489382	6.097
NIAID AIDSno	489383	6.046
NIAID AIDSno	489384	6.921
NIAID AIDSno	489385	5.886
NIAID AIDSno	489386	6.509
NIAID AIDSno	489388	5.734
NIAID AIDSno	489389	5.456
NIAID AIDSno	489390	6.222
NIAID AIDSno	489391	6.097
NIAID AIDSno	489392	6.301
NIAID AIDSno	489393	5.745
NIAID AIDSno	489395	5.398
NIAID AIDSno	489396	6.398
NIAID AIDSno	489397	5.886
NIAID AIDSno	489407	8.301
NIAID AIDSno	489418	7.208
NIAID AIDSno	489420	7.119
NIAID AIDSno	489422	8.523
NIAID AIDSno	489424	8.155
NIAID AIDSno	489426	8.155
NIAID AIDSno	489428	7.678
NIAID AIDSno	495016	5.699
NIAID AIDSno	495018	5.699
NIAID AIDSno	495025	5.097
NIAID AIDSno	495028	5.347
NIAID AIDSno	495031	5.523
NIAID AIDSno	495033	5.602
NIAID AIDSno	495034	5.699
NIAID AIDSno	495036	5.886
NIAID AIDSno	495038	5.699
NIAID AIDSno	495042	6.046
NIAID AIDSno	495043	5.824
NIAID AIDSno	495048	5.770
NIAID AIDSno	495050	5.824
NIAID AIDSno	495052	4.301
NIAID AIDSno	495054	4.921
NIAID AIDSno	495469	7.097
NIAID AIDSno	495470	6.921
NIAID AIDSno	495472	7.046
NIAID AIDSno	495475	7.699
NIAID AIDSno	495477	7.398
NIAID AIDSno	495479	7.699
NIAID AIDSno	495480	6.921

NIAID AIDSno	495481	7.523
NIAID AIDSno	496222	6.602
NIAID AIDSno	496223	7.097
NIAID AIDSno	496224	6.569
NIAID AIDSno	496225	7.699
NIAID AIDSno	496227	7.301
NIAID AIDSno	496228	6.260
NIAID AIDSno	496230	6.357
NIAID AIDSno	496232	7.000
NIAID AIDSno	496233	7.523
NIAID AIDSno	496234	7.000
NIAID AIDSno	496235	7.222
NIAID AIDSno	496236	8.398
NIAID AIDSno	496237	8.222
NIAID AIDSno	496238	7.699
NIAID AIDSno	496239	7.699
NIAID AIDSno	496243	7.699
NIAID AIDSno	496248	7.523
NIAID AIDSno	496551	4.420
NIAID AIDSno	496554	4.886
NIAID AIDSno	496556	4.013
NIAID AIDSno	496557	4.569
NIAID AIDSno	496568	4.000
NIAID AIDSno	496580	5.097
NIAID AIDSno	496586	4.137
NIAID AIDSno	496593	4.553
NIAID AIDSno	496598	4.921
NIAID AIDSno	496600	4.009
NIAID AIDSno	496602	4.229
NIAID AIDSno	496604	4.022
NIAID AIDSno	496606	4.131
NIAID AIDSno	496607	4.276
NIAID AIDSno	496608	4.036
NIAID AIDSno	496609	4.046
NIAID AIDSno	496610	4.699
NIAID AIDSno	496611	5.398
NIAID AIDSno	496612	5.398
NIAID AIDSno	496613	5.046
NIAID AIDSno	496614	4.167
NIAID AIDSno	496615	4.174
NIAID AIDSno	496616	4.699
NIAID AIDSno	496617	4.721
NIAID AIDSno	496621	4.538
NIAID AIDSno	496622	4.046
NIAID AIDSno	496624	4.509

NIAID AIDSno	496628	4.854
NIAID AIDSno	496629	4.328
NIAID AIDSno	496630	4.149
NIAID AIDSno	496631	4.022
NIAID AIDSno	496632	4.854
NIAID AIDSno	496744	4.796
NIAID AIDSno	496747	4.022
NIAID AIDSno	496750	4.000
NIAID AIDSno	496753	4.155
NIAID AIDSno	496760	5.398
NIAID AIDSno	496762	4.699
NIAID AIDSno	496764	4.167
NIAID AIDSno	496767	4.409
NIAID AIDSno	496774	4.018
NIAID AIDSno	496789	4.620
NIAID AIDSno	496792	4.585
NIAID AIDSno	496797	4.056
NIAID AIDSno	496819	4.337
NIAID AIDSno	496824	4.252
NIAID AIDSno	496829	4.310
NIAID AIDSno	496832	4.000
NIAID AIDSno	496834	4.056
NIAID AIDSno	496840	4.022
NIAID AIDSno	496851	4.066
NIAID AIDSno	496852	4.620
NIAID AIDSno	496853	4.046
NIAID AIDSno	496854	5.187
NIAID AIDSno	496856	4.824
NIAID AIDSno	496947	4.301
NIAID AIDSno	496949	4.208
NIAID AIDSno	496955	1.750
NIAID AIDSno	496957	4.854
NIAID AIDSno	496959	4.523
NIAID AIDSno	496967	4.292
NIAID AIDSno	496971	5.187
NIAID AIDSno	496975	5.155
NIAID AIDSno	496977	5.469
NIAID AIDSno	496982	4.770
NIAID AIDSno	497020	4.398
NIAID AIDSno	497024	4.377
NIAID AIDSno	497052	5.523
NIAID AIDSno	497054	5.046
NIAID AIDSno	497055	4.921
NIAID AIDSno	497057	5.097
NIAID AIDSno	497058	4.237

NIAID AIDSno	497060	4.770
NIAID AIDSno	497062	4.569
NIAID AIDSno	497064	4.770
NIAID AIDSno	497070	5.222
NIAID AIDSno	497073	4.509
NIAID AIDSno	497075	4.215
NIAID AIDSno	497077	5.301
NIAID AIDSno	497078	4.678
NIAID AIDSno	497079	5.046
NIAID AIDSno	497138	5.301
NIAID AIDSno	497139	4.222
NIAID AIDSno	497145	5.301
NIAID AIDSno	497146	4.367
NIAID AIDSno	497147	4.284
NIAID AIDSno	497148	4.337
NIAID AIDSno	497149	4.745
NIAID AIDSno	497150	4.638
NIAID AIDSno	497151	4.180
NIAID AIDSno	497152	4.469
NIAID AIDSno	497153	4.770
NIAID AIDSno	497154	5.301
NIAID AIDSno	497155	5.398
NIAID AIDSno	497156	5.398
NIAID AIDSno	497157	4.229
NIAID AIDSno	497158	4.886
NIAID AIDSno	497159	4.187
NIAID AIDSno	497375	3.401
NIAID AIDSno	497389	3.558
NIAID AIDSno	497391	3.963
NIAID AIDSno	497402	4.060
NIAID AIDSno	497498	4.000
NIAID AIDSno	497499	4.000
NIAID AIDSno	497503	4.000
NIAID AIDSno	497508	4.000
NIAID AIDSno	497514	4.000
NIAID AIDSno	497519	4.125
NIAID AIDSno	497522	4.000
NIAID AIDSno	497523	4.456
NIAID AIDSno	497524	4.456
NIAID AIDSno	497525	4.060
NIAID AIDSno	497526	4.237
NIAID AIDSno	497528	4.000
NIAID AIDSno	497529	4.796
NIAID AIDSno	497530	4.000
NIAID AIDSno	497531	4.770

NIAID AIDSno	497532	4.000
NIAID AIDSno	497534	5.456
NIAID AIDSno	497535	5.134
NIAID AIDSno	497536	5.433
NIAID AIDSno	497537	6.022
NIAID AIDSno	497538	5.924
NIAID AIDSno	497539	5.266
NIAID AIDSno	497543	5.495
NIAID AIDSno	497652	6.863
NIAID AIDSno	497653	6.991
NIAID AIDSno	497654	6.883
NIAID AIDSno	497655	6.355
NIAID AIDSno	497656	6.412
NIAID AIDSno	497657	6.108
NIAID AIDSno	497658	5.242
NIAID AIDSno	497659	6.090
NIAID AIDSno	497660	5.790
NIAID AIDSno	497662	5.886
NIAID AIDSno	497663	6.216
NIAID AIDSno	497664	5.764
NIAID AIDSno	497666	6.602
NIAID AIDSno	497667	6.449
NIAID AIDSno	497669	6.602
NIAID AIDSno	497670	4.638
NIAID AIDSno	499984	4.427
NIAID AIDSno	502863	4.237
NIAID AIDSno	502864	5.523
NIAID AIDSno	502865	5.046
NIAID AIDSno	502866	5.260
NIAID AIDSno	505079	4.167
NIAID AIDSno	505187	6.770
NIAID AIDSno	505257	4.481
NIAID AIDSno	505259	4.699
NIAID AIDSno	505260	4.032
NIAID AIDSno	505261	4.387
NIAID AIDSno	505262	4.237
NIAID AIDSno	505263	4.252
NIAID AIDSno	505264	4.131
NIAID AIDSno	505265	4.222
NIAID AIDSno	505266	4.854
NIAID AIDSno	505267	4.602
NIAID AIDSno	505268	4.071
NIAID AIDSno	505292	4.046
NIAID AIDSno	505297	4.699
NIAID AIDSno	505298	4.523

NIAID AIDSno	505299	4.143
NIAID AIDSno	505304	4.081
NIAID AIDSno	505307	4.585
NIAID AIDSno	505309	4.215
NIAID AIDSno	505311	4.456
NIAID AIDSno	505312	4.495
NIAID AIDSno	505313	4.509
NIAID AIDSno	505314	4.022
NIAID AIDSno	505316	4.174
NIAID AIDSno	505318	4.056
NIAID AIDSno	505319	4.347
NIAID AIDSno	505321	4.000
NIAID AIDSno	505323	5.097
NIAID AIDSno	505324	4.347
NIAID AIDSno	505357	4.260
NIAID AIDSno	505359	4.678
NIAID AIDSno	505360	4.638
NIAID AIDSno	505361	5.523
NIAID AIDSno	505384	7.381
NIAID AIDSno	505387	7.699
NIAID AIDSno	505916	6.231
NIAID AIDSno	506125	5.222
NIAID AIDSno	506127	4.886
NIAID AIDSno	506128	5.000
NIAID AIDSno	506129	4.921
NIAID AIDSno	506130	5.000
NIAID AIDSno	506204	7.377
NIAID AIDSno	506223	7.824
NIAID AIDSno	506228	7.155
NIAID AIDSno	506229	7.420
NIAID AIDSno	506231	7.585
NIAID AIDSno	506235	7.721
NIAID AIDSno	506241	7.328
NIAID AIDSno	506242	7.046
NIAID AIDSno	506244	7.602
NIAID AIDSno	506251	7.721
NIAID AIDSno	506359	7.115
NIAID AIDSno	506471	4.907
NIAID AIDSno	506472	4.758
NIAID AIDSno	506473	4.603
NIAID AIDSno	506475	4.715
NIAID AIDSno	506476	4.504
NIAID AIDSno	506477	4.812
NIAID AIDSno	506529	7.201
NIAID AIDSno	506530	4.717

NIAID AIDSno	506761	4.398
NIAID AIDSno	506834	4.478
NIAID AIDSno	506948	4.694
NIAID AIDSno	506995	4.824
NIAID AIDSno	506996	5.620
NIAID AIDSno	506997	6.352
NIAID AIDSno	507004	4.745
NIAID AIDSno	507005	5.108
NIAID AIDSno	507006	6.604
NIAID AIDSno	507012	6.203
NIAID AIDSno	507052	6.770
NIAID AIDSno	507053	6.611
NIAID AIDSno	507054	6.368
NIAID AIDSno	507055	6.561
NIAID AIDSno	507056	6.122
NIAID AIDSno	507113	7.301
NIAID AIDSno	507115	5.788
NIAID AIDSno	507116	5.638
NIAID AIDSno	507118	6.097
NIAID AIDSno	507119	6.387
NIAID AIDSno	507121	6.301
NIAID AIDSno	507122	5.967
NIAID AIDSno	507124	5.932
NIAID AIDSno	507125	6.143
NIAID AIDSno	507127	6.432
NIAID AIDSno	507128	6.602
NIAID AIDSno	507129	7.155
NIAID AIDSno	507142	7.921
NIAID AIDSno	507145	8.086
NIAID AIDSno	507149	6.509
NIAID AIDSno	507151	6.347
NIAID AIDSno	507154	6.648
NIAID AIDSno	507157	7.229
NIAID AIDSno	507158	7.347
NIAID AIDSno	507166	8.222
NIAID AIDSno	507167	8.009
NIAID AIDSno	507176	7.071
NIAID AIDSno	507227	6.770
NIAID AIDSno	507231	7.022
NIAID AIDSno	507232	7.155
NIAID AIDSno	507234	7.420
NIAID AIDSno	507236	7.328
NIAID AIDSno	507247	6.886
NIAID AIDSno	507248	6.870
NIAID AIDSno	507249	6.710

NIAID AIDSno	507250	6.959
NIAID AIDSno	507287	4.865
NIAID AIDSno	507289	5.186
NIAID AIDSno	507290	5.947
NIAID AIDSno	507292	5.292
NIAID AIDSno	507294	6.886
NIAID AIDSno	507899	4.456
NIAID AIDSno	508078	6.092
NIAID AIDSno	508079	6.456
NIAID AIDSno	508080	5.860
NIAID AIDSno	508081	5.593
NIAID AIDSno	508082	6.444
NIAID AIDSno	508085	6.886
NIAID AIDSno	508087	6.201
NIAID AIDSno	508092	4.325
NIAID AIDSno	508093	4.130
NIAID AIDSno	508094	5.896
NIAID AIDSno	508095	5.216
NIAID AIDSno	508096	6.569
NIAID AIDSno	508112	6.357
NIAID AIDSno	508113	6.678
NIAID AIDSno	508114	6.921
NIAID AIDSno	508503	8.398
NIAID AIDSno	508505	8.398
NIAID AIDSno	508506	7.921
NIAID AIDSno	508507	8.000
NIAID AIDSno	508508	8.222
NIAID AIDSno	508509	8.046
NIAID AIDSno	508511	8.046
NIAID AIDSno	508512	8.000
NIAID AIDSno	508513	7.745
NIAID AIDSno	508514	8.222
NIAID AIDSno	508515	8.523
NIAID AIDSno	508518	8.222
NIAID AIDSno	508519	8.460
NIAID AIDSno	508520	8.301
NIAID AIDSno	508521	8.222
NIAID AIDSno	508522	8.097
NIAID AIDSno	508523	7.886
NIAID AIDSno	508524	8.046
NIAID AIDSno	508525	8.097
NIAID AIDSno	508526	7.921
NIAID AIDSno	508783	4.469
NIAID AIDSno	508785	4.824
NIAID AIDSno	508786	4.523

NIAID AIDSno	508787	4.398
NIAID AIDSno	508788	4.658
NIAID AIDSno	508790	4.959
NIAID AIDSno	508797	4.620
NIAID AIDSno	508798	4.796
NIAID AIDSno	508799	4.721
NIAID AIDSno	508800	4.222
NIAID AIDSno	508801	4.523
NIAID AIDSno	508802	4.921
NIAID AIDSno	508803	4.602
NIAID AIDSno	508804	4.161
NIAID AIDSno	508805	4.387
NIAID AIDSno	508807	5.523
NIAID AIDSno	508811	4.854
NIAID AIDSno	508812	4.959
NIAID AIDSno	508814	5.000
NIAID AIDSno	508816	4.569
NIAID AIDSno	508818	4.244
NIAID AIDSno	508819	4.000
NIAID AIDSno	509984	7.538
NIAID AIDSno	509997	6.523
NIAID AIDSno	510002	7.387
NIAID AIDSno	510004	6.485
NIAID AIDSno	510005	5.638
NIAID AIDSno	510597	6.253
NIAID AIDSno	510999	8.222
NIAID AIDSno	511002	7.523
NIAID AIDSno	511214	8.523
NIAID AIDSno	511218	8.699
NIAID AIDSno	511225	8.301
NIAID AIDSno	511226	8.222
NIAID AIDSno	511227	8.398
NIAID AIDSno	512162	7.824
NIAID AIDSno	512164	5.889
NIAID AIDSno	512165	5.114
NIAID AIDSno	512166	4.759
NIAID AIDSno	512167	7.328
NIAID AIDSno	512168	4.457
NIAID AIDSno	512169	6.553
NIAID AIDSno	512170	6.924
NIAID AIDSno	512171	8.222
NIAID AIDSno	512173	5.196
NIAID AIDSno	512490	4.461
NIAID AIDSno	512491	4.966
NIAID AIDSno	512492	4.542

NIAID AIDSno	512494	4.523
NIAID AIDSno	512495	4.487
NIAID AIDSno	512496	5.622
NIAID AIDSno	512497	5.626
NIAID AIDSno	512498	6.409
NIAID AIDSno	512499	6.678
NIAID AIDSno	512500	6.824
NIAID AIDSno	512501	3.783
NIAID AIDSno	512502	4.893
NIAID AIDSno	512503	5.432
NIAID AIDSno	512504	5.310
NIAID AIDSno	512505	4.801
NIAID AIDSno	512506	4.272
NIAID AIDSno	512507	5.754
NIAID AIDSno	512508	6.102
NIAID AIDSno	512509	6.347
NIAID AIDSno	512510	6.237
NIAID AIDSno	512511	5.959
NIAID AIDSno	512513	5.921
NIAID AIDSno	512514	6.194
NIAID AIDSno	512515	6.481
NIAID AIDSno	512516	5.678
NIAID AIDSno	512522	4.484
NIAID AIDSno	512549	5.830
NIAID AIDSno	512556	6.187
NIAID AIDSno	512759	4.678
NIAID AIDSno	512760	5.137
NIAID AIDSno	512761	5.456
NIAID AIDSno	512762	5.260
NIAID AIDSno	512763	5.086
NIAID AIDSno	512765	4.071
NIAID AIDSno	512766	3.979
NIAID AIDSno	513276	5.319
NIAID AIDSno	513277	5.638
NIAID AIDSno	513282	5.796
NIAID AIDSno	513283	5.495
NIAID AIDSno	513284	5.347
NIAID AIDSno	513285	4.658
NIAID AIDSno	513286	4.337
NIAID AIDSno	513287	6.357
NIAID AIDSno	513291	6.252
NIAID AIDSno	513294	4.125
NIAID AIDSno	513295	4.444
NIAID AIDSno	513296	4.959
NIAID AIDSno	513297	4.495

NIAID AIDSno	513298	4.137
NIAID AIDSno	513299	4.553
NIAID AIDSno	513301	4.620
NIAID AIDSno	513308	4.602
NIAID AIDSno	513309	4.854
NIAID AIDSno	513310	4.495
NIAID AIDSno	513311	5.046
NIAID AIDSno	513312	4.921
NIAID AIDSno	513313	5.301
NIAID AIDSno	513314	4.456
NIAID AIDSno	513315	5.155
NIAID AIDSno	513316	4.721
NIAID AIDSno	513317	4.469
NIAID AIDSno	513318	4.292
NIAID AIDSno	513319	4.745
NIAID AIDSno	513320	4.796
NIAID AIDSno	513321	4.854
NIAID AIDSno	513322	4.886
NIAID AIDSno	513324	4.276
NIAID AIDSno	513326	4.036
NIAID AIDSno	513327	4.602
NIAID AIDSno	513328	4.770
NIAID AIDSno	513329	4.523
NIAID AIDSno	513330	4.387
NIAID AIDSno	513331	4.770
NIAID AIDSno	513332	4.620
NIAID AIDSno	513333	4.523
NIAID AIDSno	513334	4.538
NIAID AIDSno	513335	4.824
NIAID AIDSno	513336	4.409
NIAID AIDSno	513337	4.237
NIAID AIDSno	513338	4.119
NIAID AIDSno	513339	4.119
NIAID AIDSno	513340	5.046
NIAID AIDSno	513341	4.745
NIAID AIDSno	513342	4.796
NIAID AIDSno	513343	4.745
NIAID AIDSno	513344	4.260
NIAID AIDSno	513345	4.337
NIAID AIDSno	513346	4.268
NIAID AIDSno	513347	4.959
NIAID AIDSno	513348	4.796
NIAID AIDSno	513349	4.201
NIAID AIDSno	513350	4.284
NIAID AIDSno	513351	4.495

NIAID AIDSno	513352	4.745
NIAID AIDSno	513353	4.229
NIAID AIDSno	513354	4.357
NIAID AIDSno	513355	4.301
NIAID AIDSno	513356	4.167
NIAID AIDSno	513357	4.678
NIAID AIDSno	513358	5.097
NIAID AIDSno	513359	4.244
NIAID AIDSno	513360	4.824
NIAID AIDSno	513750	5.539
NIAID AIDSno	513751	5.310
NIAID AIDSno	513827	6.000
NIAID AIDSno	513828	6.000
NIAID AIDSno	513830	9.000
NIAID AIDSno	513831	6.000
NIAID AIDSno	513832	6.000
NIAID AIDSno	513835	9.000
NIAID AIDSno	513837	6.000
NIAID AIDSno	513838	5.000
NIAID AIDSno	513840	6.000
NIAID AIDSno	513841	6.000
NIAID AIDSno	514129	7.398
NIAID AIDSno	514133	7.362
NIAID AIDSno	514156	7.357
NIAID AIDSno	514157	7.415
NIAID AIDSno	514161	7.382
NIAID AIDSno	514354	5.495
NIAID AIDSno	514356	7.000
NIAID AIDSno	514358	7.000
NIAID AIDSno	514361	6.921
NIAID AIDSno	515391	8.538
NIAID AIDSno	515392	7.495
NIAID AIDSno	515393	7.745
NIAID AIDSno	515394	7.301
NIAID AIDSno	515395	7.770
NIAID AIDSno	515396	7.092
NIAID AIDSno	515397	7.824
NIAID AIDSno	515398	7.824
NIAID AIDSno	515399	7.585
NIAID AIDSno	515400	7.456
NIAID AIDSno	515401	7.959
NIAID AIDSno	515402	7.921
NIAID AIDSno	515403	7.959
NIAID AIDSno	515404	7.854
NIAID AIDSno	515405	7.770

NIAID AIDSno	515406	7.854
NIAID AIDSno	515407	7.796
NIAID AIDSno	515408	7.886
NIAID AIDSno	515409	7.886
NIAID AIDSno	515410	7.854
NIAID AIDSno	515411	8.056
NIAID AIDSno	515412	7.854
NIAID AIDSno	515413	7.886
NIAID AIDSno	515414	7.367
NIAID AIDSno	515415	7.180
NIAID AIDSno	515416	7.886
NIAID AIDSno	515417	7.658
NIAID AIDSno	515418	7.444
NIAID AIDSno	515466	4.432
NIAID AIDSno	515467	4.602
NIAID AIDSno	515468	4.678
NIAID AIDSno	515469	4.387
NIAID AIDSno	515470	5.387
NIAID AIDSno	515471	5.155
NIAID AIDSno	515521	6.102
NIAID AIDSno	515522	5.051
NIAID AIDSno	515978	7.301
NIAID AIDSno	515982	7.523
NIAID AIDSno	515983	7.523
NIAID AIDSno	516482	6.339
NIAID AIDSno	516552	4.367
NIAID AIDSno	521068	3.854
NIAID AIDSno	521074	3.730
NIAID AIDSno	521076	4.000
NIAID AIDSno	521077	4.071
NIAID AIDSno	521078	4.013
NIAID AIDSno	521079	3.955
NIAID AIDSno	521080	4.585
NIAID AIDSno	521081	4.638
NIAID AIDSno	521082	4.620
NIAID AIDSno	521083	4.000
NIAID AIDSno	521084	4.237
NIAID AIDSno	521085	4.921
NIAID AIDSno	521086	4.824
NIAID AIDSno	521087	4.678
NIAID AIDSno	521090	4.745
NIAID AIDSno	521091	4.252
NIAID AIDSno	521092	4.000
NIAID AIDSno	521093	5.301
NIAID AIDSno	521094	4.357

NIAID AIDSno	521095	5.097
NIAID AIDSno	521096	4.886
NIAID AIDSno	521097	4.959
NIAID AIDSno	521098	4.721
NIAID AIDSno	521099	4.357
NIAID AIDSno	521100	4.469
NIAID AIDSno	521101	4.854
NIAID AIDSno	521102	4.658
NIAID AIDSno	521103	4.699
NIAID AIDSno	521104	4.409
NIAID AIDSno	521898	5.260
NIAID AIDSno	523088	5.971
NIAID AIDSno	523089	5.866
NIAID AIDSno	523090	5.054
NIAID AIDSno	523091	5.168
NIAID AIDSno	523165	4.253
NIAID AIDSno	523171	5.024
NIAID AIDSno	523175	4.628
NIAID AIDSno	523179	5.281
NIAID AIDSno	523370	6.553
NIAID AIDSno	523372	6.022
NIAID AIDSno	523373	6.745
NIAID AIDSno	523947	7.445
NIAID AIDSno	523948	7.456
NIAID AIDSno	524493	7.886
NIAID AIDSno	524494	9.000
NIAID AIDSno	524495	8.222
NIAID AIDSno	524496	7.886
NIAID AIDSno	524497	8.222
NIAID AIDSno	524498	7.456
NIAID AIDSno	524499	7.495
NIAID AIDSno	524500	8.155
NIAID AIDSno	524501	8.000
NIAID AIDSno	524502	8.222
NIAID AIDSno	524503	8.097
NIAID AIDSno	524504	7.699
NIAID AIDSno	524505	7.770
NIAID AIDSno	524506	7.745
NIAID AIDSno	524507	7.469
NIAID AIDSno	524511	7.553
NIAID AIDSno	524512	7.553
NIAID AIDSno	524513	7.495
NIAID AIDSno	524514	7.854
NIAID AIDSno	524515	8.398
NIAID AIDSno	524516	7.721

NIAID AIDSno	524517	8.097
NIAID AIDSno	524633	4.357
NIAID AIDSno	524634	4.347
NIAID AIDSno	524635	4.398
NIAID AIDSno	524638	4.000
NIAID AIDSno	526221	4.398
NIAID AIDSno	526224	5.585
NIAID AIDSno	526230	4.495
NIAID AIDSno	526232	6.108
NIAID AIDSno	527360	6.854
NIAID AIDSno	527362	6.699
NIAID AIDSno	527363	6.602
NIAID AIDSno	527463	6.638
NIAID AIDSno	527464	8.523
NIAID AIDSno	527470	8.155
NIAID AIDSno	527471	8.097
NIAID AIDSno	527473	7.745
NIAID AIDSno	527478	7.699
NIAID AIDSno	527479	8.155
NIAID AIDSno	527480	8.155
NIAID AIDSno	527481	8.097
NIAID AIDSno	527482	8.222
NIAID AIDSno	527483	8.398
NIAID AIDSno	527523	7.071
NIAID AIDSno	528530	4.018
NIAID AIDSno	528531	4.222
NIAID AIDSno	529330	6.018
NIAID AIDSno	529331	4.939
NIAID AIDSno	529332	5.137
NIAID AIDSno	529333	6.260
NIAID AIDSno	529334	4.252
NIAID AIDSno	529335	4.706
NIAID AIDSno	529336	5.824
NIAID AIDSno	529337	5.770
NIAID AIDSno	529339	5.420
NIAID AIDSno	529340	5.036
NIAID AIDSno	536494	6.728
NIAID AIDSno	536495	5.701
NIAID AIDSno	536496	6.319
NIAID AIDSno	536497	6.276
NIAID AIDSno	536498	6.710
NIAID AIDSno	536499	5.176
NIAID AIDSno	536500	5.297
NIAID AIDSno	536501	5.351
NIAID AIDSno	536502	5.492

NIAID AIDSno	536503	4.301
NIAID AIDSno	536504	6.045
NIAID AIDSno	536505	6.959
NIAID AIDSno	536612	6.558
NIAID AIDSno	536613	7.004
NIAID AIDSno	536614	5.593
NIAID AIDSno	536615	6.595
NIAID AIDSno	536616	6.243
NIAID AIDSno	536617	6.513
NIAID AIDSno	536618	6.588
NIAID AIDSno	536619	5.457
NIAID AIDSno	536620	6.900
NIAID AIDSno	536621	6.529
NIAID AIDSno	536622	5.041
NIAID AIDSno	536623	3.686
NIAID AIDSno	536624	5.060
NIAID AIDSno	536625	5.979
NIAID AIDSno	536626	5.040
NIAID AIDSno	536627	4.683
NIAID AIDSno	536628	6.137
NIAID AIDSno	536629	5.252
NIAID AIDSno	536630	5.951
NIAID AIDSno	536652	7.523
NIAID AIDSno	536653	5.987
NIAID AIDSno	536655	7.831
NIAID AIDSno	536656	6.418
NIAID AIDSno	536657	6.893
NIAID AIDSno	536672	7.523
NIAID AIDSno	536673	8.000
NIAID AIDSno	536674	8.523
NIAID AIDSno	536677	8.000
NIAID AIDSno	536681	7.523
NIAID AIDSno	536711	7.222
NIAID AIDSno	536717	6.569
NIAID AIDSno	536730	6.678
NIAID AIDSno	536731	6.114
NIAID AIDSno	536732	6.569
NIAID AIDSno	536733	6.585
NIAID AIDSno	536734	6.678
NIAID AIDSno	536735	6.301
NIAID AIDSno	536736	6.208
NIAID AIDSno	537202	6.770
NIAID AIDSno	538122	7.537
NIAID AIDSno	540562	4.939
NIAID AIDSno	540564	5.167

NIAID AIDSno	540565	5.268
NIAID AIDSno	540566	6.060
NIAID AIDSno	540567	5.824
NIAID AIDSno	540569	6.398
NIAID AIDSno	540570	6.921
NIAID AIDSno	540571	7.268
NIAID AIDSno	540572	6.959
NIAID AIDSno	540573	6.620
NIAID AIDSno	540574	6.495
NIAID AIDSno	540575	6.509
NIAID AIDSno	540576	6.432
NIAID AIDSno	540577	6.398
NIAID AIDSno	540578	6.721
NIAID AIDSno	540579	7.328
NIAID AIDSno	540580	7.097
NIAID AIDSno	540581	7.237
NIAID AIDSno	540582	5.553
NIAID AIDSno	540585	5.638
NIAID AIDSno	540586	6.387
NIAID AIDSno	540587	6.585
NIAID AIDSno	540588	6.602
NIAID AIDSno	540589	6.721
NIAID AIDSno	541470	6.854
NIAID AIDSno	543206	4.886
NIAID AIDSno	543213	4.824
NIAID AIDSno	543252	4.000
NIAID AIDSno	543255	4.125
NIAID AIDSno	543261	4.284
NIAID AIDSno	543652	4.000
NIAID AIDSno	543658	4.509
NIAID AIDSno	547321	5.523
NIAID AIDSno	547328	5.854
NIAID AIDSno	547329	5.602
NIAID AIDSno	547330	5.301
NIAID AIDSno	547331	5.222
NIAID AIDSno	547332	4.959
NIAID AIDSno	547333	5.046
NIAID AIDSno	547334	5.097
NIAID AIDSno	547339	4.658
NIAID AIDSno	547341	5.046
NIAID AIDSno	547342	5.222
NIAID AIDSno	547343	5.155
NIAID AIDSno	547345	6.097
NIAID AIDSno	547349	5.046
NIAID AIDSno	547350	5.398

NIAID AIDSno	547351	4.854
NIAID AIDSno	547353	5.000
NIAID AIDSno	547354	5.398
NIAID AIDSno	547355	5.398
NIAID AIDSno	547358	4.824
NIAID AIDSno	547360	5.523
NIAID AIDSno	547362	5.699
NIAID AIDSno	547364	5.796
NIAID AIDSno	547365	5.796
NIAID AIDSno	547367	4.959
NIAID AIDSno	547369	5.155
NIAID AIDSno	547371	4.796
NIAID AIDSno	547373	4.886
NIAID AIDSno	548122	6.292
NIAID AIDSno	548137	6.648
NIAID AIDSno	548150	6.276
NIAID AIDSno	548179	6.252
NIAID AIDSno	548183	6.140
NIAID AIDSno	548192	5.638
NIAID AIDSno	554389	4.260
NIAID AIDSno	554390	3.840
NIAID AIDSno	554392	4.850
NIAID AIDSno	554393	5.000
NIAID AIDSno	554395	6.046
NIAID AIDSno	554396	5.398
NIAID AIDSno	554397	5.222
NIAID AIDSno	554398	4.260
NIAID AIDSno	557594	6.276
NIAID AIDSno	557595	6.108
NIAID AIDSno	557596	4.745
NIAID AIDSno	557597	4.706
NIAID AIDSno	557599	7.854
NIAID AIDSno	557600	7.770
NIAID AIDSno	557601	7.387
NIAID AIDSno	557602	7.569
NIAID AIDSno	557603	7.398
NIAID AIDSno	557604	7.260
NIAID AIDSno	557605	5.108
NIAID AIDSno	557606	5.092
NIAID AIDSno	557607	5.149
NIAID AIDSno	560850	5.750
NIAID AIDSno	560851	6.400
NIAID AIDSno	560852	8.301
NIAID AIDSno	561797	4.872
NIAID AIDSno	561805	4.799

NIAID AIDSno	561813	5.611
NIAID AIDSno	561821	4.912
NIAID AIDSno	561830	5.446
NIAID AIDSno	564361	6.222
NIAID AIDSno	564363	5.620
NIAID AIDSno	564364	6.770
NIAID AIDSno	564365	5.678
NIAID AIDSno	564366	5.538
NIAID AIDSno	564367	5.886
NIAID AIDSno	564368	5.699
NIAID AIDSno	564369	7.000
NIAID AIDSno	564370	6.678
NIAID AIDSno	564371	7.097
NIAID AIDSno	564372	6.444
NIAID AIDSno	564373	7.097
NIAID AIDSno	564374	6.824
NIAID AIDSno	564375	7.301
NIAID AIDSno	567135	5.398
NIAID AIDSno	567144	5.155
NIAID AIDSno	567261	4.456
NIAID AIDSno	567265	4.301
NIAID AIDSno	567276	4.097
NIAID AIDSno	567277	4.155
NIAID AIDSno	567281	5.119
NIAID AIDSno	567562	7.569
NIAID AIDSno	567563	7.060
NIAID AIDSno	567564	7.102
NIAID AIDSno	567565	6.337
NIAID AIDSno	567566	7.301
NIAID AIDSno	567571	6.553
NIAID AIDSno	567575	7.620
NIAID AIDSno	567579	7.796
NIAID AIDSno	567582	7.409
NIAID AIDSno	567583	7.839
NIAID AIDSno	567584	7.569
NIAID AIDSno	567585	7.678
NIAID AIDSno	567586	7.669
NIAID AIDSno	567589	8.043
NIAID AIDSno	567591	7.981
NIAID AIDSno	567593	6.509
NIAID AIDSno	567594	7.721
NIAID AIDSno	567595	6.469
NIAID AIDSno	567596	6.076
NIAID AIDSno	567597	6.886
NIAID AIDSno	567598	6.886

NIAID AIDSno	567599	7.745
NIAID AIDSno	568350	5.500
NIAID AIDSno	568351	5.100
NIAID AIDSno	568352	5.409
NIAID AIDSno	568353	6.102
NIAID AIDSno	568354	6.201
NIAID AIDSno	568355	5.701
NIAID AIDSno	568356	5.900
NIAID AIDSno	568357	6.301
NIAID AIDSno	568358	5.801
NIAID AIDSno	568359	5.886
NIAID AIDSno	568360	5.701
NIAID AIDSno	568361	4.799
NIAID AIDSno	570108	6.222
NIAID AIDSno	570109	6.097
NIAID AIDSno	570110	6.301
NIAID AIDSno	570111	6.000
NIAID AIDSno	570112	6.301
NIAID AIDSno	570113	5.097
NIAID AIDSno	570114	5.699
NIAID AIDSno	570115	5.398
NIAID AIDSno	570116	5.854
NIAID AIDSno	570117	5.222
NIAID AIDSno	570118	5.523
NIAID AIDSno	570119	5.155
NIAID AIDSno	570120	5.854
NIAID AIDSno	570121	5.398
NIAID AIDSno	570122	5.097
NIAID AIDSno	570123	6.000
NIAID AIDSno	570124	5.000
NIAID AIDSno	570125	5.699
NIAID AIDSno	570126	5.699
NIAID AIDSno	570127	6.000
NIAID AIDSno	570128	6.000
NIAID AIDSno	571124	3.547
NIAID AIDSno	573119	7.237
NIAID AIDSno	573120	7.959
NIAID AIDSno	573124	7.745
NIAID AIDSno	573132	7.678
NIAID AIDSno	573134	6.886
NIAID AIDSno	573137	7.678
NIAID AIDSno	573144	7.553
NIAID AIDSno	573152	8.097
NIAID AIDSno	573155	7.921
NIAID AIDSno	573160	7.699

NIAID AIDSno	573163	8.000
NIAID AIDSno	573164	7.699
NIAID AIDSno	573165	8.699
NIAID AIDSno	573166	9.000
NIAID AIDSno	573169	7.377
NIAID AIDSno	574848	4.706
NIAID AIDSno	574849	4.745
NIAID AIDSno	574850	6.276
NIAID AIDSno	574852	6.108
NIAID AIDSno	574865	5.292
NIAID AIDSno	574929	7.796
NIAID AIDSno	574956	4.538
NIAID AIDSno	574958	7.669
NIAID AIDSno	574962	8.043
NIAID AIDSno	574966	7.981
NIAID AIDSno	574981	6.509
NIAID AIDSno	574984	7.481
NIAID AIDSno	574985	7.959
NIAID AIDSno	574986	7.377
NIAID AIDSno	574987	4.155
NIAID AIDSno	574988	4.268
NIAID AIDSno	574989	4.018
NIAID AIDSno	574990	5.721
NIAID AIDSno	574991	4.268
NIAID AIDSno	574992	5.208
NIAID AIDSno	574998	8.495
NIAID AIDSno	575000	8.796
NIAID AIDSno	575001	5.485
NIAID AIDSno	575002	5.703
NIAID AIDSno	575007	7.839
NIAID AIDSno	575010	7.947
NIAID AIDSno	575011	8.297
NIAID AIDSno	575012	7.845
NIAID AIDSno	575013	8.268
NIAID AIDSno	575014	9.229
NIAID AIDSno	575015	5.476
NIAID AIDSno	575016	8.602
NIAID AIDSno	575017	8.569
NIAID AIDSno	575018	7.907
NIAID AIDSno	575019	8.432
NIAID AIDSno	575020	8.602
NIAID AIDSno	575022	8.745
NIAID AIDSno	575023	8.509
NIAID AIDSno	575067	7.839
NIAID AIDSno	575068	8.409

NIAID AIDSno	575069	10.155
NIAID AIDSno	575070	8.432
NIAID AIDSno	575962	7.678
NIAID AIDSno	575964	7.721
NIAID AIDSno	576007	8.301
NIAID AIDSno	576008	8.301
NIAID AIDSno	576009	8.000
NIAID AIDSno	576011	7.796
NIAID AIDSno	576012	8.699
NIAID AIDSno	576016	7.959
NIAID AIDSno	576019	7.770
NIAID AIDSno	576020	8.222
NIAID AIDSno	576234	6.046
NIAID AIDSno	576246	5.620
NIAID AIDSno	576247	4.724
NIAID AIDSno	576248	5.456
NIAID AIDSno	576249	4.650
NIAID AIDSno	576250	4.730
NIAID AIDSno	576251	5.602
NIAID AIDSno	576252	6.155
NIAID AIDSno	576369	4.788
NIAID AIDSno	576370	5.229
NIAID AIDSno	576373	4.873
NIAID AIDSno	576374	4.883
NIAID AIDSno	576375	4.674
NIAID AIDSno	584775	4.921
NIAID AIDSno	584783	6.337
NIAID AIDSno	584784	6.222
NIAID AIDSno	584785	5.553
NIAID AIDSno	584786	5.180
NIAID AIDSno	584787	4.821
NIAID AIDSno	584790	4.747
NIAID AIDSno	584791	5.119
NIAID AIDSno	584792	4.656
NIAID AIDSno	584794	6.086
NIAID AIDSno	584801	4.975
NIAID AIDSno	584804	4.928
NIAID AIDSno	584805	5.102
NIAID AIDSno	584807	4.654
NIAID AIDSno	584810	4.599
NIAID AIDSno	584812	5.284
NIAID AIDSno	584817	6.155
NIAID AIDSno	584818	5.509
NIAID AIDSno	586645	5.244
NIAID AIDSno	586646	5.018

NIAID AIDSno	586647	5.796
NIAID AIDSno	586648	4.830
NIAID AIDSno	586649	5.678
NIAID AIDSno	586650	6.398
NIAID AIDSno	586651	7.022
NIAID AIDSno	586652	7.022
NIAID AIDSno	586653	7.155
NIAID AIDSno	586654	6.796
NIAID AIDSno	586655	6.854
NIAID AIDSno	587490	5.658
NIAID AIDSno	587495	5.357
NIAID AIDSno	587499	5.337
NIAID AIDSno	587508	4.523
NIAID AIDSno	587510	5.149
NIAID AIDSno	587511	4.602
NIAID AIDSno	587521	4.921
NIAID AIDSno	587522	5.149
NIAID AIDSno	587530	6.000
NIAID AIDSno	587541	4.032
NIAID AIDSno	587542	4.357
NIAID AIDSno	587698	4.456
NIAID AIDSno	587699	5.921
NIAID AIDSno	587700	5.886
NIAID AIDSno	588087	5.620
NIAID AIDSno	588105	5.886
NIAID AIDSno	588107	6.721
NIAID AIDSno	588109	6.194
NIAID AIDSno	588113	5.959
NIAID AIDSno	588114	5.252
NIAID AIDSno	588116	5.638
NIAID AIDSno	588140	5.444
NIAID AIDSno	588141	5.481
NIAID AIDSno	588142	5.699
NIAID AIDSno	588143	5.620
NIAID AIDSno	588144	5.721
NIAID AIDSno	588145	5.886
NIAID AIDSno	588146	6.071
NIAID AIDSno	588147	5.652
NIAID AIDSno	588148	6.036
NIAID AIDSno	588149	5.644
NIAID AIDSno	588150	5.215
NIAID AIDSno	588151	5.553
NIAID AIDSno	588152	5.921
NIAID AIDSno	588195	4.434
NIAID AIDSno	588196	4.460

NIAID AIDSno	588197	4.401
NIAID AIDSno	588198	4.477
NIAID AIDSno	588199	4.447
NIAID AIDSno	588200	4.908
NIAID AIDSno	588267	7.602
NIAID AIDSno	588268	5.000
NIAID AIDSno	588269	5.638
NIAID AIDSno	588270	6.484
NIAID AIDSno	588271	6.602
NIAID AIDSno	588272	6.260
NIAID AIDSno	588273	6.260
NIAID AIDSno	588274	7.097
NIAID AIDSno	588689	4.432
NIAID AIDSno	588690	4.456
NIAID AIDSno	588691	4.456
NIAID AIDSno	588692	4.469
NIAID AIDSno	588695	4.174
NIAID AIDSno	588696	4.444
NIAID AIDSno	588697	4.602
NIAID AIDSno	588698	4.352
NIAID AIDSno	588705	4.638
NIAID AIDSno	588706	5.646
NIAID AIDSno	588707	4.854
NIAID AIDSno	588708	4.770
NIAID AIDSno	588709	4.658
NIAID AIDSno	588710	5.108
NIAID AIDSno	588711	4.796
NIAID AIDSno	588713	4.456
NIAID AIDSno	588715	4.495
NIAID AIDSno	588791	5.398
NIAID AIDSno	588792	5.474
NIAID AIDSno	589365	5.260
NIAID AIDSno	589366	4.456
NIAID AIDSno	589375	5.046
NIAID AIDSno	589378	5.046
NIAID AIDSno	589385	5.301
NIAID AIDSno	589387	5.602
NIAID AIDSno	589388	5.284
NIAID AIDSno	589639	7.481
NIAID AIDSno	589642	7.097
NIAID AIDSno	589643	6.602
NIAID AIDSno	589644	6.959
NIAID AIDSno	589645	6.959
NIAID AIDSno	589646	7.180
NIAID AIDSno	589647	7.237

NIAID AIDSno	589648	6.824
NIAID AIDSno	589649	6.679
NIAID AIDSno	589650	7.092
NIAID AIDSno	589651	6.046
NIAID AIDSno	589652	6.084
NIAID AIDSno	589653	7.310
NIAID AIDSno	589654	7.167
NIAID AIDSno	589655	7.319
NIAID AIDSno	589656	6.638
NIAID AIDSno	589657	7.114
NIAID AIDSno	589658	7.022
NIAID AIDSno	589659	6.921
NIAID AIDSno	589660	7.678
NIAID AIDSno	589661	7.292
NIAID AIDSno	589662	7.000
NIAID AIDSno	589663	4.456
NIAID AIDSno	589668	5.155
NIAID AIDSno	589669	4.432
NIAID AIDSno	589670	6.367
NIAID AIDSno	589956	6.921
NIAID AIDSno	592431	8.125
NIAID AIDSno	594716	4.924
NIAID AIDSno	594729	4.971
NIAID AIDSno	594730	5.244
NIAID AIDSno	594731	4.979
NIAID AIDSno	594732	5.018
NIAID AIDSno	594733	4.936
NIAID AIDSno	594734	4.937
NIAID AIDSno	594735	4.703
NIAID AIDSno	594736	4.693
NIAID AIDSno	594737	4.697
NIAID AIDSno	594740	4.648
NIAID AIDSno	594741	4.611
NIAID AIDSno	594742	6.699
NIAID AIDSno	594743	4.975
NIAID AIDSno	594744	6.301
NIAID AIDSno	594745	5.921
NIAID AIDSno	594746	4.602
NIAID AIDSno	597945	6.071
NIAID AIDSno	597947	6.854
NIAID AIDSno	598037	8.523
NIAID AIDSno	598162	7.104
NIAID AIDSno	598163	7.361
NIAID AIDSno	598165	7.000
NIAID AIDSno	598166	6.745

NIAID AIDSno	598167	6.699
NIAID AIDSno	598168	6.886
NIAID AIDSno	598169	7.523
NIAID AIDSno	598170	7.215
NIAID AIDSno	598171	7.699
NIAID AIDSno	598173	7.523
NIAID AIDSno	598174	6.149
NIAID AIDSno	598175	6.161
NIAID AIDSno	598176	7.678
NIAID AIDSno	598177	7.208
NIAID AIDSno	598178	7.155
NIAID AIDSno	598179	7.432
NIAID AIDSno	598180	7.000
NIAID AIDSno	598181	7.301
NIAID AIDSno	598182	6.886
NIAID AIDSno	598183	7.387
NIAID AIDSno	598184	7.495
NIAID AIDSno	598185	5.398
NIAID AIDSno	598186	7.921
NIAID AIDSno	598187	8.398
NIAID AIDSno	598188	7.284
NIAID AIDSno	598189	7.959
NIAID AIDSno	598190	8.155
NIAID AIDSno	598191	8.097
NIAID AIDSno	598192	7.301
NIAID AIDSno	598193	7.481
NIAID AIDSno	598194	7.824
NIAID AIDSno	598195	7.699
NIAID AIDSno	598196	7.180
NIAID AIDSno	598197	7.149
NIAID AIDSno	598199	6.757
NIAID AIDSno	598200	7.208
NIAID AIDSno	598201	7.208
NIAID AIDSno	598202	7.721
NIAID AIDSno	598203	6.854
NIAID AIDSno	598204	7.678
NIAID AIDSno	598205	7.066
NIAID AIDSno	598206	7.553
NIAID AIDSno	598207	7.357
NIAID AIDSno	598209	7.523
NIAID AIDSno	598498	4.310
NIAID AIDSno	598500	3.921
NIAID AIDSno	598501	4.155
NIAID AIDSno	598502	4.244
NIAID AIDSno	598504	3.943

NIAID AIDSno	598507	4.268
NIAID AIDSno	598652	5.983
NIAID AIDSno	598653	7.000
NIAID AIDSno	598655	7.398
NIAID AIDSno	598657	6.638
NIAID AIDSno	598658	6.432
NIAID AIDSno	598659	7.398
NIAID AIDSno	598660	6.420
NIAID AIDSno	598692	7.398
NIAID AIDSno	598693	7.699
NIAID AIDSno	598694	7.523
NIAID AIDSno	598695	7.699
NIAID AIDSno	598700	4.564
NIAID AIDSno	598701	4.688
NIAID AIDSno	598702	4.296
NIAID AIDSno	598703	4.971
NIAID AIDSno	598706	4.602
NIAID AIDSno	598707	4.772
NIAID AIDSno	598708	4.771
NIAID AIDSno	598714	5.623
NIAID AIDSno	598715	4.629
NIAID AIDSno	598721	5.280
NIAID AIDSno	598730	6.301
NIAID AIDSno	598731	5.180
NIAID AIDSno	598737	5.481
NIAID AIDSno	598740	5.569
NIAID AIDSno	598806	7.523
NIAID AIDSno	598807	7.097
NIAID AIDSno	598808	6.495
NIAID AIDSno	598809	6.862
NIAID AIDSno	598810	7.398
NIAID AIDSno	598811	7.337
NIAID AIDSno	598813	7.276
NIAID AIDSno	598814	7.086
NIAID AIDSno	598815	6.569
NIAID AIDSno	598816	6.854
NIAID AIDSno	598896	6.222
NIAID AIDSno	598897	6.398
NIAID AIDSno	598900	6.699
NIAID AIDSno	598901	5.252
NIAID AIDSno	598902	4.873
NIAID AIDSno	598905	6.796
NIAID AIDSno	598907	5.620
NIAID AIDSno	598908	6.638
NIAID AIDSno	598909	7.699

NIAID AIDSno	599064	5.699
NIAID AIDSno	599065	4.959
NIAID AIDSno	599069	5.347
NIAID AIDSno	599070	4.770
NIAID AIDSno	599088	4.638
NIAID AIDSno	599089	5.658
NIAID AIDSno	599090	5.301
NIAID AIDSno	599091	5.886
NIAID AIDSno	599092	5.678
NIAID AIDSno	599093	4.456
NIAID AIDSno	599094	4.921
NIAID AIDSno	599095	5.076
NIAID AIDSno	599096	5.102
NIAID AIDSno	599097	5.252
NIAID AIDSno	599098	5.444
NIAID AIDSno	599100	4.770
NIAID AIDSno	599101	5.081
NIAID AIDSno	599170	8.699
NIAID AIDSno	599171	7.921
NIAID AIDSno	599172	8.000
NIAID AIDSno	599173	7.824
NIAID AIDSno	599174	8.398
NIAID AIDSno	599175	7.824
NIAID AIDSno	599176	7.699
NIAID AIDSno	599177	7.585
NIAID AIDSno	599180	7.678
NIAID AIDSno	599182	8.398
NIAID AIDSno	599184	8.046
NIAID AIDSno	599187	7.824
NIAID AIDSno	600577	5.137
NIAID AIDSno	600578	5.131
NIAID AIDSno	600580	4.886
NIAID AIDSno	600581	5.194
NIAID AIDSno	600583	5.796
NIAID AIDSno	601515	5.046
NIAID AIDSno	601516	4.921
NIAID AIDSno	601520	6.000
NIAID AIDSno	601521	5.046
NIAID AIDSno	601522	4.886
NIAID AIDSno	601523	4.824
NIAID AIDSno	604666	8.097
NIAID AIDSno	604667	8.699
NIAID AIDSno	604669	7.959
NIAID AIDSno	604670	7.959
NIAID AIDSno	604671	8.000

NIAID AIDSno	604838	5.928
NIAID AIDSno	604839	4.642
NIAID AIDSno	604841	6.602
NIAID AIDSno	604842	5.827
NIAID AIDSno	604843	4.965
NIAID AIDSno	604845	6.658
NIAID AIDSno	604847	5.171
NIAID AIDSno	604849	5.959
NIAID AIDSno	604850	5.553
NIAID AIDSno	604851	5.886
NIAID AIDSno	604852	6.125
NIAID AIDSno	604854	6.056
NIAID AIDSno	604855	6.056
NIAID AIDSno	604856	5.167
NIAID AIDSno	604857	6.310
NIAID AIDSno	604858	6.114
NIAID AIDSno	604859	6.194
NIAID AIDSno	604860	6.229
NIAID AIDSno	604862	5.921
NIAID AIDSno	604863	5.959
NIAID AIDSno	604864	5.469
NIAID AIDSno	604865	5.796
NIAID AIDSno	604866	5.444
NIAID AIDSno	604867	5.569
NIAID AIDSno	604868	5.076
NIAID AIDSno	604869	6.102
NIAID AIDSno	604877	6.770
NIAID AIDSno	604880	6.481
NIAID AIDSno	604887	6.620
NIAID AIDSno	605123	6.002
NIAID AIDSno	605124	7.500

7.2.Protease set

Source	ID	pIC50
ChEMBL MOLREGNO	72914	6.528
ChEMBL MOLREGNO	141594	7.886
ChEMBL MOLREGNO	136201	8.658
ChEMBL MOLREGNO	95005	6.523
ChEMBL MOLREGNO	86680	9.745
ChEMBL	97239	7.210

MOLREGNO		
ChEMBL MOLREGNO	70049	9.046
ChEMBL MOLREGNO	127424	9.648
ChEMBL MOLREGNO	127846	7.860
ChEMBL MOLREGNO	11390	7.921
ChEMBL MOLREGNO	8464	9.347
ChEMBL MOLREGNO	11435	6.921
ChEMBL MOLREGNO	121512	9.237
ChEMBL MOLREGNO	51711	8.387
ChEMBL MOLREGNO	112844	8.149
ChEMBL MOLREGNO	51459	7.824
ChEMBL MOLREGNO	284474	7.055
ChEMBL MOLREGNO	282362	8.456
ChEMBL MOLREGNO	290991	6.409
ChEMBL MOLREGNO	228472	7.638
ChEMBL MOLREGNO	97357	6.440
ChEMBL MOLREGNO	170680	7.824
ChEMBL MOLREGNO	187715	9.585
ChEMBL MOLREGNO	190686	8.108
ChEMBL MOLREGNO	171629	9.046
ChEMBL MOLREGNO	96264	7.728
ChEMBL MOLREGNO	174903	5.959
ChEMBL MOLREGNO	204311	6.102
ChEMBL MOLREGNO	439912	8.008
ChEMBL MOLREGNO	157440	8.959

ChEMBL MOLREGNO	331061	6.102
ChEMBL MOLREGNO	1798165	6.933
ChEMBL MOLREGNO	308750	11.000
ChEMBL MOLREGNO	20132	8.241
ChEMBL MOLREGNO	281799	7.854
ChEMBL MOLREGNO	283653	6.327
ChEMBL MOLREGNO	282297	6.883
ChEMBL MOLREGNO	301634	8.301
ChEMBL MOLREGNO	73265	6.111
ChEMBL MOLREGNO	148997	8.252
ChEMBL MOLREGNO	174766	6.745
ChEMBL MOLREGNO	157906	8.237
ChEMBL MOLREGNO	144750	5.854
ChEMBL MOLREGNO	157945	9.620
ChEMBL MOLREGNO	197881	5.481
ChEMBL MOLREGNO	202386	7.217
ChEMBL MOLREGNO	72833	5.398
ChEMBL MOLREGNO	80455	10.301
ChEMBL MOLREGNO	120415	8.913
ChEMBL MOLREGNO	14686	5.595
ChEMBL MOLREGNO	51650	8.463
ChEMBL MOLREGNO	94744	7.143
ChEMBL MOLREGNO	95072	6.824
ChEMBL MOLREGNO	174066	7.854
ChEMBL	20135	7.583

MOLREGNO		
ChEMBL MOLREGNO	127505	9.824
ChEMBL MOLREGNO	2038625	10.301
ChEMBL MOLREGNO	5783	7.237
ChEMBL MOLREGNO	116593	6.757
ChEMBL MOLREGNO	111400	5.824
ChEMBL MOLREGNO	111243	6.699
ChEMBL MOLREGNO	108779	7.721
ChEMBL MOLREGNO	270629	8.066
ChEMBL MOLREGNO	201361	7.678
ChEMBL MOLREGNO	204756	6.569
ChEMBL MOLREGNO	231396	6.046
ChEMBL MOLREGNO	184058	7.932
ChEMBL MOLREGNO	197867	5.838
ChEMBL MOLREGNO	197450	5.450
ChEMBL MOLREGNO	197996	4.943
ChEMBL MOLREGNO	8379	8.268
ChEMBL MOLREGNO	191169	7.420
ChEMBL MOLREGNO	183559	8.149
ChEMBL MOLREGNO	157800	9.046
ChEMBL MOLREGNO	161517	6.481
ChEMBL MOLREGNO	1387201	7.972
ChEMBL MOLREGNO	308705	9.495
ChEMBL MOLREGNO	304695	5.000
ChEMBL MOLREGNO	20009	7.026

ChEMBL MOLREGNO	20583	5.721
ChEMBL MOLREGNO	145178	5.481
ChEMBL MOLREGNO	232220	8.023
ChEMBL MOLREGNO	270809	5.896
ChEMBL MOLREGNO	235908	5.523
ChEMBL MOLREGNO	144847	4.620
ChEMBL MOLREGNO	147986	8.319
ChEMBL MOLREGNO	149411	8.018
ChEMBL MOLREGNO	157932	8.102
ChEMBL MOLREGNO	173868	8.284
ChEMBL MOLREGNO	188177	9.854
ChEMBL MOLREGNO	186944	9.699
ChEMBL MOLREGNO	202119	7.557
ChEMBL MOLREGNO	70309	9.000
ChEMBL MOLREGNO	69520	9.731
ChEMBL MOLREGNO	112347	5.222
ChEMBL MOLREGNO	121675	9.108
ChEMBL MOLREGNO	71741	5.602
ChEMBL MOLREGNO	11046	5.871
ChEMBL MOLREGNO	11215	6.535
ChEMBL MOLREGNO	60425	6.460
ChEMBL MOLREGNO	116626	6.590
ChEMBL MOLREGNO	108995	8.420
ChEMBL MOLREGNO	301591	8.678
ChEMBL	108442	8.658

MOLREGNO		
ChEMBL MOLREGNO	245259	7.456
ChEMBL MOLREGNO	271180	6.710
ChEMBL MOLREGNO	96408	7.048
ChEMBL MOLREGNO	204753	4.928
ChEMBL MOLREGNO	179510	6.900
ChEMBL MOLREGNO	185295	6.820
ChEMBL MOLREGNO	190958	7.824
ChEMBL MOLREGNO	201452	8.602
ChEMBL MOLREGNO	283014	8.444
ChEMBL MOLREGNO	90431	7.097
ChEMBL MOLREGNO	112143	4.149
ChEMBL MOLREGNO	161351	5.862
ChEMBL MOLREGNO	157043	7.194
ChEMBL MOLREGNO	157160	10.000
ChEMBL MOLREGNO	1384053	7.161
ChEMBL MOLREGNO	1799218	8.349
ChEMBL MOLREGNO	148338	7.006
ChEMBL MOLREGNO	149627	8.481
ChEMBL MOLREGNO	291400	7.347
ChEMBL MOLREGNO	333200	10.699
ChEMBL MOLREGNO	205503	6.538
ChEMBL MOLREGNO	204869	6.886
ChEMBL MOLREGNO	205740	7.051
ChEMBL MOLREGNO	257816	8.824

ChEMBL MOLREGNO	269741	7.046
ChEMBL MOLREGNO	160288	8.734
ChEMBL MOLREGNO	160643	8.841
ChEMBL MOLREGNO	183846	8.704
ChEMBL MOLREGNO	188215	4.678
ChEMBL MOLREGNO	184161	5.886
ChEMBL MOLREGNO	179835	7.770
ChEMBL MOLREGNO	183882	7.699
ChEMBL MOLREGNO	70107	8.854
ChEMBL MOLREGNO	69479	9.699
ChEMBL MOLREGNO	112371	7.869
ChEMBL MOLREGNO	14423	5.125
ChEMBL MOLREGNO	40458	7.674
ChEMBL MOLREGNO	51075	7.339
ChEMBL MOLREGNO	51065	5.046
ChEMBL MOLREGNO	128877	8.796
ChEMBL MOLREGNO	8625	9.538
ChEMBL MOLREGNO	8564	8.357
ChEMBL MOLREGNO	11006	6.793
ChEMBL MOLREGNO	60943	6.854
ChEMBL MOLREGNO	112579	7.699
ChEMBL MOLREGNO	111484	7.854
ChEMBL MOLREGNO	112819	8.921
ChEMBL MOLREGNO	283182	8.276
ChEMBL	287452	6.550

MOLREGNO		
ChEMBL MOLREGNO	1548890	8.921
ChEMBL MOLREGNO	257850	6.398
ChEMBL MOLREGNO	258236	7.036
ChEMBL MOLREGNO	271195	7.036
ChEMBL MOLREGNO	231896	7.432
ChEMBL MOLREGNO	97281	7.553
ChEMBL MOLREGNO	188345	9.328
ChEMBL MOLREGNO	198213	5.151
ChEMBL MOLREGNO	94842	7.301
ChEMBL MOLREGNO	157968	10.301
ChEMBL MOLREGNO	157157	8.201
ChEMBL MOLREGNO	27507	4.276
ChEMBL MOLREGNO	1384020	6.634
ChEMBL MOLREGNO	308704	9.398
ChEMBL MOLREGNO	290992	6.372
ChEMBL MOLREGNO	333015	10.398
ChEMBL MOLREGNO	331000	6.385
ChEMBL MOLREGNO	145138	4.495
ChEMBL MOLREGNO	205319	7.375
ChEMBL MOLREGNO	76668	9.560
ChEMBL MOLREGNO	166865	7.796
ChEMBL MOLREGNO	159732	8.481
ChEMBL MOLREGNO	174738	6.714
ChEMBL MOLREGNO	191047	9.523

ChEMBL MOLREGNO	190647	6.111
ChEMBL MOLREGNO	184063	8.077
ChEMBL MOLREGNO	197683	5.878
ChEMBL MOLREGNO	183624	7.921
ChEMBL MOLREGNO	202030	8.387
ChEMBL MOLREGNO	147254	7.960
ChEMBL MOLREGNO	79941	10.398
ChEMBL MOLREGNO	97003	7.594
ChEMBL MOLREGNO	112111	8.409
ChEMBL MOLREGNO	120554	5.516
ChEMBL MOLREGNO	127748	10.111
ChEMBL MOLREGNO	14514	6.611
ChEMBL MOLREGNO	14422	5.770
ChEMBL MOLREGNO	70552	8.699
ChEMBL MOLREGNO	51680	7.960
ChEMBL MOLREGNO	95006	7.523
ChEMBL MOLREGNO	90429	7.347
ChEMBL MOLREGNO	69814	9.770
ChEMBL MOLREGNO	8595	9.469
ChEMBL MOLREGNO	41812	9.509
ChEMBL MOLREGNO	14683	6.222
ChEMBL MOLREGNO	1627599	6.409
ChEMBL MOLREGNO	8376	8.745
ChEMBL MOLREGNO	11078	7.051
ChEMBL	61583	6.602

MOLREGNO		
ChEMBL MOLREGNO	51718	7.770
ChEMBL MOLREGNO	11218	6.377
ChEMBL MOLREGNO	1595394	10.188
ChEMBL MOLREGNO	51440	8.456
ChEMBL MOLREGNO	52057	8.301
ChEMBL MOLREGNO	117265	6.321
ChEMBL MOLREGNO	333322	10.301
ChEMBL MOLREGNO	108702	5.084
ChEMBL MOLREGNO	110080	5.616
ChEMBL MOLREGNO	282658	8.066
ChEMBL MOLREGNO	304682	4.301
ChEMBL MOLREGNO	265649	4.201
ChEMBL MOLREGNO	271120	6.730
ChEMBL MOLREGNO	171590	7.204
ChEMBL MOLREGNO	174135	7.237
ChEMBL MOLREGNO	183999	7.201
ChEMBL MOLREGNO	188830	9.745
ChEMBL MOLREGNO	198023	5.387
ChEMBL MOLREGNO	205279	8.000
ChEMBL MOLREGNO	204549	7.554
ChEMBL MOLREGNO	205030	7.328
ChEMBL MOLREGNO	201421	8.509
ChEMBL MOLREGNO	87142	8.796
ChEMBL MOLREGNO	160868	7.538

ChEMBL MOLREGNO	20624	6.687
ChEMBL MOLREGNO	188236	9.770
ChEMBL MOLREGNO	153633	10.222
ChEMBL MOLREGNO	158239	8.538
ChEMBL MOLREGNO	156434	7.347
ChEMBL MOLREGNO	350091	8.452
ChEMBL MOLREGNO	75685	5.952
ChEMBL MOLREGNO	271763	8.745
ChEMBL MOLREGNO	284676	5.000
ChEMBL MOLREGNO	290820	7.357
ChEMBL MOLREGNO	75445	5.469
ChEMBL MOLREGNO	76142	5.721
ChEMBL MOLREGNO	73449	6.495
ChEMBL MOLREGNO	179836	7.469
ChEMBL MOLREGNO	145382	5.638
ChEMBL MOLREGNO	70702	9.538
ChEMBL MOLREGNO	90445	6.503
ChEMBL MOLREGNO	119475	8.852
ChEMBL MOLREGNO	108403	7.854
ChEMBL MOLREGNO	120776	7.854
ChEMBL MOLREGNO	119668	9.824
ChEMBL MOLREGNO	69831	8.538
ChEMBL MOLREGNO	14539	5.788
ChEMBL MOLREGNO	158320	9.699
ChEMBL	10983	7.585

MOLREGNO		
ChEMBL MOLREGNO	11181	7.119
ChEMBL MOLREGNO	60967	6.886
ChEMBL MOLREGNO	117570	6.991
ChEMBL MOLREGNO	111973	8.638
ChEMBL MOLREGNO	1154140	8.444
ChEMBL MOLREGNO	289083	7.216
ChEMBL MOLREGNO	268251	5.347
ChEMBL MOLREGNO	95016	7.071
ChEMBL MOLREGNO	204950	6.764
ChEMBL MOLREGNO	94822	7.066
ChEMBL MOLREGNO	179453	6.848
ChEMBL MOLREGNO	179499	7.387
ChEMBL MOLREGNO	197845	5.460
ChEMBL MOLREGNO	202115	8.770
ChEMBL MOLREGNO	204739	5.886
ChEMBL MOLREGNO	140992	5.969
ChEMBL MOLREGNO	184821	9.000
ChEMBL MOLREGNO	173867	8.357
ChEMBL MOLREGNO	284475	7.833
ChEMBL MOLREGNO	61119	7.155
ChEMBL MOLREGNO	107919	6.870
ChEMBL MOLREGNO	158408	9.056
ChEMBL MOLREGNO	160655	9.038
ChEMBL MOLREGNO	25747	7.796

ChEMBL MOLREGNO	308706	9.319
ChEMBL MOLREGNO	308790	10.699
ChEMBL MOLREGNO	73025	7.213
ChEMBL MOLREGNO	204757	5.921
ChEMBL MOLREGNO	231953	8.000
ChEMBL MOLREGNO	257548	7.398
ChEMBL MOLREGNO	270214	6.205
ChEMBL MOLREGNO	70809	10.155
ChEMBL MOLREGNO	145684	4.276
ChEMBL MOLREGNO	148664	7.456
ChEMBL MOLREGNO	141105	5.450
ChEMBL MOLREGNO	185827	10.699
ChEMBL MOLREGNO	179675	7.796
ChEMBL MOLREGNO	202641	6.876
ChEMBL MOLREGNO	202697	6.837
ChEMBL MOLREGNO	112742	8.469
ChEMBL MOLREGNO	14591	6.208
ChEMBL MOLREGNO	70004	9.745
ChEMBL MOLREGNO	51331	7.854
ChEMBL MOLREGNO	96520	6.297
ChEMBL MOLREGNO	203037	5.187
ChEMBL MOLREGNO	21386	8.260
ChEMBL MOLREGNO	10827	6.907
ChEMBL MOLREGNO	11334	5.620
ChEMBL	2038754	7.860

MOLREGNO		
ChEMBL MOLREGNO	8624	6.658
ChEMBL MOLREGNO	59768	5.699
ChEMBL MOLREGNO	112788	9.046
ChEMBL MOLREGNO	52309	7.879
ChEMBL MOLREGNO	51330	7.222
ChEMBL MOLREGNO	260426	8.387
ChEMBL MOLREGNO	174230	8.620
ChEMBL MOLREGNO	173993	7.125
ChEMBL MOLREGNO	190687	9.409
ChEMBL MOLREGNO	204965	6.357
ChEMBL MOLREGNO	119656	9.620
ChEMBL MOLREGNO	152874	10.824
ChEMBL MOLREGNO	268560	4.292
ChEMBL MOLREGNO	271161	5.957
ChEMBL MOLREGNO	97039	7.699
ChEMBL MOLREGNO	115704	8.602
ChEMBL MOLREGNO	204821	7.222
ChEMBL MOLREGNO	157161	8.569
ChEMBL MOLREGNO	249851	7.553
ChEMBL MOLREGNO	350496	9.319
ChEMBL MOLREGNO	350563	9.398
ChEMBL MOLREGNO	338301	8.620
ChEMBL MOLREGNO	173875	7.409
ChEMBL MOLREGNO	1368171	8.112

ChEMBL MOLREGNO	149284	8.469
ChEMBL MOLREGNO	158048	9.056
ChEMBL MOLREGNO	308753	11.000
ChEMBL MOLREGNO	333604	8.921
ChEMBL MOLREGNO	258210	7.071
ChEMBL MOLREGNO	284476	6.776
ChEMBL MOLREGNO	249959	8.921
ChEMBL MOLREGNO	696290	9.527
ChEMBL MOLREGNO	159641	9.200
ChEMBL MOLREGNO	174272	6.357
ChEMBL MOLREGNO	179428	7.185
ChEMBL MOLREGNO	184048	7.896
ChEMBL MOLREGNO	173660	5.699
ChEMBL MOLREGNO	173960	9.260
ChEMBL MOLREGNO	157178	5.699
ChEMBL MOLREGNO	197684	4.699
ChEMBL MOLREGNO	202874	5.194
ChEMBL MOLREGNO	202064	8.194
ChEMBL MOLREGNO	188207	9.208
ChEMBL MOLREGNO	72835	6.347
ChEMBL MOLREGNO	94937	7.046
ChEMBL MOLREGNO	112436	8.260
ChEMBL MOLREGNO	95073	6.921
ChEMBL MOLREGNO	96442	7.318
ChEMBL	11132	7.167

MOLREGNO		
ChEMBL MOLREGNO	11365	8.824
ChEMBL MOLREGNO	19892	7.927
ChEMBL MOLREGNO	609402	6.730
ChEMBL MOLREGNO	17347	9.000
ChEMBL MOLREGNO	350176	9.167
ChEMBL MOLREGNO	119669	8.548
ChEMBL MOLREGNO	51458	7.917
ChEMBL MOLREGNO	115221	8.959
ChEMBL MOLREGNO	115333	9.022
ChEMBL MOLREGNO	108936	8.000
ChEMBL MOLREGNO	282354	8.585
ChEMBL MOLREGNO	260162	7.495
ChEMBL MOLREGNO	204703	6.959
ChEMBL MOLREGNO	216460	6.699
ChEMBL MOLREGNO	94745	7.194
ChEMBL MOLREGNO	95143	6.959
ChEMBL MOLREGNO	183988	8.167
ChEMBL MOLREGNO	184557	5.316
ChEMBL MOLREGNO	197913	5.477
ChEMBL MOLREGNO	205266	7.495
ChEMBL MOLREGNO	201951	8.347
ChEMBL MOLREGNO	94823	7.301
ChEMBL MOLREGNO	270565	8.824
ChEMBL MOLREGNO	235746	7.000

ChEMBL MOLREGNO	183623	7.553
ChEMBL MOLREGNO	287093	8.046
ChEMBL MOLREGNO	25256	5.553
ChEMBL MOLREGNO	86829	8.268
ChEMBL MOLREGNO	159803	9.301
ChEMBL MOLREGNO	8374	8.921
ChEMBL MOLREGNO	27107	4.051
ChEMBL MOLREGNO	1355736	7.249
ChEMBL MOLREGNO	148962	7.509
ChEMBL MOLREGNO	231785	8.523
ChEMBL MOLREGNO	249853	8.114
ChEMBL MOLREGNO	147987	6.623
ChEMBL MOLREGNO	145383	5.260
ChEMBL MOLREGNO	173396	6.409
ChEMBL MOLREGNO	179561	7.569
ChEMBL MOLREGNO	188531	9.854
ChEMBL MOLREGNO	111895	9.000
ChEMBL MOLREGNO	117637	6.533
ChEMBL MOLREGNO	127833	9.398
ChEMBL MOLREGNO	14515	6.161
ChEMBL MOLREGNO	61120	7.155
ChEMBL MOLREGNO	69815	9.658
ChEMBL MOLREGNO	70308	8.745
ChEMBL MOLREGNO	11311	8.155
ChEMBL	20222	6.769

MOLREGNO		
ChEMBL MOLREGNO	11323	6.398
ChEMBL MOLREGNO	11042	7.456
ChEMBL MOLREGNO	2038622	9.854
ChEMBL MOLREGNO	119476	9.155
ChEMBL MOLREGNO	51623	7.959
ChEMBL MOLREGNO	112634	6.674
ChEMBL MOLREGNO	333311	10.155
ChEMBL MOLREGNO	333699	8.678
ChEMBL MOLREGNO	282221	8.523
ChEMBL MOLREGNO	270868	6.170
ChEMBL MOLREGNO	204704	6.738
ChEMBL MOLREGNO	203781	6.582
ChEMBL MOLREGNO	40690	7.553
ChEMBL MOLREGNO	95224	7.398
ChEMBL MOLREGNO	179834	6.525
ChEMBL MOLREGNO	184937	6.377
ChEMBL MOLREGNO	205117	7.854
ChEMBL MOLREGNO	96146	7.962
ChEMBL MOLREGNO	112782	7.886
ChEMBL MOLREGNO	161518	6.900
ChEMBL MOLREGNO	231492	7.824
ChEMBL MOLREGNO	157905	9.357
ChEMBL MOLREGNO	157214	7.187
ChEMBL MOLREGNO	350497	9.409

ChEMBL MOLREGNO	1387157	5.068
ChEMBL MOLREGNO	153399	4.602
ChEMBL MOLREGNO	308795	11.000
ChEMBL MOLREGNO	1355730	9.456
ChEMBL MOLREGNO	20220	5.987
ChEMBL MOLREGNO	260770	8.721
ChEMBL MOLREGNO	258115	7.854
ChEMBL MOLREGNO	283725	7.262
ChEMBL MOLREGNO	245826	7.602
ChEMBL MOLREGNO	140947	4.678
ChEMBL MOLREGNO	159731	6.896
ChEMBL MOLREGNO	161387	9.222
ChEMBL MOLREGNO	174938	4.594
ChEMBL MOLREGNO	183247	8.921
ChEMBL MOLREGNO	190997	9.000
ChEMBL MOLREGNO	174059	6.076
ChEMBL MOLREGNO	201565	8.854
ChEMBL MOLREGNO	70113	9.699
ChEMBL MOLREGNO	70307	9.328
ChEMBL MOLREGNO	14504	6.721
ChEMBL MOLREGNO	70553	8.796
ChEMBL MOLREGNO	11252	7.208
ChEMBL MOLREGNO	173502	8.387
ChEMBL MOLREGNO	10998	7.174
ChEMBL	11020	5.848

MOLREGNO		
ChEMBL MOLREGNO	157933	7.638
ChEMBL MOLREGNO	1595418	10.523
ChEMBL MOLREGNO	118100	8.699
ChEMBL MOLREGNO	112583	4.854
ChEMBL MOLREGNO	111758	6.600
ChEMBL MOLREGNO	338232	8.699
ChEMBL MOLREGNO	108731	6.919
ChEMBL MOLREGNO	288863	6.594
ChEMBL MOLREGNO	270244	7.149
ChEMBL MOLREGNO	205518	7.013
ChEMBL MOLREGNO	270308	7.301
ChEMBL MOLREGNO	202001	8.367
ChEMBL MOLREGNO	79940	10.398
ChEMBL MOLREGNO	257648	5.523
ChEMBL MOLREGNO	287516	4.074
ChEMBL MOLREGNO	86682	9.602
ChEMBL MOLREGNO	157385	7.620
ChEMBL MOLREGNO	350312	9.398
ChEMBL MOLREGNO	151671	7.745
ChEMBL MOLREGNO	149195	7.509
ChEMBL MOLREGNO	157022	6.444
ChEMBL MOLREGNO	308792	11.000
ChEMBL MOLREGNO	1355728	9.456
ChEMBL MOLREGNO	149247	7.921

ChEMBL MOLREGNO	149315	8.215
ChEMBL MOLREGNO	144920	4.046
ChEMBL MOLREGNO	201893	7.481
ChEMBL MOLREGNO	257817	8.658
ChEMBL MOLREGNO	282351	7.538
ChEMBL MOLREGNO	235201	6.699
ChEMBL MOLREGNO	152753	8.770
ChEMBL MOLREGNO	163135	6.459
ChEMBL MOLREGNO	162892	6.907
ChEMBL MOLREGNO	112584	8.398
ChEMBL MOLREGNO	117544	7.268
ChEMBL MOLREGNO	95223	7.222
ChEMBL MOLREGNO	95262	6.886
ChEMBL MOLREGNO	11404	6.678
ChEMBL MOLREGNO	11366	8.796
ChEMBL MOLREGNO	110727	6.658
ChEMBL MOLREGNO	128599	9.222
ChEMBL MOLREGNO	11217	8.398
ChEMBL MOLREGNO	11209	7.420
ChEMBL MOLREGNO	10821	6.719
ChEMBL MOLREGNO	197419	5.155
ChEMBL MOLREGNO	51889	8.294
ChEMBL MOLREGNO	115133	7.888
ChEMBL MOLREGNO	1154141	8.086
ChEMBL	110416	4.527

MOLREGNO		
ChEMBL MOLREGNO	282723	8.432
ChEMBL MOLREGNO	1548894	7.398
ChEMBL MOLREGNO	260345	8.027
ChEMBL MOLREGNO	270978	8.959
ChEMBL MOLREGNO	204976	5.642
ChEMBL MOLREGNO	206111	5.301
ChEMBL MOLREGNO	201980	6.523
ChEMBL MOLREGNO	174798	8.420
ChEMBL MOLREGNO	174067	8.125
ChEMBL MOLREGNO	184967	7.828
ChEMBL MOLREGNO	184178	7.638
ChEMBL MOLREGNO	188106	9.456
ChEMBL MOLREGNO	198377	5.620
ChEMBL MOLREGNO	201770	7.066
ChEMBL MOLREGNO	136349	4.420
ChEMBL MOLREGNO	113278	8.734
ChEMBL MOLREGNO	308703	9.620
ChEMBL MOLREGNO	166537	7.886
ChEMBL MOLREGNO	161240	8.523
ChEMBL MOLREGNO	161352	6.611
ChEMBL MOLREGNO	20208	8.186
ChEMBL MOLREGNO	350566	9.252
ChEMBL MOLREGNO	350179	9.398
ChEMBL MOLREGNO	338098	8.699

ChEMBL MOLREGNO	1386836	7.523
ChEMBL MOLREGNO	75778	6.155
ChEMBL MOLREGNO	76089	9.398
ChEMBL MOLREGNO	175056	6.268
ChEMBL MOLREGNO	191572	9.523
ChEMBL MOLREGNO	70808	10.097
ChEMBL MOLREGNO	87141	9.229
ChEMBL MOLREGNO	111820	6.347
ChEMBL MOLREGNO	110235	5.836
ChEMBL MOLREGNO	120742	9.347
ChEMBL MOLREGNO	127871	9.854
ChEMBL MOLREGNO	144751	4.420
ChEMBL MOLREGNO	14674	4.606
ChEMBL MOLREGNO	60424	6.347
ChEMBL MOLREGNO	72912	6.722
ChEMBL MOLREGNO	133095	7.812
ChEMBL MOLREGNO	20623	7.400
ChEMBL MOLREGNO	8267	7.347
ChEMBL MOLREGNO	25291	7.658
ChEMBL MOLREGNO	11133	7.658
ChEMBL MOLREGNO	10826	7.229
ChEMBL MOLREGNO	120021	8.355
ChEMBL MOLREGNO	158389	9.208
ChEMBL MOLREGNO	109285	7.824
ChEMBL	286530	6.387

MOLREGNO		
ChEMBL MOLREGNO	290819	7.208
ChEMBL MOLREGNO	257451	5.495
ChEMBL MOLREGNO	203784	7.009
ChEMBL MOLREGNO	183901	7.658
ChEMBL MOLREGNO	157044	8.469
ChEMBL MOLREGNO	54713	8.796
ChEMBL MOLREGNO	163473	9.481
ChEMBL MOLREGNO	94824	6.495
ChEMBL MOLREGNO	288862	7.100
ChEMBL MOLREGNO	154002	5.000
ChEMBL MOLREGNO	174035	5.319
ChEMBL MOLREGNO	95228	7.310
ChEMBL MOLREGNO	1385959	5.538
ChEMBL MOLREGNO	149104	6.602
ChEMBL MOLREGNO	333312	10.301
ChEMBL MOLREGNO	145551	5.409
ChEMBL MOLREGNO	231426	8.222
ChEMBL MOLREGNO	283045	8.456
ChEMBL MOLREGNO	282248	8.013
ChEMBL MOLREGNO	73116	5.886
ChEMBL MOLREGNO	179396	7.292
ChEMBL MOLREGNO	191071	9.523
ChEMBL MOLREGNO	179454	7.191
ChEMBL MOLREGNO	158456	9.125

ChEMBL MOLREGNO	82226	8.244
ChEMBL MOLREGNO	95000	7.398
ChEMBL MOLREGNO	95426	6.553
ChEMBL MOLREGNO	115134	8.854
ChEMBL MOLREGNO	127832	8.936
ChEMBL MOLREGNO	127425	8.936
ChEMBL MOLREGNO	61081	7.097
ChEMBL MOLREGNO	14615	5.466
ChEMBL MOLREGNO	11415	5.602
ChEMBL MOLREGNO	8444	9.420
ChEMBL MOLREGNO	8511	6.041
ChEMBL MOLREGNO	11116	6.582
ChEMBL MOLREGNO	2038627	10.398
ChEMBL MOLREGNO	120065	7.721
ChEMBL MOLREGNO	120744	9.721
ChEMBL MOLREGNO	51173	8.187
ChEMBL MOLREGNO	117306	6.383
ChEMBL MOLREGNO	350178	9.398
ChEMBL MOLREGNO	108989	8.721
ChEMBL MOLREGNO	281882	8.041
ChEMBL MOLREGNO	283049	8.620
ChEMBL MOLREGNO	245229	7.770
ChEMBL MOLREGNO	249852	8.174
ChEMBL MOLREGNO	269424	8.824
ChEMBL	96493	5.000

MOLREGNO		
ChEMBL MOLREGNO	183358	8.276
ChEMBL MOLREGNO	191120	10.886
ChEMBL MOLREGNO	197916	6.699
ChEMBL MOLREGNO	331040	5.000
ChEMBL MOLREGNO	161477	8.292
ChEMBL MOLREGNO	160417	8.222
ChEMBL MOLREGNO	190646	6.155
ChEMBL MOLREGNO	153634	9.921
ChEMBL MOLREGNO	1798599	6.678
ChEMBL MOLREGNO	82103	9.222
ChEMBL MOLREGNO	149368	8.854
ChEMBL MOLREGNO	148076	7.863
ChEMBL MOLREGNO	149219	6.839
ChEMBL MOLREGNO	220187	8.959
ChEMBL MOLREGNO	142161	7.886
ChEMBL MOLREGNO	179500	7.796
ChEMBL MOLREGNO	157384	7.022
ChEMBL MOLREGNO	179622	7.208
ChEMBL MOLREGNO	179708	7.721
ChEMBL MOLREGNO	191000	6.561
ChEMBL MOLREGNO	173774	7.456
ChEMBL MOLREGNO	145552	5.854
ChEMBL MOLREGNO	205116	7.921
ChEMBL MOLREGNO	201769	6.921

ChEMBL MOLREGNO	86906	9.495
ChEMBL MOLREGNO	80524	9.886
ChEMBL MOLREGNO	111589	6.046
ChEMBL MOLREGNO	119670	9.921
ChEMBL MOLREGNO	128600	9.215
ChEMBL MOLREGNO	145550	6.284
ChEMBL MOLREGNO	14660	4.849
ChEMBL MOLREGNO	14477	5.924
ChEMBL MOLREGNO	70493	9.034
ChEMBL MOLREGNO	14584	5.717
ChEMBL MOLREGNO	90036	7.097
ChEMBL MOLREGNO	86907	9.959
ChEMBL MOLREGNO	96579	7.000
ChEMBL MOLREGNO	11019	7.119
ChEMBL MOLREGNO	20544	7.826
ChEMBL MOLREGNO	11403	4.959
ChEMBL MOLREGNO	10866	6.688
ChEMBL MOLREGNO	121704	8.301
ChEMBL MOLREGNO	2038620	9.886
ChEMBL MOLREGNO	2038626	10.301
ChEMBL MOLREGNO	8375	7.357
ChEMBL MOLREGNO	120631	9.027
ChEMBL MOLREGNO	119996	8.921
ChEMBL MOLREGNO	190749	4.854
ChEMBL	111865	8.013

MOLREGNO		
ChEMBL MOLREGNO	271222	6.996
ChEMBL MOLREGNO	174133	8.060
ChEMBL MOLREGNO	173451	7.678
ChEMBL MOLREGNO	184440	8.046
ChEMBL MOLREGNO	197883	5.827
ChEMBL MOLREGNO	203128	7.268
ChEMBL MOLREGNO	160320	8.000
ChEMBL MOLREGNO	111588	7.886
ChEMBL MOLREGNO	188872	9.569
ChEMBL MOLREGNO	271192	5.613
ChEMBL MOLREGNO	69552	10.097
ChEMBL MOLREGNO	157798	6.754
ChEMBL MOLREGNO	350314	9.620
ChEMBL MOLREGNO	1798603	5.187
ChEMBL MOLREGNO	158457	8.886
ChEMBL MOLREGNO	149444	7.959
ChEMBL MOLREGNO	235199	6.377
ChEMBL MOLREGNO	287011	7.722
ChEMBL MOLREGNO	286627	6.866
ChEMBL MOLREGNO	236470	7.796
ChEMBL MOLREGNO	149409	8.585
ChEMBL MOLREGNO	147086	8.310
ChEMBL MOLREGNO	160349	8.736
ChEMBL MOLREGNO	188237	9.167

ChEMBL MOLREGNO	173486	7.398
ChEMBL MOLREGNO	69505	9.770
ChEMBL MOLREGNO	70181	7.203
ChEMBL MOLREGNO	80953	10.038
ChEMBL MOLREGNO	95261	6.398
ChEMBL MOLREGNO	109095	7.208
ChEMBL MOLREGNO	120634	9.553
ChEMBL MOLREGNO	120138	8.585
ChEMBL MOLREGNO	115327	8.921
ChEMBL MOLREGNO	14818	6.602
ChEMBL MOLREGNO	24173	9.149
ChEMBL MOLREGNO	60205	7.523
ChEMBL MOLREGNO	127831	9.432
ChEMBL MOLREGNO	14640	6.201
ChEMBL MOLREGNO	20209	8.959
ChEMBL MOLREGNO	11168	6.943
ChEMBL MOLREGNO	11367	8.770
ChEMBL MOLREGNO	8220	7.071
ChEMBL MOLREGNO	61244	6.824
ChEMBL MOLREGNO	60423	6.071
ChEMBL MOLREGNO	149121	7.638
ChEMBL MOLREGNO	332964	10.097
ChEMBL MOLREGNO	338491	9.357
ChEMBL MOLREGNO	108139	5.398
ChEMBL	282634	8.201

MOLREGNO		
ChEMBL MOLREGNO	284434	5.097
ChEMBL MOLREGNO	290823	7.027
ChEMBL MOLREGNO	281798	8.886
ChEMBL MOLREGNO	265673	4.699
ChEMBL MOLREGNO	205739	7.753
ChEMBL MOLREGNO	231495	8.301
ChEMBL MOLREGNO	236097	5.523
ChEMBL MOLREGNO	231667	6.432
ChEMBL MOLREGNO	173753	8.161
ChEMBL MOLREGNO	174228	7.824
ChEMBL MOLREGNO	184073	7.367
ChEMBL MOLREGNO	183989	8.780
ChEMBL MOLREGNO	197794	6.481
ChEMBL MOLREGNO	94841	6.523
ChEMBL MOLREGNO	8383	7.833
ChEMBL MOLREGNO	157946	9.824
ChEMBL MOLREGNO	90448	7.022
ChEMBL MOLREGNO	8510	8.638
ChEMBL MOLREGNO	157277	9.328
ChEMBL MOLREGNO	161075	5.824
ChEMBL MOLREGNO	153156	10.523
ChEMBL MOLREGNO	153635	10.301
ChEMBL MOLREGNO	338523	8.602
ChEMBL MOLREGNO	350313	9.301

ChEMBL MOLREGNO	330995	6.276
ChEMBL MOLREGNO	350498	9.201
ChEMBL MOLREGNO	1383924	5.302
ChEMBL MOLREGNO	82102	8.886
ChEMBL MOLREGNO	330949	6.161
ChEMBL MOLREGNO	20096	8.075
ChEMBL MOLREGNO	149139	8.347
ChEMBL MOLREGNO	174723	8.301
ChEMBL MOLREGNO	153155	9.854
ChEMBL MOLREGNO	183699	8.854
ChEMBL MOLREGNO	183417	8.796
ChEMBL MOLREGNO	183964	6.745
ChEMBL MOLREGNO	190989	6.460
ChEMBL MOLREGNO	188445	9.959
ChEMBL MOLREGNO	201981	7.585
ChEMBL MOLREGNO	69871	9.292
ChEMBL MOLREGNO	69508	9.770
ChEMBL MOLREGNO	71038	9.367
ChEMBL MOLREGNO	80454	9.796
ChEMBL MOLREGNO	116627	6.431
ChEMBL MOLREGNO	119659	10.149
ChEMBL MOLREGNO	179737	7.481
ChEMBL MOLREGNO	24721	8.854
ChEMBL MOLREGNO	51783	9.264
ChEMBL	11349	5.854

MOLREGNO		
ChEMBL MOLREGNO	8497	7.757
ChEMBL MOLREGNO	11277	8.745
ChEMBL MOLREGNO	11087	7.268
ChEMBL MOLREGNO	17170	9.120
ChEMBL MOLREGNO	8532	9.357
ChEMBL MOLREGNO	11351	8.585
ChEMBL MOLREGNO	61113	7.125
ChEMBL MOLREGNO	139520	7.569
ChEMBL MOLREGNO	51153	7.952
ChEMBL MOLREGNO	119590	9.108
ChEMBL MOLREGNO	350660	9.292
ChEMBL MOLREGNO	282392	7.738
ChEMBL MOLREGNO	260216	8.921
ChEMBL MOLREGNO	95004	6.854
ChEMBL MOLREGNO	202313	6.187
ChEMBL MOLREGNO	230910	7.849
ChEMBL MOLREGNO	238987	4.481
ChEMBL MOLREGNO	94965	7.222
ChEMBL MOLREGNO	174065	7.959
ChEMBL MOLREGNO	183847	8.523
ChEMBL MOLREGNO	201982	7.638
ChEMBL MOLREGNO	202743	7.678
ChEMBL MOLREGNO	161480	8.921
ChEMBL MOLREGNO	160473	9.085

ChEMBL MOLREGNO	166688	8.469
ChEMBL MOLREGNO	161479	8.770
ChEMBL MOLREGNO	97280	6.538
ChEMBL MOLREGNO	117638	6.170
ChEMBL MOLREGNO	271764	6.631
ChEMBL MOLREGNO	245735	7.649
ChEMBL MOLREGNO	163347	8.914
ChEMBL MOLREGNO	190748	4.854
ChEMBL MOLREGNO	1368167	6.561
ChEMBL MOLREGNO	25217	8.745
ChEMBL MOLREGNO	204932	6.411
ChEMBL MOLREGNO	204902	7.137
ChEMBL MOLREGNO	232221	8.222
ChEMBL MOLREGNO	281800	8.174
ChEMBL MOLREGNO	249958	8.932
ChEMBL MOLREGNO	174939	6.824
ChEMBL MOLREGNO	156775	7.553
ChEMBL MOLREGNO	188532	8.959
ChEMBL MOLREGNO	177521	7.700
ChEMBL MOLREGNO	191571	9.509
ChEMBL MOLREGNO	142561	7.187
ChEMBL MOLREGNO	121718	7.155
ChEMBL MOLREGNO	145395	4.886
ChEMBL MOLREGNO	95177	7.097
ChEMBL	69551	10.046

MOLREGNO		
ChEMBL MOLREGNO	8445	7.908
ChEMBL MOLREGNO	14619	6.310
ChEMBL MOLREGNO	11436	5.377
ChEMBL MOLREGNO	1627518	5.824
ChEMBL MOLREGNO	158238	7.222
ChEMBL MOLREGNO	1594682	5.569
ChEMBL MOLREGNO	2038629	10.523
ChEMBL MOLREGNO	117545	7.108
ChEMBL MOLREGNO	110552	4.573
ChEMBL MOLREGNO	283048	8.301
ChEMBL MOLREGNO	304750	4.000
ChEMBL MOLREGNO	1548891	9.102
ChEMBL MOLREGNO	246117	7.959
ChEMBL MOLREGNO	270624	6.174
ChEMBL MOLREGNO	281727	8.387
ChEMBL MOLREGNO	282746	8.444
ChEMBL MOLREGNO	204522	6.924
ChEMBL MOLREGNO	204811	7.770
ChEMBL MOLREGNO	184000	7.886
ChEMBL MOLREGNO	184164	8.658
ChEMBL MOLREGNO	202631	5.292
ChEMBL MOLREGNO	204964	5.921
ChEMBL MOLREGNO	90491	7.013
ChEMBL MOLREGNO	308793	10.699

ChEMBL MOLREGNO	191002	5.155
ChEMBL MOLREGNO	161077	6.286
ChEMBL MOLREGNO	269425	7.721
ChEMBL MOLREGNO	158236	9.337
ChEMBL MOLREGNO	350169	9.367
ChEMBL MOLREGNO	59730	6.398
ChEMBL MOLREGNO	283011	8.523
ChEMBL MOLREGNO	20095	7.952
ChEMBL MOLREGNO	157485	9.699
ChEMBL MOLREGNO	179741	7.432
ChEMBL MOLREGNO	185014	10.398
ChEMBL MOLREGNO	149499	8.481
ChEMBL MOLREGNO	156774	7.301
ChEMBL MOLREGNO	72866	6.057
ChEMBL MOLREGNO	81356	10.155
ChEMBL MOLREGNO	86115	9.119
ChEMBL MOLREGNO	110992	6.420
ChEMBL MOLREGNO	117394	6.322
ChEMBL MOLREGNO	115667	8.638
ChEMBL MOLREGNO	136602	5.959
ChEMBL MOLREGNO	14626	6.066
ChEMBL MOLREGNO	282610	8.745
ChEMBL MOLREGNO	70651	9.444
ChEMBL MOLREGNO	95497	6.699
ChEMBL	86663	10.000

MOLREGNO		
ChEMBL MOLREGNO	8368	9.602
ChEMBL MOLREGNO	11394	6.830
ChEMBL MOLREGNO	8315	7.137
ChEMBL MOLREGNO	2038628	10.699
ChEMBL MOLREGNO	121483	7.886
ChEMBL MOLREGNO	1154139	8.523
ChEMBL MOLREGNO	1154397	9.602
ChEMBL MOLREGNO	110081	4.412
ChEMBL MOLREGNO	282475	8.699
ChEMBL MOLREGNO	285935	6.523
ChEMBL MOLREGNO	282240	8.658
ChEMBL MOLREGNO	282671	8.678
ChEMBL MOLREGNO	1548895	8.187
ChEMBL MOLREGNO	257485	6.420
ChEMBL MOLREGNO	95015	7.000
ChEMBL MOLREGNO	184098	8.319
ChEMBL MOLREGNO	179376	7.387
ChEMBL MOLREGNO	183808	8.125
ChEMBL MOLREGNO	183811	8.000
ChEMBL MOLREGNO	201417	8.398
ChEMBL MOLREGNO	20795	6.441
ChEMBL MOLREGNO	161609	6.979
ChEMBL MOLREGNO	90350	8.770
ChEMBL MOLREGNO	8369	7.377

ChEMBL MOLREGNO	157943	6.218
ChEMBL MOLREGNO	153527	5.155
ChEMBL MOLREGNO	350661	9.284
ChEMBL MOLREGNO	81190	10.155
ChEMBL MOLREGNO	80954	10.523
ChEMBL MOLREGNO	149273	8.481
ChEMBL MOLREGNO	287453	6.319
ChEMBL MOLREGNO	145351	5.155
ChEMBL MOLREGNO	145662	5.194
ChEMBL MOLREGNO	257851	6.222
ChEMBL MOLREGNO	268250	4.745
ChEMBL MOLREGNO	281881	8.444
ChEMBL MOLREGNO	282353	8.585
ChEMBL MOLREGNO	249985	9.658
ChEMBL MOLREGNO	197915	5.491
ChEMBL MOLREGNO	72913	6.611
ChEMBL MOLREGNO	74829	5.222
ChEMBL MOLREGNO	148049	7.461
ChEMBL MOLREGNO	142139	7.824
ChEMBL MOLREGNO	160867	8.301
ChEMBL MOLREGNO	161148	8.678
ChEMBL MOLREGNO	174901	7.268
ChEMBL MOLREGNO	188873	9.495
ChEMBL MOLREGNO	179476	7.638
ChEMBL	160415	7.071

MOLREGNO		
ChEMBL MOLREGNO	201163	7.824
ChEMBL MOLREGNO	202101	6.347
ChEMBL MOLREGNO	75247	5.347
ChEMBL MOLREGNO	112820	8.208
ChEMBL MOLREGNO	82149	7.924
ChEMBL MOLREGNO	8465	8.721
ChEMBL MOLREGNO	8221	6.491
ChEMBL MOLREGNO	11018	7.638
ChEMBL MOLREGNO	120935	7.854
ChEMBL MOLREGNO	156967	9.222
ChEMBL MOLREGNO	282676	7.910
ChEMBL MOLREGNO	121626	8.886
ChEMBL MOLREGNO	112253	8.310
ChEMBL MOLREGNO	108404	7.237
ChEMBL MOLREGNO	333379	9.959
ChEMBL MOLREGNO	112588	8.409
ChEMBL MOLREGNO	281728	8.347
ChEMBL MOLREGNO	282296	7.678
ChEMBL MOLREGNO	304681	4.301
ChEMBL MOLREGNO	282724	7.772
ChEMBL MOLREGNO	204836	6.696
ChEMBL MOLREGNO	236451	4.125
ChEMBL MOLREGNO	236471	6.699
ChEMBL MOLREGNO	173797	6.086

ChEMBL MOLREGNO	160720	8.796
ChEMBL MOLREGNO	157159	8.762
ChEMBL MOLREGNO	157475	8.824
ChEMBL MOLREGNO	308791	10.699
ChEMBL MOLREGNO	149260	7.292
ChEMBL MOLREGNO	235866	5.824
ChEMBL MOLREGNO	249798	8.658
ChEMBL MOLREGNO	249933	9.585
ChEMBL MOLREGNO	174856	7.276
ChEMBL MOLREGNO	184160	7.020
ChEMBL MOLREGNO	185119	7.000
ChEMBL MOLREGNO	187893	8.770
ChEMBL MOLREGNO	160416	8.745
ChEMBL MOLREGNO	148716	7.319
ChEMBL MOLREGNO	191138	8.215
ChEMBL MOLREGNO	197795	6.097
ChEMBL MOLREGNO	70135	7.460
ChEMBL MOLREGNO	86515	10.398
ChEMBL MOLREGNO	74907	6.398
ChEMBL MOLREGNO	112586	8.585
ChEMBL MOLREGNO	135942	6.530
ChEMBL MOLREGNO	8623	7.712
ChEMBL MOLREGNO	271016	6.907
ChEMBL MOLREGNO	11117	7.824
ChEMBL	1627274	5.824

MOLREGNO		
ChEMBL MOLREGNO	11352	5.959
ChEMBL MOLREGNO	120803	9.244
ChEMBL MOLREGNO	24722	7.872
ChEMBL MOLREGNO	121871	8.796
ChEMBL MOLREGNO	51457	8.215
ChEMBL MOLREGNO	350495	9.921
ChEMBL MOLREGNO	148838	7.979
ChEMBL MOLREGNO	282532	8.658
ChEMBL MOLREGNO	249799	9.886
ChEMBL MOLREGNO	258080	6.721
ChEMBL MOLREGNO	203816	7.638
ChEMBL MOLREGNO	204312	6.471
ChEMBL MOLREGNO	231777	6.659
ChEMBL MOLREGNO	171672	8.523
ChEMBL MOLREGNO	129686	8.222
ChEMBL MOLREGNO	350097	9.398
ChEMBL MOLREGNO	160870	7.886
ChEMBL MOLREGNO	271183	6.249
ChEMBL MOLREGNO	25377	7.824
ChEMBL MOLREGNO	27521	5.046
ChEMBL MOLREGNO	157021	6.301
ChEMBL MOLREGNO	157023	7.523
ChEMBL MOLREGNO	270456	8.215
ChEMBL MOLREGNO	270188	8.959

ChEMBL MOLREGNO	158319	10.155
ChEMBL MOLREGNO	166418	5.194
ChEMBL MOLREGNO	183844	7.367
ChEMBL MOLREGNO	185867	10.398
ChEMBL MOLREGNO	148669	8.081
ChEMBL MOLREGNO	158164	7.585
ChEMBL MOLREGNO	201924	8.167
ChEMBL MOLREGNO	191168	9.886
ChEMBL MOLREGNO	70109	9.000
ChEMBL MOLREGNO	70005	9.640
ChEMBL MOLREGNO	111619	8.097
ChEMBL MOLREGNO	114785	8.824
ChEMBL MOLREGNO	108618	6.854
ChEMBL MOLREGNO	117264	6.879
ChEMBL MOLREGNO	118145	7.161
ChEMBL MOLREGNO	14659	5.211
ChEMBL MOLREGNO	51329	7.870
ChEMBL MOLREGNO	60966	6.060
ChEMBL MOLREGNO	70197	8.921
ChEMBL MOLREGNO	71575	6.585
ChEMBL MOLREGNO	149157	8.469
ChEMBL MOLREGNO	8384	7.699
ChEMBL MOLREGNO	121868	8.237
ChEMBL MOLREGNO	119657	9.409
ChEMBL	117266	6.991

MOLREGNO		
ChEMBL MOLREGNO	121484	7.721
ChEMBL MOLREGNO	60176	5.921
ChEMBL MOLREGNO	117639	6.019
ChEMBL MOLREGNO	350133	9.301
ChEMBL MOLREGNO	110726	5.722
ChEMBL MOLREGNO	249271	9.027
ChEMBL MOLREGNO	257676	7.569
ChEMBL MOLREGNO	265713	4.097
ChEMBL MOLREGNO	271157	5.956
ChEMBL MOLREGNO	271113	6.896
ChEMBL MOLREGNO	271047	7.585
ChEMBL MOLREGNO	205566	6.569
ChEMBL MOLREGNO	205544	5.051
ChEMBL MOLREGNO	219315	6.796
ChEMBL MOLREGNO	184175	7.870
ChEMBL MOLREGNO	202117	7.921
ChEMBL MOLREGNO	197449	5.784
ChEMBL MOLREGNO	197949	5.222
ChEMBL MOLREGNO	111587	7.056
ChEMBL MOLREGNO	174229	7.721
ChEMBL MOLREGNO	159985	8.180
ChEMBL MOLREGNO	173503	9.638
ChEMBL MOLREGNO	61608	7.523
ChEMBL MOLREGNO	119440	7.168

ChEMBL MOLREGNO	149175	8.276
ChEMBL MOLREGNO	149221	7.174
ChEMBL MOLREGNO	235747	6.887
ChEMBL MOLREGNO	260427	8.638
ChEMBL MOLREGNO	257649	7.347
ChEMBL MOLREGNO	286634	5.107
ChEMBL MOLREGNO	75589	7.000
ChEMBL MOLREGNO	145396	4.585
ChEMBL MOLREGNO	173606	6.086
ChEMBL MOLREGNO	179353	8.071
ChEMBL MOLREGNO	174902	7.022
ChEMBL MOLREGNO	149086	8.387
ChEMBL MOLREGNO	183597	8.796
ChEMBL MOLREGNO	138762	8.398
ChEMBL MOLREGNO	111863	8.367
ChEMBL MOLREGNO	90490	6.559
ChEMBL MOLREGNO	609544	5.240
ChEMBL MOLREGNO	14617	4.714
ChEMBL MOLREGNO	11310	7.699
ChEMBL MOLREGNO	20879	6.022
ChEMBL MOLREGNO	11395	6.821
ChEMBL MOLREGNO	118123	6.317
ChEMBL MOLREGNO	51747	8.569
ChEMBL MOLREGNO	350177	9.187
ChEMBL	51191	8.114

MOLREGNO		
ChEMBL MOLREGNO	112637	8.509
ChEMBL MOLREGNO	282732	8.721
ChEMBL MOLREGNO	258176	6.678
ChEMBL MOLREGNO	270221	7.854
ChEMBL MOLREGNO	270172	7.092
ChEMBL MOLREGNO	270173	6.836
ChEMBL MOLREGNO	97358	7.131
ChEMBL MOLREGNO	174168	7.143
ChEMBL MOLREGNO	174036	6.796
ChEMBL MOLREGNO	198192	5.509
ChEMBL MOLREGNO	201853	8.495
ChEMBL MOLREGNO	201950	6.921
ChEMBL MOLREGNO	204550	7.420
ChEMBL MOLREGNO	166472	8.018
ChEMBL MOLREGNO	183965	8.523
ChEMBL MOLREGNO	174737	6.523
ChEMBL MOLREGNO	128295	9.301
ChEMBL MOLREGNO	152875	10.523
ChEMBL MOLREGNO	157799	7.538
ChEMBL MOLREGNO	161576	7.721
ChEMBL MOLREGNO	157524	8.284
ChEMBL MOLREGNO	161534	9.678
ChEMBL MOLREGNO	350565	9.481
ChEMBL MOLREGNO	1798120	8.509

ChEMBL MOLREGNO	289086	6.387
ChEMBL MOLREGNO	20133	7.722
ChEMBL MOLREGNO	148536	7.567
ChEMBL MOLREGNO	204948	5.900
ChEMBL MOLREGNO	228501	6.571
ChEMBL MOLREGNO	287497	6.648
ChEMBL MOLREGNO	148048	7.244
ChEMBL MOLREGNO	166473	6.066
ChEMBL MOLREGNO	188209	8.469
ChEMBL MOLREGNO	185228	10.000
ChEMBL MOLREGNO	75446	5.071
ChEMBL MOLREGNO	112768	8.000
ChEMBL MOLREGNO	112580	8.319
ChEMBL MOLREGNO	118121	6.896
ChEMBL MOLREGNO	51154	8.420
ChEMBL MOLREGNO	1632644	8.222
ChEMBL MOLREGNO	8382	7.222
ChEMBL MOLREGNO	121830	9.097
ChEMBL MOLREGNO	112730	7.886
ChEMBL MOLREGNO	173605	7.328
ChEMBL MOLREGNO	112587	8.523
ChEMBL MOLREGNO	338524	7.018
ChEMBL MOLREGNO	282747	7.662
ChEMBL MOLREGNO	286626	7.076
ChEMBL	1548892	7.921

MOLREGNO		
ChEMBL MOLREGNO	281822	8.658
ChEMBL MOLREGNO	282220	8.824
ChEMBL MOLREGNO	231454	7.310
ChEMBL MOLREGNO	197685	4.569
ChEMBL MOLREGNO	90037	8.328
ChEMBL MOLREGNO	121021	7.745
ChEMBL MOLREGNO	333001	10.523
ChEMBL MOLREGNO	149410	8.125
ChEMBL MOLREGNO	284363	6.707
ChEMBL MOLREGNO	282476	8.060
ChEMBL MOLREGNO	20010	6.983
ChEMBL MOLREGNO	145036	5.638
ChEMBL MOLREGNO	161505	8.745
ChEMBL MOLREGNO	185016	10.155
ChEMBL MOLREGNO	151672	8.854
ChEMBL MOLREGNO	157396	4.301
ChEMBL MOLREGNO	205361	5.678
ChEMBL MOLREGNO	108444	8.602
ChEMBL MOLREGNO	119658	9.796
ChEMBL MOLREGNO	14664	6.000
ChEMBL MOLREGNO	14635	5.069
ChEMBL MOLREGNO	14589	5.690
ChEMBL MOLREGNO	8276	7.292
ChEMBL MOLREGNO	11389	6.585

ChEMBL MOLREGNO	2038619	10.398
ChEMBL MOLREGNO	119953	8.456
ChEMBL MOLREGNO	59728	8.301
ChEMBL MOLREGNO	119584	9.959
ChEMBL MOLREGNO	111790	8.222
ChEMBL MOLREGNO	350098	8.824
ChEMBL MOLREGNO	282300	8.770
ChEMBL MOLREGNO	108443	8.658
ChEMBL MOLREGNO	173876	7.409
ChEMBL MOLREGNO	177045	8.155
ChEMBL MOLREGNO	204387	5.886
ChEMBL MOLREGNO	203129	5.796
ChEMBL MOLREGNO	202063	8.432
ChEMBL MOLREGNO	174007	8.125
ChEMBL MOLREGNO	161386	7.620
ChEMBL MOLREGNO	271791	7.041
ChEMBL MOLREGNO	157213	7.481
ChEMBL MOLREGNO	157476	8.678
ChEMBL MOLREGNO	70653	9.367
ChEMBL MOLREGNO	157383	8.000
ChEMBL MOLREGNO	160756	8.699
ChEMBL MOLREGNO	172992	8.268
ChEMBL MOLREGNO	174368	6.404
ChEMBL MOLREGNO	187796	9.721
ChEMBL	187822	9.409

MOLREGNO		
ChEMBL MOLREGNO	153550	4.000
ChEMBL MOLREGNO	204394	6.292
ChEMBL MOLREGNO	145503	4.066
ChEMBL MOLREGNO	144785	5.398
ChEMBL MOLREGNO	120555	8.854
ChEMBL MOLREGNO	96347	7.466
ChEMBL MOLREGNO	14639	7.469
ChEMBL MOLREGNO	8498	6.770
ChEMBL MOLREGNO	11214	7.097
ChEMBL MOLREGNO	231809	8.523
ChEMBL MOLREGNO	113087	8.523
ChEMBL MOLREGNO	112769	8.155
ChEMBL MOLREGNO	333638	8.420
ChEMBL MOLREGNO	109284	7.409
ChEMBL MOLREGNO	282609	8.387
ChEMBL MOLREGNO	290818	7.222
ChEMBL MOLREGNO	258209	5.000
ChEMBL MOLREGNO	265633	4.523
ChEMBL MOLREGNO	203783	7.434
ChEMBL MOLREGNO	205004	8.301
ChEMBL MOLREGNO	205320	7.770
ChEMBL MOLREGNO	231748	7.854
ChEMBL MOLREGNO	173933	5.886
ChEMBL MOLREGNO	174204	8.004

ChEMBL MOLREGNO	180429	4.237
ChEMBL MOLREGNO	187368	9.959
ChEMBL MOLREGNO	161478	9.301
ChEMBL MOLREGNO	156773	4.284
ChEMBL MOLREGNO	159802	7.886
ChEMBL MOLREGNO	42767	8.097
ChEMBL MOLREGNO	149622	8.796
ChEMBL MOLREGNO	156467	8.046
ChEMBL MOLREGNO	333553	8.102
ChEMBL MOLREGNO	260164	8.922
ChEMBL MOLREGNO	75737	6.301
ChEMBL MOLREGNO	141628	7.651
ChEMBL MOLREGNO	153157	9.959
ChEMBL MOLREGNO	166689	7.921
ChEMBL MOLREGNO	190996	9.222
ChEMBL MOLREGNO	188355	7.585
ChEMBL MOLREGNO	69823	9.569
ChEMBL MOLREGNO	110765	4.432
ChEMBL MOLREGNO	14695	5.222
ChEMBL MOLREGNO	19705	4.553
ChEMBL MOLREGNO	1627598	6.456
ChEMBL MOLREGNO	127805	9.187
ChEMBL MOLREGNO	127872	10.155
ChEMBL MOLREGNO	115328	8.745
ChEMBL	282189	7.733

MOLREGNO		
ChEMBL MOLREGNO	289135	5.613
ChEMBL MOLREGNO	260064	8.796
ChEMBL MOLREGNO	266212	4.000
ChEMBL MOLREGNO	203780	6.818
ChEMBL MOLREGNO	231833	6.721
ChEMBL MOLREGNO	40716	6.827
ChEMBL MOLREGNO	174058	6.854
ChEMBL MOLREGNO	190851	9.092
ChEMBL MOLREGNO	198024	5.317
ChEMBL MOLREGNO	95128	7.000
ChEMBL MOLREGNO	82490	8.000
ChEMBL MOLREGNO	20210	7.801
ChEMBL MOLREGNO	185015	10.523
ChEMBL MOLREGNO	86681	8.444
ChEMBL MOLREGNO	161076	6.027
ChEMBL MOLREGNO	161535	9.377
ChEMBL MOLREGNO	350100	9.357
ChEMBL MOLREGNO	80955	9.620
ChEMBL MOLREGNO	158047	6.114
ChEMBL MOLREGNO	204428	6.959
ChEMBL MOLREGNO	235236	5.959
ChEMBL MOLREGNO	282646	8.398
ChEMBL MOLREGNO	281989	8.018
ChEMBL MOLREGNO	284067	8.000

ChEMBL MOLREGNO	75444	6.097
ChEMBL MOLREGNO	135334	8.268
ChEMBL MOLREGNO	153340	10.046
ChEMBL MOLREGNO	174882	6.602
ChEMBL MOLREGNO	179377	7.509
ChEMBL MOLREGNO	153973	4.155
ChEMBL MOLREGNO	197948	5.796
ChEMBL MOLREGNO	70810	9.886
ChEMBL MOLREGNO	95427	7.046
ChEMBL MOLREGNO	14625	6.161
ChEMBL MOLREGNO	14587	6.004
ChEMBL MOLREGNO	59766	8.000
ChEMBL MOLREGNO	20878	8.071
ChEMBL MOLREGNO	61624	7.523
ChEMBL MOLREGNO	121625	9.620
ChEMBL MOLREGNO	118122	6.815
ChEMBL MOLREGNO	120022	10.222
ChEMBL MOLREGNO	112843	8.796
ChEMBL MOLREGNO	108996	8.538
ChEMBL MOLREGNO	282786	8.481
ChEMBL MOLREGNO	282283	8.097
ChEMBL MOLREGNO	289129	5.611
ChEMBL MOLREGNO	249269	8.174
ChEMBL MOLREGNO	257849	6.456
ChEMBL	40715	8.563

MOLREGNO		
ChEMBL MOLREGNO	179749	6.785
ChEMBL MOLREGNO	188208	9.310
ChEMBL MOLREGNO	188178	8.770
ChEMBL MOLREGNO	90395	6.979
ChEMBL MOLREGNO	90469	5.921
ChEMBL MOLREGNO	87143	9.328
ChEMBL MOLREGNO	179489	6.752
ChEMBL MOLREGNO	51370	8.292
ChEMBL MOLREGNO	14675	5.670
ChEMBL MOLREGNO	159730	9.523
ChEMBL MOLREGNO	81133	9.284
ChEMBL MOLREGNO	350099	9.367
ChEMBL MOLREGNO	333376	10.398
ChEMBL MOLREGNO	148692	8.276
ChEMBL MOLREGNO	270216	6.666
ChEMBL MOLREGNO	145198	5.770
ChEMBL MOLREGNO	145352	5.398
ChEMBL MOLREGNO	152876	9.319
ChEMBL MOLREGNO	185229	10.523
ChEMBL MOLREGNO	191570	10.222
ChEMBL MOLREGNO	158455	9.161
ChEMBL MOLREGNO	161241	7.721
ChEMBL MOLREGNO	188446	8.770
ChEMBL MOLREGNO	202875	6.149

ChEMBL MOLREGNO	204771	5.638
ChEMBL MOLREGNO	69507	9.678
ChEMBL MOLREGNO	96179	5.301
ChEMBL MOLREGNO	136680	4.201
ChEMBL MOLREGNO	14787	6.102
ChEMBL MOLREGNO	73266	6.886
ChEMBL MOLREGNO	19673	8.301
ChEMBL MOLREGNO	70811	9.796
ChEMBL MOLREGNO	11013	6.648
ChEMBL MOLREGNO	8596	9.038
ChEMBL MOLREGNO	5791	5.523
ChEMBL MOLREGNO	51390	8.357
ChEMBL MOLREGNO	51651	8.125
ChEMBL MOLREGNO	350311	9.046
ChEMBL MOLREGNO	112435	8.000
ChEMBL MOLREGNO	333377	10.523
ChEMBL MOLREGNO	286635	6.409
ChEMBL MOLREGNO	249826	7.815
ChEMBL MOLREGNO	258113	7.000
ChEMBL MOLREGNO	205318	5.292
ChEMBL MOLREGNO	184176	8.796
ChEMBL MOLREGNO	191119	9.796
ChEMBL MOLREGNO	90348	8.222
ChEMBL MOLREGNO	205160	6.810
ChEMBL	1799485	8.398

MOLREGNO		
ChEMBL MOLREGNO	1799616	6.229
ChEMBL MOLREGNO	204793	7.292
ChEMBL MOLREGNO	282722	8.456
ChEMBL MOLREGNO	245381	8.745
ChEMBL MOLREGNO	145619	4.357
ChEMBL MOLREGNO	149443	8.119
ChEMBL MOLREGNO	184965	7.886
ChEMBL MOLREGNO	188591	9.602
ChEMBL MOLREGNO	174027	5.056
ChEMBL MOLREGNO	120632	8.824
ChEMBL MOLREGNO	73266	6.921
ChEMBL MOLREGNO	8470	9.114
ChEMBL MOLREGNO	120990	8.886
ChEMBL MOLREGNO	121019	9.022
ChEMBL MOLREGNO	8533	8.244
ChEMBL MOLREGNO	2038621	10.699
ChEMBL MOLREGNO	120633	7.770
ChEMBL MOLREGNO	52060	7.959
ChEMBL MOLREGNO	338225	7.688
ChEMBL MOLREGNO	350170	9.337
ChEMBL MOLREGNO	285843	7.097
ChEMBL MOLREGNO	270217	6.368
ChEMBL MOLREGNO	271084	6.545
ChEMBL MOLREGNO	271150	6.234

ChEMBL MOLREGNO	204724	7.018
ChEMBL MOLREGNO	183604	8.398
ChEMBL MOLREGNO	89976	6.538
ChEMBL MOLREGNO	333635	8.215
ChEMBL MOLREGNO	350564	9.276
ChEMBL MOLREGNO	86940	9.222
ChEMBL MOLREGNO	157156	8.959
ChEMBL MOLREGNO	1799705	8.638
ChEMBL MOLREGNO	282787	8.444
ChEMBL MOLREGNO	288564	6.159
ChEMBL MOLREGNO	144888	4.149
ChEMBL MOLREGNO	161538	6.585
ChEMBL MOLREGNO	191139	8.987
ChEMBL MOLREGNO	184162	8.824
ChEMBL MOLREGNO	179511	7.310
ChEMBL MOLREGNO	201227	8.180
ChEMBL MOLREGNO	70106	8.939
ChEMBL MOLREGNO	121013	9.009
ChEMBL MOLREGNO	14648	6.585
ChEMBL MOLREGNO	61451	7.155
ChEMBL MOLREGNO	59729	8.000
ChEMBL MOLREGNO	70271	9.699
ChEMBL MOLREGNO	8501	7.658
ChEMBL MOLREGNO	8380	7.833
ChEMBL	8509	8.678

MOLREGNO		
ChEMBL MOLREGNO	20216	6.910
ChEMBL MOLREGNO	11426	8.076
ChEMBL MOLREGNO	161533	9.149
ChEMBL MOLREGNO	121476	9.387
ChEMBL MOLREGNO	121666	9.456
ChEMBL MOLREGNO	52458	8.722
ChEMBL MOLREGNO	112157	8.000
ChEMBL MOLREGNO	111864	7.585
ChEMBL MOLREGNO	283044	8.721
ChEMBL MOLREGNO	282327	8.009
ChEMBL MOLREGNO	270623	6.301
ChEMBL MOLREGNO	238986	4.793
ChEMBL MOLREGNO	97318	6.710
ChEMBL MOLREGNO	173775	5.108
ChEMBL MOLREGNO	184059	7.854
ChEMBL MOLREGNO	185868	10.097
ChEMBL MOLREGNO	202109	5.666
ChEMBL MOLREGNO	11134	7.081
ChEMBL MOLREGNO	157158	9.337
ChEMBL MOLREGNO	333314	10.699
ChEMBL MOLREGNO	145530	6.174
ChEMBL MOLREGNO	205230	7.310
ChEMBL MOLREGNO	282794	7.983
ChEMBL MOLREGNO	245230	6.955

ChEMBL MOLREGNO	75736	6.046
ChEMBL MOLREGNO	166864	6.796
ChEMBL MOLREGNO	179395	7.174
ChEMBL MOLREGNO	147988	8.420
ChEMBL MOLREGNO	201781	7.509
ChEMBL MOLREGNO	70170	9.357
ChEMBL MOLREGNO	70652	9.229
ChEMBL MOLREGNO	108935	8.886
ChEMBL MOLREGNO	113046	8.222
ChEMBL MOLREGNO	120517	9.143
ChEMBL MOLREGNO	52167	7.041
ChEMBL MOLREGNO	89940	5.796
ChEMBL MOLREGNO	69553	10.155
ChEMBL MOLREGNO	70703	9.770
ChEMBL MOLREGNO	14819	6.131
ChEMBL MOLREGNO	14564	5.270
ChEMBL MOLREGNO	8468	8.620
ChEMBL MOLREGNO	2038623	10.000
ChEMBL MOLREGNO	112110	8.051
ChEMBL MOLREGNO	284675	6.699
ChEMBL MOLREGNO	286001	6.585
ChEMBL MOLREGNO	289252	6.108
ChEMBL MOLREGNO	287496	6.072
ChEMBL MOLREGNO	309789	4.489
ChEMBL	249957	8.921

MOLREGNO		
ChEMBL MOLREGNO	235200	7.456
ChEMBL MOLREGNO	171591	7.222
ChEMBL MOLREGNO	179623	7.377
ChEMBL MOLREGNO	204985	5.854
ChEMBL MOLREGNO	245946	6.276
ChEMBL MOLREGNO	157896	8.149
ChEMBL MOLREGNO	161044	9.658
ChEMBL MOLREGNO	160869	8.523
ChEMBL MOLREGNO	330912	5.347
ChEMBL MOLREGNO	332969	10.523
ChEMBL MOLREGNO	1355751	10.523
ChEMBL MOLREGNO	148668	8.276
ChEMBL MOLREGNO	232166	7.569
ChEMBL MOLREGNO	257675	8.000
ChEMBL MOLREGNO	148842	7.658
ChEMBL MOLREGNO	149316	8.886
ChEMBL MOLREGNO	157480	6.260
ChEMBL MOLREGNO	173776	7.155
ChEMBL MOLREGNO	70198	8.222
ChEMBL MOLREGNO	70114	10.398
ChEMBL MOLREGNO	70167	10.000
ChEMBL MOLREGNO	10865	6.870
ChEMBL MOLREGNO	8469	8.699
ChEMBL MOLREGNO	8496	7.796

ChEMBL MOLREGNO	11033	5.714
ChEMBL MOLREGNO	121674	9.469
ChEMBL MOLREGNO	52311	8.469
ChEMBL MOLREGNO	117957	7.824
ChEMBL MOLREGNO	54789	8.959
ChEMBL MOLREGNO	109221	7.721
ChEMBL MOLREGNO	350189	9.301
ChEMBL MOLREGNO	60206	7.046
ChEMBL MOLREGNO	111779	8.745
ChEMBL MOLREGNO	286637	6.319
ChEMBL MOLREGNO	245258	7.854
ChEMBL MOLREGNO	271792	7.056
ChEMBL MOLREGNO	40675	8.456
ChEMBL MOLREGNO	174008	8.538
ChEMBL MOLREGNO	203062	6.000
ChEMBL MOLREGNO	204419	6.398
ChEMBL MOLREGNO	24923	6.328
ChEMBL MOLREGNO	161185	8.000
ChEMBL MOLREGNO	70108	9.222
ChEMBL MOLREGNO	128794	8.660
ChEMBL MOLREGNO	156808	7.658
ChEMBL MOLREGNO	161149	7.301
ChEMBL MOLREGNO	25746	8.721
ChEMBL MOLREGNO	1384814	6.854
ChEMBL	1799215	8.046

MOLREGNO		
ChEMBL MOLREGNO	157122	8.155
ChEMBL MOLREGNO	260825	8.721
ChEMBL MOLREGNO	260486	8.824
ChEMBL MOLREGNO	152809	8.770
ChEMBL MOLREGNO	142560	8.523
ChEMBL MOLREGNO	161546	9.301
ChEMBL MOLREGNO	173607	7.398
ChEMBL MOLREGNO	179669	7.745
ChEMBL MOLREGNO	191354	9.268
ChEMBL MOLREGNO	148077	8.076
Integrity Entry Number	226511	8.172
Integrity Entry Number	1007674	8.699
Integrity Entry Number	1007675	8.222
Integrity Entry Number	1007676	8.398
Integrity Entry Number	1007678	8.523
Integrity Entry Number	1007679	8.301
Integrity Entry Number	1007680	8.222
Integrity Entry Number	1023957	10.495
Integrity Entry Number	1023972	8.021
Integrity Entry Number	1023974	7.925
Integrity Entry Number	1023975	8.597
Integrity Entry Number	1023976	8.710
Integrity Entry Number	1023977	9.337
Integrity Entry Number	1023996	11.469

Integrity Entry Number	1023997	10.854
Integrity Entry Number	1023998	10.398
Integrity Entry Number	1023999	11.097
Integrity Entry Number	1032219	6.921
Integrity Entry Number	1032220	6.824
Integrity Entry Number	1032221	6.824
Integrity Entry Number	1043402	7.310
Integrity Entry Number	1044422	6.710
Integrity Entry Number	1044700	8.699
Integrity Entry Number	1044701	8.495
Integrity Entry Number	1044702	8.456
Integrity Entry Number	1046635	7.377
Integrity Entry Number	1046636	7.167
Integrity Entry Number	209883	8.824
Integrity Entry Number	229396	7.456
Integrity Entry Number	230530	8.319
Integrity Entry Number	231116	8.222
Integrity Entry Number	231117	8.658
Integrity Entry Number	234527	7.000
Integrity Entry Number	236951	7.167
Integrity Entry Number	240783	7.252
Integrity Entry Number	241797	9.097
Integrity Entry Number	242215	8.699
Integrity Entry Number	245124	6.854
Integrity Entry	252165	8.699

Number		
Integrity Entry Number	262028	8.420
Integrity Entry Number	264584	5.959
Integrity Entry Number	265970	6.415
Integrity Entry Number	276660	9.301
Integrity Entry Number	276662	9.301
Integrity Entry Number	276663	9.301
Integrity Entry Number	276667	9.301
Integrity Entry Number	281065	9.097
Integrity Entry Number	282216	4.793
Integrity Entry Number	287590	9.886
Integrity Entry Number	296747	5.620
Integrity Entry Number	296748	6.481
Integrity Entry Number	296750	6.699
Integrity Entry Number	297806	8.000
Integrity Entry Number	299234	8.222
Integrity Entry Number	299235	8.000
Integrity Entry Number	299236	8.155
Integrity Entry Number	303823	7.699
Integrity Entry Number	305851	10.155
Integrity Entry Number	305857	8.000
Integrity Entry Number	318792	4.745
Integrity Entry Number	323661	10.261
Integrity Entry Number	323662	9.613
Integrity Entry Number	323663	9.983

Integrity Entry Number	330601	9.824
Integrity Entry Number	330602	10.125
Integrity Entry Number	331271	10.699
Integrity Entry Number	331284	10.699
Integrity Entry Number	338320	8.244
Integrity Entry Number	338740	8.301
Integrity Entry Number	341370	6.770
Integrity Entry Number	344797	9.611
Integrity Entry Number	344798	10.699
Integrity Entry Number	345129	5.401
Integrity Entry Number	345130	5.419
Integrity Entry Number	350948	9.301
Integrity Entry Number	351761	8.398
Integrity Entry Number	351763	8.301
Integrity Entry Number	354474	10.301
Integrity Entry Number	403899	5.301
Integrity Entry Number	409169	8.347
Integrity Entry Number	409170	8.125
Integrity Entry Number	409171	7.886
Integrity Entry Number	409172	8.046
Integrity Entry Number	409173	8.155
Integrity Entry Number	416366	10.222
Integrity Entry Number	416372	9.215
Integrity Entry Number	416380	8.018
Integrity Entry	416398	7.123

Number		
Integrity Entry Number	416402	7.072
Integrity Entry Number	419773	9.357
Integrity Entry Number	438077	8.651
Integrity Entry Number	440581	4.979
Integrity Entry Number	464643	9.398
Integrity Entry Number	464644	8.886
Integrity Entry Number	464645	9.651
Integrity Entry Number	467029	8.602
Integrity Entry Number	474989	6.538
Integrity Entry Number	631335	8.602
Integrity Entry Number	631348	7.854
Integrity Entry Number	631349	8.585
Integrity Entry Number	631351	7.959
Integrity Entry Number	642667	5.585
Integrity Entry Number	642668	5.237
Integrity Entry Number	656941	11.314
Integrity Entry Number	656942	11.000
Integrity Entry Number	656943	10.602
Integrity Entry Number	656944	11.126
Integrity Entry Number	656945	10.824
Integrity Entry Number	656946	10.921
Integrity Entry Number	659666	7.194
Integrity Entry Number	659667	6.171
Integrity Entry Number	672762	5.991

Integrity Entry Number	672937	5.569
Integrity Entry Number	672942	5.569
Integrity Entry Number	685620	7.699
Integrity Entry Number	685625	7.523
Integrity Entry Number	692479	9.699
Integrity Entry Number	692485	9.155
Integrity Entry Number	692489	9.000
Integrity Entry Number	693351	8.921
Integrity Entry Number	693359	8.921
Integrity Entry Number	693364	8.538
Integrity Entry Number	716680	10.301
Integrity Entry Number	716682	9.155
Integrity Entry Number	716684	10.260
Integrity Entry Number	716686	9.658
Integrity Entry Number	716689	9.538
Integrity Entry Number	716692	10.301
Integrity Entry Number	737301	6.971
Integrity Entry Number	737303	6.804
Integrity Entry Number	737304	6.701
Integrity Entry Number	737305	6.700
Integrity Entry Number	737307	6.773
Integrity Entry Number	737308	6.755
Integrity Entry Number	738519	10.658
Integrity Entry Number	738522	10.553
Integrity Entry	751233	7.272

Number		
Integrity Entry Number	758997	6.071
Integrity Entry Number	758998	5.959
Integrity Entry Number	772662	11.000
Integrity Entry Number	772665	10.721
Integrity Entry Number	772670	10.699
Integrity Entry Number	772673	10.000
Integrity Entry Number	774715	7.222
Integrity Entry Number	793442	6.149
Integrity Entry Number	798323	10.301
Integrity Entry Number	798324	9.959
Integrity Entry Number	798325	10.000
Integrity Entry Number	798327	9.367
Integrity Entry Number	798329	9.921
Integrity Entry Number	798330	9.444
Integrity Entry Number	800937	7.125
Integrity Entry Number	802116	6.222
Integrity Entry Number	802120	6.301
Integrity Entry Number	802133	6.125
Integrity Entry Number	811456	6.017
Integrity Entry Number	812436	10.046
Integrity Entry Number	812439	9.708
Integrity Entry Number	812441	9.807
Integrity Entry Number	812442	9.745
Integrity Entry Number	812713	4.666

Integrity Entry Number	812714	4.632
Integrity Entry Number	812715	4.605
Integrity Entry Number	816436	5.939
Integrity Entry Number	819662	8.456
Integrity Entry Number	828357	8.585
Integrity Entry Number	831317	4.312
Integrity Entry Number	839758	4.254
Integrity Entry Number	848977	6.398
Integrity Entry Number	863857	6.174
Integrity Entry Number	864288	5.000
Integrity Entry Number	866259	6.155
Integrity Entry Number	866273	5.824
Integrity Entry Number	876412	9.319
Integrity Entry Number	887185	7.824
Integrity Entry Number	887186	7.137
Integrity Entry Number	887187	7.194
Integrity Entry Number	887188	7.137
Integrity Entry Number	895670	8.857
Integrity Entry Number	917744	8.652
Integrity Entry Number	917745	8.279
Integrity Entry Number	917746	7.955
Integrity Entry Number	917747	8.391
Integrity Entry Number	917748	8.311
Integrity Entry Number	917749	8.471
Integrity Entry	972338	9.000

Number		
Integrity Entry Number	972340	8.959
Integrity Entry Number	972343	8.699
Integrity Entry Number	973970	9.456
Integrity Entry Number	973971	9.114
Integrity Entry Number	973974	9.056
Integrity Entry Number	986836	8.208
Integrity Entry Number	986838	8.409
Integrity Entry Number	170711	8.886
Integrity Entry Number	199873	6.903
Integrity Entry Number	213633	9.523
Integrity Entry Number	702064	8.409
Integrity Entry Number	702057	8.102
Integrity Entry Number	702063	8.180
Integrity Entry Number	702052	8.745
Integrity Entry Number	242962	5.824
Integrity Entry Number	300629	7.745
Integrity Entry Number	1004081	8.747
Integrity Entry Number	205414	7.525
Integrity Entry Number	364763	8.398
Integrity Entry Number	257722	8.398
Integrity Entry Number	921892	11.678
Integrity Entry Number	921891	12.046
Integrity Entry Number	238078	8.796
Integrity Entry Number	205348	6.866

Integrity Entry Number	221229	7.125
Integrity Entry Number	207668	7.301
Integrity Entry Number	199877	8.046
Integrity Entry Number	194953	7.959
Integrity Entry Number	258682	7.569
Integrity Entry Number	253172	7.237
Integrity Entry Number	199903	7.446
Integrity Entry Number	249847	7.310
Integrity Entry Number	249846	7.403
Integrity Entry Number	324168	8.398
Integrity Entry Number	672067	5.201
Integrity Entry Number	685630	7.495
Integrity Entry Number	224673	8.130
Integrity Entry Number	236937	8.523
Integrity Entry Number	736655	7.780
Integrity Entry Number	736652	8.070
Integrity Entry Number	736657	5.857
Integrity Entry Number	736653	7.812
Integrity Entry Number	284435	8.194
Integrity Entry Number	199183	8.337
Integrity Entry Number	248908	5.762
Integrity Entry Number	350656	8.000
Integrity Entry Number	460927	9.745
Integrity Entry Number	188524	8.094
Integrity Entry	155135	9.299

Number		
Integrity Entry Number	173595	9.347
Integrity Entry Number	185531	10.000
Integrity Entry Number	181027	9.699
Integrity Entry Number	216346	9.921
Integrity Entry Number	285239	9.745
Integrity Entry Number	235900	8.310
Integrity Entry Number	214134	8.456
Integrity Entry Number	234416	9.000
Integrity Entry Number	246659	7.602
Integrity Entry Number	875440	4.208
Integrity Entry Number	875441	3.790
Integrity Entry Number	236900	5.770
Integrity Entry Number	288852	7.492
Integrity Entry Number	211732	6.581
Integrity Entry Number	753812	6.301
Integrity Entry Number	735017	7.108
Integrity Entry Number	753811	5.000
Integrity Entry Number	254805	8.444
Integrity Entry Number	254807	9.264
Integrity Entry Number	226499	8.398
Integrity Entry Number	207282	8.483
Integrity Entry Number	162085	5.678
Integrity Entry Number	667685	6.403
Integrity Entry Number	280792	8.854

Integrity Entry Number	240094	5.078
Integrity Entry Number	765534	8.237
Integrity Entry Number	765533	8.357
Integrity Entry Number	765532	8.456
Integrity Entry Number	765535	8.237
Integrity Entry Number	765530	8.796
Integrity Entry Number	765536	8.284
Integrity Entry Number	765539	8.367
Integrity Entry Number	765542	8.337
NIAID AIDSno	68	6.319
NIAID AIDSno	69	4.699
NIAID AIDSno	70	6.108
NIAID AIDSno	71	6.000
NIAID AIDSno	80	5.585
NIAID AIDSno	133	3.456
NIAID AIDSno	236	2.602
NIAID AIDSno	247	5.886
NIAID AIDSno	341	3.699
NIAID AIDSno	372	3.229
NIAID AIDSno	409	4.824
NIAID AIDSno	411	3.699
NIAID AIDSno	460	3.523
NIAID AIDSno	486	4.231
NIAID AIDSno	487	4.444
NIAID AIDSno	503	5.699
NIAID AIDSno	505	5.699
NIAID AIDSno	515	3.849
NIAID AIDSno	588	5.398
NIAID AIDSno	589	5.886
NIAID AIDSno	591	3.538
NIAID AIDSno	640	6.862
NIAID AIDSno	698	5.187
NIAID AIDSno	699	6.696
NIAID AIDSno	701	6.222
NIAID AIDSno	702	6.886
NIAID AIDSno	703	6.125
NIAID AIDSno	704	6.678
NIAID AIDSno	705	7.301

NIAID AIDSno	706	7.638
NIAID AIDSno	707	7.745
NIAID AIDSno	708	7.638
NIAID AIDSno	709	7.513
NIAID AIDSno	711	7.921
NIAID AIDSno	731	3.319
NIAID AIDSno	843	6.222
NIAID AIDSno	851	8.634
NIAID AIDSno	1300	4.000
NIAID AIDSno	1321	4.939
NIAID AIDSno	1353	6.377
NIAID AIDSno	1365	3.975
NIAID AIDSno	1373	4.921
NIAID AIDSno	1374	4.456
NIAID AIDSno	1375	3.959
NIAID AIDSno	1376	4.108
NIAID AIDSno	1377	4.745
NIAID AIDSno	1383	5.097
NIAID AIDSno	1387	4.000
NIAID AIDSno	1388	3.721
NIAID AIDSno	1389	4.456
NIAID AIDSno	1392	3.678
NIAID AIDSno	1398	3.903
NIAID AIDSno	1400	4.000
NIAID AIDSno	1401	4.222
NIAID AIDSno	1404	3.602
NIAID AIDSno	1407	4.620
NIAID AIDSno	1409	3.301
NIAID AIDSno	1410	3.000
NIAID AIDSno	1411	4.658
NIAID AIDSno	1412	4.340
NIAID AIDSno	1417	3.783
NIAID AIDSno	1438	8.784
NIAID AIDSno	1623	4.470
NIAID AIDSno	1659	7.282
NIAID AIDSno	1805	9.000
NIAID AIDSno	1827	7.921
NIAID AIDSno	1828	8.000
NIAID AIDSno	1830	8.310
NIAID AIDSno	1832	7.398
NIAID AIDSno	1835	9.210
NIAID AIDSno	2117	9.000
NIAID AIDSno	2239	3.097
NIAID AIDSno	2243	3.921
NIAID AIDSno	2303	6.620

NIAID AIDSno	2598	4.921
NIAID AIDSno	2599	4.046
NIAID AIDSno	2600	3.699
NIAID AIDSno	2604	3.420
NIAID AIDSno	2609	5.009
NIAID AIDSno	2683	4.699
NIAID AIDSno	2712	8.301
NIAID AIDSno	2713	8.469
NIAID AIDSno	2714	8.569
NIAID AIDSno	2715	7.678
NIAID AIDSno	2716	8.658
NIAID AIDSno	2717	8.538
NIAID AIDSno	2718	7.854
NIAID AIDSno	2719	9.000
NIAID AIDSno	2745	6.955
NIAID AIDSno	2746	5.553
NIAID AIDSno	2747	6.886
NIAID AIDSno	2748	7.700
NIAID AIDSno	2749	7.796
NIAID AIDSno	2750	7.658
NIAID AIDSno	2751	6.640
NIAID AIDSno	2753	8.745
NIAID AIDSno	2754	10.000
NIAID AIDSno	2755	8.721
NIAID AIDSno	2759	6.770
NIAID AIDSno	2791	9.523
NIAID AIDSno	2828	9.000
NIAID AIDSno	2829	7.699
NIAID AIDSno	2830	7.611
NIAID AIDSno	2831	8.222
NIAID AIDSno	2832	9.731
NIAID AIDSno	2833	7.872
NIAID AIDSno	2834	8.854
NIAID AIDSno	2835	6.735
NIAID AIDSno	2836	9.149
NIAID AIDSno	2837	9.824
NIAID AIDSno	2838	10.155
NIAID AIDSno	2839	10.523
NIAID AIDSno	3057	4.301
NIAID AIDSno	3058	3.284
NIAID AIDSno	3410	5.048
NIAID AIDSno	3433	7.651
NIAID AIDSno	3435	6.094
NIAID AIDSno	3438	4.728
NIAID AIDSno	3445	8.959

NIAID AIDSno	3446	6.260
NIAID AIDSno	3447	8.155
NIAID AIDSno	3449	8.569
NIAID AIDSno	3450	9.046
NIAID AIDSno	3451	8.284
NIAID AIDSno	3452	7.538
NIAID AIDSno	3453	7.194
NIAID AIDSno	3454	6.218
NIAID AIDSno	3455	7.222
NIAID AIDSno	3456	6.114
NIAID AIDSno	3458	9.602
NIAID AIDSno	3461	9.620
NIAID AIDSno	3462	8.237
NIAID AIDSno	3464	8.824
NIAID AIDSno	3465	8.678
NIAID AIDSno	3466	9.337
NIAID AIDSno	3467	9.357
NIAID AIDSno	3469	10.151
NIAID AIDSno	3470	7.638
NIAID AIDSno	3471	8.102
NIAID AIDSno	3472	9.234
NIAID AIDSno	3473	7.585
NIAID AIDSno	3474	8.149
NIAID AIDSno	3475	8.762
NIAID AIDSno	3476	9.337
NIAID AIDSno	3477	8.201
NIAID AIDSno	3478	9.056
NIAID AIDSno	3479	8.538
NIAID AIDSno	3480	8.886
NIAID AIDSno	3481	9.125
NIAID AIDSno	3482	9.161
NIAID AIDSno	3483	9.208
NIAID AIDSno	3484	9.056
NIAID AIDSno	3485	9.699
NIAID AIDSno	3486	10.155
NIAID AIDSno	3488	8.959
NIAID AIDSno	3499	8.319
NIAID AIDSno	3500	8.167
NIAID AIDSno	3501	7.585
NIAID AIDSno	3502	7.367
NIAID AIDSno	3503	9.000
NIAID AIDSno	3620	5.299
NIAID AIDSno	3621	5.509
NIAID AIDSno	3659	9.347
NIAID AIDSno	3660	10.398

NIAID AIDSno	3663	8.569
NIAID AIDSno	3777	5.155
NIAID AIDSno	3780	3.735
NIAID AIDSno	3781	3.987
NIAID AIDSno	3882	4.426
NIAID AIDSno	3957	8.222
NIAID AIDSno	3958	8.921
NIAID AIDSno	3959	9.444
NIAID AIDSno	3960	9.367
NIAID AIDSno	3961	9.638
NIAID AIDSno	3962	9.770
NIAID AIDSno	3963	9.538
NIAID AIDSno	3964	9.357
NIAID AIDSno	3965	9.143
NIAID AIDSno	3966	9.569
NIAID AIDSno	3967	9.509
NIAID AIDSno	3968	9.538
NIAID AIDSno	3969	9.784
NIAID AIDSno	3970	9.585
NIAID AIDSno	3971	9.222
NIAID AIDSno	3972	9.658
NIAID AIDSno	3973	9.292
NIAID AIDSno	3974	9.770
NIAID AIDSno	3975	9.678
NIAID AIDSno	3976	9.690
NIAID AIDSno	3977	9.770
NIAID AIDSno	3978	9.796
NIAID AIDSno	3979	10.000
NIAID AIDSno	3980	9.886
NIAID AIDSno	3981	10.155
NIAID AIDSno	3982	10.097
NIAID AIDSno	3983	9.367
NIAID AIDSno	3984	9.229
NIAID AIDSno	3985	9.000
NIAID AIDSno	3986	9.222
NIAID AIDSno	3987	8.854
NIAID AIDSno	3988	8.939
NIAID AIDSno	3991	7.460
NIAID AIDSno	3992	7.203
NIAID AIDSno	3993	9.770
NIAID AIDSno	4104	9.745
NIAID AIDSno	4105	9.728
NIAID AIDSno	4106	9.912
NIAID AIDSno	4107	9.260
NIAID AIDSno	4108	8.721

NIAID AIDSno	4109	9.699
NIAID AIDSno	4110	8.796
NIAID AIDSno	4111	8.796
NIAID AIDSno	4112	8.745
NIAID AIDSno	4113	9.699
NIAID AIDSno	4114	9.046
NIAID AIDSno	4115	9.328
NIAID AIDSno	4116	10.155
NIAID AIDSno	4117	10.097
NIAID AIDSno	4118	9.699
NIAID AIDSno	4119	10.046
NIAID AIDSno	4120	9.699
NIAID AIDSno	4146	3.523
NIAID AIDSno	4187	4.324
NIAID AIDSno	4498	10.523
NIAID AIDSno	4500	9.481
NIAID AIDSno	4501	10.523
NIAID AIDSno	4503	8.914
NIAID AIDSno	4505	6.477
NIAID AIDSno	4507	6.907
NIAID AIDSno	4509	6.459
NIAID AIDSno	4513	5.824
NIAID AIDSno	4514	6.027
NIAID AIDSno	4515	6.900
NIAID AIDSno	4516	6.286
NIAID AIDSno	4517	6.481
NIAID AIDSno	4518	6.585
NIAID AIDSno	4519	5.862
NIAID AIDSno	4520	6.655
NIAID AIDSno	4521	7.886
NIAID AIDSno	4522	8.523
NIAID AIDSno	4523	7.538
NIAID AIDSno	4524	8.301
NIAID AIDSno	4525	7.721
NIAID AIDSno	4526	8.523
NIAID AIDSno	4527	5.240
NIAID AIDSno	4529	6.979
NIAID AIDSno	4530	7.721
NIAID AIDSno	4614	9.268
NIAID AIDSno	4616	8.420
NIAID AIDSno	4617	8.509
NIAID AIDSno	4618	7.617
NIAID AIDSno	4848	4.032
NIAID AIDSno	4849	4.181
NIAID AIDSno	4870	4.824

NIAID AIDSno	4874	4.854
NIAID AIDSno	4909	5.398
NIAID AIDSno	4914	3.523
NIAID AIDSno	4916	6.569
NIAID AIDSno	4917	6.125
NIAID AIDSno	4963	5.886
NIAID AIDSno	4964	9.046
NIAID AIDSno	4965	6.268
NIAID AIDSno	4966	6.276
NIAID AIDSno	4967	8.301
NIAID AIDSno	4968	7.824
NIAID AIDSno	4971	7.959
NIAID AIDSno	4972	7.602
NIAID AIDSno	4984	8.155
NIAID AIDSno	4994	7.824
NIAID AIDSno	4995	8.699
NIAID AIDSno	5002	5.523
NIAID AIDSno	5003	4.658
NIAID AIDSno	5004	4.456
NIAID AIDSno	5005	4.420
NIAID AIDSno	5007	4.347
NIAID AIDSno	5079	4.824
NIAID AIDSno	5124	6.987
NIAID AIDSno	5125	5.987
NIAID AIDSno	5127	7.026
NIAID AIDSno	5129	7.652
NIAID AIDSno	5132	7.883
NIAID AIDSno	5133	8.193
NIAID AIDSno	5135	7.722
NIAID AIDSno	5145	8.000
NIAID AIDSno	5146	8.301
NIAID AIDSno	5147	8.301
NIAID AIDSno	5148	8.000
NIAID AIDSno	5149	8.000
NIAID AIDSno	5150	7.301
NIAID AIDSno	5152	9.651
NIAID AIDSno	5154	10.699
NIAID AIDSno	5155	6.523
NIAID AIDSno	5156	7.301
NIAID AIDSno	5157	7.000
NIAID AIDSno	5158	8.000
NIAID AIDSno	5159	9.301
NIAID AIDSno	5215	3.577
NIAID AIDSno	5340	7.636
NIAID AIDSno	5366	7.222

NIAID AIDSno	5367	8.523
NIAID AIDSno	5368	7.125
NIAID AIDSno	5369	8.284
NIAID AIDSno	5370	8.357
NIAID AIDSno	5371	8.161
NIAID AIDSno	5372	7.398
NIAID AIDSno	5373	6.086
NIAID AIDSno	5374	7.328
NIAID AIDSno	5375	7.678
NIAID AIDSno	5376	7.409
NIAID AIDSno	5377	7.959
NIAID AIDSno	5378	7.398
NIAID AIDSno	5379	6.076
NIAID AIDSno	5380	6.854
NIAID AIDSno	5381	7.155
NIAID AIDSno	5382	5.108
NIAID AIDSno	5383	3.921
NIAID AIDSno	5384	6.409
NIAID AIDSno	5385	8.620
NIAID AIDSno	5386	6.086
NIAID AIDSno	5387	7.721
NIAID AIDSno	5388	7.824
NIAID AIDSno	5389	7.237
NIAID AIDSno	5391	8.060
NIAID AIDSno	5392	8.268
NIAID AIDSno	5393	8.004
NIAID AIDSno	5394	10.143
NIAID AIDSno	5395	6.796
NIAID AIDSno	5396	5.319
NIAID AIDSno	5397	8.538
NIAID AIDSno	5398	9.280
NIAID AIDSno	5399	8.125
NIAID AIDSno	5400	7.854
NIAID AIDSno	5401	8.125
NIAID AIDSno	5402	9.638
NIAID AIDSno	5403	8.387
NIAID AIDSno	5404	7.409
NIAID AIDSno	5459	7.456
NIAID AIDSno	5460	5.056
NIAID AIDSno	5463	7.444
NIAID AIDSno	5468	3.745
NIAID AIDSno	5470	6.824
NIAID AIDSno	5471	4.594
NIAID AIDSno	5482	6.404
NIAID AIDSno	5484	6.357

NIAID AIDSno	5486	6.937
NIAID AIDSno	5489	8.420
NIAID AIDSno	5490	6.745
NIAID AIDSno	5492	6.714
NIAID AIDSno	5494	6.523
NIAID AIDSno	5495	5.959
NIAID AIDSno	5496	7.022
NIAID AIDSno	5497	7.268
NIAID AIDSno	5498	7.276
NIAID AIDSno	5499	6.042
NIAID AIDSno	5552	7.038
NIAID AIDSno	5658	4.471
NIAID AIDSno	5660	4.745
NIAID AIDSno	5661	3.796
NIAID AIDSno	5824	7.769
NIAID AIDSno	5825	7.553
NIAID AIDSno	5859	5.046
NIAID AIDSno	5867	5.222
NIAID AIDSno	5874	4.678
NIAID AIDSno	5875	3.294
NIAID AIDSno	5876	3.330
NIAID AIDSno	5877	3.719
NIAID AIDSno	5878	3.281
NIAID AIDSno	5879	3.656
NIAID AIDSno	5907	3.824
NIAID AIDSno	5908	3.545
NIAID AIDSno	5909	3.735
NIAID AIDSno	5910	3.975
NIAID AIDSno	5911	3.222
NIAID AIDSno	5912	3.538
NIAID AIDSno	5913	4.959
NIAID AIDSno	5914	4.921
NIAID AIDSno	5921	3.658
NIAID AIDSno	5962	7.398
NIAID AIDSno	5963	6.398
NIAID AIDSno	5964	6.222
NIAID AIDSno	5965	6.456
NIAID AIDSno	5966	5.523
NIAID AIDSno	5968	7.347
NIAID AIDSno	6007	6.097
NIAID AIDSno	6053	4.959
NIAID AIDSno	6080	7.000
NIAID AIDSno	6104	6.950
NIAID AIDSno	6113	7.215
NIAID AIDSno	6114	6.768

NIAID AIDSno	6116	7.947
NIAID AIDSno	6117	5.721
NIAID AIDSno	6119	6.983
NIAID AIDSno	6120	7.900
NIAID AIDSno	6121	6.910
NIAID AIDSno	6123	6.441
NIAID AIDSno	6125	7.952
NIAID AIDSno	6127	8.523
NIAID AIDSno	6128	7.927
NIAID AIDSno	6130	7.583
NIAID AIDSno	6150	5.721
NIAID AIDSno	6153	4.456
NIAID AIDSno	6154	4.602
NIAID AIDSno	6155	4.658
NIAID AIDSno	6156	3.796
NIAID AIDSno	6158	4.824
NIAID AIDSno	6159	4.602
NIAID AIDSno	6160	4.444
NIAID AIDSno	6161	5.041
NIAID AIDSno	6162	5.638
NIAID AIDSno	6163	4.076
NIAID AIDSno	6164	4.638
NIAID AIDSno	6165	3.921
NIAID AIDSno	6166	5.092
NIAID AIDSno	6167	5.523
NIAID AIDSno	6169	4.959
NIAID AIDSno	6170	4.108
NIAID AIDSno	6171	4.092
NIAID AIDSno	6172	8.186
NIAID AIDSno	6174	8.959
NIAID AIDSno	6175	7.801
NIAID AIDSno	6177	8.260
NIAID AIDSno	6178	6.022
NIAID AIDSno	6179	8.071
NIAID AIDSno	6180	7.489
NIAID AIDSno	6181	8.658
NIAID AIDSno	6182	8.469
NIAID AIDSno	6183	7.387
NIAID AIDSno	6184	6.827
NIAID AIDSno	6185	7.504
NIAID AIDSno	6245	3.553
NIAID AIDSno	6261	8.721
NIAID AIDSno	6265	5.398
NIAID AIDSno	6267	4.523
NIAID AIDSno	6268	4.745

NIAID AIDSno	6273	3.561
NIAID AIDSno	6274	5.155
NIAID AIDSno	6275	4.398
NIAID AIDSno	6278	4.347
NIAID AIDSno	6279	4.097
NIAID AIDSno	6281	5.398
NIAID AIDSno	6290	7.071
NIAID AIDSno	6292	7.292
NIAID AIDSno	6293	8.268
NIAID AIDSno	6295	8.097
NIAID AIDSno	6296	7.347
NIAID AIDSno	6298	9.538
NIAID AIDSno	6299	7.377
NIAID AIDSno	6300	9.509
NIAID AIDSno	6304	8.301
NIAID AIDSno	6311	7.008
NIAID AIDSno	6313	6.906
NIAID AIDSno	6335	8.469
NIAID AIDSno	6373	5.952
NIAID AIDSno	6374	6.097
NIAID AIDSno	6375	5.347
NIAID AIDSno	6376	6.301
NIAID AIDSno	6377	7.000
NIAID AIDSno	6378	5.469
NIAID AIDSno	6379	6.046
NIAID AIDSno	6380	5.111
NIAID AIDSno	6381	6.155
NIAID AIDSno	6382	6.398
NIAID AIDSno	6383	5.398
NIAID AIDSno	6384	5.035
NIAID AIDSno	6385	5.000
NIAID AIDSno	6386	5.000
NIAID AIDSno	6387	5.000
NIAID AIDSno	6388	5.000
NIAID AIDSno	6454	8.854
NIAID AIDSno	6472	5.284
NIAID AIDSno	6473	5.310
NIAID AIDSno	6474	5.538
NIAID AIDSno	6475	5.420
NIAID AIDSno	6476	4.678
NIAID AIDSno	6478	5.319
NIAID AIDSno	6479	4.432
NIAID AIDSno	6480	4.398
NIAID AIDSno	6481	3.796
NIAID AIDSno	6482	5.268

NIAID AIDSno	6483	4.921
NIAID AIDSno	6484	4.770
NIAID AIDSno	6485	4.770
NIAID AIDSno	6486	6.046
NIAID AIDSno	6548	5.000
NIAID AIDSno	6636	4.125
NIAID AIDSno	6637	3.770
NIAID AIDSno	6639	3.939
NIAID AIDSno	6642	4.022
NIAID AIDSno	6643	3.538
NIAID AIDSno	6644	3.450
NIAID AIDSno	6645	3.678
NIAID AIDSno	6646	3.276
NIAID AIDSno	6647	4.456
NIAID AIDSno	6648	3.921
NIAID AIDSno	6687	9.409
NIAID AIDSno	6688	9.523
NIAID AIDSno	6689	9.509
NIAID AIDSno	6690	10.222
NIAID AIDSno	6691	9.268
NIAID AIDSno	6742	5.000
NIAID AIDSno	6755	8.815
NIAID AIDSno	6756	7.420
NIAID AIDSno	6757	6.460
NIAID AIDSno	6758	7.097
NIAID AIDSno	6759	7.824
NIAID AIDSno	6760	9.489
NIAID AIDSno	6761	9.886
NIAID AIDSno	6762	10.886
NIAID AIDSno	6763	9.796
NIAID AIDSno	6764	8.215
NIAID AIDSno	6766	8.000
NIAID AIDSno	6767	8.886
NIAID AIDSno	6768	7.620
NIAID AIDSno	6769	7.721
NIAID AIDSno	6770	8.444
NIAID AIDSno	6771	7.943
NIAID AIDSno	6772	8.638
NIAID AIDSno	6773	8.337
NIAID AIDSno	6797	5.824
NIAID AIDSno	6798	5.187
NIAID AIDSno	6799	7.217
NIAID AIDSno	6801	6.347
NIAID AIDSno	6802	7.678
NIAID AIDSno	6803	6.876

NIAID AIDSno	6805	5.292
NIAID AIDSno	6806	5.796
NIAID AIDSno	6808	6.187
NIAID AIDSno	6809	7.161
NIAID AIDSno	6810	7.509
NIAID AIDSno	6811	6.000
NIAID AIDSno	6812	6.149
NIAID AIDSno	6813	5.194
NIAID AIDSno	6814	7.268
NIAID AIDSno	6815	7.638
NIAID AIDSno	6816	7.627
NIAID AIDSno	6817	6.523
NIAID AIDSno	6818	7.066
NIAID AIDSno	6819	6.796
NIAID AIDSno	6820	6.921
NIAID AIDSno	6870	4.678
NIAID AIDSno	6871	6.585
NIAID AIDSno	6872	5.450
NIAID AIDSno	6874	5.969
NIAID AIDSno	7892	8.796
NIAID AIDSno	8559	9.301
NIAID AIDSno	8728	9.301
NIAID AIDSno	10791	5.291
NIAID AIDSno	12208	6.125
NIAID AIDSno	12210	4.620
NIAID AIDSno	12889	3.000
NIAID AIDSno	13043	7.571
NIAID AIDSno	14011	7.886
NIAID AIDSno	14012	8.678
NIAID AIDSno	14093	8.432
NIAID AIDSno	14728	4.796
NIAID AIDSno	14809	5.097
NIAID AIDSno	14972	4.155
NIAID AIDSno	16075	6.495
NIAID AIDSno	16150	5.770
NIAID AIDSno	16180	4.215
NIAID AIDSno	16443	4.509
NIAID AIDSno	16484	5.699
NIAID AIDSno	17083	7.912
NIAID AIDSno	17669	3.456
NIAID AIDSno	20214	8.886
NIAID AIDSno	20675	8.046
NIAID AIDSno	20771	8.155
NIAID AIDSno	21040	7.638
NIAID AIDSno	22744	4.561

NIAID AIDSno	24923	4.959
NIAID AIDSno	25249	6.456
NIAID AIDSno	25250	5.824
NIAID AIDSno	25251	6.409
NIAID AIDSno	25252	6.432
NIAID AIDSno	25253	4.237
NIAID AIDSno	25374	5.469
NIAID AIDSno	25375	5.310
NIAID AIDSno	25376	4.947
NIAID AIDSno	25377	4.714
NIAID AIDSno	25378	4.893
NIAID AIDSno	25379	4.714
NIAID AIDSno	25380	4.983
NIAID AIDSno	25381	4.585
NIAID AIDSno	25450	7.456
NIAID AIDSno	25451	6.699
NIAID AIDSno	25452	5.959
NIAID AIDSno	25453	5.688
NIAID AIDSno	25454	5.824
NIAID AIDSno	25456	6.887
NIAID AIDSno	25458	7.000
NIAID AIDSno	25459	6.638
NIAID AIDSno	25460	6.699
NIAID AIDSno	25461	7.796
NIAID AIDSno	25842	4.684
NIAID AIDSno	26478	6.678
NIAID AIDSno	28008	8.699
NIAID AIDSno	28115	6.611
NIAID AIDSno	28117	7.036
NIAID AIDSno	28119	6.347
NIAID AIDSno	28120	6.528
NIAID AIDSno	28122	7.213
NIAID AIDSno	28124	6.495
NIAID AIDSno	28126	6.057
NIAID AIDSno	28128	6.903
NIAID AIDSno	28129	6.111
NIAID AIDSno	28131	5.886
NIAID AIDSno	28138	8.347
NIAID AIDSno	28139	6.839
NIAID AIDSno	28140	8.469
NIAID AIDSno	28141	8.481
NIAID AIDSno	28142	8.387
NIAID AIDSno	28143	8.481
NIAID AIDSno	28144	7.638
NIAID AIDSno	28145	7.959

NIAID AIDSno	28146	8.119
NIAID AIDSno	28147	8.018
NIAID AIDSno	28148	8.125
NIAID AIDSno	28149	8.796
NIAID AIDSno	28150	8.585
NIAID AIDSno	28151	8.276
NIAID AIDSno	28152	8.854
NIAID AIDSno	28153	8.469
NIAID AIDSno	28154	8.481
NIAID AIDSno	28155	7.921
NIAID AIDSno	28156	7.174
NIAID AIDSno	28157	7.509
NIAID AIDSno	28158	7.292
NIAID AIDSno	28159	6.602
NIAID AIDSno	28160	8.252
NIAID AIDSno	28161	7.509
NIAID AIDSno	28162	7.658
NIAID AIDSno	28163	7.979
NIAID AIDSno	28164	8.886
NIAID AIDSno	28165	8.215
NIAID AIDSno	28168	7.472
NIAID AIDSno	28170	6.699
NIAID AIDSno	28171	6.046
NIAID AIDSno	28172	6.908
NIAID AIDSno	28173	6.830
NIAID AIDSno	28174	6.648
NIAID AIDSno	28175	4.721
NIAID AIDSno	28176	5.456
NIAID AIDSno	28236	7.699
NIAID AIDSno	28238	4.125
NIAID AIDSno	28239	4.602
NIAID AIDSno	28241	3.921
NIAID AIDSno	28244	3.699
NIAID AIDSno	28249	4.155
NIAID AIDSno	28253	6.056
NIAID AIDSno	28254	5.991
NIAID AIDSno	28255	5.155
NIAID AIDSno	28256	3.959
NIAID AIDSno	28257	4.155
NIAID AIDSno	28258	4.347
NIAID AIDSno	28261	7.706
NIAID AIDSno	28262	7.623
NIAID AIDSno	28263	8.649
NIAID AIDSno	28265	6.721
NIAID AIDSno	28266	7.770

NIAID AIDSno	28267	6.387
NIAID AIDSno	28268	5.770
NIAID AIDSno	28270	7.742
NIAID AIDSno	28271	7.977
NIAID AIDSno	28273	10.000
NIAID AIDSno	28287	3.260
NIAID AIDSno	28288	4.347
NIAID AIDSno	28289	4.000
NIAID AIDSno	28291	5.188
NIAID AIDSno	28293	5.770
NIAID AIDSno	28294	6.796
NIAID AIDSno	28295	6.387
NIAID AIDSno	28296	7.237
NIAID AIDSno	28297	7.076
NIAID AIDSno	28302	4.745
NIAID AIDSno	28303	4.602
NIAID AIDSno	28304	4.444
NIAID AIDSno	28305	5.987
NIAID AIDSno	28306	6.562
NIAID AIDSno	28308	5.876
NIAID AIDSno	28309	7.086
NIAID AIDSno	28310	6.602
NIAID AIDSno	28313	6.745
NIAID AIDSno	28314	7.051
NIAID AIDSno	28315	7.636
NIAID AIDSno	28316	6.851
NIAID AIDSno	28317	7.229
NIAID AIDSno	28319	7.066
NIAID AIDSno	28320	6.876
NIAID AIDSno	28321	6.975
NIAID AIDSno	28323	7.092
NIAID AIDSno	28324	7.201
NIAID AIDSno	28325	6.987
NIAID AIDSno	28326	6.967
NIAID AIDSno	28337	6.985
NIAID AIDSno	28339	7.357
NIAID AIDSno	28340	6.284
NIAID AIDSno	28343	7.721
NIAID AIDSno	28344	7.456
NIAID AIDSno	28345	7.174
NIAID AIDSno	28346	6.444
NIAID AIDSno	28347	6.699
NIAID AIDSno	28348	5.959
NIAID AIDSno	28349	5.456
NIAID AIDSno	28350	7.081

NIAID AIDSno	28352	5.004
NIAID AIDSno	28353	6.854
NIAID AIDSno	28354	7.167
NIAID AIDSno	28355	7.174
NIAID AIDSno	28356	7.347
NIAID AIDSno	28357	6.921
NIAID AIDSno	28358	7.060
NIAID AIDSno	28359	6.262
NIAID AIDSno	28360	6.967
NIAID AIDSno	28361	5.481
NIAID AIDSno	28362	5.969
NIAID AIDSno	28363	6.635
NIAID AIDSno	28365	7.048
NIAID AIDSno	28366	7.137
NIAID AIDSno	28367	6.495
NIAID AIDSno	28368	7.620
NIAID AIDSno	28369	7.398
NIAID AIDSno	28370	7.444
NIAID AIDSno	28371	7.041
NIAID AIDSno	28382	7.125
NIAID AIDSno	28412	7.119
NIAID AIDSno	28465	8.000
NIAID AIDSno	28466	7.432
NIAID AIDSno	28467	7.319
NIAID AIDSno	28468	8.699
NIAID AIDSno	28469	8.481
NIAID AIDSno	28470	8.886
NIAID AIDSno	28471	8.576
NIAID AIDSno	28472	8.721
NIAID AIDSno	28473	8.398
NIAID AIDSno	28474	8.481
NIAID AIDSno	28475	8.444
NIAID AIDSno	28476	8.244
NIAID AIDSno	28477	8.102
NIAID AIDSno	28478	8.523
NIAID AIDSno	28479	9.301
NIAID AIDSno	28481	9.301
NIAID AIDSno	28482	7.620
NIAID AIDSno	28483	8.699
NIAID AIDSno	28484	9.222
NIAID AIDSno	28485	8.292
NIAID AIDSno	28486	9.398
NIAID AIDSno	28487	9.301
NIAID AIDSno	28488	8.796
NIAID AIDSno	28489	8.770

NIAID AIDSno	28490	8.222
NIAID AIDSno	28491	8.921
NIAID AIDSno	28492	8.745
NIAID AIDSno	28493	9.699
NIAID AIDSno	28494	8.000
NIAID AIDSno	28495	9.658
NIAID AIDSno	28496	9.301
NIAID AIDSno	28497	9.699
NIAID AIDSno	28498	8.745
NIAID AIDSno	28499	9.699
NIAID AIDSno	28500	9.377
NIAID AIDSno	28501	9.678
NIAID AIDSno	28502	9.149
NIAID AIDSno	28503	7.071
NIAID AIDSno	28504	8.000
NIAID AIDSno	28505	7.301
NIAID AIDSno	28506	8.678
NIAID AIDSno	28510	7.782
NIAID AIDSno	28511	6.896
NIAID AIDSno	28512	8.180
NIAID AIDSno	28513	8.481
NIAID AIDSno	28514	9.301
NIAID AIDSno	28524	3.260
NIAID AIDSno	28531	5.398
NIAID AIDSno	28590	7.159
NIAID AIDSno	28594	8.398
NIAID AIDSno	29590	7.301
NIAID AIDSno	29738	7.301
NIAID AIDSno	29804	7.301
NIAID AIDSno	29826	7.863
NIAID AIDSno	29827	8.076
NIAID AIDSno	29828	7.244
NIAID AIDSno	29829	7.006
NIAID AIDSno	29830	7.567
NIAID AIDSno	29831	7.319
NIAID AIDSno	29833	7.039
NIAID AIDSno	29834	8.319
NIAID AIDSno	29835	7.498
NIAID AIDSno	29836	7.516
NIAID AIDSno	29837	7.705
NIAID AIDSno	29838	7.461
NIAID AIDSno	29839	6.623
NIAID AIDSno	29840	8.081
NIAID AIDSno	29841	8.276
NIAID AIDSno	29842	8.420

NIAID AIDSno	29843	9.038
NIAID AIDSno	29845	8.734
NIAID AIDSno	29847	8.989
NIAID AIDSno	29851	9.085
NIAID AIDSno	29853	8.736
NIAID AIDSno	29855	9.200
NIAID AIDSno	29857	8.659
NIAID AIDSno	29858	8.071
NIAID AIDSno	29859	8.959
NIAID AIDSno	29860	7.959
NIAID AIDSno	29862	7.959
NIAID AIDSno	29864	8.721
NIAID AIDSno	29866	7.886
NIAID AIDSno	29867	7.854
NIAID AIDSno	29868	8.237
NIAID AIDSno	29869	8.347
NIAID AIDSno	29870	8.432
NIAID AIDSno	29871	8.056
NIAID AIDSno	29872	7.292
NIAID AIDSno	30179	5.222
NIAID AIDSno	30222	4.699
NIAID AIDSno	30797	7.634
NIAID AIDSno	30798	8.301
NIAID AIDSno	30799	8.699
NIAID AIDSno	30800	8.301
NIAID AIDSno	30801	7.562
NIAID AIDSno	30802	6.824
NIAID AIDSno	30803	7.824
NIAID AIDSno	30804	7.301
NIAID AIDSno	30805	9.000
NIAID AIDSno	30826	7.387
NIAID AIDSno	30827	7.252
NIAID AIDSno	30828	7.796
NIAID AIDSno	30829	7.509
NIAID AIDSno	30830	7.796
NIAID AIDSno	30831	7.174
NIAID AIDSno	30832	7.377
NIAID AIDSno	30833	7.208
NIAID AIDSno	30834	7.119
NIAID AIDSno	30835	7.745
NIAID AIDSno	30836	7.387
NIAID AIDSno	30837	7.721
NIAID AIDSno	30838	6.824
NIAID AIDSno	30839	7.481
NIAID AIDSno	30840	7.469

NIAID AIDSno	30841	7.208
NIAID AIDSno	30842	7.770
NIAID AIDSno	30843	7.432
NIAID AIDSno	30844	6.025
NIAID AIDSno	30845	6.873
NIAID AIDSno	30846	8.058
NIAID AIDSno	30847	6.752
NIAID AIDSno	30848	7.569
NIAID AIDSno	30849	7.292
NIAID AIDSno	30850	7.310
NIAID AIDSno	30851	6.785
NIAID AIDSno	30852	7.638
NIAID AIDSno	30853	7.174
NIAID AIDSno	30855	6.900
NIAID AIDSno	30868	7.770
NIAID AIDSno	30869	7.886
NIAID AIDSno	30870	7.432
NIAID AIDSno	30871	8.155
NIAID AIDSno	30872	6.375
NIAID AIDSno	30873	7.658
NIAID AIDSno	30874	6.772
NIAID AIDSno	30875	8.770
NIAID AIDSno	31064	8.699
NIAID AIDSno	31066	8.046
NIAID AIDSno	31097	10.000
NIAID AIDSno	31321	4.456
NIAID AIDSno	31422	4.569
NIAID AIDSno	31423	4.678
NIAID AIDSno	31424	4.638
NIAID AIDSno	31425	4.523
NIAID AIDSno	31426	4.495
NIAID AIDSno	31427	4.796
NIAID AIDSno	31428	4.770
NIAID AIDSno	31429	4.585
NIAID AIDSno	31506	7.824
NIAID AIDSno	31507	8.102
NIAID AIDSno	31539	4.432
NIAID AIDSno	31572	7.137
NIAID AIDSno	31573	6.491
NIAID AIDSno	31574	7.833
NIAID AIDSno	31576	9.602
NIAID AIDSno	31577	7.699
NIAID AIDSno	31578	7.833
NIAID AIDSno	31579	7.222
NIAID AIDSno	31580	8.745

NIAID AIDSno	31581	7.357
NIAID AIDSno	31582	8.921
NIAID AIDSno	31583	6.041
NIAID AIDSno	31584	6.770
NIAID AIDSno	31585	8.357
NIAID AIDSno	31586	7.757
NIAID AIDSno	31587	7.796
NIAID AIDSno	31588	8.456
NIAID AIDSno	31589	8.721
NIAID AIDSno	31590	9.347
NIAID AIDSno	31591	7.908
NIAID AIDSno	31592	9.420
NIAID AIDSno	31593	8.629
NIAID AIDSno	31594	9.073
NIAID AIDSno	31595	7.712
NIAID AIDSno	31596	8.553
NIAID AIDSno	31597	9.523
NIAID AIDSno	31598	8.244
NIAID AIDSno	31599	9.357
NIAID AIDSno	31600	7.658
NIAID AIDSno	31601	8.906
NIAID AIDSno	31605	6.658
NIAID AIDSno	31606	7.469
NIAID AIDSno	31614	5.398
NIAID AIDSno	31623	7.509
NIAID AIDSno	32502	6.383
NIAID AIDSno	32503	6.590
NIAID AIDSno	32504	6.431
NIAID AIDSno	32505	6.879
NIAID AIDSno	32506	6.991
NIAID AIDSno	32507	7.321
NIAID AIDSno	32508	6.757
NIAID AIDSno	32509	7.019
NIAID AIDSno	32510	7.170
NIAID AIDSno	32511	8.116
NIAID AIDSno	32512	6.991
NIAID AIDSno	32513	8.317
NIAID AIDSno	32514	6.815
NIAID AIDSno	32515	8.896
NIAID AIDSno	32516	9.108
NIAID AIDSno	32517	9.161
NIAID AIDSno	32518	9.268
NIAID AIDSno	32519	8.889
NIAID AIDSno	32520	8.824
NIAID AIDSno	32522	9.268

NIAID AIDSno	32523	6.322
NIAID AIDSno	32525	5.495
NIAID AIDSno	32526	6.678
NIAID AIDSno	32527	7.071
NIAID AIDSno	32529	5.000
NIAID AIDSno	32530	6.420
NIAID AIDSno	32531	7.036
NIAID AIDSno	32532	8.000
NIAID AIDSno	32533	8.222
NIAID AIDSno	32534	6.721
NIAID AIDSno	32535	7.854
NIAID AIDSno	32536	7.000
NIAID AIDSno	32537	7.569
NIAID AIDSno	32538	8.658
NIAID AIDSno	32539	8.912
NIAID AIDSno	32540	6.951
NIAID AIDSno	32541	7.208
NIAID AIDSno	32543	7.972
NIAID AIDSno	32545	8.854
NIAID AIDSno	32615	8.149
NIAID AIDSno	32619	6.161
NIAID AIDSno	32620	6.444
NIAID AIDSno	32621	5.788
NIAID AIDSno	32623	4.912
NIAID AIDSno	32624	5.923
NIAID AIDSno	32625	6.025
NIAID AIDSno	32626	6.066
NIAID AIDSno	32627	6.245
NIAID AIDSno	32628	5.690
NIAID AIDSno	32629	6.222
NIAID AIDSno	32630	5.670
NIAID AIDSno	32631	6.736
NIAID AIDSno	32632	6.201
NIAID AIDSno	32633	5.222
NIAID AIDSno	32634	4.849
NIAID AIDSno	32635	5.595
NIAID AIDSno	32636	6.143
NIAID AIDSno	32637	6.611
NIAID AIDSno	32638	6.211
NIAID AIDSno	32639	6.266
NIAID AIDSno	32640	5.339
NIAID AIDSno	32641	4.991
NIAID AIDSno	32643	5.069
NIAID AIDSno	32644	5.211
NIAID AIDSno	32645	4.714

NIAID AIDSno	32646	5.270
NIAID AIDSno	32647	4.606
NIAID AIDSno	32648	6.000
NIAID AIDSno	32649	7.469
NIAID AIDSno	32650	6.208
NIAID AIDSno	32651	7.301
NIAID AIDSno	32652	5.921
NIAID AIDSno	32653	6.721
NIAID AIDSno	32654	5.466
NIAID AIDSno	32655	6.170
NIAID AIDSno	32656	6.666
NIAID AIDSno	32657	6.205
NIAID AIDSno	32658	7.092
NIAID AIDSno	32659	6.836
NIAID AIDSno	32660	6.368
NIAID AIDSno	32661	7.585
NIAID AIDSno	32662	7.149
NIAID AIDSno	32663	7.041
NIAID AIDSno	32664	6.996
NIAID AIDSno	32665	7.036
NIAID AIDSno	32666	5.613
NIAID AIDSno	32667	5.957
NIAID AIDSno	32668	5.896
NIAID AIDSno	32669	5.956
NIAID AIDSno	32670	6.730
NIAID AIDSno	32671	6.545
NIAID AIDSno	32672	6.907
NIAID AIDSno	32673	6.249
NIAID AIDSno	32674	6.710
NIAID AIDSno	32675	6.234
NIAID AIDSno	32676	6.896
NIAID AIDSno	32677	6.631
NIAID AIDSno	32678	8.745
NIAID AIDSno	32679	7.056
NIAID AIDSno	32752	5.076
NIAID AIDSno	32937	7.713
NIAID AIDSno	32941	7.310
NIAID AIDSno	32946	8.886
NIAID AIDSno	32949	9.301
NIAID AIDSno	32950	8.387
NIAID AIDSno	32955	9.000
NIAID AIDSno	32959	8.886
NIAID AIDSno	33288	6.398
NIAID AIDSno	33289	6.886
NIAID AIDSno	33290	6.699

NIAID AIDSno	33291	6.523
NIAID AIDSno	33292	7.046
NIAID AIDSno	33293	7.301
NIAID AIDSno	33294	7.222
NIAID AIDSno	33295	6.959
NIAID AIDSno	33296	7.194
NIAID AIDSno	33297	7.143
NIAID AIDSno	33298	7.310
NIAID AIDSno	33299	6.921
NIAID AIDSno	33300	6.824
NIAID AIDSno	33301	7.398
NIAID AIDSno	33302	7.398
NIAID AIDSno	33303	7.222
NIAID AIDSno	33304	7.071
NIAID AIDSno	33305	7.000
NIAID AIDSno	33306	7.046
NIAID AIDSno	33307	7.523
NIAID AIDSno	33308	6.523
NIAID AIDSno	33309	6.854
NIAID AIDSno	33310	6.553
NIAID AIDSno	33311	6.495
NIAID AIDSno	33312	7.301
NIAID AIDSno	33313	7.066
NIAID AIDSno	33314	7.097
NIAID AIDSno	33404	6.301
NIAID AIDSno	33405	4.921
NIAID AIDSno	33406	5.167
NIAID AIDSno	33450	5.301
NIAID AIDSno	33468	5.886
NIAID AIDSno	33469	5.852
NIAID AIDSno	33470	6.284
NIAID AIDSno	33471	6.244
NIAID AIDSno	33560	9.523
NIAID AIDSno	33561	8.987
NIAID AIDSno	33562	9.092
NIAID AIDSno	33607	5.523
NIAID AIDSno	33608	6.108
NIAID AIDSno	33612	5.523
NIAID AIDSno	35037	9.108
NIAID AIDSno	35052	8.886
NIAID AIDSno	39057	5.097
NIAID AIDSno	42800	7.111
NIAID AIDSno	42801	9.000
NIAID AIDSno	42802	9.523
NIAID AIDSno	42819	8.000

NIAID AIDSno	42822	8.000
NIAID AIDSno	42823	8.000
NIAID AIDSno	42824	8.000
NIAID AIDSno	42825	6.500
NIAID AIDSno	42850	5.161
NIAID AIDSno	42851	5.092
NIAID AIDSno	42852	4.721
NIAID AIDSno	42853	4.538
NIAID AIDSno	42854	4.538
NIAID AIDSno	42855	4.585
NIAID AIDSno	42856	3.959
NIAID AIDSno	42857	5.770
NIAID AIDSno	42859	6.284
NIAID AIDSno	42860	6.108
NIAID AIDSno	42861	6.319
NIAID AIDSno	42862	6.585
NIAID AIDSno	42863	7.076
NIAID AIDSno	42864	6.824
NIAID AIDSno	42865	7.161
NIAID AIDSno	42866	6.643
NIAID AIDSno	42867	5.678
NIAID AIDSno	42868	5.292
NIAID AIDSno	42869	7.018
NIAID AIDSno	42870	8.301
NIAID AIDSno	42871	6.585
NIAID AIDSno	42872	7.222
NIAID AIDSno	42935	8.523
NIAID AIDSno	42936	7.854
NIAID AIDSno	42937	8.301
NIAID AIDSno	42938	8.569
NIAID AIDSno	42939	7.824
NIAID AIDSno	42940	8.000
NIAID AIDSno	42941	7.310
NIAID AIDSno	42942	7.432
NIAID AIDSno	42943	8.222
NIAID AIDSno	42944	6.721
NIAID AIDSno	42945	6.046
NIAID AIDSno	42946	8.523
NIAID AIDSno	42947	8.301
NIAID AIDSno	42948	7.398
NIAID AIDSno	42949	8.222
NIAID AIDSno	42950	8.046
NIAID AIDSno	42951	8.000
NIAID AIDSno	42993	8.046
NIAID AIDSno	42994	8.178

NIAID AIDSno	42995	9.151
NIAID AIDSno	43043	5.856
NIAID AIDSno	43045	4.745
NIAID AIDSno	43047	4.966
NIAID AIDSno	43049	5.620
NIAID AIDSno	43050	4.347
NIAID AIDSno	43051	5.102
NIAID AIDSno	43053	6.000
NIAID AIDSno	43054	5.111
NIAID AIDSno	43056	5.523
NIAID AIDSno	43057	6.071
NIAID AIDSno	43058	5.602
NIAID AIDSno	43059	5.301
NIAID AIDSno	43060	4.854
NIAID AIDSno	43061	6.120
NIAID AIDSno	43063	5.301
NIAID AIDSno	43064	6.678
NIAID AIDSno	43065	5.222
NIAID AIDSno	43066	5.523
NIAID AIDSno	43067	3.733
NIAID AIDSno	43068	6.301
NIAID AIDSno	43069	4.469
NIAID AIDSno	43070	5.267
NIAID AIDSno	43072	6.886
NIAID AIDSno	43073	7.137
NIAID AIDSno	43074	7.434
NIAID AIDSno	43075	8.137
NIAID AIDSno	43078	8.444
NIAID AIDSno	43079	7.456
NIAID AIDSno	43118	8.398
NIAID AIDSno	43130	5.155
NIAID AIDSno	43131	5.155
NIAID AIDSno	43133	4.000
NIAID AIDSno	43240	5.959
NIAID AIDSno	43332	5.699
NIAID AIDSno	43336	9.155
NIAID AIDSno	43341	9.301
NIAID AIDSno	43382	10.000
NIAID AIDSno	43383	10.699
NIAID AIDSno	43384	9.398
NIAID AIDSno	43385	9.481
NIAID AIDSno	43386	9.620
NIAID AIDSno	43387	10.301
NIAID AIDSno	43388	8.810
NIAID AIDSno	43412	8.292

NIAID AIDSno	43413	8.409
NIAID AIDSno	43414	8.357
NIAID AIDSno	43415	8.000
NIAID AIDSno	43416	7.921
NIAID AIDSno	43417	8.420
NIAID AIDSno	43418	7.886
NIAID AIDSno	43419	7.796
NIAID AIDSno	43420	8.125
NIAID AIDSno	43421	7.456
NIAID AIDSno	43422	7.796
NIAID AIDSno	43423	7.959
NIAID AIDSno	43424	6.983
NIAID AIDSno	43425	7.420
NIAID AIDSno	43426	7.444
NIAID AIDSno	43427	9.301
NIAID AIDSno	43428	9.301
NIAID AIDSno	43429	9.301
NIAID AIDSno	43430	9.301
NIAID AIDSno	43431	9.301
NIAID AIDSno	43432	9.301
NIAID AIDSno	43479	9.301
NIAID AIDSno	43480	8.495
NIAID AIDSno	43481	9.301
NIAID AIDSno	44541	5.824
NIAID AIDSno	44542	5.569
NIAID AIDSno	44543	7.849
NIAID AIDSno	44544	6.420
NIAID AIDSno	44546	7.854
NIAID AIDSno	44702	4.886
NIAID AIDSno	44733	9.886
NIAID AIDSno	44736	8.721
NIAID AIDSno	44838	8.602
NIAID AIDSno	45019	3.390
NIAID AIDSno	45020	3.283
NIAID AIDSno	45021	4.208
NIAID AIDSno	45022	3.842
NIAID AIDSno	45023	4.971
NIAID AIDSno	45024	3.635
NIAID AIDSno	45025	4.244
NIAID AIDSno	45026	3.415
NIAID AIDSno	45027	2.004
NIAID AIDSno	45029	8.328
NIAID AIDSno	45030	5.921
NIAID AIDSno	45031	5.796
NIAID AIDSno	45032	7.097

NIAID AIDSno	45033	6.979
NIAID AIDSno	45034	7.347
NIAID AIDSno	45035	7.013
NIAID AIDSno	45036	6.559
NIAID AIDSno	45037	7.022
NIAID AIDSno	45038	6.503
NIAID AIDSno	45039	6.538
NIAID AIDSno	45040	8.222
NIAID AIDSno	45049	8.770
NIAID AIDSno	45050	7.155
NIAID AIDSno	45051	8.886
NIAID AIDSno	45052	7.824
NIAID AIDSno	45053	9.301
NIAID AIDSno	45138	5.358
NIAID AIDSno	45140	4.276
NIAID AIDSno	45141	5.000
NIAID AIDSno	45142	4.097
NIAID AIDSno	45144	6.222
NIAID AIDSno	45145	7.222
NIAID AIDSno	45146	6.523
NIAID AIDSno	45147	6.000
NIAID AIDSno	45148	7.000
NIAID AIDSno	45149	7.398
NIAID AIDSno	45150	6.222
NIAID AIDSno	45151	6.187
NIAID AIDSno	45154	6.523
NIAID AIDSno	45155	7.000
NIAID AIDSno	45156	7.523
NIAID AIDSno	45157	7.000
NIAID AIDSno	45158	7.000
NIAID AIDSno	45159	7.793
NIAID AIDSno	45225	7.055
NIAID AIDSno	45227	7.954
NIAID AIDSno	45228	6.776
NIAID AIDSno	45229	7.262
NIAID AIDSno	45230	6.327
NIAID AIDSno	45232	6.707
NIAID AIDSno	45235	8.000
NIAID AIDSno	45236	8.398
NIAID AIDSno	45237	8.523
NIAID AIDSno	45238	8.046
NIAID AIDSno	45239	8.222
NIAID AIDSno	45240	8.000
NIAID AIDSno	45241	8.046
NIAID AIDSno	45242	8.046

NIAID AIDSno	45243	8.222
NIAID AIDSno	45249	8.097
NIAID AIDSno	45250	8.222
NIAID AIDSno	45251	8.398
NIAID AIDSno	45252	7.886
NIAID AIDSno	45253	8.398
NIAID AIDSno	45254	8.398
NIAID AIDSno	45361	5.301
NIAID AIDSno	45662	4.301
NIAID AIDSno	45708	8.412
NIAID AIDSno	45709	8.349
NIAID AIDSno	45710	8.228
NIAID AIDSno	45711	7.251
NIAID AIDSno	45712	8.046
NIAID AIDSno	45713	7.854
NIAID AIDSno	45714	7.553
NIAID AIDSno	45715	8.319
NIAID AIDSno	45719	8.167
NIAID AIDSno	45720	9.000
NIAID AIDSno	45723	7.585
NIAID AIDSno	45724	8.587
NIAID AIDSno	45726	6.745
NIAID AIDSno	45727	7.699
NIAID AIDSno	45728	7.638
NIAID AIDSno	45729	7.125
NIAID AIDSno	45730	7.367
NIAID AIDSno	45731	8.854
NIAID AIDSno	45732	8.077
NIAID AIDSno	45734	8.000
NIAID AIDSno	45735	7.921
NIAID AIDSno	45736	7.658
NIAID AIDSno	45737	8.824
NIAID AIDSno	45738	7.334
NIAID AIDSno	45741	9.000
NIAID AIDSno	45744	7.658
NIAID AIDSno	45747	5.886
NIAID AIDSno	45748	7.149
NIAID AIDSno	45751	9.000
NIAID AIDSno	45754	7.886
NIAID AIDSno	45757	8.658
NIAID AIDSno	45758	9.000
NIAID AIDSno	45759	8.276
NIAID AIDSno	45760	8.921
NIAID AIDSno	45761	9.000
NIAID AIDSno	45762	8.796

NIAID AIDSno	45763	8.149
NIAID AIDSno	45764	9.000
NIAID AIDSno	45767	9.000
NIAID AIDSno	45770	9.000
NIAID AIDSno	45773	9.000
NIAID AIDSno	45774	9.000
NIAID AIDSno	45775	9.000
NIAID AIDSno	45776	9.000
NIAID AIDSno	45777	9.000
NIAID AIDSno	45778	9.000
NIAID AIDSno	45779	9.000
NIAID AIDSno	45780	7.594
NIAID AIDSno	45998	8.959
NIAID AIDSno	46320	4.398
NIAID AIDSno	48394	4.377
NIAID AIDSno	50272	4.886
NIAID AIDSno	50273	4.436
NIAID AIDSno	50368	6.854
NIAID AIDSno	50389	7.208
NIAID AIDSno	50390	7.602
NIAID AIDSno	50391	6.109
NIAID AIDSno	50393	5.084
NIAID AIDSno	50394	7.721
NIAID AIDSno	50395	7.721
NIAID AIDSno	50396	7.824
NIAID AIDSno	50397	7.409
NIAID AIDSno	50398	6.870
NIAID AIDSno	50399	7.237
NIAID AIDSno	50400	7.854
NIAID AIDSno	50401	8.538
NIAID AIDSno	50402	8.420
NIAID AIDSno	50403	8.086
NIAID AIDSno	50404	8.602
NIAID AIDSno	50405	8.658
NIAID AIDSno	50406	8.658
NIAID AIDSno	50407	8.721
NIAID AIDSno	50408	8.444
NIAID AIDSno	50409	8.523
NIAID AIDSno	50490	7.745
NIAID AIDSno	50491	7.886
NIAID AIDSno	50492	7.959
NIAID AIDSno	50493	8.167
NIAID AIDSno	50494	8.824
NIAID AIDSno	50496	9.301
NIAID AIDSno	50498	9.301

NIAID AIDSno	50503	7.481
NIAID AIDSno	50504	8.409
NIAID AIDSno	50505	8.959
NIAID AIDSno	50506	7.886
NIAID AIDSno	50509	8.180
NIAID AIDSno	50511	9.301
NIAID AIDSno	50512	8.310
NIAID AIDSno	50513	9.000
NIAID AIDSno	50626	4.051
NIAID AIDSno	50628	4.276
NIAID AIDSno	50629	5.046
NIAID AIDSno	50783	6.605
NIAID AIDSno	50784	7.684
NIAID AIDSno	50785	5.296
NIAID AIDSno	50786	4.090
NIAID AIDSno	50787	6.511
NIAID AIDSno	50788	6.524
NIAID AIDSno	50791	4.155
NIAID AIDSno	50792	4.377
NIAID AIDSno	50793	5.284
NIAID AIDSno	50795	6.793
NIAID AIDSno	50798	4.233
NIAID AIDSno	50799	5.745
NIAID AIDSno	50802	8.824
NIAID AIDSno	50804	5.081
NIAID AIDSno	50834	3.756
NIAID AIDSno	51251	8.244
NIAID AIDSno	51252	8.155
NIAID AIDSno	51254	8.301
NIAID AIDSno	51255	6.766
NIAID AIDSno	51257	6.901
NIAID AIDSno	51259	7.101
NIAID AIDSno	51261	7.027
NIAID AIDSno	51263	7.343
NIAID AIDSno	51265	7.398
NIAID AIDSno	51266	4.905
NIAID AIDSno	51268	7.310
NIAID AIDSno	51269	7.398
NIAID AIDSno	51270	7.523
NIAID AIDSno	51271	7.071
NIAID AIDSno	51272	7.886
NIAID AIDSno	51273	8.699
NIAID AIDSno	51274	9.000
NIAID AIDSno	51275	8.699
NIAID AIDSno	51276	9.301

NIAID AIDSno	51282	7.602
NIAID AIDSno	51283	7.824
NIAID AIDSno	51284	8.301
NIAID AIDSno	51285	8.699
NIAID AIDSno	51286	6.602
NIAID AIDSno	51287	8.097
NIAID AIDSno	51333	5.284
NIAID AIDSno	51336	5.056
NIAID AIDSno	51338	6.342
NIAID AIDSno	51342	5.071
NIAID AIDSno	51344	5.000
NIAID AIDSno	51345	5.699
NIAID AIDSno	51346	5.000
NIAID AIDSno	51347	5.699
NIAID AIDSno	51348	5.699
NIAID AIDSno	51349	5.000
NIAID AIDSno	51350	5.699
NIAID AIDSno	51351	5.699
NIAID AIDSno	51354	5.699
NIAID AIDSno	51371	6.409
NIAID AIDSno	51374	5.699
NIAID AIDSno	51377	5.000
NIAID AIDSno	51378	5.000
NIAID AIDSno	51379	7.886
NIAID AIDSno	51380	8.398
NIAID AIDSno	51381	7.337
NIAID AIDSno	51382	6.854
NIAID AIDSno	51391	6.377
NIAID AIDSno	51392	5.602
NIAID AIDSno	51393	5.959
NIAID AIDSno	51394	6.577
NIAID AIDSno	51395	6.965
NIAID AIDSno	51396	7.257
NIAID AIDSno	51397	6.495
NIAID AIDSno	51398	7.056
NIAID AIDSno	51399	5.669
NIAID AIDSno	51400	6.145
NIAID AIDSno	51401	7.553
NIAID AIDSno	51402	6.854
NIAID AIDSno	51403	6.569
NIAID AIDSno	51404	5.963
NIAID AIDSno	51405	6.959
NIAID AIDSno	51406	7.102
NIAID AIDSno	51407	7.013
NIAID AIDSno	51408	6.000

NIAID AIDSno	51409	6.398
NIAID AIDSno	51410	5.770
NIAID AIDSno	51411	5.721
NIAID AIDSno	51412	6.481
NIAID AIDSno	51413	5.706
NIAID AIDSno	51415	6.125
NIAID AIDSno	51416	5.796
NIAID AIDSno	51417	6.347
NIAID AIDSno	51418	5.721
NIAID AIDSno	51419	5.824
NIAID AIDSno	51420	6.699
NIAID AIDSno	51421	6.444
NIAID AIDSno	51422	6.201
NIAID AIDSno	51423	7.824
NIAID AIDSno	51424	6.387
NIAID AIDSno	51425	7.585
NIAID AIDSno	51426	6.658
NIAID AIDSno	51427	6.658
NIAID AIDSno	51444	5.721
NIAID AIDSno	51445	5.870
NIAID AIDSno	51446	6.077
NIAID AIDSno	51447	5.228
NIAID AIDSno	51448	5.780
NIAID AIDSno	51449	5.553
NIAID AIDSno	51450	5.668
NIAID AIDSno	51451	5.301
NIAID AIDSno	51452	6.198
NIAID AIDSno	51453	6.398
NIAID AIDSno	51643	4.796
NIAID AIDSno	51644	5.849
NIAID AIDSno	51645	5.699
NIAID AIDSno	51646	4.523
NIAID AIDSno	51647	4.000
NIAID AIDSno	51648	4.347
NIAID AIDSno	51649	4.699
NIAID AIDSno	51652	4.398
NIAID AIDSno	51653	4.000
NIAID AIDSno	51657	4.952
NIAID AIDSno	51803	5.398
NIAID AIDSno	52067	8.071
NIAID AIDSno	56887	8.444
NIAID AIDSno	56937	6.538
NIAID AIDSno	56938	6.398
NIAID AIDSno	56939	6.907
NIAID AIDSno	56940	6.745

NIAID AIDSno	56941	6.951
NIAID AIDSno	56942	5.921
NIAID AIDSno	56943	6.738
NIAID AIDSno	56944	6.810
NIAID AIDSno	56945	6.292
NIAID AIDSno	56946	6.959
NIAID AIDSno	56947	6.818
NIAID AIDSno	56948	4.928
NIAID AIDSno	56950	5.886
NIAID AIDSno	56951	6.485
NIAID AIDSno	56952	5.921
NIAID AIDSno	56953	6.569
NIAID AIDSno	56954	5.886
NIAID AIDSno	56955	6.471
NIAID AIDSno	56956	5.642
NIAID AIDSno	56957	6.764
NIAID AIDSno	56958	5.900
NIAID AIDSno	56959	6.411
NIAID AIDSno	56960	7.638
NIAID AIDSno	56961	7.770
NIAID AIDSno	56962	8.000
NIAID AIDSno	56963	7.854
NIAID AIDSno	56964	6.896
NIAID AIDSno	56965	7.495
NIAID AIDSno	56966	7.921
NIAID AIDSno	56967	7.310
NIAID AIDSno	56968	7.420
NIAID AIDSno	56969	6.924
NIAID AIDSno	56970	7.854
NIAID AIDSno	56971	7.328
NIAID AIDSno	56972	7.432
NIAID AIDSno	56973	6.962
NIAID AIDSno	56974	7.770
NIAID AIDSno	56975	7.051
NIAID AIDSno	56976	5.854
NIAID AIDSno	56977	7.481
NIAID AIDSno	56978	8.167
NIAID AIDSno	56979	8.187
NIAID AIDSno	56980	7.824
NIAID AIDSno	56981	7.959
NIAID AIDSno	56982	7.620
NIAID AIDSno	56983	7.398
NIAID AIDSno	56984	7.495
NIAID AIDSno	56985	7.921
NIAID AIDSno	56986	8.589

NIAID AIDSno	56987	8.602
NIAID AIDSno	56988	8.509
NIAID AIDSno	56989	8.398
NIAID AIDSno	56990	8.854
NIAID AIDSno	56991	8.194
NIAID AIDSno	56992	8.432
NIAID AIDSno	56993	8.347
NIAID AIDSno	56994	6.921
NIAID AIDSno	56996	8.602
NIAID AIDSno	56997	9.222
NIAID AIDSno	56998	8.367
NIAID AIDSno	56999	8.495
NIAID AIDSno	57034	5.222
NIAID AIDSno	57248	3.975
NIAID AIDSno	57713	7.495
NIAID AIDSno	57714	7.886
NIAID AIDSno	57715	7.387
NIAID AIDSno	57716	7.420
NIAID AIDSno	57717	7.398
NIAID AIDSno	57718	7.658
NIAID AIDSno	57719	7.854
NIAID AIDSno	57720	8.000
NIAID AIDSno	57721	7.721
NIAID AIDSno	57722	7.699
NIAID AIDSno	57723	7.432
NIAID AIDSno	57724	7.699
NIAID AIDSno	57725	7.495
NIAID AIDSno	57726	7.509
NIAID AIDSno	57727	7.301
NIAID AIDSno	57728	7.481
NIAID AIDSno	57729	7.398
NIAID AIDSno	57730	7.602
NIAID AIDSno	57731	7.658
NIAID AIDSno	57732	7.824
NIAID AIDSno	57733	7.367
NIAID AIDSno	57734	7.398
NIAID AIDSno	57735	7.469
NIAID AIDSno	57736	7.301
NIAID AIDSno	57737	7.000
NIAID AIDSno	57738	7.678
NIAID AIDSno	57739	7.569
NIAID AIDSno	57740	7.081
NIAID AIDSno	57741	7.854
NIAID AIDSno	57742	7.268
NIAID AIDSno	57743	6.767

NIAID AIDSno	57744	7.143
NIAID AIDSno	57745	7.237
NIAID AIDSno	57746	7.538
NIAID AIDSno	57747	7.071
NIAID AIDSno	57748	7.921
NIAID AIDSno	57749	7.678
NIAID AIDSno	57750	7.495
NIAID AIDSno	57751	7.824
NIAID AIDSno	57752	7.432
NIAID AIDSno	57753	7.538
NIAID AIDSno	57754	7.921
NIAID AIDSno	57755	7.585
NIAID AIDSno	57756	7.398
NIAID AIDSno	57757	7.509
NIAID AIDSno	57758	7.699
NIAID AIDSno	57759	7.553
NIAID AIDSno	57760	7.469
NIAID AIDSno	57761	7.469
NIAID AIDSno	57762	7.292
NIAID AIDSno	57763	10.260
NIAID AIDSno	57764	7.082
NIAID AIDSno	57765	10.602
NIAID AIDSno	57771	3.613
NIAID AIDSno	57776	4.523
NIAID AIDSno	57778	4.357
NIAID AIDSno	57779	4.854
NIAID AIDSno	57784	5.796
NIAID AIDSno	57785	5.745
NIAID AIDSno	57786	4.983
NIAID AIDSno	57787	4.538
NIAID AIDSno	57801	7.634
NIAID AIDSno	57822	6.571
NIAID AIDSno	57825	5.222
NIAID AIDSno	57827	8.301
NIAID AIDSno	57992	6.699
NIAID AIDSno	58043	8.495
NIAID AIDSno	58044	8.658
NIAID AIDSno	58047	6.328
NIAID AIDSno	58149	5.523
NIAID AIDSno	58274	8.824
NIAID AIDSno	58277	6.469
NIAID AIDSno	58278	8.046
NIAID AIDSno	58279	7.000
NIAID AIDSno	58280	8.796
NIAID AIDSno	58281	9.000

NIAID AIDSno	58282	9.301
NIAID AIDSno	58302	5.232
NIAID AIDSno	58306	5.068
NIAID AIDSno	58307	4.420
NIAID AIDSno	58309	4.201
NIAID AIDSno	58310	6.530
NIAID AIDSno	58311	5.959
NIAID AIDSno	58312	8.268
NIAID AIDSno	58369	6.244
NIAID AIDSno	58370	6.796
NIAID AIDSno	58460	6.188
NIAID AIDSno	58462	5.722
NIAID AIDSno	58464	6.658
NIAID AIDSno	58465	4.432
NIAID AIDSno	58466	5.836
NIAID AIDSno	58467	4.527
NIAID AIDSno	58468	4.412
NIAID AIDSno	58470	4.573
NIAID AIDSno	58826	6.699
NIAID AIDSno	58827	8.000
NIAID AIDSno	58830	4.523
NIAID AIDSno	58831	4.523
NIAID AIDSno	58834	6.222
NIAID AIDSno	58835	6.699
NIAID AIDSno	58836	6.347
NIAID AIDSno	58837	6.398
NIAID AIDSno	58843	7.301
NIAID AIDSno	58845	8.000
NIAID AIDSno	58846	2.699
NIAID AIDSno	58848	2.699
NIAID AIDSno	58850	7.222
NIAID AIDSno	58851	7.699
NIAID AIDSno	59169	8.367
NIAID AIDSno	59170	6.870
NIAID AIDSno	59171	6.799
NIAID AIDSno	59172	8.721
NIAID AIDSno	59173	8.886
NIAID AIDSno	59174	7.569
NIAID AIDSno	59176	6.933
NIAID AIDSno	59177	7.886
NIAID AIDSno	59179	7.778
NIAID AIDSno	59183	6.347
NIAID AIDSno	59184	6.046
NIAID AIDSno	59185	7.886
NIAID AIDSno	59186	8.000

NIAID AIDSno	59187	7.056
NIAID AIDSno	59188	8.260
NIAID AIDSno	59189	8.097
NIAID AIDSno	59190	8.137
NIAID AIDSno	59191	8.046
NIAID AIDSno	59192	7.602
NIAID AIDSno	59193	8.824
NIAID AIDSno	59194	9.000
NIAID AIDSno	59195	8.469
NIAID AIDSno	59196	9.046
NIAID AIDSno	59197	8.523
NIAID AIDSno	59198	8.222
NIAID AIDSno	59229	6.319
NIAID AIDSno	59230	6.495
NIAID AIDSno	59231	6.267
NIAID AIDSno	59232	6.523
NIAID AIDSno	59233	6.072
NIAID AIDSno	59234	4.074
NIAID AIDSno	59235	5.107
NIAID AIDSno	59236	5.613
NIAID AIDSno	59237	6.409
NIAID AIDSno	59238	5.611
NIAID AIDSno	59354	8.770
NIAID AIDSno	59355	8.770
NIAID AIDSno	59356	10.523
NIAID AIDSno	59357	9.824
NIAID AIDSno	59358	9.854
NIAID AIDSno	59359	9.319
NIAID AIDSno	59360	10.523
NIAID AIDSno	59361	9.959
NIAID AIDSno	59362	10.046
NIAID AIDSno	59363	10.097
NIAID AIDSno	59364	9.921
NIAID AIDSno	59365	10.097
NIAID AIDSno	59472	8.553
NIAID AIDSno	59629	7.602
NIAID AIDSno	59630	6.793
NIAID AIDSno	59654	7.721
NIAID AIDSno	59655	8.301
NIAID AIDSno	59657	8.114
NIAID AIDSno	59658	7.959
NIAID AIDSno	59660	7.602
NIAID AIDSno	59661	7.497
NIAID AIDSno	59663	8.347
NIAID AIDSno	59664	7.854

NIAID AIDSno	59666	7.432
NIAID AIDSno	59667	9.071
NIAID AIDSno	59668	7.585
NIAID AIDSno	59669	8.292
NIAID AIDSno	59670	7.959
NIAID AIDSno	59671	7.921
NIAID AIDSno	59672	7.854
NIAID AIDSno	59673	7.538
NIAID AIDSno	59674	8.444
NIAID AIDSno	59677	7.409
NIAID AIDSno	59678	8.046
NIAID AIDSno	59679	7.481
NIAID AIDSno	59680	7.699
NIAID AIDSno	59681	8.252
NIAID AIDSno	59682	8.824
NIAID AIDSno	59683	9.046
NIAID AIDSno	59684	8.796
NIAID AIDSno	59685	8.602
NIAID AIDSno	59686	8.268
NIAID AIDSno	59695	6.372
NIAID AIDSno	59697	7.455
NIAID AIDSno	59699	6.599
NIAID AIDSno	59701	6.387
NIAID AIDSno	59704	7.192
NIAID AIDSno	59706	7.357
NIAID AIDSno	59707	7.208
NIAID AIDSno	59708	7.222
NIAID AIDSno	59709	6.409
NIAID AIDSno	59710	7.027
NIAID AIDSno	59722	5.798
NIAID AIDSno	59724	5.699
NIAID AIDSno	59726	5.585
NIAID AIDSno	59729	8.046
NIAID AIDSno	59730	7.301
NIAID AIDSno	60003	3.620
NIAID AIDSno	60271	7.456
NIAID AIDSno	60273	7.745
NIAID AIDSno	60274	7.292
NIAID AIDSno	60275	7.071
NIAID AIDSno	60276	7.237
NIAID AIDSno	60277	7.387
NIAID AIDSno	60282	6.863
NIAID AIDSno	60301	5.051
NIAID AIDSno	60314	10.000
NIAID AIDSno	60315	9.699

NIAID AIDSno	60316	10.222
NIAID AIDSno	60317	9.398
NIAID AIDSno	60318	10.398
NIAID AIDSno	60319	10.000
NIAID AIDSno	60445	3.793
NIAID AIDSno	60446	3.939
NIAID AIDSno	60447	4.409
NIAID AIDSno	60448	3.541
NIAID AIDSno	60449	3.426
NIAID AIDSno	60450	4.959
NIAID AIDSno	60451	3.939
NIAID AIDSno	60847	5.301
NIAID AIDSno	60856	7.824
NIAID AIDSno	60947	4.208
NIAID AIDSno	60949	4.000
NIAID AIDSno	60950	3.240
NIAID AIDSno	60991	7.339
NIAID AIDSno	60995	5.046
NIAID AIDSno	63280	8.244
NIAID AIDSno	70225	7.602
NIAID AIDSno	70226	8.108
NIAID AIDSno	70227	7.921
NIAID AIDSno	70228	7.916
NIAID AIDSno	70229	7.912
NIAID AIDSno	70230	7.387
NIAID AIDSno	70231	6.886
NIAID AIDSno	70264	6.389
NIAID AIDSno	70265	6.369
NIAID AIDSno	70267	6.928
NIAID AIDSno	70268	7.460
NIAID AIDSno	70269	7.509
NIAID AIDSno	70270	8.038
NIAID AIDSno	70271	7.347
NIAID AIDSno	70272	7.553
NIAID AIDSno	70273	7.849
NIAID AIDSno	70274	7.921
NIAID AIDSno	70275	8.143
NIAID AIDSno	70276	8.523
NIAID AIDSno	70277	8.638
NIAID AIDSno	70291	7.301
NIAID AIDSno	70292	6.174
NIAID AIDSno	70293	6.301
NIAID AIDSno	70294	8.215
NIAID AIDSno	70296	7.860
NIAID AIDSno	70297	8.066

NIAID AIDSno	70302	2.745
NIAID AIDSno	70303	4.000
NIAID AIDSno	70304	2.796
NIAID AIDSno	70305	4.222
NIAID AIDSno	70306	5.409
NIAID AIDSno	70313	4.097
NIAID AIDSno	70314	4.854
NIAID AIDSno	70315	4.886
NIAID AIDSno	70316	5.155
NIAID AIDSno	70317	5.222
NIAID AIDSno	70318	5.222
NIAID AIDSno	70319	5.398
NIAID AIDSno	70321	4.699
NIAID AIDSno	70322	5.046
NIAID AIDSno	70323	4.699
NIAID AIDSno	70324	5.097
NIAID AIDSno	70326	5.155
NIAID AIDSno	70327	5.222
NIAID AIDSno	70339	8.000
NIAID AIDSno	70340	5.836
NIAID AIDSno	70342	5.398
NIAID AIDSno	70343	5.953
NIAID AIDSno	70347	4.843
NIAID AIDSno	70476	4.398
NIAID AIDSno	70495	7.481
NIAID AIDSno	70496	8.071
NIAID AIDSno	70497	5.842
NIAID AIDSno	70498	6.462
NIAID AIDSno	70500	6.894
NIAID AIDSno	70501	5.524
NIAID AIDSno	70502	5.098
NIAID AIDSno	70504	7.420
NIAID AIDSno	70516	3.602
NIAID AIDSno	70517	3.301
NIAID AIDSno	70518	3.745
NIAID AIDSno	70519	3.301
NIAID AIDSno	70520	3.699
NIAID AIDSno	70521	5.372
NIAID AIDSno	70522	3.959
NIAID AIDSno	70524	6.097
NIAID AIDSno	70525	4.699
NIAID AIDSno	70547	8.959
NIAID AIDSno	70548	7.854
NIAID AIDSno	70549	7.721
NIAID AIDSno	70551	9.602

NIAID AIDSno	70552	8.824
NIAID AIDSno	70553	8.959
NIAID AIDSno	70555	8.854
NIAID AIDSno	70556	7.319
NIAID AIDSno	70557	7.620
NIAID AIDSno	70558	7.310
NIAID AIDSno	70559	7.796
NIAID AIDSno	70560	7.481
NIAID AIDSno	70561	8.569
NIAID AIDSno	70562	7.770
NIAID AIDSno	70563	8.328
NIAID AIDSno	70758	3.721
NIAID AIDSno	70759	3.367
NIAID AIDSno	70760	3.812
NIAID AIDSno	70761	3.682
NIAID AIDSno	70762	3.699
NIAID AIDSno	70763	3.638
NIAID AIDSno	70764	3.770
NIAID AIDSno	70765	3.495
NIAID AIDSno	70766	4.301
NIAID AIDSno	70769	3.745
NIAID AIDSno	70790	7.917
NIAID AIDSno	70939	7.301
NIAID AIDSno	71228	7.523
NIAID AIDSno	71230	7.023
NIAID AIDSno	71232	7.000
NIAID AIDSno	71233	6.000
NIAID AIDSno	71234	7.000
NIAID AIDSno	71235	7.000
NIAID AIDSno	71236	7.000
NIAID AIDSno	71237	6.523
NIAID AIDSno	71238	6.523
NIAID AIDSno	71239	6.523
NIAID AIDSno	71240	7.420
NIAID AIDSno	71241	8.108
NIAID AIDSno	71242	8.158
NIAID AIDSno	71243	8.387
NIAID AIDSno	71244	8.509
NIAID AIDSno	71245	8.056
NIAID AIDSno	71246	7.886
NIAID AIDSno	71247	8.523
NIAID AIDSno	71248	8.495
NIAID AIDSno	71249	8.495
NIAID AIDSno	71259	7.046
NIAID AIDSno	71260	7.097

NIAID AIDSno	71261	7.796
NIAID AIDSno	71262	7.367
NIAID AIDSno	71263	5.974
NIAID AIDSno	71264	8.222
NIAID AIDSno	71265	5.438
NIAID AIDSno	71266	5.000
NIAID AIDSno	71267	5.481
NIAID AIDSno	71268	5.337
NIAID AIDSno	71269	5.143
NIAID AIDSno	71272	6.715
NIAID AIDSno	71273	6.146
NIAID AIDSno	71274	6.946
NIAID AIDSno	71277	5.523
NIAID AIDSno	71278	5.000
NIAID AIDSno	71279	5.523
NIAID AIDSno	71280	7.523
NIAID AIDSno	71281	7.000
NIAID AIDSno	71282	6.523
NIAID AIDSno	71283	6.000
NIAID AIDSno	71284	5.523
NIAID AIDSno	71285	6.523
NIAID AIDSno	71304	8.921
NIAID AIDSno	71314	7.500
NIAID AIDSno	71316	8.000
NIAID AIDSno	71318	5.500
NIAID AIDSno	71320	5.761
NIAID AIDSno	71322	6.261
NIAID AIDSno	71325	4.523
NIAID AIDSno	71331	6.000
NIAID AIDSno	71335	5.154
NIAID AIDSno	71337	4.770
NIAID AIDSno	71346	8.721
NIAID AIDSno	71348	8.435
NIAID AIDSno	71350	8.157
NIAID AIDSno	71360	8.167
NIAID AIDSno	71361	8.638
NIAID AIDSno	71362	8.796
NIAID AIDSno	71363	8.553
NIAID AIDSno	71364	8.420
NIAID AIDSno	71365	8.387
NIAID AIDSno	71366	8.319
NIAID AIDSno	71367	8.538
NIAID AIDSno	71368	7.952
NIAID AIDSno	71369	8.678
NIAID AIDSno	71370	8.886

NIAID AIDSno	71371	8.678
NIAID AIDSno	71372	8.046
NIAID AIDSno	71373	8.469
NIAID AIDSno	71374	8.796
NIAID AIDSno	71375	8.292
NIAID AIDSno	71376	8.886
NIAID AIDSno	71377	8.745
NIAID AIDSno	71378	8.260
NIAID AIDSno	71379	8.149
NIAID AIDSno	71380	8.201
NIAID AIDSno	71381	8.745
NIAID AIDSno	71382	7.921
NIAID AIDSno	71383	7.620
NIAID AIDSno	71384	8.279
NIAID AIDSno	71385	8.509
NIAID AIDSno	71386	8.509
NIAID AIDSno	71387	8.509
NIAID AIDSno	71388	7.252
NIAID AIDSno	71389	8.432
NIAID AIDSno	71390	8.357
NIAID AIDSno	71391	8.060
NIAID AIDSno	71392	7.387
NIAID AIDSno	71393	8.886
NIAID AIDSno	71394	8.770
NIAID AIDSno	71395	7.921
NIAID AIDSno	71396	7.959
NIAID AIDSno	71398	8.658
NIAID AIDSno	71400	6.824
NIAID AIDSno	71401	7.456
NIAID AIDSno	71402	7.155
NIAID AIDSno	71403	8.538
NIAID AIDSno	71412	9.301
NIAID AIDSno	71417	9.301
NIAID AIDSno	71419	6.921
NIAID AIDSno	71420	9.301
NIAID AIDSno	71421	9.301
NIAID AIDSno	71422	9.301
NIAID AIDSno	71423	9.301
NIAID AIDSno	71424	9.301
NIAID AIDSno	71425	9.301
NIAID AIDSno	71426	9.301
NIAID AIDSno	71427	9.301
NIAID AIDSno	71428	9.301
NIAID AIDSno	71429	9.301
NIAID AIDSno	71430	9.301

NIAID AIDSno	71431	9.301
NIAID AIDSno	71432	9.301
NIAID AIDSno	71434	9.301
NIAID AIDSno	71436	9.301
NIAID AIDSno	71437	9.301
NIAID AIDSno	71487	6.523
NIAID AIDSno	71488	6.523
NIAID AIDSno	71489	6.523
NIAID AIDSno	71490	6.000
NIAID AIDSno	71491	6.523
NIAID AIDSno	71492	6.523
NIAID AIDSno	71493	6.523
NIAID AIDSno	71494	6.523
NIAID AIDSno	71495	6.523
NIAID AIDSno	71496	7.523
NIAID AIDSno	71497	6.523
NIAID AIDSno	71498	7.523
NIAID AIDSno	71499	8.000
NIAID AIDSno	71500	6.523
NIAID AIDSno	71501	8.000
NIAID AIDSno	71502	7.000
NIAID AIDSno	71503	8.000
NIAID AIDSno	71504	8.000
NIAID AIDSno	71505	10.125
NIAID AIDSno	71509	5.000
NIAID AIDSno	71542	9.301
NIAID AIDSno	71546	9.301
NIAID AIDSno	71547	9.301
NIAID AIDSno	71548	9.301
NIAID AIDSno	71550	9.301
NIAID AIDSno	71551	9.301
NIAID AIDSno	71552	9.301
NIAID AIDSno	71553	9.301
NIAID AIDSno	71554	9.301
NIAID AIDSno	71555	9.301
NIAID AIDSno	71556	9.301
NIAID AIDSno	71558	9.301
NIAID AIDSno	71559	9.301
NIAID AIDSno	71565	9.301
NIAID AIDSno	71566	9.301
NIAID AIDSno	71704	5.398
NIAID AIDSno	71786	5.699
NIAID AIDSno	71822	8.398
NIAID AIDSno	71973	5.000
NIAID AIDSno	71978	8.276

NIAID AIDSno	71979	7.876
NIAID AIDSno	71982	7.987
NIAID AIDSno	71983	8.108
NIAID AIDSno	71984	8.301
NIAID AIDSno	71985	8.398
NIAID AIDSno	71995	6.415
NIAID AIDSno	71996	5.387
NIAID AIDSno	71997	5.310
NIAID AIDSno	71998	6.071
NIAID AIDSno	71999	6.229
NIAID AIDSno	72000	5.337
NIAID AIDSno	72001	6.174
NIAID AIDSno	72002	6.260
NIAID AIDSno	72020	7.000
NIAID AIDSno	72021	8.658
NIAID AIDSno	72022	7.222
NIAID AIDSno	72023	8.678
NIAID AIDSno	72024	9.523
NIAID AIDSno	72025	6.444
NIAID AIDSno	72026	8.538
NIAID AIDSno	72027	9.398
NIAID AIDSno	72029	6.854
NIAID AIDSno	72030	9.000
NIAID AIDSno	72031	9.523
NIAID AIDSno	72032	9.301
NIAID AIDSno	72033	8.770
NIAID AIDSno	72034	9.523
NIAID AIDSno	72035	9.398
NIAID AIDSno	72036	7.301
NIAID AIDSno	72037	7.000
NIAID AIDSno	72038	9.523
NIAID AIDSno	72039	9.125
NIAID AIDSno	72040	6.469
NIAID AIDSno	72041	4.699
NIAID AIDSno	72042	9.071
NIAID AIDSno	72043	9.602
NIAID AIDSno	72044	8.854
NIAID AIDSno	72045	7.658
NIAID AIDSno	72047	7.420
NIAID AIDSno	72048	9.046
NIAID AIDSno	72049	9.009
NIAID AIDSno	72050	9.469
NIAID AIDSno	72051	9.097
NIAID AIDSno	72052	8.155
NIAID AIDSno	72053	8.398

NIAID AIDSno	72054	8.319
NIAID AIDSno	72056	8.398
NIAID AIDSno	72057	9.097
NIAID AIDSno	72177	5.509
NIAID AIDSno	72184	5.000
NIAID AIDSno	72185	6.699
NIAID AIDSno	72186	5.097
NIAID AIDSno	72206	9.523
NIAID AIDSno	72209	7.097
NIAID AIDSno	72448	3.903
NIAID AIDSno	72451	2.699
NIAID AIDSno	72510	5.301
NIAID AIDSno	72513	8.187
NIAID AIDSno	72514	8.420
NIAID AIDSno	72515	8.357
NIAID AIDSno	72516	8.114
NIAID AIDSno	72517	7.870
NIAID AIDSno	72518	8.387
NIAID AIDSno	72519	8.222
NIAID AIDSno	72520	7.699
NIAID AIDSno	72521	8.125
NIAID AIDSno	72522	8.463
NIAID AIDSno	72523	7.959
NIAID AIDSno	72524	8.469
NIAID AIDSno	72525	8.301
NIAID AIDSno	72526	7.824
NIAID AIDSno	72527	8.215
NIAID AIDSno	72528	8.292
NIAID AIDSno	72529	8.294
NIAID AIDSno	72530	8.456
NIAID AIDSno	72531	7.854
NIAID AIDSno	72532	7.222
NIAID AIDSno	72533	7.041
NIAID AIDSno	72549	4.398
NIAID AIDSno	72550	5.921
NIAID AIDSno	72554	5.314
NIAID AIDSno	72556	5.509
NIAID AIDSno	72557	5.638
NIAID AIDSno	72559	5.879
NIAID AIDSno	72560	5.060
NIAID AIDSno	72565	8.569
NIAID AIDSno	72770	8.018
NIAID AIDSno	72856	8.357
NIAID AIDSno	72858	7.824
NIAID AIDSno	72886	8.222

NIAID AIDSno	73030	8.699
NIAID AIDSno	73031	8.523
NIAID AIDSno	73034	8.387
NIAID AIDSno	73035	8.102
NIAID AIDSno	73039	8.699
NIAID AIDSno	73040	8.523
NIAID AIDSno	73041	8.699
NIAID AIDSno	73042	9.000
NIAID AIDSno	73043	8.699
NIAID AIDSno	73044	8.301
NIAID AIDSno	73045	8.097
NIAID AIDSno	73046	9.000
NIAID AIDSno	73047	7.959
NIAID AIDSno	73048	7.824
NIAID AIDSno	73049	8.000
NIAID AIDSno	73050	8.155
NIAID AIDSno	73051	9.301
NIAID AIDSno	73052	8.097
NIAID AIDSno	73053	7.745
NIAID AIDSno	73054	7.886
NIAID AIDSno	73055	8.301
NIAID AIDSno	73056	9.301
NIAID AIDSno	73057	8.125
NIAID AIDSno	73058	7.658
NIAID AIDSno	73059	7.959
NIAID AIDSno	73060	7.796
NIAID AIDSno	73061	8.699
NIAID AIDSno	73062	8.097
NIAID AIDSno	73063	8.097
NIAID AIDSno	73064	8.523
NIAID AIDSno	79982	4.114
NIAID AIDSno	79985	5.301
NIAID AIDSno	79990	7.301
NIAID AIDSno	79992	7.301
NIAID AIDSno	79993	7.301
NIAID AIDSno	80351	7.854
NIAID AIDSno	80353	8.638
NIAID AIDSno	80551	10.000
NIAID AIDSno	80599	6.925
NIAID AIDSno	80600	7.256
NIAID AIDSno	80601	7.103
NIAID AIDSno	80613	6.271
NIAID AIDSno	80716	6.000
NIAID AIDSno	81394	7.125
NIAID AIDSno	81741	7.301

NIAID AIDSno	81743	7.301
NIAID AIDSno	81744	7.301
NIAID AIDSno	81746	7.301
NIAID AIDSno	81748	7.301
NIAID AIDSno	81749	7.301
NIAID AIDSno	82016	6.745
NIAID AIDSno	82017	6.432
NIAID AIDSno	82184	7.301
NIAID AIDSno	82207	7.287
NIAID AIDSno	82209	8.319
NIAID AIDSno	82210	7.075
NIAID AIDSno	82212	6.685
NIAID AIDSno	82243	7.022
NIAID AIDSno	82256	7.432
NIAID AIDSno	82257	7.071
NIAID AIDSno	82393	8.000
NIAID AIDSno	82394	8.000
NIAID AIDSno	82395	8.000
NIAID AIDSno	82551	6.301
NIAID AIDSno	82552	6.824
NIAID AIDSno	85039	5.849
NIAID AIDSno	85040	6.523
NIAID AIDSno	85041	8.222
NIAID AIDSno	85042	8.097
NIAID AIDSno	85043	6.427
NIAID AIDSno	85044	4.921
NIAID AIDSno	85172	3.796
NIAID AIDSno	85173	4.292
NIAID AIDSno	85174	4.745
NIAID AIDSno	85176	5.347
NIAID AIDSno	85249	4.491
NIAID AIDSno	85276	5.244
NIAID AIDSno	85277	5.623
NIAID AIDSno	85278	6.143
NIAID AIDSno	85279	4.523
NIAID AIDSno	85280	4.699
NIAID AIDSno	85282	4.824
NIAID AIDSno	85283	4.745
NIAID AIDSno	85284	6.086
NIAID AIDSno	85286	5.252
NIAID AIDSno	85287	5.848
NIAID AIDSno	85290	4.222
NIAID AIDSno	85293	4.340
NIAID AIDSno	85295	5.155
NIAID AIDSno	85296	5.222

NIAID AIDSno	85298	5.924
NIAID AIDSno	85305	3.824
NIAID AIDSno	85308	4.222
NIAID AIDSno	85309	3.699
NIAID AIDSno	85310	4.301
NIAID AIDSno	85311	4.155
NIAID AIDSno	85312	3.921
NIAID AIDSno	85313	4.377
NIAID AIDSno	85314	5.046
NIAID AIDSno	85315	5.201
NIAID AIDSno	85316	4.194
NIAID AIDSno	85318	5.018
NIAID AIDSno	85320	5.387
NIAID AIDSno	85321	6.772
NIAID AIDSno	85323	5.081
NIAID AIDSno	85325	4.924
NIAID AIDSno	85327	5.046
NIAID AIDSno	85328	5.886
NIAID AIDSno	85329	6.487
NIAID AIDSno	85330	6.167
NIAID AIDSno	85331	5.097
NIAID AIDSno	85332	5.759
NIAID AIDSno	85333	5.910
NIAID AIDSno	85334	6.377
NIAID AIDSno	85335	5.456
NIAID AIDSno	85336	6.638
NIAID AIDSno	85337	6.983
NIAID AIDSno	85338	5.602
NIAID AIDSno	85339	6.455
NIAID AIDSno	85340	6.349
NIAID AIDSno	85341	8.119
NIAID AIDSno	85342	6.638
NIAID AIDSno	85343	6.842
NIAID AIDSno	85344	6.699
NIAID AIDSno	85345	6.738
NIAID AIDSno	85346	6.983
NIAID AIDSno	85347	7.046
NIAID AIDSno	85348	6.648
NIAID AIDSno	85349	6.708
NIAID AIDSno	85350	6.959
NIAID AIDSno	85351	6.991
NIAID AIDSno	85352	7.174
NIAID AIDSno	85353	7.036
NIAID AIDSno	85354	7.167
NIAID AIDSno	85355	7.284

NIAID AIDSno	85356	6.987
NIAID AIDSno	85358	6.538
NIAID AIDSno	85359	3.646
NIAID AIDSno	85361	4.257
NIAID AIDSno	85451	8.921
NIAID AIDSno	85452	8.310
NIAID AIDSno	85453	8.523
NIAID AIDSno	85454	8.469
NIAID AIDSno	85455	8.585
NIAID AIDSno	85456	7.699
NIAID AIDSno	85457	8.013
NIAID AIDSno	85458	7.585
NIAID AIDSno	85459	8.367
NIAID AIDSno	85460	8.409
NIAID AIDSno	85461	9.000
NIAID AIDSno	85462	8.051
NIAID AIDSno	85463	8.409
NIAID AIDSno	85467	6.561
NIAID AIDSno	85470	4.854
NIAID AIDSno	85471	4.854
NIAID AIDSno	85472	5.155
NIAID AIDSno	85502	8.854
NIAID AIDSno	85503	9.046
NIAID AIDSno	85504	9.222
NIAID AIDSno	85505	8.347
NIAID AIDSno	85506	9.046
NIAID AIDSno	85507	9.222
NIAID AIDSno	85508	9.155
NIAID AIDSno	85509	9.097
NIAID AIDSno	85510	9.097
NIAID AIDSno	85511	8.796
NIAID AIDSno	85512	9.222
NIAID AIDSno	85513	9.046
NIAID AIDSno	85514	9.301
NIAID AIDSno	85515	8.886
NIAID AIDSno	85516	9.046
NIAID AIDSno	85517	8.921
NIAID AIDSno	85518	8.854
NIAID AIDSno	85519	8.824
NIAID AIDSno	85520	10.000
NIAID AIDSno	85521	8.086
NIAID AIDSno	85522	8.886
NIAID AIDSno	85523	8.770
NIAID AIDSno	85524	8.456
NIAID AIDSno	85525	8.721

NIAID AIDSno	85526	8.678
NIAID AIDSno	85527	8.337
NIAID AIDSno	85528	8.201
NIAID AIDSno	85529	8.409
NIAID AIDSno	85530	8.602
NIAID AIDSno	85531	8.886
NIAID AIDSno	85532	8.469
NIAID AIDSno	85533	8.066
NIAID AIDSno	85534	8.444
NIAID AIDSno	85535	7.302
NIAID AIDSno	85536	7.465
NIAID AIDSno	85537	8.886
NIAID AIDSno	85538	8.921
NIAID AIDSno	85539	8.495
NIAID AIDSno	85540	9.097
NIAID AIDSno	85541	8.553
NIAID AIDSno	85542	8.921
NIAID AIDSno	85543	8.824
NIAID AIDSno	85544	8.721
NIAID AIDSno	85545	5.886
NIAID AIDSno	85546	5.796
NIAID AIDSno	85547	5.824
NIAID AIDSno	85548	5.602
NIAID AIDSno	85549	6.046
NIAID AIDSno	85550	5.187
NIAID AIDSno	85551	6.097
NIAID AIDSno	85552	6.301
NIAID AIDSno	85553	7.538
NIAID AIDSno	85554	7.387
NIAID AIDSno	85555	6.245
NIAID AIDSno	85556	4.772
NIAID AIDSno	85557	4.582
NIAID AIDSno	85558	5.367
NIAID AIDSno	85559	5.745
NIAID AIDSno	85560	6.097
NIAID AIDSno	85561	5.222
NIAID AIDSno	85563	6.420
NIAID AIDSno	85564	5.602
NIAID AIDSno	85566	5.420
NIAID AIDSno	85567	5.824
NIAID AIDSno	85568	5.658
NIAID AIDSno	85569	5.824
NIAID AIDSno	85570	5.745
NIAID AIDSno	85571	5.959
NIAID AIDSno	85572	5.796

NIAID AIDSno	85573	4.000
NIAID AIDSno	85574	5.301
NIAID AIDSno	85703	5.318
NIAID AIDSno	85899	9.301
NIAID AIDSno	85909	9.796
NIAID AIDSno	85919	4.301
NIAID AIDSno	85920	4.046
NIAID AIDSno	85921	4.155
NIAID AIDSno	85926	8.000
NIAID AIDSno	85927	8.000
NIAID AIDSno	85928	6.000
NIAID AIDSno	85929	7.000
NIAID AIDSno	85930	8.000
NIAID AIDSno	85931	6.000
NIAID AIDSno	85934	5.000
NIAID AIDSno	85935	6.523
NIAID AIDSno	85936	8.000
NIAID AIDSno	85937	7.000
NIAID AIDSno	85938	3.523
NIAID AIDSno	85939	7.523
NIAID AIDSno	85941	5.000
NIAID AIDSno	85942	7.000
NIAID AIDSno	85943	5.000
NIAID AIDSno	85944	6.000
NIAID AIDSno	85945	8.000
NIAID AIDSno	85946	7.000
NIAID AIDSno	85947	6.000
NIAID AIDSno	85948	8.000
NIAID AIDSno	85949	8.000
NIAID AIDSno	85950	5.000
NIAID AIDSno	85951	6.523
NIAID AIDSno	85952	6.000
NIAID AIDSno	86118	9.301
NIAID AIDSno	86119	9.301
NIAID AIDSno	86120	9.301
NIAID AIDSno	86122	9.301
NIAID AIDSno	86123	9.301
NIAID AIDSno	86125	9.301
NIAID AIDSno	86126	9.301
NIAID AIDSno	86127	9.301
NIAID AIDSno	86128	8.678
NIAID AIDSno	86129	9.301
NIAID AIDSno	86130	9.301
NIAID AIDSno	86131	9.301
NIAID AIDSno	86132	9.301

NIAID AIDSno	86133	9.301
NIAID AIDSno	86135	8.131
NIAID AIDSno	86137	8.004
NIAID AIDSno	86138	7.745
NIAID AIDSno	86255	8.699
NIAID AIDSno	86256	7.924
NIAID AIDSno	86457	7.824
NIAID AIDSno	86590	8.602
NIAID AIDSno	86591	7.678
NIAID AIDSno	86741	9.301
NIAID AIDSno	87050	4.699
NIAID AIDSno	87536	4.203
NIAID AIDSno	87919	4.301
NIAID AIDSno	87925	4.697
NIAID AIDSno	87951	3.582
NIAID AIDSno	87952	3.710
NIAID AIDSno	87953	4.793
NIAID AIDSno	87954	4.481
NIAID AIDSno	87955	3.644
NIAID AIDSno	87956	3.680
NIAID AIDSno	88152	8.022
NIAID AIDSno	88153	7.721
NIAID AIDSno	88154	7.523
NIAID AIDSno	88156	8.337
NIAID AIDSno	88157	8.097
NIAID AIDSno	88158	8.143
NIAID AIDSno	88159	7.481
NIAID AIDSno	88160	6.471
NIAID AIDSno	88161	8.585
NIAID AIDSno	88162	8.229
NIAID AIDSno	88163	8.481
NIAID AIDSno	88164	9.000
NIAID AIDSno	88166	8.167
NIAID AIDSno	88167	7.699
NIAID AIDSno	88168	8.222
NIAID AIDSno	88169	8.194
NIAID AIDSno	88170	8.538
NIAID AIDSno	88171	8.420
NIAID AIDSno	88172	8.553
NIAID AIDSno	88173	8.538
NIAID AIDSno	88174	8.959
NIAID AIDSno	88175	8.658
NIAID AIDSno	88177	8.588
NIAID AIDSno	88711	7.939
NIAID AIDSno	88712	7.939

NIAID AIDSno	88713	7.939
NIAID AIDSno	88714	9.000
NIAID AIDSno	88715	9.000
NIAID AIDSno	88716	9.000
NIAID AIDSno	88845	8.569
NIAID AIDSno	88876	8.208
NIAID AIDSno	89068	7.155
NIAID AIDSno	89069	6.060
NIAID AIDSno	89070	6.824
NIAID AIDSno	89071	6.886
NIAID AIDSno	89206	8.398
NIAID AIDSno	89207	8.745
NIAID AIDSno	89208	8.387
NIAID AIDSno	89209	8.276
NIAID AIDSno	89210	7.738
NIAID AIDSno	89212	8.658
NIAID AIDSno	89213	8.585
NIAID AIDSno	89214	8.585
NIAID AIDSno	89216	8.013
NIAID AIDSno	89217	8.824
NIAID AIDSno	89218	8.456
NIAID AIDSno	89219	7.910
NIAID AIDSno	89221	8.201
NIAID AIDSno	89222	8.721
NIAID AIDSno	89223	8.444
NIAID AIDSno	89224	8.066
NIAID AIDSno	89228	8.041
NIAID AIDSno	89229	8.444
NIAID AIDSno	89230	8.523
NIAID AIDSno	89231	8.456
NIAID AIDSno	89232	8.721
NIAID AIDSno	89234	8.658
NIAID AIDSno	89235	7.983
NIAID AIDSno	89236	8.018
NIAID AIDSno	89237	8.347
NIAID AIDSno	89238	7.662
NIAID AIDSno	89239	8.620
NIAID AIDSno	89240	8.301
NIAID AIDSno	89241	8.456
NIAID AIDSno	89242	8.009
NIAID AIDSno	89243	7.772
NIAID AIDSno	89244	8.699
NIAID AIDSno	89245	6.883
NIAID AIDSno	89246	7.678
NIAID AIDSno	89247	7.538

NIAID AIDSno	89248	8.097
NIAID AIDSno	89249	8.658
NIAID AIDSno	89250	8.444
NIAID AIDSno	89251	8.523
NIAID AIDSno	89252	8.174
NIAID AIDSno	89253	7.854
NIAID AIDSno	89254	8.387
NIAID AIDSno	89255	8.658
NIAID AIDSno	89256	7.733
NIAID AIDSno	89257	8.444
NIAID AIDSno	89258	8.770
NIAID AIDSno	89259	8.481
NIAID AIDSno	89548	5.097
NIAID AIDSno	89549	5.553
NIAID AIDSno	89550	3.620
NIAID AIDSno	89551	4.620
NIAID AIDSno	89552	4.398
NIAID AIDSno	89553	4.323
NIAID AIDSno	89554	4.612
NIAID AIDSno	92663	7.921
NIAID AIDSno	92664	8.046
NIAID AIDSno	92665	5.398
NIAID AIDSno	92666	7.824
NIAID AIDSno	92667	8.301
NIAID AIDSno	92669	6.301
NIAID AIDSno	92670	4.602
NIAID AIDSno	92671	4.046
NIAID AIDSno	92672	5.000
NIAID AIDSno	92674	6.222
NIAID AIDSno	92675	6.602
NIAID AIDSno	92676	6.886
NIAID AIDSno	92677	8.301
NIAID AIDSno	92678	5.398
NIAID AIDSno	92679	5.398
NIAID AIDSno	92680	5.699
NIAID AIDSno	92681	6.886
NIAID AIDSno	92682	8.495
NIAID AIDSno	92683	6.301
NIAID AIDSno	92684	8.046
NIAID AIDSno	93719	8.398
NIAID AIDSno	94296	8.921
NIAID AIDSno	94297	9.029
NIAID AIDSno	94298	9.824
NIAID AIDSno	94299	7.770
NIAID AIDSno	94413	7.466

NIAID AIDSno	94414	7.553
NIAID AIDSno	94415	7.318
NIAID AIDSno	94416	7.962
NIAID AIDSno	94417	7.210
NIAID AIDSno	94418	6.297
NIAID AIDSno	94419	7.553
NIAID AIDSno	94420	7.131
NIAID AIDSno	94421	6.440
NIAID AIDSno	94422	6.710
NIAID AIDSno	94423	6.538
NIAID AIDSno	94424	9.000
NIAID AIDSno	95135	4.284
NIAID AIDSno	95136	4.301
NIAID AIDSno	95137	7.187
NIAID AIDSno	95138	7.523
NIAID AIDSno	95139	6.444
NIAID AIDSno	95140	6.301
NIAID AIDSno	95141	7.481
NIAID AIDSno	95143	5.699
NIAID AIDSno	95168	4.155
NIAID AIDSno	95169	4.155
NIAID AIDSno	95170	4.155
NIAID AIDSno	95171	4.155
NIAID AIDSno	95172	4.155
NIAID AIDSno	95173	4.155
NIAID AIDSno	95564	13.319
NIAID AIDSno	95565	12.796
NIAID AIDSno	95571	12.921
NIAID AIDSno	95590	12.585
NIAID AIDSno	95593	12.678
NIAID AIDSno	95674	13.268
NIAID AIDSno	95923	6.301
NIAID AIDSno	95982	5.620
NIAID AIDSno	95983	5.357
NIAID AIDSno	95985	5.860
NIAID AIDSno	95986	5.367
NIAID AIDSno	95987	5.810
NIAID AIDSno	95988	5.385
NIAID AIDSno	95989	6.201
NIAID AIDSno	95990	5.866
NIAID AIDSno	95991	5.703
NIAID AIDSno	95992	6.143
NIAID AIDSno	95993	5.676
NIAID AIDSno	95994	6.268
NIAID AIDSno	95995	5.857

NIAID AIDSno	95997	6.796
NIAID AIDSno	96242	9.854
NIAID AIDSno	96243	8.469
NIAID AIDSno	96244	9.208
NIAID AIDSno	96245	9.310
NIAID AIDSno	96246	8.959
NIAID AIDSno	96247	9.585
NIAID AIDSno	96248	9.602
NIAID AIDSno	96249	9.569
NIAID AIDSno	96250	9.167
NIAID AIDSno	96251	9.783
NIAID AIDSno	96252	8.770
NIAID AIDSno	96253	9.854
NIAID AIDSno	96254	9.456
NIAID AIDSno	96255	8.770
NIAID AIDSno	96256	9.328
NIAID AIDSno	96282	8.770
NIAID AIDSno	96283	9.959
NIAID AIDSno	96284	9.495
NIAID AIDSno	96285	9.569
NIAID AIDSno	96286	9.745
NIAID AIDSno	96287	9.409
NIAID AIDSno	96288	9.648
NIAID AIDSno	96289	9.721
NIAID AIDSno	96290	9.699
NIAID AIDSno	96291	9.959
NIAID AIDSno	96427	4.149
NIAID AIDSno	96888	7.164
NIAID AIDSno	96890	6.061
NIAID AIDSno	96891	6.243
NIAID AIDSno	96892	7.678
NIAID AIDSno	96893	7.758
NIAID AIDSno	96894	7.844
NIAID AIDSno	96895	7.921
NIAID AIDSno	96896	7.927
NIAID AIDSno	96897	7.979
NIAID AIDSno	96898	7.979
NIAID AIDSno	96899	8.569
NIAID AIDSno	96900	7.301
NIAID AIDSno	96902	7.301
NIAID AIDSno	96903	7.301
NIAID AIDSno	96943	7.048
NIAID AIDSno	96946	7.699
NIAID AIDSno	96948	7.594
NIAID AIDSno	96950	5.000

NIAID AIDSno	96952	7.000
NIAID AIDSno	96953	5.301
NIAID AIDSno	96975	4.903
NIAID AIDSno	97251	5.000
NIAID AIDSno	97252	5.000
NIAID AIDSno	97253	5.000
NIAID AIDSno	97254	5.000
NIAID AIDSno	97256	5.000
NIAID AIDSno	97264	5.000
NIAID AIDSno	97292	5.000
NIAID AIDSno	97297	5.000
NIAID AIDSno	97301	5.000
NIAID AIDSno	97304	5.000
NIAID AIDSno	97305	5.000
NIAID AIDSno	97312	5.000
NIAID AIDSno	97325	5.000
NIAID AIDSno	97337	5.000
NIAID AIDSno	97340	5.000
NIAID AIDSno	97354	5.000
NIAID AIDSno	97371	5.000
NIAID AIDSno	97408	5.000
NIAID AIDSno	97417	5.000
NIAID AIDSno	97463	5.000
NIAID AIDSno	97480	5.000
NIAID AIDSno	98111	5.523
NIAID AIDSno	98112	5.125
NIAID AIDSno	98113	5.523
NIAID AIDSno	98114	5.398
NIAID AIDSno	98115	5.252
NIAID AIDSno	98118	4.699
NIAID AIDSno	98119	5.022
NIAID AIDSno	98120	5.260
NIAID AIDSno	98123	5.260
NIAID AIDSno	98124	5.398
NIAID AIDSno	98125	5.600
NIAID AIDSno	98126	5.489
NIAID AIDSno	98129	5.222
NIAID AIDSno	98131	5.481
NIAID AIDSno	98133	4.726
NIAID AIDSno	98134	5.638
NIAID AIDSno	98135	5.770
NIAID AIDSno	98136	5.770
NIAID AIDSno	104007	7.824
NIAID AIDSno	104008	7.886
NIAID AIDSno	104009	8.523

NIAID AIDSno	104010	7.651
NIAID AIDSno	104011	7.187
NIAID AIDSno	104012	7.886
NIAID AIDSno	104100	5.367
NIAID AIDSno	104101	5.291
NIAID AIDSno	104102	5.538
NIAID AIDSno	104103	6.051
NIAID AIDSno	104104	5.654
NIAID AIDSno	104105	5.489
NIAID AIDSno	104106	5.785
NIAID AIDSno	104107	6.180
NIAID AIDSno	104108	7.347
NIAID AIDSno	104109	7.000
NIAID AIDSno	104110	7.137
NIAID AIDSno	104111	6.796
NIAID AIDSno	104112	6.456
NIAID AIDSno	104293	7.301
NIAID AIDSno	104294	7.301
NIAID AIDSno	104295	7.301
NIAID AIDSno	104296	7.301
NIAID AIDSno	104297	7.301
NIAID AIDSno	104298	7.301
NIAID AIDSno	104299	7.301
NIAID AIDSno	104302	5.301
NIAID AIDSno	104314	9.301
NIAID AIDSno	104315	9.301
NIAID AIDSno	104316	9.301
NIAID AIDSno	104317	9.301
NIAID AIDSno	104318	9.301
NIAID AIDSno	104319	9.301
NIAID AIDSno	104320	9.301
NIAID AIDSno	104321	9.301
NIAID AIDSno	104322	9.301
NIAID AIDSno	104323	9.301
NIAID AIDSno	104324	9.301
NIAID AIDSno	104325	9.301
NIAID AIDSno	104326	9.301
NIAID AIDSno	104327	9.301
NIAID AIDSno	104328	9.301
NIAID AIDSno	104329	9.301
NIAID AIDSno	104330	9.301
NIAID AIDSno	104331	9.301
NIAID AIDSno	104332	9.301
NIAID AIDSno	104333	9.301
NIAID AIDSno	104334	9.301

NIAID AIDSno	104335	9.301
NIAID AIDSno	104336	9.301
NIAID AIDSno	104337	9.301
NIAID AIDSno	104338	9.301
NIAID AIDSno	104339	9.301
NIAID AIDSno	104340	9.301
NIAID AIDSno	104341	9.301
NIAID AIDSno	104342	9.301
NIAID AIDSno	104343	9.301
NIAID AIDSno	104344	9.301
NIAID AIDSno	104345	9.301
NIAID AIDSno	104346	9.301
NIAID AIDSno	104347	9.301
NIAID AIDSno	104348	9.301
NIAID AIDSno	104349	9.301
NIAID AIDSno	104351	9.301
NIAID AIDSno	104352	9.301
NIAID AIDSno	104353	9.301
NIAID AIDSno	104354	9.301
NIAID AIDSno	104355	9.301
NIAID AIDSno	104356	9.301
NIAID AIDSno	104357	9.301
NIAID AIDSno	104909	7.699
NIAID AIDSno	104910	6.921
NIAID AIDSno	104911	6.140
NIAID AIDSno	104912	7.260
NIAID AIDSno	104913	7.060
NIAID AIDSno	104914	6.310
NIAID AIDSno	104915	5.921
NIAID AIDSno	104963	5.699
NIAID AIDSno	104964	5.000
NIAID AIDSno	105703	9.770
NIAID AIDSno	105773	8.959
NIAID AIDSno	105774	8.237
NIAID AIDSno	105775	9.310
NIAID AIDSno	105776	8.886
NIAID AIDSno	105777	8.638
NIAID AIDSno	105778	9.585
NIAID AIDSno	105779	8.886
NIAID AIDSno	105780	8.244
NIAID AIDSno	105781	8.444
NIAID AIDSno	105782	7.523
NIAID AIDSno	105783	8.721
NIAID AIDSno	105784	7.602
NIAID AIDSno	105785	8.367

NIAID AIDSno	105786	8.000
NIAID AIDSno	105788	4.166
NIAID AIDSno	105859	7.022
NIAID AIDSno	105860	7.886
NIAID AIDSno	105861	8.000
NIAID AIDSno	105862	8.155
NIAID AIDSno	105863	8.222
NIAID AIDSno	105865	6.600
NIAID AIDSno	105867	4.854
NIAID AIDSno	105868	3.947
NIAID AIDSno	105869	4.149
NIAID AIDSno	105870	6.674
NIAID AIDSno	105871	7.886
NIAID AIDSno	106202	7.602
NIAID AIDSno	106285	5.125
NIAID AIDSno	106330	4.796
NIAID AIDSno	106331	5.377
NIAID AIDSno	106333	5.523
NIAID AIDSno	106334	4.469
NIAID AIDSno	106338	5.577
NIAID AIDSno	106341	5.000
NIAID AIDSno	106351	5.000
NIAID AIDSno	106352	7.699
NIAID AIDSno	106353	7.824
NIAID AIDSno	106354	6.921
NIAID AIDSno	106355	6.260
NIAID AIDSno	106356	5.292
NIAID AIDSno	106357	5.319
NIAID AIDSno	106500	7.155
NIAID AIDSno	106502	9.469
NIAID AIDSno	106503	9.456
NIAID AIDSno	106506	9.240
NIAID AIDSno	106507	8.886
NIAID AIDSno	106508	9.620
NIAID AIDSno	106509	9.009
NIAID AIDSno	106510	9.143
NIAID AIDSno	106511	8.854
NIAID AIDSno	106513	9.387
NIAID AIDSno	106514	9.347
NIAID AIDSno	106515	8.720
NIAID AIDSno	106518	9.097
NIAID AIDSno	106519	9.022
NIAID AIDSno	107006	4.000
NIAID AIDSno	107007	4.000
NIAID AIDSno	107008	4.000

NIAID AIDSno	107030	4.125
NIAID AIDSno	107031	5.046
NIAID AIDSno	107032	4.469
NIAID AIDSno	107033	4.678
NIAID AIDSno	107038	7.301
NIAID AIDSno	107039	7.602
NIAID AIDSno	107040	6.301
NIAID AIDSno	107041	7.000
NIAID AIDSno	107042	5.801
NIAID AIDSno	107043	7.301
NIAID AIDSno	107047	6.523
NIAID AIDSno	107048	6.398
NIAID AIDSno	107049	6.699
NIAID AIDSno	107050	6.638
NIAID AIDSno	107051	6.155
NIAID AIDSno	107054	6.523
NIAID AIDSno	107055	6.046
NIAID AIDSno	107056	3.522
NIAID AIDSno	107057	7.000
NIAID AIDSno	107058	7.000
NIAID AIDSno	107065	3.682
NIAID AIDSno	107067	4.553
NIAID AIDSno	107932	7.331
NIAID AIDSno	107933	8.035
NIAID AIDSno	107934	7.099
NIAID AIDSno	107935	7.003
NIAID AIDSno	107936	6.872
NIAID AIDSno	107937	7.104
NIAID AIDSno	107938	7.283
NIAID AIDSno	107939	7.288
NIAID AIDSno	107940	7.583
NIAID AIDSno	107941	4.835
NIAID AIDSno	107942	5.699
NIAID AIDSno	107943	6.796
NIAID AIDSno	107944	7.854
NIAID AIDSno	107945	6.714
NIAID AIDSno	107946	5.349
NIAID AIDSno	107947	7.361
NIAID AIDSno	107948	6.371
NIAID AIDSno	107949	6.554
NIAID AIDSno	107951	6.018
NIAID AIDSno	107952	6.801
NIAID AIDSno	107953	7.168
NIAID AIDSno	107954	6.921
NIAID AIDSno	107955	7.460

NIAID AIDSno	107956	7.708
NIAID AIDSno	107957	7.466
NIAID AIDSno	107958	7.418
NIAID AIDSno	107997	6.523
NIAID AIDSno	107999	7.796
NIAID AIDSno	108000	8.921
NIAID AIDSno	108001	9.097
NIAID AIDSno	108246	4.378
NIAID AIDSno	108257	3.301
NIAID AIDSno	108259	3.301
NIAID AIDSno	108261	4.276
NIAID AIDSno	108265	4.102
NIAID AIDSno	108267	4.796
NIAID AIDSno	108268	4.357
NIAID AIDSno	108269	3.747
NIAID AIDSno	108270	3.907
NIAID AIDSno	108271	4.009
NIAID AIDSno	108631	9.301
NIAID AIDSno	108982	5.013
NIAID AIDSno	108983	5.387
NIAID AIDSno	108984	5.244
NIAID AIDSno	108985	5.745
NIAID AIDSno	108986	6.367
NIAID AIDSno	108987	5.538
NIAID AIDSno	108988	5.523
NIAID AIDSno	108989	5.409
NIAID AIDSno	108991	4.523
NIAID AIDSno	109009	4.402
NIAID AIDSno	109075	5.301
NIAID AIDSno	109111	9.553
NIAID AIDSno	109112	8.301
NIAID AIDSno	109114	9.237
NIAID AIDSno	109115	7.854
NIAID AIDSno	109116	9.721
NIAID AIDSno	109117	7.886
NIAID AIDSno	109118	7.854
NIAID AIDSno	109119	8.796
NIAID AIDSno	109120	8.238
NIAID AIDSno	109121	7.745
NIAID AIDSno	109290	6.137
NIAID AIDSno	109291	6.319
NIAID AIDSno	109292	6.220
NIAID AIDSno	109294	6.051
NIAID AIDSno	109295	5.991
NIAID AIDSno	109296	5.876

NIAID AIDSno	109297	5.684
NIAID AIDSno	109298	5.553
NIAID AIDSno	109299	6.032
NIAID AIDSno	109300	5.658
NIAID AIDSno	109301	5.469
NIAID AIDSno	109504	9.301
NIAID AIDSno	109505	9.301
NIAID AIDSno	109507	9.301
NIAID AIDSno	109508	9.301
NIAID AIDSno	109509	9.301
NIAID AIDSno	109510	9.301
NIAID AIDSno	109539	3.595
NIAID AIDSno	109540	3.654
NIAID AIDSno	109541	4.678
NIAID AIDSno	109542	4.538
NIAID AIDSno	109543	4.917
NIAID AIDSno	109544	4.959
NIAID AIDSno	110004	5.959
NIAID AIDSno	110006	5.721
NIAID AIDSno	110007	5.036
NIAID AIDSno	110008	4.745
NIAID AIDSno	110009	4.268
NIAID AIDSno	110010	4.553
NIAID AIDSno	110011	5.013
NIAID AIDSno	110012	5.264
NIAID AIDSno	110013	5.301
NIAID AIDSno	110014	3.996
NIAID AIDSno	110015	4.745
NIAID AIDSno	110016	4.851
NIAID AIDSno	110017	4.959
NIAID AIDSno	110018	5.086
NIAID AIDSno	110019	6.167
NIAID AIDSno	110816	7.046
NIAID AIDSno	110817	7.770
NIAID AIDSno	110818	6.699
NIAID AIDSno	110819	7.602
NIAID AIDSno	110820	7.097
NIAID AIDSno	110857	9.222
NIAID AIDSno	110858	8.444
NIAID AIDSno	110859	9.602
NIAID AIDSno	110860	9.745
NIAID AIDSno	110861	9.229
NIAID AIDSno	110862	9.119
NIAID AIDSno	110863	8.796
NIAID AIDSno	110864	10.000

NIAID AIDSno	110865	9.328
NIAID AIDSno	110866	10.398
NIAID AIDSno	110867	9.959
NIAID AIDSno	110868	9.495
NIAID AIDSno	110869	8.398
NIAID AIDSno	110870	7.222
NIAID AIDSno	110873	8.244
NIAID AIDSno	110875	8.301
NIAID AIDSno	110876	8.745
NIAID AIDSno	110877	8.301
NIAID AIDSno	110878	9.000
NIAID AIDSno	110879	8.000
NIAID AIDSno	110880	8.000
NIAID AIDSno	110881	8.000
NIAID AIDSno	110882	7.824
NIAID AIDSno	110883	7.886
NIAID AIDSno	110884	8.222
NIAID AIDSno	110885	8.398
NIAID AIDSno	111151	6.947
NIAID AIDSno	111152	6.492
NIAID AIDSno	111153	6.084
NIAID AIDSno	111154	6.640
NIAID AIDSno	111155	6.914
NIAID AIDSno	111156	7.523
NIAID AIDSno	111157	7.284
NIAID AIDSno	111158	7.638
NIAID AIDSno	112388	7.301
NIAID AIDSno	112389	8.699
NIAID AIDSno	112390	7.824
NIAID AIDSno	112391	8.222
NIAID AIDSno	112392	7.155
NIAID AIDSno	112393	6.000
NIAID AIDSno	112394	8.097
NIAID AIDSno	112395	4.319
NIAID AIDSno	112397	7.824
NIAID AIDSno	112398	7.523
NIAID AIDSno	112400	7.155
NIAID AIDSno	112402	7.155
NIAID AIDSno	112403	7.523
NIAID AIDSno	112404	7.097
NIAID AIDSno	112406	7.523
NIAID AIDSno	112407	6.602
NIAID AIDSno	112408	6.854
NIAID AIDSno	112409	7.125
NIAID AIDSno	112410	7.046

NIAID AIDSno	112411	8.301
NIAID AIDSno	112412	7.523
NIAID AIDSno	112413	8.000
NIAID AIDSno	112414	5.699
NIAID AIDSno	112415	6.398
NIAID AIDSno	112417	8.000
NIAID AIDSno	112418	7.960
NIAID AIDSno	112419	6.347
NIAID AIDSno	112420	6.071
NIAID AIDSno	112421	5.921
NIAID AIDSno	112430	4.237
NIAID AIDSno	112444	6.523
NIAID AIDSno	113252	7.601
NIAID AIDSno	113253	7.456
NIAID AIDSno	113254	7.854
NIAID AIDSno	113255	6.959
NIAID AIDSno	113287	8.959
NIAID AIDSno	113288	9.602
NIAID AIDSno	113289	8.854
NIAID AIDSno	113292	8.824
NIAID AIDSno	113293	9.602
NIAID AIDSno	113667	8.745
NIAID AIDSno	113932	4.553
NIAID AIDSno	113935	5.071
NIAID AIDSno	113936	5.523
NIAID AIDSno	113937	4.481
NIAID AIDSno	115733	8.268
NIAID AIDSno	115738	8.296
NIAID AIDSno	115743	5.481
NIAID AIDSno	115744	7.903
NIAID AIDSno	121149	5.260
NIAID AIDSno	121150	4.886
NIAID AIDSno	121151	5.398
NIAID AIDSno	121153	4.276
NIAID AIDSno	121154	4.420
NIAID AIDSno	121155	4.357
NIAID AIDSno	121156	5.398
NIAID AIDSno	121157	5.194
NIAID AIDSno	121158	5.409
NIAID AIDSno	121161	9.301
NIAID AIDSno	121162	9.301
NIAID AIDSno	121163	9.301
NIAID AIDSno	121165	9.301
NIAID AIDSno	121166	9.301
NIAID AIDSno	121168	9.301

NIAID AIDSno	121169	9.301
NIAID AIDSno	121170	9.301
NIAID AIDSno	121171	9.301
NIAID AIDSno	121172	9.301
NIAID AIDSno	121597	8.699
NIAID AIDSno	121598	8.523
NIAID AIDSno	123218	4.919
NIAID AIDSno	123222	5.048
NIAID AIDSno	123384	5.111
NIAID AIDSno	123385	5.349
NIAID AIDSno	123386	5.314
NIAID AIDSno	123387	5.332
NIAID AIDSno	123567	10.111
NIAID AIDSno	123568	9.936
NIAID AIDSno	123569	9.215
NIAID AIDSno	123570	9.398
NIAID AIDSno	123571	9.222
NIAID AIDSno	123572	9.301
NIAID AIDSno	123573	9.432
NIAID AIDSno	123574	7.860
NIAID AIDSno	123575	8.660
NIAID AIDSno	123576	9.187
NIAID AIDSno	123577	10.155
NIAID AIDSno	123578	9.854
NIAID AIDSno	123579	8.796
NIAID AIDSno	128174	5.456
NIAID AIDSno	147874	5.097
NIAID AIDSno	153897	8.936
NIAID AIDSno	153898	9.824
NIAID AIDSno	154348	5.301
NIAID AIDSno	154349	4.509
NIAID AIDSno	154350	5.699
NIAID AIDSno	154351	5.155
NIAID AIDSno	154352	5.022
NIAID AIDSno	154353	5.620
NIAID AIDSno	154354	5.523
NIAID AIDSno	154355	5.398
NIAID AIDSno	154356	5.046
NIAID AIDSno	154357	5.125
NIAID AIDSno	155413	8.398
NIAID AIDSno	155414	8.523
NIAID AIDSno	155415	8.398
NIAID AIDSno	155416	9.412
NIAID AIDSno	155599	9.301
NIAID AIDSno	155600	9.301

NIAID AIDSno	155601	9.301
NIAID AIDSno	155602	9.301
NIAID AIDSno	155603	9.301
NIAID AIDSno	155864	5.155
NIAID AIDSno	155881	4.469
NIAID AIDSno	155888	4.538
NIAID AIDSno	159646	3.939
NIAID AIDSno	159647	4.244
NIAID AIDSno	159648	4.143
NIAID AIDSno	159649	4.602
NIAID AIDSno	159658	3.759
NIAID AIDSno	159659	3.793
NIAID AIDSno	159660	3.928
NIAID AIDSno	159663	3.699
NIAID AIDSno	160223	4.229
NIAID AIDSno	161807	4.031
NIAID AIDSno	163612	3.000
NIAID AIDSno	163617	3.000
NIAID AIDSno	163619	3.000
NIAID AIDSno	163633	3.000
NIAID AIDSno	163641	5.301
NIAID AIDSno	163648	5.301
NIAID AIDSno	163650	3.000
NIAID AIDSno	163651	5.301
NIAID AIDSno	163674	5.301
NIAID AIDSno	163681	7.301
NIAID AIDSno	163682	7.301
NIAID AIDSno	163684	7.301
NIAID AIDSno	163692	7.301
NIAID AIDSno	163693	7.301
NIAID AIDSno	163694	7.301
NIAID AIDSno	163695	7.301
NIAID AIDSno	163696	7.301
NIAID AIDSno	163697	7.301
NIAID AIDSno	163698	7.301
NIAID AIDSno	163699	7.301
NIAID AIDSno	163701	7.301
NIAID AIDSno	163702	7.301
NIAID AIDSno	163703	7.301
NIAID AIDSno	163704	7.301
NIAID AIDSno	163707	7.301
NIAID AIDSno	163708	7.301
NIAID AIDSno	163709	7.301
NIAID AIDSno	163710	7.301
NIAID AIDSno	163712	7.301

NIAID AIDSno	163713	7.301
NIAID AIDSno	163714	7.301
NIAID AIDSno	163715	7.301
NIAID AIDSno	163716	7.301
NIAID AIDSno	163719	7.301
NIAID AIDSno	163727	7.301
NIAID AIDSno	163731	7.301
NIAID AIDSno	163732	7.301
NIAID AIDSno	163737	7.301
NIAID AIDSno	163738	7.301
NIAID AIDSno	163739	7.301
NIAID AIDSno	163740	7.301
NIAID AIDSno	163741	7.301
NIAID AIDSno	163742	7.301
NIAID AIDSno	163743	7.301
NIAID AIDSno	163744	7.301
NIAID AIDSno	163745	7.301
NIAID AIDSno	163746	7.301
NIAID AIDSno	163749	7.301
NIAID AIDSno	163750	7.301
NIAID AIDSno	163752	7.301
NIAID AIDSno	163754	7.301
NIAID AIDSno	163755	7.301
NIAID AIDSno	163760	7.301
NIAID AIDSno	163761	7.301
NIAID AIDSno	164093	5.495
NIAID AIDSno	164094	5.398
NIAID AIDSno	164095	5.721
NIAID AIDSno	164096	4.796
NIAID AIDSno	164097	5.821
NIAID AIDSno	164098	4.898
NIAID AIDSno	165125	7.301
NIAID AIDSno	165126	6.398
NIAID AIDSno	165128	6.523
NIAID AIDSno	165132	6.708
NIAID AIDSno	165148	7.232
NIAID AIDSno	165150	7.222
NIAID AIDSno	165162	7.097
NIAID AIDSno	165173	6.611
NIAID AIDSno	165178	6.638
NIAID AIDSno	165180	7.000
NIAID AIDSno	165248	8.022
NIAID AIDSno	165249	6.398
NIAID AIDSno	165250	6.337
NIAID AIDSno	165251	7.523

NIAID AIDSno	165252	8.000
NIAID AIDSno	165253	7.276
NIAID AIDSno	165254	5.678
NIAID AIDSno	165255	8.409
NIAID AIDSno	165256	8.398
NIAID AIDSno	165257	8.509
NIAID AIDSno	165258	8.432
NIAID AIDSno	165259	8.201
NIAID AIDSno	165260	8.387
NIAID AIDSno	165261	8.638
NIAID AIDSno	165262	8.538
NIAID AIDSno	165263	8.569
NIAID AIDSno	165264	8.602
NIAID AIDSno	165265	8.745
NIAID AIDSno	165266	8.097
NIAID AIDSno	165267	8.509
NIAID AIDSno	165268	8.268
NIAID AIDSno	165269	8.328
NIAID AIDSno	165270	8.745
NIAID AIDSno	165271	8.638
NIAID AIDSno	165272	8.721
NIAID AIDSno	165273	8.456
NIAID AIDSno	165274	8.310
NIAID AIDSno	165275	7.796
NIAID AIDSno	165276	8.097
NIAID AIDSno	165277	7.721
NIAID AIDSno	165278	7.921
NIAID AIDSno	165279	8.000
NIAID AIDSno	165280	8.495
NIAID AIDSno	165281	8.602
NIAID AIDSno	165282	8.432
NIAID AIDSno	165283	8.523
NIAID AIDSno	165284	8.638
NIAID AIDSno	165285	8.367
NIAID AIDSno	165286	8.444
NIAID AIDSno	165287	8.553
NIAID AIDSno	165288	8.409
NIAID AIDSno	165289	8.569
NIAID AIDSno	165290	8.824
NIAID AIDSno	165291	8.721
NIAID AIDSno	165292	8.469
NIAID AIDSno	165293	8.377
NIAID AIDSno	165294	8.495
NIAID AIDSno	165295	8.398
NIAID AIDSno	165296	8.347

NIAID AIDSno	165297	8.444
NIAID AIDSno	165298	8.538
NIAID AIDSno	165299	8.585
NIAID AIDSno	165300	8.620
NIAID AIDSno	165301	8.745
NIAID AIDSno	165302	8.337
NIAID AIDSno	165303	8.770
NIAID AIDSno	165304	8.770
NIAID AIDSno	165305	8.620
NIAID AIDSno	165306	8.553
NIAID AIDSno	165307	8.432
NIAID AIDSno	165308	8.523
NIAID AIDSno	165309	8.658
NIAID AIDSno	165310	8.678
NIAID AIDSno	165311	8.432
NIAID AIDSno	165312	8.658
NIAID AIDSno	165313	8.585
NIAID AIDSno	165314	8.678
NIAID AIDSno	165315	8.538
NIAID AIDSno	165316	8.432
NIAID AIDSno	165317	7.469
NIAID AIDSno	165318	8.208
NIAID AIDSno	165319	8.268
NIAID AIDSno	165320	8.009
NIAID AIDSno	165321	7.854
NIAID AIDSno	165322	8.167
NIAID AIDSno	165965	10.155
NIAID AIDSno	166076	9.699
NIAID AIDSno	166077	9.699
NIAID AIDSno	166078	9.301
NIAID AIDSno	166079	10.000
NIAID AIDSno	166091	9.097
NIAID AIDSno	166147	9.284
NIAID AIDSno	166148	8.301
NIAID AIDSno	166150	10.398
NIAID AIDSno	166151	10.523
NIAID AIDSno	166152	10.398
NIAID AIDSno	166153	9.620
NIAID AIDSno	166154	9.886
NIAID AIDSno	166155	9.796
NIAID AIDSno	166156	10.168
NIAID AIDSno	166157	10.324
NIAID AIDSno	166180	6.959
NIAID AIDSno	166181	7.639
NIAID AIDSno	166182	7.492

NIAID AIDSno	166184	7.570
NIAID AIDSno	166185	7.315
NIAID AIDSno	166186	7.519
NIAID AIDSno	166187	7.824
NIAID AIDSno	166188	7.866
NIAID AIDSno	167463	5.432
NIAID AIDSno	167464	5.654
NIAID AIDSno	167465	6.432
NIAID AIDSno	167466	5.932
NIAID AIDSno	167467	6.009
NIAID AIDSno	167468	5.996
NIAID AIDSno	167866	5.578
NIAID AIDSno	167870	5.762
NIAID AIDSno	167871	6.208
NIAID AIDSno	167872	6.527
NIAID AIDSno	167873	6.438
NIAID AIDSno	167874	6.770
NIAID AIDSno	168990	8.485
NIAID AIDSno	169100	9.569
NIAID AIDSno	169101	8.824
NIAID AIDSno	169102	9.444
NIAID AIDSno	169103	9.060
NIAID AIDSno	169104	9.409
NIAID AIDSno	169105	9.301
NIAID AIDSno	169106	8.854
NIAID AIDSno	169107	9.097
NIAID AIDSno	169108	9.959
NIAID AIDSno	169109	9.959
NIAID AIDSno	169110	9.420
NIAID AIDSno	169111	9.658
NIAID AIDSno	169112	9.921
NIAID AIDSno	169113	10.215
NIAID AIDSno	169114	9.921
NIAID AIDSno	169115	9.387
NIAID AIDSno	170273	8.036
NIAID AIDSno	170274	7.357
NIAID AIDSno	170275	7.959
NIAID AIDSno	170276	8.721
NIAID AIDSno	170277	7.097
NIAID AIDSno	170278	8.071
NIAID AIDSno	170279	8.538
NIAID AIDSno	170281	6.699
NIAID AIDSno	170282	8.066
NIAID AIDSno	170285	7.222
NIAID AIDSno	170286	9.301

NIAID AIDSno	170733	6.506
NIAID AIDSno	170734	6.745
NIAID AIDSno	170735	6.523
NIAID AIDSno	170736	7.143
NIAID AIDSno	171202	5.000
NIAID AIDSno	171203	6.000
NIAID AIDSno	171205	8.155
NIAID AIDSno	171207	8.180
NIAID AIDSno	171208	5.000
NIAID AIDSno	171209	5.770
NIAID AIDSno	171210	5.570
NIAID AIDSno	171917	9.301
NIAID AIDSno	171918	9.886
NIAID AIDSno	171919	9.854
NIAID AIDSno	171920	10.000
NIAID AIDSno	171921	10.398
NIAID AIDSno	171922	10.398
NIAID AIDSno	171923	10.699
NIAID AIDSno	171924	10.699
NIAID AIDSno	179702	7.699
NIAID AIDSno	179703	7.398
NIAID AIDSno	179704	7.699
NIAID AIDSno	179705	8.000
NIAID AIDSno	179706	7.585
NIAID AIDSno	179707	7.638
NIAID AIDSno	179708	8.155
NIAID AIDSno	179709	7.174
NIAID AIDSno	179710	7.745
NIAID AIDSno	179711	8.097
NIAID AIDSno	179713	6.886
NIAID AIDSno	179714	7.036
NIAID AIDSno	179717	7.328
NIAID AIDSno	179719	7.721
NIAID AIDSno	179720	8.000
NIAID AIDSno	179721	8.377
NIAID AIDSno	179722	8.456
NIAID AIDSno	179723	7.523
NIAID AIDSno	179724	7.699
NIAID AIDSno	179726	8.854
NIAID AIDSno	179727	7.000
NIAID AIDSno	179728	7.092
NIAID AIDSno	179729	7.699
NIAID AIDSno	179730	7.959
NIAID AIDSno	179731	7.796
NIAID AIDSno	179735	7.310

NIAID AIDSno	179736	7.000
NIAID AIDSno	179737	8.444
NIAID AIDSno	179739	8.523
NIAID AIDSno	179740	7.071
NIAID AIDSno	179741	7.276
NIAID AIDSno	179742	7.347
NIAID AIDSno	179743	8.611
NIAID AIDSno	179744	8.611
NIAID AIDSno	179745	8.460
NIAID AIDSno	179746	8.349
NIAID AIDSno	179747	8.173
NIAID AIDSno	179748	8.398
NIAID AIDSno	179749	8.097
NIAID AIDSno	179750	8.398
NIAID AIDSno	179751	7.137
NIAID AIDSno	179752	8.699
NIAID AIDSno	179753	7.222
NIAID AIDSno	179754	7.167
NIAID AIDSno	179755	8.301
NIAID AIDSno	179756	8.301
NIAID AIDSno	179757	7.745
NIAID AIDSno	179758	8.620
NIAID AIDSno	179759	8.745
NIAID AIDSno	179760	8.102
NIAID AIDSno	179761	8.796
NIAID AIDSno	179762	8.824
NIAID AIDSno	179763	8.854
NIAID AIDSno	179764	8.745
NIAID AIDSno	179766	6.886
NIAID AIDSno	179767	8.481
NIAID AIDSno	179768	8.402
NIAID AIDSno	180035	6.215
NIAID AIDSno	180036	6.398
NIAID AIDSno	180037	6.509
NIAID AIDSno	180038	6.260
NIAID AIDSno	180222	5.377
NIAID AIDSno	180243	7.921
NIAID AIDSno	184176	4.292
NIAID AIDSno	188026	10.097
NIAID AIDSno	188027	10.699
NIAID AIDSno	188028	10.398
NIAID AIDSno	188029	10.523
NIAID AIDSno	188030	10.155
NIAID AIDSno	188031	10.398
NIAID AIDSno	188032	10.000

NIAID AIDSno	188033	10.699
NIAID AIDSno	188034	10.523
NIAID AIDSno	188105	4.174
NIAID AIDSno	188981	5.959
NIAID AIDSno	188982	5.174
NIAID AIDSno	188983	6.119
NIAID AIDSno	188984	6.469
NIAID AIDSno	188985	5.886
NIAID AIDSno	188986	6.437
NIAID AIDSno	188987	6.268
NIAID AIDSno	188988	6.137
NIAID AIDSno	188989	6.470
NIAID AIDSno	188990	5.456
NIAID AIDSno	188991	6.467
NIAID AIDSno	188992	5.658
NIAID AIDSno	188993	6.000
NIAID AIDSno	188994	5.959
NIAID AIDSno	188995	5.523
NIAID AIDSno	188996	6.281
NIAID AIDSno	189012	5.936
NIAID AIDSno	189283	6.951
NIAID AIDSno	189284	7.921
NIAID AIDSno	189285	8.699
NIAID AIDSno	189286	8.523
NIAID AIDSno	189287	7.328
NIAID AIDSno	189288	6.000
NIAID AIDSno	189289	8.699
NIAID AIDSno	189290	8.699
NIAID AIDSno	189291	8.523
NIAID AIDSno	189292	8.523
NIAID AIDSno	189293	8.699
NIAID AIDSno	189294	8.398
NIAID AIDSno	189295	8.523
NIAID AIDSno	189296	8.699
NIAID AIDSno	189297	8.398
NIAID AIDSno	189298	8.398
NIAID AIDSno	189299	8.398
NIAID AIDSno	189300	8.699
NIAID AIDSno	189301	8.301
NIAID AIDSno	189302	8.398
NIAID AIDSno	189303	8.699
NIAID AIDSno	189304	8.222
NIAID AIDSno	189708	8.921
NIAID AIDSno	189709	8.921
NIAID AIDSno	189714	8.187

NIAID AIDSno	189719	9.283
NIAID AIDSno	189720	9.292
NIAID AIDSno	189751	8.824
NIAID AIDSno	189752	9.301
NIAID AIDSno	189753	9.167
NIAID AIDSno	189754	9.187
NIAID AIDSno	189755	9.367
NIAID AIDSno	189756	8.420
NIAID AIDSno	189774	9.357
NIAID AIDSno	189775	9.921
NIAID AIDSno	189776	9.337
NIAID AIDSno	189777	9.284
NIAID AIDSno	189778	9.367
NIAID AIDSno	189779	9.620
NIAID AIDSno	189780	9.319
NIAID AIDSno	189781	9.409
NIAID AIDSno	189782	9.276
NIAID AIDSno	189783	9.252
NIAID AIDSno	189784	9.201
NIAID AIDSno	189785	9.398
NIAID AIDSno	189786	9.301
NIAID AIDSno	189787	9.481
NIAID AIDSno	189788	9.046
NIAID AIDSno	189789	9.398
NIAID AIDSno	189790	9.301
NIAID AIDSno	189791	9.398
NIAID AIDSno	189792	9.398
NIAID AIDSno	190331	7.301
NIAID AIDSno	190340	7.301
NIAID AIDSno	190987	13.215
NIAID AIDSno	190988	12.284
NIAID AIDSno	190989	13.854
NIAID AIDSno	190990	13.237
NIAID AIDSno	192312	9.301
NIAID AIDSno	192313	9.301
NIAID AIDSno	192314	9.301
NIAID AIDSno	192315	9.301
NIAID AIDSno	192316	9.301
NIAID AIDSno	192317	9.301
NIAID AIDSno	192318	9.301
NIAID AIDSno	192319	9.301
NIAID AIDSno	192320	9.301
NIAID AIDSno	192321	9.301
NIAID AIDSno	192322	9.301
NIAID AIDSno	192323	9.301

NIAID AIDSno	192324	9.301
NIAID AIDSno	192325	9.301
NIAID AIDSno	192326	9.301
NIAID AIDSno	192327	9.301
NIAID AIDSno	192328	9.301
NIAID AIDSno	192329	9.000
NIAID AIDSno	192330	9.000
NIAID AIDSno	192331	9.000
NIAID AIDSno	192332	9.000
NIAID AIDSno	192333	9.000
NIAID AIDSno	192334	9.301
NIAID AIDSno	192335	9.301
NIAID AIDSno	192336	9.301
NIAID AIDSno	192337	9.301
NIAID AIDSno	192338	9.301
NIAID AIDSno	192339	9.301
NIAID AIDSno	192340	9.000
NIAID AIDSno	192341	9.301
NIAID AIDSno	192342	9.301
NIAID AIDSno	192343	9.301
NIAID AIDSno	192344	9.000
NIAID AIDSno	192345	9.301
NIAID AIDSno	192346	9.301
NIAID AIDSno	192347	9.301
NIAID AIDSno	192348	9.301
NIAID AIDSno	192349	9.301
NIAID AIDSno	192350	9.301
NIAID AIDSno	192351	9.301
NIAID AIDSno	192352	9.301
NIAID AIDSno	192353	9.301
NIAID AIDSno	192355	9.301
NIAID AIDSno	192356	9.301
NIAID AIDSno	192357	9.301
NIAID AIDSno	192358	9.301
NIAID AIDSno	192359	9.301
NIAID AIDSno	192360	9.301
NIAID AIDSno	192361	9.301
NIAID AIDSno	192362	9.301
NIAID AIDSno	192363	9.301
NIAID AIDSno	192364	9.301
NIAID AIDSno	192365	9.301
NIAID AIDSno	192366	9.301
NIAID AIDSno	192367	9.301
NIAID AIDSno	192368	9.301
NIAID AIDSno	192369	9.301

NIAID AIDSno	192370	9.301
NIAID AIDSno	192371	9.301
NIAID AIDSno	192372	9.301
NIAID AIDSno	192373	9.301
NIAID AIDSno	192374	9.301
NIAID AIDSno	192375	9.301
NIAID AIDSno	192376	9.301
NIAID AIDSno	192377	9.301
NIAID AIDSno	192378	9.301
NIAID AIDSno	192379	9.301
NIAID AIDSno	192380	9.301
NIAID AIDSno	192381	9.301
NIAID AIDSno	192382	9.301
NIAID AIDSno	192383	9.301
NIAID AIDSno	192384	9.301
NIAID AIDSno	192385	9.301
NIAID AIDSno	192386	9.301
NIAID AIDSno	192387	9.301
NIAID AIDSno	192388	9.301
NIAID AIDSno	192389	9.301
NIAID AIDSno	192390	9.301
NIAID AIDSno	192391	9.301
NIAID AIDSno	192392	9.301
NIAID AIDSno	192393	9.301
NIAID AIDSno	192394	9.301
NIAID AIDSno	192395	9.301
NIAID AIDSno	192396	9.301
NIAID AIDSno	192397	9.301
NIAID AIDSno	192398	9.301
NIAID AIDSno	192399	9.301
NIAID AIDSno	192400	9.301
NIAID AIDSno	192401	9.301
NIAID AIDSno	192402	9.301
NIAID AIDSno	192403	9.301
NIAID AIDSno	192404	9.301
NIAID AIDSno	192405	9.301
NIAID AIDSno	192406	9.301
NIAID AIDSno	192407	9.301
NIAID AIDSno	192408	9.301
NIAID AIDSno	192409	9.301
NIAID AIDSno	192410	9.301
NIAID AIDSno	192411	9.301
NIAID AIDSno	192412	9.301
NIAID AIDSno	192413	9.301
NIAID AIDSno	192414	9.301

NIAID AIDSno	192416	9.301
NIAID AIDSno	192417	9.301
NIAID AIDSno	192418	9.301
NIAID AIDSno	192420	9.301
NIAID AIDSno	192421	9.301
NIAID AIDSno	192422	9.301
NIAID AIDSno	192423	9.301
NIAID AIDSno	192424	9.301
NIAID AIDSno	192425	9.301
NIAID AIDSno	192429	9.301
NIAID AIDSno	192431	9.000
NIAID AIDSno	192434	9.301
NIAID AIDSno	192435	9.301
NIAID AIDSno	192436	9.301
NIAID AIDSno	192493	8.903
NIAID AIDSno	192495	8.891
NIAID AIDSno	192497	7.409
NIAID AIDSno	192498	7.168
NIAID AIDSno	192499	10.149
NIAID AIDSno	192500	9.796
NIAID AIDSno	192501	9.409
NIAID AIDSno	192502	9.921
NIAID AIDSno	192503	8.548
NIAID AIDSno	192504	9.620
NIAID AIDSno	192505	10.222
NIAID AIDSno	192506	9.824
NIAID AIDSno	192507	9.959
NIAID AIDSno	192509	8.155
NIAID AIDSno	192510	6.730
NIAID AIDSno	192512	10.523
NIAID AIDSno	196516	7.119
NIAID AIDSno	209030	4.409
NIAID AIDSno	209031	3.824
NIAID AIDSno	209032	3.244
NIAID AIDSno	209852	4.089
NIAID AIDSno	210543	4.155
NIAID AIDSno	210545	3.821
NIAID AIDSno	210546	3.979
NIAID AIDSno	210547	3.650
NIAID AIDSno	210549	3.532
NIAID AIDSno	210550	3.613
NIAID AIDSno	210551	5.398
NIAID AIDSno	210554	4.959
NIAID AIDSno	210555	5.523
NIAID AIDSno	210556	4.770

NIAID AIDSno	210557	4.328
NIAID AIDSno	210558	4.569
NIAID AIDSno	210559	3.971
NIAID AIDSno	210560	5.301
NIAID AIDSno	210561	6.000
NIAID AIDSno	210562	4.509
NIAID AIDSno	210566	4.672
NIAID AIDSno	211886	9.301
NIAID AIDSno	211887	9.301
NIAID AIDSno	211888	9.301
NIAID AIDSno	211889	9.301
NIAID AIDSno	211890	9.301
NIAID AIDSno	211891	9.301
NIAID AIDSno	211892	9.301
NIAID AIDSno	211893	9.301
NIAID AIDSno	212164	5.301
NIAID AIDSno	212165	4.347
NIAID AIDSno	212166	3.658
NIAID AIDSno	212167	4.268
NIAID AIDSno	212169	5.097
NIAID AIDSno	212170	5.398
NIAID AIDSno	212171	4.921
NIAID AIDSno	212172	4.222
NIAID AIDSno	212173	4.260
NIAID AIDSno	212174	5.046
NIAID AIDSno	212176	4.523
NIAID AIDSno	212177	3.237
NIAID AIDSno	212178	4.398
NIAID AIDSno	212179	4.602
NIAID AIDSno	212180	3.432
NIAID AIDSno	212181	4.155
NIAID AIDSno	212182	4.180
NIAID AIDSno	212183	4.787
NIAID AIDSno	212258	5.013
NIAID AIDSno	212259	4.775
NIAID AIDSno	212260	6.485
NIAID AIDSno	212261	6.607
NIAID AIDSno	212262	6.442
NIAID AIDSno	212263	5.432
NIAID AIDSno	212264	4.699
NIAID AIDSno	214742	8.000
NIAID AIDSno	214743	8.602
NIAID AIDSno	214745	8.301
NIAID AIDSno	214746	8.602
NIAID AIDSno	214747	9.000

NIAID AIDSno	214748	8.000
NIAID AIDSno	214749	8.301
NIAID AIDSno	214750	8.301
NIAID AIDSno	214752	8.301
NIAID AIDSno	214753	8.000
NIAID AIDSno	214755	9.000
NIAID AIDSno	214757	8.000
NIAID AIDSno	214758	8.000
NIAID AIDSno	214760	8.301
NIAID AIDSno	214764	8.000
NIAID AIDSno	214765	8.301
NIAID AIDSno	214767	9.000
NIAID AIDSno	214768	8.301
NIAID AIDSno	214769	9.000
NIAID AIDSno	214770	9.000
NIAID AIDSno	214771	7.000
NIAID AIDSno	214772	8.000
NIAID AIDSno	215842	9.301
NIAID AIDSno	215843	9.301
NIAID AIDSno	215844	9.301
NIAID AIDSno	215845	9.301
NIAID AIDSno	215846	9.301
NIAID AIDSno	215847	9.301
NIAID AIDSno	215848	9.301
NIAID AIDSno	215849	9.301
NIAID AIDSno	215850	9.301
NIAID AIDSno	215851	8.620
NIAID AIDSno	215852	9.000
NIAID AIDSno	215853	7.921
NIAID AIDSno	215854	9.301
NIAID AIDSno	215857	9.301
NIAID AIDSno	215858	9.301
NIAID AIDSno	215859	9.301
NIAID AIDSno	215860	9.301
NIAID AIDSno	215861	9.301
NIAID AIDSno	215863	7.921
NIAID AIDSno	215864	9.301
NIAID AIDSno	215865	9.301
NIAID AIDSno	215866	9.301
NIAID AIDSno	215867	9.301
NIAID AIDSno	215868	9.301
NIAID AIDSno	215869	9.301
NIAID AIDSno	215870	9.301
NIAID AIDSno	215871	9.301
NIAID AIDSno	215872	9.301

NIAID AIDSno	215876	9.301
NIAID AIDSno	215877	9.301
NIAID AIDSno	215878	9.301
NIAID AIDSno	215879	9.301
NIAID AIDSno	215882	5.620
NIAID AIDSno	215883	6.339
NIAID AIDSno	215884	5.222
NIAID AIDSno	215885	5.796
NIAID AIDSno	215886	5.509
NIAID AIDSno	215887	6.155
NIAID AIDSno	215888	5.699
NIAID AIDSno	215889	5.678
NIAID AIDSno	215890	5.745
NIAID AIDSno	215891	5.432
NIAID AIDSno	215892	5.194
NIAID AIDSno	215893	5.585
NIAID AIDSno	215894	5.000
NIAID AIDSno	215895	5.432
NIAID AIDSno	215896	5.268
NIAID AIDSno	215897	5.076
NIAID AIDSno	215898	4.585
NIAID AIDSno	215899	5.000
NIAID AIDSno	215900	4.569
NIAID AIDSno	215901	4.699
NIAID AIDSno	215902	5.602
NIAID AIDSno	215903	5.824
NIAID AIDSno	215904	6.222
NIAID AIDSno	215905	6.481
NIAID AIDSno	215906	5.377
NIAID AIDSno	215907	5.222
NIAID AIDSno	215908	5.284
NIAID AIDSno	215909	5.301
NIAID AIDSno	215910	5.523
NIAID AIDSno	215911	5.367
NIAID AIDSno	215912	5.699
NIAID AIDSno	215913	5.301
NIAID AIDSno	215914	5.921
NIAID AIDSno	215915	6.097
NIAID AIDSno	215916	6.481
NIAID AIDSno	215917	6.699
NIAID AIDSno	215925	9.959
NIAID AIDSno	215926	8.879
NIAID AIDSno	215927	9.337
NIAID AIDSno	215928	9.796
NIAID AIDSno	215929	9.796

NIAID AIDSno	215930	9.745
NIAID AIDSno	215931	10.301
NIAID AIDSno	215932	9.824
NIAID AIDSno	215933	10.301
NIAID AIDSno	215934	10.000
NIAID AIDSno	215935	10.301
NIAID AIDSno	215936	10.398
NIAID AIDSno	215937	10.000
NIAID AIDSno	215938	10.301
NIAID AIDSno	215939	10.222
NIAID AIDSno	215940	10.523
NIAID AIDSno	215941	10.699
NIAID AIDSno	215942	10.699
NIAID AIDSno	215944	8.038
NIAID AIDSno	215946	6.452
NIAID AIDSno	215948	4.962
NIAID AIDSno	215950	4.884
NIAID AIDSno	215952	4.403
NIAID AIDSno	215954	4.097
NIAID AIDSno	219939	10.620
NIAID AIDSno	219940	10.569
NIAID AIDSno	219941	10.796
NIAID AIDSno	219942	10.699
NIAID AIDSno	219944	10.824
NIAID AIDSno	219947	10.796
NIAID AIDSno	219948	10.824
NIAID AIDSno	219949	10.824
NIAID AIDSno	219953	10.824
NIAID AIDSno	219954	10.824
NIAID AIDSno	219955	10.824
NIAID AIDSno	219956	10.721
NIAID AIDSno	219957	9.620
NIAID AIDSno	219958	9.398
NIAID AIDSno	219959	9.495
NIAID AIDSno	219960	9.319
NIAID AIDSno	220021	5.961
NIAID AIDSno	220022	5.119
NIAID AIDSno	220023	5.509
NIAID AIDSno	220024	5.785
NIAID AIDSno	220025	5.699
NIAID AIDSno	220026	5.514
NIAID AIDSno	220027	5.444
NIAID AIDSno	220028	5.469
NIAID AIDSno	220029	5.215
NIAID AIDSno	220030	4.491

NIAID AIDSno	220031	3.903
NIAID AIDSno	220037	5.604
NIAID AIDSno	220038	5.803
NIAID AIDSno	220039	5.569
NIAID AIDSno	220040	5.538
NIAID AIDSno	220041	5.337
NIAID AIDSno	220042	5.305
NIAID AIDSno	220043	5.143
NIAID AIDSno	220044	4.979
NIAID AIDSno	220045	4.893
NIAID AIDSno	220048	6.252
NIAID AIDSno	220049	6.060
NIAID AIDSno	220050	5.883
NIAID AIDSno	220051	5.928
NIAID AIDSno	220052	5.996
NIAID AIDSno	220053	5.721
NIAID AIDSno	220054	5.706
NIAID AIDSno	220055	5.564
NIAID AIDSno	220056	5.260
NIAID AIDSno	220061	6.319
NIAID AIDSno	220062	6.208
NIAID AIDSno	220063	5.703
NIAID AIDSno	220064	5.833
NIAID AIDSno	220065	5.538
NIAID AIDSno	220066	5.456
NIAID AIDSno	220067	5.613
NIAID AIDSno	220068	5.456
NIAID AIDSno	220069	5.444
NIAID AIDSno	220070	5.390
NIAID AIDSno	220071	5.357
NIAID AIDSno	220072	5.276
NIAID AIDSno	220073	4.925
NIAID AIDSno	220074	4.900
NIAID AIDSno	220077	5.357
NIAID AIDSno	220078	5.432
NIAID AIDSno	220079	5.538
NIAID AIDSno	220080	5.921
NIAID AIDSno	220081	5.921
NIAID AIDSno	220082	5.585
NIAID AIDSno	220083	5.284
NIAID AIDSno	220084	5.252
NIAID AIDSno	220085	4.921
NIAID AIDSno	220086	4.523
NIAID AIDSno	222535	5.602
NIAID AIDSno	222536	4.244

NIAID AIDSno	222598	7.301
NIAID AIDSno	222602	7.301
NIAID AIDSno	222604	7.301
NIAID AIDSno	222605	7.301
NIAID AIDSno	222606	7.301
NIAID AIDSno	222608	7.301
NIAID AIDSno	222612	7.301
NIAID AIDSno	222613	7.301
NIAID AIDSno	222614	7.301
NIAID AIDSno	222615	7.301
NIAID AIDSno	222616	7.301
NIAID AIDSno	223868	8.000
NIAID AIDSno	224620	5.398
NIAID AIDSno	224621	5.854
NIAID AIDSno	224622	5.367
NIAID AIDSno	224623	5.770
NIAID AIDSno	224624	5.959
NIAID AIDSno	224625	5.745
NIAID AIDSno	224626	5.553
NIAID AIDSno	224628	5.854
NIAID AIDSno	224630	5.276
NIAID AIDSno	224631	5.292
NIAID AIDSno	224632	5.553
NIAID AIDSno	224634	5.959
NIAID AIDSno	224635	6.000
NIAID AIDSno	225362	8.301
NIAID AIDSno	225376	8.678
NIAID AIDSno	228483	3.367
NIAID AIDSno	228484	3.870
NIAID AIDSno	228485	3.561
NIAID AIDSno	228486	4.357
NIAID AIDSno	228487	4.114
NIAID AIDSno	228488	4.638
NIAID AIDSno	228489	4.071
NIAID AIDSno	228490	5.301
NIAID AIDSno	228492	3.670
NIAID AIDSno	228493	3.688
NIAID AIDSno	228494	3.883
NIAID AIDSno	231456	8.444
NIAID AIDSno	231481	6.509
NIAID AIDSno	231482	8.155
NIAID AIDSno	231483	8.398
NIAID AIDSno	231484	7.796
NIAID AIDSno	231485	8.523
NIAID AIDSno	231486	8.523

NIAID AIDSno	231487	8.699
NIAID AIDSno	231488	8.398
NIAID AIDSno	231489	8.699
NIAID AIDSno	231490	7.770
NIAID AIDSno	231491	7.854
NIAID AIDSno	231493	7.678
NIAID AIDSno	231494	7.432
NIAID AIDSno	231495	8.398
NIAID AIDSno	231496	8.398
NIAID AIDSno	231497	8.699
NIAID AIDSno	231498	8.301
NIAID AIDSno	231499	7.796
NIAID AIDSno	231500	7.553
NIAID AIDSno	231501	8.155
NIAID AIDSno	231502	8.155
NIAID AIDSno	231503	8.398
NIAID AIDSno	231504	8.398
NIAID AIDSno	231505	8.301
NIAID AIDSno	231506	8.155
NIAID AIDSno	231507	8.398
NIAID AIDSno	231508	8.301
NIAID AIDSno	231509	8.301
NIAID AIDSno	231510	8.301
NIAID AIDSno	231511	8.398
NIAID AIDSno	231512	8.523
NIAID AIDSno	231513	8.097
NIAID AIDSno	231514	8.301
NIAID AIDSno	257754	6.000
NIAID AIDSno	257756	5.959
NIAID AIDSno	257757	6.024
NIAID AIDSno	257758	6.009
NIAID AIDSno	257759	5.947
NIAID AIDSno	257760	5.796
NIAID AIDSno	257761	5.678
NIAID AIDSno	257762	5.114
NIAID AIDSno	257769	6.503
NIAID AIDSno	257770	6.299
NIAID AIDSno	257771	6.264
NIAID AIDSno	257772	6.264
NIAID AIDSno	257773	6.200
NIAID AIDSno	257774	6.229
NIAID AIDSno	257775	6.131
NIAID AIDSno	257776	6.059
NIAID AIDSno	257777	5.886
NIAID AIDSno	257778	5.854

NIAID AIDSno	257779	5.772
NIAID AIDSno	257780	5.503
NIAID AIDSno	257781	5.449
NIAID AIDSno	257786	6.559
NIAID AIDSno	257787	6.583
NIAID AIDSno	257788	6.588
NIAID AIDSno	257789	6.545
NIAID AIDSno	257790	6.503
NIAID AIDSno	257791	6.527
NIAID AIDSno	257792	6.456
NIAID AIDSno	257793	6.478
NIAID AIDSno	257794	6.371
NIAID AIDSno	257795	6.258
NIAID AIDSno	257796	6.322
NIAID AIDSno	257797	6.111
NIAID AIDSno	257798	5.921
NIAID AIDSno	257799	5.678
NIAID AIDSno	257800	6.197
NIAID AIDSno	257801	6.041
NIAID AIDSno	257802	6.203
NIAID AIDSno	257803	6.101
NIAID AIDSno	257804	6.177
NIAID AIDSno	257805	6.061
NIAID AIDSno	284315	4.328
NIAID AIDSno	284382	7.509
NIAID AIDSno	284383	6.775
NIAID AIDSno	284384	5.924
NIAID AIDSno	284385	6.506
NIAID AIDSno	284386	5.000
NIAID AIDSno	284387	7.409
NIAID AIDSno	284388	6.830
NIAID AIDSno	284389	6.595
NIAID AIDSno	284390	6.845
NIAID AIDSno	284391	6.996
NIAID AIDSno	284392	7.292
NIAID AIDSno	284393	7.796
NIAID AIDSno	284394	8.301
NIAID AIDSno	284395	6.185
NIAID AIDSno	284396	7.347
NIAID AIDSno	284397	7.796
NIAID AIDSno	293250	7.444
NIAID AIDSno	293251	7.886
NIAID AIDSno	293252	8.155
NIAID AIDSno	293253	6.959
NIAID AIDSno	293254	7.032

NIAID AIDSno	293255	6.886
NIAID AIDSno	293256	7.398
NIAID AIDSno	293257	6.745
NIAID AIDSno	293258	7.745
NIAID AIDSno	293259	7.071
NIAID AIDSno	293260	6.921
NIAID AIDSno	293261	6.420
NIAID AIDSno	293262	8.252
NIAID AIDSno	293263	7.824
NIAID AIDSno	293264	7.921
NIAID AIDSno	293265	7.745
NIAID AIDSno	293266	7.602
NIAID AIDSno	293267	7.495
NIAID AIDSno	293268	7.481
NIAID AIDSno	293269	7.481
NIAID AIDSno	293270	7.081
NIAID AIDSno	293271	7.041
NIAID AIDSno	293272	8.097
NIAID AIDSno	293273	8.076
NIAID AIDSno	293274	7.921
NIAID AIDSno	293275	7.824
NIAID AIDSno	293276	7.796
NIAID AIDSno	293277	7.523
NIAID AIDSno	293278	8.201
NIAID AIDSno	293279	8.180
NIAID AIDSno	293280	8.149
NIAID AIDSno	293281	8.114
NIAID AIDSno	293282	8.066
NIAID AIDSno	293283	8.027
NIAID AIDSno	293284	7.854
NIAID AIDSno	293285	7.167
NIAID AIDSno	293286	6.921
NIAID AIDSno	293287	6.854
NIAID AIDSno	293288	6.824
NIAID AIDSno	293289	6.796
NIAID AIDSno	293290	6.585
NIAID AIDSno	293291	6.222
NIAID AIDSno	334787	4.523
NIAID AIDSno	334788	5.301
NIAID AIDSno	339020	6.921
NIAID AIDSno	339021	10.000
NIAID AIDSno	339022	10.000
NIAID AIDSno	339023	10.000
NIAID AIDSno	339024	10.000
NIAID AIDSno	339025	10.000

NIAID AIDSno	339026	10.000
NIAID AIDSno	339027	10.000
NIAID AIDSno	339028	10.000
NIAID AIDSno	339029	10.000
NIAID AIDSno	339030	10.000
NIAID AIDSno	342599	9.301
NIAID AIDSno	342600	9.301
NIAID AIDSno	342601	8.102
NIAID AIDSno	342602	8.215
NIAID AIDSno	342603	9.301
NIAID AIDSno	342604	8.420
NIAID AIDSno	342605	8.678
NIAID AIDSno	342606	9.301
NIAID AIDSno	342607	9.301
NIAID AIDSno	342608	9.301
NIAID AIDSno	342609	9.301
NIAID AIDSno	342610	9.301
NIAID AIDSno	342611	9.301
NIAID AIDSno	342612	9.301
NIAID AIDSno	342613	9.301
NIAID AIDSno	342614	9.301
NIAID AIDSno	342615	8.921
NIAID AIDSno	342616	9.301
NIAID AIDSno	342617	9.301
NIAID AIDSno	342618	9.301
NIAID AIDSno	342619	9.301
NIAID AIDSno	342620	9.301
NIAID AIDSno	342621	9.301
NIAID AIDSno	342622	9.301
NIAID AIDSno	342623	9.301
NIAID AIDSno	342624	9.301
NIAID AIDSno	342625	9.301
NIAID AIDSno	342626	9.301
NIAID AIDSno	345118	4.728
NIAID AIDSno	345119	5.252
NIAID AIDSno	345145	8.620
NIAID AIDSno	345146	9.357
NIAID AIDSno	345147	7.688
NIAID AIDSno	345149	8.602
NIAID AIDSno	345150	7.018
NIAID AIDSno	345479	4.810
NIAID AIDSno	345484	4.699
NIAID AIDSno	345487	5.000
NIAID AIDSno	345492	7.412
NIAID AIDSno	345519	9.301

NIAID AIDSno	345524	9.301
NIAID AIDSno	345527	9.301
NIAID AIDSno	345528	9.301
NIAID AIDSno	345530	9.301
NIAID AIDSno	345532	9.301
NIAID AIDSno	345533	9.301
NIAID AIDSno	345536	9.301
NIAID AIDSno	345537	9.301
NIAID AIDSno	345540	9.301
NIAID AIDSno	345543	9.301
NIAID AIDSno	346004	7.071
NIAID AIDSno	346008	6.854
NIAID AIDSno	346009	5.000
NIAID AIDSno	346010	5.523
NIAID AIDSno	346011	5.252
NIAID AIDSno	346012	6.276
NIAID AIDSno	346016	5.377
NIAID AIDSno	346018	10.699
NIAID AIDSno	346019	10.398
NIAID AIDSno	346020	10.523
NIAID AIDSno	346021	10.301
NIAID AIDSno	346022	10.155
NIAID AIDSno	346023	10.301
NIAID AIDSno	346024	10.699
NIAID AIDSno	346025	10.699
NIAID AIDSno	346026	10.699
NIAID AIDSno	346027	9.959
NIAID AIDSno	346028	10.097
NIAID AIDSno	346029	10.523
NIAID AIDSno	346030	10.398
NIAID AIDSno	346031	10.523
NIAID AIDSno	352976	7.398
NIAID AIDSno	352977	6.886
NIAID AIDSno	352981	7.886
NIAID AIDSno	352982	9.398
NIAID AIDSno	354157	6.292
NIAID AIDSno	354158	5.991
NIAID AIDSno	354159	6.337
NIAID AIDSno	354160	6.161
NIAID AIDSno	354161	6.194
NIAID AIDSno	354162	6.292
NIAID AIDSno	354163	6.174
NIAID AIDSno	354164	6.237
NIAID AIDSno	354165	6.046
NIAID AIDSno	354166	6.260

NIAID AIDSno	354167	6.276
NIAID AIDSno	354168	6.509
NIAID AIDSno	354169	6.076
NIAID AIDSno	354170	6.060
NIAID AIDSno	354171	5.873
NIAID AIDSno	354172	6.201
NIAID AIDSno	354173	6.114
NIAID AIDSno	354174	6.276
NIAID AIDSno	354175	5.932
NIAID AIDSno	354176	6.013
NIAID AIDSno	354177	5.777
NIAID AIDSno	354178	6.114
NIAID AIDSno	354179	5.907
NIAID AIDSno	354181	6.237
NIAID AIDSno	354182	6.149
NIAID AIDSno	354183	6.319
NIAID AIDSno	354184	6.237
NIAID AIDSno	354185	6.187
NIAID AIDSno	354186	6.432
NIAID AIDSno	354187	6.328
NIAID AIDSno	354188	6.481
NIAID AIDSno	354189	6.187
NIAID AIDSno	354190	6.268
NIAID AIDSno	354191	6.292
NIAID AIDSno	354192	6.409
NIAID AIDSno	354193	6.319
NIAID AIDSno	354194	6.495
NIAID AIDSno	354195	6.237
NIAID AIDSno	354196	6.387
NIAID AIDSno	354197	6.276
NIAID AIDSno	354198	6.420
NIAID AIDSno	354199	6.328
NIAID AIDSno	354200	6.276
NIAID AIDSno	354201	6.237
NIAID AIDSno	354202	6.301
NIAID AIDSno	354203	6.244
NIAID AIDSno	354204	6.432
NIAID AIDSno	367837	5.886
NIAID AIDSno	367838	6.155
NIAID AIDSno	367839	5.854
NIAID AIDSno	367840	5.699
NIAID AIDSno	367841	5.602
NIAID AIDSno	367842	5.658
NIAID AIDSno	367843	5.398
NIAID AIDSno	367844	5.456

NIAID AIDSno	367845	5.886
NIAID AIDSno	367846	5.854
NIAID AIDSno	414354	4.021
NIAID AIDSno	414355	4.979
NIAID AIDSno	414356	4.125
NIAID AIDSno	414357	4.714
NIAID AIDSno	414358	4.257
NIAID AIDSno	414359	4.804
NIAID AIDSno	415342	9.215
NIAID AIDSno	415343	10.222
NIAID AIDSno	415344	8.018
NIAID AIDSno	415345	7.123
NIAID AIDSno	415346	7.072
NIAID AIDSno	439586	7.301
NIAID AIDSno	439587	7.301
NIAID AIDSno	439588	7.301
NIAID AIDSno	439589	7.301
NIAID AIDSno	439590	7.301
NIAID AIDSno	439591	7.301
NIAID AIDSno	439592	7.301
NIAID AIDSno	439593	7.301
NIAID AIDSno	439594	7.301
NIAID AIDSno	439595	7.301
NIAID AIDSno	439596	7.301
NIAID AIDSno	439597	7.301
NIAID AIDSno	439598	7.301
NIAID AIDSno	439599	7.301
NIAID AIDSno	439600	7.301
NIAID AIDSno	439601	7.301
NIAID AIDSno	439602	7.301
NIAID AIDSno	439603	7.301
NIAID AIDSno	439604	7.301
NIAID AIDSno	439605	7.301
NIAID AIDSno	439606	7.301
NIAID AIDSno	439607	7.301
NIAID AIDSno	439608	7.301
NIAID AIDSno	439609	7.301
NIAID AIDSno	439610	7.301
NIAID AIDSno	439611	7.301
NIAID AIDSno	439612	7.301
NIAID AIDSno	439613	7.301
NIAID AIDSno	439614	7.301
NIAID AIDSno	439615	7.301
NIAID AIDSno	439616	7.301
NIAID AIDSno	439617	7.301

NIAID AIDSno	439618	7.301
NIAID AIDSno	439619	7.301
NIAID AIDSno	439620	7.301
NIAID AIDSno	439621	7.301
NIAID AIDSno	439622	7.301
NIAID AIDSno	470558	5.301
NIAID AIDSno	470562	5.301
NIAID AIDSno	470572	5.301
NIAID AIDSno	471190	5.824
NIAID AIDSno	471191	5.398
NIAID AIDSno	471192	4.638
NIAID AIDSno	471193	4.602
NIAID AIDSno	471195	4.509
NIAID AIDSno	471196	4.319
NIAID AIDSno	471197	4.000
NIAID AIDSno	471199	3.959
NIAID AIDSno	471200	5.398
NIAID AIDSno	471201	4.699
NIAID AIDSno	471202	4.398
NIAID AIDSno	471203	4.167
NIAID AIDSno	471777	4.447
NIAID AIDSno	474660	8.523
NIAID AIDSno	474661	6.452
NIAID AIDSno	474662	6.559
NIAID AIDSno	474663	6.203
NIAID AIDSno	474664	6.259
NIAID AIDSno	474665	8.796
NIAID AIDSno	474666	8.161
NIAID AIDSno	474667	7.509
NIAID AIDSno	474668	7.745
NIAID AIDSno	474669	7.155
NIAID AIDSno	474670	8.081
NIAID AIDSno	474671	8.000
NIAID AIDSno	474672	7.770
NIAID AIDSno	474673	8.180
NIAID AIDSno	474674	7.456
NIAID AIDSno	474675	7.824
NIAID AIDSno	474676	7.638
NIAID AIDSno	474677	7.745
NIAID AIDSno	474678	7.509
NIAID AIDSno	474679	8.469
NIAID AIDSno	474680	8.481
NIAID AIDSno	474681	8.796
NIAID AIDSno	474682	8.301
NIAID AIDSno	474683	8.137

NIAID AIDSno	474684	8.854
NIAID AIDSno	474685	8.886
NIAID AIDSno	474686	7.824
NIAID AIDSno	474687	8.959
NIAID AIDSno	474688	7.745
NIAID AIDSno	474689	8.638
NIAID AIDSno	474690	8.215
NIAID AIDSno	474691	7.638
NIAID AIDSno	474692	7.229
NIAID AIDSno	474693	7.032
NIAID AIDSno	474694	7.602
NIAID AIDSno	474695	8.553
NIAID AIDSno	474696	7.886
NIAID AIDSno	474697	7.377
NIAID AIDSno	474698	8.237
NIAID AIDSno	474699	8.046
NIAID AIDSno	474700	8.409
NIAID AIDSno	474701	9.222
NIAID AIDSno	474702	8.959
NIAID AIDSno	474703	8.854
NIAID AIDSno	474704	8.046
NIAID AIDSno	474705	8.409
NIAID AIDSno	474707	8.585
NIAID AIDSno	474708	8.678
NIAID AIDSno	474709	8.745
NIAID AIDSno	474710	8.060
NIAID AIDSno	474711	8.469
NIAID AIDSno	474712	8.444
NIAID AIDSno	474714	7.947
NIAID AIDSno	474715	8.004
NIAID AIDSno	474716	8.180
NIAID AIDSno	474717	7.824
NIAID AIDSno	474718	8.131
NIAID AIDSno	474719	8.013
NIAID AIDSno	474720	8.180
NIAID AIDSno	474721	8.301
NIAID AIDSno	474722	8.180
NIAID AIDSno	474723	6.963
NIAID AIDSno	474724	6.520
NIAID AIDSno	474726	6.886
NIAID AIDSno	474809	4.000
NIAID AIDSno	475077	9.745
NIAID AIDSno	475078	9.585
NIAID AIDSno	475079	9.000
NIAID AIDSno	475080	9.000

NIAID AIDSno	475081	9.000
NIAID AIDSno	475082	9.000
NIAID AIDSno	475083	10.046
NIAID AIDSno	475084	10.081
NIAID AIDSno	475085	9.000
NIAID AIDSno	475086	9.000
NIAID AIDSno	475087	10.066
NIAID AIDSno	475088	10.046
NIAID AIDSno	477065	4.398
NIAID AIDSno	477877	8.784
NIAID AIDSno	477878	7.854
NIAID AIDSno	477879	7.377
NIAID AIDSno	477880	9.854
NIAID AIDSno	477881	9.770
NIAID AIDSno	477882	10.000
NIAID AIDSno	477883	9.959
NIAID AIDSno	477884	9.849
NIAID AIDSno	477885	10.000
NIAID AIDSno	479606	9.222
NIAID AIDSno	479607	7.428
NIAID AIDSno	479608	8.602
NIAID AIDSno	479609	4.000
NIAID AIDSno	479610	5.761
NIAID AIDSno	479633	8.310
NIAID AIDSno	479637	8.469
NIAID AIDSno	479679	5.456
NIAID AIDSno	479680	5.301
NIAID AIDSno	479681	5.602
NIAID AIDSno	479682	5.347
NIAID AIDSno	479683	4.740
NIAID AIDSno	479684	4.521
NIAID AIDSno	481004	3.824
NIAID AIDSno	481005	4.060
NIAID AIDSno	481006	4.301
NIAID AIDSno	481007	4.827
NIAID AIDSno	481008	5.398
NIAID AIDSno	481009	4.710
NIAID AIDSno	481010	5.022
NIAID AIDSno	481011	5.602
NIAID AIDSno	481012	6.171
NIAID AIDSno	481013	5.602
NIAID AIDSno	481014	5.187
NIAID AIDSno	481015	5.538
NIAID AIDSno	481016	5.921
NIAID AIDSno	481017	7.194

NIAID AIDSno	481453	4.405
NIAID AIDSno	481455	4.374
NIAID AIDSno	481461	4.616
NIAID AIDSno	481462	4.545
NIAID AIDSno	481463	4.924
NIAID AIDSno	481464	4.642
NIAID AIDSno	481465	5.000
NIAID AIDSno	481466	4.524
NIAID AIDSno	481470	5.569
NIAID AIDSno	481471	5.187
NIAID AIDSno	481473	5.409
NIAID AIDSno	481474	5.569
NIAID AIDSno	481475	5.268
NIAID AIDSno	481477	4.963
NIAID AIDSno	482087	6.671
NIAID AIDSno	482092	8.420
NIAID AIDSno	482332	7.703
NIAID AIDSno	482333	6.179
NIAID AIDSno	482590	3.265
NIAID AIDSno	482592	4.352
NIAID AIDSno	482597	3.467
NIAID AIDSno	482608	3.772
NIAID AIDSno	482753	3.264
NIAID AIDSno	482754	3.690
NIAID AIDSno	485746	7.699
NIAID AIDSno	485748	6.420
NIAID AIDSno	485749	4.859
NIAID AIDSno	485750	4.447
NIAID AIDSno	485752	5.452
NIAID AIDSno	485755	4.200
NIAID AIDSno	485756	4.967
NIAID AIDSno	485758	4.302
NIAID AIDSno	485846	4.830
NIAID AIDSno	485847	5.427
NIAID AIDSno	485850	7.301
NIAID AIDSno	485851	5.312
NIAID AIDSno	485852	4.479
NIAID AIDSno	485853	4.525
NIAID AIDSno	485854	4.705
NIAID AIDSno	485856	5.143
NIAID AIDSno	485857	4.983
NIAID AIDSno	485858	4.372
NIAID AIDSno	485860	4.431
NIAID AIDSno	485861	5.750
NIAID AIDSno	486928	5.770

NIAID AIDSno	486948	4.192
NIAID AIDSno	486950	3.996
NIAID AIDSno	487002	3.834
NIAID AIDSno	487012	4.056
NIAID AIDSno	487020	4.310
NIAID AIDSno	489801	6.786
NIAID AIDSno	489802	4.740
NIAID AIDSno	489803	3.661
NIAID AIDSno	489804	4.230
NIAID AIDSno	489805	5.496
NIAID AIDSno	489806	4.910
NIAID AIDSno	489807	3.804
NIAID AIDSno	489808	4.028
NIAID AIDSno	489809	3.680
NIAID AIDSno	489810	5.620
NIAID AIDSno	494775	4.496
NIAID AIDSno	494777	4.627
NIAID AIDSno	494779	4.473
NIAID AIDSno	494781	4.388
NIAID AIDSno	494783	4.836
NIAID AIDSno	494784	4.726
NIAID AIDSno	494785	4.609
NIAID AIDSno	494786	4.801
NIAID AIDSno	494787	4.595
NIAID AIDSno	494788	4.617
NIAID AIDSno	494789	4.368
NIAID AIDSno	494790	4.963
NIAID AIDSno	494791	4.804
NIAID AIDSno	494792	4.595
NIAID AIDSno	494793	5.244
NIAID AIDSno	494794	4.754
NIAID AIDSno	494795	5.409
NIAID AIDSno	494796	4.055
NIAID AIDSno	494798	4.498
NIAID AIDSno	494800	4.733
NIAID AIDSno	494802	4.609
NIAID AIDSno	494803	4.544
NIAID AIDSno	494805	4.304
NIAID AIDSno	495358	5.585
NIAID AIDSno	495363	5.237
NIAID AIDSno	495373	4.750
NIAID AIDSno	495376	4.991
NIAID AIDSno	495377	4.604
NIAID AIDSno	495460	5.201
NIAID AIDSno	497302	3.996

NIAID AIDSno	497478	4.410
NIAID AIDSno	497488	5.252
NIAID AIDSno	500711	5.398
NIAID AIDSno	500712	8.538
NIAID AIDSno	500713	8.222
NIAID AIDSno	500714	7.824
NIAID AIDSno	500715	5.824
NIAID AIDSno	500716	7.678
NIAID AIDSno	500717	8.921
NIAID AIDSno	502138	4.143
NIAID AIDSno	504578	4.764
NIAID AIDSno	505153	10.824
NIAID AIDSno	505155	9.406
NIAID AIDSno	505156	10.796
NIAID AIDSno	505157	9.987
NIAID AIDSno	505158	8.495
NIAID AIDSno	505159	10.886
NIAID AIDSno	506445	7.495
NIAID AIDSno	506446	7.796
NIAID AIDSno	506966	8.097
NIAID AIDSno	506967	7.854
NIAID AIDSno	506968	7.620
NIAID AIDSno	506969	7.222
NIAID AIDSno	506970	7.699
NIAID AIDSno	506971	7.347
NIAID AIDSno	506972	7.523
NIAID AIDSno	506973	7.319
NIAID AIDSno	506974	7.602
NIAID AIDSno	506975	7.252
NIAID AIDSno	506978	7.921
NIAID AIDSno	506980	7.569
NIAID AIDSno	506981	7.796
NIAID AIDSno	506982	7.244
NIAID AIDSno	506983	7.222
NIAID AIDSno	506984	7.721
NIAID AIDSno	506985	7.658
NIAID AIDSno	506986	7.699
NIAID AIDSno	506987	8.301
NIAID AIDSno	506988	7.161
NIAID AIDSno	506989	7.398
NIAID AIDSno	506990	7.347
NIAID AIDSno	510591	8.699
NIAID AIDSno	510592	8.244
NIAID AIDSno	510593	8.620
NIAID AIDSno	510594	9.398

NIAID AIDSno	511118	8.292
NIAID AIDSno	511123	9.333
NIAID AIDSno	511124	9.147
NIAID AIDSno	511153	9.186
NIAID AIDSno	511158	8.969
NIAID AIDSno	511160	8.967
NIAID AIDSno	511161	9.327
NIAID AIDSno	511165	9.429
NIAID AIDSno	511167	9.474
NIAID AIDSno	511172	9.209
NIAID AIDSno	511173	8.246
NIAID AIDSno	511175	9.078
NIAID AIDSno	511176	9.298
NIAID AIDSno	511178	9.166
NIAID AIDSno	511180	9.036
NIAID AIDSno	511181	9.222
NIAID AIDSno	511182	9.538
NIAID AIDSno	511199	9.002
NIAID AIDSno	511200	8.694
NIAID AIDSno	511201	9.223
NIAID AIDSno	511202	8.437
NIAID AIDSno	511204	9.046
NIAID AIDSno	511206	8.593
NIAID AIDSno	511207	8.419
NIAID AIDSno	511208	8.668
NIAID AIDSno	511210	8.891
NIAID AIDSno	511211	8.869
NIAID AIDSno	511212	8.385
NIAID AIDSno	511213	9.028
NIAID AIDSno	511215	8.283
NIAID AIDSno	511216	8.541
NIAID AIDSno	511217	8.480
NIAID AIDSno	511328	9.367
NIAID AIDSno	511329	8.301
NIAID AIDSno	511330	9.292
NIAID AIDSno	511331	9.284
NIAID AIDSno	511332	9.699
NIAID AIDSno	511333	10.523
NIAID AIDSno	511334	9.879
NIAID AIDSno	511335	10.553
NIAID AIDSno	511336	10.658
NIAID AIDSno	511337	10.119
NIAID AIDSno	511338	9.917
NIAID AIDSno	511339	10.940
NIAID AIDSno	511340	10.959

NIAID AIDSno	511341	10.602
NIAID AIDSno	511342	10.745
NIAID AIDSno	511343	9.876
NIAID AIDSno	511344	9.032
NIAID AIDSno	511345	10.824
NIAID AIDSno	511346	9.547
NIAID AIDSno	511347	9.432
NIAID AIDSno	511348	10.745
NIAID AIDSno	511349	10.268
NIAID AIDSno	511351	10.377
NIAID AIDSno	511352	10.194
NIAID AIDSno	511353	9.485
NIAID AIDSno	511354	10.456
NIAID AIDSno	511355	9.963
NIAID AIDSno	511356	10.921
NIAID AIDSno	511357	9.062
NIAID AIDSno	511358	9.285
NIAID AIDSno	511359	9.572
NIAID AIDSno	511360	9.483
NIAID AIDSno	511361	9.420
NIAID AIDSno	511362	10.770
NIAID AIDSno	511363	10.244
NIAID AIDSno	511364	9.697
NIAID AIDSno	511365	10.276
NIAID AIDSno	511366	9.453
NIAID AIDSno	511367	10.180
NIAID AIDSno	511368	8.855
NIAID AIDSno	511369	9.451
NIAID AIDSno	511370	9.708
NIAID AIDSno	511371	10.260
NIAID AIDSno	511372	9.792
NIAID AIDSno	511373	11.000
NIAID AIDSno	511374	9.064
NIAID AIDSno	511375	8.840
NIAID AIDSno	511376	10.194
NIAID AIDSno	511377	11.000
NIAID AIDSno	511378	10.678
NIAID AIDSno	511379	8.677
NIAID AIDSno	511380	11.000
NIAID AIDSno	511381	8.754
NIAID AIDSno	511382	11.000
NIAID AIDSno	511383	9.893
NIAID AIDSno	511384	11.000
NIAID AIDSno	511385	10.301
NIAID AIDSno	511386	10.086

NIAID AIDSno	511387	9.801
NIAID AIDSno	511388	9.227
NIAID AIDSno	511389	9.421
NIAID AIDSno	511390	9.425
NIAID AIDSno	511391	10.620
NIAID AIDSno	511392	8.495
NIAID AIDSno	511393	9.201
NIAID AIDSno	511394	9.410
NIAID AIDSno	511395	8.887
NIAID AIDSno	511396	10.260
NIAID AIDSno	511397	9.536
NIAID AIDSno	511398	11.000
NIAID AIDSno	511399	9.234
NIAID AIDSno	511400	11.000
NIAID AIDSno	511401	10.114
NIAID AIDSno	511402	9.122
NIAID AIDSno	511403	10.560
NIAID AIDSno	511404	8.588
NIAID AIDSno	511405	11.000
NIAID AIDSno	511406	9.762
NIAID AIDSno	511407	10.699
NIAID AIDSno	511408	8.995
NIAID AIDSno	511409	9.883
NIAID AIDSno	511410	9.198
NIAID AIDSno	511411	8.647
NIAID AIDSno	511412	9.873
NIAID AIDSno	511413	10.237
NIAID AIDSno	511414	10.959
NIAID AIDSno	511415	8.988
NIAID AIDSno	511416	9.689
NIAID AIDSno	511417	9.461
NIAID AIDSno	511418	8.495
NIAID AIDSno	511419	10.153
NIAID AIDSno	511424	9.536
NIAID AIDSno	511426	9.206
NIAID AIDSno	511427	10.824
NIAID AIDSno	511428	10.738
NIAID AIDSno	511432	9.733
NIAID AIDSno	511433	10.444
NIAID AIDSno	511434	11.000
NIAID AIDSno	511436	9.738
NIAID AIDSno	511438	9.429
NIAID AIDSno	511441	11.000
NIAID AIDSno	511443	11.000
NIAID AIDSno	511445	11.000

NIAID AIDSno	511448	10.469
NIAID AIDSno	511449	10.699
NIAID AIDSno	511450	9.378
NIAID AIDSno	511451	10.495
NIAID AIDSno	511452	10.602
NIAID AIDSno	511453	10.260
NIAID AIDSno	511454	10.097
NIAID AIDSno	511455	8.876
NIAID AIDSno	511456	11.000
NIAID AIDSno	511457	10.409
NIAID AIDSno	511458	11.000
NIAID AIDSno	511459	11.000
NIAID AIDSno	511461	10.854
NIAID AIDSno	511462	10.387
NIAID AIDSno	511463	9.245
NIAID AIDSno	511464	10.009
NIAID AIDSno	511465	9.225
NIAID AIDSno	511466	10.009
NIAID AIDSno	511467	10.796
NIAID AIDSno	511468	10.824
NIAID AIDSno	511469	9.249
NIAID AIDSno	511470	11.000
NIAID AIDSno	511471	9.900
NIAID AIDSno	511472	9.092
NIAID AIDSno	511473	10.387
NIAID AIDSno	511474	11.000
NIAID AIDSno	511475	9.631
NIAID AIDSno	511476	11.000
NIAID AIDSno	511477	9.812
NIAID AIDSno	511478	10.022
NIAID AIDSno	511479	10.208
NIAID AIDSno	511480	9.573
NIAID AIDSno	511481	10.000
NIAID AIDSno	511484	10.886
NIAID AIDSno	514135	10.260
NIAID AIDSno	514136	9.658
NIAID AIDSno	514137	8.444
NIAID AIDSno	514138	8.495
NIAID AIDSno	514139	10.301
NIAID AIDSno	514140	8.432
NIAID AIDSno	514141	7.658
NIAID AIDSno	514142	8.066
NIAID AIDSno	514143	7.824
NIAID AIDSno	514144	9.538
NIAID AIDSno	514145	10.301

NIAID AIDSno	514146	9.155
NIAID AIDSno	515677	7.699
NIAID AIDSno	515678	7.523
NIAID AIDSno	515679	7.495
NIAID AIDSno	515817	5.651
NIAID AIDSno	515829	5.000
NIAID AIDSno	515832	7.108
NIAID AIDSno	516446	10.721
NIAID AIDSno	516549	8.569
NIAID AIDSno	516550	9.000
NIAID AIDSno	516551	9.770
NIAID AIDSno	518766	7.780
NIAID AIDSno	518767	4.996
NIAID AIDSno	518768	4.830
NIAID AIDSno	518769	4.812
NIAID AIDSno	518770	6.857
NIAID AIDSno	518771	4.886
NIAID AIDSno	518772	4.818
NIAID AIDSno	518773	4.928
NIAID AIDSno	519282	8.420
NIAID AIDSno	519294	6.420
NIAID AIDSno	519299	6.770
NIAID AIDSno	519306	8.114
NIAID AIDSno	522104	5.794
NIAID AIDSno	522105	5.831
NIAID AIDSno	523013	8.721
NIAID AIDSno	523066	8.222
NIAID AIDSno	523070	5.398
NIAID AIDSno	523076	8.921
NIAID AIDSno	523081	5.824
NIAID AIDSno	523095	8.602
NIAID AIDSno	523096	8.456
NIAID AIDSno	523098	8.602
NIAID AIDSno	523118	8.602
NIAID AIDSno	523121	7.780
NIAID AIDSno	523122	7.812
NIAID AIDSno	523123	6.857
NIAID AIDSno	523124	8.699
NIAID AIDSno	523125	8.699
NIAID AIDSno	523126	8.244
NIAID AIDSno	523127	8.620
NIAID AIDSno	523128	9.357
NIAID AIDSno	523307	9.409
NIAID AIDSno	523310	9.585
NIAID AIDSno	523311	9.745

NIAID AIDSno	523312	9.658
NIAID AIDSno	523319	8.097
NIAID AIDSno	523320	7.796
NIAID AIDSno	523321	7.854
NIAID AIDSno	523322	7.699
NIAID AIDSno	523323	7.921
NIAID AIDSno	523324	7.699
NIAID AIDSno	523325	7.161
NIAID AIDSno	523346	9.959
NIAID AIDSno	523352	6.813
NIAID AIDSno	523363	4.000
NIAID AIDSno	523364	4.000
NIAID AIDSno	523365	6.337
NIAID AIDSno	523367	4.854
NIAID AIDSno	523368	3.754
NIAID AIDSno	524824	8.013
NIAID AIDSno	525367	8.237
NIAID AIDSno	525368	8.187
NIAID AIDSno	525369	8.357
NIAID AIDSno	525370	8.456
NIAID AIDSno	525371	8.194
NIAID AIDSno	525372	8.092
NIAID AIDSno	525373	8.237
NIAID AIDSno	525374	7.951
NIAID AIDSno	525375	8.796
NIAID AIDSno	525376	8.131
NIAID AIDSno	525377	8.284
NIAID AIDSno	525378	8.092
NIAID AIDSno	525379	8.367
NIAID AIDSno	525380	8.721
NIAID AIDSno	525381	8.415
NIAID AIDSno	525382	9.367
NIAID AIDSno	525383	9.319
NIAID AIDSno	525384	8.703
NIAID AIDSno	525385	8.604
NIAID AIDSno	525386	8.821
NIAID AIDSno	525387	8.337
NIAID AIDSno	525388	8.717
NIAID AIDSno	530716	3.886
NIAID AIDSno	530717	7.222
NIAID AIDSno	532009	9.222
NIAID AIDSno	532010	9.222
NIAID AIDSno	532011	8.745
NIAID AIDSno	532012	7.125
NIAID AIDSno	532013	6.212

NIAID AIDSno	532014	6.184
NIAID AIDSno	532015	5.503
NIAID AIDSno	532016	8.018
NIAID AIDSno	532017	6.757
NIAID AIDSno	532019	5.481
NIAID AIDSno	532020	3.538
NIAID AIDSno	532021	7.553
NIAID AIDSno	540353	4.222
NIAID AIDSno	541588	7.125
NIAID AIDSno	541590	5.187
NIAID AIDSno	541640	6.000
NIAID AIDSno	541641	6.301
NIAID AIDSno	542556	10.886
NIAID AIDSno	544303	4.568
NIAID AIDSno	544306	4.666
NIAID AIDSno	545467	5.624
NIAID AIDSno	545470	5.301
NIAID AIDSno	545473	5.875
NIAID AIDSno	545477	5.456
NIAID AIDSno	545480	5.959
NIAID AIDSno	545481	5.824
NIAID AIDSno	545483	5.516
NIAID AIDSno	545488	5.187
NIAID AIDSno	545490	5.602
NIAID AIDSno	545491	6.017
NIAID AIDSno	545492	5.523
NIAID AIDSno	545493	5.581
NIAID AIDSno	546657	7.138
NIAID AIDSno	546658	6.602
NIAID AIDSno	546659	7.917
NIAID AIDSno	546660	8.092
NIAID AIDSno	546661	9.268
NIAID AIDSno	546662	7.370
NIAID AIDSno	546663	8.456
NIAID AIDSno	546664	9.509
NIAID AIDSno	546665	9.201
NIAID AIDSno	546666	8.585
NIAID AIDSno	546700	8.793
NIAID AIDSno	546702	7.726
NIAID AIDSno	546705	8.409
NIAID AIDSno	546707	8.387
NIAID AIDSno	546711	8.180
NIAID AIDSno	546713	9.357
NIAID AIDSno	546715	8.620
NIAID AIDSno	546716	7.815

NIAID AIDSno	546723	8.157
NIAID AIDSno	546724	7.710
NIAID AIDSno	546726	7.541
NIAID AIDSno	546732	8.097
NIAID AIDSno	546734	7.810
NIAID AIDSno	546739	6.975
NIAID AIDSno	546742	8.441
NIAID AIDSno	546748	7.235
NIAID AIDSno	546755	8.181
NIAID AIDSno	546757	8.248
NIAID AIDSno	546758	7.866
NIAID AIDSno	546761	7.570
NIAID AIDSno	546764	6.757
NIAID AIDSno	546767	8.054
NIAID AIDSno	546770	6.959
NIAID AIDSno	546773	8.298
NIAID AIDSno	546779	9.392
NIAID AIDSno	546784	9.356
NIAID AIDSno	546787	8.987
NIAID AIDSno	546789	7.472
NIAID AIDSno	546791	6.734
NIAID AIDSno	546798	8.402
NIAID AIDSno	546801	6.917
NIAID AIDSno	546805	7.757
NIAID AIDSno	546807	8.699
NIAID AIDSno	546809	9.807
NIAID AIDSno	546810	7.664
NIAID AIDSno	546812	7.339
NIAID AIDSno	546814	7.226
NIAID AIDSno	546818	8.879
NIAID AIDSno	546820	8.567
NIAID AIDSno	546822	8.536
NIAID AIDSno	546824	7.638
NIAID AIDSno	546825	7.733
NIAID AIDSno	546826	8.400
NIAID AIDSno	546827	8.188
NIAID AIDSno	546828	9.745
NIAID AIDSno	546830	8.047
NIAID AIDSno	546831	7.924
NIAID AIDSno	546832	7.561
NIAID AIDSno	546833	8.026
NIAID AIDSno	546838	8.500
NIAID AIDSno	546841	8.178
NIAID AIDSno	546844	7.359
NIAID AIDSno	546846	8.693

NIAID AIDSno	546847	9.347
NIAID AIDSno	546849	8.103
NIAID AIDSno	546853	7.638
NIAID AIDSno	546856	7.180
NIAID AIDSno	546858	7.903
NIAID AIDSno	546859	7.516
NIAID AIDSno	546860	7.130
NIAID AIDSno	546862	7.034
NIAID AIDSno	546864	9.523
NIAID AIDSno	546866	9.125
NIAID AIDSno	546867	9.481
NIAID AIDSno	546868	9.469
NIAID AIDSno	546869	6.712
NIAID AIDSno	546871	9.481
NIAID AIDSno	546873	8.585
NIAID AIDSno	546874	7.587
NIAID AIDSno	546875	8.538
NIAID AIDSno	546876	7.177
NIAID AIDSno	546877	9.509
NIAID AIDSno	546878	10.046
NIAID AIDSno	546879	8.158
NIAID AIDSno	546880	8.710
NIAID AIDSno	546882	8.910
NIAID AIDSno	546883	8.901
NIAID AIDSno	546884	8.470
NIAID AIDSno	546885	9.046
NIAID AIDSno	546888	8.212
NIAID AIDSno	546889	9.071
NIAID AIDSno	546890	8.752
NIAID AIDSno	546891	7.460
NIAID AIDSno	546892	7.631
NIAID AIDSno	546893	8.027
NIAID AIDSno	546894	6.502
NIAID AIDSno	546895	7.979
NIAID AIDSno	546896	7.714
NIAID AIDSno	546897	7.134
NIAID AIDSno	546898	7.587
NIAID AIDSno	546899	9.114
NIAID AIDSno	546900	7.680
NIAID AIDSno	546923	8.451
NIAID AIDSno	546924	9.328
NIAID AIDSno	546925	8.614
NIAID AIDSno	546926	7.857
NIAID AIDSno	546927	8.523
NIAID AIDSno	546929	8.238

NIAID AIDSno	546930	8.201
NIAID AIDSno	546931	6.246
NIAID AIDSno	546932	6.388
NIAID AIDSno	546933	8.398
NIAID AIDSno	546934	8.097
NIAID AIDSno	546935	7.276
NIAID AIDSno	546936	8.155
NIAID AIDSno	546941	6.021
NIAID AIDSno	546944	7.102
NIAID AIDSno	546946	6.666
NIAID AIDSno	546948	8.322
NIAID AIDSno	546949	7.818
NIAID AIDSno	547381	6.125
NIAID AIDSno	547384	6.222
NIAID AIDSno	547385	6.125
NIAID AIDSno	547395	6.301
NIAID AIDSno	547398	6.301
NIAID AIDSno	547400	5.000
NIAID AIDSno	547401	5.000
NIAID AIDSno	547403	5.301
NIAID AIDSno	550327	5.000
NIAID AIDSno	550328	5.301
NIAID AIDSno	550331	5.301
NIAID AIDSno	550332	5.000
NIAID AIDSno	550335	8.398
NIAID AIDSno	550938	3.000
NIAID AIDSno	550939	3.301
NIAID AIDSno	550940	3.000
NIAID AIDSno	550941	4.456
NIAID AIDSno	550984	3.699
NIAID AIDSno	550986	3.900
NIAID AIDSno	551835	7.509
NIAID AIDSno	551836	6.635
NIAID AIDSno	552946	6.292
NIAID AIDSno	552947	7.854
NIAID AIDSno	552948	8.585
NIAID AIDSno	552949	7.071
NIAID AIDSno	552951	7.959
NIAID AIDSno	552952	8.602
NIAID AIDSno	555177	8.678
NIAID AIDSno	555180	8.585
NIAID AIDSno	555181	7.513
NIAID AIDSno	560768	8.301
NIAID AIDSno	560769	6.260
NIAID AIDSno	560770	7.456

NIAID AIDSno	560772	6.616
NIAID AIDSno	560773	5.445
NIAID AIDSno	560774	6.413
NIAID AIDSno	560776	7.301
NIAID AIDSno	560777	5.851
NIAID AIDSno	560779	5.367
NIAID AIDSno	560783	4.714
NIAID AIDSno	560786	4.928
NIAID AIDSno	560868	8.000
NIAID AIDSno	560869	6.845
NIAID AIDSno	560872	8.409
NIAID AIDSno	560881	5.699
NIAID AIDSno	560882	5.097
NIAID AIDSno	560883	5.523
NIAID AIDSno	560886	4.804
NIAID AIDSno	560888	7.523
NIAID AIDSno	560910	5.143
NIAID AIDSno	560911	4.575
NIAID AIDSno	560912	5.314
NIAID AIDSno	560915	5.319
NIAID AIDSno	560973	4.644
NIAID AIDSno	560974	5.056
NIAID AIDSno	560975	7.523
NIAID AIDSno	561016	4.678
NIAID AIDSno	561184	6.155
NIAID AIDSno	561192	6.398
NIAID AIDSno	561202	4.506
NIAID AIDSno	561203	3.905
NIAID AIDSno	561211	4.848
NIAID AIDSno	561212	5.638
NIAID AIDSno	561213	5.456
NIAID AIDSno	561214	5.721
NIAID AIDSno	561215	5.495
NIAID AIDSno	561216	5.854
NIAID AIDSno	561217	4.845
NIAID AIDSno	561218	4.561
NIAID AIDSno	561223	3.646
NIAID AIDSno	561226	3.824
NIAID AIDSno	561228	4.137
NIAID AIDSno	561231	6.097
NIAID AIDSno	561232	5.721
NIAID AIDSno	561233	4.807
NIAID AIDSno	561234	5.284
NIAID AIDSno	561236	5.409
NIAID AIDSno	563527	8.854

NIAID AIDSno	563529	7.187
NIAID AIDSno	563531	7.824
NIAID AIDSno	563532	7.602
NIAID AIDSno	563533	7.097
NIAID AIDSno	563534	8.004
NIAID AIDSno	563535	9.097
NIAID AIDSno	563537	7.387
NIAID AIDSno	563538	7.056
NIAID AIDSno	563539	7.770
NIAID AIDSno	563540	7.097
NIAID AIDSno	563541	8.444
NIAID AIDSno	563542	6.959
NIAID AIDSno	563543	8.195
NIAID AIDSno	563545	8.200
NIAID AIDSno	563547	8.387
NIAID AIDSno	563548	7.569
NIAID AIDSno	563549	7.553
NIAID AIDSno	563552	7.319
NIAID AIDSno	563554	7.108
NIAID AIDSno	563556	7.284
NIAID AIDSno	563557	7.237
NIAID AIDSno	563559	7.097
NIAID AIDSno	563561	7.071
NIAID AIDSno	563562	6.921
NIAID AIDSno	563564	6.678
NIAID AIDSno	563566	6.745
NIAID AIDSno	563571	6.602
NIAID AIDSno	563573	7.745
NIAID AIDSno	563575	7.495
NIAID AIDSno	563576	7.538
NIAID AIDSno	563577	7.678
NIAID AIDSno	563579	8.244
NIAID AIDSno	563581	7.854
NIAID AIDSno	563582	7.222
NIAID AIDSno	563584	7.620
NIAID AIDSno	563585	6.745
NIAID AIDSno	563587	9.222
NIAID AIDSno	563588	8.638
NIAID AIDSno	563589	7.284
NIAID AIDSno	563590	8.538
NIAID AIDSno	563593	7.770
NIAID AIDSno	563594	7.018
NIAID AIDSno	563596	7.585
NIAID AIDSno	563597	7.678
NIAID AIDSno	563599	7.301

NIAID AIDSno	563602	7.076
NIAID AIDSno	563603	7.041
NIAID AIDSno	563605	7.027
NIAID AIDSno	563606	7.310
NIAID AIDSno	563608	7.420
NIAID AIDSno	563609	7.018
NIAID AIDSno	563610	7.921
NIAID AIDSno	563611	7.585
NIAID AIDSno	563613	7.523
NIAID AIDSno	563614	7.469
NIAID AIDSno	563616	7.301
NIAID AIDSno	563617	7.180
NIAID AIDSno	563618	7.149
NIAID AIDSno	563619	7.143
NIAID AIDSno	563620	6.796
NIAID AIDSno	563621	6.886
NIAID AIDSno	563623	7.310
NIAID AIDSno	563624	7.252
NIAID AIDSno	563625	7.367
NIAID AIDSno	563627	7.000
NIAID AIDSno	563628	7.585
NIAID AIDSno	563630	10.523
NIAID AIDSno	563631	8.469
NIAID AIDSno	563632	7.620
NIAID AIDSno	563633	8.886
NIAID AIDSno	563635	7.854
NIAID AIDSno	563636	7.678
NIAID AIDSno	563637	8.000
NIAID AIDSno	563638	8.387
NIAID AIDSno	563639	7.284
NIAID AIDSno	563640	7.824
NIAID AIDSno	563641	7.391
NIAID AIDSno	563642	8.444
NIAID AIDSno	563643	8.027
NIAID AIDSno	563644	9.398
NIAID AIDSno	563645	8.959
NIAID AIDSno	563647	6.174
NIAID AIDSno	563704	8.737
NIAID AIDSno	563705	8.620
NIAID AIDSno	563706	8.305
NIAID AIDSno	563707	8.433
NIAID AIDSno	563708	8.770
NIAID AIDSno	563709	9.398
NIAID AIDSno	563710	8.312
NIAID AIDSno	563711	9.097

NIAID AIDSno	563718	9.523
NIAID AIDSno	563727	7.770
NIAID AIDSno	563730	8.161
NIAID AIDSno	563732	8.602
NIAID AIDSno	563734	8.495
NIAID AIDSno	563737	7.512
NIAID AIDSno	563738	7.167
NIAID AIDSno	563748	8.678
NIAID AIDSno	563750	6.745
NIAID AIDSno	563758	7.886
NIAID AIDSno	563760	8.310
NIAID AIDSno	563761	7.658
NIAID AIDSno	563762	8.854
NIAID AIDSno	563763	7.959
NIAID AIDSno	563764	7.000
NIAID AIDSno	563768	8.292
NIAID AIDSno	563769	8.000
NIAID AIDSno	563770	8.215
NIAID AIDSno	563771	8.018
NIAID AIDSno	563775	7.921
NIAID AIDSno	563776	6.292
NIAID AIDSno	563777	5.959
NIAID AIDSno	563778	8.469
NIAID AIDSno	563779	8.347
NIAID AIDSno	563780	7.854
NIAID AIDSno	563781	8.824
NIAID AIDSno	563807	5.762
NIAID AIDSno	563808	6.292
NIAID AIDSno	563809	6.886
NIAID AIDSno	563810	6.886
NIAID AIDSno	563812	6.921
NIAID AIDSno	563813	7.000
NIAID AIDSno	563815	7.041
NIAID AIDSno	563817	7.102
NIAID AIDSno	563818	7.167
NIAID AIDSno	563819	7.357
NIAID AIDSno	563821	7.387
NIAID AIDSno	563823	7.509
NIAID AIDSno	563824	7.553
NIAID AIDSno	563826	7.553
NIAID AIDSno	563835	7.585
NIAID AIDSno	563836	7.620
NIAID AIDSno	563837	7.678
NIAID AIDSno	563838	7.699
NIAID AIDSno	563839	7.745

NIAID AIDSno	563840	7.796
NIAID AIDSno	563841	7.886
NIAID AIDSno	563842	7.921
NIAID AIDSno	563843	7.959
NIAID AIDSno	563845	8.215
NIAID AIDSno	563846	8.284
NIAID AIDSno	563847	8.456
NIAID AIDSno	563848	8.481
NIAID AIDSno	563849	8.569
NIAID AIDSno	563850	8.678
NIAID AIDSno	563852	8.484
NIAID AIDSno	563856	5.569
NIAID AIDSno	563857	6.444
NIAID AIDSno	563858	6.469
NIAID AIDSno	563859	6.721
NIAID AIDSno	563860	6.721
NIAID AIDSno	563861	6.745
NIAID AIDSno	563862	6.824
NIAID AIDSno	563863	6.824
NIAID AIDSno	563865	7.536
NIAID AIDSno	563866	7.013
NIAID AIDSno	563867	7.161
NIAID AIDSno	563868	7.161
NIAID AIDSno	563869	7.161
NIAID AIDSno	563871	7.347
NIAID AIDSno	563872	7.409
NIAID AIDSno	563873	7.495
NIAID AIDSno	563874	7.602
NIAID AIDSno	563875	7.620
NIAID AIDSno	563876	7.658
NIAID AIDSno	563877	7.796
NIAID AIDSno	563886	7.886
NIAID AIDSno	563887	7.921
NIAID AIDSno	563889	8.027
NIAID AIDSno	563890	8.137
NIAID AIDSno	563891	8.086
NIAID AIDSno	563892	8.114
NIAID AIDSno	563894	8.119
NIAID AIDSno	563895	8.194
NIAID AIDSno	563896	8.244
NIAID AIDSno	563897	8.292
NIAID AIDSno	563898	8.301
NIAID AIDSno	563899	8.310
NIAID AIDSno	563902	8.367
NIAID AIDSno	563903	8.398

NIAID AIDSno	563904	8.432
NIAID AIDSno	563905	8.444
NIAID AIDSno	563907	8.509
NIAID AIDSno	563908	8.509
NIAID AIDSno	563909	8.553
NIAID AIDSno	563912	8.569
NIAID AIDSno	563913	8.678
NIAID AIDSno	563914	8.699
NIAID AIDSno	563915	8.699
NIAID AIDSno	563916	8.854
NIAID AIDSno	563917	8.886
NIAID AIDSno	563918	9.398
NIAID AIDSno	563919	9.699
NIAID AIDSno	563920	8.495
NIAID AIDSno	563921	8.495
NIAID AIDSno	563923	9.000
NIAID AIDSno	563929	8.432
NIAID AIDSno	563933	7.854
NIAID AIDSno	563934	8.886
NIAID AIDSno	563935	7.921
NIAID AIDSno	563936	8.337
NIAID AIDSno	563937	7.930
NIAID AIDSno	563938	8.495
NIAID AIDSno	563939	8.796
NIAID AIDSno	563940	7.523
NIAID AIDSno	563941	7.553
NIAID AIDSno	563942	8.444
NIAID AIDSno	563943	7.000
NIAID AIDSno	563944	8.420
NIAID AIDSno	563945	8.602
NIAID AIDSno	563947	9.000
NIAID AIDSno	563948	9.523
NIAID AIDSno	563949	7.824
NIAID AIDSno	563951	8.796
NIAID AIDSno	563978	8.276
NIAID AIDSno	563981	6.638
NIAID AIDSno	563984	6.770
NIAID AIDSno	563985	8.523
NIAID AIDSno	566144	9.959
NIAID AIDSno	566146	10.000
NIAID AIDSno	566147	10.301
NIAID AIDSno	566149	9.921
NIAID AIDSno	566150	9.367
NIAID AIDSno	569610	3.860
NIAID AIDSno	569628	4.921

NIAID AIDSno	569630	4.337
NIAID AIDSno	569633	6.000
NIAID AIDSno	569634	3.461
NIAID AIDSno	569636	2.580
NIAID AIDSno	569638	3.474
NIAID AIDSno	569640	2.357
NIAID AIDSno	569643	3.218
NIAID AIDSno	569646	5.699
NIAID AIDSno	569647	3.211
NIAID AIDSno	569648	2.854
NIAID AIDSno	569649	5.097
NIAID AIDSno	570394	5.252
NIAID AIDSno	570399	6.155
NIAID AIDSno	570400	5.125
NIAID AIDSno	570402	5.721
NIAID AIDSno	570422	5.585
NIAID AIDSno	570521	6.919
NIAID AIDSno	576715	5.854
NIAID AIDSno	576717	9.319
NIAID AIDSno	576743	9.084
NIAID AIDSno	576748	8.849
NIAID AIDSno	576763	8.886
NIAID AIDSno	576764	8.456
NIAID AIDSno	576778	10.523
NIAID AIDSno	576806	5.886
NIAID AIDSno	576807	7.155
NIAID AIDSno	576940	8.215
NIAID AIDSno	577403	8.301
NIAID AIDSno	577458	8.000
NIAID AIDSno	577459	7.620
NIAID AIDSno	577460	7.886
NIAID AIDSno	577462	6.886
NIAID AIDSno	577466	6.602
NIAID AIDSno	579363	6.325
NIAID AIDSno	579365	6.268
NIAID AIDSno	579366	6.538
NIAID AIDSno	579369	5.948
NIAID AIDSno	579372	7.824
NIAID AIDSno	579374	6.745
NIAID AIDSno	579377	5.119
NIAID AIDSno	579381	5.672
NIAID AIDSno	579383	5.991
NIAID AIDSno	579385	5.697
NIAID AIDSno	579387	6.184
NIAID AIDSno	579390	5.884

NIAID AIDSno	579392	5.179
NIAID AIDSno	579395	5.728
NIAID AIDSno	579397	5.517
NIAID AIDSno	579398	6.006
NIAID AIDSno	579399	6.390
NIAID AIDSno	579400	6.088
NIAID AIDSno	579401	5.917
NIAID AIDSno	579402	6.824
NIAID AIDSno	579403	5.632
NIAID AIDSno	579404	5.852
NIAID AIDSno	579405	6.093
NIAID AIDSno	579479	4.762
NIAID AIDSno	582422	7.757
NIAID AIDSno	582423	6.653
NIAID AIDSno	582425	8.124
NIAID AIDSno	582427	7.974
NIAID AIDSno	582428	8.305
NIAID AIDSno	582432	8.290
NIAID AIDSno	582433	8.100
NIAID AIDSno	582435	8.078
NIAID AIDSno	582438	8.071
NIAID AIDSno	582441	8.025
NIAID AIDSno	582442	7.931
NIAID AIDSno	582443	7.926
NIAID AIDSno	582444	7.903
NIAID AIDSno	582445	7.847
NIAID AIDSno	582446	7.751
NIAID AIDSno	582447	7.667
NIAID AIDSno	582448	7.600
NIAID AIDSno	582449	7.457
NIAID AIDSno	582450	7.383
NIAID AIDSno	582451	7.364
NIAID AIDSno	582452	7.354
NIAID AIDSno	582453	7.352
NIAID AIDSno	582454	7.285
NIAID AIDSno	582455	7.235
NIAID AIDSno	582456	7.000
NIAID AIDSno	582457	6.906
NIAID AIDSno	582458	6.905
NIAID AIDSno	582459	6.670
NIAID AIDSno	582460	6.628
NIAID AIDSno	582461	10.476
NIAID AIDSno	582462	9.768
NIAID AIDSno	582465	9.204
NIAID AIDSno	582468	9.326

NIAID AIDSno	582469	10.518
NIAID AIDSno	582470	6.812
NIAID AIDSno	582471	8.509
NIAID AIDSno	582472	8.357
NIAID AIDSno	582473	6.377
NIAID AIDSno	582474	6.125
NIAID AIDSno	582475	6.051
NIAID AIDSno	582476	7.097
NIAID AIDSno	582477	9.301
NIAID AIDSno	582478	7.424
NIAID AIDSno	582479	6.793
NIAID AIDSno	582480	7.695
NIAID AIDSno	582481	8.196
NIAID AIDSno	582483	7.523
NIAID AIDSno	582484	7.114
NIAID AIDSno	582485	7.284
NIAID AIDSno	582487	7.523
NIAID AIDSno	582488	8.161
NIAID AIDSno	582489	7.770
NIAID AIDSno	582491	7.569
NIAID AIDSno	582492	6.770
NIAID AIDSno	582493	6.886
NIAID AIDSno	583438	10.427
NIAID AIDSno	583439	8.099
NIAID AIDSno	583440	10.195
NIAID AIDSno	583441	10.035
NIAID AIDSno	583442	8.130
NIAID AIDSno	583443	9.476
NIAID AIDSno	583444	7.430
NIAID AIDSno	583445	9.297
NIAID AIDSno	583446	10.287
NIAID AIDSno	583447	8.800
NIAID AIDSno	583448	8.105
NIAID AIDSno	583449	9.151
NIAID AIDSno	583450	9.862
NIAID AIDSno	583451	9.228
NIAID AIDSno	583452	10.252
NIAID AIDSno	583453	7.609
NIAID AIDSno	583454	10.127
NIAID AIDSno	583455	9.902
NIAID AIDSno	583456	9.242
NIAID AIDSno	583457	9.280
NIAID AIDSno	583458	8.774
NIAID AIDSno	583459	8.714
NIAID AIDSno	583460	8.504

NIAID AIDSno	583461	7.787
NIAID AIDSno	583462	7.585
NIAID AIDSno	583463	7.183
NIAID AIDSno	583464	9.182
NIAID AIDSno	583465	9.914
NIAID AIDSno	583466	10.896
NIAID AIDSno	583467	9.835
NIAID AIDSno	583468	11.015
NIAID AIDSno	583469	10.398
NIAID AIDSno	583470	8.390
NIAID AIDSno	583471	9.775
NIAID AIDSno	583472	9.799
NIAID AIDSno	583473	7.784
NIAID AIDSno	583474	8.654
NIAID AIDSno	583475	8.700
NIAID AIDSno	583477	7.957
NIAID AIDSno	583478	8.581
NIAID AIDSno	583479	8.991
NIAID AIDSno	583480	10.387
NIAID AIDSno	583481	10.387
NIAID AIDSno	583482	6.075
NIAID AIDSno	583483	6.422
NIAID AIDSno	583484	8.742
NIAID AIDSno	583485	6.084
NIAID AIDSno	583486	7.441
NIAID AIDSno	583487	6.239
NIAID AIDSno	583546	6.602
NIAID AIDSno	583547	7.353
NIAID AIDSno	583548	6.845
NIAID AIDSno	583549	6.817
NIAID AIDSno	583550	7.008
NIAID AIDSno	583551	7.154
NIAID AIDSno	583552	7.118
NIAID AIDSno	583553	7.016
NIAID AIDSno	583554	7.053
NIAID AIDSno	583555	6.260
NIAID AIDSno	583556	6.806
NIAID AIDSno	583563	11.000
NIAID AIDSno	583564	11.000
NIAID AIDSno	583565	11.000
NIAID AIDSno	583566	11.000
NIAID AIDSno	583567	11.000
NIAID AIDSno	583568	11.000
NIAID AIDSno	583569	11.000
NIAID AIDSno	583571	8.456

NIAID AIDSno	583572	7.208
NIAID AIDSno	583573	7.229
NIAID AIDSno	583574	6.658
NIAID AIDSno	583575	8.553
NIAID AIDSno	583576	6.553
NIAID AIDSno	583577	7.051
NIAID AIDSno	583580	8.745
NIAID AIDSno	583581	6.509
NIAID AIDSno	583582	6.824
NIAID AIDSno	583584	10.155
NIAID AIDSno	583585	10.222
NIAID AIDSno	583586	10.699
NIAID AIDSno	583587	9.658
NIAID AIDSno	583588	10.523
NIAID AIDSno	583589	8.678
NIAID AIDSno	583590	10.398
NIAID AIDSno	583591	10.301
NIAID AIDSno	583592	7.959
NIAID AIDSno	583595	7.585
NIAID AIDSno	583596	7.770
NIAID AIDSno	583599	7.509
NIAID AIDSno	583600	8.959
NIAID AIDSno	584731	4.208
NIAID AIDSno	584732	3.790
NIAID AIDSno	588084	4.418
NIAID AIDSno	588095	4.202
NIAID AIDSno	588097	4.155
NIAID AIDSno	588194	6.775
NIAID AIDSno	589302	8.553
NIAID AIDSno	589303	8.894
NIAID AIDSno	589305	9.000
NIAID AIDSno	589306	9.137
NIAID AIDSno	589307	8.959
NIAID AIDSno	589308	8.396
NIAID AIDSno	589310	7.620
NIAID AIDSno	589321	8.215
NIAID AIDSno	589322	8.469
NIAID AIDSno	589324	8.456
NIAID AIDSno	589325	7.432
NIAID AIDSno	589326	7.699
NIAID AIDSno	589327	7.921
NIAID AIDSno	589328	8.509
NIAID AIDSno	589329	8.456
NIAID AIDSno	589330	8.004
NIAID AIDSno	589335	9.097

NIAID AIDSno	597832	9.000
NIAID AIDSno	597835	5.398
NIAID AIDSno	597837	7.824
NIAID AIDSno	597838	8.959
NIAID AIDSno	597839	8.699
NIAID AIDSno	597840	7.565
NIAID AIDSno	597989	8.652
NIAID AIDSno	597990	7.565
NIAID AIDSno	597991	8.386
NIAID AIDSno	597992	7.991
NIAID AIDSno	597993	6.538
NIAID AIDSno	597994	6.680
NIAID AIDSno	597995	8.783
NIAID AIDSno	597996	7.690
NIAID AIDSno	597997	7.474
NIAID AIDSno	597998	7.244
NIAID AIDSno	597999	7.714
NIAID AIDSno	598000	6.276
NIAID AIDSno	598002	8.004
NIAID AIDSno	598003	7.544
NIAID AIDSno	598004	6.569
NIAID AIDSno	598005	7.420
NIAID AIDSno	598006	7.187
NIAID AIDSno	598008	7.357
NIAID AIDSno	598009	7.008
NIAID AIDSno	598010	6.796
NIAID AIDSno	598011	6.959
NIAID AIDSno	598012	6.041
NIAID AIDSno	598014	7.419
NIAID AIDSno	598015	9.456
NIAID AIDSno	598016	7.958
NIAID AIDSno	598017	9.056
NIAID AIDSno	598018	8.371
NIAID AIDSno	598019	7.167
NIAID AIDSno	598020	7.182
NIAID AIDSno	598021	9.114
NIAID AIDSno	598022	7.900
NIAID AIDSno	598023	6.536
NIAID AIDSno	598024	7.223
NIAID AIDSno	598025	7.644
NIAID AIDSno	598028	7.640
NIAID AIDSno	598036	7.940
NIAID AIDSno	598862	6.754
NIAID AIDSno	598863	7.091
NIAID AIDSno	598867	7.337

NIAID AIDSno	598868	7.509
NIAID AIDSno	598869	8.276
NIAID AIDSno	598870	8.699
NIAID AIDSno	599845	10.921
NIAID AIDSno	600226	8.747
NIAID AIDSno	600229	7.036
NIAID AIDSno	600230	7.652
NIAID AIDSno	600231	8.004
NIAID AIDSno	600232	6.692
NIAID AIDSno	600233	6.858
NIAID AIDSno	600234	6.888
NIAID AIDSno	603496	10.301
NIAID AIDSno	603498	9.854
NIAID AIDSno	603499	9.677
NIAID AIDSno	603500	9.105
NIAID AIDSno	603505	9.585
NIAID AIDSno	603507	9.409
NIAID AIDSno	603509	9.284
NIAID AIDSno	603510	8.138
NIAID AIDSno	603512	8.291
NIAID AIDSno	603513	8.932
NIAID AIDSno	603514	8.879
NIAID AIDSno	603515	8.812
NIAID AIDSno	603520	8.752
NIAID AIDSno	603521	8.710
NIAID AIDSno	603522	8.703
NIAID AIDSno	603523	8.701
NIAID AIDSno	603525	8.672
NIAID AIDSno	603526	8.638
NIAID AIDSno	603527	8.636
NIAID AIDSno	603528	8.635
NIAID AIDSno	603529	8.623
NIAID AIDSno	603530	8.618
NIAID AIDSno	603533	8.587
NIAID AIDSno	603535	8.545
NIAID AIDSno	603536	8.541
NIAID AIDSno	603538	8.445
NIAID AIDSno	603540	8.387
NIAID AIDSno	603544	8.504
NIAID AIDSno	603546	8.493
NIAID AIDSno	603548	8.487
NIAID AIDSno	603552	8.474
NIAID AIDSno	603554	8.466
NIAID AIDSno	603556	8.455
NIAID AIDSno	603558	8.410

NIAID AIDSno	603560	8.395
NIAID AIDSno	603562	8.387
NIAID AIDSno	603565	8.384
NIAID AIDSno	603568	8.371
NIAID AIDSno	603569	8.350
NIAID AIDSno	603571	8.342
NIAID AIDSno	603574	8.337
NIAID AIDSno	603577	8.317
NIAID AIDSno	603578	8.309
NIAID AIDSno	603579	8.279
NIAID AIDSno	603581	8.265
NIAID AIDSno	603585	8.260
NIAID AIDSno	603591	8.259
NIAID AIDSno	603594	8.253
NIAID AIDSno	603596	8.249
NIAID AIDSno	603597	8.248
NIAID AIDSno	603598	8.223
NIAID AIDSno	603599	8.208
NIAID AIDSno	603600	8.205
NIAID AIDSno	603601	8.192
NIAID AIDSno	603602	8.190
NIAID AIDSno	603603	8.184
NIAID AIDSno	603604	8.180
NIAID AIDSno	603605	8.164
NIAID AIDSno	603606	8.159
NIAID AIDSno	603607	8.149
NIAID AIDSno	603608	8.131
NIAID AIDSno	603609	8.112
NIAID AIDSno	603610	8.104
NIAID AIDSno	603611	8.081
NIAID AIDSno	603612	7.621
NIAID AIDSno	603613	8.051
NIAID AIDSno	603614	8.037
NIAID AIDSno	603615	8.036
NIAID AIDSno	603616	8.032
NIAID AIDSno	603617	7.757
NIAID AIDSno	603618	7.991
NIAID AIDSno	603619	7.984
NIAID AIDSno	603621	7.806
NIAID AIDSno	603622	7.978
NIAID AIDSno	603623	7.966
NIAID AIDSno	603624	7.940
NIAID AIDSno	603661	7.860
NIAID AIDSno	603662	7.883
NIAID AIDSno	603663	7.857

NIAID AIDSno	603664	7.850
NIAID AIDSno	603665	7.841
NIAID AIDSno	603666	7.840
NIAID AIDSno	603667	7.830
NIAID AIDSno	603668	7.818
NIAID AIDSno	603669	7.804
NIAID AIDSno	603671	7.787
NIAID AIDSno	603672	7.762
NIAID AIDSno	603674	7.733
NIAID AIDSno	603675	7.718
NIAID AIDSno	603676	7.711
NIAID AIDSno	603678	7.691
NIAID AIDSno	603679	7.687
NIAID AIDSno	603681	7.684
NIAID AIDSno	603683	7.663
NIAID AIDSno	603684	7.635
NIAID AIDSno	603686	7.623
NIAID AIDSno	603687	7.616
NIAID AIDSno	603689	7.608
NIAID AIDSno	603690	7.595
NIAID AIDSno	603693	7.586
NIAID AIDSno	603694	7.583
NIAID AIDSno	603697	7.571
NIAID AIDSno	603698	7.549
NIAID AIDSno	603699	7.513
NIAID AIDSno	603703	7.462
NIAID AIDSno	603704	7.438
NIAID AIDSno	603705	7.423
NIAID AIDSno	603707	7.410
NIAID AIDSno	603709	7.375
NIAID AIDSno	603710	7.354
NIAID AIDSno	603712	7.317
NIAID AIDSno	603713	7.254
NIAID AIDSno	603714	7.169
NIAID AIDSno	603715	7.243
NIAID AIDSno	603716	7.221
NIAID AIDSno	603720	7.168
NIAID AIDSno	603721	7.150
NIAID AIDSno	603723	7.071
NIAID AIDSno	603724	7.062
NIAID AIDSno	603726	7.062
NIAID AIDSno	603728	8.967
NIAID AIDSno	603729	8.893
NIAID AIDSno	603730	8.742
NIAID AIDSno	603731	8.726

NIAID AIDSno	603732	8.556
NIAID AIDSno	603733	8.533
NIAID AIDSno	603734	8.533
NIAID AIDSno	603735	8.400
NIAID AIDSno	603736	8.491
NIAID AIDSno	603737	7.961
NIAID AIDSno	603738	8.428
NIAID AIDSno	603739	7.914
NIAID AIDSno	603740	8.390
NIAID AIDSno	603741	8.323
NIAID AIDSno	603744	8.281
NIAID AIDSno	603746	8.179
NIAID AIDSno	603747	8.108
NIAID AIDSno	603748	7.997
NIAID AIDSno	603749	7.991
NIAID AIDSno	603750	7.946
NIAID AIDSno	603751	7.869
NIAID AIDSno	603752	7.850
NIAID AIDSno	603753	7.802
NIAID AIDSno	603754	7.798
NIAID AIDSno	603755	7.694
NIAID AIDSno	603756	7.599
NIAID AIDSno	603757	7.491
NIAID AIDSno	603758	7.477
NIAID AIDSno	603759	7.467
NIAID AIDSno	603760	7.467
NIAID AIDSno	603763	7.414
NIAID AIDSno	603764	7.413
NIAID AIDSno	603765	7.395
NIAID AIDSno	603766	7.275
NIAID AIDSno	603767	7.273
NIAID AIDSno	603768	7.273
NIAID AIDSno	603769	7.260
NIAID AIDSno	603770	7.224
NIAID AIDSno	603771	7.198
NIAID AIDSno	603772	7.153
NIAID AIDSno	603773	7.145
NIAID AIDSno	603774	7.105
NIAID AIDSno	603775	7.104
NIAID AIDSno	603776	7.047
NIAID AIDSno	603777	8.770
NIAID AIDSno	603778	8.646
NIAID AIDSno	603779	8.562
NIAID AIDSno	603780	7.393
NIAID AIDSno	603781	7.945

NIAID AIDSno	603782	8.435
NIAID AIDSno	603783	8.423
NIAID AIDSno	603784	8.417
NIAID AIDSno	603785	8.413
NIAID AIDSno	603786	8.380
NIAID AIDSno	603787	7.744
NIAID AIDSno	603788	8.311
NIAID AIDSno	603789	7.448
NIAID AIDSno	603790	7.245
NIAID AIDSno	603791	7.500
NIAID AIDSno	603792	8.280
NIAID AIDSno	603794	8.271
NIAID AIDSno	603795	8.253
NIAID AIDSno	603796	7.847
NIAID AIDSno	603797	8.199
NIAID AIDSno	603798	8.184
NIAID AIDSno	603799	8.173
NIAID AIDSno	603800	8.132
NIAID AIDSno	603801	8.115
NIAID AIDSno	603802	8.086
NIAID AIDSno	603804	7.453
NIAID AIDSno	603805	7.900
NIAID AIDSno	603806	8.040
NIAID AIDSno	603807	7.535
NIAID AIDSno	603808	8.023
NIAID AIDSno	603809	8.010
NIAID AIDSno	603810	8.008
NIAID AIDSno	603811	7.639
NIAID AIDSno	603812	7.994
NIAID AIDSno	603813	7.987
NIAID AIDSno	603814	7.978
NIAID AIDSno	603815	7.960
NIAID AIDSno	603816	7.959
NIAID AIDSno	603817	7.957
NIAID AIDSno	603818	7.956
NIAID AIDSno	603820	7.926
NIAID AIDSno	603821	7.925
NIAID AIDSno	603822	7.907
NIAID AIDSno	603823	7.753
NIAID AIDSno	603824	7.883
NIAID AIDSno	603825	7.882
NIAID AIDSno	603826	7.537
NIAID AIDSno	603827	7.877
NIAID AIDSno	603828	7.869
NIAID AIDSno	603829	7.855

NIAID AIDSno	603830	7.848
NIAID AIDSno	603831	7.841
NIAID AIDSno	603832	7.841
NIAID AIDSno	603833	7.840
NIAID AIDSno	603834	7.824
NIAID AIDSno	603835	7.821
NIAID AIDSno	603836	7.819
NIAID AIDSno	603837	7.806
NIAID AIDSno	603838	7.780
NIAID AIDSno	603839	7.776
NIAID AIDSno	603840	7.776
NIAID AIDSno	603841	7.767
NIAID AIDSno	603842	7.758
NIAID AIDSno	603869	7.741
NIAID AIDSno	603870	7.725
NIAID AIDSno	603872	7.717
NIAID AIDSno	603873	7.703
NIAID AIDSno	603874	7.697
NIAID AIDSno	603875	7.694
NIAID AIDSno	603876	7.680
NIAID AIDSno	603877	7.675
NIAID AIDSno	603878	7.674
NIAID AIDSno	603879	7.655
NIAID AIDSno	603881	7.647
NIAID AIDSno	603882	7.635
NIAID AIDSno	603886	7.629
NIAID AIDSno	603887	7.612
NIAID AIDSno	603888	7.607
NIAID AIDSno	603889	7.606
NIAID AIDSno	603892	7.584
NIAID AIDSno	603896	7.580
NIAID AIDSno	603899	7.572
NIAID AIDSno	603901	7.570
NIAID AIDSno	603902	7.569
NIAID AIDSno	603903	7.554
NIAID AIDSno	603906	7.548
NIAID AIDSno	603907	7.545
NIAID AIDSno	603909	7.541
NIAID AIDSno	603910	7.540
NIAID AIDSno	603911	7.529
NIAID AIDSno	603912	7.527
NIAID AIDSno	603913	6.740
NIAID AIDSno	603914	7.515
NIAID AIDSno	603915	7.508
NIAID AIDSno	603916	7.508

NIAID AIDSno	603919	7.503
NIAID AIDSno	603920	7.498
NIAID AIDSno	603921	7.485
NIAID AIDSno	603922	7.478
NIAID AIDSno	603923	7.472
NIAID AIDSno	603924	7.469
NIAID AIDSno	603925	7.453
NIAID AIDSno	603927	7.442
NIAID AIDSno	603929	7.440
NIAID AIDSno	603931	7.298
NIAID AIDSno	603933	7.409
NIAID AIDSno	603935	7.407
NIAID AIDSno	603936	7.400
NIAID AIDSno	603937	7.397
NIAID AIDSno	603938	7.395
NIAID AIDSno	603941	7.362
NIAID AIDSno	603943	7.361
NIAID AIDSno	603946	7.357
NIAID AIDSno	603948	7.357
NIAID AIDSno	603949	7.222
NIAID AIDSno	603952	7.343
NIAID AIDSno	603954	7.338
NIAID AIDSno	603956	7.338
NIAID AIDSno	603957	7.193
NIAID AIDSno	603959	7.324
NIAID AIDSno	603960	7.322
NIAID AIDSno	603961	7.310
NIAID AIDSno	603962	6.913
NIAID AIDSno	603963	7.286
NIAID AIDSno	603964	7.286
NIAID AIDSno	603980	7.283
NIAID AIDSno	603982	7.283
NIAID AIDSno	603983	7.283
NIAID AIDSno	603984	7.281
NIAID AIDSno	603985	7.277
NIAID AIDSno	603986	7.261
NIAID AIDSno	603987	7.250
NIAID AIDSno	603988	7.250
NIAID AIDSno	603999	7.246
NIAID AIDSno	604000	7.246
NIAID AIDSno	604001	7.245
NIAID AIDSno	604002	7.244
NIAID AIDSno	604004	7.233
NIAID AIDSno	604005	7.226
NIAID AIDSno	604006	7.094

NIAID AIDSno	604007	7.188
NIAID AIDSno	604009	7.170
NIAID AIDSno	604010	7.168
NIAID AIDSno	604011	7.157
NIAID AIDSno	604012	7.155
NIAID AIDSno	604013	7.152
NIAID AIDSno	604014	7.149
NIAID AIDSno	604015	7.143
NIAID AIDSno	604016	7.134
NIAID AIDSno	604017	7.121
NIAID AIDSno	604018	7.117
NIAID AIDSno	604019	7.111
NIAID AIDSno	604020	7.103
NIAID AIDSno	604021	7.090
NIAID AIDSno	604022	7.087
NIAID AIDSno	604023	7.084
NIAID AIDSno	604024	7.079
NIAID AIDSno	604025	7.075
NIAID AIDSno	604026	7.075
NIAID AIDSno	604027	7.069
NIAID AIDSno	604028	7.062
NIAID AIDSno	604029	7.061
NIAID AIDSno	604030	7.052
NIAID AIDSno	604031	7.050
NIAID AIDSno	604032	7.047
NIAID AIDSno	604033	7.040
NIAID AIDSno	604034	7.040
NIAID AIDSno	604035	7.029
NIAID AIDSno	604036	7.027
NIAID AIDSno	604037	7.022
NIAID AIDSno	604038	7.017
NIAID AIDSno	604039	8.873
NIAID AIDSno	604040	8.780
NIAID AIDSno	604041	8.380
NIAID AIDSno	604042	8.312
NIAID AIDSno	604043	8.294
NIAID AIDSno	604044	8.290
NIAID AIDSno	604045	8.266
NIAID AIDSno	604046	7.503
NIAID AIDSno	604047	8.236
NIAID AIDSno	604048	7.530
NIAID AIDSno	604049	8.161
NIAID AIDSno	604050	8.159
NIAID AIDSno	604051	8.154
NIAID AIDSno	604052	8.121

NIAID AIDSno	604053	8.018
NIAID AIDSno	604054	8.000
NIAID AIDSno	604055	7.973
NIAID AIDSno	604056	7.969
NIAID AIDSno	604057	7.952
NIAID AIDSno	604058	7.907
NIAID AIDSno	604059	7.881
NIAID AIDSno	604060	7.832
NIAID AIDSno	604061	7.666
NIAID AIDSno	604062	7.589
NIAID AIDSno	604063	7.550
NIAID AIDSno	604064	7.492
NIAID AIDSno	604065	7.265
NIAID AIDSno	604066	7.007
NIAID AIDSno	604067	8.073
NIAID AIDSno	604068	7.866
NIAID AIDSno	604069	7.210
NIAID AIDSno	604070	7.173
NIAID AIDSno	604071	7.031
NIAID AIDSno	604072	7.011
NIAID AIDSno	604073	7.779
NIAID AIDSno	604074	7.743
NIAID AIDSno	604075	7.718
NIAID AIDSno	604076	7.691
NIAID AIDSno	604077	7.591
NIAID AIDSno	604078	7.503
NIAID AIDSno	604079	7.466
NIAID AIDSno	604080	7.265
NIAID AIDSno	604081	7.232
NIAID AIDSno	604082	7.091
NIAID AIDSno	604083	7.976
NIAID AIDSno	604084	7.132
NIAID AIDSno	604085	7.094
NIAID AIDSno	604162	7.745
NIAID AIDSno	604164	7.959
NIAID AIDSno	604165	8.097
NIAID AIDSno	604166	8.000
NIAID AIDSno	604167	8.222
NIAID AIDSno	604168	8.398
NIAID AIDSno	604169	8.699
NIAID AIDSno	604170	7.921
NIAID AIDSno	604171	7.509
NIAID AIDSno	604172	7.469
NIAID AIDSno	604173	8.155
NIAID AIDSno	604174	7.678

NIAID AIDSno	604175	8.155
NIAID AIDSno	604177	7.174
NIAID AIDSno	604178	7.009
NIAID AIDSno	604180	7.721
NIAID AIDSno	604181	7.699
NIAID AIDSno	604182	7.602
NIAID AIDSno	604183	7.620
NIAID AIDSno	604184	7.041
NIAID AIDSno	604185	7.337
NIAID AIDSno	604186	7.222
NIAID AIDSno	604187	7.796
NIAID AIDSno	604188	7.658
NIAID AIDSno	604189	7.770
NIAID AIDSno	604190	7.569
NIAID AIDSno	604191	7.569
NIAID AIDSno	604192	8.046
NIAID AIDSno	604193	7.481
NIAID AIDSno	604194	7.013
NIAID AIDSno	604195	7.097
NIAID AIDSno	604196	7.367
NIAID AIDSno	604197	7.638
NIAID AIDSno	604198	7.745
NIAID AIDSno	604199	7.699
NIAID AIDSno	604200	8.046
NIAID AIDSno	604201	7.215
NIAID AIDSno	604202	7.638
NIAID AIDSno	604203	8.523
NIAID AIDSno	604204	7.268
NIAID AIDSno	604205	7.509
NIAID AIDSno	604206	7.337
NIAID AIDSno	604207	8.301
NIAID AIDSno	604208	7.699
NIAID AIDSno	604209	7.569
NIAID AIDSno	604210	7.495
NIAID AIDSno	604211	7.658
NIAID AIDSno	604212	7.854
NIAID AIDSno	604213	7.824
NIAID AIDSno	604214	8.188
NIAID AIDSno	604216	6.218
NIAID AIDSno	604217	6.604
NIAID AIDSno	604218	6.721
NIAID AIDSno	604219	6.274
NIAID AIDSno	604220	7.000
NIAID AIDSno	604567	8.770
NIAID AIDSno	604568	10.770

NIAID AIDSno	604569	10.260
NIAID AIDSno	604570	9.602
NIAID AIDSno	604901	7.795
NIAID AIDSno	604902	7.740
NIAID AIDSno	604903	7.652
NIAID AIDSno	604904	7.510
NIAID AIDSno	604905	7.367
NIAID AIDSno	604906	7.334
NIAID AIDSno	604907	7.281
NIAID AIDSno	604908	7.263
NIAID AIDSno	604909	7.165
NIAID AIDSno	604910	7.040
NIAID AIDSno	604911	7.036
NIAID AIDSno	604912	7.027
NIAID AIDSno	604913	8.175
NIAID AIDSno	604914	8.134
NIAID AIDSno	604915	8.040
NIAID AIDSno	604916	8.040
NIAID AIDSno	604917	7.988
NIAID AIDSno	604918	7.985
NIAID AIDSno	604919	7.937
NIAID AIDSno	604921	7.930
NIAID AIDSno	604922	7.896
NIAID AIDSno	604923	7.890
NIAID AIDSno	604924	7.888
NIAID AIDSno	604925	7.880
NIAID AIDSno	604926	7.861
NIAID AIDSno	604927	7.857
NIAID AIDSno	604928	7.772
NIAID AIDSno	604929	7.759
NIAID AIDSno	604930	7.751
NIAID AIDSno	604931	7.748
NIAID AIDSno	604932	7.721
NIAID AIDSno	604933	7.499
NIAID AIDSno	604934	7.704
NIAID AIDSno	604935	7.701
NIAID AIDSno	604936	7.688
NIAID AIDSno	604937	7.664
NIAID AIDSno	604938	7.629
NIAID AIDSno	604939	7.623
NIAID AIDSno	604940	7.606
NIAID AIDSno	604941	7.587
NIAID AIDSno	604943	7.577
NIAID AIDSno	604944	7.544
NIAID AIDSno	604945	7.452

NIAID AIDSno	604946	7.534
NIAID AIDSno	604947	7.518
NIAID AIDSno	604948	7.508
NIAID AIDSno	604949	7.478
NIAID AIDSno	604950	7.402
NIAID AIDSno	604952	7.346
NIAID AIDSno	604953	7.342
NIAID AIDSno	604954	7.316
NIAID AIDSno	604955	7.313
NIAID AIDSno	604957	7.276
NIAID AIDSno	604958	7.250
NIAID AIDSno	604959	7.230
NIAID AIDSno	604960	7.198
NIAID AIDSno	604961	7.160
NIAID AIDSno	604962	7.115
NIAID AIDSno	604963	7.112
NIAID AIDSno	604964	7.079
NIAID AIDSno	604965	7.061
NIAID AIDSno	604966	7.046
NIAID AIDSno	604967	7.043
NIAID AIDSno	604968	7.021
NIAID AIDSno	604969	7.015
NIAID AIDSno	604970	7.013
NIAID AIDSno	604972	7.004
NIAID AIDSno	604973	8.087
NIAID AIDSno	604974	8.022
NIAID AIDSno	604975	7.958
NIAID AIDSno	604976	7.942
NIAID AIDSno	604977	7.504
NIAID AIDSno	604979	7.396
NIAID AIDSno	604980	7.363
NIAID AIDSno	604981	7.270
NIAID AIDSno	604982	7.863
NIAID AIDSno	604983	7.812
NIAID AIDSno	604984	7.643
NIAID AIDSno	604985	7.360
NIAID AIDSno	604986	7.298
NIAID AIDSno	604987	7.032
NIAID AIDSno	604988	8.268
NIAID AIDSno	604989	8.112
NIAID AIDSno	604990	7.811
NIAID AIDSno	604991	7.768
NIAID AIDSno	604992	7.723
NIAID AIDSno	604993	7.609
NIAID AIDSno	604994	7.467

NIAID AIDSno	604995	7.275
NIAID AIDSno	604996	7.487
NIAID AIDSno	604997	8.051

7.3.Reverse transcriptase set

Source	ID	pIC50
ChEMBL MOLREGNO	1803	5.398
ChEMBL MOLREGNO	4344	7.284
ChEMBL MOLREGNO	4345	6.762
ChEMBL MOLREGNO	16626	7.155
ChEMBL MOLREGNO	19573	6.960
ChEMBL MOLREGNO	26590	4.836
ChEMBL MOLREGNO	27307	8.222
ChEMBL MOLREGNO	31429	7.602
ChEMBL MOLREGNO	32116	6.069
ChEMBL MOLREGNO	37777	7.385
ChEMBL MOLREGNO	41249	6.016
ChEMBL MOLREGNO	41281	7.638
ChEMBL MOLREGNO	41282	6.139
ChEMBL MOLREGNO	41742	5.600
ChEMBL MOLREGNO	41797	7.337
ChEMBL MOLREGNO	41809	6.967
ChEMBL MOLREGNO	41854	6.629
ChEMBL MOLREGNO	41855	6.097
ChEMBL MOLREGNO	41856	6.408
ChEMBL MOLREGNO	41865	6.733
ChEMBL	41885	6.889

MOLREGNO		
ChEMBL MOLREGNO	41892	6.224
ChEMBL MOLREGNO	42234	6.714
ChEMBL MOLREGNO	42262	6.374
ChEMBL MOLREGNO	42266	6.365
ChEMBL MOLREGNO	42303	6.762
ChEMBL MOLREGNO	42761	5.481
ChEMBL MOLREGNO	42875	6.821
ChEMBL MOLREGNO	46252	4.000
ChEMBL MOLREGNO	47562	5.757
ChEMBL MOLREGNO	47575	4.480
ChEMBL MOLREGNO	47598	5.569
ChEMBL MOLREGNO	47920	4.347
ChEMBL MOLREGNO	47921	4.648
ChEMBL MOLREGNO	47982	5.131
ChEMBL MOLREGNO	48077	4.312
ChEMBL MOLREGNO	48131	5.092
ChEMBL MOLREGNO	48807	4.527
ChEMBL MOLREGNO	49198	4.726
ChEMBL MOLREGNO	49286	5.215
ChEMBL MOLREGNO	49287	5.013
ChEMBL MOLREGNO	49787	5.319
ChEMBL MOLREGNO	49830	6.747
ChEMBL MOLREGNO	52468	5.081
ChEMBL MOLREGNO	52482	4.432

ChEMBL MOLREGNO	52483	5.886
ChEMBL MOLREGNO	52515	5.357
ChEMBL MOLREGNO	52550	5.921
ChEMBL MOLREGNO	52742	5.367
ChEMBL MOLREGNO	52743	6.000
ChEMBL MOLREGNO	52776	5.699
ChEMBL MOLREGNO	52953	4.569
ChEMBL MOLREGNO	52957	5.252
ChEMBL MOLREGNO	52958	6.119
ChEMBL MOLREGNO	52993	5.658
ChEMBL MOLREGNO	53132	5.260
ChEMBL MOLREGNO	53135	7.699
ChEMBL MOLREGNO	53136	5.409
ChEMBL MOLREGNO	53269	5.602
ChEMBL MOLREGNO	53270	4.469
ChEMBL MOLREGNO	53271	5.921
ChEMBL MOLREGNO	53308	5.745
ChEMBL MOLREGNO	53386	6.301
ChEMBL MOLREGNO	53665	8.523
ChEMBL MOLREGNO	53747	6.046
ChEMBL MOLREGNO	53748	6.699
ChEMBL MOLREGNO	53796	6.460
ChEMBL MOLREGNO	53840	7.495
ChEMBL MOLREGNO	53887	5.886
ChEMBL	53917	5.854

MOLREGNO		
ChEMBL MOLREGNO	54219	7.602
ChEMBL MOLREGNO	54296	6.981
ChEMBL MOLREGNO	54297	8.699
ChEMBL MOLREGNO	54298	8.382
ChEMBL MOLREGNO	54355	8.810
ChEMBL MOLREGNO	54356	8.097
ChEMBL MOLREGNO	54357	8.398
ChEMBL MOLREGNO	54358	8.097
ChEMBL MOLREGNO	54396	8.523
ChEMBL MOLREGNO	54404	6.600
ChEMBL MOLREGNO	54405	8.155
ChEMBL MOLREGNO	54406	8.855
ChEMBL MOLREGNO	54407	8.699
ChEMBL MOLREGNO	55235	6.347
ChEMBL MOLREGNO	55236	5.538
ChEMBL MOLREGNO	55238	7.022
ChEMBL MOLREGNO	55239	6.640
ChEMBL MOLREGNO	55247	6.357
ChEMBL MOLREGNO	55248	5.721
ChEMBL MOLREGNO	55286	6.119
ChEMBL MOLREGNO	55509	6.966
ChEMBL MOLREGNO	55604	5.903
ChEMBL MOLREGNO	55605	5.523
ChEMBL MOLREGNO	55606	6.071

ChEMBL MOLREGNO	55649	6.959
ChEMBL MOLREGNO	56377	4.815
ChEMBL MOLREGNO	56378	4.959
ChEMBL MOLREGNO	56379	6.347
ChEMBL MOLREGNO	56988	6.960
ChEMBL MOLREGNO	56989	6.460
ChEMBL MOLREGNO	57978	6.398
ChEMBL MOLREGNO	58528	6.523
ChEMBL MOLREGNO	58570	6.301
ChEMBL MOLREGNO	58587	6.699
ChEMBL MOLREGNO	58601	5.824
ChEMBL MOLREGNO	58603	5.638
ChEMBL MOLREGNO	58690	5.854
ChEMBL MOLREGNO	58968	7.301
ChEMBL MOLREGNO	59002	5.678
ChEMBL MOLREGNO	59255	6.301
ChEMBL MOLREGNO	59256	6.222
ChEMBL MOLREGNO	59291	6.523
ChEMBL MOLREGNO	60009	5.237
ChEMBL MOLREGNO	60013	6.222
ChEMBL MOLREGNO	60560	6.155
ChEMBL MOLREGNO	60893	5.155
ChEMBL MOLREGNO	61022	6.155
ChEMBL MOLREGNO	61023	5.194
ChEMBL	61058	5.252

MOLREGNO		
ChEMBL MOLREGNO	61640	8.222
ChEMBL MOLREGNO	64121	6.406
ChEMBL MOLREGNO	64122	6.664
ChEMBL MOLREGNO	64149	5.959
ChEMBL MOLREGNO	64684	5.107
ChEMBL MOLREGNO	64708	6.611
ChEMBL MOLREGNO	64709	5.561
ChEMBL MOLREGNO	64742	6.372
ChEMBL MOLREGNO	65160	5.660
ChEMBL MOLREGNO	65270	5.756
ChEMBL MOLREGNO	65354	6.807
ChEMBL MOLREGNO	66414	6.126
ChEMBL MOLREGNO	66415	5.886
ChEMBL MOLREGNO	66555	5.194
ChEMBL MOLREGNO	72225	6.221
ChEMBL MOLREGNO	72255	5.740
ChEMBL MOLREGNO	72256	5.743
ChEMBL MOLREGNO	72296	6.747
ChEMBL MOLREGNO	72305	6.469
ChEMBL MOLREGNO	72371	6.772
ChEMBL MOLREGNO	72372	6.967
ChEMBL MOLREGNO	72373	7.137
ChEMBL MOLREGNO	72472	6.580
ChEMBL MOLREGNO	72473	6.886

ChEMBL MOLREGNO	72474	6.917
ChEMBL MOLREGNO	72475	6.502
ChEMBL MOLREGNO	72584	5.942
ChEMBL MOLREGNO	72612	6.173
ChEMBL MOLREGNO	72797	6.747
ChEMBL MOLREGNO	72813	6.910
ChEMBL MOLREGNO	72827	6.738
ChEMBL MOLREGNO	72987	6.963
ChEMBL MOLREGNO	72988	6.187
ChEMBL MOLREGNO	72989	6.394
ChEMBL MOLREGNO	72990	6.483
ChEMBL MOLREGNO	73042	6.907
ChEMBL MOLREGNO	73147	6.796
ChEMBL MOLREGNO	73183	6.896
ChEMBL MOLREGNO	73185	6.298
ChEMBL MOLREGNO	73186	6.833
ChEMBL MOLREGNO	73899	6.796
ChEMBL MOLREGNO	74121	5.604
ChEMBL MOLREGNO	74276	5.531
ChEMBL MOLREGNO	74413	4.889
ChEMBL MOLREGNO	74444	4.352
ChEMBL MOLREGNO	74624	4.471
ChEMBL MOLREGNO	74806	4.203
ChEMBL MOLREGNO	74930	5.660
ChEMBL	74932	5.554

MOLREGNO		
ChEMBL MOLREGNO	74963	5.204
ChEMBL MOLREGNO	75027	4.648
ChEMBL MOLREGNO	75029	4.410
ChEMBL MOLREGNO	75063	4.138
ChEMBL MOLREGNO	76430	7.523
ChEMBL MOLREGNO	77351	5.547
ChEMBL MOLREGNO	77800	5.538
ChEMBL MOLREGNO	78268	5.650
ChEMBL MOLREGNO	79138	5.455
ChEMBL MOLREGNO	84743	6.523
ChEMBL MOLREGNO	86168	6.398
ChEMBL MOLREGNO	86225	6.194
ChEMBL MOLREGNO	86226	7.367
ChEMBL MOLREGNO	86245	5.658
ChEMBL MOLREGNO	86246	6.469
ChEMBL MOLREGNO	86248	6.553
ChEMBL MOLREGNO	86265	7.699
ChEMBL MOLREGNO	86267	6.046
ChEMBL MOLREGNO	86320	6.174
ChEMBL MOLREGNO	86321	6.783
ChEMBL MOLREGNO	86345	6.678
ChEMBL MOLREGNO	86360	6.585
ChEMBL MOLREGNO	86365	7.301
ChEMBL MOLREGNO	86383	7.143

ChEMBL MOLREGNO	86442	7.244
ChEMBL MOLREGNO	86474	7.201
ChEMBL MOLREGNO	86695	7.509
ChEMBL MOLREGNO	86714	6.620
ChEMBL MOLREGNO	86715	6.886
ChEMBL MOLREGNO	86716	7.523
ChEMBL MOLREGNO	86825	7.046
ChEMBL MOLREGNO	86842	7.222
ChEMBL MOLREGNO	86861	7.721
ChEMBL MOLREGNO	86862	6.879
ChEMBL MOLREGNO	86866	6.398
ChEMBL MOLREGNO	86871	7.658
ChEMBL MOLREGNO	86905	6.492
ChEMBL MOLREGNO	86914	6.000
ChEMBL MOLREGNO	86936	6.602
ChEMBL MOLREGNO	86946	7.638
ChEMBL MOLREGNO	86947	7.260
ChEMBL MOLREGNO	86950	6.333
ChEMBL MOLREGNO	86951	6.252
ChEMBL MOLREGNO	86970	7.149
ChEMBL MOLREGNO	86978	6.745
ChEMBL MOLREGNO	87025	7.538
ChEMBL MOLREGNO	87063	7.569
ChEMBL MOLREGNO	87064	7.337
ChEMBL	87065	7.347

MOLREGNO		
ChEMBL MOLREGNO	87067	6.222
ChEMBL MOLREGNO	87090	6.839
ChEMBL MOLREGNO	87104	6.426
ChEMBL MOLREGNO	87139	6.465
ChEMBL MOLREGNO	89638	5.328
ChEMBL MOLREGNO	89860	5.194
ChEMBL MOLREGNO	90022	4.569
ChEMBL MOLREGNO	90032	4.745
ChEMBL MOLREGNO	90040	4.530
ChEMBL MOLREGNO	90047	5.602
ChEMBL MOLREGNO	90048	4.658
ChEMBL MOLREGNO	90050	4.770
ChEMBL MOLREGNO	90382	6.618
ChEMBL MOLREGNO	90383	6.359
ChEMBL MOLREGNO	90413	5.804
ChEMBL MOLREGNO	90414	7.000
ChEMBL MOLREGNO	90424	4.796
ChEMBL MOLREGNO	90438	5.887
ChEMBL MOLREGNO	90439	5.751
ChEMBL MOLREGNO	90443	4.249
ChEMBL MOLREGNO	90447	6.357
ChEMBL MOLREGNO	90467	5.335
ChEMBL MOLREGNO	90471	5.978
ChEMBL MOLREGNO	90473	5.843

ChEMBL MOLREGNO	90474	5.837
ChEMBL MOLREGNO	90503	7.745
ChEMBL MOLREGNO	90510	4.608
ChEMBL MOLREGNO	90516	6.046
ChEMBL MOLREGNO	90567	5.584
ChEMBL MOLREGNO	90568	4.530
ChEMBL MOLREGNO	90691	5.992
ChEMBL MOLREGNO	90741	5.796
ChEMBL MOLREGNO	90779	4.445
ChEMBL MOLREGNO	90808	6.863
ChEMBL MOLREGNO	91234	6.889
ChEMBL MOLREGNO	91340	5.367
ChEMBL MOLREGNO	91358	6.886
ChEMBL MOLREGNO	91360	6.385
ChEMBL MOLREGNO	91951	4.866
ChEMBL MOLREGNO	91952	5.237
ChEMBL MOLREGNO	93808	5.999
ChEMBL MOLREGNO	93844	6.420
ChEMBL MOLREGNO	96201	6.907
ChEMBL MOLREGNO	96242	7.229
ChEMBL MOLREGNO	96245	7.268
ChEMBL MOLREGNO	96266	7.131
ChEMBL MOLREGNO	96420	7.041
ChEMBL MOLREGNO	96509	6.625
ChEMBL	96510	6.551

MOLREGNO		
ChEMBL MOLREGNO	96530	6.845
ChEMBL MOLREGNO	96535	6.558
ChEMBL MOLREGNO	96554	6.889
ChEMBL MOLREGNO	96556	6.742
ChEMBL MOLREGNO	96561	6.780
ChEMBL MOLREGNO	96563	6.959
ChEMBL MOLREGNO	96594	6.660
ChEMBL MOLREGNO	96596	7.409
ChEMBL MOLREGNO	96623	7.108
ChEMBL MOLREGNO	96624	7.208
ChEMBL MOLREGNO	96883	7.143
ChEMBL MOLREGNO	96885	7.167
ChEMBL MOLREGNO	96886	6.397
ChEMBL MOLREGNO	97070	7.301
ChEMBL MOLREGNO	97461	7.097
ChEMBL MOLREGNO	97462	6.991
ChEMBL MOLREGNO	97463	6.900
ChEMBL MOLREGNO	97912	7.131
ChEMBL MOLREGNO	99746	5.796
ChEMBL MOLREGNO	100213	5.921
ChEMBL MOLREGNO	100243	5.854
ChEMBL MOLREGNO	100414	5.921
ChEMBL MOLREGNO	100558	4.886
ChEMBL MOLREGNO	101758	4.959

ChEMBL MOLREGNO	101855	5.495
ChEMBL MOLREGNO	101857	4.000
ChEMBL MOLREGNO	101858	5.444
ChEMBL MOLREGNO	101921	4.018
ChEMBL MOLREGNO	101922	4.796
ChEMBL MOLREGNO	101923	4.027
ChEMBL MOLREGNO	102013	4.500
ChEMBL MOLREGNO	102045	5.252
ChEMBL MOLREGNO	102046	4.585
ChEMBL MOLREGNO	102389	4.201
ChEMBL MOLREGNO	102616	5.352
ChEMBL MOLREGNO	106017	6.613
ChEMBL MOLREGNO	106139	7.155
ChEMBL MOLREGNO	106411	6.194
ChEMBL MOLREGNO	106412	4.299
ChEMBL MOLREGNO	106413	4.939
ChEMBL MOLREGNO	106567	7.268
ChEMBL MOLREGNO	106602	7.046
ChEMBL MOLREGNO	106603	6.914
ChEMBL MOLREGNO	106605	6.857
ChEMBL MOLREGNO	106638	6.367
ChEMBL MOLREGNO	106666	6.842
ChEMBL MOLREGNO	107199	4.833
ChEMBL MOLREGNO	108375	4.301
ChEMBL	108782	6.523

MOLREGNO		
ChEMBL MOLREGNO	108805	7.398
ChEMBL MOLREGNO	111689	6.222
ChEMBL MOLREGNO	113067	6.770
ChEMBL MOLREGNO	113100	4.569
ChEMBL MOLREGNO	113162	6.538
ChEMBL MOLREGNO	113174	7.398
ChEMBL MOLREGNO	113198	7.208
ChEMBL MOLREGNO	113206	7.444
ChEMBL MOLREGNO	113207	6.377
ChEMBL MOLREGNO	113643	6.444
ChEMBL MOLREGNO	113673	6.367
ChEMBL MOLREGNO	113704	5.347
ChEMBL MOLREGNO	113718	6.638
ChEMBL MOLREGNO	113719	6.420
ChEMBL MOLREGNO	113733	6.398
ChEMBL MOLREGNO	113759	5.959
ChEMBL MOLREGNO	113791	6.000
ChEMBL MOLREGNO	113805	6.051
ChEMBL MOLREGNO	113843	5.862
ChEMBL MOLREGNO	113866	5.678
ChEMBL MOLREGNO	113868	6.721
ChEMBL MOLREGNO	113894	6.301
ChEMBL MOLREGNO	113911	6.699
ChEMBL MOLREGNO	113951	6.387

ChEMBL MOLREGNO	114022	7.420
ChEMBL MOLREGNO	114116	6.229
ChEMBL MOLREGNO	114148	6.071
ChEMBL MOLREGNO	114156	6.187
ChEMBL MOLREGNO	114189	6.620
ChEMBL MOLREGNO	114232	5.097
ChEMBL MOLREGNO	114235	6.638
ChEMBL MOLREGNO	114278	5.119
ChEMBL MOLREGNO	114284	6.161
ChEMBL MOLREGNO	114308	6.187
ChEMBL MOLREGNO	114314	6.237
ChEMBL MOLREGNO	114316	5.538
ChEMBL MOLREGNO	114356	6.620
ChEMBL MOLREGNO	114395	4.796
ChEMBL MOLREGNO	114397	5.180
ChEMBL MOLREGNO	114398	6.854
ChEMBL MOLREGNO	114439	6.071
ChEMBL MOLREGNO	114486	5.721
ChEMBL MOLREGNO	114531	6.456
ChEMBL MOLREGNO	114638	6.420
ChEMBL MOLREGNO	114654	5.854
ChEMBL MOLREGNO	114698	5.046
ChEMBL MOLREGNO	114722	5.481
ChEMBL MOLREGNO	114723	6.854
ChEMBL	123969	4.910

MOLREGNO		
ChEMBL MOLREGNO	128210	7.357
ChEMBL MOLREGNO	128677	6.569
ChEMBL MOLREGNO	128678	5.824
ChEMBL MOLREGNO	128789	6.151
ChEMBL MOLREGNO	128792	7.553
ChEMBL MOLREGNO	128793	8.509
ChEMBL MOLREGNO	128823	7.502
ChEMBL MOLREGNO	128825	6.886
ChEMBL MOLREGNO	128861	6.699
ChEMBL MOLREGNO	129312	6.886
ChEMBL MOLREGNO	129342	6.884
ChEMBL MOLREGNO	129343	6.476
ChEMBL MOLREGNO	129420	7.708
ChEMBL MOLREGNO	129431	7.963
ChEMBL MOLREGNO	129440	7.752
ChEMBL MOLREGNO	129471	7.967
ChEMBL MOLREGNO	129473	7.893
ChEMBL MOLREGNO	129509	8.036
ChEMBL MOLREGNO	130095	6.347
ChEMBL MOLREGNO	130097	7.190
ChEMBL MOLREGNO	132877	7.143
ChEMBL MOLREGNO	133299	7.509
ChEMBL MOLREGNO	135295	5.342
ChEMBL MOLREGNO	135296	4.896

ChEMBL MOLREGNO	135335	6.855
ChEMBL MOLREGNO	135336	5.456
ChEMBL MOLREGNO	135444	5.201
ChEMBL MOLREGNO	135445	6.046
ChEMBL MOLREGNO	135508	5.658
ChEMBL MOLREGNO	135509	4.824
ChEMBL MOLREGNO	135519	5.432
ChEMBL MOLREGNO	135534	4.398
ChEMBL MOLREGNO	135535	4.122
ChEMBL MOLREGNO	135536	4.585
ChEMBL MOLREGNO	135540	5.000
ChEMBL MOLREGNO	135542	7.097
ChEMBL MOLREGNO	135799	5.533
ChEMBL MOLREGNO	135801	5.365
ChEMBL MOLREGNO	135815	6.261
ChEMBL MOLREGNO	135860	5.398
ChEMBL MOLREGNO	135895	5.833
ChEMBL MOLREGNO	135896	5.000
ChEMBL MOLREGNO	135897	6.114
ChEMBL MOLREGNO	135910	7.436
ChEMBL MOLREGNO	135936	5.903
ChEMBL MOLREGNO	135937	7.699
ChEMBL MOLREGNO	135938	7.721
ChEMBL MOLREGNO	135955	5.349
ChEMBL	135957	5.097

MOLREGNO		
ChEMBL MOLREGNO	135960	5.478
ChEMBL MOLREGNO	135962	8.523
ChEMBL MOLREGNO	135979	4.267
ChEMBL MOLREGNO	136008	5.125
ChEMBL MOLREGNO	136009	4.450
ChEMBL MOLREGNO	136027	5.706
ChEMBL MOLREGNO	136045	5.870
ChEMBL MOLREGNO	136046	5.577
ChEMBL MOLREGNO	136067	5.398
ChEMBL MOLREGNO	136120	5.337
ChEMBL MOLREGNO	136121	5.180
ChEMBL MOLREGNO	136131	6.409
ChEMBL MOLREGNO	136155	4.638
ChEMBL MOLREGNO	136157	6.000
ChEMBL MOLREGNO	136238	4.425
ChEMBL MOLREGNO	136241	4.292
ChEMBL MOLREGNO	136265	5.462
ChEMBL MOLREGNO	136266	4.770
ChEMBL MOLREGNO	136304	5.839
ChEMBL MOLREGNO	136335	5.215
ChEMBL MOLREGNO	136337	5.742
ChEMBL MOLREGNO	136346	4.229
ChEMBL MOLREGNO	136389	5.854
ChEMBL MOLREGNO	136390	6.658

ChEMBL MOLREGNO	136399	5.678
ChEMBL MOLREGNO	136400	5.959
ChEMBL MOLREGNO	136508	5.000
ChEMBL MOLREGNO	136515	6.456
ChEMBL MOLREGNO	136536	6.102
ChEMBL MOLREGNO	136558	5.991
ChEMBL MOLREGNO	136560	6.097
ChEMBL MOLREGNO	136561	5.432
ChEMBL MOLREGNO	136570	5.839
ChEMBL MOLREGNO	136571	5.658
ChEMBL MOLREGNO	136589	7.481
ChEMBL MOLREGNO	136590	7.495
ChEMBL MOLREGNO	136591	6.292
ChEMBL MOLREGNO	136604	7.699
ChEMBL MOLREGNO	136607	7.412
ChEMBL MOLREGNO	136624	5.886
ChEMBL MOLREGNO	136647	7.097
ChEMBL MOLREGNO	136660	6.229
ChEMBL MOLREGNO	136673	6.046
ChEMBL MOLREGNO	136699	6.013
ChEMBL MOLREGNO	136700	5.783
ChEMBL MOLREGNO	136796	4.553
ChEMBL MOLREGNO	136824	5.387
ChEMBL MOLREGNO	136829	5.233
ChEMBL	136845	5.638

MOLREGNO		
ChEMBL MOLREGNO	136846	5.678
ChEMBL MOLREGNO	136847	7.456
ChEMBL MOLREGNO	136849	8.046
ChEMBL MOLREGNO	136878	7.469
ChEMBL MOLREGNO	136879	7.721
ChEMBL MOLREGNO	136926	5.237
ChEMBL MOLREGNO	136967	7.398
ChEMBL MOLREGNO	136987	6.699
ChEMBL MOLREGNO	136991	7.187
ChEMBL MOLREGNO	136994	7.041
ChEMBL MOLREGNO	137005	7.301
ChEMBL MOLREGNO	137006	7.301
ChEMBL MOLREGNO	137021	6.398
ChEMBL MOLREGNO	137040	7.669
ChEMBL MOLREGNO	137068	8.097
ChEMBL MOLREGNO	137164	5.824
ChEMBL MOLREGNO	137165	5.854
ChEMBL MOLREGNO	137174	7.638
ChEMBL MOLREGNO	137175	7.495
ChEMBL MOLREGNO	137192	6.276
ChEMBL MOLREGNO	137209	5.420
ChEMBL MOLREGNO	137210	5.810
ChEMBL MOLREGNO	137244	6.577
ChEMBL MOLREGNO	137262	5.638

ChEMBL MOLREGNO	137329	4.553
ChEMBL MOLREGNO	137330	4.903
ChEMBL MOLREGNO	137331	5.161
ChEMBL MOLREGNO	137332	5.337
ChEMBL MOLREGNO	137334	6.167
ChEMBL MOLREGNO	137351	7.363
ChEMBL MOLREGNO	137355	7.155
ChEMBL MOLREGNO	137379	7.155
ChEMBL MOLREGNO	137506	7.301
ChEMBL MOLREGNO	137530	7.301
ChEMBL MOLREGNO	137531	7.097
ChEMBL MOLREGNO	142387	4.975
ChEMBL MOLREGNO	142388	4.854
ChEMBL MOLREGNO	142390	5.053
ChEMBL MOLREGNO	142391	4.456
ChEMBL MOLREGNO	142531	4.824
ChEMBL MOLREGNO	143000	5.323
ChEMBL MOLREGNO	143019	5.071
ChEMBL MOLREGNO	143080	4.721
ChEMBL MOLREGNO	143121	4.921
ChEMBL MOLREGNO	143142	4.180
ChEMBL MOLREGNO	143205	4.678
ChEMBL MOLREGNO	143262	4.824
ChEMBL MOLREGNO	143298	4.721
ChEMBL	145220	5.000

MOLREGNO		
ChEMBL MOLREGNO	145760	8.523
ChEMBL MOLREGNO	145763	8.398
ChEMBL MOLREGNO	145960	5.000
ChEMBL MOLREGNO	145988	4.000
ChEMBL MOLREGNO	146018	8.222
ChEMBL MOLREGNO	146071	7.328
ChEMBL MOLREGNO	146099	7.097
ChEMBL MOLREGNO	146100	7.268
ChEMBL MOLREGNO	146132	6.301
ChEMBL MOLREGNO	146154	8.523
ChEMBL MOLREGNO	146188	5.301
ChEMBL MOLREGNO	146194	9.000
ChEMBL MOLREGNO	146227	6.745
ChEMBL MOLREGNO	146228	6.398
ChEMBL MOLREGNO	146229	8.523
ChEMBL MOLREGNO	146286	8.097
ChEMBL MOLREGNO	146300	8.000
ChEMBL MOLREGNO	146637	4.824
ChEMBL MOLREGNO	146682	5.301
ChEMBL MOLREGNO	146748	4.602
ChEMBL MOLREGNO	146788	6.602
ChEMBL MOLREGNO	149229	6.398
ChEMBL MOLREGNO	149253	5.866
ChEMBL MOLREGNO	149265	5.398

ChEMBL MOLREGNO	154246	6.321
ChEMBL MOLREGNO	154495	6.355
ChEMBL MOLREGNO	154534	6.097
ChEMBL MOLREGNO	154568	5.719
ChEMBL MOLREGNO	154571	6.721
ChEMBL MOLREGNO	154572	6.099
ChEMBL MOLREGNO	154573	6.876
ChEMBL MOLREGNO	154616	5.708
ChEMBL MOLREGNO	154617	7.076
ChEMBL MOLREGNO	154619	5.903
ChEMBL MOLREGNO	154644	6.883
ChEMBL MOLREGNO	154650	6.088
ChEMBL MOLREGNO	161890	7.260
ChEMBL MOLREGNO	161891	6.912
ChEMBL MOLREGNO	161918	7.000
ChEMBL MOLREGNO	162143	6.432
ChEMBL MOLREGNO	162172	5.194
ChEMBL MOLREGNO	162225	6.629
ChEMBL MOLREGNO	162243	7.936
ChEMBL MOLREGNO	162245	7.596
ChEMBL MOLREGNO	164328	6.509
ChEMBL MOLREGNO	164330	6.481
ChEMBL MOLREGNO	164331	7.000
ChEMBL MOLREGNO	164333	5.523
ChEMBL	164334	6.602

MOLREGNO		
ChEMBL MOLREGNO	164336	6.921
ChEMBL MOLREGNO	164388	7.301
ChEMBL MOLREGNO	164389	6.921
ChEMBL MOLREGNO	164536	7.046
ChEMBL MOLREGNO	164539	6.208
ChEMBL MOLREGNO	164544	7.301
ChEMBL MOLREGNO	164577	6.678
ChEMBL MOLREGNO	164754	7.699
ChEMBL MOLREGNO	164855	6.721
ChEMBL MOLREGNO	164869	7.699
ChEMBL MOLREGNO	164884	6.943
ChEMBL MOLREGNO	164889	6.921
ChEMBL MOLREGNO	164893	7.046
ChEMBL MOLREGNO	164894	7.301
ChEMBL MOLREGNO	164898	6.481
ChEMBL MOLREGNO	164910	6.824
ChEMBL MOLREGNO	164931	7.097
ChEMBL MOLREGNO	164932	6.854
ChEMBL MOLREGNO	164944	6.796
ChEMBL MOLREGNO	164947	7.222
ChEMBL MOLREGNO	165009	7.301
ChEMBL MOLREGNO	165010	7.097
ChEMBL MOLREGNO	165011	6.337
ChEMBL MOLREGNO	165065	6.229

ChEMBL MOLREGNO	165066	6.921
ChEMBL MOLREGNO	165068	7.097
ChEMBL MOLREGNO	165091	6.959
ChEMBL MOLREGNO	165092	7.699
ChEMBL MOLREGNO	165112	6.638
ChEMBL MOLREGNO	165113	5.991
ChEMBL MOLREGNO	165114	7.222
ChEMBL MOLREGNO	165115	6.796
ChEMBL MOLREGNO	165128	6.367
ChEMBL MOLREGNO	165155	7.301
ChEMBL MOLREGNO	165181	6.523
ChEMBL MOLREGNO	165237	7.398
ChEMBL MOLREGNO	165271	7.222
ChEMBL MOLREGNO	165273	7.097
ChEMBL MOLREGNO	165299	6.125
ChEMBL MOLREGNO	165386	5.959
ChEMBL MOLREGNO	165387	5.824
ChEMBL MOLREGNO	165398	6.699
ChEMBL MOLREGNO	165460	7.301
ChEMBL MOLREGNO	165461	6.721
ChEMBL MOLREGNO	165490	7.222
ChEMBL MOLREGNO	165492	7.523
ChEMBL MOLREGNO	165493	7.155
ChEMBL MOLREGNO	165527	6.886
ChEMBL	165528	7.523

MOLREGNO		
ChEMBL MOLREGNO	165529	7.301
ChEMBL MOLREGNO	165530	6.886
ChEMBL MOLREGNO	165532	7.000
ChEMBL MOLREGNO	165608	6.097
ChEMBL MOLREGNO	165625	6.770
ChEMBL MOLREGNO	165633	7.046
ChEMBL MOLREGNO	165635	6.585
ChEMBL MOLREGNO	165660	6.638
ChEMBL MOLREGNO	165662	7.523
ChEMBL MOLREGNO	165702	6.678
ChEMBL MOLREGNO	165718	7.155
ChEMBL MOLREGNO	165719	7.699
ChEMBL MOLREGNO	165720	6.921
ChEMBL MOLREGNO	165721	7.000
ChEMBL MOLREGNO	165759	7.398
ChEMBL MOLREGNO	165782	7.097
ChEMBL MOLREGNO	165783	6.959
ChEMBL MOLREGNO	165798	7.155
ChEMBL MOLREGNO	165799	8.111
ChEMBL MOLREGNO	165800	7.523
ChEMBL MOLREGNO	165816	6.678
ChEMBL MOLREGNO	165817	7.000
ChEMBL MOLREGNO	165826	7.301
ChEMBL MOLREGNO	165843	7.398

ChEMBL MOLREGNO	165844	6.268
ChEMBL MOLREGNO	165850	7.523
ChEMBL MOLREGNO	165851	6.959
ChEMBL MOLREGNO	165958	6.553
ChEMBL MOLREGNO	165984	6.155
ChEMBL MOLREGNO	165991	5.921
ChEMBL MOLREGNO	166008	6.770
ChEMBL MOLREGNO	166015	5.745
ChEMBL MOLREGNO	166016	4.886
ChEMBL MOLREGNO	166018	5.131
ChEMBL MOLREGNO	166051	6.229
ChEMBL MOLREGNO	166068	6.678
ChEMBL MOLREGNO	166069	6.886
ChEMBL MOLREGNO	166071	7.000
ChEMBL MOLREGNO	166097	7.699
ChEMBL MOLREGNO	166224	8.046
ChEMBL MOLREGNO	166250	8.699
ChEMBL MOLREGNO	166255	6.222
ChEMBL MOLREGNO	166276	5.620
ChEMBL MOLREGNO	166315	5.824
ChEMBL MOLREGNO	166337	5.409
ChEMBL MOLREGNO	166359	7.699
ChEMBL MOLREGNO	166400	7.638
ChEMBL MOLREGNO	166475	6.855
ChEMBL	172855	5.091

MOLREGNO		
ChEMBL MOLREGNO	173628	5.870
ChEMBL MOLREGNO	173659	6.889
ChEMBL MOLREGNO	173685	5.710
ChEMBL MOLREGNO	173708	7.386
ChEMBL MOLREGNO	173743	5.415
ChEMBL MOLREGNO	173783	5.735
ChEMBL MOLREGNO	173790	5.569
ChEMBL MOLREGNO	173850	7.592
ChEMBL MOLREGNO	173886	7.358
ChEMBL MOLREGNO	173915	7.927
ChEMBL MOLREGNO	173994	7.778
ChEMBL MOLREGNO	174021	5.215
ChEMBL MOLREGNO	174022	6.213
ChEMBL MOLREGNO	174033	7.585
ChEMBL MOLREGNO	174234	6.726
ChEMBL MOLREGNO	174235	6.979
ChEMBL MOLREGNO	174238	6.738
ChEMBL MOLREGNO	174248	6.114
ChEMBL MOLREGNO	174249	7.444
ChEMBL MOLREGNO	174250	7.932
ChEMBL MOLREGNO	174251	7.614
ChEMBL MOLREGNO	176294	6.261
ChEMBL MOLREGNO	176304	5.222
ChEMBL MOLREGNO	176349	7.131

ChEMBL MOLREGNO	176397	4.004
ChEMBL MOLREGNO	176403	4.150
ChEMBL MOLREGNO	176404	4.527
ChEMBL MOLREGNO	176406	5.039
ChEMBL MOLREGNO	176435	5.081
ChEMBL MOLREGNO	176556	4.219
ChEMBL MOLREGNO	176730	7.108
ChEMBL MOLREGNO	176764	6.495
ChEMBL MOLREGNO	176765	7.347
ChEMBL MOLREGNO	176916	6.585
ChEMBL MOLREGNO	177099	5.851
ChEMBL MOLREGNO	177249	5.326
ChEMBL MOLREGNO	177285	7.337
ChEMBL MOLREGNO	177288	6.620
ChEMBL MOLREGNO	177361	6.387
ChEMBL MOLREGNO	177364	6.495
ChEMBL MOLREGNO	177392	7.523
ChEMBL MOLREGNO	177395	7.108
ChEMBL MOLREGNO	177436	5.156
ChEMBL MOLREGNO	177452	5.951
ChEMBL MOLREGNO	177454	6.174
ChEMBL MOLREGNO	177466	6.260
ChEMBL MOLREGNO	177469	6.745
ChEMBL MOLREGNO	177479	6.432
ChEMBL	177480	5.348

MOLREGNO		
ChEMBL MOLREGNO	177492	5.155
ChEMBL MOLREGNO	177574	6.620
ChEMBL MOLREGNO	181170	5.367
ChEMBL MOLREGNO	181213	5.367
ChEMBL MOLREGNO	181268	5.337
ChEMBL MOLREGNO	181279	5.337
ChEMBL MOLREGNO	181283	5.215
ChEMBL MOLREGNO	181418	5.602
ChEMBL MOLREGNO	181420	5.585
ChEMBL MOLREGNO	181714	5.834
ChEMBL MOLREGNO	181774	5.310
ChEMBL MOLREGNO	181776	5.027
ChEMBL MOLREGNO	181931	5.659
ChEMBL MOLREGNO	181962	5.602
ChEMBL MOLREGNO	181969	5.921
ChEMBL MOLREGNO	181992	5.784
ChEMBL MOLREGNO	181999	5.076
ChEMBL MOLREGNO	182000	5.538
ChEMBL MOLREGNO	182167	5.960
ChEMBL MOLREGNO	182168	5.658
ChEMBL MOLREGNO	182169	5.602
ChEMBL MOLREGNO	189579	5.921
ChEMBL MOLREGNO	189581	5.310
ChEMBL MOLREGNO	189651	6.097

ChEMBL MOLREGNO	189686	6.784
ChEMBL MOLREGNO	190029	6.155
ChEMBL MOLREGNO	203700	6.347
ChEMBL MOLREGNO	203819	6.420
ChEMBL MOLREGNO	204315	6.284
ChEMBL MOLREGNO	204319	6.854
ChEMBL MOLREGNO	204356	6.398
ChEMBL MOLREGNO	204639	6.638
ChEMBL MOLREGNO	204702	7.046
ChEMBL MOLREGNO	204712	6.620
ChEMBL MOLREGNO	204736	6.602
ChEMBL MOLREGNO	204743	4.959
ChEMBL MOLREGNO	204749	6.620
ChEMBL MOLREGNO	204750	6.319
ChEMBL MOLREGNO	204780	7.036
ChEMBL MOLREGNO	204805	6.745
ChEMBL MOLREGNO	204864	6.770
ChEMBL MOLREGNO	204865	6.155
ChEMBL MOLREGNO	204868	6.276
ChEMBL MOLREGNO	204900	5.991
ChEMBL MOLREGNO	204907	6.102
ChEMBL MOLREGNO	204909	7.155
ChEMBL MOLREGNO	204946	5.979
ChEMBL MOLREGNO	204953	6.886
ChEMBL	204966	6.979

MOLREGNO		
ChEMBL MOLREGNO	204991	6.824
ChEMBL MOLREGNO	205001	6.854
ChEMBL MOLREGNO	205002	6.658
ChEMBL MOLREGNO	205003	6.721
ChEMBL MOLREGNO	205021	6.352
ChEMBL MOLREGNO	205033	5.959
ChEMBL MOLREGNO	205137	6.456
ChEMBL MOLREGNO	205155	6.796
ChEMBL MOLREGNO	205169	6.420
ChEMBL MOLREGNO	205179	5.509
ChEMBL MOLREGNO	205205	6.523
ChEMBL MOLREGNO	205259	6.081
ChEMBL MOLREGNO	205311	6.137
ChEMBL MOLREGNO	205315	6.444
ChEMBL MOLREGNO	205316	6.131
ChEMBL MOLREGNO	205327	5.280
ChEMBL MOLREGNO	205352	6.523
ChEMBL MOLREGNO	205364	5.602
ChEMBL MOLREGNO	205377	6.987
ChEMBL MOLREGNO	205402	5.174
ChEMBL MOLREGNO	205403	6.638
ChEMBL MOLREGNO	205405	6.959
ChEMBL MOLREGNO	205410	6.184
ChEMBL MOLREGNO	205418	5.903

ChEMBL MOLREGNO	205433	7.097
ChEMBL MOLREGNO	205435	6.328
ChEMBL MOLREGNO	205438	7.187
ChEMBL MOLREGNO	205445	4.611
ChEMBL MOLREGNO	205446	6.357
ChEMBL MOLREGNO	205449	6.260
ChEMBL MOLREGNO	205451	7.000
ChEMBL MOLREGNO	205478	6.824
ChEMBL MOLREGNO	205499	7.237
ChEMBL MOLREGNO	205500	5.710
ChEMBL MOLREGNO	205508	6.585
ChEMBL MOLREGNO	205529	5.783
ChEMBL MOLREGNO	205532	5.523
ChEMBL MOLREGNO	205537	5.979
ChEMBL MOLREGNO	205539	6.553
ChEMBL MOLREGNO	205568	6.523
ChEMBL MOLREGNO	205576	5.177
ChEMBL MOLREGNO	205602	9.222
ChEMBL MOLREGNO	205605	5.469
ChEMBL MOLREGNO	205611	5.721
ChEMBL MOLREGNO	205616	6.939
ChEMBL MOLREGNO	205619	6.947
ChEMBL MOLREGNO	205620	7.367
ChEMBL MOLREGNO	205635	5.947
ChEMBL	205636	5.979

MOLREGNO		
ChEMBL MOLREGNO	205644	7.301
ChEMBL MOLREGNO	205645	6.569
ChEMBL MOLREGNO	205654	7.377
ChEMBL MOLREGNO	205665	7.638
ChEMBL MOLREGNO	205680	4.174
ChEMBL MOLREGNO	205683	6.481
ChEMBL MOLREGNO	205733	6.337
ChEMBL MOLREGNO	205751	6.310
ChEMBL MOLREGNO	205947	4.502
ChEMBL MOLREGNO	205948	5.757
ChEMBL MOLREGNO	205950	5.939
ChEMBL MOLREGNO	205951	6.222
ChEMBL MOLREGNO	205954	7.071
ChEMBL MOLREGNO	205955	6.337
ChEMBL MOLREGNO	205988	5.879
ChEMBL MOLREGNO	205997	6.119
ChEMBL MOLREGNO	206056	6.367
ChEMBL MOLREGNO	206065	7.222
ChEMBL MOLREGNO	206066	6.699
ChEMBL MOLREGNO	206098	6.553
ChEMBL MOLREGNO	206115	6.721
ChEMBL MOLREGNO	206116	5.545
ChEMBL MOLREGNO	206117	6.013
ChEMBL MOLREGNO	206139	6.475

ChEMBL MOLREGNO	206140	7.244
ChEMBL MOLREGNO	206172	5.699
ChEMBL MOLREGNO	206174	6.420
ChEMBL MOLREGNO	206190	4.854
ChEMBL MOLREGNO	206207	5.886
ChEMBL MOLREGNO	206212	6.469
ChEMBL MOLREGNO	206213	5.000
ChEMBL MOLREGNO	206231	5.697
ChEMBL MOLREGNO	206232	5.638
ChEMBL MOLREGNO	206239	6.252
ChEMBL MOLREGNO	206252	4.796
ChEMBL MOLREGNO	206256	5.022
ChEMBL MOLREGNO	206260	5.569
ChEMBL MOLREGNO	206267	4.658
ChEMBL MOLREGNO	206269	6.553
ChEMBL MOLREGNO	206289	6.009
ChEMBL MOLREGNO	206295	6.721
ChEMBL MOLREGNO	206313	5.602
ChEMBL MOLREGNO	206314	5.629
ChEMBL MOLREGNO	206316	5.903
ChEMBL MOLREGNO	206318	5.409
ChEMBL MOLREGNO	206370	6.745
ChEMBL MOLREGNO	206378	6.194
ChEMBL MOLREGNO	206379	7.000
ChEMBL	206380	5.469

MOLREGNO		
ChEMBL MOLREGNO	206417	5.585
ChEMBL MOLREGNO	212102	7.000
ChEMBL MOLREGNO	212277	7.699
ChEMBL MOLREGNO	212281	6.699
ChEMBL MOLREGNO	218140	5.000
ChEMBL MOLREGNO	218170	6.000
ChEMBL MOLREGNO	218173	6.742
ChEMBL MOLREGNO	218174	6.633
ChEMBL MOLREGNO	218645	6.445
ChEMBL MOLREGNO	218674	6.163
ChEMBL MOLREGNO	218710	6.602
ChEMBL MOLREGNO	218719	5.714
ChEMBL MOLREGNO	218720	5.622
ChEMBL MOLREGNO	218743	5.307
ChEMBL MOLREGNO	218790	5.354
ChEMBL MOLREGNO	218835	7.796
ChEMBL MOLREGNO	218864	5.148
ChEMBL MOLREGNO	218947	6.302
ChEMBL MOLREGNO	218989	5.248
ChEMBL MOLREGNO	219005	5.530
ChEMBL MOLREGNO	219007	4.184
ChEMBL MOLREGNO	219113	5.972
ChEMBL MOLREGNO	219173	4.155
ChEMBL MOLREGNO	219234	4.500

ChEMBL MOLREGNO	219270	5.024
ChEMBL MOLREGNO	219353	4.337
ChEMBL MOLREGNO	219373	6.143
ChEMBL MOLREGNO	219375	5.046
ChEMBL MOLREGNO	219414	6.569
ChEMBL MOLREGNO	219441	6.886
ChEMBL MOLREGNO	219569	6.770
ChEMBL MOLREGNO	219577	8.569
ChEMBL MOLREGNO	219650	8.456
ChEMBL MOLREGNO	219690	8.369
ChEMBL MOLREGNO	219739	7.009
ChEMBL MOLREGNO	219762	6.854
ChEMBL MOLREGNO	219837	6.387
ChEMBL MOLREGNO	219868	6.292
ChEMBL MOLREGNO	219925	6.854
ChEMBL MOLREGNO	219977	7.215
ChEMBL MOLREGNO	220075	6.650
ChEMBL MOLREGNO	220093	8.284
ChEMBL MOLREGNO	220231	8.276
ChEMBL MOLREGNO	220241	8.167
ChEMBL MOLREGNO	220285	7.796
ChEMBL MOLREGNO	220311	8.307
ChEMBL MOLREGNO	220319	6.870
ChEMBL MOLREGNO	221629	4.730
ChEMBL	223489	8.699

MOLREGNO		
ChEMBL MOLREGNO	223790	7.155
ChEMBL MOLREGNO	223909	8.523
ChEMBL MOLREGNO	224000	8.398
ChEMBL MOLREGNO	224099	8.699
ChEMBL MOLREGNO	224140	6.824
ChEMBL MOLREGNO	224141	8.523
ChEMBL MOLREGNO	224142	6.495
ChEMBL MOLREGNO	224195	7.328
ChEMBL MOLREGNO	224250	9.000
ChEMBL MOLREGNO	224271	7.678
ChEMBL MOLREGNO	224301	9.000
ChEMBL MOLREGNO	224398	8.155
ChEMBL MOLREGNO	224400	7.046
ChEMBL MOLREGNO	224416	8.097
ChEMBL MOLREGNO	224417	8.523
ChEMBL MOLREGNO	224418	6.699
ChEMBL MOLREGNO	224708	7.222
ChEMBL MOLREGNO	224887	8.301
ChEMBL MOLREGNO	224890	8.699
ChEMBL MOLREGNO	236165	7.000
ChEMBL MOLREGNO	236552	7.824
ChEMBL MOLREGNO	236950	6.398
ChEMBL MOLREGNO	237008	6.222
ChEMBL MOLREGNO	239796	6.337

ChEMBL MOLREGNO	239797	6.237
ChEMBL MOLREGNO	239806	7.000
ChEMBL MOLREGNO	239807	6.420
ChEMBL MOLREGNO	239869	5.658
ChEMBL MOLREGNO	239870	6.959
ChEMBL MOLREGNO	239889	7.046
ChEMBL MOLREGNO	239890	6.824
ChEMBL MOLREGNO	239891	6.398
ChEMBL MOLREGNO	239920	6.620
ChEMBL MOLREGNO	239925	6.523
ChEMBL MOLREGNO	239971	6.097
ChEMBL MOLREGNO	239973	6.310
ChEMBL MOLREGNO	240006	7.699
ChEMBL MOLREGNO	240007	7.699
ChEMBL MOLREGNO	240009	6.036
ChEMBL MOLREGNO	240042	8.000
ChEMBL MOLREGNO	240105	6.086
ChEMBL MOLREGNO	240107	6.638
ChEMBL MOLREGNO	240109	7.699
ChEMBL MOLREGNO	240113	6.377
ChEMBL MOLREGNO	240139	7.222
ChEMBL MOLREGNO	240140	6.658
ChEMBL MOLREGNO	240206	7.301
ChEMBL MOLREGNO	240213	5.987
ChEMBL	240214	5.987

MOLREGNO		
ChEMBL MOLREGNO	240224	5.921
ChEMBL MOLREGNO	240225	6.745
ChEMBL MOLREGNO	240229	6.000
ChEMBL MOLREGNO	240254	5.959
ChEMBL MOLREGNO	240259	6.824
ChEMBL MOLREGNO	240260	7.000
ChEMBL MOLREGNO	240284	7.155
ChEMBL MOLREGNO	240285	6.854
ChEMBL MOLREGNO	240302	6.721
ChEMBL MOLREGNO	240324	7.398
ChEMBL MOLREGNO	240325	6.328
ChEMBL MOLREGNO	240326	6.509
ChEMBL MOLREGNO	240330	6.155
ChEMBL MOLREGNO	240339	6.387
ChEMBL MOLREGNO	240346	5.614
ChEMBL MOLREGNO	240351	6.194
ChEMBL MOLREGNO	240353	6.959
ChEMBL MOLREGNO	240362	7.097
ChEMBL MOLREGNO	240383	6.071
ChEMBL MOLREGNO	240384	6.854
ChEMBL MOLREGNO	240395	6.921
ChEMBL MOLREGNO	240401	6.538
ChEMBL MOLREGNO	240419	6.377
ChEMBL MOLREGNO	240431	7.699

ChEMBL MOLREGNO	240434	6.469
ChEMBL MOLREGNO	240436	5.398
ChEMBL MOLREGNO	240449	6.824
ChEMBL MOLREGNO	240460	7.398
ChEMBL MOLREGNO	240461	7.523
ChEMBL MOLREGNO	240462	8.000
ChEMBL MOLREGNO	240470	7.301
ChEMBL MOLREGNO	240475	5.886
ChEMBL MOLREGNO	240480	7.046
ChEMBL MOLREGNO	240486	6.959
ChEMBL MOLREGNO	240487	7.000
ChEMBL MOLREGNO	240505	7.301
ChEMBL MOLREGNO	240518	6.824
ChEMBL MOLREGNO	240520	6.602
ChEMBL MOLREGNO	240535	7.523
ChEMBL MOLREGNO	240551	6.854
ChEMBL MOLREGNO	240552	6.745
ChEMBL MOLREGNO	240560	6.481
ChEMBL MOLREGNO	240564	5.708
ChEMBL MOLREGNO	240577	6.409
ChEMBL MOLREGNO	240578	6.886
ChEMBL MOLREGNO	240606	7.046
ChEMBL MOLREGNO	240613	5.432
ChEMBL MOLREGNO	240619	6.569
ChEMBL	240628	7.745

MOLREGNO		
ChEMBL MOLREGNO	240629	7.699
ChEMBL MOLREGNO	240631	7.398
ChEMBL MOLREGNO	240632	7.155
ChEMBL MOLREGNO	240633	5.854
ChEMBL MOLREGNO	240648	5.851
ChEMBL MOLREGNO	240649	5.516
ChEMBL MOLREGNO	240650	5.983
ChEMBL MOLREGNO	240661	7.155
ChEMBL MOLREGNO	240686	6.959
ChEMBL MOLREGNO	240687	6.699
ChEMBL MOLREGNO	240756	7.000
ChEMBL MOLREGNO	240793	6.409
ChEMBL MOLREGNO	240794	6.638
ChEMBL MOLREGNO	240812	7.046
ChEMBL MOLREGNO	240813	7.046
ChEMBL MOLREGNO	240820	6.721
ChEMBL MOLREGNO	240880	7.155
ChEMBL MOLREGNO	240881	7.398
ChEMBL MOLREGNO	240897	5.340
ChEMBL MOLREGNO	240912	7.699
ChEMBL MOLREGNO	240940	6.921
ChEMBL MOLREGNO	240986	4.347
ChEMBL MOLREGNO	240991	4.155
ChEMBL MOLREGNO	241012	5.991

ChEMBL MOLREGNO	241013	4.500
ChEMBL MOLREGNO	241044	5.343
ChEMBL MOLREGNO	241056	5.947
ChEMBL MOLREGNO	241409	5.092
ChEMBL MOLREGNO	241435	5.143
ChEMBL MOLREGNO	241647	5.721
ChEMBL MOLREGNO	241678	5.921
ChEMBL MOLREGNO	241679	6.187
ChEMBL MOLREGNO	241707	5.347
ChEMBL MOLREGNO	241736	6.347
ChEMBL MOLREGNO	241759	4.000
ChEMBL MOLREGNO	241826	4.998
ChEMBL MOLREGNO	241839	6.000
ChEMBL MOLREGNO	241901	5.658
ChEMBL MOLREGNO	242088	6.699
ChEMBL MOLREGNO	242635	6.699
ChEMBL MOLREGNO	242773	5.959
ChEMBL MOLREGNO	242798	7.523
ChEMBL MOLREGNO	243016	6.699
ChEMBL MOLREGNO	243051	5.721
ChEMBL MOLREGNO	248837	5.469
ChEMBL MOLREGNO	248839	5.495
ChEMBL MOLREGNO	248863	5.959
ChEMBL MOLREGNO	248870	5.699
ChEMBL	248892	5.319

MOLREGNO		
ChEMBL MOLREGNO	248917	5.745
ChEMBL MOLREGNO	248944	4.780
ChEMBL MOLREGNO	248975	5.678
ChEMBL MOLREGNO	249007	4.470
ChEMBL MOLREGNO	249092	5.699
ChEMBL MOLREGNO	249108	5.569
ChEMBL MOLREGNO	249110	5.658
ChEMBL MOLREGNO	249204	5.314
ChEMBL MOLREGNO	249205	4.701
ChEMBL MOLREGNO	249261	5.215
ChEMBL MOLREGNO	249302	5.596
ChEMBL MOLREGNO	249359	4.429
ChEMBL MOLREGNO	249369	5.854
ChEMBL MOLREGNO	249389	5.092
ChEMBL MOLREGNO	249487	5.721
ChEMBL MOLREGNO	249523	5.225
ChEMBL MOLREGNO	249551	4.016
ChEMBL MOLREGNO	249552	4.649
ChEMBL MOLREGNO	249573	6.347
ChEMBL MOLREGNO	249863	6.046
ChEMBL MOLREGNO	250111	5.398
ChEMBL MOLREGNO	250164	5.301
ChEMBL MOLREGNO	250259	6.569
ChEMBL MOLREGNO	250384	6.398

ChEMBL MOLREGNO	250666	4.373
ChEMBL MOLREGNO	251093	5.385
ChEMBL MOLREGNO	251200	5.385
ChEMBL MOLREGNO	251479	5.545
ChEMBL MOLREGNO	251551	4.247
ChEMBL MOLREGNO	251552	4.174
ChEMBL MOLREGNO	251759	4.212
ChEMBL MOLREGNO	256435	4.821
ChEMBL MOLREGNO	256607	5.149
ChEMBL MOLREGNO	256608	5.523
ChEMBL MOLREGNO	256621	5.237
ChEMBL MOLREGNO	256654	4.896
ChEMBL MOLREGNO	256685	4.688
ChEMBL MOLREGNO	256744	4.876
ChEMBL MOLREGNO	261005	5.703
ChEMBL MOLREGNO	261008	5.611
ChEMBL MOLREGNO	261537	5.023
ChEMBL MOLREGNO	261586	6.222
ChEMBL MOLREGNO	261590	6.301
ChEMBL MOLREGNO	261591	5.824
ChEMBL MOLREGNO	261670	5.939
ChEMBL MOLREGNO	261672	7.046
ChEMBL MOLREGNO	261694	6.699
ChEMBL MOLREGNO	261748	6.699
ChEMBL	261760	5.631

MOLREGNO		
ChEMBL MOLREGNO	261858	6.222
ChEMBL MOLREGNO	261889	6.523
ChEMBL MOLREGNO	261959	5.319
ChEMBL MOLREGNO	261977	5.939
ChEMBL MOLREGNO	261996	6.523
ChEMBL MOLREGNO	261998	6.097
ChEMBL MOLREGNO	262091	7.046
ChEMBL MOLREGNO	262131	6.398
ChEMBL MOLREGNO	262133	7.000
ChEMBL MOLREGNO	262169	5.824
ChEMBL MOLREGNO	262170	6.387
ChEMBL MOLREGNO	262176	5.540
ChEMBL MOLREGNO	262178	7.222
ChEMBL MOLREGNO	262201	5.071
ChEMBL MOLREGNO	262227	5.523
ChEMBL MOLREGNO	262305	5.796
ChEMBL MOLREGNO	262306	5.721
ChEMBL MOLREGNO	262307	6.523
ChEMBL MOLREGNO	262309	6.046
ChEMBL MOLREGNO	262310	6.398
ChEMBL MOLREGNO	262314	5.959
ChEMBL MOLREGNO	262316	6.398
ChEMBL MOLREGNO	262414	7.046
ChEMBL MOLREGNO	262515	7.155

ChEMBL MOLREGNO	262957	6.699
ChEMBL MOLREGNO	271781	5.060
ChEMBL MOLREGNO	271820	7.959
ChEMBL MOLREGNO	271845	8.377
ChEMBL MOLREGNO	271876	7.921
ChEMBL MOLREGNO	271877	8.161
ChEMBL MOLREGNO	271881	8.658
ChEMBL MOLREGNO	271930	8.222
ChEMBL MOLREGNO	271931	7.585
ChEMBL MOLREGNO	272292	6.081
ChEMBL MOLREGNO	272382	8.097
ChEMBL MOLREGNO	272387	7.569
ChEMBL MOLREGNO	272488	8.155
ChEMBL MOLREGNO	275314	5.699
ChEMBL MOLREGNO	275913	5.638
ChEMBL MOLREGNO	275942	5.602
ChEMBL MOLREGNO	284890	6.398
ChEMBL MOLREGNO	287077	6.046
ChEMBL MOLREGNO	287078	6.000
ChEMBL MOLREGNO	287656	7.155
ChEMBL MOLREGNO	288733	7.456
ChEMBL MOLREGNO	289489	5.611
ChEMBL MOLREGNO	289584	6.415
ChEMBL MOLREGNO	289585	7.523
ChEMBL	289713	7.284

MOLREGNO		
ChEMBL MOLREGNO	289871	5.268
ChEMBL MOLREGNO	289938	4.301
ChEMBL MOLREGNO	290005	6.719
ChEMBL MOLREGNO	290006	4.000
ChEMBL MOLREGNO	290010	7.523
ChEMBL MOLREGNO	290159	7.538
ChEMBL MOLREGNO	290299	7.013
ChEMBL MOLREGNO	290300	6.752
ChEMBL MOLREGNO	290301	5.733
ChEMBL MOLREGNO	290395	7.237
ChEMBL MOLREGNO	290430	7.469
ChEMBL MOLREGNO	290469	7.367
ChEMBL MOLREGNO	290522	7.319
ChEMBL MOLREGNO	290663	7.081
ChEMBL MOLREGNO	290664	6.770
ChEMBL MOLREGNO	290706	7.056
ChEMBL MOLREGNO	290776	5.678
ChEMBL MOLREGNO	290907	7.824
ChEMBL MOLREGNO	290967	7.398
ChEMBL MOLREGNO	291013	4.796
ChEMBL MOLREGNO	291025	4.347
ChEMBL MOLREGNO	291066	7.959
ChEMBL MOLREGNO	291067	6.000
ChEMBL MOLREGNO	291132	7.523

ChEMBL MOLREGNO	291205	7.398
ChEMBL MOLREGNO	291331	7.620
ChEMBL MOLREGNO	292337	7.886
ChEMBL MOLREGNO	292396	5.076
ChEMBL MOLREGNO	292460	5.376
ChEMBL MOLREGNO	292461	7.824
ChEMBL MOLREGNO	292462	7.432
ChEMBL MOLREGNO	292620	5.903
ChEMBL MOLREGNO	292640	5.097
ChEMBL MOLREGNO	292641	6.509
ChEMBL MOLREGNO	292757	5.469
ChEMBL MOLREGNO	292920	5.959
ChEMBL MOLREGNO	293201	7.481
ChEMBL MOLREGNO	293202	4.870
ChEMBL MOLREGNO	293229	6.688
ChEMBL MOLREGNO	293359	5.854
ChEMBL MOLREGNO	293465	7.208
ChEMBL MOLREGNO	293633	5.770
ChEMBL MOLREGNO	293643	6.355
ChEMBL MOLREGNO	293644	6.764
ChEMBL MOLREGNO	293797	5.420
ChEMBL MOLREGNO	316154	6.149
ChEMBL MOLREGNO	316201	6.201
ChEMBL MOLREGNO	316228	6.022
ChEMBL	316448	6.206

MOLREGNO		
ChEMBL MOLREGNO	318263	7.824
ChEMBL MOLREGNO	318268	7.395
ChEMBL MOLREGNO	318380	7.721
ChEMBL MOLREGNO	318566	8.000
ChEMBL MOLREGNO	318767	7.770
ChEMBL MOLREGNO	318810	7.824
ChEMBL MOLREGNO	318838	7.745
ChEMBL MOLREGNO	318864	7.201
ChEMBL MOLREGNO	318987	7.022
ChEMBL MOLREGNO	318994	7.658
ChEMBL MOLREGNO	319050	7.721
ChEMBL MOLREGNO	319172	7.854
ChEMBL MOLREGNO	319178	7.638
ChEMBL MOLREGNO	321699	4.896
ChEMBL MOLREGNO	321700	5.284
ChEMBL MOLREGNO	321868	5.125
ChEMBL MOLREGNO	321869	4.790
ChEMBL MOLREGNO	322039	4.222
ChEMBL MOLREGNO	322040	4.699
ChEMBL MOLREGNO	322073	4.301
ChEMBL MOLREGNO	322542	5.770
ChEMBL MOLREGNO	322724	7.046
ChEMBL MOLREGNO	329161	6.000
ChEMBL MOLREGNO	329167	6.886

ChEMBL MOLREGNO	329173	4.010
ChEMBL MOLREGNO	329210	4.257
ChEMBL MOLREGNO	329220	6.172
ChEMBL MOLREGNO	329227	6.125
ChEMBL MOLREGNO	329228	5.120
ChEMBL MOLREGNO	329232	6.046
ChEMBL MOLREGNO	329233	5.244
ChEMBL MOLREGNO	329235	7.301
ChEMBL MOLREGNO	329842	4.493
ChEMBL MOLREGNO	329843	8.000
ChEMBL MOLREGNO	329942	4.951
ChEMBL MOLREGNO	329943	5.161
ChEMBL MOLREGNO	329949	4.496
ChEMBL MOLREGNO	329950	5.602
ChEMBL MOLREGNO	329989	4.654
ChEMBL MOLREGNO	330002	6.301
ChEMBL MOLREGNO	346766	8.155
ChEMBL MOLREGNO	346767	7.509
ChEMBL MOLREGNO	346780	6.699
ChEMBL MOLREGNO	346781	7.638
ChEMBL MOLREGNO	346789	8.398
ChEMBL MOLREGNO	347078	8.398
ChEMBL MOLREGNO	347079	7.658
ChEMBL MOLREGNO	347086	8.000
ChEMBL	347087	6.638

MOLREGNO		
ChEMBL MOLREGNO	347094	8.523
ChEMBL MOLREGNO	347095	7.921
ChEMBL MOLREGNO	347143	8.398
ChEMBL MOLREGNO	347246	6.783
ChEMBL MOLREGNO	347253	5.902
ChEMBL MOLREGNO	347254	7.268
ChEMBL MOLREGNO	347260	7.745
ChEMBL MOLREGNO	347261	8.523
ChEMBL MOLREGNO	347425	8.301
ChEMBL MOLREGNO	347426	8.301
ChEMBL MOLREGNO	347433	8.523
ChEMBL MOLREGNO	347434	8.301
ChEMBL MOLREGNO	347445	7.886
ChEMBL MOLREGNO	347446	8.398
ChEMBL MOLREGNO	347479	7.260
ChEMBL MOLREGNO	347486	8.523
ChEMBL MOLREGNO	347496	8.046
ChEMBL MOLREGNO	347503	8.523
ChEMBL MOLREGNO	350630	5.699
ChEMBL MOLREGNO	354550	5.307
ChEMBL MOLREGNO	354557	5.509
ChEMBL MOLREGNO	354811	5.092
ChEMBL MOLREGNO	354889	4.824
ChEMBL MOLREGNO	354890	5.936

ChEMBL MOLREGNO	356067	5.337
ChEMBL MOLREGNO	356068	5.167
ChEMBL MOLREGNO	356069	4.919
ChEMBL MOLREGNO	356070	4.553
ChEMBL MOLREGNO	356235	4.796
ChEMBL MOLREGNO	356236	5.319
ChEMBL MOLREGNO	356237	5.174
ChEMBL MOLREGNO	356238	5.357
ChEMBL MOLREGNO	356274	5.699
ChEMBL MOLREGNO	356275	4.305
ChEMBL MOLREGNO	356302	5.006
ChEMBL MOLREGNO	356303	5.119
ChEMBL MOLREGNO	356340	7.699
ChEMBL MOLREGNO	356400	5.143
ChEMBL MOLREGNO	356401	5.268
ChEMBL MOLREGNO	356402	5.420
ChEMBL MOLREGNO	356413	5.066
ChEMBL MOLREGNO	356459	5.092
ChEMBL MOLREGNO	356460	5.328
ChEMBL MOLREGNO	356461	4.886
ChEMBL MOLREGNO	356462	5.398
ChEMBL MOLREGNO	356481	6.155
ChEMBL MOLREGNO	356512	5.921
ChEMBL MOLREGNO	356513	4.308
ChEMBL	374719	7.611

MOLREGNO		
ChEMBL MOLREGNO	390877	5.270
ChEMBL MOLREGNO	396794	5.544
ChEMBL MOLREGNO	403087	7.151
ChEMBL MOLREGNO	409515	7.000
ChEMBL MOLREGNO	419801	7.222
ChEMBL MOLREGNO	419802	6.824
ChEMBL MOLREGNO	419803	6.000
ChEMBL MOLREGNO	419804	7.398
ChEMBL MOLREGNO	419805	6.824
ChEMBL MOLREGNO	419806	6.456
ChEMBL MOLREGNO	419807	7.097
ChEMBL MOLREGNO	419808	6.886
ChEMBL MOLREGNO	419809	5.959
ChEMBL MOLREGNO	419810	7.222
ChEMBL MOLREGNO	419811	6.699
ChEMBL MOLREGNO	419812	6.398
ChEMBL MOLREGNO	419813	7.046
ChEMBL MOLREGNO	419814	6.699
ChEMBL MOLREGNO	419815	7.523
ChEMBL MOLREGNO	419816	8.000
ChEMBL MOLREGNO	419817	8.000
ChEMBL MOLREGNO	419818	8.000
ChEMBL MOLREGNO	419819	8.222
ChEMBL MOLREGNO	419824	7.000

ChEMBL MOLREGNO	419825	6.222
ChEMBL MOLREGNO	419827	6.456
ChEMBL MOLREGNO	419829	6.699
ChEMBL MOLREGNO	419831	5.602
ChEMBL MOLREGNO	419833	6.260
ChEMBL MOLREGNO	419834	6.699
ChEMBL MOLREGNO	419837	7.523
ChEMBL MOLREGNO	419838	8.222
ChEMBL MOLREGNO	419840	8.523
ChEMBL MOLREGNO	419841	8.347
ChEMBL MOLREGNO	419842	8.222
ChEMBL MOLREGNO	419843	7.301
ChEMBL MOLREGNO	419844	8.301
ChEMBL MOLREGNO	419846	7.347
ChEMBL MOLREGNO	419848	8.097
ChEMBL MOLREGNO	419849	8.155
ChEMBL MOLREGNO	419850	8.222
ChEMBL MOLREGNO	419851	8.398
ChEMBL MOLREGNO	419852	8.398
ChEMBL MOLREGNO	419853	7.921
ChEMBL MOLREGNO	419854	8.222
ChEMBL MOLREGNO	419855	8.097
ChEMBL MOLREGNO	419869	8.301
ChEMBL MOLREGNO	419871	8.301
ChEMBL	419873	6.745

MOLREGNO		
ChEMBL MOLREGNO	419876	8.301
ChEMBL MOLREGNO	419880	9.000
ChEMBL MOLREGNO	419881	8.301
ChEMBL MOLREGNO	419889	7.678
ChEMBL MOLREGNO	677187	6.194
ChEMBL MOLREGNO	677188	7.097
ChEMBL MOLREGNO	1164974	5.358
ChEMBL MOLREGNO	1164977	5.943
ChEMBL MOLREGNO	1165015	4.662
ChEMBL MOLREGNO	1165021	4.971
ChEMBL MOLREGNO	1165023	5.268
ChEMBL MOLREGNO	1165024	5.827
ChEMBL MOLREGNO	1165025	5.180
ChEMBL MOLREGNO	1165026	5.460
ChEMBL MOLREGNO	1165027	4.948
ChEMBL MOLREGNO	1165028	4.840
ChEMBL MOLREGNO	1165029	5.358
ChEMBL MOLREGNO	1165030	4.914
ChEMBL MOLREGNO	1165031	5.335
ChEMBL MOLREGNO	1165034	5.124
ChEMBL MOLREGNO	1386760	6.845
ChEMBL MOLREGNO	1386761	6.367
ChEMBL MOLREGNO	1386762	6.355
ChEMBL MOLREGNO	1386772	6.824

ChEMBL MOLREGNO	1387273	5.754
ChEMBL MOLREGNO	1387274	6.000
ChEMBL MOLREGNO	1387276	6.553
ChEMBL MOLREGNO	1387277	5.886
ChEMBL MOLREGNO	1387279	6.538
ChEMBL MOLREGNO	1387285	6.119
ChEMBL MOLREGNO	1387286	6.260
ChEMBL MOLREGNO	1387287	6.721
ChEMBL MOLREGNO	1632205	5.201
ChEMBL MOLREGNO	1632209	5.432
ChEMBL MOLREGNO	1632210	5.420
ChEMBL MOLREGNO	1632211	4.959
ChEMBL MOLREGNO	1633051	5.523
ChEMBL MOLREGNO	1633055	5.538
ChEMBL MOLREGNO	1633058	5.180
ChEMBL MOLREGNO	1633059	5.509
ChEMBL MOLREGNO	1633083	5.509
ChEMBL MOLREGNO	1633084	5.456
ChEMBL MOLREGNO	1633088	4.523
ChEMBL MOLREGNO	1633089	5.347
ChEMBL MOLREGNO	1633090	4.886
ChEMBL MOLREGNO	1972870	4.046
Integrity Entry Number	314034	7.398
Integrity Entry Number	294230	5.921
Integrity Entry	294231	5.854

Number		
Integrity Entry Number	294232	5.796
Integrity Entry Number	294233	5.921
Integrity Entry Number	294234	4.886
Integrity Entry Number	772680	8.699
Integrity Entry Number	772683	7.926
Integrity Entry Number	1000088	6.131
Integrity Entry Number	1000089	7.523
Integrity Entry Number	1000090	7.699
Integrity Entry Number	1000091	8.000
Integrity Entry Number	1001157	5.796
Integrity Entry Number	1001158	5.807
Integrity Entry Number	1002290	7.585
Integrity Entry Number	1002303	7.125
Integrity Entry Number	1002309	7.009
Integrity Entry Number	1013724	7.041
Integrity Entry Number	1013725	7.292
Integrity Entry Number	1013726	6.804
Integrity Entry Number	1024513	3.944
Integrity Entry Number	1031761	5.903
Integrity Entry Number	1031762	6.282
Integrity Entry Number	1031763	5.676
Integrity Entry Number	1031765	6.032
Integrity Entry Number	1032501	5.171
Integrity Entry Number	1036720	7.252

Integrity Entry Number	1037778	7.310
Integrity Entry Number	1037779	7.174
Integrity Entry Number	1037781	7.119
Integrity Entry Number	1039742	7.222
Integrity Entry Number	1039743	7.301
Integrity Entry Number	1039745	7.398
Integrity Entry Number	1041188	5.018
Integrity Entry Number	1053683	8.000
Integrity Entry Number	1053689	6.347
Integrity Entry Number	1053690	6.469
Integrity Entry Number	1053692	6.959
Integrity Entry Number	1053693	6.432
Integrity Entry Number	119547	7.398
Integrity Entry Number	218583	5.959
Integrity Entry Number	228466	6.097
Integrity Entry Number	166014	5.000
Integrity Entry Number	228467	5.921
Integrity Entry Number	175220	7.056
Integrity Entry Number	175221	7.921
Integrity Entry Number	175223	8.569
Integrity Entry Number	177225	8.569
Integrity Entry Number	180973	7.886
Integrity Entry Number	221280	8.699
Integrity Entry Number	224608	8.097
Integrity Entry	225433	3.819

Number		
Integrity Entry Number	229334	6.000
Integrity Entry Number	229335	6.000
Integrity Entry Number	229353	6.131
Integrity Entry Number	233314	7.000
Integrity Entry Number	233315	7.097
Integrity Entry Number	250925	8.301
Integrity Entry Number	251236	7.509
Integrity Entry Number	259164	6.699
Integrity Entry Number	269046	6.699
Integrity Entry Number	269048	7.222
Integrity Entry Number	270092	5.509
Integrity Entry Number	274078	6.883
Integrity Entry Number	274079	7.076
Integrity Entry Number	282469	7.699
Integrity Entry Number	285555	7.174
Integrity Entry Number	285758	9.000
Integrity Entry Number	286316	4.839
Integrity Entry Number	294324	6.523
Integrity Entry Number	294325	6.301
Integrity Entry Number	294326	6.301
Integrity Entry Number	303702	5.092
Integrity Entry Number	304494	7.523
Integrity Entry Number	304495	8.301
Integrity Entry Number	307670	5.538

Integrity Entry Number	307671	5.650
Integrity Entry Number	308555	5.000
Integrity Entry Number	308556	4.620
Integrity Entry Number	308557	4.143
Integrity Entry Number	311769	3.860
Integrity Entry Number	311771	3.684
Integrity Entry Number	311772	4.471
Integrity Entry Number	313496	6.237
Integrity Entry Number	313498	5.930
Integrity Entry Number	314521	7.921
Integrity Entry Number	331516	3.850
Integrity Entry Number	333073	5.602
Integrity Entry Number	333075	5.699
Integrity Entry Number	344891	5.678
Integrity Entry Number	344892	6.060
Integrity Entry Number	345337	7.333
Integrity Entry Number	350614	8.155
Integrity Entry Number	355167	5.523
Integrity Entry Number	366484	8.523
Integrity Entry Number	371636	5.432
Integrity Entry Number	376872	7.770
Integrity Entry Number	379107	6.041
Integrity Entry Number	379108	6.060
Integrity Entry Number	379109	6.013
Integrity Entry	387815	6.620

Number		
Integrity Entry Number	387816	6.420
Integrity Entry Number	394295	7.770
Integrity Entry Number	394298	7.796
Integrity Entry Number	394299	8.046
Integrity Entry Number	394881	6.076
Integrity Entry Number	394883	6.125
Integrity Entry Number	394884	4.257
Integrity Entry Number	394885	6.046
Integrity Entry Number	398235	5.544
Integrity Entry Number	704872	5.367
Integrity Entry Number	400693	7.046
Integrity Entry Number	402337	6.560
Integrity Entry Number	402338	6.247
Integrity Entry Number	408304	4.564
Integrity Entry Number	408305	5.886
Integrity Entry Number	408306	5.817
Integrity Entry Number	408307	5.673
Integrity Entry Number	408309	6.000
Integrity Entry Number	408310	5.921
Integrity Entry Number	408311	5.770
Integrity Entry Number	408313	5.699
Integrity Entry Number	408320	5.398
Integrity Entry Number	408324	4.886
Integrity Entry Number	408326	6.523

Integrity Entry Number	408327	4.686
Integrity Entry Number	408328	4.721
Integrity Entry Number	408330	5.387
Integrity Entry Number	408331	5.174
Integrity Entry Number	408332	5.509
Integrity Entry Number	408333	5.481
Integrity Entry Number	408334	5.328
Integrity Entry Number	408335	5.237
Integrity Entry Number	408337	5.081
Integrity Entry Number	408338	4.943
Integrity Entry Number	412439	7.420
Integrity Entry Number	412444	6.452
Integrity Entry Number	412446	6.833
Integrity Entry Number	412449	6.870
Integrity Entry Number	412645	5.509
Integrity Entry Number	412646	6.252
Integrity Entry Number	416718	7.585
Integrity Entry Number	418366	6.962
Integrity Entry Number	418370	6.020
Integrity Entry Number	418372	6.354
Integrity Entry Number	418375	6.015
Integrity Entry Number	418379	5.903
Integrity Entry Number	429796	8.222
Integrity Entry Number	429798	7.583
Integrity Entry	429799	7.558

Number		
Integrity Entry Number	433876	5.120
Integrity Entry Number	433879	5.553
Integrity Entry Number	433880	5.921
Integrity Entry Number	433881	4.308
Integrity Entry Number	435182	7.889
Integrity Entry Number	435183	7.341
Integrity Entry Number	439201	5.006
Integrity Entry Number	445536	7.699
Integrity Entry Number	452369	8.301
Integrity Entry Number	453105	5.456
Integrity Entry Number	454820	5.245
Integrity Entry Number	468132	6.000
Integrity Entry Number	472011	9.000
Integrity Entry Number	472013	8.481
Integrity Entry Number	472016	8.432
Integrity Entry Number	472087	9.046
Integrity Entry Number	472557	8.420
Integrity Entry Number	472558	8.620
Integrity Entry Number	472559	8.509
Integrity Entry Number	474005	6.086
Integrity Entry Number	474008	5.457
Integrity Entry Number	474009	5.293
Integrity Entry Number	476948	7.108
Integrity Entry Number	476955	7.301

Integrity Entry Number	631294	7.650
Integrity Entry Number	631298	7.551
Integrity Entry Number	631302	7.590
Integrity Entry Number	631304	7.572
Integrity Entry Number	631306	7.554
Integrity Entry Number	631309	8.066
Integrity Entry Number	632329	9.398
Integrity Entry Number	632335	9.620
Integrity Entry Number	632337	9.239
Integrity Entry Number	632342	6.284
Integrity Entry Number	632345	9.046
Integrity Entry Number	634082	6.694
Integrity Entry Number	634083	7.092
Integrity Entry Number	634094	8.387
Integrity Entry Number	634095	7.432
Integrity Entry Number	634097	8.161
Integrity Entry Number	634099	7.788
Integrity Entry Number	634258	7.959
Integrity Entry Number	634259	7.495
Integrity Entry Number	637105	7.149
Integrity Entry Number	637107	7.377
Integrity Entry Number	637109	7.638
Integrity Entry Number	639921	6.699
Integrity Entry Number	641267	7.000
Integrity Entry	642063	8.301

Number		
Integrity Entry Number	643392	7.613
Integrity Entry Number	643395	7.959
Integrity Entry Number	647448	8.466
Integrity Entry Number	647449	8.046
Integrity Entry Number	649266	8.420
Integrity Entry Number	649267	7.721
Integrity Entry Number	649297	8.301
Integrity Entry Number	649298	8.237
Integrity Entry Number	649300	8.155
Integrity Entry Number	649302	8.292
Integrity Entry Number	652069	7.548
Integrity Entry Number	652799	8.721
Integrity Entry Number	652800	8.620
Integrity Entry Number	658136	6.041
Integrity Entry Number	660611	9.523
Integrity Entry Number	660612	9.301
Integrity Entry Number	660613	9.301
Integrity Entry Number	660614	9.097
Integrity Entry Number	664577	8.310
Integrity Entry Number	666925	6.585
Integrity Entry Number	666926	6.638
Integrity Entry Number	669309	6.131
Integrity Entry Number	672127	8.523
Integrity Entry Number	672860	7.602

Integrity Entry Number	672946	5.585
Integrity Entry Number	672947	5.420
Integrity Entry Number	675377	6.456
Integrity Entry Number	675380	6.921
Integrity Entry Number	675381	7.187
Integrity Entry Number	675383	6.444
Integrity Entry Number	676205	4.387
Integrity Entry Number	694798	8.222
Integrity Entry Number	695000	5.985
Integrity Entry Number	696552	5.088
Integrity Entry Number	696839	5.533
Integrity Entry Number	696841	5.690
Integrity Entry Number	696842	5.620
Integrity Entry Number	696843	6.745
Integrity Entry Number	699514	2.959
Integrity Entry Number	699517	2.699
Integrity Entry Number	702346	6.602
Integrity Entry Number	702444	7.347
Integrity Entry Number	702447	7.046
Integrity Entry Number	711141	8.222
Integrity Entry Number	711144	8.097
Integrity Entry Number	712753	7.569
Integrity Entry Number	713229	5.979
Integrity Entry Number	714270	7.260
Integrity Entry	714272	8.097

Number		
Integrity Entry Number	722072	6.572
Integrity Entry Number	729337	4.939
Integrity Entry Number	735646	7.745
Integrity Entry Number	735647	7.854
Integrity Entry Number	735648	7.770
Integrity Entry Number	735649	7.678
Integrity Entry Number	736619	6.801
Integrity Entry Number	738243	5.398
Integrity Entry Number	738945	6.301
Integrity Entry Number	738947	6.000
Integrity Entry Number	739138	5.420
Integrity Entry Number	739140	5.456
Integrity Entry Number	739987	7.847
Integrity Entry Number	228751	7.655
Integrity Entry Number	741324	5.410
Integrity Entry Number	746681	6.215
Integrity Entry Number	746683	7.222
Integrity Entry Number	746686	7.222
Integrity Entry Number	748273	7.678
Integrity Entry Number	748340	5.460
Integrity Entry Number	748349	4.513
Integrity Entry Number	750307	8.796
Integrity Entry Number	754068	4.000
Integrity Entry Number	764369	6.456

Integrity Entry Number	764372	6.310
Integrity Entry Number	764374	6.319
Integrity Entry Number	764376	6.229
Integrity Entry Number	766212	6.119
Integrity Entry Number	766215	6.233
Integrity Entry Number	769733	7.678
Integrity Entry Number	769735	7.367
Integrity Entry Number	769737	6.959
Integrity Entry Number	773309	8.469
Integrity Entry Number	774147	8.000
Integrity Entry Number	775596	5.602
Integrity Entry Number	776111	7.244
Integrity Entry Number	776112	8.222
Integrity Entry Number	778266	5.752
Integrity Entry Number	778267	5.706
Integrity Entry Number	786242	5.153
Integrity Entry Number	786244	4.802
Integrity Entry Number	786247	5.033
Integrity Entry Number	795141	5.759
Integrity Entry Number	796931	5.523
Integrity Entry Number	798247	4.301
Integrity Entry Number	801302	8.943
Integrity Entry Number	801304	8.688
Integrity Entry Number	805137	6.585
Integrity Entry	805140	6.509

Number		
Integrity Entry Number	805141	6.509
Integrity Entry Number	807319	5.815
Integrity Entry Number	807324	5.268
Integrity Entry Number	807326	4.499
Integrity Entry Number	807378	3.790
Integrity Entry Number	807379	4.100
Integrity Entry Number	807713	7.319
Integrity Entry Number	807715	7.538
Integrity Entry Number	810026	8.237
Integrity Entry Number	812590	5.498
Integrity Entry Number	812712	5.253
Integrity Entry Number	815034	8.699
Integrity Entry Number	815471	8.377
Integrity Entry Number	818288	8.215
Integrity Entry Number	819925	8.125
Integrity Entry Number	822276	5.921
Integrity Entry Number	829550	6.796
Integrity Entry Number	829551	7.143
Integrity Entry Number	829552	7.398
Integrity Entry Number	829553	6.721
Integrity Entry Number	833018	5.086
Integrity Entry Number	833019	5.292
Integrity Entry Number	834147	5.824
Integrity Entry Number	836045	4.481

Integrity Entry Number	837510	8.367
Integrity Entry Number	841002	7.301
Integrity Entry Number	841003	6.095
Integrity Entry Number	841007	7.301
Integrity Entry Number	841011	7.301
Integrity Entry Number	841015	7.301
Integrity Entry Number	841016	7.301
Integrity Entry Number	841017	7.301
Integrity Entry Number	841020	7.301
Integrity Entry Number	841021	7.301
Integrity Entry Number	844610	7.097
Integrity Entry Number	844612	6.959
Integrity Entry Number	844613	6.585
Integrity Entry Number	845178	5.284
Integrity Entry Number	846320	6.638
Integrity Entry Number	846321	6.770
Integrity Entry Number	849473	5.646
Integrity Entry Number	849523	5.699
Integrity Entry Number	849525	6.796
Integrity Entry Number	849526	6.201
Integrity Entry Number	849528	6.409
Integrity Entry Number	850676	5.161
Integrity Entry Number	850678	5.071
Integrity Entry Number	850681	4.983
Integrity Entry	850951	5.027

Number		
Integrity Entry Number	850953	4.613
Integrity Entry Number	850955	4.155
Integrity Entry Number	850958	6.653
Integrity Entry Number	852704	8.523
Integrity Entry Number	852706	8.097
Integrity Entry Number	852708	8.097
Integrity Entry Number	852709	8.155
Integrity Entry Number	852711	8.398
Integrity Entry Number	852712	8.222
Integrity Entry Number	852714	8.398
Integrity Entry Number	852715	8.222
Integrity Entry Number	855995	7.355
Integrity Entry Number	855998	5.678
Integrity Entry Number	855999	5.284
Integrity Entry Number	856327	8.425
Integrity Entry Number	856335	8.427
Integrity Entry Number	856338	8.046
Integrity Entry Number	857002	5.851
Integrity Entry Number	858114	6.387
Integrity Entry Number	858116	6.229
Integrity Entry Number	858117	6.149
Integrity Entry Number	861845	7.495
Integrity Entry Number	861846	7.102
Integrity Entry Number	861847	7.796

Integrity Entry Number	861848	7.301
Integrity Entry Number	861849	7.000
Integrity Entry Number	861850	6.699
Integrity Entry Number	863427	5.745
Integrity Entry Number	863454	7.699
Integrity Entry Number	863455	7.523
Integrity Entry Number	863456	7.523
Integrity Entry Number	864097	5.076
Integrity Entry Number	866604	5.883
Integrity Entry Number	868360	6.409
Integrity Entry Number	875385	6.759
Integrity Entry Number	875388	6.586
Integrity Entry Number	875502	7.051
Integrity Entry Number	875503	7.032
Integrity Entry Number	877838	5.720
Integrity Entry Number	877839	6.009
Integrity Entry Number	877844	5.240
Integrity Entry Number	878797	6.921
Integrity Entry Number	878798	7.081
Integrity Entry Number	878799	6.796
Integrity Entry Number	878800	7.337
Integrity Entry Number	881596	4.805
Integrity Entry Number	881606	4.897
Integrity Entry Number	881607	4.918
Integrity Entry	882948	6.301

Number		
Integrity Entry Number	885066	6.396
Integrity Entry Number	885067	6.770
Integrity Entry Number	890240	4.680
Integrity Entry Number	893748	5.146
Integrity Entry Number	893749	5.457
Integrity Entry Number	893750	5.216
Integrity Entry Number	894971	5.886
Integrity Entry Number	894986	5.818
Integrity Entry Number	894988	5.502
Integrity Entry Number	895844	6.701
Integrity Entry Number	895845	6.991
Integrity Entry Number	895848	6.936
Integrity Entry Number	896898	7.027
Integrity Entry Number	896899	7.097
Integrity Entry Number	903185	7.097
Integrity Entry Number	903188	7.155
Integrity Entry Number	903189	7.699
Integrity Entry Number	904333	4.745
Integrity Entry Number	906124	7.276
Integrity Entry Number	906125	7.187
Integrity Entry Number	907220	7.620
Integrity Entry Number	907222	7.796
Integrity Entry Number	907223	8.046
Integrity Entry Number	908224	6.325

Integrity Entry Number	908225	5.932
Integrity Entry Number	908226	5.557
Integrity Entry Number	914907	5.824
Integrity Entry Number	928715	5.697
Integrity Entry Number	928716	6.086
Integrity Entry Number	930709	4.417
Integrity Entry Number	933694	4.699
Integrity Entry Number	934966	3.982
Integrity Entry Number	936887	7.138
Integrity Entry Number	937014	5.706
Integrity Entry Number	937017	6.387
Integrity Entry Number	937396	6.131
Integrity Entry Number	937397	6.086
Integrity Entry Number	937398	5.102
Integrity Entry Number	937909	5.192
Integrity Entry Number	939026	5.921
Integrity Entry Number	939028	7.000
Integrity Entry Number	939029	6.046
Integrity Entry Number	939199	5.487
Integrity Entry Number	940003	6.137
Integrity Entry Number	940004	5.201
Integrity Entry Number	940005	6.237
Integrity Entry Number	940242	5.472
Integrity Entry Number	945383	5.469
Integrity Entry	945384	5.085

Number		
Integrity Entry Number	949644	4.996
Integrity Entry Number	949650	5.155
Integrity Entry Number	949723	6.106
Integrity Entry Number	953191	4.687
Integrity Entry Number	957154	5.801
Integrity Entry Number	960446	5.218
Integrity Entry Number	960447	5.227
Integrity Entry Number	964853	6.796
Integrity Entry Number	964854	6.377
Integrity Entry Number	964855	6.051
Integrity Entry Number	965298	5.032
Integrity Entry Number	965299	5.854
Integrity Entry Number	967161	5.066
Integrity Entry Number	967162	5.022
Integrity Entry Number	967168	5.092
Integrity Entry Number	968074	6.102
Integrity Entry Number	971855	7.658
Integrity Entry Number	971858	7.796
Integrity Entry Number	971859	7.469
Integrity Entry Number	971860	6.730
Integrity Entry Number	971862	6.900
Integrity Entry Number	973528	7.796
Integrity Entry Number	973529	7.770
Integrity Entry Number	973530	7.770

Integrity Entry Number	973531	7.553
Integrity Entry Number	973532	7.276
Integrity Entry Number	973533	7.481
Integrity Entry Number	973534	7.432
Integrity Entry Number	973535	7.377
Integrity Entry Number	975735	5.804
Integrity Entry Number	975749	5.569
Integrity Entry Number	980676	7.000
Integrity Entry Number	982840	6.222
Integrity Entry Number	982841	6.097
Integrity Entry Number	982842	6.155
Integrity Entry Number	982843	6.046
Integrity Entry Number	982844	6.046
Integrity Entry Number	985665	6.444
Integrity Entry Number	985668	6.187
Integrity Entry Number	990967	6.268
Integrity Entry Number	990969	6.699
Integrity Entry Number	990972	6.678
Integrity Entry Number	992925	5.521
Integrity Entry Number	992926	6.161
Integrity Entry Number	995445	7.886
Integrity Entry Number	995446	7.745
Integrity Entry Number	998410	7.076
Integrity Entry Number	999099	7.357
Integrity Entry	999102	6.527

Number		
Integrity Entry Number	999103	7.678
Integrity Entry Number	464325	6.409
Integrity Entry Number	464326	6.328
Integrity Entry Number	460675	6.174
Integrity Entry Number	727473	7.420
Integrity Entry Number	173602	6.886
Integrity Entry Number	174753	5.000
Integrity Entry Number	275308	5.398
Integrity Entry Number	217021	6.222
Integrity Entry Number	706725	7.301
Integrity Entry Number	694799	8.398
Integrity Entry Number	308542	5.398
Integrity Entry Number	204331	6.495
Integrity Entry Number	256200	7.328
Integrity Entry Number	310713	6.222
Integrity Entry Number	310715	7.000
Integrity Entry Number	310714	5.907
Integrity Entry Number	310716	6.036
Integrity Entry Number	690815	4.948
Integrity Entry Number	690814	4.840
Integrity Entry Number	690813	5.180
Integrity Entry Number	972018	5.944
Integrity Entry Number	972013	5.983
Integrity Entry Number	270600	7.509

Integrity Entry Number	270601	7.745
Integrity Entry Number	281553	7.887
Integrity Entry Number	275740	5.824
Integrity Entry Number	196540	6.585
Integrity Entry Number	260501	6.703
Integrity Entry Number	143041	6.188
Integrity Entry Number	401924	5.611
Integrity Entry Number	307361	7.886
Integrity Entry Number	748272	7.959
Integrity Entry Number	175214	8.229
Integrity Entry Number	175211	7.721
Integrity Entry Number	250430	4.785
Integrity Entry Number	207217	7.678
Integrity Entry Number	201056	6.387
Integrity Entry Number	190016	6.377
Integrity Entry Number	290137	6.886
Integrity Entry Number	419245	5.721
Integrity Entry Number	633514	5.745
Integrity Entry Number	860311	6.481
Integrity Entry Number	333529	8.328
Integrity Entry Number	307536	6.149
Integrity Entry Number	333528	8.770
Integrity Entry Number	333527	8.523
Integrity Entry Number	307537	6.201
Integrity Entry	327226	8.657

Number		
Integrity Entry Number	157734	6.523
Integrity Entry Number	274160	6.222
Integrity Entry Number	274162	6.155
Integrity Entry Number	288279	6.398
Integrity Entry Number	270091	6.155
Integrity Entry Number	265590	5.252
Integrity Entry Number	274159	5.155
Integrity Entry Number	281425	5.866
Integrity Entry Number	374030	6.770
Integrity Entry Number	395968	7.217
Integrity Entry Number	757770	9.877
Integrity Entry Number	855053	5.940
Integrity Entry Number	202156	4.268
Integrity Entry Number	179298	7.038
Integrity Entry Number	175876	7.000
Integrity Entry Number	196365	7.062
Integrity Entry Number	225432	2.651
Integrity Entry Number	184356	6.225
Integrity Entry Number	329622	6.926
Integrity Entry Number	184343	5.548
Integrity Entry Number	285544	8.097
Integrity Entry Number	313300	6.651
Integrity Entry Number	475073	9.398
Integrity Entry Number	465080	9.699

Integrity Entry Number	679466	9.456
Integrity Entry Number	632330	9.222
Integrity Entry Number	690811	5.358
Integrity Entry Number	323507	8.260
Integrity Entry Number	281389	8.319
Integrity Entry Number	652135	7.347
Integrity Entry Number	214849	5.564
Integrity Entry Number	186829	5.594
Integrity Entry Number	246087	4.959
Integrity Entry Number	287461	7.066
Integrity Entry Number	287462	7.131
Integrity Entry Number	287463	7.119
Integrity Entry Number	287460	7.432
Integrity Entry Number	170581	6.161
Integrity Entry Number	272215	7.475
Integrity Entry Number	274161	7.000
Integrity Entry Number	281424	6.398
Integrity Entry Number	284362	6.097
Integrity Entry Number	280505	6.292
Integrity Entry Number	245740	8.151
Integrity Entry Number	221016	5.667
Integrity Entry Number	236942	5.996
Integrity Entry Number	240630	6.301
Integrity Entry Number	866466	5.114
Integrity Entry	866467	5.137

Number		
Integrity Entry Number	459875	8.036
Integrity Entry Number	339200	7.509
Integrity Entry Number	366479	8.770
Integrity Entry Number	422002	8.089
Integrity Entry Number	137195	7.097
Integrity Entry Number	855052	5.906
Integrity Entry Number	628696	7.398
Integrity Entry Number	188169	5.444
Integrity Entry Number	215948	7.597
Integrity Entry Number	224671	5.921
Integrity Entry Number	196028	6.097
Integrity Entry Number	176559	5.921
Integrity Entry Number	221548	6.699
Integrity Entry Number	221545	7.301
Integrity Entry Number	221546	7.000
Integrity Entry Number	221547	6.824
Integrity Entry Number	205337	6.634
Integrity Entry Number	769736	8.496
Integrity Entry Number	325267	8.367
Integrity Entry Number	1027492	5.011
Integrity Entry Number	124501	7.213
Integrity Entry Number	113563	7.301
Integrity Entry Number	452410	7.215
Integrity Entry Number	466860	5.268

NIAID AIDSno	1	4.739
NIAID AIDSno	4	6.699
NIAID AIDSno	5	6.120
NIAID AIDSno	8	7.489
NIAID AIDSno	9	6.921
NIAID AIDSno	13	6.796
NIAID AIDSno	15	6.824
NIAID AIDSno	22	6.097
NIAID AIDSno	54	7.062
NIAID AIDSno	55	5.553
NIAID AIDSno	56	5.054
NIAID AIDSno	58	7.469
NIAID AIDSno	59	6.602
NIAID AIDSno	60	6.066
NIAID AIDSno	61	5.357
NIAID AIDSno	62	5.654
NIAID AIDSno	63	5.446
NIAID AIDSno	64	7.155
NIAID AIDSno	65	6.469
NIAID AIDSno	66	4.699
NIAID AIDSno	68	4.429
NIAID AIDSno	71	5.050
NIAID AIDSno	77	7.903
NIAID AIDSno	80	5.886
NIAID AIDSno	115	7.155
NIAID AIDSno	116	5.125
NIAID AIDSno	122	4.386
NIAID AIDSno	142	5.963
NIAID AIDSno	173	4.495
NIAID AIDSno	175	4.357
NIAID AIDSno	214	3.495
NIAID AIDSno	215	3.337
NIAID AIDSno	225	3.222
NIAID AIDSno	238	5.161
NIAID AIDSno	239	4.717
NIAID AIDSno	240	5.268
NIAID AIDSno	241	5.444
NIAID AIDSno	242	4.456
NIAID AIDSno	243	4.018
NIAID AIDSno	251	7.097
NIAID AIDSno	293	5.252
NIAID AIDSno	296	5.130
NIAID AIDSno	298	4.013
NIAID AIDSno	300	4.046
NIAID AIDSno	341	5.337

NIAID AIDSno	369	3.903
NIAID AIDSno	388	5.839
NIAID AIDSno	441	6.222
NIAID AIDSno	457	5.337
NIAID AIDSno	461	5.097
NIAID AIDSno	472	4.027
NIAID AIDSno	474	4.276
NIAID AIDSno	485	3.924
NIAID AIDSno	487	4.602
NIAID AIDSno	531	5.699
NIAID AIDSno	542	5.408
NIAID AIDSno	543	6.278
NIAID AIDSno	544	5.733
NIAID AIDSno	547	7.155
NIAID AIDSno	548	6.347
NIAID AIDSno	549	7.398
NIAID AIDSno	550	7.097
NIAID AIDSno	551	6.699
NIAID AIDSno	611	8.013
NIAID AIDSno	612	6.951
NIAID AIDSno	640	8.328
NIAID AIDSno	674	6.918
NIAID AIDSno	675	6.909
NIAID AIDSno	676	4.699
NIAID AIDSno	677	5.260
NIAID AIDSno	680	4.857
NIAID AIDSno	695	6.728
NIAID AIDSno	806	4.241
NIAID AIDSno	1101	4.410
NIAID AIDSno	1102	4.854
NIAID AIDSno	1103	5.337
NIAID AIDSno	1210	3.553
NIAID AIDSno	1299	2.921
NIAID AIDSno	1300	3.284
NIAID AIDSno	1342	6.000
NIAID AIDSno	1348	6.602
NIAID AIDSno	1373	4.102
NIAID AIDSno	1379	4.222
NIAID AIDSno	1401	3.637
NIAID AIDSno	1402	4.658
NIAID AIDSno	1411	5.119
NIAID AIDSno	1450	6.784
NIAID AIDSno	1451	5.432
NIAID AIDSno	1504	7.000
NIAID AIDSno	1505	6.284

NIAID AIDSno	1509	5.301
NIAID AIDSno	1510	4.699
NIAID AIDSno	1531	6.561
NIAID AIDSno	1776	3.936
NIAID AIDSno	1777	6.009
NIAID AIDSno	1778	6.208
NIAID AIDSno	1794	4.301
NIAID AIDSno	1795	4.660
NIAID AIDSno	1856	6.174
NIAID AIDSno	1858	5.936
NIAID AIDSno	1875	6.432
NIAID AIDSno	1905	4.301
NIAID AIDSno	1907	4.456
NIAID AIDSno	1909	7.301
NIAID AIDSno	1989	2.721
NIAID AIDSno	1994	3.284
NIAID AIDSno	2107	3.413
NIAID AIDSno	2246	4.589
NIAID AIDSno	2300	5.071
NIAID AIDSno	2301	5.347
NIAID AIDSno	2302	6.046
NIAID AIDSno	2303	5.745
NIAID AIDSno	2506	4.854
NIAID AIDSno	2507	4.969
NIAID AIDSno	2508	4.297
NIAID AIDSno	2583	5.387
NIAID AIDSno	2584	4.469
NIAID AIDSno	2585	5.898
NIAID AIDSno	2586	5.721
NIAID AIDSno	2587	6.569
NIAID AIDSno	2588	6.699
NIAID AIDSno	2589	4.959
NIAID AIDSno	2644	4.156
NIAID AIDSno	2678	5.000
NIAID AIDSno	2710	4.854
NIAID AIDSno	2728	7.699
NIAID AIDSno	2738	6.305
NIAID AIDSno	2739	6.523
NIAID AIDSno	2740	2.903
NIAID AIDSno	2756	6.921
NIAID AIDSno	2919	6.959
NIAID AIDSno	2976	7.523
NIAID AIDSno	2977	5.432
NIAID AIDSno	2978	6.678
NIAID AIDSno	2979	7.513

NIAID AIDSno	3030	4.523
NIAID AIDSno	3062	4.340
NIAID AIDSno	3075	6.132
NIAID AIDSno	3076	5.461
NIAID AIDSno	3077	5.921
NIAID AIDSno	3078	6.678
NIAID AIDSno	3079	5.943
NIAID AIDSno	3104	6.420
NIAID AIDSno	3105	5.046
NIAID AIDSno	3109	5.854
NIAID AIDSno	3112	6.456
NIAID AIDSno	3115	6.420
NIAID AIDSno	3116	6.638
NIAID AIDSno	3117	6.699
NIAID AIDSno	3118	6.000
NIAID AIDSno	3119	5.180
NIAID AIDSno	3121	4.796
NIAID AIDSno	3124	6.000
NIAID AIDSno	3129	6.187
NIAID AIDSno	3130	5.097
NIAID AIDSno	3132	6.620
NIAID AIDSno	3134	7.420
NIAID AIDSno	3135	6.721
NIAID AIDSno	3137	5.678
NIAID AIDSno	3139	5.119
NIAID AIDSno	3140	6.638
NIAID AIDSno	3141	5.538
NIAID AIDSno	3143	6.398
NIAID AIDSno	3144	6.151
NIAID AIDSno	3146	6.854
NIAID AIDSno	3147	5.959
NIAID AIDSno	3149	4.569
NIAID AIDSno	3154	7.398
NIAID AIDSno	3155	6.377
NIAID AIDSno	3156	7.444
NIAID AIDSno	3157	7.208
NIAID AIDSno	3158	6.538
NIAID AIDSno	3164	6.051
NIAID AIDSno	3166	6.770
NIAID AIDSno	3169	6.770
NIAID AIDSno	3170	6.903
NIAID AIDSno	3171	6.357
NIAID AIDSno	3172	6.347
NIAID AIDSno	3173	6.367
NIAID AIDSno	3175	6.347

NIAID AIDSno	3176	6.229
NIAID AIDSno	3177	4.959
NIAID AIDSno	3179	6.071
NIAID AIDSno	3183	6.444
NIAID AIDSno	3184	4.815
NIAID AIDSno	3185	5.538
NIAID AIDSno	3187	6.071
NIAID AIDSno	3188	6.845
NIAID AIDSno	3189	6.960
NIAID AIDSno	3190	6.119
NIAID AIDSno	3194	6.611
NIAID AIDSno	3195	5.721
NIAID AIDSno	3197	7.022
NIAID AIDSno	3198	6.959
NIAID AIDSno	3201	5.523
NIAID AIDSno	3202	5.903
NIAID AIDSno	3203	6.387
NIAID AIDSno	3204	6.620
NIAID AIDSno	3206	6.071
NIAID AIDSno	3209	5.862
NIAID AIDSno	3211	6.000
NIAID AIDSno	3215	6.301
NIAID AIDSno	3216	6.161
NIAID AIDSno	3217	6.237
NIAID AIDSno	3218	6.187
NIAID AIDSno	3219	5.481
NIAID AIDSno	3226	2.385
NIAID AIDSno	3227	2.469
NIAID AIDSno	3229	4.625
NIAID AIDSno	3230	6.409
NIAID AIDSno	3231	6.372
NIAID AIDSno	3232	6.569
NIAID AIDSno	3233	6.663
NIAID AIDSno	3234	3.517
NIAID AIDSno	3267	5.886
NIAID AIDSno	3268	4.469
NIAID AIDSno	3269	6.000
NIAID AIDSno	3270	5.745
NIAID AIDSno	3271	5.367
NIAID AIDSno	3272	5.886
NIAID AIDSno	3273	5.357
NIAID AIDSno	3274	4.432
NIAID AIDSno	3334	5.157
NIAID AIDSno	3335	6.456
NIAID AIDSno	3336	6.432

NIAID AIDSno	3337	7.393
NIAID AIDSno	3338	6.555
NIAID AIDSno	3339	7.000
NIAID AIDSno	3340	6.347
NIAID AIDSno	3341	6.912
NIAID AIDSno	3342	7.260
NIAID AIDSno	3343	7.596
NIAID AIDSno	3344	7.854
NIAID AIDSno	3363	3.425
NIAID AIDSno	3366	3.599
NIAID AIDSno	3373	6.208
NIAID AIDSno	3440	6.355
NIAID AIDSno	3516	3.857
NIAID AIDSno	3517	4.044
NIAID AIDSno	3579	4.620
NIAID AIDSno	3602	4.208
NIAID AIDSno	3610	6.000
NIAID AIDSno	3611	4.155
NIAID AIDSno	3619	5.237
NIAID AIDSno	3623	5.046
NIAID AIDSno	3689	6.959
NIAID AIDSno	3690	7.222
NIAID AIDSno	3691	5.102
NIAID AIDSno	3736	4.775
NIAID AIDSno	3763	4.851
NIAID AIDSno	3846	6.222
NIAID AIDSno	3915	4.959
NIAID AIDSno	3918	5.554
NIAID AIDSno	3919	6.057
NIAID AIDSno	3995	6.398
NIAID AIDSno	4000	6.252
NIAID AIDSno	4001	6.333
NIAID AIDSno	4002	6.426
NIAID AIDSno	4003	6.469
NIAID AIDSno	4004	5.658
NIAID AIDSno	4010	6.745
NIAID AIDSno	4011	6.839
NIAID AIDSno	4016	6.222
NIAID AIDSno	4018	7.523
NIAID AIDSno	4019	6.886
NIAID AIDSno	4020	6.620
NIAID AIDSno	4023	7.222
NIAID AIDSno	4024	6.585
NIAID AIDSno	4025	6.783
NIAID AIDSno	4026	6.174

NIAID AIDSno	4027	6.046
NIAID AIDSno	4028	7.699
NIAID AIDSno	4029	7.367
NIAID AIDSno	4030	6.194
NIAID AIDSno	4031	6.553
NIAID AIDSno	4033	7.244
NIAID AIDSno	4034	7.301
NIAID AIDSno	4035	7.201
NIAID AIDSno	4039	6.678
NIAID AIDSno	4041	7.143
NIAID AIDSno	4056	6.465
NIAID AIDSno	4059	6.602
NIAID AIDSno	4060	6.492
NIAID AIDSno	4061	7.149
NIAID AIDSno	4062	7.347
NIAID AIDSno	4063	7.337
NIAID AIDSno	4064	7.569
NIAID AIDSno	4065	7.538
NIAID AIDSno	4066	7.046
NIAID AIDSno	4067	7.509
NIAID AIDSno	4068	6.879
NIAID AIDSno	4069	7.721
NIAID AIDSno	4070	7.260
NIAID AIDSno	4071	7.638
NIAID AIDSno	4072	6.000
NIAID AIDSno	4073	7.658
NIAID AIDSno	4222	6.057
NIAID AIDSno	4227	4.854
NIAID AIDSno	4228	3.959
NIAID AIDSno	4245	4.000
NIAID AIDSno	4250	3.143
NIAID AIDSno	4327	5.699
NIAID AIDSno	4330	4.523
NIAID AIDSno	4332	4.824
NIAID AIDSno	4334	4.921
NIAID AIDSno	4336	3.614
NIAID AIDSno	4338	5.620
NIAID AIDSno	4340	3.790
NIAID AIDSno	4344	4.347
NIAID AIDSno	4462	5.721
NIAID AIDSno	4573	6.398
NIAID AIDSno	4574	7.041
NIAID AIDSno	4575	7.187
NIAID AIDSno	4576	7.721
NIAID AIDSno	4577	8.000

NIAID AIDSno	4578	7.167
NIAID AIDSno	4579	7.328
NIAID AIDSno	4580	6.167
NIAID AIDSno	4581	3.523
NIAID AIDSno	4582	6.585
NIAID AIDSno	4583	5.274
NIAID AIDSno	4584	6.456
NIAID AIDSno	4585	6.276
NIAID AIDSno	4586	6.102
NIAID AIDSno	4587	5.854
NIAID AIDSno	4588	5.839
NIAID AIDSno	4589	5.824
NIAID AIDSno	4590	5.658
NIAID AIDSno	4591	5.337
NIAID AIDSno	4592	5.161
NIAID AIDSno	4593	4.903
NIAID AIDSno	4594	4.553
NIAID AIDSno	4595	4.450
NIAID AIDSno	4596	5.903
NIAID AIDSno	4597	5.125
NIAID AIDSno	4598	3.959
NIAID AIDSno	4599	5.000
NIAID AIDSno	4600	5.810
NIAID AIDSno	4601	5.420
NIAID AIDSno	4602	7.495
NIAID AIDSno	4603	7.638
NIAID AIDSno	4604	5.886
NIAID AIDSno	4605	6.292
NIAID AIDSno	4606	7.495
NIAID AIDSno	4607	7.481
NIAID AIDSno	4608	7.398
NIAID AIDSno	4609	8.046
NIAID AIDSno	4610	7.456
NIAID AIDSno	4611	7.469
NIAID AIDSno	4612	7.155
NIAID AIDSno	4613	7.398
NIAID AIDSno	4620	6.553
NIAID AIDSno	4621	6.301
NIAID AIDSno	4622	6.276
NIAID AIDSno	4623	5.959
NIAID AIDSno	4624	5.721
NIAID AIDSno	4625	5.678
NIAID AIDSno	4626	5.629
NIAID AIDSno	4627	5.602
NIAID AIDSno	4628	5.569

NIAID AIDSno	4629	5.357
NIAID AIDSno	4631	5.000
NIAID AIDSno	4632	4.824
NIAID AIDSno	4633	4.648
NIAID AIDSno	4634	4.538
NIAID AIDSno	4635	4.491
NIAID AIDSno	4636	3.979
NIAID AIDSno	4637	3.839
NIAID AIDSno	4638	5.903
NIAID AIDSno	4639	5.783
NIAID AIDSno	4640	6.585
NIAID AIDSno	4641	6.824
NIAID AIDSno	4642	7.187
NIAID AIDSno	4643	6.959
NIAID AIDSno	4644	6.328
NIAID AIDSno	4645	5.903
NIAID AIDSno	4646	6.036
NIAID AIDSno	4647	6.979
NIAID AIDSno	4648	7.155
NIAID AIDSno	4649	6.745
NIAID AIDSno	4650	6.357
NIAID AIDSno	4651	4.611
NIAID AIDSno	4652	4.174
NIAID AIDSno	4653	7.237
NIAID AIDSno	4654	6.184
NIAID AIDSno	4655	7.000
NIAID AIDSno	4656	6.013
NIAID AIDSno	4657	5.979
NIAID AIDSno	4658	7.638
NIAID AIDSno	4659	6.947
NIAID AIDSno	4660	6.939
NIAID AIDSno	4661	7.377
NIAID AIDSno	4662	7.367
NIAID AIDSno	4663	5.947
NIAID AIDSno	4664	6.523
NIAID AIDSno	4665	5.979
NIAID AIDSno	4666	5.545
NIAID AIDSno	4667	6.721
NIAID AIDSno	4668	6.367
NIAID AIDSno	4669	6.222
NIAID AIDSno	4670	5.939
NIAID AIDSno	4671	5.757
NIAID AIDSno	4672	4.502
NIAID AIDSno	4673	6.523
NIAID AIDSno	4674	4.959

NIAID AIDSno	4676	6.475
NIAID AIDSno	4694	7.244
NIAID AIDSno	4695	6.475
NIAID AIDSno	4696	5.710
NIAID AIDSno	4704	5.138
NIAID AIDSno	4706	5.076
NIAID AIDSno	4707	4.921
NIAID AIDSno	4708	5.092
NIAID AIDSno	4709	5.270
NIAID AIDSno	4742	3.824
NIAID AIDSno	4743	3.699
NIAID AIDSno	4756	3.824
NIAID AIDSno	4757	4.155
NIAID AIDSno	4760	3.444
NIAID AIDSno	4764	4.107
NIAID AIDSno	4865	3.370
NIAID AIDSno	4867	3.333
NIAID AIDSno	4869	3.870
NIAID AIDSno	4870	4.284
NIAID AIDSno	4874	3.903
NIAID AIDSno	4877	4.260
NIAID AIDSno	4880	3.866
NIAID AIDSno	4958	4.848
NIAID AIDSno	4959	5.357
NIAID AIDSno	5053	5.699
NIAID AIDSno	5055	5.910
NIAID AIDSno	5056	5.409
NIAID AIDSno	5057	5.854
NIAID AIDSno	5058	5.260
NIAID AIDSno	5059	6.375
NIAID AIDSno	5060	5.602
NIAID AIDSno	5061	4.569
NIAID AIDSno	5062	5.081
NIAID AIDSno	5063	6.301
NIAID AIDSno	5064	5.658
NIAID AIDSno	5065	6.119
NIAID AIDSno	5066	5.252
NIAID AIDSno	5138	4.098
NIAID AIDSno	5139	4.020
NIAID AIDSno	5140	4.338
NIAID AIDSno	5141	3.779
NIAID AIDSno	5142	7.000
NIAID AIDSno	5162	4.301
NIAID AIDSno	5163	5.076
NIAID AIDSno	5164	3.585

NIAID AIDSno	5165	5.611
NIAID AIDSno	5166	7.237
NIAID AIDSno	5167	6.420
NIAID AIDSno	5168	4.870
NIAID AIDSno	5169	7.481
NIAID AIDSno	5170	7.208
NIAID AIDSno	5171	7.824
NIAID AIDSno	5172	7.398
NIAID AIDSno	5173	7.409
NIAID AIDSno	5174	7.432
NIAID AIDSno	5175	7.854
NIAID AIDSno	5176	6.770
NIAID AIDSno	5177	6.357
NIAID AIDSno	5178	5.770
NIAID AIDSno	5179	7.594
NIAID AIDSno	5180	7.284
NIAID AIDSno	5181	7.523
NIAID AIDSno	5182	7.824
NIAID AIDSno	5183	7.886
NIAID AIDSno	5184	7.367
NIAID AIDSno	5185	7.523
NIAID AIDSno	5186	7.538
NIAID AIDSno	5187	4.000
NIAID AIDSno	5188	6.721
NIAID AIDSno	5189	7.056
NIAID AIDSno	5190	7.959
NIAID AIDSno	5191	5.854
NIAID AIDSno	5192	6.699
NIAID AIDSno	5193	5.903
NIAID AIDSno	5194	5.376
NIAID AIDSno	5196	4.347
NIAID AIDSno	5197	4.796
NIAID AIDSno	5198	6.770
NIAID AIDSno	5199	7.081
NIAID AIDSno	5200	7.301
NIAID AIDSno	5201	5.959
NIAID AIDSno	5202	6.509
NIAID AIDSno	5203	4.382
NIAID AIDSno	5204	5.469
NIAID AIDSno	5205	5.678
NIAID AIDSno	5206	5.733
NIAID AIDSno	5207	6.752
NIAID AIDSno	5208	6.013
NIAID AIDSno	5209	7.523
NIAID AIDSno	5224	3.602

NIAID AIDSno	5228	8.062
NIAID AIDSno	5229	7.222
NIAID AIDSno	5230	6.959
NIAID AIDSno	5231	4.138
NIAID AIDSno	5232	5.602
NIAID AIDSno	5233	6.796
NIAID AIDSno	5234	8.261
NIAID AIDSno	5235	4.057
NIAID AIDSno	5236	6.125
NIAID AIDSno	5237	7.699
NIAID AIDSno	5238	8.111
NIAID AIDSno	5242	4.634
NIAID AIDSno	5262	4.795
NIAID AIDSno	5263	7.398
NIAID AIDSno	5265	5.770
NIAID AIDSno	5266	5.638
NIAID AIDSno	5267	4.842
NIAID AIDSno	5269	6.000
NIAID AIDSno	5271	5.569
NIAID AIDSno	5272	5.699
NIAID AIDSno	5273	5.495
NIAID AIDSno	5275	5.921
NIAID AIDSno	5276	5.357
NIAID AIDSno	5277	5.678
NIAID AIDSno	5278	5.208
NIAID AIDSno	5279	4.796
NIAID AIDSno	5316	4.934
NIAID AIDSno	5317	4.366
NIAID AIDSno	5499	5.776
NIAID AIDSno	5513	6.041
NIAID AIDSno	5514	5.851
NIAID AIDSno	5515	5.955
NIAID AIDSno	5517	7.222
NIAID AIDSno	5518	7.398
NIAID AIDSno	5520	5.879
NIAID AIDSno	5521	5.833
NIAID AIDSno	5523	6.347
NIAID AIDSno	5524	6.086
NIAID AIDSno	5525	7.046
NIAID AIDSno	5531	6.013
NIAID AIDSno	5532	5.975
NIAID AIDSno	5533	5.824
NIAID AIDSno	5534	5.553
NIAID AIDSno	5537	5.728
NIAID AIDSno	5539	5.259

NIAID AIDSno	5540	5.113
NIAID AIDSno	5545	4.699
NIAID AIDSno	5557	6.003
NIAID AIDSno	5558	5.456
NIAID AIDSno	5559	5.577
NIAID AIDSno	5560	5.870
NIAID AIDSno	5562	6.855
NIAID AIDSno	5563	6.046
NIAID AIDSno	5564	5.398
NIAID AIDSno	5565	6.114
NIAID AIDSno	5566	5.000
NIAID AIDSno	5568	6.854
NIAID AIDSno	5569	7.473
NIAID AIDSno	5570	6.013
NIAID AIDSno	5571	5.959
NIAID AIDSno	5572	5.678
NIAID AIDSno	5573	5.979
NIAID AIDSno	5574	4.896
NIAID AIDSno	5575	5.337
NIAID AIDSno	5576	6.066
NIAID AIDSno	5577	5.742
NIAID AIDSno	5578	5.201
NIAID AIDSno	5579	5.783
NIAID AIDSno	5580	6.013
NIAID AIDSno	5581	6.409
NIAID AIDSno	5582	7.248
NIAID AIDSno	5583	6.046
NIAID AIDSno	5584	6.229
NIAID AIDSno	5585	5.398
NIAID AIDSno	5586	5.839
NIAID AIDSno	5588	5.215
NIAID AIDSno	5590	4.824
NIAID AIDSno	5591	5.658
NIAID AIDSno	5592	4.292
NIAID AIDSno	5594	4.122
NIAID AIDSno	5595	4.425
NIAID AIDSno	5597	4.267
NIAID AIDSno	5599	6.658
NIAID AIDSno	5601	4.229
NIAID AIDSno	5602	4.398
NIAID AIDSno	5603	5.097
NIAID AIDSno	5604	5.000
NIAID AIDSno	5605	4.620
NIAID AIDSno	5606	5.398
NIAID AIDSno	5607	5.349

NIAID AIDSno	5608	6.143
NIAID AIDSno	5609	6.884
NIAID AIDSno	5610	5.444
NIAID AIDSno	5611	4.752
NIAID AIDSno	5612	5.000
NIAID AIDSno	5613	5.347
NIAID AIDSno	5614	6.538
NIAID AIDSno	5616	5.000
NIAID AIDSno	5617	4.585
NIAID AIDSno	5620	4.770
NIAID AIDSno	5621	5.462
NIAID AIDSno	5622	5.233
NIAID AIDSno	5623	4.553
NIAID AIDSno	5625	5.387
NIAID AIDSno	5626	6.000
NIAID AIDSno	5627	4.638
NIAID AIDSno	5628	5.180
NIAID AIDSno	5629	5.337
NIAID AIDSno	5650	6.796
NIAID AIDSno	5657	7.000
NIAID AIDSno	5710	5.286
NIAID AIDSno	5715	6.349
NIAID AIDSno	5739	4.796
NIAID AIDSno	5742	4.794
NIAID AIDSno	5748	4.854
NIAID AIDSno	5783	8.097
NIAID AIDSno	5824	8.155
NIAID AIDSno	5828	6.489
NIAID AIDSno	5832	4.500
NIAID AIDSno	5837	4.155
NIAID AIDSno	5838	4.337
NIAID AIDSno	5839	6.523
NIAID AIDSno	5841	5.046
NIAID AIDSno	5848	2.433
NIAID AIDSno	5859	5.668
NIAID AIDSno	5881	5.215
NIAID AIDSno	5930	5.432
NIAID AIDSno	5932	5.310
NIAID AIDSno	5940	5.102
NIAID AIDSno	5987	5.699
NIAID AIDSno	6045	4.187
NIAID AIDSno	6080	5.000
NIAID AIDSno	6081	7.708
NIAID AIDSno	6082	7.432
NIAID AIDSno	6083	7.190

NIAID AIDSno	6084	6.347
NIAID AIDSno	6085	6.569
NIAID AIDSno	6086	5.824
NIAID AIDSno	6087	6.886
NIAID AIDSno	6089	7.752
NIAID AIDSno	6090	7.893
NIAID AIDSno	6091	7.967
NIAID AIDSno	6092	7.963
NIAID AIDSno	6132	8.036
NIAID AIDSno	6195	5.886
NIAID AIDSno	6196	5.638
NIAID AIDSno	6197	6.076
NIAID AIDSno	6198	4.854
NIAID AIDSno	6199	5.409
NIAID AIDSno	6200	5.319
NIAID AIDSno	6201	5.523
NIAID AIDSno	6203	3.959
NIAID AIDSno	6204	4.658
NIAID AIDSno	6205	6.420
NIAID AIDSno	6206	6.469
NIAID AIDSno	6207	5.699
NIAID AIDSno	6208	3.959
NIAID AIDSno	6209	6.252
NIAID AIDSno	6210	6.194
NIAID AIDSno	6211	7.097
NIAID AIDSno	6212	5.523
NIAID AIDSno	6213	5.469
NIAID AIDSno	6214	6.569
NIAID AIDSno	6215	7.301
NIAID AIDSno	6216	5.174
NIAID AIDSno	6217	7.097
NIAID AIDSno	6218	9.222
NIAID AIDSno	6219	5.469
NIAID AIDSno	6220	7.000
NIAID AIDSno	6221	6.194
NIAID AIDSno	6222	5.585
NIAID AIDSno	6223	5.886
NIAID AIDSno	6224	7.000
NIAID AIDSno	6225	6.260
NIAID AIDSno	6226	5.602
NIAID AIDSno	6227	6.638
NIAID AIDSno	6242	5.678
NIAID AIDSno	6254	5.959
NIAID AIDSno	6265	5.174
NIAID AIDSno	6267	4.301

NIAID AIDSno	6274	5.161
NIAID AIDSno	6275	4.040
NIAID AIDSno	6279	4.301
NIAID AIDSno	6281	5.102
NIAID AIDSno	6284	4.883
NIAID AIDSno	6285	5.180
NIAID AIDSno	6339	5.260
NIAID AIDSno	6340	5.155
NIAID AIDSno	6341	5.377
NIAID AIDSno	6348	5.456
NIAID AIDSno	6349	5.585
NIAID AIDSno	6350	5.538
NIAID AIDSno	6351	5.337
NIAID AIDSno	6353	5.398
NIAID AIDSno	6354	5.658
NIAID AIDSno	6357	5.000
NIAID AIDSno	6427	3.699
NIAID AIDSno	6428	3.699
NIAID AIDSno	6429	3.699
NIAID AIDSno	6453	4.965
NIAID AIDSno	6468	6.097
NIAID AIDSno	6615	5.367
NIAID AIDSno	6616	4.456
NIAID AIDSno	6669	7.611
NIAID AIDSno	6671	7.347
NIAID AIDSno	6693	4.368
NIAID AIDSno	6847	5.063
NIAID AIDSno	6862	5.036
NIAID AIDSno	7227	7.000
NIAID AIDSno	7689	8.222
NIAID AIDSno	7845	6.222
NIAID AIDSno	7929	3.602
NIAID AIDSno	7953	7.027
NIAID AIDSno	8293	7.678
NIAID AIDSno	8715	6.886
NIAID AIDSno	8737	8.301
NIAID AIDSno	8871	6.398
NIAID AIDSno	9886	3.770
NIAID AIDSno	11160	3.369
NIAID AIDSno	11168	4.549
NIAID AIDSno	11880	4.614
NIAID AIDSno	11914	3.699
NIAID AIDSno	11943	5.699
NIAID AIDSno	11944	5.420
NIAID AIDSno	12081	7.252

NIAID AIDSno	13588	4.456
NIAID AIDSno	14115	8.260
NIAID AIDSno	14809	5.509
NIAID AIDSno	14854	4.959
NIAID AIDSno	16060	3.465
NIAID AIDSno	16150	5.149
NIAID AIDSno	17392	3.155
NIAID AIDSno	17408	3.658
NIAID AIDSno	18938	4.056
NIAID AIDSno	20768	6.409
NIAID AIDSno	21800	6.420
NIAID AIDSno	22252	6.301
NIAID AIDSno	23909	4.857
NIAID AIDSno	25260	6.000
NIAID AIDSno	25261	5.699
NIAID AIDSno	25263	5.509
NIAID AIDSno	25264	5.745
NIAID AIDSno	25266	5.678
NIAID AIDSno	25267	5.260
NIAID AIDSno	25269	5.770
NIAID AIDSno	25270	5.495
NIAID AIDSno	25271	5.469
NIAID AIDSno	25272	6.000
NIAID AIDSno	25273	5.523
NIAID AIDSno	25274	6.000
NIAID AIDSno	25275	5.699
NIAID AIDSno	25343	4.803
NIAID AIDSno	25344	4.914
NIAID AIDSno	25352	5.886
NIAID AIDSno	25353	6.000
NIAID AIDSno	25354	6.538
NIAID AIDSno	25356	5.754
NIAID AIDSno	25358	6.721
NIAID AIDSno	25359	6.260
NIAID AIDSno	25360	6.119
NIAID AIDSno	25361	7.046
NIAID AIDSno	25363	7.077
NIAID AIDSno	25367	5.886
NIAID AIDSno	25368	6.553
NIAID AIDSno	25369	6.943
NIAID AIDSno	25370	6.824
NIAID AIDSno	25385	8.456
NIAID AIDSno	25386	8.314
NIAID AIDSno	25387	8.292
NIAID AIDSno	25388	8.027

NIAID AIDSno	25389	8.000
NIAID AIDSno	25390	8.092
NIAID AIDSno	25391	5.623
NIAID AIDSno	25392	7.780
NIAID AIDSno	25393	7.699
NIAID AIDSno	25394	8.187
NIAID AIDSno	25490	5.641
NIAID AIDSno	25491	7.301
NIAID AIDSno	25492	7.538
NIAID AIDSno	25842	4.699
NIAID AIDSno	25858	5.409
NIAID AIDSno	26332	4.096
NIAID AIDSno	26336	5.373
NIAID AIDSno	26341	3.651
NIAID AIDSno	28020	4.470
NIAID AIDSno	28022	2.943
NIAID AIDSno	28029	3.359
NIAID AIDSno	28030	4.530
NIAID AIDSno	28032	3.005
NIAID AIDSno	28033	4.866
NIAID AIDSno	28058	5.456
NIAID AIDSno	28059	5.260
NIAID AIDSno	28061	4.085
NIAID AIDSno	28062	5.936
NIAID AIDSno	28064	4.027
NIAID AIDSno	28065	5.770
NIAID AIDSno	28066	4.139
NIAID AIDSno	28068	5.107
NIAID AIDSno	28069	5.883
NIAID AIDSno	28070	4.036
NIAID AIDSno	28073	5.752
NIAID AIDSno	28404	3.803
NIAID AIDSno	28407	4.921
NIAID AIDSno	28408	5.097
NIAID AIDSno	28413	6.131
NIAID AIDSno	28593	6.126
NIAID AIDSno	29767	5.155
NIAID AIDSno	29768	4.072
NIAID AIDSno	29811	5.222
NIAID AIDSno	29815	4.838
NIAID AIDSno	29818	6.876
NIAID AIDSno	29821	5.742
NIAID AIDSno	29822	6.699
NIAID AIDSno	29940	7.470
NIAID AIDSno	29941	6.699

NIAID AIDSno	29945	5.222
NIAID AIDSno	29946	5.517
NIAID AIDSno	29951	4.775
NIAID AIDSno	29968	6.699
NIAID AIDSno	29983	6.824
NIAID AIDSno	29992	7.301
NIAID AIDSno	29997	5.111
NIAID AIDSno	30000	5.780
NIAID AIDSno	30002	6.633
NIAID AIDSno	30013	6.928
NIAID AIDSno	30015	5.023
NIAID AIDSno	30017	5.234
NIAID AIDSno	30406	4.959
NIAID AIDSno	30561	5.420
NIAID AIDSno	30565	5.268
NIAID AIDSno	30712	4.824
NIAID AIDSno	30879	4.249
NIAID AIDSno	30973	6.137
NIAID AIDSno	31508	5.222
NIAID AIDSno	31515	5.155
NIAID AIDSno	31518	4.658
NIAID AIDSno	31539	6.420
NIAID AIDSno	31558	8.398
NIAID AIDSno	31903	4.810
NIAID AIDSno	32896	6.403
NIAID AIDSno	32897	6.523
NIAID AIDSno	32898	6.301
NIAID AIDSno	32899	6.745
NIAID AIDSno	32900	6.155
NIAID AIDSno	32901	6.770
NIAID AIDSno	32902	6.409
NIAID AIDSno	32903	6.456
NIAID AIDSno	32904	6.284
NIAID AIDSno	32905	7.046
NIAID AIDSno	32906	6.796
NIAID AIDSno	32907	6.602
NIAID AIDSno	32908	6.620
NIAID AIDSno	32909	6.638
NIAID AIDSno	32910	6.495
NIAID AIDSno	32911	6.319
NIAID AIDSno	32912	6.352
NIAID AIDSno	32913	6.398
NIAID AIDSno	32914	6.347
NIAID AIDSno	32915	6.824
NIAID AIDSno	32916	6.420

NIAID AIDSno	32917	6.886
NIAID AIDSno	32918	6.854
NIAID AIDSno	32919	6.620
NIAID AIDSno	32920	6.854
NIAID AIDSno	32921	6.420
NIAID AIDSno	32922	6.658
NIAID AIDSno	32923	6.102
NIAID AIDSno	32924	6.721
NIAID AIDSno	32925	6.131
NIAID AIDSno	32926	6.444
NIAID AIDSno	32927	6.276
NIAID AIDSno	32928	6.337
NIAID AIDSno	32929	5.991
NIAID AIDSno	32930	6.137
NIAID AIDSno	32931	6.678
NIAID AIDSno	32932	6.081
NIAID AIDSno	32934	7.639
NIAID AIDSno	32937	8.301
NIAID AIDSno	32941	7.056
NIAID AIDSno	33134	9.000
NIAID AIDSno	33135	7.638
NIAID AIDSno	33136	7.155
NIAID AIDSno	33137	8.097
NIAID AIDSno	33138	7.046
NIAID AIDSno	33139	6.000
NIAID AIDSno	33141	6.745
NIAID AIDSno	33142	6.602
NIAID AIDSno	33143	8.222
NIAID AIDSno	33144	7.699
NIAID AIDSno	33145	7.699
NIAID AIDSno	33146	8.000
NIAID AIDSno	33147	7.523
NIAID AIDSno	33149	6.721
NIAID AIDSno	33150	6.638
NIAID AIDSno	33151	6.409
NIAID AIDSno	33152	7.046
NIAID AIDSno	33153	7.046
NIAID AIDSno	33190	7.155
NIAID AIDSno	33191	7.398
NIAID AIDSno	33192	6.745
NIAID AIDSno	33193	6.745
NIAID AIDSno	33194	5.921
NIAID AIDSno	33195	5.959
NIAID AIDSno	33196	7.301
NIAID AIDSno	33197	6.824

NIAID AIDSno	33220	5.292
NIAID AIDSno	33221	6.523
NIAID AIDSno	33222	6.076
NIAID AIDSno	33223	5.347
NIAID AIDSno	33224	5.022
NIAID AIDSno	33228	6.046
NIAID AIDSno	33229	7.398
NIAID AIDSno	33241	6.699
NIAID AIDSno	33242	6.398
NIAID AIDSno	33244	5.959
NIAID AIDSno	33245	7.046
NIAID AIDSno	33246	6.699
NIAID AIDSno	33247	8.523
NIAID AIDSno	33248	8.000
NIAID AIDSno	33249	8.000
NIAID AIDSno	33250	8.000
NIAID AIDSno	33251	8.222
NIAID AIDSno	33252	6.699
NIAID AIDSno	33255	5.721
NIAID AIDSno	33256	6.187
NIAID AIDSno	33257	5.921
NIAID AIDSno	33258	5.347
NIAID AIDSno	33260	6.347
NIAID AIDSno	33263	5.824
NIAID AIDSno	33264	7.000
NIAID AIDSno	33265	6.222
NIAID AIDSno	33266	6.456
NIAID AIDSno	33267	6.699
NIAID AIDSno	33268	5.602
NIAID AIDSno	33269	6.699
NIAID AIDSno	33270	6.260
NIAID AIDSno	33272	6.699
NIAID AIDSno	33273	5.620
NIAID AIDSno	33275	5.721
NIAID AIDSno	33276	5.409
NIAID AIDSno	33277	7.699
NIAID AIDSno	33279	5.886
NIAID AIDSno	33280	7.523
NIAID AIDSno	33281	6.699
NIAID AIDSno	33282	8.260
NIAID AIDSno	33283	8.222
NIAID AIDSno	33284	8.523
NIAID AIDSno	33285	8.347
NIAID AIDSno	33286	7.301
NIAID AIDSno	33419	8.222

NIAID AIDSno	33420	8.301
NIAID AIDSno	33421	8.523
NIAID AIDSno	33422	9.000
NIAID AIDSno	33423	8.301
NIAID AIDSno	33424	8.155
NIAID AIDSno	33425	8.699
NIAID AIDSno	33426	8.699
NIAID AIDSno	33427	7.699
NIAID AIDSno	33428	8.097
NIAID AIDSno	33429	7.678
NIAID AIDSno	33430	7.155
NIAID AIDSno	33431	7.046
NIAID AIDSno	33432	8.398
NIAID AIDSno	33433	7.222
NIAID AIDSno	33434	6.784
NIAID AIDSno	33435	6.495
NIAID AIDSno	33436	8.523
NIAID AIDSno	33437	6.824
NIAID AIDSno	33438	7.678
NIAID AIDSno	33439	9.000
NIAID AIDSno	33440	8.699
NIAID AIDSno	33451	7.347
NIAID AIDSno	33452	8.046
NIAID AIDSno	33453	8.155
NIAID AIDSno	33454	8.222
NIAID AIDSno	33455	8.398
NIAID AIDSno	33456	8.398
NIAID AIDSno	33457	7.921
NIAID AIDSno	33458	8.097
NIAID AIDSno	33459	8.301
NIAID AIDSno	33460	8.699
NIAID AIDSno	33461	9.000
NIAID AIDSno	33462	8.523
NIAID AIDSno	33463	8.301
NIAID AIDSno	33464	8.301
NIAID AIDSno	33465	7.745
NIAID AIDSno	33466	8.301
NIAID AIDSno	33467	7.328
NIAID AIDSno	33549	7.222
NIAID AIDSno	33550	6.824
NIAID AIDSno	33551	6.000
NIAID AIDSno	33552	6.824
NIAID AIDSno	33553	6.456
NIAID AIDSno	33554	7.097
NIAID AIDSno	33555	7.523

NIAID AIDSno	33556	6.824
NIAID AIDSno	33557	5.959
NIAID AIDSno	33558	6.222
NIAID AIDSno	33559	7.222
NIAID AIDSno	33563	7.699
NIAID AIDSno	33564	6.854
NIAID AIDSno	33565	7.155
NIAID AIDSno	33566	8.000
NIAID AIDSno	33567	7.699
NIAID AIDSno	33568	7.523
NIAID AIDSno	33569	7.398
NIAID AIDSno	33570	6.523
NIAID AIDSno	33571	6.398
NIAID AIDSno	33572	6.824
NIAID AIDSno	33573	7.046
NIAID AIDSno	33574	6.509
NIAID AIDSno	33575	6.328
NIAID AIDSno	33576	7.398
NIAID AIDSno	33577	6.921
NIAID AIDSno	33578	7.699
NIAID AIDSno	33579	6.959
NIAID AIDSno	33580	7.398
NIAID AIDSno	33581	7.155
NIAID AIDSno	33582	7.502
NIAID AIDSno	33583	7.301
NIAID AIDSno	33584	6.854
NIAID AIDSno	33585	7.000
NIAID AIDSno	33586	6.420
NIAID AIDSno	33587	7.000
NIAID AIDSno	33588	6.959
NIAID AIDSno	33589	6.658
NIAID AIDSno	33590	5.432
NIAID AIDSno	33591	6.886
NIAID AIDSno	33592	6.409
NIAID AIDSno	33593	7.699
NIAID AIDSno	33594	7.222
NIAID AIDSno	33595	6.638
NIAID AIDSno	33596	6.086
NIAID AIDSno	33597	6.824
NIAID AIDSno	33598	7.155
NIAID AIDSno	33599	5.854
NIAID AIDSno	39057	5.194
NIAID AIDSno	39976	6.155
NIAID AIDSno	42741	4.420
NIAID AIDSno	42873	5.780

NIAID AIDSno	42874	5.092
NIAID AIDSno	42875	6.662
NIAID AIDSno	42876	7.237
NIAID AIDSno	42877	6.893
NIAID AIDSno	42878	6.056
NIAID AIDSno	42879	5.432
NIAID AIDSno	42880	4.347
NIAID AIDSno	42881	3.721
NIAID AIDSno	42883	4.577
NIAID AIDSno	42884	3.678
NIAID AIDSno	42887	4.268
NIAID AIDSno	42888	4.796
NIAID AIDSno	42889	5.208
NIAID AIDSno	42961	8.523
NIAID AIDSno	43128	4.208
NIAID AIDSno	43129	3.000
NIAID AIDSno	43134	5.851
NIAID AIDSno	43135	6.921
NIAID AIDSno	43136	6.569
NIAID AIDSno	43137	6.377
NIAID AIDSno	43139	7.222
NIAID AIDSno	43140	5.987
NIAID AIDSno	43142	5.987
NIAID AIDSno	43143	6.155
NIAID AIDSno	43145	5.658
NIAID AIDSno	43146	6.194
NIAID AIDSno	43148	6.538
NIAID AIDSno	43149	5.614
NIAID AIDSno	43150	6.237
NIAID AIDSno	43151	6.337
NIAID AIDSno	43152	5.516
NIAID AIDSno	43153	7.000
NIAID AIDSno	43154	5.983
NIAID AIDSno	43155	6.387
NIAID AIDSno	43156	5.947
NIAID AIDSno	43157	6.854
NIAID AIDSno	43158	6.721
NIAID AIDSno	43159	6.071
NIAID AIDSno	43160	6.699
NIAID AIDSno	43161	6.959
NIAID AIDSno	43169	6.036
NIAID AIDSno	43170	6.097
NIAID AIDSno	43171	6.620
NIAID AIDSno	43172	7.301
NIAID AIDSno	43173	5.398

NIAID AIDSno	43174	6.469
NIAID AIDSno	43175	6.959
NIAID AIDSno	43178	6.377
NIAID AIDSno	43183	6.481
NIAID AIDSno	43185	6.310
NIAID AIDSno	43188	7.523
NIAID AIDSno	43192	7.000
NIAID AIDSno	43194	7.824
NIAID AIDSno	43213	6.222
NIAID AIDSno	43214	6.398
NIAID AIDSno	43333	6.398
NIAID AIDSno	43349	5.886
NIAID AIDSno	43389	7.000
NIAID AIDSno	43407	7.362
NIAID AIDSno	43409	4.156
NIAID AIDSno	43411	5.292
NIAID AIDSno	43484	5.301
NIAID AIDSno	43485	5.398
NIAID AIDSno	43504	6.347
NIAID AIDSno	43507	6.398
NIAID AIDSno	43509	6.569
NIAID AIDSno	43511	6.046
NIAID AIDSno	44622	5.177
NIAID AIDSno	44625	2.699
NIAID AIDSno	44626	4.301
NIAID AIDSno	44627	2.699
NIAID AIDSno	44630	3.903
NIAID AIDSno	44632	4.699
NIAID AIDSno	44634	3.000
NIAID AIDSno	44705	6.485
NIAID AIDSno	44707	6.699
NIAID AIDSno	45028	7.097
NIAID AIDSno	45138	5.022
NIAID AIDSno	45160	4.337
NIAID AIDSno	45161	4.420
NIAID AIDSno	45162	4.495
NIAID AIDSno	45163	5.155
NIAID AIDSno	45312	5.092
NIAID AIDSno	45315	4.312
NIAID AIDSno	45320	4.648
NIAID AIDSno	45321	4.347
NIAID AIDSno	45323	5.131
NIAID AIDSno	45324	4.480
NIAID AIDSno	45326	4.527
NIAID AIDSno	45345	5.569

NIAID AIDSno	45346	5.469
NIAID AIDSno	45347	5.966
NIAID AIDSno	45536	5.428
NIAID AIDSno	45663	5.467
NIAID AIDSno	45999	4.000
NIAID AIDSno	46000	4.187
NIAID AIDSno	46005	3.699
NIAID AIDSno	46009	4.002
NIAID AIDSno	46013	3.770
NIAID AIDSno	48396	4.658
NIAID AIDSno	48857	5.721
NIAID AIDSno	50093	7.367
NIAID AIDSno	50410	5.854
NIAID AIDSno	50415	4.910
NIAID AIDSno	50645	7.553
NIAID AIDSno	50646	6.699
NIAID AIDSno	50647	6.151
NIAID AIDSno	50649	6.886
NIAID AIDSno	50650	7.357
NIAID AIDSno	50651	6.678
NIAID AIDSno	50724	4.658
NIAID AIDSno	50725	4.570
NIAID AIDSno	50737	4.857
NIAID AIDSno	50738	5.092
NIAID AIDSno	50739	4.588
NIAID AIDSno	50741	5.131
NIAID AIDSno	50742	4.757
NIAID AIDSno	50743	4.991
NIAID AIDSno	50744	5.097
NIAID AIDSno	50747	5.108
NIAID AIDSno	50748	4.860
NIAID AIDSno	50749	4.471
NIAID AIDSno	51610	4.398
NIAID AIDSno	51722	5.027
NIAID AIDSno	51723	5.784
NIAID AIDSno	51724	5.674
NIAID AIDSno	51725	5.523
NIAID AIDSno	51726	5.585
NIAID AIDSno	51727	5.921
NIAID AIDSno	51729	5.585
NIAID AIDSno	51733	5.538
NIAID AIDSno	51734	5.076
NIAID AIDSno	51735	5.310
NIAID AIDSno	51736	5.602
NIAID AIDSno	51737	5.658

NIAID AIDSno	51738	5.602
NIAID AIDSno	51739	5.602
NIAID AIDSno	51740	5.337
NIAID AIDSno	51741	5.960
NIAID AIDSno	51742	5.885
NIAID AIDSno	51743	5.367
NIAID AIDSno	51744	5.215
NIAID AIDSno	51745	5.367
NIAID AIDSno	51746	5.337
NIAID AIDSno	51747	5.367
NIAID AIDSno	51787	6.155
NIAID AIDSno	51788	6.301
NIAID AIDSno	51793	5.509
NIAID AIDSno	51860	4.000
NIAID AIDSno	51861	5.301
NIAID AIDSno	51862	3.222
NIAID AIDSno	51864	3.301
NIAID AIDSno	51865	3.301
NIAID AIDSno	51867	4.000
NIAID AIDSno	51871	5.000
NIAID AIDSno	51872	5.301
NIAID AIDSno	51873	5.000
NIAID AIDSno	51874	6.662
NIAID AIDSno	51875	3.301
NIAID AIDSno	51876	4.602
NIAID AIDSno	51878	6.301
NIAID AIDSno	51879	3.824
NIAID AIDSno	51881	6.523
NIAID AIDSno	51935	3.903
NIAID AIDSno	57025	3.627
NIAID AIDSno	57146	6.523
NIAID AIDSno	57755	7.620
NIAID AIDSno	57840	4.000
NIAID AIDSno	57843	4.462
NIAID AIDSno	57849	5.721
NIAID AIDSno	57850	5.658
NIAID AIDSno	57901	5.097
NIAID AIDSno	57906	5.398
NIAID AIDSno	57908	5.222
NIAID AIDSno	57911	5.000
NIAID AIDSno	57913	7.699
NIAID AIDSno	57983	8.810
NIAID AIDSno	57985	8.855
NIAID AIDSno	57986	8.495
NIAID AIDSno	57988	8.369

NIAID AIDSno	58025	7.202
NIAID AIDSno	58054	5.328
NIAID AIDSno	58092	5.201
NIAID AIDSno	58093	5.022
NIAID AIDSno	58098	4.509
NIAID AIDSno	58106	5.569
NIAID AIDSno	58112	5.538
NIAID AIDSno	58123	4.620
NIAID AIDSno	58124	4.000
NIAID AIDSno	58190	6.770
NIAID AIDSno	58193	5.569
NIAID AIDSno	58194	5.319
NIAID AIDSno	58195	5.745
NIAID AIDSno	58196	5.959
NIAID AIDSno	58197	5.678
NIAID AIDSno	58198	5.721
NIAID AIDSno	58202	5.658
NIAID AIDSno	58204	6.301
NIAID AIDSno	58205	6.120
NIAID AIDSno	58206	6.372
NIAID AIDSno	58207	6.345
NIAID AIDSno	58208	6.427
NIAID AIDSno	58209	4.620
NIAID AIDSno	58210	5.000
NIAID AIDSno	58211	4.959
NIAID AIDSno	58212	5.398
NIAID AIDSno	58237	4.301
NIAID AIDSno	58239	5.597
NIAID AIDSno	58247	5.301
NIAID AIDSno	58249	4.495
NIAID AIDSno	58265	4.222
NIAID AIDSno	58266	3.839
NIAID AIDSno	58267	3.523
NIAID AIDSno	58268	3.523
NIAID AIDSno	58269	3.523
NIAID AIDSno	58270	3.523
NIAID AIDSno	58271	3.523
NIAID AIDSno	58773	8.523
NIAID AIDSno	58774	8.523
NIAID AIDSno	58775	8.523
NIAID AIDSno	58776	8.523
NIAID AIDSno	58777	8.523
NIAID AIDSno	58778	8.523
NIAID AIDSno	58779	8.523
NIAID AIDSno	58780	8.523

NIAID AIDSno	58781	8.523
NIAID AIDSno	58782	8.523
NIAID AIDSno	58783	8.523
NIAID AIDSno	58784	8.523
NIAID AIDSno	58785	8.523
NIAID AIDSno	58786	8.523
NIAID AIDSno	58787	8.523
NIAID AIDSno	58790	3.924
NIAID AIDSno	58791	4.495
NIAID AIDSno	58800	4.770
NIAID AIDSno	58803	4.284
NIAID AIDSno	58860	4.796
NIAID AIDSno	58867	6.959
NIAID AIDSno	58868	6.699
NIAID AIDSno	58875	4.730
NIAID AIDSno	58876	5.796
NIAID AIDSno	58877	5.000
NIAID AIDSno	58880	5.000
NIAID AIDSno	59182	2.754
NIAID AIDSno	59215	8.523
NIAID AIDSno	59241	7.770
NIAID AIDSno	59281	4.347
NIAID AIDSno	59282	5.921
NIAID AIDSno	59283	5.959
NIAID AIDSno	59284	6.097
NIAID AIDSno	59435	4.337
NIAID AIDSno	59436	4.842
NIAID AIDSno	59437	4.447
NIAID AIDSno	59439	4.581
NIAID AIDSno	59442	5.469
NIAID AIDSno	59515	4.585
NIAID AIDSno	59517	4.500
NIAID AIDSno	59518	4.027
NIAID AIDSno	59519	5.252
NIAID AIDSno	59521	4.796
NIAID AIDSno	59523	5.699
NIAID AIDSno	59524	4.201
NIAID AIDSno	59526	5.444
NIAID AIDSno	59528	4.018
NIAID AIDSno	59534	5.495
NIAID AIDSno	59535	4.959
NIAID AIDSno	59537	8.523
NIAID AIDSno	59538	7.602
NIAID AIDSno	59539	8.523
NIAID AIDSno	59540	8.097

NIAID AIDSno	59541	7.000
NIAID AIDSno	59542	8.398
NIAID AIDSno	59543	8.382
NIAID AIDSno	59544	8.155
NIAID AIDSno	59596	4.726
NIAID AIDSno	59597	5.013
NIAID AIDSno	59598	5.215
NIAID AIDSno	59599	5.319
NIAID AIDSno	59615	8.699
NIAID AIDSno	59616	8.097
NIAID AIDSno	59618	8.699
NIAID AIDSno	59983	4.816
NIAID AIDSno	60326	5.301
NIAID AIDSno	60334	5.509
NIAID AIDSno	60335	5.469
NIAID AIDSno	60338	5.319
NIAID AIDSno	60342	5.187
NIAID AIDSno	60343	5.155
NIAID AIDSno	60345	5.456
NIAID AIDSno	60347	5.328
NIAID AIDSno	60349	4.983
NIAID AIDSno	60379	7.398
NIAID AIDSno	60979	6.000
NIAID AIDSno	60980	6.222
NIAID AIDSno	60981	6.155
NIAID AIDSno	60982	6.398
NIAID AIDSno	60983	6.155
NIAID AIDSno	60984	5.555
NIAID AIDSno	60985	6.046
NIAID AIDSno	60986	5.194
NIAID AIDSno	60987	5.602
NIAID AIDSno	61017	5.444
NIAID AIDSno	61019	5.770
NIAID AIDSno	61021	3.914
NIAID AIDSno	61022	5.276
NIAID AIDSno	64681	4.699
NIAID AIDSno	64683	4.347
NIAID AIDSno	64684	4.638
NIAID AIDSno	64685	5.301
NIAID AIDSno	64688	4.959
NIAID AIDSno	64689	5.137
NIAID AIDSno	70149	6.333
NIAID AIDSno	70157	6.721
NIAID AIDSno	70159	4.796
NIAID AIDSno	70162	7.071

NIAID AIDSno	70179	6.538
NIAID AIDSno	70192	5.280
NIAID AIDSno	70193	6.699
NIAID AIDSno	70194	7.301
NIAID AIDSno	70197	6.553
NIAID AIDSno	70202	6.481
NIAID AIDSno	70204	6.009
NIAID AIDSno	70206	6.119
NIAID AIDSno	70209	6.310
NIAID AIDSno	70222	6.384
NIAID AIDSno	70232	5.509
NIAID AIDSno	70233	4.678
NIAID AIDSno	70234	4.824
NIAID AIDSno	70241	5.060
NIAID AIDSno	70242	5.071
NIAID AIDSno	70243	4.854
NIAID AIDSno	70244	4.975
NIAID AIDSno	70245	3.921
NIAID AIDSno	70247	4.721
NIAID AIDSno	70248	4.921
NIAID AIDSno	70250	5.137
NIAID AIDSno	70251	5.328
NIAID AIDSno	70252	4.745
NIAID AIDSno	70253	5.060
NIAID AIDSno	70254	5.046
NIAID AIDSno	70255	4.721
NIAID AIDSno	70256	4.824
NIAID AIDSno	70258	4.180
NIAID AIDSno	70261	4.456
NIAID AIDSno	70290	5.237
NIAID AIDSno	70298	7.699
NIAID AIDSno	70299	7.398
NIAID AIDSno	70313	5.481
NIAID AIDSno	70314	5.260
NIAID AIDSno	70348	7.097
NIAID AIDSno	70349	7.301
NIAID AIDSno	70350	6.585
NIAID AIDSno	70351	7.097
NIAID AIDSno	70353	6.959
NIAID AIDSno	70355	6.678
NIAID AIDSno	70356	6.229
NIAID AIDSno	70357	7.222
NIAID AIDSno	70358	7.000
NIAID AIDSno	70359	6.886
NIAID AIDSno	70360	6.678

NIAID AIDSno	70361	6.770
NIAID AIDSno	70363	6.553
NIAID AIDSno	70365	6.155
NIAID AIDSno	70366	6.097
NIAID AIDSno	70367	7.046
NIAID AIDSno	70368	6.886
NIAID AIDSno	70369	7.301
NIAID AIDSno	70370	6.770
NIAID AIDSno	70372	7.000
NIAID AIDSno	70373	6.481
NIAID AIDSno	70374	6.509
NIAID AIDSno	70375	6.721
NIAID AIDSno	70376	7.699
NIAID AIDSno	70377	7.301
NIAID AIDSno	70378	7.155
NIAID AIDSno	70379	7.523
NIAID AIDSno	70380	7.398
NIAID AIDSno	70381	6.678
NIAID AIDSno	70382	7.523
NIAID AIDSno	70383	6.638
NIAID AIDSno	70384	7.000
NIAID AIDSno	70385	6.921
NIAID AIDSno	70386	7.699
NIAID AIDSno	70387	7.155
NIAID AIDSno	70388	7.000
NIAID AIDSno	70389	7.523
NIAID AIDSno	70390	8.111
NIAID AIDSno	70391	7.155
NIAID AIDSno	70392	7.301
NIAID AIDSno	70395	6.125
NIAID AIDSno	70396	5.959
NIAID AIDSno	70398	7.301
NIAID AIDSno	70399	6.367
NIAID AIDSno	70400	6.523
NIAID AIDSno	70401	6.678
NIAID AIDSno	70402	6.208
NIAID AIDSno	70403	7.046
NIAID AIDSno	70405	6.921
NIAID AIDSno	70407	7.301
NIAID AIDSno	70408	6.921
NIAID AIDSno	70409	6.602
NIAID AIDSno	70410	5.523
NIAID AIDSno	70411	7.523
NIAID AIDSno	70412	6.886
NIAID AIDSno	70413	6.721

NIAID AIDSno	70414	6.268
NIAID AIDSno	70415	7.398
NIAID AIDSno	70416	7.000
NIAID AIDSno	70417	6.959
NIAID AIDSno	70418	6.699
NIAID AIDSno	70419	7.097
NIAID AIDSno	70420	6.921
NIAID AIDSno	70421	5.991
NIAID AIDSno	70422	7.699
NIAID AIDSno	70423	6.638
NIAID AIDSno	70424	6.959
NIAID AIDSno	70425	6.229
NIAID AIDSno	70426	7.046
NIAID AIDSno	70427	7.301
NIAID AIDSno	70429	6.337
NIAID AIDSno	70430	7.222
NIAID AIDSno	70431	7.097
NIAID AIDSno	70432	5.824
NIAID AIDSno	70433	7.699
NIAID AIDSno	70434	7.523
NIAID AIDSno	70435	7.222
NIAID AIDSno	70436	7.398
NIAID AIDSno	70437	7.097
NIAID AIDSno	70438	6.796
NIAID AIDSno	70439	6.921
NIAID AIDSno	70440	7.222
NIAID AIDSno	70441	6.824
NIAID AIDSno	70442	6.796
NIAID AIDSno	70443	6.854
NIAID AIDSno	70444	6.481
NIAID AIDSno	70445	7.301
NIAID AIDSno	70452	6.357
NIAID AIDSno	70453	6.357
NIAID AIDSno	70457	5.824
NIAID AIDSno	70458	5.553
NIAID AIDSno	70770	6.398
NIAID AIDSno	70771	5.824
NIAID AIDSno	70772	4.886
NIAID AIDSno	70773	6.398
NIAID AIDSno	70774	5.745
NIAID AIDSno	70775	5.523
NIAID AIDSno	70776	5.824
NIAID AIDSno	70777	6.301
NIAID AIDSno	70778	6.222
NIAID AIDSno	70779	7.000

NIAID AIDSno	70780	6.398
NIAID AIDSno	70781	6.398
NIAID AIDSno	70782	6.097
NIAID AIDSno	70783	7.301
NIAID AIDSno	70784	6.699
NIAID AIDSno	70785	6.699
NIAID AIDSno	70786	7.301
NIAID AIDSno	70787	7.097
NIAID AIDSno	70788	7.046
NIAID AIDSno	70792	5.959
NIAID AIDSno	70793	7.222
NIAID AIDSno	70794	5.770
NIAID AIDSno	70795	5.310
NIAID AIDSno	70796	6.699
NIAID AIDSno	70797	7.301
NIAID AIDSno	70798	7.046
NIAID AIDSno	70799	7.046
NIAID AIDSno	70800	7.301
NIAID AIDSno	70801	7.097
NIAID AIDSno	70802	7.155
NIAID AIDSno	70817	5.092
NIAID AIDSno	70818	5.754
NIAID AIDSno	70819	5.854
NIAID AIDSno	70920	5.796
NIAID AIDSno	70921	5.721
NIAID AIDSno	70922	5.319
NIAID AIDSno	70923	6.523
NIAID AIDSno	70924	6.222
NIAID AIDSno	70925	5.131
NIAID AIDSno	70926	6.523
NIAID AIDSno	70927	6.523
NIAID AIDSno	70928	5.921
NIAID AIDSno	70929	6.398
NIAID AIDSno	70930	6.046
NIAID AIDSno	70931	5.071
NIAID AIDSno	70933	5.939
NIAID AIDSno	70934	5.939
NIAID AIDSno	71008	6.529
NIAID AIDSno	71018	4.000
NIAID AIDSno	71475	3.000
NIAID AIDSno	71476	3.903
NIAID AIDSno	71477	4.081
NIAID AIDSno	71478	6.097
NIAID AIDSno	71540	6.630
NIAID AIDSno	71569	6.398

NIAID AIDSno	72063	5.412
NIAID AIDSno	72064	5.301
NIAID AIDSno	72065	5.523
NIAID AIDSno	72088	4.509
NIAID AIDSno	72091	5.237
NIAID AIDSno	72092	4.685
NIAID AIDSno	72094	4.821
NIAID AIDSno	72198	6.420
NIAID AIDSno	72433	3.125
NIAID AIDSno	72461	5.068
NIAID AIDSno	72662	6.456
NIAID AIDSno	73035	9.149
NIAID AIDSno	75039	5.272
NIAID AIDSno	75042	5.401
NIAID AIDSno	75044	5.456
NIAID AIDSno	75047	4.553
NIAID AIDSno	75049	5.377
NIAID AIDSno	75056	4.481
NIAID AIDSno	75058	5.276
NIAID AIDSno	76481	4.018
NIAID AIDSno	76591	5.194
NIAID AIDSno	76643	5.721
NIAID AIDSno	80350	6.456
NIAID AIDSno	80364	5.886
NIAID AIDSno	80370	5.469
NIAID AIDSno	80403	5.824
NIAID AIDSno	80404	6.002
NIAID AIDSno	80571	5.155
NIAID AIDSno	80572	5.252
NIAID AIDSno	80573	6.301
NIAID AIDSno	80741	6.420
NIAID AIDSno	81404	8.577
NIAID AIDSno	81518	7.312
NIAID AIDSno	81612	9.398
NIAID AIDSno	81670	7.131
NIAID AIDSno	81671	7.131
NIAID AIDSno	81734	6.955
NIAID AIDSno	81735	7.513
NIAID AIDSno	81737	7.268
NIAID AIDSno	82403	4.947
NIAID AIDSno	82407	5.357
NIAID AIDSno	82408	7.523
NIAID AIDSno	82413	6.678
NIAID AIDSno	82414	6.618
NIAID AIDSno	82417	6.420

NIAID AIDSno	82418	6.592
NIAID AIDSno	82420	6.186
NIAID AIDSno	82423	6.889
NIAID AIDSno	82424	6.804
NIAID AIDSno	82425	6.833
NIAID AIDSno	82426	6.772
NIAID AIDSno	82428	5.699
NIAID AIDSno	82429	7.745
NIAID AIDSno	82430	7.959
NIAID AIDSno	82431	7.125
NIAID AIDSno	82432	7.569
NIAID AIDSno	82433	6.924
NIAID AIDSno	82505	2.656
NIAID AIDSno	82506	2.352
NIAID AIDSno	82507	3.449
NIAID AIDSno	82508	5.237
NIAID AIDSno	82509	6.523
NIAID AIDSno	82905	5.678
NIAID AIDSno	82909	5.481
NIAID AIDSno	85085	5.721
NIAID AIDSno	85207	3.523
NIAID AIDSno	85216	4.623
NIAID AIDSno	85217	5.208
NIAID AIDSno	85218	5.187
NIAID AIDSno	85500	6.097
NIAID AIDSno	85619	3.699
NIAID AIDSno	85621	3.569
NIAID AIDSno	85625	5.000
NIAID AIDSno	85665	8.398
NIAID AIDSno	85666	6.650
NIAID AIDSno	85667	6.963
NIAID AIDSno	85668	8.284
NIAID AIDSno	85669	7.796
NIAID AIDSno	85670	8.569
NIAID AIDSno	85671	8.456
NIAID AIDSno	85672	8.276
NIAID AIDSno	85673	8.167
NIAID AIDSno	85674	6.870
NIAID AIDSno	85675	6.310
NIAID AIDSno	85676	5.398
NIAID AIDSno	85692	4.921
NIAID AIDSno	85693	4.921
NIAID AIDSno	85791	4.268
NIAID AIDSno	85792	4.041
NIAID AIDSno	85864	6.321

NIAID AIDSno	85865	6.721
NIAID AIDSno	85866	5.708
NIAID AIDSno	85867	6.088
NIAID AIDSno	85868	5.903
NIAID AIDSno	85869	7.076
NIAID AIDSno	85870	6.099
NIAID AIDSno	85871	6.097
NIAID AIDSno	85872	6.355
NIAID AIDSno	85876	6.883
NIAID AIDSno	85879	5.876
NIAID AIDSno	85880	5.719
NIAID AIDSno	85980	7.108
NIAID AIDSno	85981	6.896
NIAID AIDSno	85982	6.807
NIAID AIDSno	85983	6.991
NIAID AIDSno	85984	6.680
NIAID AIDSno	85985	6.559
NIAID AIDSno	85986	6.517
NIAID AIDSno	85987	6.701
NIAID AIDSno	85988	6.096
NIAID AIDSno	85989	5.723
NIAID AIDSno	85990	5.822
NIAID AIDSno	85991	6.048
NIAID AIDSno	85992	6.216
NIAID AIDSno	85993	6.325
NIAID AIDSno	86014	5.276
NIAID AIDSno	86015	5.310
NIAID AIDSno	86018	6.155
NIAID AIDSno	86019	5.921
NIAID AIDSno	86081	4.319
NIAID AIDSno	86082	4.409
NIAID AIDSno	86104	4.387
NIAID AIDSno	86105	6.469
NIAID AIDSno	86108	5.323
NIAID AIDSno	86109	4.827
NIAID AIDSno	86186	4.530
NIAID AIDSno	86189	4.745
NIAID AIDSno	86190	4.770
NIAID AIDSno	86191	4.569
NIAID AIDSno	86192	4.658
NIAID AIDSno	86193	5.602
NIAID AIDSno	86194	5.194
NIAID AIDSno	86198	5.328
NIAID AIDSno	86213	5.481
NIAID AIDSno	86215	4.481

NIAID AIDSno	86216	5.481
NIAID AIDSno	86217	4.481
NIAID AIDSno	86431	4.575
NIAID AIDSno	86432	7.564
NIAID AIDSno	86446	7.222
NIAID AIDSno	86447	7.155
NIAID AIDSno	86734	6.523
NIAID AIDSno	86739	5.000
NIAID AIDSno	87065	4.648
NIAID AIDSno	87066	4.352
NIAID AIDSno	87067	4.138
NIAID AIDSno	87068	4.410
NIAID AIDSno	87069	5.023
NIAID AIDSno	87070	3.703
NIAID AIDSno	87071	4.471
NIAID AIDSno	87072	4.203
NIAID AIDSno	87075	6.201
NIAID AIDSno	87076	6.176
NIAID AIDSno	87077	5.531
NIAID AIDSno	87078	6.143
NIAID AIDSno	87079	5.604
NIAID AIDSno	87080	5.256
NIAID AIDSno	87081	6.406
NIAID AIDSno	87082	4.889
NIAID AIDSno	87083	5.204
NIAID AIDSno	87084	5.554
NIAID AIDSno	87085	5.534
NIAID AIDSno	87086	5.660
NIAID AIDSno	87087	5.789
NIAID AIDSno	87088	6.764
NIAID AIDSno	87360	4.613
NIAID AIDSno	87380	4.631
NIAID AIDSno	87387	5.441
NIAID AIDSno	87575	4.199
NIAID AIDSno	87578	4.000
NIAID AIDSno	87580	4.000
NIAID AIDSno	87864	5.276
NIAID AIDSno	87978	7.301
NIAID AIDSno	87979	5.678
NIAID AIDSno	87980	5.824
NIAID AIDSno	87982	5.638
NIAID AIDSno	87984	5.854
NIAID AIDSno	88110	7.041
NIAID AIDSno	88111	7.167
NIAID AIDSno	88112	6.742

NIAID AIDSno	88114	6.959
NIAID AIDSno	88115	6.551
NIAID AIDSno	88116	6.558
NIAID AIDSno	88117	6.889
NIAID AIDSno	88333	7.237
NIAID AIDSno	88334	7.602
NIAID AIDSno	88335	7.921
NIAID AIDSno	88336	7.921
NIAID AIDSno	88337	5.770
NIAID AIDSno	88338	7.041
NIAID AIDSno	88339	6.788
NIAID AIDSno	88340	7.081
NIAID AIDSno	88341	7.745
NIAID AIDSno	88342	7.796
NIAID AIDSno	88344	6.409
NIAID AIDSno	88345	6.886
NIAID AIDSno	88346	7.538
NIAID AIDSno	88347	5.721
NIAID AIDSno	88348	5.638
NIAID AIDSno	88349	7.824
NIAID AIDSno	88350	7.620
NIAID AIDSno	88351	6.839
NIAID AIDSno	88352	6.066
NIAID AIDSno	88353	7.260
NIAID AIDSno	88354	5.886
NIAID AIDSno	88355	7.367
NIAID AIDSno	88356	6.658
NIAID AIDSno	88357	9.620
NIAID AIDSno	88358	6.260
NIAID AIDSno	88359	6.513
NIAID AIDSno	88360	5.721
NIAID AIDSno	88361	6.387
NIAID AIDSno	88362	6.387
NIAID AIDSno	88363	5.268
NIAID AIDSno	88364	6.523
NIAID AIDSno	88365	4.783
NIAID AIDSno	88366	6.187
NIAID AIDSno	88367	7.284
NIAID AIDSno	88368	5.276
NIAID AIDSno	88369	6.866
NIAID AIDSno	88370	6.292
NIAID AIDSno	88371	7.319
NIAID AIDSno	88372	5.854
NIAID AIDSno	88373	5.839
NIAID AIDSno	88374	6.215

NIAID AIDSno	88375	7.824
NIAID AIDSno	88376	6.252
NIAID AIDSno	88377	7.387
NIAID AIDSno	88410	5.215
NIAID AIDSno	88412	4.983
NIAID AIDSno	88413	5.097
NIAID AIDSno	88416	5.335
NIAID AIDSno	88417	4.445
NIAID AIDSno	88418	4.608
NIAID AIDSno	88420	4.617
NIAID AIDSno	88658	3.177
NIAID AIDSno	88685	6.409
NIAID AIDSno	88717	5.060
NIAID AIDSno	88718	6.081
NIAID AIDSno	88877	5.824
NIAID AIDSno	88884	3.111
NIAID AIDSno	89101	5.000
NIAID AIDSno	89266	7.097
NIAID AIDSno	89268	7.301
NIAID AIDSno	89269	6.959
NIAID AIDSno	89270	6.770
NIAID AIDSno	89271	6.553
NIAID AIDSno	89272	6.222
NIAID AIDSno	89273	6.222
NIAID AIDSno	89274	6.301
NIAID AIDSno	89275	6.523
NIAID AIDSno	89542	7.060
NIAID AIDSno	89543	7.284
NIAID AIDSno	89544	7.310
NIAID AIDSno	89545	7.131
NIAID AIDSno	92008	5.804
NIAID AIDSno	92323	4.625
NIAID AIDSno	92362	4.577
NIAID AIDSno	92462	6.699
NIAID AIDSno	92463	6.046
NIAID AIDSno	92464	4.057
NIAID AIDSno	92505	5.597
NIAID AIDSno	92588	7.268
NIAID AIDSno	92589	7.208
NIAID AIDSno	92590	7.108
NIAID AIDSno	92591	7.143
NIAID AIDSno	92592	7.229
NIAID AIDSno	92593	6.907
NIAID AIDSno	92594	7.301
NIAID AIDSno	92595	6.900

NIAID AIDSno	92596	6.991
NIAID AIDSno	92597	7.097
NIAID AIDSno	92598	6.397
NIAID AIDSno	92599	6.660
NIAID AIDSno	92600	6.845
NIAID AIDSno	92601	7.409
NIAID AIDSno	92602	6.780
NIAID AIDSno	92603	6.625
NIAID AIDSno	93126	6.747
NIAID AIDSno	94263	3.523
NIAID AIDSno	94264	3.523
NIAID AIDSno	94265	3.523
NIAID AIDSno	94266	3.523
NIAID AIDSno	94267	3.523
NIAID AIDSno	94268	3.523
NIAID AIDSno	94269	3.523
NIAID AIDSno	94270	3.523
NIAID AIDSno	94300	4.960
NIAID AIDSno	94312	5.854
NIAID AIDSno	94678	5.301
NIAID AIDSno	94679	6.409
NIAID AIDSno	94681	7.377
NIAID AIDSno	94722	3.481
NIAID AIDSno	94723	3.481
NIAID AIDSno	94729	3.481
NIAID AIDSno	94740	3.481
NIAID AIDSno	95028	5.796
NIAID AIDSno	95029	5.921
NIAID AIDSno	95030	4.886
NIAID AIDSno	95178	4.458
NIAID AIDSno	95188	5.347
NIAID AIDSno	95189	4.522
NIAID AIDSno	95204	4.919
NIAID AIDSno	96006	4.167
NIAID AIDSno	96262	7.000
NIAID AIDSno	96268	7.000
NIAID AIDSno	96271	6.699
NIAID AIDSno	96275	7.188
NIAID AIDSno	96416	5.699
NIAID AIDSno	96417	5.523
NIAID AIDSno	96418	5.046
NIAID AIDSno	96427	3.097
NIAID AIDSno	96975	7.301
NIAID AIDSno	97767	5.337
NIAID AIDSno	98045	6.013

NIAID AIDSno	104167	3.824
NIAID AIDSno	104168	3.839
NIAID AIDSno	104171	3.745
NIAID AIDSno	104172	3.699
NIAID AIDSno	104178	3.824
NIAID AIDSno	104179	3.824
NIAID AIDSno	104184	4.553
NIAID AIDSno	104359	7.324
NIAID AIDSno	104384	6.301
NIAID AIDSno	104385	6.301
NIAID AIDSno	104386	6.301
NIAID AIDSno	104387	6.301
NIAID AIDSno	104389	5.000
NIAID AIDSno	104393	5.000
NIAID AIDSno	104394	5.000
NIAID AIDSno	104404	6.222
NIAID AIDSno	104409	6.097
NIAID AIDSno	104412	5.000
NIAID AIDSno	104423	5.921
NIAID AIDSno	104433	5.000
NIAID AIDSno	104442	5.000
NIAID AIDSno	104447	6.658
NIAID AIDSno	104467	5.000
NIAID AIDSno	104468	5.000
NIAID AIDSno	104471	6.000
NIAID AIDSno	104481	6.357
NIAID AIDSno	104484	5.824
NIAID AIDSno	104874	4.886
NIAID AIDSno	104875	5.347
NIAID AIDSno	104876	6.699
NIAID AIDSno	104877	5.854
NIAID AIDSno	104878	6.222
NIAID AIDSno	104880	5.137
NIAID AIDSno	104881	5.229
NIAID AIDSno	104882	6.398
NIAID AIDSno	104884	4.921
NIAID AIDSno	104885	6.699
NIAID AIDSno	104887	5.301
NIAID AIDSno	104889	5.222
NIAID AIDSno	104890	5.745
NIAID AIDSno	104892	7.839
NIAID AIDSno	104894	8.523
NIAID AIDSno	104895	8.155
NIAID AIDSno	104916	6.845
NIAID AIDSno	104917	6.613

NIAID AIDSno	104918	6.592
NIAID AIDSno	104919	6.260
NIAID AIDSno	104920	5.812
NIAID AIDSno	104921	5.943
NIAID AIDSno	104922	6.754
NIAID AIDSno	104923	4.807
NIAID AIDSno	104924	7.638
NIAID AIDSno	104925	7.699
NIAID AIDSno	104926	8.523
NIAID AIDSno	104927	7.022
NIAID AIDSno	104928	7.796
NIAID AIDSno	104929	5.706
NIAID AIDSno	104973	8.523
NIAID AIDSno	104974	8.523
NIAID AIDSno	104975	8.523
NIAID AIDSno	104976	8.523
NIAID AIDSno	104977	8.523
NIAID AIDSno	104978	8.523
NIAID AIDSno	104979	8.523
NIAID AIDSno	104980	8.523
NIAID AIDSno	104981	8.523
NIAID AIDSno	104982	8.523
NIAID AIDSno	104983	8.412
NIAID AIDSno	104984	8.523
NIAID AIDSno	104985	8.523
NIAID AIDSno	104986	8.523
NIAID AIDSno	104988	8.523
NIAID AIDSno	104989	8.523
NIAID AIDSno	104990	8.523
NIAID AIDSno	104991	8.523
NIAID AIDSno	104992	8.523
NIAID AIDSno	104993	8.523
NIAID AIDSno	105156	7.523
NIAID AIDSno	105189	5.959
NIAID AIDSno	105256	7.155
NIAID AIDSno	105257	6.842
NIAID AIDSno	105258	6.857
NIAID AIDSno	105259	7.268
NIAID AIDSno	105260	6.759
NIAID AIDSno	105261	7.046
NIAID AIDSno	105262	6.613
NIAID AIDSno	105263	6.914
NIAID AIDSno	105265	4.770
NIAID AIDSno	105266	4.357
NIAID AIDSno	105267	4.921

NIAID AIDSno	105268	4.585
NIAID AIDSno	105269	4.593
NIAID AIDSno	105270	5.091
NIAID AIDSno	105271	4.614
NIAID AIDSno	105272	4.932
NIAID AIDSno	105273	4.444
NIAID AIDSno	105274	6.114
NIAID AIDSno	105275	6.738
NIAID AIDSno	105276	6.979
NIAID AIDSno	105277	6.726
NIAID AIDSno	105278	6.889
NIAID AIDSno	105279	5.870
NIAID AIDSno	105280	6.213
NIAID AIDSno	105281	5.215
NIAID AIDSno	105282	4.386
NIAID AIDSno	105283	5.710
NIAID AIDSno	105284	5.569
NIAID AIDSno	105285	5.415
NIAID AIDSno	105286	5.735
NIAID AIDSno	105293	7.887
NIAID AIDSno	105317	0.387
NIAID AIDSno	105717	6.886
NIAID AIDSno	105723	5.843
NIAID AIDSno	105724	5.978
NIAID AIDSno	105725	6.357
NIAID AIDSno	105726	5.804
NIAID AIDSno	105727	6.359
NIAID AIDSno	105728	6.889
NIAID AIDSno	105729	5.887
NIAID AIDSno	105730	5.751
NIAID AIDSno	105731	6.046
NIAID AIDSno	105732	5.992
NIAID AIDSno	105733	5.837
NIAID AIDSno	105734	6.385
NIAID AIDSno	105735	6.618
NIAID AIDSno	105903	7.215
NIAID AIDSno	105905	6.292
NIAID AIDSno	105906	7.009
NIAID AIDSno	105907	6.569
NIAID AIDSno	105908	6.387
NIAID AIDSno	105909	6.886
NIAID AIDSno	105910	6.854
NIAID AIDSno	105911	6.602
NIAID AIDSno	105912	6.854
NIAID AIDSno	105913	4.301

NIAID AIDSno	106290	4.432
NIAID AIDSno	106291	4.538
NIAID AIDSno	106292	4.423
NIAID AIDSno	106293	4.159
NIAID AIDSno	106294	4.467
NIAID AIDSno	106321	6.398
NIAID AIDSno	106322	4.000
NIAID AIDSno	106323	4.000
NIAID AIDSno	107272	3.046
NIAID AIDSno	107885	5.699
NIAID AIDSno	108284	5.659
NIAID AIDSno	108387	5.060
NIAID AIDSno	108414	5.569
NIAID AIDSno	108418	8.000
NIAID AIDSno	108430	5.824
NIAID AIDSno	108433	6.018
NIAID AIDSno	108434	5.244
NIAID AIDSno	108435	5.143
NIAID AIDSno	108436	4.796
NIAID AIDSno	108437	4.921
NIAID AIDSno	108439	4.824
NIAID AIDSno	108440	4.921
NIAID AIDSno	108441	5.041
NIAID AIDSno	108442	5.959
NIAID AIDSno	108444	5.149
NIAID AIDSno	108446	5.456
NIAID AIDSno	108447	5.959
NIAID AIDSno	108448	6.921
NIAID AIDSno	108449	5.770
NIAID AIDSno	108450	6.854
NIAID AIDSno	108451	4.886
NIAID AIDSno	108452	4.921
NIAID AIDSno	108453	4.721
NIAID AIDSno	108456	5.000
NIAID AIDSno	108459	5.319
NIAID AIDSno	108461	5.004
NIAID AIDSno	108465	6.301
NIAID AIDSno	108466	5.208
NIAID AIDSno	108467	6.284
NIAID AIDSno	108468	6.046
NIAID AIDSno	108470	5.161
NIAID AIDSno	108471	5.276
NIAID AIDSno	108472	6.523
NIAID AIDSno	108473	7.398
NIAID AIDSno	108476	6.155

NIAID AIDSno	108477	6.000
NIAID AIDSno	108478	7.523
NIAID AIDSno	108643	8.523
NIAID AIDSno	108649	8.328
NIAID AIDSno	108653	8.770
NIAID AIDSno	108658	8.745
NIAID AIDSno	108666	5.658
NIAID AIDSno	109132	5.538
NIAID AIDSno	109133	5.650
NIAID AIDSno	109134	5.547
NIAID AIDSno	109139	5.481
NIAID AIDSno	109140	6.016
NIAID AIDSno	109141	6.374
NIAID AIDSno	109142	5.600
NIAID AIDSno	109143	6.365
NIAID AIDSno	109144	6.845
NIAID AIDSno	109146	6.408
NIAID AIDSno	109147	6.097
NIAID AIDSno	109148	6.224
NIAID AIDSno	109149	6.629
NIAID AIDSno	109150	6.367
NIAID AIDSno	109151	6.355
NIAID AIDSno	109154	6.714
NIAID AIDSno	109155	6.967
NIAID AIDSno	109156	7.337
NIAID AIDSno	109157	6.733
NIAID AIDSno	109158	6.889
NIAID AIDSno	109159	6.821
NIAID AIDSno	109160	6.762
NIAID AIDSno	109436	9.000
NIAID AIDSno	109940	3.481
NIAID AIDSno	109947	3.481
NIAID AIDSno	109952	4.481
NIAID AIDSno	109953	4.481
NIAID AIDSno	109964	4.481
NIAID AIDSno	109967	3.481
NIAID AIDSno	110065	4.046
NIAID AIDSno	110287	4.244
NIAID AIDSno	110291	5.631
NIAID AIDSno	110293	5.611
NIAID AIDSno	110294	5.023
NIAID AIDSno	110296	5.703
NIAID AIDSno	111109	6.222
NIAID AIDSno	111110	7.000
NIAID AIDSno	111111	5.907

NIAID AIDSno	111112	6.036
NIAID AIDSno	111113	4.903
NIAID AIDSno	111115	5.000
NIAID AIDSno	111117	5.367
NIAID AIDSno	111266	5.638
NIAID AIDSno	111267	5.237
NIAID AIDSno	111269	5.678
NIAID AIDSno	111271	6.398
NIAID AIDSno	111272	5.585
NIAID AIDSno	111276	6.699
NIAID AIDSno	111281	7.412
NIAID AIDSno	111283	7.699
NIAID AIDSno	111284	8.122
NIAID AIDSno	111285	7.604
NIAID AIDSno	111286	8.523
NIAID AIDSno	111291	5.638
NIAID AIDSno	111294	5.432
NIAID AIDSno	111295	6.097
NIAID AIDSno	111297	5.854
NIAID AIDSno	111313	6.456
NIAID AIDSno	111314	5.796
NIAID AIDSno	111315	5.194
NIAID AIDSno	111334	6.095
NIAID AIDSno	111335	6.287
NIAID AIDSno	111336	6.231
NIAID AIDSno	111341	5.180
NIAID AIDSno	111342	3.684
NIAID AIDSno	111350	5.244
NIAID AIDSno	111630	7.886
NIAID AIDSno	112306	5.314
NIAID AIDSno	112307	5.107
NIAID AIDSno	112308	4.780
NIAID AIDSno	112309	4.701
NIAID AIDSno	112312	4.429
NIAID AIDSno	112313	4.016
NIAID AIDSno	112314	5.596
NIAID AIDSno	112322	5.417
NIAID AIDSno	112324	4.440
NIAID AIDSno	112325	4.342
NIAID AIDSno	112328	4.223
NIAID AIDSno	112330	4.969
NIAID AIDSno	112352	5.479
NIAID AIDSno	112375	6.000
NIAID AIDSno	112391	6.000
NIAID AIDSno	112392	7.131

NIAID AIDSno	112393	4.000
NIAID AIDSno	112394	6.298
NIAID AIDSno	112395	4.000
NIAID AIDSno	112396	4.055
NIAID AIDSno	112397	5.757
NIAID AIDSno	112398	4.000
NIAID AIDSno	112400	4.000
NIAID AIDSno	112401	4.000
NIAID AIDSno	112469	4.000
NIAID AIDSno	112481	4.000
NIAID AIDSno	112489	4.000
NIAID AIDSno	112495	4.000
NIAID AIDSno	112500	4.000
NIAID AIDSno	112571	4.523
NIAID AIDSno	113349	5.097
NIAID AIDSno	113411	5.481
NIAID AIDSno	113413	5.100
NIAID AIDSno	113415	5.658
NIAID AIDSno	113416	5.367
NIAID AIDSno	113417	5.481
NIAID AIDSno	113421	5.409
NIAID AIDSno	113507	6.237
NIAID AIDSno	113509	6.174
NIAID AIDSno	113511	6.347
NIAID AIDSno	113513	5.987
NIAID AIDSno	113515	5.932
NIAID AIDSno	113517	4.921
NIAID AIDSno	113519	4.398
NIAID AIDSno	113521	6.051
NIAID AIDSno	113523	6.357
NIAID AIDSno	113525	4.991
NIAID AIDSno	113527	5.917
NIAID AIDSno	113997	10.222
NIAID AIDSno	114547	4.620
NIAID AIDSno	114548	4.143
NIAID AIDSno	114549	4.347
NIAID AIDSno	115880	7.602
NIAID AIDSno	115890	6.155
NIAID AIDSno	115891	4.824
NIAID AIDSno	115893	5.699
NIAID AIDSno	115894	4.699
NIAID AIDSno	115896	5.097
NIAID AIDSno	115897	5.000
NIAID AIDSno	115901	6.699
NIAID AIDSno	115902	4.602

NIAID AIDSno	121173	6.699
NIAID AIDSno	121174	6.398
NIAID AIDSno	121175	6.222
NIAID AIDSno	121176	6.523
NIAID AIDSno	121177	6.301
NIAID AIDSno	121178	6.301
NIAID AIDSno	121179	6.523
NIAID AIDSno	122000	5.793
NIAID AIDSno	122002	5.580
NIAID AIDSno	122003	5.000
NIAID AIDSno	122005	5.241
NIAID AIDSno	122007	8.137
NIAID AIDSno	122008	6.495
NIAID AIDSno	122009	4.757
NIAID AIDSno	122010	6.553
NIAID AIDSno	122011	4.262
NIAID AIDSno	122012	5.306
NIAID AIDSno	122013	4.398
NIAID AIDSno	122014	3.802
NIAID AIDSno	122043	6.745
NIAID AIDSno	122044	8.097
NIAID AIDSno	122045	6.398
NIAID AIDSno	122047	8.398
NIAID AIDSno	122048	8.000
NIAID AIDSno	122049	8.523
NIAID AIDSno	122050	7.268
NIAID AIDSno	122051	7.097
NIAID AIDSno	122052	7.328
NIAID AIDSno	122054	8.222
NIAID AIDSno	122055	8.523
NIAID AIDSno	122092	7.222
NIAID AIDSno	122094	6.996
NIAID AIDSno	122096	4.301
NIAID AIDSno	122099	7.721
NIAID AIDSno	122102	7.174
NIAID AIDSno	122103	6.883
NIAID AIDSno	122106	7.824
NIAID AIDSno	122107	7.301
NIAID AIDSno	122108	6.419
NIAID AIDSno	122109	7.086
NIAID AIDSno	122146	6.550
NIAID AIDSno	122147	6.305
NIAID AIDSno	122148	6.851
NIAID AIDSno	122149	6.465
NIAID AIDSno	122172	4.301

NIAID AIDSno	122173	7.721
NIAID AIDSno	122176	6.726
NIAID AIDSno	122177	7.770
NIAID AIDSno	122178	6.721
NIAID AIDSno	122181	6.312
NIAID AIDSno	122182	7.569
NIAID AIDSno	122187	7.721
NIAID AIDSno	122188	7.229
NIAID AIDSno	122191	7.143
NIAID AIDSno	122192	7.143
NIAID AIDSno	122193	7.638
NIAID AIDSno	122194	6.815
NIAID AIDSno	122195	6.842
NIAID AIDSno	122196	6.757
NIAID AIDSno	122197	6.693
NIAID AIDSno	122199	7.032
NIAID AIDSno	122201	7.000
NIAID AIDSno	122202	7.060
NIAID AIDSno	122203	7.155
NIAID AIDSno	122207	6.462
NIAID AIDSno	122208	6.633
NIAID AIDSno	122209	6.674
NIAID AIDSno	122210	7.108
NIAID AIDSno	122213	6.363
NIAID AIDSno	122214	7.509
NIAID AIDSno	122215	6.951
NIAID AIDSno	122248	5.301
NIAID AIDSno	122370	4.824
NIAID AIDSno	122703	4.103
NIAID AIDSno	122862	4.046
NIAID AIDSno	122886	5.319
NIAID AIDSno	123395	5.276
NIAID AIDSno	123400	5.057
NIAID AIDSno	123427	8.119
NIAID AIDSno	125707	3.257
NIAID AIDSno	131122	4.590
NIAID AIDSno	141533	4.459
NIAID AIDSno	141559	5.219
NIAID AIDSno	141571	4.073
NIAID AIDSno	146625	4.883
NIAID AIDSno	153705	6.000
NIAID AIDSno	153864	3.850
NIAID AIDSno	153866	3.226
NIAID AIDSno	153868	3.174
NIAID AIDSno	154308	6.699

NIAID AIDSno	154309	6.155
NIAID AIDSno	154310	6.097
NIAID AIDSno	154316	6.347
NIAID AIDSno	154332	6.036
NIAID AIDSno	154333	5.385
NIAID AIDSno	154334	6.041
NIAID AIDSno	154471	6.301
NIAID AIDSno	154474	6.222
NIAID AIDSno	155605	5.523
NIAID AIDSno	155607	4.910
NIAID AIDSno	155608	5.921
NIAID AIDSno	155609	5.252
NIAID AIDSno	155611	5.699
NIAID AIDSno	159520	7.538
NIAID AIDSno	160908	5.003
NIAID AIDSno	161915	9.000
NIAID AIDSno	162728	4.174
NIAID AIDSno	162729	4.247
NIAID AIDSno	162731	4.212
NIAID AIDSno	162734	5.385
NIAID AIDSno	162735	4.373
NIAID AIDSno	162736	5.545
NIAID AIDSno	164439	4.310
NIAID AIDSno	164455	4.745
NIAID AIDSno	164467	5.201
NIAID AIDSno	164468	5.553
NIAID AIDSno	164469	5.347
NIAID AIDSno	165196	5.222
NIAID AIDSno	165197	3.588
NIAID AIDSno	165198	4.004
NIAID AIDSno	165202	6.633
NIAID AIDSno	165203	6.742
NIAID AIDSno	165204	6.445
NIAID AIDSno	165205	5.081
NIAID AIDSno	165206	4.527
NIAID AIDSno	165207	6.824
NIAID AIDSno	165209	4.219
NIAID AIDSno	165210	4.150
NIAID AIDSno	165211	6.163
NIAID AIDSno	165212	5.248
NIAID AIDSno	165214	5.148
NIAID AIDSno	165223	5.354
NIAID AIDSno	165224	5.307
NIAID AIDSno	165225	5.622
NIAID AIDSno	165226	5.714

NIAID AIDSno	165227	7.796
NIAID AIDSno	165236	4.184
NIAID AIDSno	165237	5.530
NIAID AIDSno	165238	5.024
NIAID AIDSno	165239	6.302
NIAID AIDSno	165544	5.708
NIAID AIDSno	165545	5.947
NIAID AIDSno	165557	6.066
NIAID AIDSno	165929	5.638
NIAID AIDSno	165930	5.602
NIAID AIDSno	165931	5.699
NIAID AIDSno	166195	4.903
NIAID AIDSno	166206	7.155
NIAID AIDSno	166208	7.398
NIAID AIDSno	166211	7.523
NIAID AIDSno	166213	7.489
NIAID AIDSno	166214	5.602
NIAID AIDSno	166227	7.097
NIAID AIDSno	166235	6.097
NIAID AIDSno	167802	4.046
NIAID AIDSno	169030	7.824
NIAID AIDSno	170251	7.143
NIAID AIDSno	170257	8.523
NIAID AIDSno	180878	4.235
NIAID AIDSno	184193	5.319
NIAID AIDSno	184600	4.000
NIAID AIDSno	184601	4.097
NIAID AIDSno	184602	4.432
NIAID AIDSno	184603	4.237
NIAID AIDSno	184604	4.000
NIAID AIDSno	184605	4.097
NIAID AIDSno	184606	4.456
NIAID AIDSno	184607	4.301
NIAID AIDSno	184608	4.569
NIAID AIDSno	184610	4.229
NIAID AIDSno	184612	3.886
NIAID AIDSno	184613	4.620
NIAID AIDSno	184614	4.523
NIAID AIDSno	184615	3.495
NIAID AIDSno	184617	4.215
NIAID AIDSno	184619	4.572
NIAID AIDSno	184620	4.114
NIAID AIDSno	184621	4.149
NIAID AIDSno	184622	4.066
NIAID AIDSno	184623	4.301

NIAID AIDSno	184624	5.444
NIAID AIDSno	184625	4.928
NIAID AIDSno	184626	4.796
NIAID AIDSno	184627	4.921
NIAID AIDSno	184628	4.721
NIAID AIDSno	184629	7.222
NIAID AIDSno	184630	7.585
NIAID AIDSno	184637	8.714
NIAID AIDSno	184727	4.046
NIAID AIDSno	184849	4.000
NIAID AIDSno	189308	4.547
NIAID AIDSno	189310	4.396
NIAID AIDSno	189320	6.081
NIAID AIDSno	189334	5.013
NIAID AIDSno	189862	5.228
NIAID AIDSno	190295	7.046
NIAID AIDSno	190296	6.767
NIAID AIDSno	190297	6.284
NIAID AIDSno	190298	6.137
NIAID AIDSno	190299	5.620
NIAID AIDSno	190300	5.936
NIAID AIDSno	190301	5.720
NIAID AIDSno	190302	5.476
NIAID AIDSno	190303	5.000
NIAID AIDSno	190304	5.000
NIAID AIDSno	190305	5.717
NIAID AIDSno	190306	6.188
NIAID AIDSno	190307	6.504
NIAID AIDSno	190308	6.216
NIAID AIDSno	190309	6.939
NIAID AIDSno	190310	7.208
NIAID AIDSno	190311	7.495
NIAID AIDSno	190312	6.146
NIAID AIDSno	190313	7.357
NIAID AIDSno	190314	5.101
NIAID AIDSno	190315	6.280
NIAID AIDSno	190316	7.004
NIAID AIDSno	190317	6.738
NIAID AIDSno	190318	6.363
NIAID AIDSno	190319	6.686
NIAID AIDSno	190320	6.676
NIAID AIDSno	190321	7.509
NIAID AIDSno	190322	6.286
NIAID AIDSno	190323	7.292
NIAID AIDSno	190324	6.910

NIAID AIDSno	190325	7.215
NIAID AIDSno	190326	5.200
NIAID AIDSno	190327	6.602
NIAID AIDSno	191076	8.658
NIAID AIDSno	191077	7.569
NIAID AIDSno	191078	7.959
NIAID AIDSno	191079	8.155
NIAID AIDSno	191080	7.585
NIAID AIDSno	191081	8.222
NIAID AIDSno	191082	8.097
NIAID AIDSno	191083	8.161
NIAID AIDSno	191084	8.377
NIAID AIDSno	191085	7.921
NIAID AIDSno	195742	4.886
NIAID AIDSno	195770	3.745
NIAID AIDSno	208921	4.637
NIAID AIDSno	208922	4.853
NIAID AIDSno	208923	5.443
NIAID AIDSno	208924	4.677
NIAID AIDSno	208925	4.920
NIAID AIDSno	208926	5.266
NIAID AIDSno	208927	4.885
NIAID AIDSno	208928	4.637
NIAID AIDSno	208929	6.075
NIAID AIDSno	208930	4.720
NIAID AIDSno	208931	5.468
NIAID AIDSno	208932	5.221
NIAID AIDSno	208933	5.958
NIAID AIDSno	208934	4.148
NIAID AIDSno	208935	4.720
NIAID AIDSno	208936	5.584
NIAID AIDSno	208937	5.568
NIAID AIDSno	208938	4.920
NIAID AIDSno	208939	4.885
NIAID AIDSno	208940	5.243
NIAID AIDSno	208941	4.999
NIAID AIDSno	208942	4.468
NIAID AIDSno	208943	4.085
NIAID AIDSno	208944	4.657
NIAID AIDSno	208945	6.583
NIAID AIDSno	208946	5.885
NIAID AIDSno	208947	6.656
NIAID AIDSno	208948	5.102
NIAID AIDSno	208949	5.136
NIAID AIDSno	208950	4.999

NIAID AIDSno	208951	5.601
NIAID AIDSno	208952	5.180
NIAID AIDSno	208953	4.744
NIAID AIDSno	208954	6.959
NIAID AIDSno	208955	8.097
NIAID AIDSno	208956	8.301
NIAID AIDSno	208957	7.367
NIAID AIDSno	208958	5.468
NIAID AIDSno	208959	5.677
NIAID AIDSno	208960	5.443
NIAID AIDSno	208961	5.327
NIAID AIDSno	208962	7.056
NIAID AIDSno	208963	8.398
NIAID AIDSno	208964	7.886
NIAID AIDSno	208965	6.656
NIAID AIDSno	208966	6.455
NIAID AIDSno	208967	8.097
NIAID AIDSno	208968	8.155
NIAID AIDSno	208969	7.108
NIAID AIDSno	208970	7.921
NIAID AIDSno	208971	7.041
NIAID AIDSno	208972	7.854
NIAID AIDSno	208973	8.155
NIAID AIDSno	208974	7.022
NIAID AIDSno	208975	6.467
NIAID AIDSno	208976	5.397
NIAID AIDSno	208977	6.346
NIAID AIDSno	208978	7.018
NIAID AIDSno	208979	5.657
NIAID AIDSno	208980	5.920
NIAID AIDSno	208981	6.455
NIAID AIDSno	208982	7.886
NIAID AIDSno	208983	7.387
NIAID AIDSno	208984	7.201
NIAID AIDSno	208985	8.523
NIAID AIDSno	208986	8.398
NIAID AIDSno	208987	9.000
NIAID AIDSno	208988	7.377
NIAID AIDSno	208989	6.600
NIAID AIDSno	208990	7.284
NIAID AIDSno	208991	5.154
NIAID AIDSno	208992	4.744
NIAID AIDSno	208993	5.481
NIAID AIDSno	208994	7.229
NIAID AIDSno	208995	5.060

NIAID AIDSno	208996	6.480
NIAID AIDSno	208997	7.585
NIAID AIDSno	208998	7.721
NIAID AIDSno	208999	8.301
NIAID AIDSno	209000	8.523
NIAID AIDSno	209001	7.921
NIAID AIDSno	209002	7.000
NIAID AIDSno	209003	7.201
NIAID AIDSno	209004	6.678
NIAID AIDSno	209005	6.008
NIAID AIDSno	209787	6.054
NIAID AIDSno	209789	6.428
NIAID AIDSno	209791	5.703
NIAID AIDSno	209793	4.777
NIAID AIDSno	209795	5.295
NIAID AIDSno	209799	5.000
NIAID AIDSno	209803	4.411
NIAID AIDSno	209804	5.268
NIAID AIDSno	209805	5.469
NIAID AIDSno	209806	6.097
NIAID AIDSno	209807	4.883
NIAID AIDSno	209809	4.757
NIAID AIDSno	209810	5.237
NIAID AIDSno	209811	6.097
NIAID AIDSno	209815	5.824
NIAID AIDSno	209816	5.244
NIAID AIDSno	209817	5.060
NIAID AIDSno	209820	5.770
NIAID AIDSno	209823	5.921
NIAID AIDSno	209825	5.854
NIAID AIDSno	209829	4.886
NIAID AIDSno	209852	6.292
NIAID AIDSno	209853	4.854
NIAID AIDSno	209894	6.699
NIAID AIDSno	210411	4.902
NIAID AIDSno	210602	6.420
NIAID AIDSno	212721	7.638
NIAID AIDSno	212728	4.959
NIAID AIDSno	212729	5.328
NIAID AIDSno	212730	5.201
NIAID AIDSno	212731	5.420
NIAID AIDSno	212732	5.432
NIAID AIDSno	212755	7.979
NIAID AIDSno	212756	8.260
NIAID AIDSno	212757	7.783

NIAID AIDSno	212758	7.959
NIAID AIDSno	212759	7.721
NIAID AIDSno	212760	7.602
NIAID AIDSno	212761	8.009
NIAID AIDSno	212762	8.000
NIAID AIDSno	212763	7.854
NIAID AIDSno	212764	7.387
NIAID AIDSno	212765	8.022
NIAID AIDSno	212766	7.921
NIAID AIDSno	212767	7.824
NIAID AIDSno	212768	7.215
NIAID AIDSno	212769	7.854
NIAID AIDSno	212770	7.959
NIAID AIDSno	212771	8.155
NIAID AIDSno	212772	8.000
NIAID AIDSno	212773	7.770
NIAID AIDSno	212774	7.347
NIAID AIDSno	212775	8.215
NIAID AIDSno	212776	8.252
NIAID AIDSno	212777	8.097
NIAID AIDSno	212778	8.051
NIAID AIDSno	212779	7.420
NIAID AIDSno	212780	7.721
NIAID AIDSno	212781	7.456
NIAID AIDSno	212782	7.569
NIAID AIDSno	212853	7.000
NIAID AIDSno	212855	7.854
NIAID AIDSno	214666	5.000
NIAID AIDSno	214667	4.398
NIAID AIDSno	214978	4.654
NIAID AIDSno	215962	5.097
NIAID AIDSno	215964	5.523
NIAID AIDSno	215965	5.260
NIAID AIDSno	215966	5.462
NIAID AIDSno	215967	5.509
NIAID AIDSno	215969	5.834
NIAID AIDSno	215971	5.810
NIAID AIDSno	215972	5.538
NIAID AIDSno	215973	5.509
NIAID AIDSno	215975	5.097
NIAID AIDSno	215976	5.180
NIAID AIDSno	215978	3.485
NIAID AIDSno	215979	5.495
NIAID AIDSno	215980	4.886
NIAID AIDSno	215984	5.904

NIAID AIDSno	215986	4.523
NIAID AIDSno	215987	5.347
NIAID AIDSno	215989	5.540
NIAID AIDSno	215992	5.757
NIAID AIDSno	215993	6.658
NIAID AIDSno	215995	6.757
NIAID AIDSno	218023	4.821
NIAID AIDSno	218024	5.237
NIAID AIDSno	218025	4.896
NIAID AIDSno	218026	4.876
NIAID AIDSno	218027	5.523
NIAID AIDSno	218028	5.149
NIAID AIDSno	218029	4.688
NIAID AIDSno	219833	7.509
NIAID AIDSno	219834	7.222
NIAID AIDSno	219835	7.201
NIAID AIDSno	219836	7.770
NIAID AIDSno	219837	7.638
NIAID AIDSno	219838	7.658
NIAID AIDSno	219839	7.745
NIAID AIDSno	219840	8.000
NIAID AIDSno	219841	7.824
NIAID AIDSno	219842	7.721
NIAID AIDSno	219843	7.824
NIAID AIDSno	220097	5.747
NIAID AIDSno	220101	6.060
NIAID AIDSno	220102	5.602
NIAID AIDSno	220104	6.013
NIAID AIDSno	220105	6.071
NIAID AIDSno	220390	7.301
NIAID AIDSno	220392	7.301
NIAID AIDSno	220393	7.301
NIAID AIDSno	220394	7.301
NIAID AIDSno	220408	7.301
NIAID AIDSno	220409	7.301
NIAID AIDSno	220412	7.301
NIAID AIDSno	220415	7.301
NIAID AIDSno	220416	7.301
NIAID AIDSno	220418	7.301
NIAID AIDSno	220419	7.301
NIAID AIDSno	220423	7.921
NIAID AIDSno	220428	7.770
NIAID AIDSno	220431	7.678
NIAID AIDSno	220433	7.745
NIAID AIDSno	220435	7.796

NIAID AIDSno	220463	7.886
NIAID AIDSno	220464	7.620
NIAID AIDSno	220486	7.301
NIAID AIDSno	220487	8.131
NIAID AIDSno	220488	8.046
NIAID AIDSno	220503	8.260
NIAID AIDSno	220504	7.886
NIAID AIDSno	220513	8.420
NIAID AIDSno	220514	8.260
NIAID AIDSno	220518	8.553
NIAID AIDSno	220572	7.301
NIAID AIDSno	221978	4.611
NIAID AIDSno	222516	5.900
NIAID AIDSno	222538	7.301
NIAID AIDSno	222543	5.022
NIAID AIDSno	222544	5.699
NIAID AIDSno	222545	5.097
NIAID AIDSno	222547	6.432
NIAID AIDSno	222548	6.569
NIAID AIDSno	222549	5.222
NIAID AIDSno	222551	6.013
NIAID AIDSno	222552	6.071
NIAID AIDSno	222553	6.469
NIAID AIDSno	222555	6.222
NIAID AIDSno	222557	6.222
NIAID AIDSno	222559	7.114
NIAID AIDSno	222560	6.824
NIAID AIDSno	222561	4.409
NIAID AIDSno	222562	6.208
NIAID AIDSno	223008	0.201
NIAID AIDSno	223821	5.780
NIAID AIDSno	223822	5.807
NIAID AIDSno	223823	7.417
NIAID AIDSno	223824	6.188
NIAID AIDSno	223825	5.971
NIAID AIDSno	224387	5.252
NIAID AIDSno	224393	4.387
NIAID AIDSno	224580	6.301
NIAID AIDSno	224753	6.000
NIAID AIDSno	224754	5.678
NIAID AIDSno	224755	6.060
NIAID AIDSno	225416	6.523
NIAID AIDSno	225419	4.301
NIAID AIDSno	225420	5.056
NIAID AIDSno	225422	5.310

NIAID AIDSno	225423	5.796
NIAID AIDSno	225424	5.056
NIAID AIDSno	225426	5.620
NIAID AIDSno	225427	5.201
NIAID AIDSno	225428	7.495
NIAID AIDSno	225429	6.721
NIAID AIDSno	225434	6.745
NIAID AIDSno	225435	7.959
NIAID AIDSno	225436	7.036
NIAID AIDSno	225437	7.745
NIAID AIDSno	225438	8.027
NIAID AIDSno	225439	8.367
NIAID AIDSno	225440	7.921
NIAID AIDSno	225441	8.046
NIAID AIDSno	225442	8.022
NIAID AIDSno	225826	8.301
NIAID AIDSno	226064	5.484
NIAID AIDSno	226099	7.602
NIAID AIDSno	226100	7.824
NIAID AIDSno	226105	5.444
NIAID AIDSno	226165	5.810
NIAID AIDSno	226166	6.699
NIAID AIDSno	226167	5.722
NIAID AIDSno	226168	5.199
NIAID AIDSno	226178	7.385
NIAID AIDSno	227499	8.022
NIAID AIDSno	227507	7.301
NIAID AIDSno	227509	7.301
NIAID AIDSno	227513	7.301
NIAID AIDSno	227516	7.301
NIAID AIDSno	227520	7.745
NIAID AIDSno	227521	7.921
NIAID AIDSno	227522	8.523
NIAID AIDSno	227528	8.523
NIAID AIDSno	227534	7.301
NIAID AIDSno	227539	8.523
NIAID AIDSno	227542	8.398
NIAID AIDSno	227555	7.301
NIAID AIDSno	227575	7.301
NIAID AIDSno	227576	7.301
NIAID AIDSno	227579	7.301
NIAID AIDSno	227581	7.301
NIAID AIDSno	227584	7.301
NIAID AIDSno	227592	7.301
NIAID AIDSno	227607	7.301

NIAID AIDSno	227608	7.301
NIAID AIDSno	227609	7.301
NIAID AIDSno	227621	7.301
NIAID AIDSno	227639	7.301
NIAID AIDSno	227678	8.056
NIAID AIDSno	227681	8.000
NIAID AIDSno	227683	8.509
NIAID AIDSno	227684	8.237
NIAID AIDSno	227696	8.301
NIAID AIDSno	227701	7.301
NIAID AIDSno	228394	5.921
NIAID AIDSno	228400	7.538
NIAID AIDSno	228401	6.000
NIAID AIDSno	228462	6.824
NIAID AIDSno	228516	5.523
NIAID AIDSno	228517	4.602
NIAID AIDSno	228518	5.839
NIAID AIDSno	231385	6.155
NIAID AIDSno	231386	4.222
NIAID AIDSno	231387	4.699
NIAID AIDSno	231388	5.125
NIAID AIDSno	231389	4.790
NIAID AIDSno	231390	4.896
NIAID AIDSno	231391	5.284
NIAID AIDSno	231392	4.301
NIAID AIDSno	231406	5.092
NIAID AIDSno	231407	4.649
NIAID AIDSno	231408	5.225
NIAID AIDSno	231604	5.377
NIAID AIDSno	231616	4.131
NIAID AIDSno	231625	5.097
NIAID AIDSno	257685	5.347
NIAID AIDSno	257687	5.569
NIAID AIDSno	257690	5.387
NIAID AIDSno	257694	5.432
NIAID AIDSno	257696	5.538
NIAID AIDSno	257697	5.469
NIAID AIDSno	257698	5.509
NIAID AIDSno	257699	5.745
NIAID AIDSno	257700	5.602
NIAID AIDSno	257704	5.204
NIAID AIDSno	257705	6.046
NIAID AIDSno	257706	6.000
NIAID AIDSno	257707	6.000
NIAID AIDSno	257708	5.959

NIAID AIDSno	257709	5.870
NIAID AIDSno	257710	5.824
NIAID AIDSno	257711	5.721
NIAID AIDSno	257712	5.678
NIAID AIDSno	257713	5.678
NIAID AIDSno	257714	5.620
NIAID AIDSno	257715	5.569
NIAID AIDSno	257716	5.538
NIAID AIDSno	257717	5.481
NIAID AIDSno	257718	5.469
NIAID AIDSno	257719	5.456
NIAID AIDSno	257720	5.409
NIAID AIDSno	257721	5.409
NIAID AIDSno	257722	5.337
NIAID AIDSno	257723	5.337
NIAID AIDSno	257724	5.301
NIAID AIDSno	257725	5.284
NIAID AIDSno	257726	5.268
NIAID AIDSno	257727	5.102
NIAID AIDSno	257728	5.081
NIAID AIDSno	257729	5.022
NIAID AIDSno	257730	4.947
NIAID AIDSno	257731	4.870
NIAID AIDSno	257732	4.810
NIAID AIDSno	261941	0.523
NIAID AIDSno	262689	6.125
NIAID AIDSno	279353	5.000
NIAID AIDSno	284339	4.547
NIAID AIDSno	284345	5.745
NIAID AIDSno	284357	5.377
NIAID AIDSno	284361	5.108
NIAID AIDSno	284362	5.108
NIAID AIDSno	284369	4.398
NIAID AIDSno	292492	7.046
NIAID AIDSno	292516	5.770
NIAID AIDSno	293206	5.000
NIAID AIDSno	293207	5.000
NIAID AIDSno	293208	5.000
NIAID AIDSno	293213	5.678
NIAID AIDSno	293217	5.932
NIAID AIDSno	293226	4.973
NIAID AIDSno	293234	4.783
NIAID AIDSno	314086	7.022
NIAID AIDSno	314087	7.721
NIAID AIDSno	314088	7.745

NIAID AIDSno	314104	3.364
NIAID AIDSno	335015	4.670
NIAID AIDSno	335867	7.959
NIAID AIDSno	335868	7.602
NIAID AIDSno	339007	5.198
NIAID AIDSno	339181	7.131
NIAID AIDSno	339182	6.000
NIAID AIDSno	339184	4.804
NIAID AIDSno	339185	4.010
NIAID AIDSno	339192	4.257
NIAID AIDSno	339193	6.076
NIAID AIDSno	339194	6.125
NIAID AIDSno	339195	5.120
NIAID AIDSno	339196	6.046
NIAID AIDSno	339197	5.244
NIAID AIDSno	339198	5.086
NIAID AIDSno	339996	1.222
NIAID AIDSno	342636	5.456
NIAID AIDSno	342638	5.620
NIAID AIDSno	342640	5.129
NIAID AIDSno	343654	6.730
NIAID AIDSno	345109	7.473
NIAID AIDSno	345110	6.032
NIAID AIDSno	345111	6.004
NIAID AIDSno	345114	4.308
NIAID AIDSno	345115	6.301
NIAID AIDSno	345116	6.602
NIAID AIDSno	345945	5.602
NIAID AIDSno	345946	5.796
NIAID AIDSno	345947	4.699
NIAID AIDSno	345948	6.409
NIAID AIDSno	345950	8.398
NIAID AIDSno	345951	5.699
NIAID AIDSno	345952	5.553
NIAID AIDSno	345954	3.987
NIAID AIDSno	345955	8.046
NIAID AIDSno	345956	5.699
NIAID AIDSno	345957	5.638
NIAID AIDSno	345958	5.022
NIAID AIDSno	345959	4.602
NIAID AIDSno	345960	5.921
NIAID AIDSno	345961	3.592
NIAID AIDSno	345962	7.585
NIAID AIDSno	345963	7.432
NIAID AIDSno	345964	8.155

NIAID AIDSno	345965	5.523
NIAID AIDSno	345966	6.119
NIAID AIDSno	345971	6.301
NIAID AIDSno	345972	5.456
NIAID AIDSno	345973	8.222
NIAID AIDSno	345976	7.444
NIAID AIDSno	345977	7.301
NIAID AIDSno	345979	6.119
NIAID AIDSno	345980	6.523
NIAID AIDSno	345981	5.921
NIAID AIDSno	345984	6.097
NIAID AIDSno	345985	5.553
NIAID AIDSno	345986	6.824
NIAID AIDSno	345987	7.046
NIAID AIDSno	345988	6.000
NIAID AIDSno	345989	6.602
NIAID AIDSno	345990	5.824
NIAID AIDSno	345991	6.398
NIAID AIDSno	345992	6.398
NIAID AIDSno	345993	5.796
NIAID AIDSno	345995	6.770
NIAID AIDSno	345997	5.481
NIAID AIDSno	346048	5.108
NIAID AIDSno	346049	5.517
NIAID AIDSno	346050	5.762
NIAID AIDSno	346051	4.903
NIAID AIDSno	346052	5.352
NIAID AIDSno	346053	5.582
NIAID AIDSno	346054	4.090
NIAID AIDSno	346055	5.013
NIAID AIDSno	346056	4.548
NIAID AIDSno	346057	4.916
NIAID AIDSno	346058	4.558
NIAID AIDSno	346059	3.515
NIAID AIDSno	346060	3.665
NIAID AIDSno	346061	5.921
NIAID AIDSno	346064	5.119
NIAID AIDSno	346065	3.761
NIAID AIDSno	346066	3.429
NIAID AIDSno	346067	3.444
NIAID AIDSno	347677	9.097
NIAID AIDSno	347678	8.699
NIAID AIDSno	347688	5.033
NIAID AIDSno	347693	4.125
NIAID AIDSno	347694	3.879

NIAID AIDSno	347695	3.538
NIAID AIDSno	347697	3.928
NIAID AIDSno	347699	3.796
NIAID AIDSno	348038	8.301
NIAID AIDSno	348069	7.268
NIAID AIDSno	348082	6.699
NIAID AIDSno	348086	6.783
NIAID AIDSno	348094	5.902
NIAID AIDSno	348109	8.155
NIAID AIDSno	348116	7.509
NIAID AIDSno	348118	8.398
NIAID AIDSno	348121	7.658
NIAID AIDSno	348126	8.349
NIAID AIDSno	348127	8.523
NIAID AIDSno	348128	8.523
NIAID AIDSno	348540	6.000
NIAID AIDSno	348562	6.000
NIAID AIDSno	349532	4.523
NIAID AIDSno	349533	4.222
NIAID AIDSno	349534	4.398
NIAID AIDSno	349549	7.260
NIAID AIDSno	349550	8.301
NIAID AIDSno	349551	7.886
NIAID AIDSno	349552	8.398
NIAID AIDSno	349553	7.638
NIAID AIDSno	349554	8.000
NIAID AIDSno	349555	6.638
NIAID AIDSno	349556	8.046
NIAID AIDSno	350960	5.541
NIAID AIDSno	351819	7.745
NIAID AIDSno	351820	7.921
NIAID AIDSno	351821	6.750
NIAID AIDSno	351823	7.699
NIAID AIDSno	351824	7.699
NIAID AIDSno	351825	6.629
NIAID AIDSno	351826	6.921
NIAID AIDSno	351827	5.619
NIAID AIDSno	351828	7.167
NIAID AIDSno	351829	6.818
NIAID AIDSno	351830	6.785
NIAID AIDSno	351831	6.879
NIAID AIDSno	351832	6.762
NIAID AIDSno	351833	8.097
NIAID AIDSno	351834	6.863
NIAID AIDSno	351835	6.943

NIAID AIDSno	352956	4.523
NIAID AIDSno	352957	4.495
NIAID AIDSno	352958	4.658
NIAID AIDSno	352961	4.444
NIAID AIDSno	352962	5.000
NIAID AIDSno	352963	4.638
NIAID AIDSno	352964	5.456
NIAID AIDSno	353015	5.699
NIAID AIDSno	353156	3.815
NIAID AIDSno	354387	6.347
NIAID AIDSno	354388	4.567
NIAID AIDSno	354389	6.000
NIAID AIDSno	354390	6.398
NIAID AIDSno	354391	5.620
NIAID AIDSno	354392	5.979
NIAID AIDSno	354393	4.783
NIAID AIDSno	354394	5.155
NIAID AIDSno	354428	5.721
NIAID AIDSno	354429	5.638
NIAID AIDSno	355041	4.996
NIAID AIDSno	356419	5.686
NIAID AIDSno	356420	5.466
NIAID AIDSno	356421	5.095
NIAID AIDSno	356422	6.569
NIAID AIDSno	356423	6.979
NIAID AIDSno	356424	7.076
NIAID AIDSno	356425	7.125
NIAID AIDSno	356426	6.457
NIAID AIDSno	356427	6.022
NIAID AIDSno	356428	6.504
NIAID AIDSno	356429	6.187
NIAID AIDSno	356430	6.951
NIAID AIDSno	356431	6.243
NIAID AIDSno	356432	7.119
NIAID AIDSno	356433	6.535
NIAID AIDSno	356434	6.341
NIAID AIDSno	356435	6.599
NIAID AIDSno	356436	6.428
NIAID AIDSno	356437	6.752
NIAID AIDSno	356438	6.400
NIAID AIDSno	356439	6.754
NIAID AIDSno	356440	6.963
NIAID AIDSno	356441	6.056
NIAID AIDSno	356442	6.569
NIAID AIDSno	356443	6.526

NIAID AIDSno	356444	5.284
NIAID AIDSno	356445	5.966
NIAID AIDSno	356446	6.217
NIAID AIDSno	356447	6.334
NIAID AIDSno	356448	6.319
NIAID AIDSno	356449	7.046
NIAID AIDSno	362591	6.510
NIAID AIDSno	362592	6.141
NIAID AIDSno	362593	5.785
NIAID AIDSno	362594	6.807
NIAID AIDSno	362595	5.467
NIAID AIDSno	362597	6.406
NIAID AIDSno	362599	6.633
NIAID AIDSno	362600	5.854
NIAID AIDSno	362601	5.441
NIAID AIDSno	362602	6.139
NIAID AIDSno	362603	6.364
NIAID AIDSno	375306	5.699
NIAID AIDSno	387786	4.136
NIAID AIDSno	387790	4.848
NIAID AIDSno	391158	4.290
NIAID AIDSno	403053	4.307
NIAID AIDSno	410847	5.921
NIAID AIDSno	410848	5.796
NIAID AIDSno	410849	4.886
NIAID AIDSno	411741	4.919
NIAID AIDSno	411744	5.092
NIAID AIDSno	411745	5.328
NIAID AIDSno	411746	4.886
NIAID AIDSno	411747	5.398
NIAID AIDSno	411749	5.143
NIAID AIDSno	411750	5.268
NIAID AIDSno	411751	5.420
NIAID AIDSno	411752	5.337
NIAID AIDSno	411753	5.167
NIAID AIDSno	411755	4.553
NIAID AIDSno	411756	4.796
NIAID AIDSno	411757	5.319
NIAID AIDSno	411758	5.174
NIAID AIDSno	411759	5.357
NIAID AIDSno	411790	5.408
NIAID AIDSno	411792	6.000
NIAID AIDSno	411793	6.161
NIAID AIDSno	411794	6.585
NIAID AIDSno	411798	5.672

NIAID AIDSno	411916	4.301
NIAID AIDSno	411920	4.097
NIAID AIDSno	412001	5.205
NIAID AIDSno	412003	5.137
NIAID AIDSno	412020	3.998
NIAID AIDSno	412022	5.821
NIAID AIDSno	412023	5.031
NIAID AIDSno	412025	3.994
NIAID AIDSno	413150	5.041
NIAID AIDSno	413216	5.187
NIAID AIDSno	413217	5.678
NIAID AIDSno	413218	5.745
NIAID AIDSno	413219	5.167
NIAID AIDSno	413220	4.796
NIAID AIDSno	415890	6.770
NIAID AIDSno	415900	6.319
NIAID AIDSno	415901	6.347
NIAID AIDSno	415902	6.201
NIAID AIDSno	415903	5.396
NIAID AIDSno	415904	6.292
NIAID AIDSno	415910	4.688
NIAID AIDSno	415911	5.798
NIAID AIDSno	415912	6.553
NIAID AIDSno	415913	6.824
NIAID AIDSno	415915	6.000
NIAID AIDSno	415917	6.602
NIAID AIDSno	415924	6.523
NIAID AIDSno	415928	6.000
NIAID AIDSno	415932	5.983
NIAID AIDSno	415933	6.215
NIAID AIDSno	415934	6.367
NIAID AIDSno	415935	7.097
NIAID AIDSno	415936	4.418
NIAID AIDSno	415937	6.260
NIAID AIDSno	415938	7.398
NIAID AIDSno	415939	6.469
NIAID AIDSno	415943	4.071
NIAID AIDSno	415944	4.300
NIAID AIDSno	415945	5.060
NIAID AIDSno	416578	4.833
NIAID AIDSno	416584	4.939
NIAID AIDSno	416585	4.299
NIAID AIDSno	416586	6.194
NIAID AIDSno	416634	6.699
NIAID AIDSno	416635	7.745

NIAID AIDSno	416636	8.102
NIAID AIDSno	416637	8.620
NIAID AIDSno	416639	8.699
NIAID AIDSno	416640	8.721
NIAID AIDSno	416641	8.022
NIAID AIDSno	416655	8.620
NIAID AIDSno	416658	8.721
NIAID AIDSno	416666	8.114
NIAID AIDSno	416670	8.000
NIAID AIDSno	416757	8.260
NIAID AIDSno	416775	8.432
NIAID AIDSno	416844	8.538
NIAID AIDSno	416887	8.699
NIAID AIDSno	424258	8.796
NIAID AIDSno	434138	6.469
NIAID AIDSno	461350	7.301
NIAID AIDSno	461389	7.301
NIAID AIDSno	461393	7.301
NIAID AIDSno	461397	7.301
NIAID AIDSno	461400	7.301
NIAID AIDSno	461404	7.301
NIAID AIDSno	461411	7.301
NIAID AIDSno	470833	6.824
NIAID AIDSno	470834	8.301
NIAID AIDSno	471188	7.861
NIAID AIDSno	471309	8.097
NIAID AIDSno	471478	3.321
NIAID AIDSno	471494	4.227
NIAID AIDSno	471496	4.382
NIAID AIDSno	471497	4.710
NIAID AIDSno	471502	7.658
NIAID AIDSno	471503	4.672
NIAID AIDSno	471770	6.036
NIAID AIDSno	471771	6.420
NIAID AIDSno	471778	7.000
NIAID AIDSno	471779	7.000
NIAID AIDSno	471780	7.301
NIAID AIDSno	471781	7.398
NIAID AIDSno	471782	7.155
NIAID AIDSno	471783	8.523
NIAID AIDSno	471784	7.222
NIAID AIDSno	471785	7.215
NIAID AIDSno	472889	4.970
NIAID AIDSno	472891	4.509
NIAID AIDSno	472892	4.276

NIAID AIDSno	472893	5.456
NIAID AIDSno	472894	4.071
NIAID AIDSno	472895	4.529
NIAID AIDSno	472900	4.058
NIAID AIDSno	472901	4.451
NIAID AIDSno	472903	3.548
NIAID AIDSno	472904	3.902
NIAID AIDSno	472905	4.264
NIAID AIDSno	472906	3.927
NIAID AIDSno	472907	4.356
NIAID AIDSno	472908	3.916
NIAID AIDSno	472909	4.093
NIAID AIDSno	472910	4.098
NIAID AIDSno	472911	5.004
NIAID AIDSno	472914	3.523
NIAID AIDSno	473149	6.370
NIAID AIDSno	473150	6.121
NIAID AIDSno	473162	5.752
NIAID AIDSno	473163	5.796
NIAID AIDSno	473164	5.824
NIAID AIDSno	473165	1.046
NIAID AIDSno	473166	2.382
NIAID AIDSno	473167	0.268
NIAID AIDSno	473168	5.585
NIAID AIDSno	473169	0.699
NIAID AIDSno	473170	5.745
NIAID AIDSno	473171	5.921
NIAID AIDSno	473172	1.097
NIAID AIDSno	473173	5.321
NIAID AIDSno	473174	5.331
NIAID AIDSno	473175	5.323
NIAID AIDSno	473491	4.735
NIAID AIDSno	473492	4.695
NIAID AIDSno	473533	7.276
NIAID AIDSno	473534	8.131
NIAID AIDSno	473557	9.000
NIAID AIDSno	473562	7.167
NIAID AIDSno	473565	8.854
NIAID AIDSno	473567	7.824
NIAID AIDSno	473568	9.398
NIAID AIDSno	473569	9.699
NIAID AIDSno	475030	5.268
NIAID AIDSno	475033	6.409
NIAID AIDSno	475034	5.268
NIAID AIDSno	475069	3.313

NIAID AIDSno	475070	2.597
NIAID AIDSno	475071	4.128
NIAID AIDSno	475072	4.359
NIAID AIDSno	475073	4.526
NIAID AIDSno	475074	4.196
NIAID AIDSno	475075	4.453
NIAID AIDSno	475076	3.928
NIAID AIDSno	475959	3.694
NIAID AIDSno	475962	3.443
NIAID AIDSno	475964	3.438
NIAID AIDSno	475966	3.583
NIAID AIDSno	475969	3.268
NIAID AIDSno	475970	3.649
NIAID AIDSno	475971	3.251
NIAID AIDSno	475972	3.257
NIAID AIDSno	475975	3.105
NIAID AIDSno	475976	3.029
NIAID AIDSno	475977	3.149
NIAID AIDSno	475978	3.332
NIAID AIDSno	476003	3.470
NIAID AIDSno	476004	3.363
NIAID AIDSno	476005	3.321
NIAID AIDSno	476006	3.312
NIAID AIDSno	476007	3.077
NIAID AIDSno	476714	6.638
NIAID AIDSno	476715	7.620
NIAID AIDSno	476716	7.244
NIAID AIDSno	476717	8.036
NIAID AIDSno	476783	4.000
NIAID AIDSno	476784	5.000
NIAID AIDSno	476785	4.000
NIAID AIDSno	476786	5.000
NIAID AIDSno	476787	6.831
NIAID AIDSno	476788	6.770
NIAID AIDSno	476789	6.080
NIAID AIDSno	476790	5.236
NIAID AIDSno	476791	4.000
NIAID AIDSno	476792	4.000
NIAID AIDSno	476793	4.000
NIAID AIDSno	476794	4.000
NIAID AIDSno	476795	4.000
NIAID AIDSno	476796	4.000
NIAID AIDSno	476797	4.000
NIAID AIDSno	476798	4.000
NIAID AIDSno	476799	5.000

NIAID AIDSno	476800	6.953
NIAID AIDSno	476801	4.000
NIAID AIDSno	476802	5.623
NIAID AIDSno	476803	6.798
NIAID AIDSno	476804	5.000
NIAID AIDSno	476805	6.598
NIAID AIDSno	476806	6.694
NIAID AIDSno	476807	6.803
NIAID AIDSno	476808	4.000
NIAID AIDSno	476809	4.000
NIAID AIDSno	476810	4.000
NIAID AIDSno	476811	5.000
NIAID AIDSno	476812	4.000
NIAID AIDSno	476813	4.000
NIAID AIDSno	476814	5.000
NIAID AIDSno	476815	7.074
NIAID AIDSno	476816	4.000
NIAID AIDSno	476817	6.404
NIAID AIDSno	476818	6.983
NIAID AIDSno	476819	5.000
NIAID AIDSno	476820	6.094
NIAID AIDSno	476821	7.033
NIAID AIDSno	476822	4.000
NIAID AIDSno	476823	6.790
NIAID AIDSno	476824	4.000
NIAID AIDSno	476825	6.158
NIAID AIDSno	476826	7.146
NIAID AIDSno	476827	7.198
NIAID AIDSno	476828	5.222
NIAID AIDSno	476829	6.470
NIAID AIDSno	476830	7.719
NIAID AIDSno	476831	5.629
NIAID AIDSno	476832	7.870
NIAID AIDSno	476833	5.745
NIAID AIDSno	476834	6.286
NIAID AIDSno	476835	6.405
NIAID AIDSno	476836	5.613
NIAID AIDSno	476837	6.378
NIAID AIDSno	476838	6.550
NIAID AIDSno	476839	5.000
NIAID AIDSno	476840	5.000
NIAID AIDSno	476841	6.129
NIAID AIDSno	476842	7.025
NIAID AIDSno	476843	7.092
NIAID AIDSno	476844	6.934

NIAID AIDSno	476845	7.275
NIAID AIDSno	476846	7.213
NIAID AIDSno	476847	5.506
NIAID AIDSno	476848	7.088
NIAID AIDSno	476849	3.561
NIAID AIDSno	476850	3.390
NIAID AIDSno	476854	5.678
NIAID AIDSno	476855	8.155
NIAID AIDSno	476856	5.419
NIAID AIDSno	476857	6.726
NIAID AIDSno	476858	6.109
NIAID AIDSno	476859	7.252
NIAID AIDSno	476860	5.102
NIAID AIDSno	476861	7.745
NIAID AIDSno	476862	6.991
NIAID AIDSno	476863	4.925
NIAID AIDSno	476864	6.602
NIAID AIDSno	476865	6.548
NIAID AIDSno	476866	6.534
NIAID AIDSno	476867	6.793
NIAID AIDSno	476868	7.796
NIAID AIDSno	476869	8.046
NIAID AIDSno	476870	7.319
NIAID AIDSno	476871	6.381
NIAID AIDSno	476874	5.345
NIAID AIDSno	476875	7.376
NIAID AIDSno	476877	7.295
NIAID AIDSno	476878	7.498
NIAID AIDSno	476879	7.607
NIAID AIDSno	476880	7.611
NIAID AIDSno	476881	7.866
NIAID AIDSno	476882	7.395
NIAID AIDSno	476883	7.180
NIAID AIDSno	476885	6.673
NIAID AIDSno	476886	5.023
NIAID AIDSno	476887	6.894
NIAID AIDSno	476888	7.940
NIAID AIDSno	476889	7.815
NIAID AIDSno	476890	7.886
NIAID AIDSno	476892	7.745
NIAID AIDSno	476893	7.184
NIAID AIDSno	476894	7.329
NIAID AIDSno	476895	7.752
NIAID AIDSno	476896	7.845
NIAID AIDSno	476897	5.580

NIAID AIDSno	476898	7.932
NIAID AIDSno	476899	8.180
NIAID AIDSno	476900	5.151
NIAID AIDSno	476901	6.846
NIAID AIDSno	476902	7.570
NIAID AIDSno	476903	6.378
NIAID AIDSno	476904	6.481
NIAID AIDSno	476905	6.686
NIAID AIDSno	476906	6.461
NIAID AIDSno	476907	6.865
NIAID AIDSno	476908	6.364
NIAID AIDSno	476909	7.488
NIAID AIDSno	477052	7.686
NIAID AIDSno	477053	7.812
NIAID AIDSno	477054	7.738
NIAID AIDSno	477055	7.780
NIAID AIDSno	477056	7.258
NIAID AIDSno	477057	7.680
NIAID AIDSno	477058	8.337
NIAID AIDSno	477059	7.432
NIAID AIDSno	477060	8.387
NIAID AIDSno	477061	8.161
NIAID AIDSno	477062	7.788
NIAID AIDSno	477856	4.699
NIAID AIDSno	478126	8.108
NIAID AIDSno	478127	7.796
NIAID AIDSno	478128	8.301
NIAID AIDSno	478129	6.222
NIAID AIDSno	478130	6.071
NIAID AIDSno	478131	6.174
NIAID AIDSno	478132	6.260
NIAID AIDSno	479273	8.420
NIAID AIDSno	479274	7.569
NIAID AIDSno	479279	7.387
NIAID AIDSno	479281	7.000
NIAID AIDSno	479284	7.721
NIAID AIDSno	479285	7.620
NIAID AIDSno	479286	8.444
NIAID AIDSno	479287	8.921
NIAID AIDSno	479288	9.097
NIAID AIDSno	479322	5.721
NIAID AIDSno	479323	5.114
NIAID AIDSno	479327	4.796
NIAID AIDSno	479331	6.699
NIAID AIDSno	479332	5.569

NIAID AIDSno	479485	7.620
NIAID AIDSno	479486	7.620
NIAID AIDSno	479487	8.097
NIAID AIDSno	479488	8.108
NIAID AIDSno	479489	8.301
NIAID AIDSno	479490	8.523
NIAID AIDSno	479491	8.523
NIAID AIDSno	479893	7.745
NIAID AIDSno	479894	8.523
NIAID AIDSno	479895	7.180
NIAID AIDSno	479897	5.678
NIAID AIDSno	479899	6.322
NIAID AIDSno	479900	6.987
NIAID AIDSno	479902	6.883
NIAID AIDSno	479903	7.745
NIAID AIDSno	479909	7.229
NIAID AIDSno	479910	6.876
NIAID AIDSno	479911	5.680
NIAID AIDSno	479912	7.149
NIAID AIDSno	479913	7.108
NIAID AIDSno	480584	7.346
NIAID AIDSno	480586	7.261
NIAID AIDSno	480587	8.046
NIAID AIDSno	480588	8.155
NIAID AIDSno	480589	8.460
NIAID AIDSno	480591	7.886
NIAID AIDSno	480592	8.301
NIAID AIDSno	480593	8.222
NIAID AIDSno	480594	8.301
NIAID AIDSno	480595	7.721
NIAID AIDSno	480596	8.398
NIAID AIDSno	480597	8.523
NIAID AIDSno	480598	8.398
NIAID AIDSno	480599	8.222
NIAID AIDSno	480727	6.041
NIAID AIDSno	480728	5.470
NIAID AIDSno	480734	5.231
NIAID AIDSno	480735	6.032
NIAID AIDSno	480736	5.444
NIAID AIDSno	480737	4.407
NIAID AIDSno	480780	7.495
NIAID AIDSno	480781	7.125
NIAID AIDSno	480824	7.824
NIAID AIDSno	480895	6.565
NIAID AIDSno	480898	7.745

NIAID AIDSno	480900	6.752
NIAID AIDSno	480914	6.438
NIAID AIDSno	480916	5.680
NIAID AIDSno	480919	5.619
NIAID AIDSno	481415	7.398
NIAID AIDSno	481445	4.756
NIAID AIDSno	481447	5.292
NIAID AIDSno	481449	5.025
NIAID AIDSno	481450	4.323
NIAID AIDSno	481452	4.591
NIAID AIDSno	481454	4.788
NIAID AIDSno	481456	4.836
NIAID AIDSno	481457	4.816
NIAID AIDSno	481459	9.523
NIAID AIDSno	481460	5.319
NIAID AIDSno	481765	7.469
NIAID AIDSno	481766	7.602
NIAID AIDSno	481767	7.796
NIAID AIDSno	481769	7.658
NIAID AIDSno	481770	7.208
NIAID AIDSno	481771	7.569
NIAID AIDSno	481772	7.066
NIAID AIDSno	481773	7.569
NIAID AIDSno	481775	7.469
NIAID AIDSno	481776	6.724
NIAID AIDSno	481777	7.092
NIAID AIDSno	481779	7.481
NIAID AIDSno	481782	9.398
NIAID AIDSno	481784	6.445
NIAID AIDSno	481786	8.699
NIAID AIDSno	481788	7.215
NIAID AIDSno	481790	9.398
NIAID AIDSno	481791	8.523
NIAID AIDSno	481792	7.553
NIAID AIDSno	481793	7.444
NIAID AIDSno	481794	8.000
NIAID AIDSno	481795	8.523
NIAID AIDSno	481870	4.000
NIAID AIDSno	482059	5.244
NIAID AIDSno	482064	4.509
NIAID AIDSno	482071	5.387
NIAID AIDSno	482074	6.495
NIAID AIDSno	482268	4.573
NIAID AIDSno	482275	4.577
NIAID AIDSno	482292	6.479

NIAID AIDSno	482293	6.951
NIAID AIDSno	482295	5.777
NIAID AIDSno	482296	6.399
NIAID AIDSno	482297	7.301
NIAID AIDSno	482298	7.602
NIAID AIDSno	482299	7.149
NIAID AIDSno	482324	6.602
NIAID AIDSno	482326	6.959
NIAID AIDSno	482504	6.502
NIAID AIDSno	482551	7.027
NIAID AIDSno	482958	7.721
NIAID AIDSno	482959	9.301
NIAID AIDSno	482961	7.745
NIAID AIDSno	482963	7.854
NIAID AIDSno	482964	8.444
NIAID AIDSno	482966	8.244
NIAID AIDSno	482967	9.699
NIAID AIDSno	482969	8.347
NIAID AIDSno	482970	8.409
NIAID AIDSno	482972	8.041
NIAID AIDSno	482974	6.027
NIAID AIDSno	482975	6.367
NIAID AIDSno	482976	6.260
NIAID AIDSno	482977	5.658
NIAID AIDSno	482979	7.229
NIAID AIDSno	482981	7.824
NIAID AIDSno	482983	8.420
NIAID AIDSno	482985	8.620
NIAID AIDSno	482986	7.854
NIAID AIDSno	482987	8.602
NIAID AIDSno	482988	8.553
NIAID AIDSno	482989	8.208
NIAID AIDSno	482991	8.509
NIAID AIDSno	482992	8.509
NIAID AIDSno	482993	8.056
NIAID AIDSno	482994	7.796
NIAID AIDSno	482995	7.959
NIAID AIDSno	482996	8.000
NIAID AIDSno	482997	8.161
NIAID AIDSno	482998	7.347
NIAID AIDSno	482999	7.824
NIAID AIDSno	483000	7.398
NIAID AIDSno	483001	5.217
NIAID AIDSno	483002	5.149
NIAID AIDSno	483003	5.442

NIAID AIDSno	483004	5.467
NIAID AIDSno	483005	5.077
NIAID AIDSno	483009	7.071
NIAID AIDSno	483011	7.000
NIAID AIDSno	483013	6.114
NIAID AIDSno	483015	6.389
NIAID AIDSno	483016	7.699
NIAID AIDSno	483017	7.155
NIAID AIDSno	483018	6.227
NIAID AIDSno	483020	5.011
NIAID AIDSno	483021	6.097
NIAID AIDSno	483022	6.301
NIAID AIDSno	483030	4.319
NIAID AIDSno	483032	6.301
NIAID AIDSno	483034	7.523
NIAID AIDSno	483035	6.770
NIAID AIDSno	483036	7.155
NIAID AIDSno	483038	8.523
NIAID AIDSno	483039	8.000
NIAID AIDSno	483040	7.523
NIAID AIDSno	483041	7.328
NIAID AIDSno	483044	7.538
NIAID AIDSno	483045	7.658
NIAID AIDSno	483054	6.699
NIAID AIDSno	483056	6.022
NIAID AIDSno	483057	5.125
NIAID AIDSno	483065	5.703
NIAID AIDSno	483066	7.699
NIAID AIDSno	483083	8.000
NIAID AIDSno	483085	8.301
NIAID AIDSno	483086	6.381
NIAID AIDSno	483088	7.201
NIAID AIDSno	483092	7.658
NIAID AIDSno	483094	6.706
NIAID AIDSno	483095	8.086
NIAID AIDSno	483097	6.130
NIAID AIDSno	483168	6.585
NIAID AIDSno	483169	6.114
NIAID AIDSno	483170	5.368
NIAID AIDSno	483172	4.128
NIAID AIDSno	483173	5.914
NIAID AIDSno	483174	5.991
NIAID AIDSno	483175	5.008
NIAID AIDSno	483176	6.638
NIAID AIDSno	483177	6.071

NIAID AIDSno	483178	4.797
NIAID AIDSno	483179	4.792
NIAID AIDSno	483180	5.535
NIAID AIDSno	483181	5.697
NIAID AIDSno	483182	5.818
NIAID AIDSno	483183	6.900
NIAID AIDSno	483184	6.863
NIAID AIDSno	483191	6.860
NIAID AIDSno	483192	7.102
NIAID AIDSno	483194	6.914
NIAID AIDSno	483195	7.495
NIAID AIDSno	483197	7.056
NIAID AIDSno	483199	6.437
NIAID AIDSno	483203	5.582
NIAID AIDSno	483204	5.309
NIAID AIDSno	483205	3.924
NIAID AIDSno	483206	4.897
NIAID AIDSno	483207	6.745
NIAID AIDSno	483216	5.660
NIAID AIDSno	483218	6.633
NIAID AIDSno	483221	6.631
NIAID AIDSno	483225	7.187
NIAID AIDSno	483228	5.666
NIAID AIDSno	483229	5.627
NIAID AIDSno	483231	4.986
NIAID AIDSno	483235	6.652
NIAID AIDSno	483236	6.893
NIAID AIDSno	483237	6.609
NIAID AIDSno	483238	6.678
NIAID AIDSno	483241	6.747
NIAID AIDSno	483242	6.595
NIAID AIDSno	483246	8.244
NIAID AIDSno	483262	6.959
NIAID AIDSno	483326	6.921
NIAID AIDSno	483895	6.658
NIAID AIDSno	483962	4.187
NIAID AIDSno	484080	8.229
NIAID AIDSno	484193	5.456
NIAID AIDSno	484194	7.222
NIAID AIDSno	484199	7.301
NIAID AIDSno	484291	4.368
NIAID AIDSno	484295	3.646
NIAID AIDSno	484307	3.305
NIAID AIDSno	484309	4.499
NIAID AIDSno	484359	8.111

NIAID AIDSno	485203	5.086
NIAID AIDSno	485211	6.398
NIAID AIDSno	485213	6.301
NIAID AIDSno	485253	6.921
NIAID AIDSno	485260	5.959
NIAID AIDSno	485324	8.721
NIAID AIDSno	485816	7.959
NIAID AIDSno	485817	8.699
NIAID AIDSno	485818	8.699
NIAID AIDSno	485819	8.699
NIAID AIDSno	485820	8.699
NIAID AIDSno	485827	4.469
NIAID AIDSno	485829	4.745
NIAID AIDSno	485831	9.000
NIAID AIDSno	485834	6.886
NIAID AIDSno	485835	6.638
NIAID AIDSno	485837	6.824
NIAID AIDSno	485838	6.161
NIAID AIDSno	485840	6.276
NIAID AIDSno	485841	5.237
NIAID AIDSno	486013	6.398
NIAID AIDSno	486015	5.086
NIAID AIDSno	486016	5.409
NIAID AIDSno	486017	6.000
NIAID AIDSno	486019	4.678
NIAID AIDSno	486021	4.854
NIAID AIDSno	486024	6.194
NIAID AIDSno	486025	7.000
NIAID AIDSno	486028	6.284
NIAID AIDSno	486029	6.444
NIAID AIDSno	486030	7.000
NIAID AIDSno	486032	7.699
NIAID AIDSno	486036	8.479
NIAID AIDSno	486037	8.261
NIAID AIDSno	486039	8.577
NIAID AIDSno	486086	6.796
NIAID AIDSno	486114	7.000
NIAID AIDSno	486117	4.114
NIAID AIDSno	486121	8.523
NIAID AIDSno	486124	6.854
NIAID AIDSno	486125	6.553
NIAID AIDSno	486128	7.770
NIAID AIDSno	486131	4.523
NIAID AIDSno	486134	7.000
NIAID AIDSno	486137	5.886

NIAID AIDSno	486138	7.000
NIAID AIDSno	486141	6.699
NIAID AIDSno	486143	6.801
NIAID AIDSno	486254	7.337
NIAID AIDSno	486259	4.415
NIAID AIDSno	486609	5.733
NIAID AIDSno	486610	6.456
NIAID AIDSno	486611	6.180
NIAID AIDSno	486612	5.959
NIAID AIDSno	486613	5.975
NIAID AIDSno	486614	5.836
NIAID AIDSno	486736	4.750
NIAID AIDSno	486737	4.423
NIAID AIDSno	486738	6.347
NIAID AIDSno	486739	6.367
NIAID AIDSno	486741	3.260
NIAID AIDSno	486744	5.730
NIAID AIDSno	486746	5.015
NIAID AIDSno	486747	5.223
NIAID AIDSno	486749	4.757
NIAID AIDSno	486752	3.348
NIAID AIDSno	486753	3.287
NIAID AIDSno	486754	3.302
NIAID AIDSno	486755	3.317
NIAID AIDSno	486756	3.293
NIAID AIDSno	486757	3.261
NIAID AIDSno	486758	3.277
NIAID AIDSno	487070	6.000
NIAID AIDSno	487101	7.921
NIAID AIDSno	487102	6.678
NIAID AIDSno	487103	7.268
NIAID AIDSno	487104	6.638
NIAID AIDSno	487105	6.721
NIAID AIDSno	487107	7.149
NIAID AIDSno	487108	7.377
NIAID AIDSno	487109	7.738
NIAID AIDSno	487110	7.638
NIAID AIDSno	487112	7.509
NIAID AIDSno	487113	8.237
NIAID AIDSno	487114	7.699
NIAID AIDSno	487115	7.854
NIAID AIDSno	487116	8.699
NIAID AIDSno	487117	6.032
NIAID AIDSno	487119	7.854
NIAID AIDSno	487120	7.921

NIAID AIDSno	487121	6.745
NIAID AIDSno	487122	7.745
NIAID AIDSno	487124	8.097
NIAID AIDSno	487126	7.481
NIAID AIDSno	487130	9.000
NIAID AIDSno	487131	7.620
NIAID AIDSno	487132	9.155
NIAID AIDSno	487135	7.620
NIAID AIDSno	487136	8.155
NIAID AIDSno	487140	6.481
NIAID AIDSno	487141	6.387
NIAID AIDSno	487143	6.585
NIAID AIDSno	487144	7.367
NIAID AIDSno	487145	7.215
NIAID AIDSno	487146	7.509
NIAID AIDSno	487147	8.097
NIAID AIDSno	487148	7.086
NIAID AIDSno	487149	7.585
NIAID AIDSno	495131	6.738
NIAID AIDSno	495133	7.730
NIAID AIDSno	495134	7.640
NIAID AIDSno	495135	4.518
NIAID AIDSno	495137	6.907
NIAID AIDSno	495138	7.082
NIAID AIDSno	495140	7.067
NIAID AIDSno	495141	4.563
NIAID AIDSno	495144	5.218
NIAID AIDSno	495205	8.523
NIAID AIDSno	495206	8.222
NIAID AIDSno	495207	8.097
NIAID AIDSno	495208	8.699
NIAID AIDSno	495209	9.000
NIAID AIDSno	495210	8.398
NIAID AIDSno	495211	8.155
NIAID AIDSno	495212	8.699
NIAID AIDSno	495213	8.398
NIAID AIDSno	495214	8.699
NIAID AIDSno	495215	7.721
NIAID AIDSno	495216	6.712
NIAID AIDSno	495217	6.474
NIAID AIDSno	495218	7.211
NIAID AIDSno	495250	4.485
NIAID AIDSno	495252	5.777
NIAID AIDSno	495254	8.523
NIAID AIDSno	495255	4.799

NIAID AIDSno	495257	5.474
NIAID AIDSno	495259	6.071
NIAID AIDSno	495263	6.879
NIAID AIDSno	495278	6.444
NIAID AIDSno	495279	3.547
NIAID AIDSno	495373	4.233
NIAID AIDSno	495376	3.769
NIAID AIDSno	495377	4.472
NIAID AIDSno	495885	5.523
NIAID AIDSno	495886	7.511
NIAID AIDSno	495887	6.959
NIAID AIDSno	495888	9.921
NIAID AIDSno	495889	7.721
NIAID AIDSno	495890	5.721
NIAID AIDSno	495891	6.114
NIAID AIDSno	495892	5.678
NIAID AIDSno	495893	6.000
NIAID AIDSno	495894	5.187
NIAID AIDSno	495895	6.180
NIAID AIDSno	495896	6.036
NIAID AIDSno	495897	6.201
NIAID AIDSno	495898	6.268
NIAID AIDSno	495913	4.444
NIAID AIDSno	495914	5.201
NIAID AIDSno	495915	4.268
NIAID AIDSno	495916	4.569
NIAID AIDSno	495935	4.114
NIAID AIDSno	495938	5.009
NIAID AIDSno	495942	4.260
NIAID AIDSno	495943	6.444
NIAID AIDSno	495945	4.824
NIAID AIDSno	495946	5.959
NIAID AIDSno	495949	5.699
NIAID AIDSno	495953	7.187
NIAID AIDSno	495954	6.921
NIAID AIDSno	495955	5.420
NIAID AIDSno	495956	6.252
NIAID AIDSno	495958	5.367
NIAID AIDSno	495960	5.921
NIAID AIDSno	495963	5.215
NIAID AIDSno	495964	4.509
NIAID AIDSno	495966	5.481
NIAID AIDSno	495967	5.921
NIAID AIDSno	495969	5.585
NIAID AIDSno	495971	6.092

NIAID AIDSno	495972	6.018
NIAID AIDSno	495974	6.222
NIAID AIDSno	495975	6.292
NIAID AIDSno	495976	6.456
NIAID AIDSno	495980	4.620
NIAID AIDSno	495981	4.456
NIAID AIDSno	495983	4.509
NIAID AIDSno	495985	5.495
NIAID AIDSno	495989	5.377
NIAID AIDSno	495993	3.495
NIAID AIDSno	495995	3.530
NIAID AIDSno	495996	4.013
NIAID AIDSno	495998	3.283
NIAID AIDSno	496002	3.428
NIAID AIDSno	497260	6.328
NIAID AIDSno	497272	7.092
NIAID AIDSno	497274	6.928
NIAID AIDSno	497276	7.469
NIAID AIDSno	497278	7.377
NIAID AIDSno	497280	6.051
NIAID AIDSno	497282	6.000
NIAID AIDSno	497283	6.620
NIAID AIDSno	497285	6.886
NIAID AIDSno	497286	7.131
NIAID AIDSno	497288	6.222
NIAID AIDSno	497289	6.585
NIAID AIDSno	497291	6.509
NIAID AIDSno	497292	7.367
NIAID AIDSno	497293	7.398
NIAID AIDSno	499082	5.201
NIAID AIDSno	499586	4.854
NIAID AIDSno	502108	7.699
NIAID AIDSno	502109	6.988
NIAID AIDSno	502110	6.570
NIAID AIDSno	502111	7.678
NIAID AIDSno	502112	6.827
NIAID AIDSno	502113	6.201
NIAID AIDSno	502114	7.310
NIAID AIDSno	502115	8.398
NIAID AIDSno	502116	8.921
NIAID AIDSno	502117	7.398
NIAID AIDSno	502118	7.328
NIAID AIDSno	502119	6.509
NIAID AIDSno	502120	5.764
NIAID AIDSno	502121	6.516

NIAID AIDSno	502122	7.293
NIAID AIDSno	502686	4.233
NIAID AIDSno	502688	4.429
NIAID AIDSno	502689	4.496
NIAID AIDSno	502690	4.370
NIAID AIDSno	502868	1.699
NIAID AIDSno	503067	3.233
NIAID AIDSno	503544	5.051
NIAID AIDSno	503686	2.699
NIAID AIDSno	503703	2.959
NIAID AIDSno	503732	5.489
NIAID AIDSno	503986	4.758
NIAID AIDSno	503987	5.088
NIAID AIDSno	503988	4.321
NIAID AIDSno	503989	4.040
NIAID AIDSno	503990	4.042
NIAID AIDSno	503998	4.042
NIAID AIDSno	504006	4.524
NIAID AIDSno	504007	4.744
NIAID AIDSno	504010	4.766
NIAID AIDSno	504011	4.194
NIAID AIDSno	504148	4.225
NIAID AIDSno	504149	5.533
NIAID AIDSno	504152	4.312
NIAID AIDSno	504156	4.287
NIAID AIDSno	504157	4.721
NIAID AIDSno	504159	4.995
NIAID AIDSno	504160	5.620
NIAID AIDSno	504161	6.745
NIAID AIDSno	504162	5.381
NIAID AIDSno	504163	5.519
NIAID AIDSno	504164	5.081
NIAID AIDSno	504165	5.250
NIAID AIDSno	504166	5.690
NIAID AIDSno	504167	4.855
NIAID AIDSno	504168	4.603
NIAID AIDSno	504169	4.574
NIAID AIDSno	504170	4.912
NIAID AIDSno	504280	8.092
NIAID AIDSno	504800	4.756
NIAID AIDSno	504871	4.050
NIAID AIDSno	504873	4.219
NIAID AIDSno	504939	6.796
NIAID AIDSno	504940	7.000
NIAID AIDSno	504941	6.149

NIAID AIDSno	504942	7.553
NIAID AIDSno	505134	7.060
NIAID AIDSno	505135	7.721
NIAID AIDSno	505136	6.618
NIAID AIDSno	505137	6.818
NIAID AIDSno	505138	7.538
NIAID AIDSno	505139	7.638
NIAID AIDSno	505140	7.678
NIAID AIDSno	505141	7.468
NIAID AIDSno	505142	7.553
NIAID AIDSno	505143	7.658
NIAID AIDSno	505144	7.347
NIAID AIDSno	505145	7.076
NIAID AIDSno	505146	7.046
NIAID AIDSno	505147	6.836
NIAID AIDSno	505148	6.883
NIAID AIDSno	505149	7.495
NIAID AIDSno	505150	6.046
NIAID AIDSno	505151	6.717
NIAID AIDSno	505152	7.174
NIAID AIDSno	505177	8.638
NIAID AIDSno	505180	8.268
NIAID AIDSno	505301	6.240
NIAID AIDSno	505303	6.071
NIAID AIDSno	505306	5.854
NIAID AIDSno	505308	5.638
NIAID AIDSno	505500	5.985
NIAID AIDSno	505501	6.328
NIAID AIDSno	506065	7.409
NIAID AIDSno	506075	9.046
NIAID AIDSno	506083	6.337
NIAID AIDSno	506084	8.585
NIAID AIDSno	506085	7.509
NIAID AIDSno	506086	8.796
NIAID AIDSno	506087	9.523
NIAID AIDSno	506096	9.155
NIAID AIDSno	506098	8.495
NIAID AIDSno	506099	8.959
NIAID AIDSno	506100	9.155
NIAID AIDSno	506101	8.276
NIAID AIDSno	506102	9.155
NIAID AIDSno	506103	9.398
NIAID AIDSno	506104	9.301
NIAID AIDSno	506105	9.523
NIAID AIDSno	506107	7.538

NIAID AIDSno	506108	8.678
NIAID AIDSno	506109	8.284
NIAID AIDSno	506110	7.509
NIAID AIDSno	506111	8.310
NIAID AIDSno	506112	8.678
NIAID AIDSno	506196	4.896
NIAID AIDSno	507035	7.620
NIAID AIDSno	507036	7.721
NIAID AIDSno	507037	7.828
NIAID AIDSno	507038	7.495
NIAID AIDSno	507040	7.721
NIAID AIDSno	507041	8.155
NIAID AIDSno	507042	7.620
NIAID AIDSno	507043	7.377
NIAID AIDSno	507044	7.959
NIAID AIDSno	507045	6.987
NIAID AIDSno	507046	7.292
NIAID AIDSno	507047	7.796
NIAID AIDSno	507048	8.046
NIAID AIDSno	507049	7.553
NIAID AIDSno	507051	7.119
NIAID AIDSno	507057	7.620
NIAID AIDSno	507058	7.854
NIAID AIDSno	507059	7.921
NIAID AIDSno	507060	7.066
NIAID AIDSno	507061	7.721
NIAID AIDSno	507062	6.963
NIAID AIDSno	507063	8.097
NIAID AIDSno	507064	7.554
NIAID AIDSno	507065	7.699
NIAID AIDSno	507066	8.222
NIAID AIDSno	507067	7.745
NIAID AIDSno	507068	7.252
NIAID AIDSno	507069	7.721
NIAID AIDSno	507070	7.678
NIAID AIDSno	507071	8.222
NIAID AIDSno	507072	7.638
NIAID AIDSno	507073	7.636
NIAID AIDSno	507074	7.444
NIAID AIDSno	507075	7.824
NIAID AIDSno	507906	8.292
NIAID AIDSno	508250	5.456
NIAID AIDSno	508251	4.398
NIAID AIDSno	508252	4.000
NIAID AIDSno	508253	6.301

NIAID AIDSno	508254	4.000
NIAID AIDSno	508255	4.000
NIAID AIDSno	508256	5.357
NIAID AIDSno	508257	7.155
NIAID AIDSno	508258	7.046
NIAID AIDSno	508259	5.292
NIAID AIDSno	508260	5.387
NIAID AIDSno	508261	4.000
NIAID AIDSno	508262	7.081
NIAID AIDSno	508263	8.097
NIAID AIDSno	508264	7.155
NIAID AIDSno	508265	7.097
NIAID AIDSno	508266	7.260
NIAID AIDSno	508267	7.046
NIAID AIDSno	508268	5.013
NIAID AIDSno	508269	4.627
NIAID AIDSno	508270	4.000
NIAID AIDSno	508271	4.000
NIAID AIDSno	508272	5.590
NIAID AIDSno	508313	5.190
NIAID AIDSno	508410	6.308
NIAID AIDSno	508469	5.174
NIAID AIDSno	508490	7.914
NIAID AIDSno	508493	8.237
NIAID AIDSno	508496	7.799
NIAID AIDSno	508517	8.301
NIAID AIDSno	508529	8.155
NIAID AIDSno	508531	7.706
NIAID AIDSno	508532	7.480
NIAID AIDSno	508533	7.166
NIAID AIDSno	508605	7.613
NIAID AIDSno	508606	7.959
NIAID AIDSno	509439	7.928
NIAID AIDSno	509440	8.066
NIAID AIDSno	509441	8.481
NIAID AIDSno	509442	8.432
NIAID AIDSno	509443	9.046
NIAID AIDSno	509444	7.284
NIAID AIDSno	509445	7.260
NIAID AIDSno	509817	6.523
NIAID AIDSno	510055	7.424
NIAID AIDSno	510076	7.626
NIAID AIDSno	510079	8.247
NIAID AIDSno	510540	5.550
NIAID AIDSno	510542	5.302

NIAID AIDSno	510543	5.326
NIAID AIDSno	510544	5.213
NIAID AIDSno	510546	5.426
NIAID AIDSno	510547	5.409
NIAID AIDSno	510548	5.315
NIAID AIDSno	510549	5.452
NIAID AIDSno	511013	7.790
NIAID AIDSno	511016	7.893
NIAID AIDSno	511019	8.187
NIAID AIDSno	511021	8.187
NIAID AIDSno	511024	7.728
NIAID AIDSno	511028	8.076
NIAID AIDSno	511033	7.738
NIAID AIDSno	511035	8.114
NIAID AIDSno	511039	8.509
NIAID AIDSno	511042	8.086
NIAID AIDSno	511044	8.420
NIAID AIDSno	511045	8.194
NIAID AIDSno	511047	8.187
NIAID AIDSno	511051	8.292
NIAID AIDSno	511053	7.900
NIAID AIDSno	511056	8.180
NIAID AIDSno	511058	7.827
NIAID AIDSno	511059	7.499
NIAID AIDSno	511061	7.719
NIAID AIDSno	511064	7.719
NIAID AIDSno	511068	8.319
NIAID AIDSno	511071	8.244
NIAID AIDSno	511074	7.735
NIAID AIDSno	511076	7.177
NIAID AIDSno	511078	8.215
NIAID AIDSno	511081	8.161
NIAID AIDSno	511085	7.564
NIAID AIDSno	511088	7.810
NIAID AIDSno	511091	7.086
NIAID AIDSno	511093	7.670
NIAID AIDSno	511731	9.523
NIAID AIDSno	511732	9.301
NIAID AIDSno	511733	8.721
NIAID AIDSno	511734	8.420
NIAID AIDSno	511735	8.796
NIAID AIDSno	511736	8.921
NIAID AIDSno	511737	8.456
NIAID AIDSno	511738	7.602
NIAID AIDSno	511739	8.886

NIAID AIDSno	511740	9.046
NIAID AIDSno	511741	8.319
NIAID AIDSno	511742	9.301
NIAID AIDSno	511743	8.378
NIAID AIDSno	511744	7.602
NIAID AIDSno	511745	8.659
NIAID AIDSno	511746	6.523
NIAID AIDSno	511747	8.481
NIAID AIDSno	511748	8.721
NIAID AIDSno	511750	8.469
NIAID AIDSno	511751	8.959
NIAID AIDSno	511753	8.810
NIAID AIDSno	511754	8.886
NIAID AIDSno	511755	9.018
NIAID AIDSno	511756	9.046
NIAID AIDSno	511757	8.495
NIAID AIDSno	511974	4.411
NIAID AIDSno	512160	8.310
NIAID AIDSno	512161	8.131
NIAID AIDSno	512199	5.959
NIAID AIDSno	512206	5.959
NIAID AIDSno	512258	4.631
NIAID AIDSno	512300	4.738
NIAID AIDSno	512301	4.777
NIAID AIDSno	512302	4.939
NIAID AIDSno	512759	5.208
NIAID AIDSno	512761	6.770
NIAID AIDSno	512763	5.027
NIAID AIDSno	512764	5.824
NIAID AIDSno	512765	4.886
NIAID AIDSno	513112	5.475
NIAID AIDSno	513128	5.810
NIAID AIDSno	513431	5.398
NIAID AIDSno	513432	3.824
NIAID AIDSno	513434	4.409
NIAID AIDSno	513436	4.229
NIAID AIDSno	513438	5.301
NIAID AIDSno	513443	3.367
NIAID AIDSno	513451	3.244
NIAID AIDSno	513549	6.556
NIAID AIDSno	513550	6.571
NIAID AIDSno	513611	5.180
NIAID AIDSno	513612	4.840
NIAID AIDSno	513613	4.948
NIAID AIDSno	513614	4.914

NIAID AIDSno	513616	5.000
NIAID AIDSno	513617	4.046
NIAID AIDSno	513618	4.662
NIAID AIDSno	513619	5.335
NIAID AIDSno	513620	5.289
NIAID AIDSno	513621	5.124
NIAID AIDSno	513622	4.799
NIAID AIDSno	513623	5.427
NIAID AIDSno	513624	5.268
NIAID AIDSno	513625	5.144
NIAID AIDSno	513626	5.845
NIAID AIDSno	513627	5.460
NIAID AIDSno	513750	5.521
NIAID AIDSno	513751	7.215
NIAID AIDSno	513759	7.921
NIAID AIDSno	513760	8.097
NIAID AIDSno	513761	7.469
NIAID AIDSno	513763	8.097
NIAID AIDSno	513764	7.523
NIAID AIDSno	513765	7.886
NIAID AIDSno	513767	7.268
NIAID AIDSno	513768	6.775
NIAID AIDSno	513769	6.824
NIAID AIDSno	513770	7.097
NIAID AIDSno	513773	7.678
NIAID AIDSno	513774	5.977
NIAID AIDSno	513775	7.770
NIAID AIDSno	513776	7.398
NIAID AIDSno	513781	7.097
NIAID AIDSno	513783	7.187
NIAID AIDSno	513784	6.553
NIAID AIDSno	513786	6.801
NIAID AIDSno	513787	7.004
NIAID AIDSno	513788	7.201
NIAID AIDSno	513789	7.260
NIAID AIDSno	513790	6.148
NIAID AIDSno	513793	7.114
NIAID AIDSno	513794	6.893
NIAID AIDSno	513795	7.222
NIAID AIDSno	513797	7.854
NIAID AIDSno	513815	5.621
NIAID AIDSno	513817	5.326
NIAID AIDSno	513818	6.022
NIAID AIDSno	514118	8.252
NIAID AIDSno	514119	8.137

NIAID AIDSno	514122	9.398
NIAID AIDSno	514124	8.201
NIAID AIDSno	514125	8.824
NIAID AIDSno	514167	5.503
NIAID AIDSno	514168	4.959
NIAID AIDSno	514169	5.897
NIAID AIDSno	514170	6.668
NIAID AIDSno	514171	7.137
NIAID AIDSno	514172	6.971
NIAID AIDSno	514173	6.200
NIAID AIDSno	514174	6.284
NIAID AIDSno	514175	5.602
NIAID AIDSno	514176	6.509
NIAID AIDSno	514177	6.971
NIAID AIDSno	514178	6.228
NIAID AIDSno	514179	6.493
NIAID AIDSno	514180	7.481
NIAID AIDSno	514181	7.699
NIAID AIDSno	514182	7.456
NIAID AIDSno	514183	5.301
NIAID AIDSno	514184	7.620
NIAID AIDSno	514185	5.938
NIAID AIDSno	514186	6.119
NIAID AIDSno	514187	6.403
NIAID AIDSno	514188	6.838
NIAID AIDSno	514189	7.569
NIAID AIDSno	514190	7.658
NIAID AIDSno	514191	7.398
NIAID AIDSno	514192	7.509
NIAID AIDSno	514193	7.125
NIAID AIDSno	514194	8.097
NIAID AIDSno	514195	8.155
NIAID AIDSno	514196	8.301
NIAID AIDSno	514197	6.000
NIAID AIDSno	514200	6.553
NIAID AIDSno	514201	7.222
NIAID AIDSno	514202	7.854
NIAID AIDSno	514203	7.602
NIAID AIDSno	514204	8.222
NIAID AIDSno	514205	8.387
NIAID AIDSno	514206	7.155
NIAID AIDSno	514537	4.086
NIAID AIDSno	514548	4.215
NIAID AIDSno	514551	4.222
NIAID AIDSno	514553	5.301

NIAID AIDSno	514555	5.222
NIAID AIDSno	514562	4.921
NIAID AIDSno	515466	4.854
NIAID AIDSno	515467	4.638
NIAID AIDSno	515468	5.119
NIAID AIDSno	515469	6.000
NIAID AIDSno	515471	5.000
NIAID AIDSno	515472	4.367
NIAID AIDSno	515473	6.301
NIAID AIDSno	515621	9.097
NIAID AIDSno	515622	6.699
NIAID AIDSno	515623	7.553
NIAID AIDSno	515624	9.398
NIAID AIDSno	515625	7.229
NIAID AIDSno	515626	7.770
NIAID AIDSno	515627	7.921
NIAID AIDSno	515628	8.056
NIAID AIDSno	515629	8.222
NIAID AIDSno	515630	8.046
NIAID AIDSno	515631	8.046
NIAID AIDSno	515632	8.161
NIAID AIDSno	515633	7.959
NIAID AIDSno	515634	7.959
NIAID AIDSno	515635	7.959
NIAID AIDSno	515636	7.854
NIAID AIDSno	515705	7.590
NIAID AIDSno	515706	7.572
NIAID AIDSno	515707	6.979
NIAID AIDSno	515708	7.424
NIAID AIDSno	515709	7.398
NIAID AIDSno	519068	7.222
NIAID AIDSno	519069	6.602
NIAID AIDSno	519070	7.222
NIAID AIDSno	519071	6.215
NIAID AIDSno	519072	5.133
NIAID AIDSno	519594	4.717
NIAID AIDSno	519595	4.735
NIAID AIDSno	519596	4.765
NIAID AIDSno	520463	6.222
NIAID AIDSno	520465	6.187
NIAID AIDSno	520468	4.767
NIAID AIDSno	521013	4.724
NIAID AIDSno	521014	5.124
NIAID AIDSno	521064	8.398
NIAID AIDSno	521112	4.708

NIAID AIDSno	521114	4.910
NIAID AIDSno	522147	5.602
NIAID AIDSno	522148	5.553
NIAID AIDSno	522151	5.796
NIAID AIDSno	522152	5.721
NIAID AIDSno	522176	5.824
NIAID AIDSno	522178	5.699
NIAID AIDSno	522180	5.824
NIAID AIDSno	522181	5.770
NIAID AIDSno	522183	5.886
NIAID AIDSno	522184	5.796
NIAID AIDSno	522185	5.770
NIAID AIDSno	522188	5.678
NIAID AIDSno	522291	6.347
NIAID AIDSno	522646	5.252
NIAID AIDSno	522647	6.678
NIAID AIDSno	522648	4.745
NIAID AIDSno	522649	5.051
NIAID AIDSno	522650	5.585
NIAID AIDSno	522651	4.310
NIAID AIDSno	522652	5.000
NIAID AIDSno	522653	5.523
NIAID AIDSno	522654	6.824
NIAID AIDSno	523552	3.414
NIAID AIDSno	523553	3.527
NIAID AIDSno	523619	5.839
NIAID AIDSno	523625	6.155
NIAID AIDSno	524038	3.119
NIAID AIDSno	524045	3.262
NIAID AIDSno	524048	4.000
NIAID AIDSno	524063	3.032
NIAID AIDSno	524663	5.736
NIAID AIDSno	525576	8.034
NIAID AIDSno	525958	6.119
NIAID AIDSno	525965	6.233
NIAID AIDSno	526784	4.076
NIAID AIDSno	526785	4.721
NIAID AIDSno	533119	5.409
NIAID AIDSno	533121	5.456
NIAID AIDSno	533122	5.602
NIAID AIDSno	533131	5.420
NIAID AIDSno	533531	5.609
NIAID AIDSno	533535	5.706
NIAID AIDSno	533537	5.752
NIAID AIDSno	533847	7.854

NIAID AIDSno	536091	5.215
NIAID AIDSno	536098	5.215
NIAID AIDSno	536100	4.759
NIAID AIDSno	536103	5.676
NIAID AIDSno	536105	5.092
NIAID AIDSno	536107	5.538
NIAID AIDSno	536108	5.633
NIAID AIDSno	536110	5.357
NIAID AIDSno	536111	5.252
NIAID AIDSno	536119	4.790
NIAID AIDSno	536123	5.469
NIAID AIDSno	540224	4.301
NIAID AIDSno	540451	4.252
NIAID AIDSno	540478	4.911
NIAID AIDSno	540481	4.848
NIAID AIDSno	540484	4.131
NIAID AIDSno	540485	4.653
NIAID AIDSno	540591	4.745
NIAID AIDSno	540592	5.187
NIAID AIDSno	541312	6.222
NIAID AIDSno	541864	5.377
NIAID AIDSno	542430	4.578
NIAID AIDSno	543987	4.149
NIAID AIDSno	544137	3.790
NIAID AIDSno	544139	4.100
NIAID AIDSno	544212	5.498
NIAID AIDSno	544303	5.253
NIAID AIDSno	544306	3.973
NIAID AIDSno	544393	8.523
NIAID AIDSno	544395	8.569
NIAID AIDSno	544398	7.523
NIAID AIDSno	544551	4.092
NIAID AIDSno	544554	5.277
NIAID AIDSno	544555	4.703
NIAID AIDSno	544556	4.483
NIAID AIDSno	544557	4.431
NIAID AIDSno	544558	4.133
NIAID AIDSno	544560	4.227
NIAID AIDSno	544561	5.917
NIAID AIDSno	544562	6.229
NIAID AIDSno	544563	6.387
NIAID AIDSno	544564	6.886
NIAID AIDSno	544565	6.796
NIAID AIDSno	544566	7.481
NIAID AIDSno	544567	7.229

NIAID AIDSno	544569	6.187
NIAID AIDSno	544570	6.924
NIAID AIDSno	544571	8.000
NIAID AIDSno	544788	4.067
NIAID AIDSno	544794	4.163
NIAID AIDSno	544879	7.000
NIAID AIDSno	544880	5.742
NIAID AIDSno	544881	7.155
NIAID AIDSno	544882	5.420
NIAID AIDSno	544885	7.319
NIAID AIDSno	544886	7.538
NIAID AIDSno	545186	5.509
NIAID AIDSno	545187	8.523
NIAID AIDSno	545321	8.481
NIAID AIDSno	545560	5.921
NIAID AIDSno	545572	5.187
NIAID AIDSno	545610	4.595
NIAID AIDSno	545612	6.187
NIAID AIDSno	545622	6.409
NIAID AIDSno	545623	6.921
NIAID AIDSno	545624	6.921
NIAID AIDSno	545625	6.087
NIAID AIDSno	545626	6.025
NIAID AIDSno	545627	7.620
NIAID AIDSno	545628	8.000
NIAID AIDSno	545629	8.000
NIAID AIDSno	545631	7.398
NIAID AIDSno	545634	7.377
NIAID AIDSno	545671	7.569
NIAID AIDSno	545674	8.377
NIAID AIDSno	545675	8.131
NIAID AIDSno	545676	8.066
NIAID AIDSno	545678	7.699
NIAID AIDSno	545679	8.229
NIAID AIDSno	545873	5.633
NIAID AIDSno	545877	7.409
NIAID AIDSno	545880	7.066
NIAID AIDSno	545883	6.190
NIAID AIDSno	545885	6.051
NIAID AIDSno	545892	6.097
NIAID AIDSno	545895	6.184
NIAID AIDSno	545898	7.377
NIAID AIDSno	545905	6.721
NIAID AIDSno	545908	7.260
NIAID AIDSno	545914	6.454

NIAID AIDSno	545932	8.770
NIAID AIDSno	545937	8.137
NIAID AIDSno	545948	8.553
NIAID AIDSno	545954	8.041
NIAID AIDSno	545960	8.770
NIAID AIDSno	545969	7.886
NIAID AIDSno	545982	8.000
NIAID AIDSno	545987	8.523
NIAID AIDSno	545990	8.097
NIAID AIDSno	546022	8.398
NIAID AIDSno	546024	8.699
NIAID AIDSno	546026	8.824
NIAID AIDSno	546029	8.301
NIAID AIDSno	546570	6.914
NIAID AIDSno	547290	5.081
NIAID AIDSno	547307	5.350
NIAID AIDSno	548516	8.509
NIAID AIDSno	548518	8.409
NIAID AIDSno	548520	7.066
NIAID AIDSno	548522	8.319
NIAID AIDSno	548525	8.301
NIAID AIDSno	548527	8.086
NIAID AIDSno	548529	8.260
NIAID AIDSno	548531	8.171
NIAID AIDSno	548535	8.229
NIAID AIDSno	548537	7.921
NIAID AIDSno	548539	8.000
NIAID AIDSno	548542	8.060
NIAID AIDSno	548544	7.886
NIAID AIDSno	548547	7.387
NIAID AIDSno	548549	6.143
NIAID AIDSno	548551	7.678
NIAID AIDSno	548552	8.328
NIAID AIDSno	548553	6.921
NIAID AIDSno	548555	8.284
NIAID AIDSno	548556	8.027
NIAID AIDSno	548558	8.523
NIAID AIDSno	548561	7.959
NIAID AIDSno	548562	8.194
NIAID AIDSno	548563	8.027
NIAID AIDSno	548565	7.886
NIAID AIDSno	548566	8.004
NIAID AIDSno	548567	6.886
NIAID AIDSno	548571	8.538
NIAID AIDSno	548573	7.959

NIAID AIDSno	548622	8.114
NIAID AIDSno	548623	8.495
NIAID AIDSno	548628	8.155
NIAID AIDSno	548630	8.215
NIAID AIDSno	548631	8.585
NIAID AIDSno	548632	7.620
NIAID AIDSno	548634	8.086
NIAID AIDSno	548635	8.155
NIAID AIDSno	548636	8.229
NIAID AIDSno	548638	8.237
NIAID AIDSno	548871	4.097
NIAID AIDSno	548878	4.071
NIAID AIDSno	549556	4.804
NIAID AIDSno	549558	5.329
NIAID AIDSno	549559	5.301
NIAID AIDSno	549562	5.759
NIAID AIDSno	550465	5.492
NIAID AIDSno	550466	5.492
NIAID AIDSno	551126	7.420
NIAID AIDSno	551132	5.492
NIAID AIDSno	551133	5.462
NIAID AIDSno	551135	5.147
NIAID AIDSno	551136	4.509
NIAID AIDSno	551137	5.570
NIAID AIDSno	551142	4.772
NIAID AIDSno	551143	4.759
NIAID AIDSno	551144	4.934
NIAID AIDSno	551146	5.514
NIAID AIDSno	551147	5.281
NIAID AIDSno	551149	5.283
NIAID AIDSno	551151	5.896
NIAID AIDSno	552655	6.580
NIAID AIDSno	553554	6.424
NIAID AIDSno	553555	4.509
NIAID AIDSno	553625	5.618
NIAID AIDSno	553629	6.721
NIAID AIDSno	553631	7.143
NIAID AIDSno	553632	7.398
NIAID AIDSno	553637	6.796
NIAID AIDSno	554099	4.854
NIAID AIDSno	555148	4.699
NIAID AIDSno	555149	4.060
NIAID AIDSno	555152	4.051
NIAID AIDSno	555153	4.377
NIAID AIDSno	555154	4.319

NIAID AIDSno	555155	4.301
NIAID AIDSno	555156	4.284
NIAID AIDSno	555157	4.276
NIAID AIDSno	555158	4.215
NIAID AIDSno	555159	4.076
NIAID AIDSno	555160	4.056
NIAID AIDSno	555162	4.377
NIAID AIDSno	555166	4.208
NIAID AIDSno	555167	4.056
NIAID AIDSno	555168	4.018
NIAID AIDSno	555647	8.975
NIAID AIDSno	556193	6.444
NIAID AIDSno	557506	8.367
NIAID AIDSno	557507	8.222
NIAID AIDSno	560393	5.678
NIAID AIDSno	560399	5.284
NIAID AIDSno	560729	4.293
NIAID AIDSno	560771	4.565
NIAID AIDSno	560780	5.870
NIAID AIDSno	560785	4.375
NIAID AIDSno	560790	4.285
NIAID AIDSno	560791	4.730
NIAID AIDSno	560792	4.823
NIAID AIDSno	560794	6.745
NIAID AIDSno	560795	4.197
NIAID AIDSno	560796	5.408
NIAID AIDSno	560798	4.000
NIAID AIDSno	560799	4.431
NIAID AIDSno	560800	3.747
NIAID AIDSno	560803	7.398
NIAID AIDSno	560804	4.333
NIAID AIDSno	560805	4.395
NIAID AIDSno	560808	3.790
NIAID AIDSno	560809	4.425
NIAID AIDSno	560811	4.152
NIAID AIDSno	560812	6.921
NIAID AIDSno	560813	5.583
NIAID AIDSno	560814	5.991
NIAID AIDSno	560815	4.062
NIAID AIDSno	560816	4.733
NIAID AIDSno	560817	5.517
NIAID AIDSno	560848	6.481
NIAID AIDSno	560851	6.071
NIAID AIDSno	560863	4.318
NIAID AIDSno	560864	4.237

NIAID AIDSno	560934	4.854
NIAID AIDSno	561254	9.000
NIAID AIDSno	561255	8.222
NIAID AIDSno	561259	8.699
NIAID AIDSno	561261	8.301
NIAID AIDSno	561262	8.046
NIAID AIDSno	561263	8.155
NIAID AIDSno	561268	8.155
NIAID AIDSno	561270	8.155
NIAID AIDSno	561271	8.155
NIAID AIDSno	561272	8.523
NIAID AIDSno	561274	8.398
NIAID AIDSno	561275	8.222
NIAID AIDSno	561277	8.301
NIAID AIDSno	561278	7.796
NIAID AIDSno	561279	6.409
NIAID AIDSno	561280	7.959
NIAID AIDSno	561281	6.854
NIAID AIDSno	561344	6.523
NIAID AIDSno	561345	7.367
NIAID AIDSno	561348	7.301
NIAID AIDSno	561349	7.959
NIAID AIDSno	561351	7.678
NIAID AIDSno	561811	4.409
NIAID AIDSno	561812	5.284
NIAID AIDSno	562737	5.161
NIAID AIDSno	562738	5.071
NIAID AIDSno	562748	4.983
NIAID AIDSno	562811	5.646
NIAID AIDSno	564541	8.367
NIAID AIDSno	564542	6.660
NIAID AIDSno	564543	7.770
NIAID AIDSno	564544	6.997
NIAID AIDSno	564545	6.457
NIAID AIDSno	564546	7.003
NIAID AIDSno	564547	7.886
NIAID AIDSno	564920	6.292
NIAID AIDSno	564933	5.810
NIAID AIDSno	564985	6.638
NIAID AIDSno	564991	6.770
NIAID AIDSno	565092	6.719
NIAID AIDSno	565093	6.928
NIAID AIDSno	565094	7.481
NIAID AIDSno	565095	6.907
NIAID AIDSno	565097	8.222

NIAID AIDSno	565098	8.100
NIAID AIDSno	565100	8.071
NIAID AIDSno	565104	6.963
NIAID AIDSno	565106	8.155
NIAID AIDSno	565110	6.907
NIAID AIDSno	565112	7.495
NIAID AIDSno	565117	7.538
NIAID AIDSno	565121	6.963
NIAID AIDSno	565129	7.886
NIAID AIDSno	565133	7.921
NIAID AIDSno	565137	6.747
NIAID AIDSno	565168	8.301
NIAID AIDSno	565169	7.509
NIAID AIDSno	565174	7.620
NIAID AIDSno	565175	7.538
NIAID AIDSno	565176	8.523
NIAID AIDSno	565177	7.620
NIAID AIDSno	565178	7.620
NIAID AIDSno	565179	8.046
NIAID AIDSno	565180	8.301
NIAID AIDSno	565182	7.854
NIAID AIDSno	565183	8.301
NIAID AIDSno	565184	8.523
NIAID AIDSno	565186	8.398
NIAID AIDSno	565187	8.301
NIAID AIDSno	565189	7.886
NIAID AIDSno	565190	8.155
NIAID AIDSno	565191	8.155
NIAID AIDSno	565193	8.097
NIAID AIDSno	565195	8.398
NIAID AIDSno	565198	8.301
NIAID AIDSno	565199	6.959
NIAID AIDSno	565200	8.000
NIAID AIDSno	565201	7.155
NIAID AIDSno	565202	7.229
NIAID AIDSno	565203	8.155
NIAID AIDSno	565204	8.046
NIAID AIDSno	565205	8.398
NIAID AIDSno	565206	8.222
NIAID AIDSno	565208	8.301
NIAID AIDSno	565209	7.959
NIAID AIDSno	565211	8.046
NIAID AIDSno	565213	7.824
NIAID AIDSno	565214	6.783
NIAID AIDSno	565216	6.446

NIAID AIDSno	565218	8.398
NIAID AIDSno	565220	7.155
NIAID AIDSno	565221	8.097
NIAID AIDSno	565222	7.959
NIAID AIDSno	565223	7.119
NIAID AIDSno	565224	7.959
NIAID AIDSno	565226	8.398
NIAID AIDSno	565228	6.903
NIAID AIDSno	565230	8.155
NIAID AIDSno	565232	7.959
NIAID AIDSno	565234	8.000
NIAID AIDSno	565235	8.000
NIAID AIDSno	565236	8.523
NIAID AIDSno	565237	8.301
NIAID AIDSno	565238	8.155
NIAID AIDSno	565239	7.921
NIAID AIDSno	565240	8.155
NIAID AIDSno	565241	7.824
NIAID AIDSno	565243	7.824
NIAID AIDSno	565244	8.301
NIAID AIDSno	565245	8.155
NIAID AIDSno	565246	7.959
NIAID AIDSno	565248	7.770
NIAID AIDSno	565249	7.699
NIAID AIDSno	565250	7.004
NIAID AIDSno	565251	8.415
NIAID AIDSno	565252	8.222
NIAID AIDSno	565253	8.222
NIAID AIDSno	565254	7.854
NIAID AIDSno	565256	8.046
NIAID AIDSno	565257	8.155
NIAID AIDSno	565258	7.854
NIAID AIDSno	565259	7.569
NIAID AIDSno	565260	7.854
NIAID AIDSno	565261	8.222
NIAID AIDSno	565262	7.745
NIAID AIDSno	565263	7.770
NIAID AIDSno	565264	7.745
NIAID AIDSno	565265	8.222
NIAID AIDSno	565266	8.222
NIAID AIDSno	565267	8.222
NIAID AIDSno	565268	7.959
NIAID AIDSno	565269	7.319
NIAID AIDSno	565270	8.097
NIAID AIDSno	565271	6.474

NIAID AIDSno	565272	6.607
NIAID AIDSno	565273	8.046
NIAID AIDSno	565274	8.155
NIAID AIDSno	565275	7.638
NIAID AIDSno	565276	6.815
NIAID AIDSno	565277	7.699
NIAID AIDSno	565278	7.131
NIAID AIDSno	565279	7.824
NIAID AIDSno	565280	7.721
NIAID AIDSno	565281	7.921
NIAID AIDSno	565282	6.500
NIAID AIDSno	565283	6.652
NIAID AIDSno	565284	8.155
NIAID AIDSno	565287	8.523
NIAID AIDSno	565289	8.000
NIAID AIDSno	565291	8.301
NIAID AIDSno	565294	8.222
NIAID AIDSno	565295	8.155
NIAID AIDSno	565296	8.155
NIAID AIDSno	565297	7.678
NIAID AIDSno	565298	8.222
NIAID AIDSno	565299	8.155
NIAID AIDSno	565302	7.921
NIAID AIDSno	565304	8.523
NIAID AIDSno	565307	8.301
NIAID AIDSno	565308	8.222
NIAID AIDSno	565310	8.222
NIAID AIDSno	565311	8.222
NIAID AIDSno	565313	8.097
NIAID AIDSno	565315	8.398
NIAID AIDSno	565316	6.363
NIAID AIDSno	565317	8.446
NIAID AIDSno	565318	8.280
NIAID AIDSno	565319	7.921
NIAID AIDSno	565320	6.362
NIAID AIDSno	565321	7.921
NIAID AIDSno	565322	8.097
NIAID AIDSno	565323	7.770
NIAID AIDSno	565324	8.097
NIAID AIDSno	565327	8.222
NIAID AIDSno	565328	7.959
NIAID AIDSno	565330	7.959
NIAID AIDSno	565332	7.347
NIAID AIDSno	565333	7.444
NIAID AIDSno	565334	7.886

NIAID AIDSno	565336	7.456
NIAID AIDSno	565338	8.155
NIAID AIDSno	565340	8.046
NIAID AIDSno	565342	8.046
NIAID AIDSno	565343	7.721
NIAID AIDSno	565346	8.046
NIAID AIDSno	565351	7.770
NIAID AIDSno	565353	8.046
NIAID AIDSno	565355	8.046
NIAID AIDSno	565358	7.553
NIAID AIDSno	565359	7.495
NIAID AIDSno	565361	7.481
NIAID AIDSno	565362	7.377
NIAID AIDSno	565364	7.027
NIAID AIDSno	565365	7.620
NIAID AIDSno	565367	7.495
NIAID AIDSno	565369	7.959
NIAID AIDSno	565370	7.585
NIAID AIDSno	565371	7.585
NIAID AIDSno	565374	7.620
NIAID AIDSno	565376	7.102
NIAID AIDSno	565379	7.658
NIAID AIDSno	565381	8.155
NIAID AIDSno	565383	8.523
NIAID AIDSno	565385	8.523
NIAID AIDSno	565386	8.222
NIAID AIDSno	565387	8.301
NIAID AIDSno	565388	8.398
NIAID AIDSno	565391	8.301
NIAID AIDSno	565395	8.523
NIAID AIDSno	565401	8.301
NIAID AIDSno	565403	8.222
NIAID AIDSno	565405	8.301
NIAID AIDSno	565406	8.523
NIAID AIDSno	565407	8.222
NIAID AIDSno	565408	7.770
NIAID AIDSno	565409	8.046
NIAID AIDSno	565410	8.097
NIAID AIDSno	565411	8.314
NIAID AIDSno	565412	7.921
NIAID AIDSno	565413	8.046
NIAID AIDSno	565964	5.479
NIAID AIDSno	565967	4.528
NIAID AIDSno	565969	4.802
NIAID AIDSno	565970	4.260

NIAID AIDSno	565971	4.769
NIAID AIDSno	565972	4.470
NIAID AIDSno	565974	5.125
NIAID AIDSno	565979	4.172
NIAID AIDSno	565981	5.854
NIAID AIDSno	565982	4.741
NIAID AIDSno	565985	5.339
NIAID AIDSno	565987	6.229
NIAID AIDSno	565990	5.883
NIAID AIDSno	565991	4.777
NIAID AIDSno	565992	5.578
NIAID AIDSno	565993	4.841
NIAID AIDSno	565997	5.740
NIAID AIDSno	565999	5.263
NIAID AIDSno	566001	5.036
NIAID AIDSno	566003	5.780
NIAID AIDSno	566004	5.260
NIAID AIDSno	566005	4.974
NIAID AIDSno	566006	5.395
NIAID AIDSno	566007	5.348
NIAID AIDSno	566008	4.860
NIAID AIDSno	566009	6.387
NIAID AIDSno	566010	5.439
NIAID AIDSno	566011	5.439
NIAID AIDSno	566012	5.955
NIAID AIDSno	566013	6.149
NIAID AIDSno	566014	5.063
NIAID AIDSno	566015	6.155
NIAID AIDSno	566016	5.162
NIAID AIDSno	566017	5.202
NIAID AIDSno	566018	5.883
NIAID AIDSno	566019	5.592
NIAID AIDSno	566020	5.231
NIAID AIDSno	566021	4.889
NIAID AIDSno	566022	4.744
NIAID AIDSno	566023	4.991
NIAID AIDSno	566024	5.058
NIAID AIDSno	566025	5.824
NIAID AIDSno	567118	7.398
NIAID AIDSno	567621	8.097
NIAID AIDSno	567622	8.097
NIAID AIDSno	567623	7.570
NIAID AIDSno	567624	8.000
NIAID AIDSno	567625	7.824
NIAID AIDSno	567626	7.824

NIAID AIDSno	567627	9.046
NIAID AIDSno	567674	7.854
NIAID AIDSno	567675	7.665
NIAID AIDSno	567676	7.959
NIAID AIDSno	567677	7.319
NIAID AIDSno	567678	8.000
NIAID AIDSno	567679	8.097
NIAID AIDSno	567680	7.658
NIAID AIDSno	567681	7.620
NIAID AIDSno	567682	8.222
NIAID AIDSno	567683	8.301
NIAID AIDSno	567684	8.699
NIAID AIDSno	567685	8.523
NIAID AIDSno	567686	8.398
NIAID AIDSno	567687	9.000
NIAID AIDSno	567688	7.921
NIAID AIDSno	567689	8.523
NIAID AIDSno	567690	7.959
NIAID AIDSno	567691	8.523
NIAID AIDSno	567693	8.398
NIAID AIDSno	567694	8.222
NIAID AIDSno	567695	8.222
NIAID AIDSno	567696	8.523
NIAID AIDSno	567697	8.301
NIAID AIDSno	567870	5.520
NIAID AIDSno	567871	5.006
NIAID AIDSno	567872	4.761
NIAID AIDSno	567875	4.420
NIAID AIDSno	567876	5.514
NIAID AIDSno	567877	5.349
NIAID AIDSno	567878	4.867
NIAID AIDSno	567879	4.851
NIAID AIDSno	567880	4.540
NIAID AIDSno	567922	4.960
NIAID AIDSno	567927	4.581
NIAID AIDSno	567933	4.721
NIAID AIDSno	568140	5.222
NIAID AIDSno	568142	5.301
NIAID AIDSno	568143	5.222
NIAID AIDSno	568144	5.301
NIAID AIDSno	568145	5.301
NIAID AIDSno	568146	5.398
NIAID AIDSno	569150	5.745
NIAID AIDSno	569153	5.434
NIAID AIDSno	569165	6.041

NIAID AIDSno	569169	7.004
NIAID AIDSno	569181	5.943
NIAID AIDSno	569189	6.921
NIAID AIDSno	569199	4.503
NIAID AIDSno	569205	5.958
NIAID AIDSno	569216	5.332
NIAID AIDSno	569220	6.708
NIAID AIDSno	569221	5.014
NIAID AIDSno	569230	5.688
NIAID AIDSno	569235	5.013
NIAID AIDSno	569870	5.014
NIAID AIDSno	569891	5.014
NIAID AIDSno	569902	5.650
NIAID AIDSno	569903	5.695
NIAID AIDSno	569909	5.708
NIAID AIDSno	569911	5.801
NIAID AIDSno	569912	5.733
NIAID AIDSno	569913	5.721
NIAID AIDSno	569928	4.983
NIAID AIDSno	569929	5.000
NIAID AIDSno	569930	5.921
NIAID AIDSno	569944	5.046
NIAID AIDSno	569945	4.699
NIAID AIDSno	569946	5.829
NIAID AIDSno	569947	5.620
NIAID AIDSno	569948	5.149
NIAID AIDSno	569949	5.081
NIAID AIDSno	571152	5.128
NIAID AIDSno	571154	4.509
NIAID AIDSno	571156	4.917
NIAID AIDSno	571158	4.602
NIAID AIDSno	571176	4.585
NIAID AIDSno	572397	6.668
NIAID AIDSno	572560	4.589
NIAID AIDSno	572561	5.383
NIAID AIDSno	572564	4.948
NIAID AIDSno	572565	5.372
NIAID AIDSno	572566	5.889
NIAID AIDSno	572567	4.944
NIAID AIDSno	572568	5.472
NIAID AIDSno	572569	4.962
NIAID AIDSno	572570	5.545
NIAID AIDSno	572571	5.405
NIAID AIDSno	572572	7.097
NIAID AIDSno	572573	5.462

NIAID AIDSno	572574	6.495
NIAID AIDSno	572575	6.284
NIAID AIDSno	572576	6.167
NIAID AIDSno	572577	7.155
NIAID AIDSno	572578	5.724
NIAID AIDSno	572580	6.638
NIAID AIDSno	572581	5.921
NIAID AIDSno	572582	6.770
NIAID AIDSno	572583	6.721
NIAID AIDSno	572584	7.699
NIAID AIDSno	573908	10.432
NIAID AIDSno	573909	10.721
NIAID AIDSno	573910	10.569
NIAID AIDSno	573911	10.456
NIAID AIDSno	573912	10.796
NIAID AIDSno	573913	10.444
NIAID AIDSno	573914	10.569
NIAID AIDSno	573915	10.337
NIAID AIDSno	573917	10.745
NIAID AIDSno	573919	10.347
NIAID AIDSno	573921	10.721
NIAID AIDSno	573922	10.553
NIAID AIDSno	574186	7.721
NIAID AIDSno	574187	7.377
NIAID AIDSno	574208	7.398
NIAID AIDSno	574213	7.194
NIAID AIDSno	574217	7.000
NIAID AIDSno	574252	7.796
NIAID AIDSno	574253	8.523
NIAID AIDSno	574254	7.523
NIAID AIDSno	574255	7.569
NIAID AIDSno	574256	7.155
NIAID AIDSno	574257	7.357
NIAID AIDSno	575116	4.920
NIAID AIDSno	575120	4.818
NIAID AIDSno	575121	6.377
NIAID AIDSno	575123	4.620
NIAID AIDSno	575134	5.089
NIAID AIDSno	575139	4.693
NIAID AIDSno	575141	6.538
NIAID AIDSno	575142	7.051
NIAID AIDSno	575143	4.772
NIAID AIDSno	575144	4.395
NIAID AIDSno	575146	6.167
NIAID AIDSno	575147	4.363

NIAID AIDSno	575148	4.920
NIAID AIDSno	575149	4.243
NIAID AIDSno	575150	4.818
NIAID AIDSno	575151	7.032
NIAID AIDSno	575682	4.107
NIAID AIDSno	575683	4.900
NIAID AIDSno	575684	4.245
NIAID AIDSno	575685	4.257
NIAID AIDSno	575751	4.571
NIAID AIDSno	575752	6.921
NIAID AIDSno	575756	4.622
NIAID AIDSno	575758	4.609
NIAID AIDSno	575762	6.268
NIAID AIDSno	575769	4.723
NIAID AIDSno	575771	7.081
NIAID AIDSno	575772	6.796
NIAID AIDSno	575774	7.337
NIAID AIDSno	575779	4.755
NIAID AIDSno	575780	4.919
NIAID AIDSno	576061	5.770
NIAID AIDSno	576063	5.699
NIAID AIDSno	576064	5.638
NIAID AIDSno	576065	5.420
NIAID AIDSno	576067	5.824
NIAID AIDSno	576069	5.481
NIAID AIDSno	576072	5.638
NIAID AIDSno	576074	5.886
NIAID AIDSno	576082	5.796
NIAID AIDSno	576083	5.824
NIAID AIDSno	576086	5.601
NIAID AIDSno	576090	6.125
NIAID AIDSno	576097	5.602
NIAID AIDSno	576098	5.745
NIAID AIDSno	576099	6.301
NIAID AIDSno	576100	5.585
NIAID AIDSno	576107	5.699
NIAID AIDSno	576110	5.187
NIAID AIDSno	576111	5.721
NIAID AIDSno	576693	4.292
NIAID AIDSno	576698	5.420
NIAID AIDSno	576699	5.721
NIAID AIDSno	576700	5.620
NIAID AIDSno	576701	3.330
NIAID AIDSno	576702	3.836
NIAID AIDSno	577047	4.000

NIAID AIDSno	577049	4.538
NIAID AIDSno	577080	5.883
NIAID AIDSno	578062	6.086
NIAID AIDSno	578065	3.476
NIAID AIDSno	578067	5.244
NIAID AIDSno	578068	4.824
NIAID AIDSno	578070	6.398
NIAID AIDSno	578071	5.328
NIAID AIDSno	578072	6.444
NIAID AIDSno	578073	5.229
NIAID AIDSno	578074	6.585
NIAID AIDSno	578075	4.854
NIAID AIDSno	578076	6.770
NIAID AIDSno	578077	5.260
NIAID AIDSno	578079	6.328
NIAID AIDSno	578080	4.959
NIAID AIDSno	578081	6.387
NIAID AIDSno	578083	5.387
NIAID AIDSno	578084	5.959
NIAID AIDSno	578085	5.886
NIAID AIDSno	578327	5.770
NIAID AIDSno	578330	4.837
NIAID AIDSno	578331	4.908
NIAID AIDSno	578335	5.121
NIAID AIDSno	578336	5.158
NIAID AIDSno	578337	4.696
NIAID AIDSno	578338	4.956
NIAID AIDSno	578339	5.059
NIAID AIDSno	578874	4.959
NIAID AIDSno	578883	4.863
NIAID AIDSno	578886	4.357
NIAID AIDSno	580105	6.701
NIAID AIDSno	580118	6.742
NIAID AIDSno	580135	6.684
NIAID AIDSno	580137	6.690
NIAID AIDSno	580147	6.759
NIAID AIDSno	580148	6.714
NIAID AIDSno	580232	6.807
NIAID AIDSno	580233	6.866
NIAID AIDSno	580234	6.991
NIAID AIDSno	580235	6.936
NIAID AIDSno	580236	6.780
NIAID AIDSno	581368	5.146
NIAID AIDSno	581369	5.040
NIAID AIDSno	581372	5.457

NIAID AIDSno	581373	4.854
NIAID AIDSno	581375	5.216
NIAID AIDSno	581377	5.151
NIAID AIDSno	581511	5.502
NIAID AIDSno	581567	5.818
NIAID AIDSno	581884	6.325
NIAID AIDSno	581902	5.932
NIAID AIDSno	581916	5.557
NIAID AIDSno	582270	5.046
NIAID AIDSno	582946	4.804
NIAID AIDSno	582947	3.876
NIAID AIDSno	582948	6.116
NIAID AIDSno	582949	4.564
NIAID AIDSno	582950	5.332
NIAID AIDSno	582951	4.614
NIAID AIDSno	582952	6.658
NIAID AIDSno	582953	5.735
NIAID AIDSno	582954	3.845
NIAID AIDSno	582955	6.010
NIAID AIDSno	582956	6.143
NIAID AIDSno	582957	4.815
NIAID AIDSno	582958	6.752
NIAID AIDSno	583015	6.037
NIAID AIDSno	583022	6.138
NIAID AIDSno	583023	7.187
NIAID AIDSno	583024	6.529
NIAID AIDSno	583025	5.851
NIAID AIDSno	583026	7.276
NIAID AIDSno	583306	4.328
NIAID AIDSno	583330	4.363
NIAID AIDSno	584411	3.824
NIAID AIDSno	585217	8.481
NIAID AIDSno	585218	8.569
NIAID AIDSno	585551	3.924
NIAID AIDSno	586504	6.030
NIAID AIDSno	586505	6.678
NIAID AIDSno	586506	5.205
NIAID AIDSno	586507	5.740
NIAID AIDSno	586508	6.075
NIAID AIDSno	586509	5.382
NIAID AIDSno	586510	5.451
NIAID AIDSno	586511	5.889
NIAID AIDSno	586512	6.043
NIAID AIDSno	587188	5.592
NIAID AIDSno	587196	5.565

NIAID AIDSno	587394	4.432
NIAID AIDSno	587395	5.090
NIAID AIDSno	587396	4.166
NIAID AIDSno	587397	4.078
NIAID AIDSno	587398	4.044
NIAID AIDSno	587399	4.142
NIAID AIDSno	587400	4.314
NIAID AIDSno	587401	4.624
NIAID AIDSno	587402	4.828
NIAID AIDSno	587403	5.266
NIAID AIDSno	587404	4.409
NIAID AIDSno	587405	4.909
NIAID AIDSno	587413	4.059
NIAID AIDSno	587418	4.364
NIAID AIDSno	587463	5.469
NIAID AIDSno	587484	5.586
NIAID AIDSno	587486	5.376
NIAID AIDSno	587489	6.097
NIAID AIDSno	587492	4.770
NIAID AIDSno	587494	4.921
NIAID AIDSno	587498	5.444
NIAID AIDSno	587500	5.180
NIAID AIDSno	587502	5.187
NIAID AIDSno	587504	5.237
NIAID AIDSno	587505	5.201
NIAID AIDSno	587507	5.481
NIAID AIDSno	587509	5.000
NIAID AIDSno	587510	5.301
NIAID AIDSno	587538	4.854
NIAID AIDSno	587540	5.699
NIAID AIDSno	587541	5.244
NIAID AIDSno	587542	5.509
NIAID AIDSno	587543	6.086
NIAID AIDSno	587544	6.102
NIAID AIDSno	587545	6.081
NIAID AIDSno	587546	5.137
NIAID AIDSno	587608	4.538
NIAID AIDSno	587611	3.690
NIAID AIDSno	587613	3.652
NIAID AIDSno	587637	6.131
NIAID AIDSno	587638	6.086
NIAID AIDSno	587639	5.921
NIAID AIDSno	587640	5.013
NIAID AIDSno	587641	5.102
NIAID AIDSno	587642	5.018

NIAID AIDSno	587643	4.886
NIAID AIDSno	587644	5.432
NIAID AIDSno	587645	6.086
NIAID AIDSno	587646	6.387
NIAID AIDSno	587647	5.706
NIAID AIDSno	587648	3.735
NIAID AIDSno	587649	4.523
NIAID AIDSno	587697	5.201
NIAID AIDSno	587698	6.469
NIAID AIDSno	587699	5.495
NIAID AIDSno	587700	5.770
NIAID AIDSno	587701	5.180
NIAID AIDSno	587702	6.004
NIAID AIDSno	587703	6.035
NIAID AIDSno	587955	4.873
NIAID AIDSno	588158	3.247
NIAID AIDSno	588159	4.620
NIAID AIDSno	588161	4.420
NIAID AIDSno	588162	4.268
NIAID AIDSno	588164	4.921
NIAID AIDSno	588605	4.382
NIAID AIDSno	588607	4.007
NIAID AIDSno	588609	4.513
NIAID AIDSno	588632	3.936
NIAID AIDSno	588633	4.259
NIAID AIDSno	588705	5.071
NIAID AIDSno	588706	4.721
NIAID AIDSno	588707	5.086
NIAID AIDSno	588709	5.658
NIAID AIDSno	588710	5.495
NIAID AIDSno	588711	5.959
NIAID AIDSno	588712	5.921
NIAID AIDSno	588716	5.620
NIAID AIDSno	588806	5.898
NIAID AIDSno	588808	5.625
NIAID AIDSno	588810	5.842
NIAID AIDSno	588811	5.733
NIAID AIDSno	588812	4.331
NIAID AIDSno	588814	4.431
NIAID AIDSno	588815	5.268
NIAID AIDSno	588817	4.353
NIAID AIDSno	588821	6.041
NIAID AIDSno	588823	5.274
NIAID AIDSno	588824	5.378
NIAID AIDSno	588825	4.201

NIAID AIDSno	588826	4.471
NIAID AIDSno	588827	5.095
NIAID AIDSno	588828	4.478
NIAID AIDSno	588832	4.488
NIAID AIDSno	588833	7.000
NIAID AIDSno	588835	4.376
NIAID AIDSno	588836	5.469
NIAID AIDSno	588837	4.439
NIAID AIDSno	588838	4.921
NIAID AIDSno	588849	3.982
NIAID AIDSno	588854	4.344
NIAID AIDSno	588856	7.138
NIAID AIDSno	588919	5.113
NIAID AIDSno	588922	5.361
NIAID AIDSno	589451	4.159
NIAID AIDSno	589452	5.134
NIAID AIDSno	589453	4.726
NIAID AIDSno	589938	4.615
NIAID AIDSno	589939	4.904
NIAID AIDSno	589940	4.546
NIAID AIDSno	589941	4.277
NIAID AIDSno	589942	4.235
NIAID AIDSno	589944	4.324
NIAID AIDSno	589945	4.105
NIAID AIDSno	589946	4.483
NIAID AIDSno	589947	4.731
NIAID AIDSno	589950	4.604
NIAID AIDSno	589951	4.083
NIAID AIDSno	589954	4.313
NIAID AIDSno	589955	4.840
NIAID AIDSno	589958	5.066
NIAID AIDSno	589959	5.022
NIAID AIDSno	590014	4.680
NIAID AIDSno	590022	6.046
NIAID AIDSno	590029	5.921
NIAID AIDSno	590030	6.155
NIAID AIDSno	590031	6.097
NIAID AIDSno	590033	6.222
NIAID AIDSno	590034	6.097
NIAID AIDSno	590035	6.523
NIAID AIDSno	590036	7.000
NIAID AIDSno	590037	6.046
NIAID AIDSno	590038	6.222
NIAID AIDSno	590102	5.824
NIAID AIDSno	590123	5.301

NIAID AIDSno	590125	5.098
NIAID AIDSno	590127	5.041
NIAID AIDSno	590129	4.926
NIAID AIDSno	590130	4.889
NIAID AIDSno	590131	4.866
NIAID AIDSno	590132	4.780
NIAID AIDSno	590135	4.090
NIAID AIDSno	590137	4.068
NIAID AIDSno	590140	3.733
NIAID AIDSno	590142	3.443
NIAID AIDSno	590155	3.491
NIAID AIDSno	590237	4.046
NIAID AIDSno	590238	4.476
NIAID AIDSno	590257	5.821
NIAID AIDSno	590267	4.049
NIAID AIDSno	590268	4.186
NIAID AIDSno	590269	4.139
NIAID AIDSno	590270	4.173
NIAID AIDSno	590271	5.487
NIAID AIDSno	590272	5.198
NIAID AIDSno	590273	5.089
NIAID AIDSno	590274	4.463
NIAID AIDSno	590275	4.407
NIAID AIDSno	590276	6.523
NIAID AIDSno	590277	4.503
NIAID AIDSno	590278	4.815
NIAID AIDSno	590279	4.676
NIAID AIDSno	590280	6.092
NIAID AIDSno	590281	4.584
NIAID AIDSno	590282	5.886
NIAID AIDSno	590306	6.187
NIAID AIDSno	590307	5.907
NIAID AIDSno	590308	5.910
NIAID AIDSno	590309	6.102
NIAID AIDSno	590310	5.975
NIAID AIDSno	590311	6.678
NIAID AIDSno	590312	5.796
NIAID AIDSno	590318	5.472
NIAID AIDSno	590521	5.192
NIAID AIDSno	590686	4.854
NIAID AIDSno	590690	4.921
NIAID AIDSno	590691	4.824
NIAID AIDSno	590692	6.018
NIAID AIDSno	590715	5.201
NIAID AIDSno	590718	6.237

NIAID AIDSno	590733	5.538
NIAID AIDSno	590734	5.854
NIAID AIDSno	590736	4.585
NIAID AIDSno	590737	6.444
NIAID AIDSno	590738	4.237
NIAID AIDSno	591516	5.367
NIAID AIDSno	591981	7.854
NIAID AIDSno	591983	7.602
NIAID AIDSno	591984	7.699
NIAID AIDSno	591986	7.194
NIAID AIDSno	592101	4.349
NIAID AIDSno	592392	7.301
NIAID AIDSno	592393	6.553
NIAID AIDSno	592394	6.886
NIAID AIDSno	592395	6.538
NIAID AIDSno	592396	6.155
NIAID AIDSno	592397	6.310
NIAID AIDSno	592398	6.252
NIAID AIDSno	592399	6.602
NIAID AIDSno	592400	6.237
NIAID AIDSno	592401	6.000
NIAID AIDSno	592488	3.427
NIAID AIDSno	592605	4.687
NIAID AIDSno	592607	4.629
NIAID AIDSno	592608	4.551
NIAID AIDSno	592609	4.789
NIAID AIDSno	592610	4.464
NIAID AIDSno	592611	4.111
NIAID AIDSno	592612	4.416
NIAID AIDSno	592613	4.250
NIAID AIDSno	592614	4.209
NIAID AIDSno	592615	4.502
NIAID AIDSno	592616	4.848
NIAID AIDSno	592617	4.667
NIAID AIDSno	592618	4.912
NIAID AIDSno	592687	5.155
NIAID AIDSno	592699	4.996
NIAID AIDSno	593678	5.939
NIAID AIDSno	593682	6.387
NIAID AIDSno	593683	6.081
NIAID AIDSno	593684	5.857
NIAID AIDSno	593685	4.998
NIAID AIDSno	593686	6.187
NIAID AIDSno	593687	4.998
NIAID AIDSno	593688	5.137

NIAID AIDSno	593689	5.561
NIAID AIDSno	593690	5.551
NIAID AIDSno	593691	5.463
NIAID AIDSno	593692	6.432
NIAID AIDSno	593693	6.201
NIAID AIDSno	593694	6.292
NIAID AIDSno	593695	5.201
NIAID AIDSno	593696	5.860
NIAID AIDSno	593697	6.229
NIAID AIDSno	593706	4.688
NIAID AIDSno	593707	4.538
NIAID AIDSno	593708	4.269
NIAID AIDSno	593709	4.786
NIAID AIDSno	593710	4.529
NIAID AIDSno	593711	4.623
NIAID AIDSno	593712	4.713
NIAID AIDSno	593713	4.390
NIAID AIDSno	593714	4.460
NIAID AIDSno	593715	4.668
NIAID AIDSno	593716	4.658
NIAID AIDSno	593717	4.763
NIAID AIDSno	593718	4.766
NIAID AIDSno	593719	4.514
NIAID AIDSno	593720	4.616
NIAID AIDSno	593721	4.553
NIAID AIDSno	593722	4.631
NIAID AIDSno	593723	5.011
NIAID AIDSno	593724	4.561
NIAID AIDSno	593725	4.473
NIAID AIDSno	593726	4.489
NIAID AIDSno	593727	4.393
NIAID AIDSno	593728	4.396
NIAID AIDSno	593729	4.687
NIAID AIDSno	593730	4.301
NIAID AIDSno	593823	4.004
NIAID AIDSno	593858	4.338
NIAID AIDSno	594106	6.004
NIAID AIDSno	594222	6.310
NIAID AIDSno	594517	5.801
NIAID AIDSno	594566	4.600
NIAID AIDSno	594570	4.740
NIAID AIDSno	594576	5.009
NIAID AIDSno	594577	4.301
NIAID AIDSno	594578	4.108
NIAID AIDSno	594579	4.155

NIAID AIDSno	594580	4.569
NIAID AIDSno	594581	4.947
NIAID AIDSno	594582	4.740
NIAID AIDSno	594583	4.914
NIAID AIDSno	594584	5.796
NIAID AIDSno	594585	5.824
NIAID AIDSno	594586	5.638
NIAID AIDSno	594587	4.276
NIAID AIDSno	594609	6.347
NIAID AIDSno	594610	5.004
NIAID AIDSno	594611	5.638
NIAID AIDSno	594612	5.824
NIAID AIDSno	594613	5.187
NIAID AIDSno	594615	5.155
NIAID AIDSno	594616	5.187
NIAID AIDSno	594619	5.000
NIAID AIDSno	594620	5.000
NIAID AIDSno	594622	7.071
NIAID AIDSno	594623	6.523
NIAID AIDSno	594624	6.658
NIAID AIDSno	594625	6.319
NIAID AIDSno	594626	5.824
NIAID AIDSno	594627	6.086
NIAID AIDSno	594628	6.092
NIAID AIDSno	594631	6.444
NIAID AIDSno	594633	5.167
NIAID AIDSno	594634	5.000
NIAID AIDSno	594635	5.000
NIAID AIDSno	594636	5.000
NIAID AIDSno	594657	5.232
NIAID AIDSno	594660	5.218
NIAID AIDSno	594661	4.598
NIAID AIDSno	594662	5.227
NIAID AIDSno	594864	7.194
NIAID AIDSno	594865	7.174
NIAID AIDSno	594905	5.143
NIAID AIDSno	594906	5.076
NIAID AIDSno	595103	4.757
NIAID AIDSno	595104	4.844
NIAID AIDSno	595105	4.578
NIAID AIDSno	595107	4.056
NIAID AIDSno	595108	4.263
NIAID AIDSno	595110	4.118
NIAID AIDSno	595111	4.602
NIAID AIDSno	595112	5.064

NIAID AIDSno	595113	5.163
NIAID AIDSno	595114	4.416
NIAID AIDSno	595115	4.034
NIAID AIDSno	595116	4.163
NIAID AIDSno	595169	5.396
NIAID AIDSno	595189	6.377
NIAID AIDSno	595201	7.581
NIAID AIDSno	595203	6.051
NIAID AIDSno	595378	5.745
NIAID AIDSno	595379	5.208
NIAID AIDSno	595380	5.092
NIAID AIDSno	595381	5.432
NIAID AIDSno	595382	5.538
NIAID AIDSno	595383	5.678
NIAID AIDSno	595385	4.602
NIAID AIDSno	595386	5.097
NIAID AIDSno	595387	5.131
NIAID AIDSno	595388	5.032
NIAID AIDSno	595389	5.854
NIAID AIDSno	595390	5.770
NIAID AIDSno	595391	6.097
NIAID AIDSno	595392	5.854
NIAID AIDSno	595393	6.398
NIAID AIDSno	595394	5.796
NIAID AIDSno	595395	5.854
NIAID AIDSno	595405	5.000
NIAID AIDSno	595406	5.000
NIAID AIDSno	595407	5.000
NIAID AIDSno	595413	5.092
NIAID AIDSno	595414	5.114
NIAID AIDSno	595416	6.301
NIAID AIDSno	597049	7.638
NIAID AIDSno	597050	7.387
NIAID AIDSno	597051	6.556
NIAID AIDSno	597082	6.695
NIAID AIDSno	597084	6.839
NIAID AIDSno	597090	6.909
NIAID AIDSno	597092	5.591
NIAID AIDSno	597096	6.291
NIAID AIDSno	597100	6.036
NIAID AIDSno	597102	6.893
NIAID AIDSno	597216	4.605
NIAID AIDSno	597217	3.846
NIAID AIDSno	597218	3.990
NIAID AIDSno	597219	3.972

NIAID AIDSno	597455	6.602
NIAID AIDSno	597461	6.602
NIAID AIDSno	597464	6.602
NIAID AIDSno	597466	6.602
NIAID AIDSno	597467	6.602
NIAID AIDSno	597766	4.770
NIAID AIDSno	597805	4.770
NIAID AIDSno	597806	5.886
NIAID AIDSno	597807	5.222
NIAID AIDSno	597808	4.745
NIAID AIDSno	597809	4.678
NIAID AIDSno	597810	6.102
NIAID AIDSno	597811	6.102
NIAID AIDSno	597812	4.119
NIAID AIDSno	597813	3.936
NIAID AIDSno	597814	3.883
NIAID AIDSno	597815	4.022
NIAID AIDSno	597816	4.538
NIAID AIDSno	597817	4.409
NIAID AIDSno	597818	3.662
NIAID AIDSno	597948	11.036
NIAID AIDSno	597949	11.041
NIAID AIDSno	597950	11.056
NIAID AIDSno	597951	11.060
NIAID AIDSno	597952	11.051
NIAID AIDSno	597953	11.201
NIAID AIDSno	597954	11.208
NIAID AIDSno	597955	11.252
NIAID AIDSno	597956	11.276
NIAID AIDSno	597957	11.229
NIAID AIDSno	597965	11.071
NIAID AIDSno	597970	11.102
NIAID AIDSno	597971	11.174
NIAID AIDSno	597972	11.131
NIAID AIDSno	597973	11.086
NIAID AIDSno	597974	11.108
NIAID AIDSno	597975	11.187
NIAID AIDSno	597976	11.149
NIAID AIDSno	597977	11.076
NIAID AIDSno	597978	11.187
NIAID AIDSno	597979	11.161
NIAID AIDSno	597980	11.301
NIAID AIDSno	597981	11.387
NIAID AIDSno	597982	11.367
NIAID AIDSno	597983	11.310

NIAID AIDSno	597984	11.432
NIAID AIDSno	597985	11.328
NIAID AIDSno	597986	11.357
NIAID AIDSno	597987	11.495
NIAID AIDSno	597988	11.469
NIAID AIDSno	598057	5.432
NIAID AIDSno	598058	5.745
NIAID AIDSno	598059	5.745
NIAID AIDSno	598060	5.921
NIAID AIDSno	598210	4.975
NIAID AIDSno	598211	4.860
NIAID AIDSno	598696	6.721
NIAID AIDSno	598697	5.585
NIAID AIDSno	598698	5.959
NIAID AIDSno	598699	5.432
NIAID AIDSno	598875	6.553
NIAID AIDSno	598918	6.187
NIAID AIDSno	598919	6.770
NIAID AIDSno	598920	7.046
NIAID AIDSno	598921	7.301
NIAID AIDSno	598922	6.041
NIAID AIDSno	598923	3.836
NIAID AIDSno	598925	6.509
NIAID AIDSno	598926	6.585
NIAID AIDSno	598927	6.796
NIAID AIDSno	598928	6.770
NIAID AIDSno	598998	5.910
NIAID AIDSno	599001	5.171
NIAID AIDSno	599002	5.081
NIAID AIDSno	599003	5.010
NIAID AIDSno	599004	6.444
NIAID AIDSno	599005	5.327
NIAID AIDSno	599006	6.187
NIAID AIDSno	599007	4.631
NIAID AIDSno	599008	5.469
NIAID AIDSno	599009	5.721
NIAID AIDSno	599010	5.328
NIAID AIDSno	599011	5.513
NIAID AIDSno	599012	5.896
NIAID AIDSno	599684	5.071
NIAID AIDSno	599685	7.284
NIAID AIDSno	599686	6.036
NIAID AIDSno	599687	5.824
NIAID AIDSno	599688	5.959
NIAID AIDSno	599690	6.268

NIAID AIDSno	599691	6.678
NIAID AIDSno	599693	6.699
NIAID AIDSno	599694	6.444
NIAID AIDSno	599695	6.347
NIAID AIDSno	599819	7.620
NIAID AIDSno	599820	7.310
NIAID AIDSno	599821	7.149
NIAID AIDSno	599822	7.886
NIAID AIDSno	599823	6.883
NIAID AIDSno	599824	7.310
NIAID AIDSno	599825	7.796
NIAID AIDSno	599826	7.114
NIAID AIDSno	599827	7.658
NIAID AIDSno	599828	7.745
NIAID AIDSno	599829	7.538
NIAID AIDSno	599830	7.678
NIAID AIDSno	599831	7.469
NIAID AIDSno	599832	7.444
NIAID AIDSno	599833	6.674
NIAID AIDSno	599834	5.572
NIAID AIDSno	599835	6.730
NIAID AIDSno	599836	6.740
NIAID AIDSno	599837	6.623
NIAID AIDSno	599838	6.900
NIAID AIDSno	599904	4.873
NIAID AIDSno	599905	4.963
NIAID AIDSno	599906	4.988
NIAID AIDSno	599909	5.459
NIAID AIDSno	599910	5.445
NIAID AIDSno	599911	6.161
NIAID AIDSno	599916	6.095
NIAID AIDSno	600084	7.796
NIAID AIDSno	600086	7.854
NIAID AIDSno	600087	7.959
NIAID AIDSno	600088	7.585
NIAID AIDSno	600089	7.699
NIAID AIDSno	600090	7.699
NIAID AIDSno	600091	7.770
NIAID AIDSno	600092	7.921
NIAID AIDSno	600093	8.046
NIAID AIDSno	600094	7.721
NIAID AIDSno	600095	7.745
NIAID AIDSno	600096	7.745
NIAID AIDSno	600097	7.569
NIAID AIDSno	600098	7.569

NIAID AIDSno	600099	7.678
NIAID AIDSno	600100	7.174
NIAID AIDSno	600101	7.237
NIAID AIDSno	600102	7.337
NIAID AIDSno	600103	7.432
NIAID AIDSno	600104	6.936
NIAID AIDSno	600105	7.337
NIAID AIDSno	600106	7.284
NIAID AIDSno	600116	3.899
NIAID AIDSno	600120	3.694
NIAID AIDSno	600512	6.654
NIAID AIDSno	600513	4.671
NIAID AIDSno	600514	4.805
NIAID AIDSno	600515	4.217
NIAID AIDSno	600516	5.115
NIAID AIDSno	600517	7.041
NIAID AIDSno	600518	6.886
NIAID AIDSno	600519	7.056
NIAID AIDSno	600520	7.328
NIAID AIDSno	600521	7.301
NIAID AIDSno	600522	4.803
NIAID AIDSno	600523	4.325
NIAID AIDSno	600524	5.264
NIAID AIDSno	600525	6.119
NIAID AIDSno	600526	5.234
NIAID AIDSno	600551	3.566
NIAID AIDSno	600554	4.194
NIAID AIDSno	600577	4.593
NIAID AIDSno	600578	4.910
NIAID AIDSno	600580	4.706
NIAID AIDSno	600581	4.036
NIAID AIDSno	600583	5.013
NIAID AIDSno	600585	5.708
NIAID AIDSno	600586	5.466
NIAID AIDSno	600588	6.119
NIAID AIDSno	600589	5.879
NIAID AIDSno	601381	5.070
NIAID AIDSno	601383	4.389
NIAID AIDSno	601384	4.440
NIAID AIDSno	601385	4.507
NIAID AIDSno	601386	4.480
NIAID AIDSno	601387	4.556
NIAID AIDSno	601388	4.610
NIAID AIDSno	601534	7.796
NIAID AIDSno	601535	6.578

NIAID AIDSno	601578	4.337
NIAID AIDSno	601579	4.013
NIAID AIDSno	601580	4.658
NIAID AIDSno	601581	4.854
NIAID AIDSno	601582	5.796
NIAID AIDSno	601583	5.807
NIAID AIDSno	601586	4.699
NIAID AIDSno	601588	4.387
NIAID AIDSno	601589	4.187
NIAID AIDSno	601590	4.310
NIAID AIDSno	601592	4.602
NIAID AIDSno	601593	4.208
NIAID AIDSno	602791	5.569
NIAID AIDSno	602792	5.804
NIAID AIDSno	604547	7.260
NIAID AIDSno	604548	7.086
NIAID AIDSno	604549	7.284
NIAID AIDSno	604550	7.276
NIAID AIDSno	604551	7.658
NIAID AIDSno	604552	6.656
NIAID AIDSno	604554	6.714
NIAID AIDSno	604555	6.420
NIAID AIDSno	604556	6.710
NIAID AIDSno	604557	7.125
NIAID AIDSno	604558	7.009
NIAID AIDSno	604559	6.967
NIAID AIDSno	604560	6.788
NIAID AIDSno	604561	6.724
NIAID AIDSno	604562	6.801
NIAID AIDSno	604572	6.796
NIAID AIDSno	604573	6.066
NIAID AIDSno	604574	6.553
NIAID AIDSno	604575	6.538
NIAID AIDSno	604820	4.809
NIAID AIDSno	604821	5.282
NIAID AIDSno	604822	5.860
NIAID AIDSno	604823	6.086
NIAID AIDSno	604824	5.693
NIAID AIDSno	604825	4.490
NIAID AIDSno	604826	4.083
NIAID AIDSno	604827	4.205
NIAID AIDSno	604828	4.157
NIAID AIDSno	604830	5.228
NIAID AIDSno	604831	4.564
NIAID AIDSno	604832	6.569

NIAID AIDSno	604833	6.119
NIAID AIDSno	604834	4.465
NIAID AIDSno	604835	4.207
NIAID AIDSno	604836	4.233
NIAID AIDSno	604837	4.162
NIAID AIDSno	604859	6.194
NIAID AIDSno	604861	5.155
NIAID AIDSno	604869	5.000
NIAID AIDSno	604870	5.495
NIAID AIDSno	604871	5.155
NIAID AIDSno	604872	5.076
NIAID AIDSno	604875	5.000
NIAID AIDSno	604878	5.000
NIAID AIDSno	604879	5.000
NIAID AIDSno	604880	5.000
NIAID AIDSno	604883	5.167
NIAID AIDSno	604884	5.000
NIAID AIDSno	604885	5.114
NIAID AIDSno	604886	5.004
NIAID AIDSno	604887	5.018
NIAID AIDSno	604888	5.108
NIAID AIDSno	604889	5.252
NIAID AIDSno	604892	5.301
NIAID AIDSno	604893	6.337
NIAID AIDSno	605050	7.886
NIAID AIDSno	605051	7.398
NIAID AIDSno	605053	6.648
NIAID AIDSno	605056	6.219
NIAID AIDSno	605057	7.180
NIAID AIDSno	605059	7.125
NIAID AIDSno	605061	7.076
NIAID AIDSno	605064	6.393
NIAID AIDSno	605066	6.064
NIAID AIDSno	605072	6.435
NIAID AIDSno	605075	6.804
NIAID AIDSno	605086	7.357
NIAID AIDSno	605094	6.527
NIAID AIDSno	605096	7.638
NIAID AIDSno	605097	7.699
NIAID AIDSno	605098	7.620
NIAID AIDSno	605099	7.495
NIAID AIDSno	605100	7.523
NIAID AIDSno	605101	7.796
NIAID AIDSno	605102	7.678
NIAID AIDSno	605103	7.585

NIAID AIDSno	605446	5.000
NIAID AIDSno	605753	4.514
NIAID AIDSno	605758	4.403
NIAID AIDSno	605770	4.551
NIAID AIDSno	605823	6.943
NIAID AIDSno	605824	5.461
NIAID AIDSno	605825	4.695
NIAID AIDSno	605826	6.489
NIAID AIDSno	605827	6.281
NIAID AIDSno	605829	6.353
NIAID AIDSno	606008	5.284
NIAID AIDSno	606009	4.034
NIAID AIDSno	606010	4.143
NIAID AIDSno	606011	4.521
NIAID AIDSno	606012	5.896
NIAID AIDSno	606013	4.198
NIAID AIDSno	606014	3.876
NIAID AIDSno	606015	4.488
NIAID AIDSno	606016	5.903
NIAID AIDSno	606017	4.712
NIAID AIDSno	606018	6.282
NIAID AIDSno	606019	5.676
NIAID AIDSno	606020	6.032
NIAID AIDSno	606051	6.678
NIAID AIDSno	606052	6.717
NIAID AIDSno	606057	7.102
NIAID AIDSno	606059	6.647
NIAID AIDSno	606076	3.944
NIAID AIDSno	606081	4.197
NIAID AIDSno	606106	4.395
NIAID AIDSno	606107	4.226
NIAID AIDSno	606108	5.011
NIAID AIDSno	606121	4.048
NIAID AIDSno	606122	4.063
NIAID AIDSno	606123	4.000
NIAID AIDSno	606124	4.149
NIAID AIDSno	606150	4.107
NIAID AIDSno	606180	4.292
NIAID AIDSno	606181	4.108
NIAID AIDSno	606182	4.409
NIAID AIDSno	606186	4.252
NIAID AIDSno	606187	5.041
NIAID AIDSno	606191	4.431
NIAID AIDSno	606192	4.092
NIAID AIDSno	606193	4.266

NIAID AIDSno	606194	4.676
NIAID AIDSno	606195	4.172
NIAID AIDSno	606197	4.565
NIAID AIDSno	606198	5.538
NIAID AIDSno	606199	4.741
